# Evaluation of Head Movement Periodicity and Irregularity during Locomotion of *Caenorhabditis elegans*

**DOI:** 10.3389/fnbeh.2013.00020

**Published:** 2013-03-21

**Authors:** Ryuzo Shingai, Morimichi Furudate, Katsunori Hoshi, Yuishi Iwasaki

**Affiliations:** ^1^Laboratory of Bioscience, Faculty of Engineering, Iwate UniversityMorioka, Iwate, Japan; ^2^Department of Applied Chemistry and Bioengineering, Faculty of Engineering, Iwate UniversityMorioka, Iwate, Japan; ^3^Department of Intelligent Systems Engineering, College of Engineering, Ibaraki UniversityHitachi, Ibaraki, Japan

**Keywords:** nematode, behavior, image analysis, mutant, senescence

## Abstract

*Caenorhabditis elegans* is suitable for studying the nervous system, which controls behavior. *C. elegans* shows sinusoidal locomotion on an agar plate. The head moves not only sinusoidally but also more complexly, which reflects regulation of the head muscles by the nervous system. The head movement becomes more irregular with senescence. To date, the head movement complexity has not been quantitatively analyzed. We propose two simple methods for evaluation of the head movement regularity on an agar plate using image analysis. The methods calculate metrics that are a measure of how the head end movement is correlated with body movement. In the first method, the length along the trace of the head end on the agar plate between adjacent intersecting points of the head trace and the quasi-midline of the head trace, which was made by sliding an averaging window of 1/2 the body wavelength, was obtained. Histograms of the lengths showed periodic movement of the head and deviation from it. In the second method, the intersections between the trace of the head end and the trace of the 5 (near the pharynx) or 50% (the mid-body) point from the head end in the centerline length of the worm image were marked. The length of the head trace between adjacent intersections was measured, and a histogram of the lengths was produced. The histogram for the 5% point showed deviation of the head end movement from the movement near the pharynx. The histogram for the 50% point showed deviation of the head movement from the sinusoidal movement of the body center. Application of these methods to wild type and several mutant strains enabled evaluation of their head movement periodicity and irregularity, and revealed a difference in the age-dependence of head movement irregularity between the strains. A set of five parameters obtained from the histograms reliably identifies differences in head movement between strains.

## Introduction

*Caenorhabditis elegans* is an excellent model for studying behavior regulation by the nervous system, because the worm has a simple nervous system and shows various behaviors, and a large amount of genetic information concerning its neural functions is available. Almost all behaviors are performed by undulatory movements. In many cases, behavior analyses are conducted on agar plates. To date, locomotion phenotypes, in relation to the nervous system, have been studied in terms of moving speed, body bending frequency, and duration or frequency of forward/backward movements in wild type worms, or in comparison to various mutants or worms with laser-ablated neurons (Horvitz et al., 1982; Ségalat et al., 1995; Zheng et al., 1999; Brockie et al., 2001; Hardaker et al., 2001; Tsalik and Hobert, 2003; Hills et al., 2004; Wakabayashi et al., 2004; Gray et al., 2005), and in senescent worms (Bolanowski et al., 1981; Duhon and Johnson, 1995; Glenn et al., 2004; Hsu et al., 2009). Locomotion phenotypes are affected by neurotransmitters and their receptors, such as amino acids and their receptors (Zheng et al., 1999; Brockie et al., 2001; Hills et al., 2004; Dittman and Kaplan, 2008), and monoamines (Horvitz et al., 1982; Ségalat et al., 1995; Wakabayashi et al., 2005). Computer analysis of worm images is an indispensable tool for phenotypic analysis (Dusenbery, 1985; Shingai, 2000; Baek et al., 2002; Geng et al., 2004; Cronin et al., 2005; Hoshi and Shingai, 2006; Karbowski et al., 2006, 2008; Buckingham and Sattelle, 2008; Braun et al., 2010; Maguire et al., 2011). In the moving worm, the head moves not only sinusoidally but also more complexly, which reflects regulation of the neuromuscular system of the head. For example, several mutants concerning neural functions show different body bending angles in locomotion compared with the wild type (Karbowski et al., 2008). Moreover, the head movement depends on the developmental stage, and it becomes more irregular with senescence. Therefore, the head movement complexity is an important index for evaluation of both behavior and nervous system functions, but its characteristics have rarely been quantified. Here, we propose two simple methods for quantification of the head movement periodicity and irregularity, which we applied at various stages of senescence of wild type and mutants to evaluate the differences between their head movements.

## Materials and Methods

### *C. elegans* strains

Wild type (N2 Bristol), CB156*unc-25*(*e156*)III, CB382*unc-49*(*e382*)III, KP4*glr-1*(*n2461*)III, VM487*nmr-1*(*ak4*)II, CB1111*cat-1*(*e1111*)X, CB1112*cat-2*(*e1112*)II, and GR1321*tph-1*(*mg280*)II were obtained from the *Caenorhabditis* Genetic Center, University of Minnesota, and grown on nematode growth medium (NGM) agar plates carrying a lawn of OP50 *Escherichia coli* (Brenner, 1974), at 20°C. The deficiencies in these mutants are listed in Table 1.

**Table 1:** **Mutants used**.

Strain	Deficiency
*unc-25(e156)*	Glutamic acid decarboxylase, GABA biosynthetic enzyme
*unc-49(e382)*	Ionotropic GABA_A_ receptor subunit
*glr-1(n2461)*	AMPA-type ionotropic glutamate receptor subunit
*nmr-1(ak4)*	NMDA-type ionotropic glutamate receptor subunit
*cat-1(e1111)*	Synaptic vesicular monoamine transporter required for the presence of dopamine and serotonin in nerve terminals
*cat-2(e1112)*	Tyrosine hydroxylase, the rate-limiting enzyme in the synthesis of catecholamines, such as dopamine
*tph-1(mg280)*	Tryptophan hydroxylase, the enzyme that catalyzes the rate-limiting first step in serotonin biosynthesis

*List of mutant strains and their deficiencies. Deficiency descriptions are from wormbase (http://www.wormbase.org/)*.

### Synchronous culture and determination of age

Synchronously growing nematodes were prepared as follows: 10 gravid adult worms were transferred to a fresh NGM plate (3 g/L NaCl, 2.5 g/L polypeptone, 5 mg/L cholesterol, 1 mM CaCl_2_, 1 mM MgSO_4_, 25 mM KH_2_PO_4_, pH 6.0 adjusted with KOH, and 17 g/L agar) carrying an OP50 lawn for 2 h to allow the nematodes to lay eggs. Next, the adult nematodes were removed from the NGM plates and the plates containing the eggs were kept at 20°C. The end of the L4 larval stage was determined by cessation of pharyngeal pumping in the molt between the L4 stage and the young adult stage. The beginning of the young adult stage was determined as follows: the crescent-shaped structure at the vulva disappeared and the pumping of the pharynx started again. The young adult stage was named A1; the succeeding stages occurred every 24 h and were designated A2–A10. Every 2 days, at A1, A3, A5, and A7, adult worms were transferred to freshly seeded plates to discriminate from worms of other generations.

### Locomotion recording

The entire procedure was conducted at 20°C. A single nematode was transferred to a drop of approximately 20 μL of phosphate buffer (1 mM CaCl_2_, 1 mM MgSO_4_, 5 mM KH_2_PO_4_, pH 6.0 adjusted with KOH, and 0.05% Tween20) on a bacteria-free agar plate, using a platinum wire to remove bacteria attached to the cuticle, and after about 1 min the worm was transferred to a bacteria-free assay plate (15 cm diameter) containing 30 mL of agar medium (1 mM CaCl_2_, 1 mM MgSO_4_, 5 mM KH_2_PO_4_, pH 6.0 adjusted with KOH, 15g/L agar). Agar (BA-10) was obtained from Funakoshi Co. (Tokyo, Japan) and the other chemicals were from Wako Co. (Osaka, Japan). All reagents used were of the highest grade available. The behavior of the nematodes, as viewed through a dissection microscope lens and a CCD camera (C2400-77, Hamamatsu Photonics K.K., Hamamatsu, Japan), was recorded on a computer hard disk for 10 or 30 min at 3 images/s, starting 40 min after the worm was transferred to the assay plate, because when a worm is placed on an assay plate, it initially shows frequent changes in locomotion states (Hills et al., 2004; Wakabayashi et al., 2004; Gray et al., 2005). The method to determine the duration of recording and the number of worms is shown at the end of Section “Regularity of Trace of Head Swing.”

### Regularity of trace of head swing

**Figure 1:**
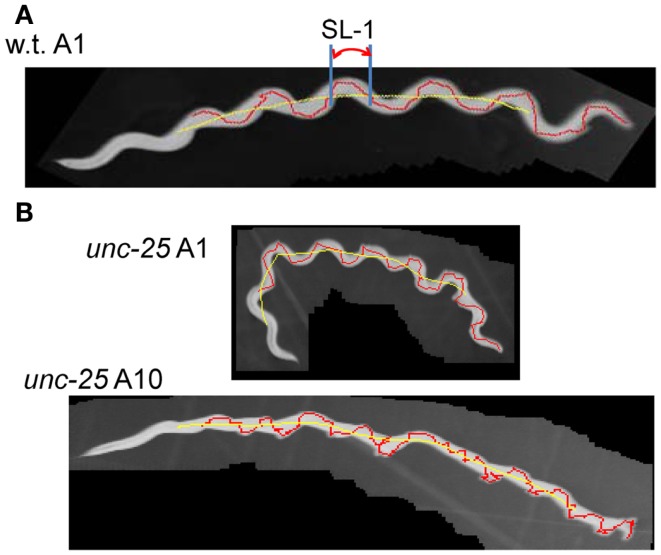
**Trace of head and Type-1 segment-length**. **(A)** Superimposed images (gray) of the wild type worm at the A1 stage with the trace of the head movement (red) and the quasi-midline (yellow). A Type-1 segment-length is indicated by SL-1. **(B)** Superimposed worm images (gray) of an *unc-25*(*e156*) mutant worm at the A1 and A10 stages with the head trace (red) and the quasi-midline (yellow). **(A,B)** The worms moved from the left to the right.

**Figure 2:**
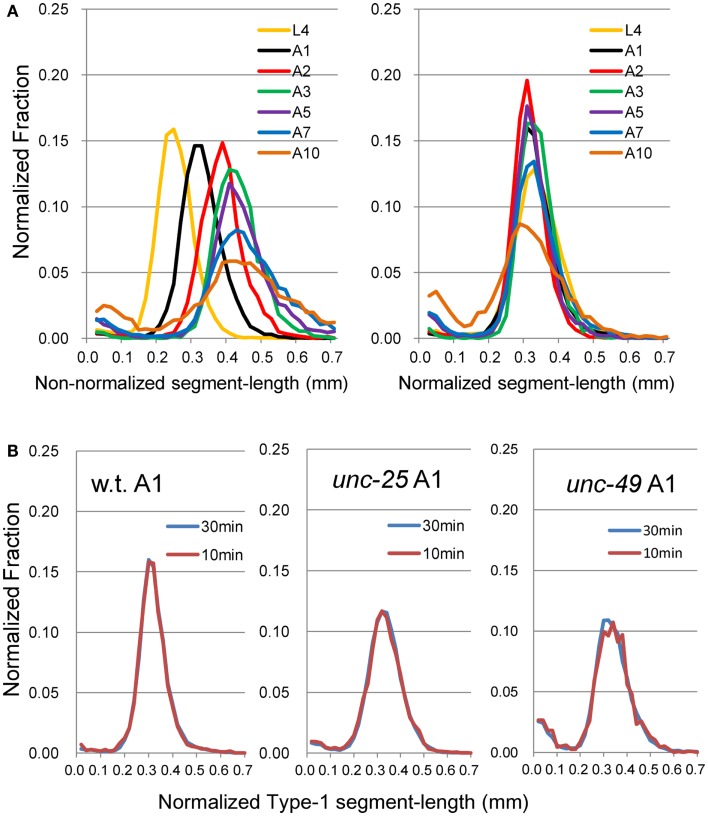
**Histograms of Type-1 segment-length**. **(A)** (Left) Histogram of Type-1 segment-length without normalization by the length of each worm. (Right) nSL-1 histogram after normalization of the length of the worm to 1 mm. The worms are wild type at the L4–A10 stages. Numbers of worms are listed in Table 2. **(B)** Comparison of histogram made from the 30-min data set with that from the 10-min data set, which was taken from the initial part of the 30-min data set of wild type, *unc-25*, and *unc-49* at the A1 stage. There was no statistical difference between the histograms of the 10 and 30-min data sets.

Forward movement was discriminated from three other locomotion states (backward movement, rest, and curl) as described previously (Hoshi and Shingai, 2006). The software described in Sections “Regularity of Trace of Head Swing” and “Small Head Movements and Deviation from Sinusoidal Body Movement” was developed in our laboratory using the programing language Microsoft Visual Basic 2010 Express, and shown in Softwares S1–S4 in Supplementary Material. Periodicity and irregularity of the head swing during forward movement was determined as follows. An image of 1 mm scale was used to determine the conversion factor between millimeter-based length and pixel-based length (2^1/2^ pixel in the case of adjacent two points in diagonal direction). Two-valued (binary) images of a worm were extracted using a threshold value, and thinned to obtain the centerline of the worm’s image. The trace of the head on the agar plate was obtained by connecting the coordinates of the head end in a sequence of the worm’s centerlines, which formed a polygonal line. A quasi-midline (the yellow line in Figure 1A) of the head trace was obtained by taking, for every point *x* on the head trace, the average of the coordinates of the points with *Dx* intervals: *x* − (BL/4), …, *x* − *Dx*, *x*, *x* + *Dx*, …, *x* + (BL/4) where BL is the worm’s centerline length and *Dx* is the length between adjacent points on the trace. The value *Dx* can be set arbitrarily, but was usually the length of adjacent pixels on the head trace (i.e., the shortest length). The pixel-based length of the head trace between adjacent intersecting points of the head trace and the quasi-midline (“SL-1” in Figure 1A) was obtained and converted to millimeter-based length using the abovementioned conversion factor. The resulting length was termed “Type-1 segment-length.” To avoid the effect of the worm’s length, the average length of the centerlines of one worm was regarded as 1 mm, and the Type-1 segment-length of the worm was normalized using this conversion factor. The histograms of the Type-1 segment-length of all worms of a particular age were added, and a combined histogram was made; the sum of all segment-length fractions in the histogram was normalized to 1. The bin width of the histogram’s abscissa was 0.02 mm. The histogram of the Type-1 segment-length normalized by both the length of the centerline and the sum of all segment-length fractions was abbreviated to nSL-1 histogram (Figure 2A). For comparison, the nSL-1 histogram of the middle point of the worm’s body in *unc-25* was obtained in the same manner. The height of the histogram peak is a measure of periodicity, while short segment-length fraction (<0.18 mm) shows irregular head movement. The 0.18 mm threshold was determined by inspection of the nSL-1 histogram of the wild type worms.

We recorded images of the wild type and GABAergic mutants *unc-25* and *unc-49* for 30 min to obtain sufficient data, and compared the Type-1 normalized histogram made by the whole 30-min data set with that made by the initial 10-min data set. We found that the two histograms for the 10 and 30 min data sets were almost identical (Figure 2B), and there was no statistical difference between the peak values of the histograms (*p* = 0.58, 0.25, and 0.21 by *t*-test for wild type, *unc-25*, and *unc-49*, respectively), or between the fractions <0.18 mm (*p* = 0.43, 0.05, and 0.09 by *t*-test). Therefore, we considered that 10-min observations were sufficient. The 10-min data set included, in total, 1,986, 3,823, and 1,165 Type-1 segments in 12 of the wild type, 18 *unc-25*, and 10 *unc-49* worms, respectively. Data from the 30-min observations were used for these three strains. For other strains, we used 10-min observations from many worms rather than 30 min observations on a small number of worms, because differences among single worms existed, especially in mutants, potentially because there is an age-dependent change within 1 day. The 10 min data set from 20 *tph-1* worms at the A1 stage included 1,944 Type-1 segments, which was the smallest data size among the strains at A1.

### Small head movements and deviation from sinusoidal body movement

Although every point on the worm’s body depicts undulatory locomotion on the agar plate, the head moves more complexly than other body parts during forward movement in adult stages. Deviation of the head movement from the body movement was evaluated in the following manner. The intersections between the trace of the head end and the trace of the 5 or 50% point from the head end of the centerline length of the worm image were marked. The length of the head trace between the adjacent intersections was measured, and a histogram of the lengths was produced. The histogram for the 5% point showed small head movements, and the histogram for the 50% point showed deviation of the head movement from the sinusoidal movement of the body center. This length was named “Type-2 segment-length (5 or 50%),” and normalized so that the length of the worm was 1 mm. The histogram of the Type-2 segment-length normalized to 1 by the sum of all the fractions was named nSL-2 histogram (5 or 50%).

### Statistical difference

The number of worms of each strain at each age is listed in Table 2. To examine the statistical differences in the nSL-1 histograms or nSL-2 histograms between the different strains, an nSL-1 histogram or nSL-2 histogram was obtained for each worm, and the average of the values at each bin of the histograms was determined. Student’s *t*-test was used to compare the peak values or the short segment-length fraction (<0.18 mm) of the histograms between the strains at the same age. The *t*-test was also used to compare the normalized Type-2 segment-lengths at the peak of nSL-2 (5%) between the strains at the same age.

**Table 2:** **Number of worms used in analysis**.

	L4	A1	A2	A3	A5	A7	A10
w.t.	19	12	10	10	13	25	11
*unc-25*	14	18	10	10	17	23	15
*unc-49*	19	10	10	10	10	18	15
*cat-1*		19		20	27	21	9
*cat-2*		20		19	20	20	15
*glr-1*		20		19	19	18	13
*nmr-1*		19		20	20	20	14
*tph-1*		20		18	12	9	9

## Results

### Age-dependent changes in regularity and irregularity of head swing

The proposed methods were applied to the wild type worm and several mutants. Figures 1A,B show superimposed images of the wild type worm during forward movement as well as of the *unc-25*(*e156*) mutant, the traces of the head ends, the quasi-midlines, and a Type-1 segment-length. Figure 2A (left panel) shows the histograms of the segment-length of the wild type at various ages without length normalization. The histogram shifted to the right as age progressed from L4 larva to A10, which reflects the growth of worms with age. However, when the length of each worm was normalized to 1 mm (Figure 2A, right panel), the position of the histogram peaks in the abscissa was similar, although the histogram peak decreased with age. This indicates that the sinusoidal curves depicted by the head during forward locomotion had similar values when normalized by the length of the worm, regardless of age. The peak of the histogram corresponded to the sinusoidal head swing, while the short segment-length fraction (<0.18 mm) corresponded to small irregular head swings that deviated from the sinusoidal movement.

**Figure 3:**
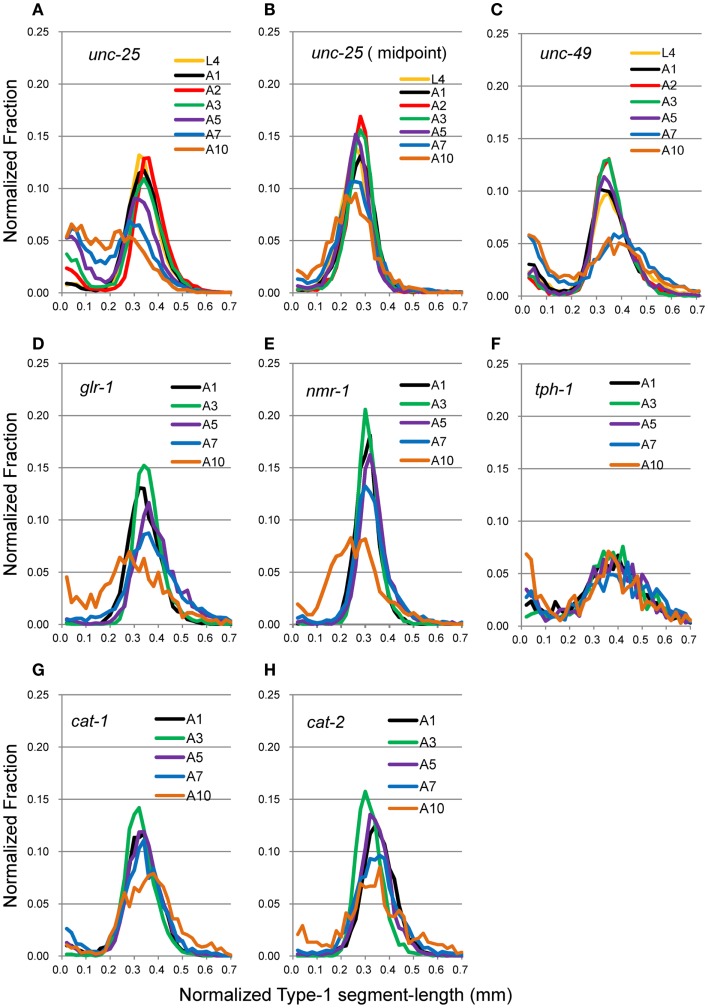
**nSL-1 histograms of mutant strains at various ages**. **(A,B)** *unc-25*(*e156*). **(C)** *unc-49*(*e382*). **(D)** *glr-1*(*n2461*). **(E)** *nmr-1*(*ak4*). **(F)** *tph-1*(*mg280*). **(G)** *cat-1*(*e1111*). **(H)** *cat-2*(*e1112*). **(A–C)** Ages between L4 and A10. **(D–H)** Ages between A1 and A10. **(B)** Histogram of the middle point of the body of *unc-25*(*e156*). All the other histograms are of the head end. Numbers of worms are listed in Table 2.

Head movement during locomotion changed with age (Figure 2A). Figure 2A (right panel) and Figure 3 show nSL-1 histograms of several strains at different ages. The nSL-1 histograms of individual worms are shown in Figure S1 in Supplementary Material. When the wild type was examined (Figure 2A) the peak was highest at A2, and then decreased as the GABA-deficient *unc-25*(*e156*) and ionotropic GABA receptor-defective *unc-49*(*e382*) mutants (Figures 3A,C). The short segment-length fraction (<0.18 mm) in *unc-25* mutants increased considerably with age compared with *unc-49*, indicating that loss of GABA induces more severe deterioration of head swing with age compared with the defect in ionotropic GABA receptor UNC-49. However, the nSL-1 histogram of the body midpoint of *unc-25* (Figure 3B) had a higher peak and a smaller short-length fraction than the head in each stage, indicating that the body showed more regular sinusoidal movement than the head in each stage. Among the monoaminergic mutants (Figures 3F–H), the peak of tryptophan hydroxylase defective mutant *tph-1*(*mg280*) was low, with a long tail at the longer nSL-1 side, and the histograms hardly changed with age between A1 and A10. Therefore, serotonin deficiency in *tph-1*(*mg280*) induced more irregular head swing than the deficiency in synaptic vesicular monoamine transporter in *cat-1*(*e1111*) and in dopamine in *cat-2*(*e1112*). The histograms of *cat-1*(*e1111*) and *cat-2*(*e1112*) showed similar patterns.

**Figure 4:**
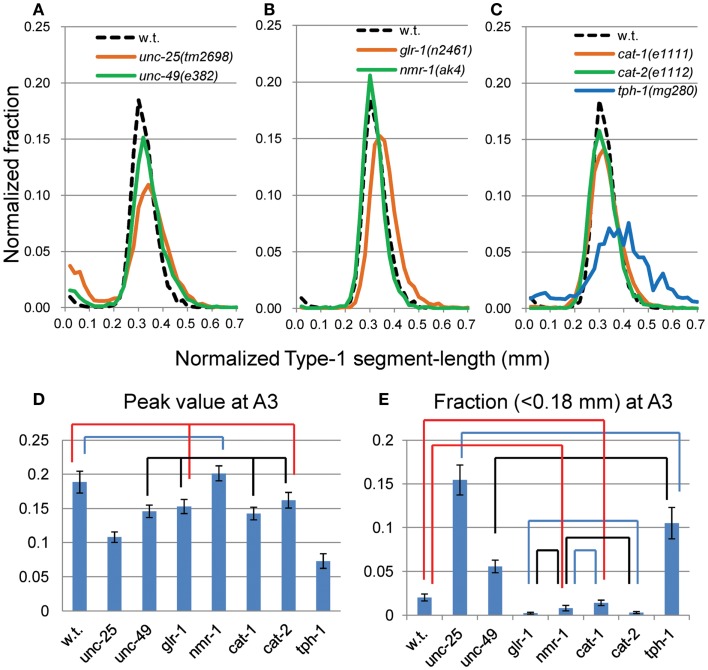
**nSL-1 histograms of forward movement of the wild type and mutant strains at the A3 stage**. Normalized histograms of Type-1 segment-length (nSL-1 histograms) at A3. **(A)** GABAergic mutants *unc-25*(*e156*) and *unc-49*(*e382*). **(B)** Glutamatergic mutants *glr-1*(*n2461*) and *nmr-1*(*ak4*). **(C)** Monoaminergic mutants *cat-1*(*e1111*), *cat-2*(*e1112*), and *tph-1*(*mg280*). **(A–C)** For comparison, the histogram of the wild type at the A3 stage is shown by a dotted line. **(D)** Peak values of nSL-1 histograms. **(E)** Fractions of the small segment-length (<0.18 mm) of nSL-1 histograms. **(D,E)** Statistically indifferent (*p* ≥ 0.01) values are connected by lines. Error bar indicates SE **(A–E)**. Numbers of worms are listed in Table 2.

The peak was highest at A3 in most strains. To examine more closely the differences in nSL-1 histograms between the strains, the peak values, and the short segment-length fraction (<0.18 mm) were analyzed at A3 (Figure 4). As shown in Figure 4D, the peak values were classified into five statistically indifferent classes; for example, the wild type, *glr-1*, and *cat-2* had similar values. On average, *nmr-1*(*ak4*) showed the highest peak, indicating the most regular head movement among the examined strains, while *tph-1*(*mg280*) showed the lowest peak with broad marginal fractions, indicating the most irregular head movement. There was a significant difference between the peak of AMPA-type receptor-channel defective *glr-1*(*n2461*) and the NMDA-type receptor-channel defective *nmr-1*(*ak4*). Figure 4E shows that there was a statistical difference in the short segment-length fractions between *cat-1*(*e1111*) and *cat-2*(*e1112*). The statistical differences between the peaks and between the short segment-length fractions of any two strains at the same age are shown in Table 3. Accordingly, the nSL-1 histogram enables us to compare the head movement periodicity between strains, and to evaluate the age-dependence of the head movement irregularity.

**Table 3:** ***t*-Test for the peak value and the small segment-length fraction of the nSL-1 histogram and the peaks in the nSL-2 histograms for the 5 and 50% points**.

Stage	A1					A3					A5					A7			
w.t. vs. *unc-25*	++	*	##	&	−	++	**	##	−	$	++	**	−	−	$$	++	**	−	$$
w.t. vs. *unc-49*	++	**	−	&&	−	++	**	−	−	$$	++	−	#	−	$	++	**	#	$$
w.t. vs. *glr-1*	−	*	#	&&	−	−	**	−	&&	−	++	**	−	&&	−	++	−	−	−
w.t. vs. *nmr-1*	−	**	−	−	−	−	*	−	&&	$$	−	**	−	&&	$$	−	**	#	$$
w.t. vs. *tph-1*	++	**	##	−	−	++	**	##	−	$$	++	**	−	−	$$	++	**	−	$
w.t. vs. *cat-1*	−	*	−	−	−	+	−	##	&&	$	++	−	#	&&	−	+	*	−	−
w.t. vs. *cat-2*	−	−	−	&&	−	−	**	−	&&	$	−	**	−	&&	$$	++	**	##	$$
*unc-25* vs. *unc-49*	−	**	##	&&	$$	++	**	##	−	−	−	**	##	−	−	−	**	##	−
*unc-25* vs. *glr-1*	−	**	##	&&	−	++	**	##	&&	$	+	**	−	&&	$	−	**	##	$
*unc-25* vs. *nmr-1*	++	**	##	&&	−	++	**	#	&&	$$	++	**	−	&&	$$	−	**	##	$$
*unc-25* vs. *tph-1*	++	**	−	−	−	++	−	−	&&	−	++	**	−	&	$	−	**	−	−
*unc-25* vs. *cat-1*	−	−	#	&	$$	+	**	−	&&	$$	+	**	−	&	$	++	**	−	−
*unc-25* vs. *cat-2*	−	**	##	&&	−	++	**	#	&&	$$	++	**	−	&&	$$	++	**	##	$$
*unc-49* vs. *glr-1*	−	**	##	&&	$	−	**	−	&&	$$	−	**	##	&	−	−	**	##	$
*unc-49* vs. *nmr-1*	++	**	−	&&	−	++	**	−	&&	$$	+	**	##	&&	$$	++	**	##	$$
*unc-49* vs. *tph-1*	++	−	##	−	$	++	−	##	−	−	+	−	−	&&	−	−	−	#	−
*unc-49* vs. *cat-1*	−	−	−	&&	−	−	**	##	&&	$$	−	−	##	−	$	++	**	−	$
*unc-49* vs. *cat-2*	−	**	−	&&	$$	−	**	−	&&	$$	−	**	#	&&	$$	++	**	##	$$
*glr-1* vs. *nmr-1*	++	−	−	&&	−	++	−	−	−	$$	++	−	−	−	$$	++	−	−	$$
*glr-1* vs. *tph-1*	++	**	##	&&	−	++	**	##	&&	&&	++	**	#	&&	$$	−	−	#	−
*glr-1* vs. *cat-1*	−	**	##	&&	$	−	**	##	−	−	−	*	−	−	−	−	−	#	−
*glr-1* vs. *cat-2*	−	−	−	−	−	−	−	−	−	−	−	−	−	&&	$$	−	−	#	$
*nmr-1* vs. *tph-1*	++	**	##	−	−	++	**	##	&&	$$	++	**	−	&&	$$	++	**	##	$$
*nmr-1* vs. *cat-1*	++	**	#	−	−	++	−	#	−	−	++	−	−	−	$$	+	**	#	$$
*nmr-1* vs. *cat-2*	++	−	−	&&	−	+	−	−	−	−	−	−	−	&&	−	+	−	−	−
*tph-1* vs. *cat-1*	++	**	##	−	$	++	**	#	&&	$$	++	−	#	&&	$$	++	−	−	−
*tph-1* vs. *cat-2*	++	**	##	&&	−	++	**	##	&&	$$	++	**	−	&&	$$	++	**	##	$$
*cat-1* vs. *cat-2*	−	**	−	&&	$$	−	**	#	&&	−	+	*	−	&&	$$	−	**	##	$$

Each entry is composed of results of five (four in A7) *t*-tests, between the peak values (+ and ++) or the small segment-length (<0.18 mm) fractions (* and **) in the nSL-1 histograms, between the peak values at 0.06–0.1 mm (# and ##) or the segment-length values at the peak (& and &&) in nSL-2 histograms (5%), and between the peaks values at 0.3–0.4 mm ($ and $$) in the nSL-2 histograms (50%). +, *, #, &, $: *p* < 0.05; ++, **, ##, &&, $$: *p* < 0.01; −: *p* ≥ 0.05. Light gray row indicates the maximal difference for the five (four in A7) pairs was *p* < 0.05. Dark gray row indicates *p* ≥ 0.05 for all five pairs.

### Small head movements and deviation from sinusoidal body movement

**Figure 5:**
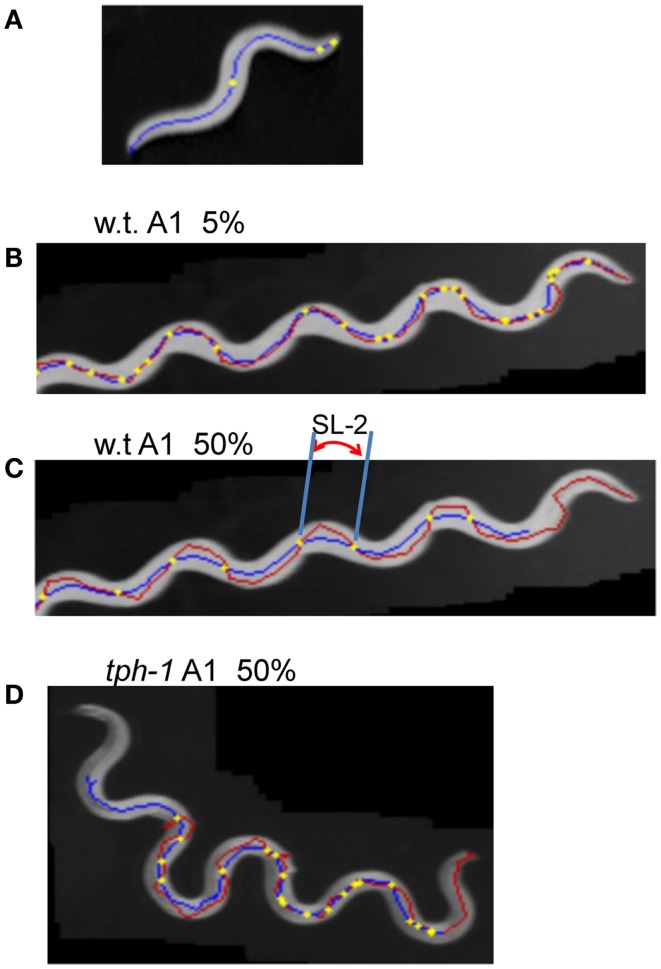
**Type-2 segment-length**. **(A)** An image of a wild type worm at the A1 stage. The blue line is the centerline of the image. The three yellow points indicate the head end, the 5 and 50% points on the centerline from the head end. **(B–D)** Superimposed images of a worm (gray), the head trace (red), and the trace of the 5 or 50% point (blue). The yellow points indicate the intersections of the two traces. The length of the head trace between adjacent intersections is the Type-2 segment-length; an example is indicated by SL-2 in **(C)**. **(B)** Intersections and the trace of the 5% point of the wild type worm at the A1 stage. **(C)** Intersections and the trace of the 50% point of the wild type worm at the A1 stage. **(D)** Trace of the 50% point of the *tph-1* mutant worm at A1.

**Figure 6:**
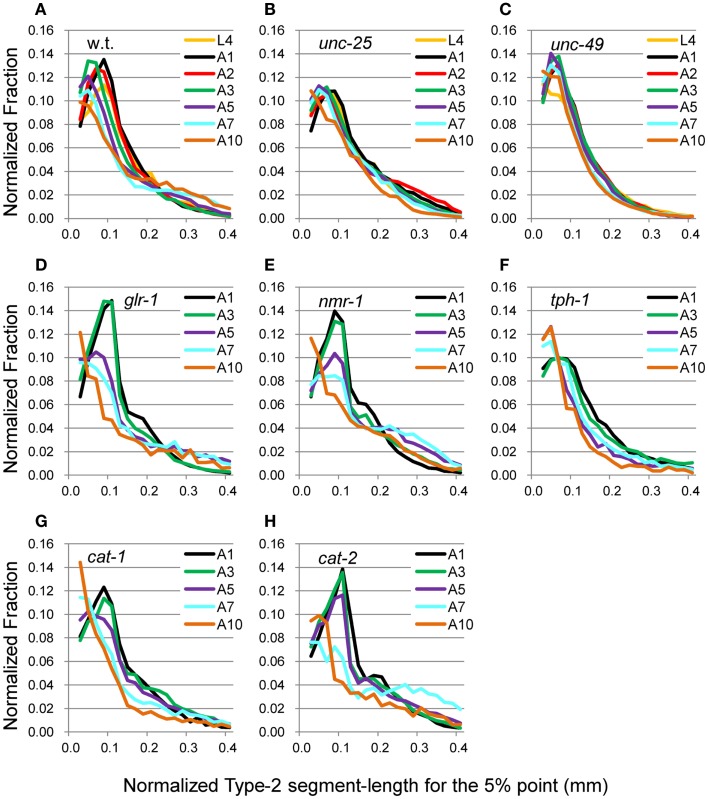
**nSL-2 histograms (5%) of the wild type and mutant strains at various ages**. Normalized histograms of Type-2 segment-length for the 5% point [nSL-2 histograms (5%)]. **(A)** Wild type (N2). **(B)** *unc-25*(*e156*). **(C)** *unc-49*(*e382*). **(D)** *glr-1*(*n2461*). **(E)** *nmr-1*(*ak4*). **(F)** *tph-1*(*mg280*). **(G)** *cat-1*(*e1111*). **(H)** *cat-2*(*e1112*). **(A–C)** Ages L4–A10. **(D–H)** Ages A1–A10. Numbers of worms are listed in Table 2.

**Figure 7:**
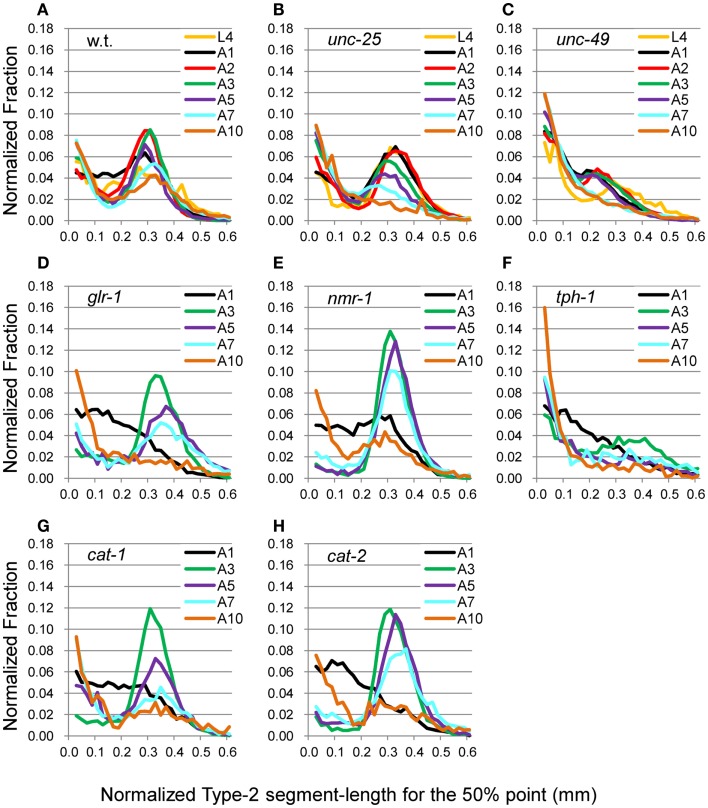
**nSL-2 histograms (50%) of the wild type and mutant strains at various ages**. Normalized histograms of Type-2 segment-length for the 50% point [nSL-2 histograms (50%)]. **(A)** Wild type (N2). **(B)** *unc-25*(*e156*). **(C)** *unc-49*(*e382*). **(D)** *glr-1*(*n2461*). **(E)** *nmr-1*(*ak4*). **(F)** *tph-1*(*mg280*). **(G)** *cat-1*(*e1111*). **(H)** *cat-2*(*e1112*). **(A–C)** Ages L4–A10. **(D–H)** Ages A1–A10. Numbers of worms are listed in Table 2.

The head end and the 5 and 50% points from the head end in the worm’s centerline are shown in Figure 5A. Figures 5B,C show the superimposition of wild type worm images during forward movement, with the trace of the head end and that of the 5% point (Figure 5B) or the 50% point (Figure 5C) in the worm’s centerline. The normalized histogram of the Type-2 segment-length [nSL-2 histogram (5 or 50%)] was defined as described in Section “Materials and Methods.” The nSL-2 histograms (5%) of the wild type and mutants at various ages are shown in Figure 6. The nSL-2 histograms (5 and 50%) of individual worms are shown in Figures S2 and S3 in Supplementary Material. When the nSL-2 histogram (5%) had a peak, it was located between 0.06 and 0.1 mm. In each strain between A1 and A5 there was an apparent peak. In several strains (*unc-25*, *unc-49*, and *tph-1*) the peak value barely changed between A1 and A5. In the other strains the peak slightly decreased with age. The peak disappeared at A10 in all strains. The nSL-2 histograms (50%) of the L4 and A1 stages demonstrated no apparent peaks or only small peaks, except for *unc-25*(*e156*) in which there was a peak (Figure 7). However, similarly to the nSL-1 histogram, a peak appeared around 0.3–0.4 mm between ages A3 and A7 in all strains except for *unc-49*(*e382*) and *tph-1*(*mg280*). These peaks indicated that the head swings were coordinated with body bending, but with different amplitudes from the body bending, as seen in Figure 5C. Therefore, the head swing or body bending changed between A1 and A3 in these strains. In contrast, *unc-49*(*e07*) and *tph-1*(*mg280*) did not show an apparent peak at 0.3–0.4 mm, although there was a shoulder around 0.2–0.3 mm at the A1–A5 stages in *unc-49*. In these two mutants, the trace of the head end and the trace of the worm’s middle point intersected randomly (Figure 5D, representative image). There were some differences between the histograms for the 5 and 50% points. Both the change in the peak by age and the difference in the peak between strains in the nSL-2 histogram for the 5% were smaller than in that for the 50% point. Also, at the A1 stage, there was a peak in the histogram for the 5% point in all strains, while in the histogram for the 50% point some strains showed no peak (*glr-1*, *tph-1*, and *cat-2*) or only a small peak (*unc-49*, *nmr-1*, and *cat-1*). Therefore, the small head movement was more stable than the deviation of head swing from the sinusoidal body movement.

### Peak values and short segment-length component

**Figure 8:**
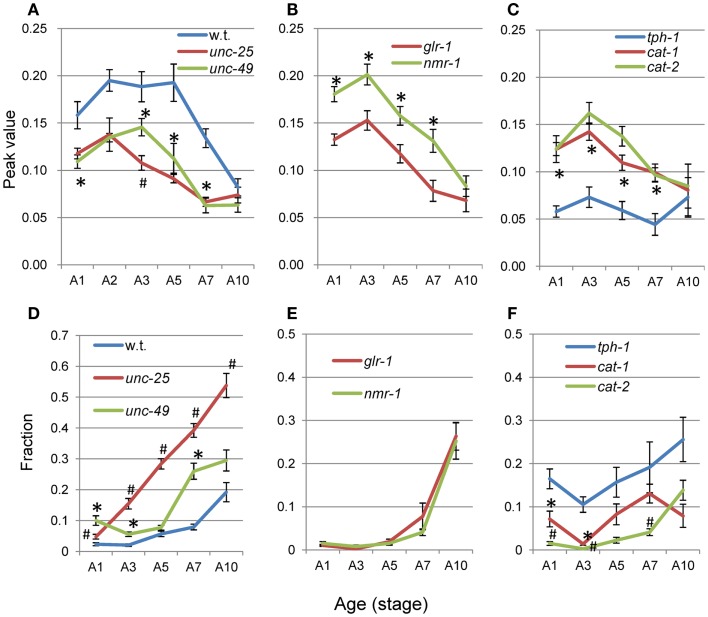
**Age changes in peak values and small segment-length fractions of nSL-1 histograms**. **(A–C)** Peak values of the nSL-1 histograms. **(D–F)** Small segment-length fractions (<0.18 mm) in the nSL-1 histograms. **(A,D)** Wild type (N2), *unc-25*(*e156*), and *unc-49*(*e382*). **(B**,**E)** *glr-1*(*n2461*) and *nmr-1*(*ak4*). **(C,F)** *tph-1*(*mg280*), *cat-1*(*e1111*), and *cat-2*(*e1112*). *Indicates a statistically significant difference (*p* < 0.01) between the wild type and *unc-49* in **(A,D)**; between *glr-1* and *nmr-1* in **(B,E)**; and between *tph-1* and *cat-1* in **(C,F)**. ^#^Indicates a statistically significant difference (*p* < 0.01) between *unc-25* and *unc-49* in **(A,D)**; and between *cat-1* and *cat-2* in **(C,F)**. Numbers of worms are listed in Table 2. Error bar indicates SE.

The average nSL-1 histogram at each stage of each strain was obtained, and the peak values are shown in Figures 8A–C. The peak occurred between A2 and A5 in all strains except for *tph-1*. After these stages, the peak values decreased with age. Figures 8D–F show the short segment-length fraction (<0.18 mm) of the nSL-1 histograms. This fraction generally increased with age, but the most rapid increase was observed in *unc-25*(*e156*). The large effects seen in the GABAergic mutants are probably because GABA neurons in the head send synaptic output directly to head muscles.

**Figure 9:**
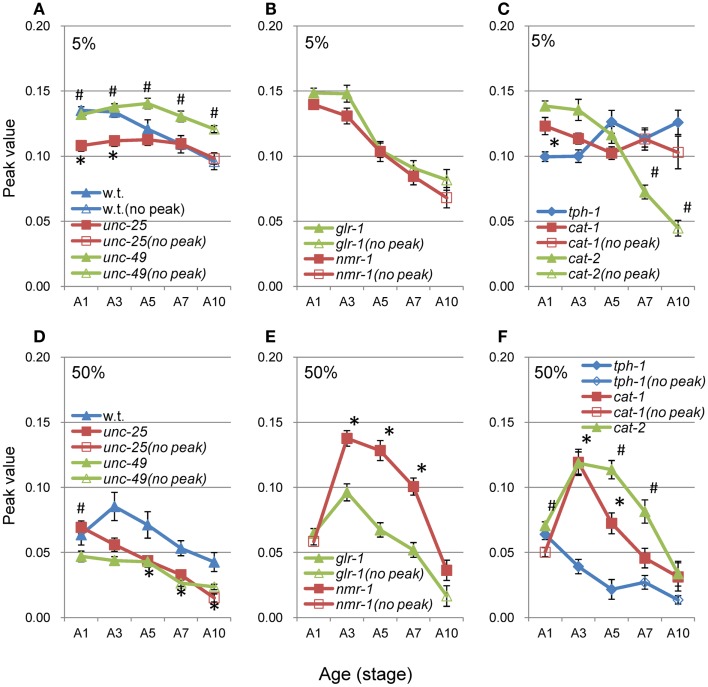
**Age changes in peak values of nSL-2 histograms**. **(A–C)** Peak values (closed symbols) in the nSL-2 histograms (5%) around 0.06–0.10 mm. **(D–F)** Peak values (closed symbols) in the nSL-2 histograms (50%) around 0.3–0.4 mm. When there was no peak around 0.06–0.10 mm in the nSL-2 (5%) histogram or 0.3–0.4 mm in the nSL-2 (50%) histogram, the histogram value at the same segment-length as that of the peak in the closest age histogram having a peak was plotted (open symbols). * and ^#^ have the same meaning as in Figure 8. Numbers of worms are listed in Table 2. Error bar indicates SE.

When there was no peak around 0.06–0.1 mm in the nSL-2 (5%) histogram or around 0.3–0.4 mm in the nSL-2 (50%) histogram, the histogram value at the same segment-length (mm) as that of the peak in the closest age histogram with a peak was plotted as shown in Figure 9 (open symbols). If there was a peak around 0.1 mm in the nSL-2 (5%) histogram or around 0.3–0.4 mm in the nSL-2 (50%) histogram (Figure 9, filled symbols), the peak value was plotted. Differences were observed between GABAergic mutants (Figures 9A,D), glutamatergic mutants (Figures 9B,E), and monoaminergic mutants (Figures 9C,F). Among the strains, the values of *nmr-1*(*ak4*) in the nSL-2 (50%) histogram were highest between A3 and A7, indicating that this mutant worm showed the most periodic crossings between the trace of the head and the trace of the worm’s midpoint. Although there was no difference between the peak height of the nSL-1 histograms of monoaminergic mutants *cat-1*(*e1111*) and *cat-2*(*e1112*), significant differences were observed between the small segment-length fractions of the nSL-1 histograms, and between the peaks around 0.3–0.4 mm of the nSL-2 histograms. The statistical differences of the normalized Type-2 segment-length value at the peak of nSL-2 (5%) between the different strains at A1, A3, or A5 were also examined.

Table 3 shows a quintuplet of statistical differences between the strains: the differences between the peak value of the nSL-1 histogram, the short segment-length (<0.18 mm) fraction of the nSL-1 histogram, the peak values of the nSL-2 (5%) and nSL-2 (50%) histograms, and the normalized Type-2 segment-length values at the peak of nSL-2 (5%). At the A1 and A3 stages, 27 out of 28 entries (96%) included at least one value of *p* < 0.01 or *p* < 0.05 within the quintuplet, and 26 out of 28 entries (93%) included at least one value of *p* < 0.01 or at least two values of *p* < 0.05. At the A5 stage, these values were 100 and 96%, respectively. Therefore, when the quintuplet is used, the difference between the majority of the strains could be identified. When both A5 and either of A1 or A3 were examined, the statistical difference between every pair of strains could be detected.

## Discussion

Head movement of *C. elegans* is more complex than the sinusoidal body movement during locomotion. We expected that some abnormalities in the central nervous system would emerge as abnormal head movements. The methods described here enable us to evaluate differences in head movements between different groups of worms, although the methods are only applicable to strains or worms in conditions that show locomotion. The methods are applicable to backward movements, although we applied them here only to forward locomotion. The methods can be used to image data that are captured at any sampling frequency. Images of the worm were captured here at 3 images/s, which was not sufficient for analysis of very fast movements of foraging. However, the results show that the methods can extract features such as the small irregular head movements of GABA-deficient mutant *unc-25*, the age-dependence of head movements, and differences between strains.

The peak height of the nSL-1 histogram is a measure of the periodicity of head swing. A significant difference was observed between the peak heights of the *glr-1*(*n2461*) and *nmr-1*(*ak4*) mutant worms. In all strains, irregular head swing, which was seen in the short segment-length fraction (<0.18 mm) of the nSL-1 histogram, increased with age. *unc-25*(*e156*) exhibited the most prominent feature. Lack of GABA in *unc-25* (McIntire et al., 1993a,b) produced larger irregularity of head swing than the defect in ionotropic GABA receptors in *unc-49*(*e382*) (Bamber et al., 1999); GABA neural transmission *via* receptors other than UNC-49, such as GABA_B_ receptors (Dittman and Kaplan, 2008) may be the reason for the difference between the two mutant strains. The short segment-length fraction of *unc-25*(*e156*) rapidly increased as age progressed. In contrast, in *tph-1*(*mg280*), the main peak height of nSL-1 was almost constant during A1–A10, indicating that the irregularity showed the least change with age, which was different from the other strains. Of all the mutants we examined here, *unc-25*(*e156*) and *tph-1*(*mg280*) showed the most evident phenotypes, probably because they are deficient in neurotransmitters, which have vast effects on the nervous system functions compared with defects in receptors or vesicular transporters.

Comparing the age-dependency of the nSL-2 histograms for the 5 and 50% points, the small movements at the head end were more stable than the deviation of the head end movement from the body center movement. Perhaps the small head movements are more robust in the neuromuscular system for exploratory behavior to external cues. White et al. (1986) demonstrated that the head muscles are innervated in the nerve ring in the head, while the neck muscles are innervated in the nerve ring and the ventral cord. Therefore, the muscles at the head end are controlled by neurons different from the neurons that control the neck muscles, which may be the cause of the difference between the histograms for the 5 and 50% points. In the wild type, *glr-1*, *nmr-1*, *cat-1*, and *cat-2*, the nSL-2 histograms (50%) showed that there was a turning point between A1 and the later stages (Figure 7); only before this turning stage, the histograms showed no peak or only a small peak. In *unc-49* and *tph-1*, only a very small peak or no peak was detected at each stage. The curves without or with a very small peak indicate that the head trace crossed the trace of the 50% point almost randomly, as seen in *tph-1*(*mg280*) (Figure 5D).

The reasons in terms of the nervous system that would explain the differences in the head movement remain to be elucidated. However, the methods proposed here add the head movement to the locomotion phenotypes, which had been restricted to speed, distance, duration of movement (or frequency of sharp turns), and body bending. The five values listed in each entry in Table 3 discriminate the difference between most pairs of strains, which would also be useful for examining effects of exogenously applied neuroactive chemicals.

In conclusion, it is rational that some abnormalities in the central nervous system could emerge as abnormal head movements. The methods proposed here provide simple measures for comparing head movements during locomotion between different groups of worms, and they are especially useful for evaluating age-dependent changes. Furthermore, the methods would probably be useful for assessing effects of external environment or application of chemicals.

## Conflict of Interest Statement

The authors declare that the research was conducted in the absence of any commercial or financial relationships that could be construed as a potential conflict of interest.

## Supplementary Material

The Supplementary Material for this article can be found online at http://www.frontiersin.org/Behavioral_Neuroscience/10.3389/fnbeh.2013.00020/abstract

Supplementary Figure S1 **nSL-1 histograms for each worms**.

Supplementary Figure S2 **nSL-2 histograms (5%) for each worms**.

Supplementary Figure S3 **nSL-2 histograms (50%) for each worms**.

Supplementary Software S1 **Outline of program**.

Supplementary Software S2 **Test input data for Softwares S3 and S4**.

Supplementary Software S3 **Computer programs for Type-1 segment-lengths**.

Supplementary Software S4 **Computer programs for Type-2 segment-lengths**.
