# Supplementary material for: Evaluation of Head Movement Periodicity and Irregularity during Locomotion of Caenorhabditis elegans
Source: Front Behav Neurosci. 2013 Mar 21;7:20. doi: 10.3389/fnbeh.2013.00020 (PMC3604732; doi:10.3389/fnbeh.2013.00020)
Supplement: Supplementary Figure S1 — nSL-1 histograms for each worms. [file 42064_Shingai_Presentation1.PPTX]

## Slide 1
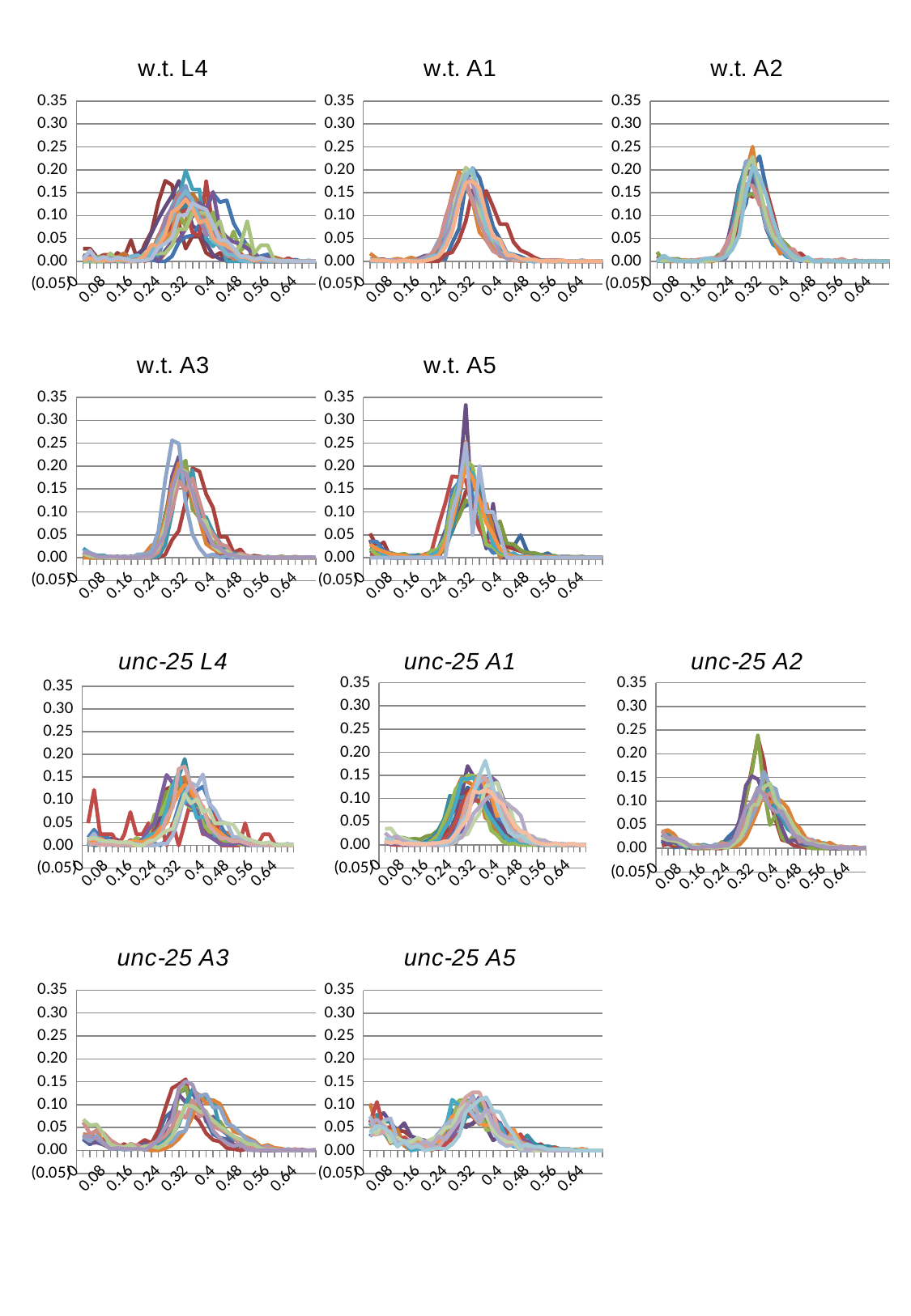

### Chart: w.t. L4
| Category | N2 L4 No.1 | N2 L4 No.2 | N2 L4 No.3 | N2 L4 No.4 | N2 L4 No.5 | N2 L4 No.6 | N2 L4 No.7 | N2 L4 No.8 | N2 L4 No.9 | N2 L4 No.10 | N2 L4 No.11 | N2 L4 No.12 | N2 L4 No.13 | N2 L4 No.14 | N2 L4 No.15 | N2 L4 No.16 | N2 L4 No.17 | N2 L4 No.18 | N2 L4 No.19 |
|---|---|---|---|---|---|---|---|---|---|---|---|---|---|---|---|---|---|---|---|
| 0 | None | None | None | None | None | None | None | None | None | None | None | None | None | None | None | None | None | None | None |
| 2.0000000000000007E-2 | 0.001769911 | 0.02777778000000001 | 0.0 | 0.0 | 0.0017985610000000004 | 0.008968610000000005 | 0.0 | 0.013986010000000004 | 0.0 | 0.0 | 0.003984064000000002 | 0.001501502 | 0.014218009999999998 | 0.004373178000000002 | 0.0 | 0.004545454 | 0.00877193 | 0.003300330000000001 | 0.00483871 |
| 4.0000000000000015E-2 | 0.005309734000000002 | 0.02777778000000001 | 0.0 | 0.002812939 | 0.0017985610000000004 | 0.008968610000000005 | 0.0 | 0.0069930070000000025 | 0.004651163000000002 | 0.0 | 0.003984064000000002 | 0.001501502 | 0.004739337000000002 | 0.001457726 | 0.0 | 0.01590909 | 0.002192982 | 0.006600660000000002 | 0.024193549999999998 |
| 6.0000000000000019E-2 | 0.008849557000000006 | 0.009259259000000004 | 0.0 | 0.00140647 | 0.0 | 0.004484305000000002 | 0.0 | 0.0069930070000000025 | 0.009302326000000007 | 0.0 | 0.0 | 0.0 | 0.0 | 0.001457726 | 0.0 | 0.002272727000000001 | 0.0065789470000000025 | 0.0016501650000000005 | 0.003225806 |
| 8.0000000000000029E-2 | 0.003539823 | 0.009259259000000004 | 0.0 | 0.00140647 | 0.0017985610000000004 | 0.01345291 | 0.0 | 0.013986010000000004 | 0.009302326000000007 | 0.0 | 0.0019920320000000012 | 0.0 | 0.004739337000000002 | 0.0 | 0.0 | 0.004545454 | 0.004385965 | 0.003300330000000001 | 0.009677419000000003 |
| 9.9999990000000039E-2 | 0.003539823 | 0.0 | 0.0 | 0.002812939 | 0.0 | 0.01345291 | 0.0 | 0.013986010000000004 | 0.0 | 0.0 | 0.0019920320000000012 | 0.0 | 0.004739337000000002 | 0.001457726 | 0.01754386 | 0.006818182 | 0.004385965 | 0.0016501650000000005 | 0.003225806 |
| 0.12000000000000002 | 0.003539823 | 0.018518520000000007 | 0.0 | 0.00140647 | 0.0017985610000000004 | 0.008968610000000005 | 0.003802281 | 0.0 | 0.009302326000000007 | 0.0 | 0.003984064000000002 | 0.001501502 | 0.0 | 0.002915452 | 0.0 | 0.004545454 | 0.002192982 | 0.003300330000000001 | 0.008064516000000004 |
| 0.14000000000000001 | 0.008849557000000006 | 0.009259259000000004 | 0.0 | 0.004219409000000002 | 0.0 | 0.01793722 | 0.003802281 | 0.0069930070000000025 | 0.004651163000000002 | 0.0 | 0.0 | 0.0 | 0.0 | 0.005830904000000001 | 0.0 | 0.0 | 0.002192982 | 0.003300330000000001 | 0.006451613000000004 |
| 0.16 | 0.003539823 | 0.04629629000000001 | 0.002421308 | 0.007032349000000004 | 0.005395683000000002 | 0.004484305000000002 | 0.0 | 0.0069930070000000025 | 0.0 | 0.0 | 0.005976096000000004 | 0.0 | 0.0 | 0.0 | 0.0 | 0.004545454 | 0.01096491 | 0.0 | 0.0016129030000000004 |
| 0.18000000000000005 | 0.0 | 0.0 | 0.0 | 0.01687764 | 0.0035971220000000016 | 0.0 | 0.0 | 0.0069930070000000025 | 0.004651163000000002 | 0.008264462000000005 | 0.003984064000000002 | 0.0 | 0.004739337000000002 | 0.005830904000000001 | 0.0 | 0.0 | 0.01315789 | 0.003300330000000001 | 0.0016129030000000004 |
| 0.2 | 0.008849557000000006 | 0.03703704000000002 | 0.014527850000000004 | 0.028129399999999992 | 0.0035971220000000016 | 0.004484305000000002 | 0.003802281 | 0.0 | 0.009302326000000007 | 0.008264462000000005 | 0.007968128000000003 | 0.004504504999999998 | 0.009478673 | 0.008746356 | 0.01754386 | 0.004545454 | 0.0065789470000000025 | 0.011551150000000005 | 0.0016129030000000004 |
| 0.22 | 0.023008849999999997 | 0.06481481000000001 | 0.019370460000000006 | 0.06188467000000002 | 0.01438849 | 0.01345291 | 0.003802281 | 0.0069930070000000025 | 0.02325581 | 0.02479339 | 0.03585657 | 0.012012009999999998 | 0.014218009999999998 | 0.01603498 | 0.01754386 | 0.018181820000000008 | 0.019736840000000005 | 0.03630363000000001 | 0.006451613000000004 |
| 0.24000000000000005 | 0.040707960000000015 | 0.12962959999999996 | 0.055690070000000015 | 0.09282701 | 0.01798561 | 0.0 | 0.0 | 0.013986010000000004 | 0.02790698 | 0.008264462000000005 | 0.03585657 | 0.022522519999999997 | 0.0 | 0.05247813 | 0.01754386 | 0.022727270000000008 | 0.04605263000000001 | 0.0330033 | 0.03064516000000001 |
| 0.26 | 0.07787611 | 0.17592590000000005 | 0.08232445000000005 | 0.1195499 | 0.05395683 | 0.01793722 | 0.0 | 0.06293707 | 0.04651163 | 0.0661157 | 0.05179283000000002 | 0.07357357000000002 | 0.023696679999999998 | 0.09037901000000001 | 0.01754386 | 0.09318182000000003 | 0.06798246000000002 | 0.059405940000000004 | 0.03870968 |
| 0.28000000000000008 | 0.09380531000000004 | 0.1666667 | 0.10895879999999998 | 0.14345990000000006 | 0.08093525 | 0.040358740000000004 | 0.011406840000000001 | 0.07692308000000002 | 0.05116279000000002 | 0.09917355000000004 | 0.06374502 | 0.1201201 | 0.04265403 | 0.09912536000000008 | 0.03508772000000001 | 0.1181818 | 0.09429824 | 0.10726070000000006 | 0.05161290000000002 |
| 0.3000000000000001 | 0.1115044 | 0.08333334000000003 | 0.15254240000000008 | 0.1758087 | 0.102518 | 0.08520179000000001 | 0.04182509 | 0.10489510000000005 | 0.1069767 | 0.14049590000000006 | 0.12350600000000003 | 0.1486486 | 0.04739337000000001 | 0.14723030000000006 | 0.07017544 | 0.12045450000000002 | 0.1337719 | 0.1171617 | 0.10161290000000002 |
| 0.32000000000000012 | 0.12566369999999993 | 0.02777778000000001 | 0.1501211 | 0.13220820000000005 | 0.12050360000000003 | 0.08520179000000001 | 0.05323194 | 0.11188809999999996 | 0.07441860000000004 | 0.19834710000000005 | 0.15139440000000007 | 0.1651652 | 0.07109005 | 0.1457726 | 0.07017544 | 0.1431818 | 0.15350880000000006 | 0.1353135 | 0.1 |
| 0.34 | 0.1362832 | 0.05555555999999998 | 0.09927361000000001 | 0.07735584 | 0.13848920000000006 | 0.14798210000000006 | 0.05703422000000001 | 0.06293707 | 0.1255814 | 0.1570248 | 0.1294821 | 0.1186186 | 0.09952607000000009 | 0.1180758 | 0.10526320000000006 | 0.1340909 | 0.1337719 | 0.11551160000000002 | 0.12741940000000007 |
| 0.3600000000000001 | 0.11858410000000003 | 0.05555555999999998 | 0.10411620000000005 | 0.06751055 | 0.13129500000000005 | 0.1255605 | 0.07984791 | 0.05594405999999998 | 0.09302326000000004 | 0.1570248 | 0.10358570000000003 | 0.11411410000000002 | 0.12796210000000005 | 0.1107872 | 0.10526320000000006 | 0.09545454000000005 | 0.09210526000000001 | 0.08415841000000003 | 0.1193548 |
| 0.38000000000000012 | 0.08672567000000005 | 0.018518520000000007 | 0.07506053000000003 | 0.039381150000000004 | 0.102518 | 0.1345291 | 0.1140684 | 0.1748252 | 0.07906977 | 0.04958678000000001 | 0.10159360000000005 | 0.07207207000000003 | 0.11374410000000003 | 0.061224489999999986 | 0.10526320000000006 | 0.05681818 | 0.08333334000000003 | 0.09075908 | 0.11290320000000002 |
| 0.4 | 0.061946899999999985 | 0.009259259000000004 | 0.046004839999999984 | 0.014064699999999998 | 0.07913669000000004 | 0.08520179000000001 | 0.14828900000000006 | 0.06993007 | 0.1069767 | 0.03305785 | 0.07171315 | 0.04804804999999999 | 0.1516588 | 0.05685131 | 0.07017544 | 0.06136364000000002 | 0.04385965 | 0.05115511999999997 | 0.08064516000000004 |
| 0.4200000000000001 | 0.040707960000000015 | 0.018518520000000007 | 0.03147700000000001 | 0.005625879 | 0.052158270000000014 | 0.05829597 | 0.12927759999999994 | 0.07692308000000002 | 0.03720930000000002 | 0.04958678000000001 | 0.061752990000000035 | 0.054054060000000015 | 0.07109005 | 0.0335277 | 0.0877193 | 0.029545449999999997 | 0.03508772000000001 | 0.039603960000000014 | 0.04677419 |
| 0.44 | 0.0159292 | 0.0 | 0.019370460000000006 | 0.002812939 | 0.03956835 | 0.05381166 | 0.1330798 | 0.03496503 | 0.02790698 | 0.0 | 0.01792829 | 0.01501502 | 0.05213270000000002 | 0.01311953 | 0.03508772000000001 | 0.022727270000000008 | 0.015350880000000004 | 0.03465347 | 0.045161290000000014 |
| 0.46 | 0.008849557000000006 | 0.0 | 0.009685230000000006 | 0.00140647 | 0.01978417000000001 | 0.01793722 | 0.08365019000000007 | 0.02097902 | 0.06511628000000003 | 0.0 | 0.005976096000000004 | 0.01501502 | 0.04265403 | 0.008746356 | 0.01754386 | 0.018181820000000008 | 0.01315789 | 0.02475247999999999 | 0.029032260000000008 |
| 0.48000000000000009 | 0.007079646000000005 | 0.009259259000000004 | 0.009685230000000006 | 0.0 | 0.008992806000000004 | 0.01793722 | 0.05703422000000001 | 0.013986010000000004 | 0.02325581 | 0.0 | 0.005976096000000004 | 0.007507508000000002 | 0.03791469 | 0.005830904000000001 | 0.03508772000000001 | 0.002272727000000001 | 0.002192982 | 0.009900990000000005 | 0.011290320000000001 |
| 0.5 | 0.003539823 | 0.0 | 0.01210654 | 0.0 | 0.007194245000000002 | 0.004484305000000002 | 0.034220529999999985 | 0.0069930070000000025 | 0.03720930000000002 | 0.0 | 0.003984064000000002 | 0.001501502 | 0.02843602 | 0.005830904000000001 | 0.0877193 | 0.009090909000000003 | 0.0 | 0.008250825000000002 | 0.011290320000000001 |
| 0.52 | 0.0 | 0.0 | 0.0 | 0.0 | 0.005395683000000002 | 0.01345291 | 0.003802281 | 0.02097902 | 0.01395349 | 0.0 | 0.0019920320000000012 | 0.001501502 | 0.01895735 | 0.002915452 | 0.01754386 | 0.004545454 | 0.002192982 | 0.0016501650000000005 | 0.00483871 |
| 0.54 | 0.0 | 0.0 | 0.0 | 0.0 | 0.0 | 0.0 | 0.011406840000000001 | 0.0 | 0.004651163000000002 | 0.0 | 0.0 | 0.001501502 | 0.004739337000000002 | 0.0 | 0.03508772000000001 | 0.0 | 0.002192982 | 0.004950495000000002 | 0.008064516000000004 |
| 0.56000000000000005 | 0.0 | 0.0 | 0.0 | 0.0 | 0.0 | 0.0 | 0.015209130000000001 | 0.0 | 0.0 | 0.0 | 0.0 | 0.0 | 0.009478673 | 0.0 | 0.03508772000000001 | 0.0 | 0.0 | 0.003300330000000001 | 0.003225806 |
| 0.58000000000000007 | 0.0 | 0.0 | 0.004842615 | 0.0 | 0.0017985610000000004 | 0.008968610000000005 | 0.003802281 | 0.0 | 0.004651163000000002 | 0.0 | 0.0 | 0.0 | 0.0 | 0.0 | 0.0 | 0.0 | 0.0 | 0.0016501650000000005 | 0.003225806 |
| 0.6000000000000002 | 0.0 | 0.0 | 0.0 | 0.0 | 0.0017985610000000004 | 0.004484305000000002 | 0.0 | 0.0 | 0.0 | 0.0 | 0.0 | 0.0 | 0.0 | 0.0 | 0.0 | 0.002272727000000001 | 0.0 | 0.0016501650000000005 | 0.0 |
| 0.62000000000000022 | 0.0 | 0.0 | 0.0 | 0.0 | 0.0 | 0.0 | 0.0 | 0.0069930070000000025 | 0.0 | 0.0 | 0.0 | 0.0 | 0.0 | 0.0 | 0.0 | 0.0 | 0.0 | 0.0016501650000000005 | 0.0016129030000000004 |
| 0.64000000000000024 | 0.0 | 0.0 | 0.0 | 0.0 | 0.0 | 0.0 | 0.003802281 | 0.0 | 0.0 | 0.0 | 0.0 | 0.0 | 0.0 | 0.0 | 0.0 | 0.0 | 0.0 | 0.0 | 0.0 |
| 0.66000000000000025 | 0.0 | 0.0 | 0.0 | 0.0 | 0.0 | 0.0 | 0.0 | 0.0 | 0.0 | 0.0 | 0.0 | 0.0 | 0.0 | 0.0 | 0.0 | 0.0 | 0.0 | 0.0 | 0.0 |
| 0.68 | 0.0 | 0.0 | 0.0 | 0.0 | 0.0017985610000000004 | 0.0 | 0.0 | 0.0 | 0.0 | 0.0 | 0.0 | 0.0 | 0.0 | 0.0 | 0.0 | 0.0 | 0.0 | 0.0 | 0.0 |
| 0.70000000000000018 | 0.0 | 0.0 | 0.0 | 0.0 | 0.0 | 0.0 | 0.0 | 0.0 | 0.0 | 0.0 | 0.0 | 0.0 | 0.0 | 0.0 | 0.0 | 0.0 | 0.0 | 0.0 | 0.0 |
### Chart: w.t. A1
| Category | N2 A1 No.1 | N2 A1 No.2 | N2 A1 No.4 | N2 A1 No.5 | N2 A1 No.6 | N2 A1 No.7 | N2 A1 No.8 | N2 A1 No.9 | N2 A1 No.10 | N2 A1 No.11 | N2 A1 No.12 |
|---|---|---|---|---|---|---|---|---|---|---|---|
| 0 | None | None | None | None | None | None | None | None | None | None | None |
| 2.0000000000000004E-2 | 0.0 | 0.004395605 | 0.004914004999999999 | 0.001067236 | 0.017341040000000002 | 0.0037383180000000005 | 0.001477105 | 0.002806361 | 0.0030456850000000002 | 0.001731602 | 0.003303055 |
| 4.0000000000000008E-2 | 0.0 | 0.004395605 | 0.0024570020000000002 | 0.002134472 | 0.005780347000000001 | 0.0037383180000000005 | 0.001477105 | 0.002806361 | 0.001015228 | 0.0008658010000000001 | 0.0016515280000000002 |
| 6.0000000000000005E-2 | 0.0 | 0.004395605 | 0.0024570020000000002 | 0.0 | 0.0 | 0.0018691590000000002 | 0.001477105 | 0.0009354540000000006 | 0.001015228 | 0.001731602 | 0.0024772920000000003 |
| 8.0000000000000016E-2 | 0.00210084 | 0.0 | 0.0024570020000000002 | 0.002134472 | 0.002890173 | 0.0028037380000000005 | 0.001477105 | 0.0009354540000000006 | 0.001015228 | 0.0 | 0.0 |
| 0.1 | 0.00210084 | 0.002197802 | 0.003685504000000001 | 0.0 | 0.005780347000000001 | 0.0018691590000000002 | 0.00295421 | 0.0 | 0.001015228 | 0.0 | 0.0 |
| 0.12000000000000001 | 0.0 | 0.002197802 | 0.0024570020000000002 | 0.0 | 0.002890173 | 0.0009345800000000004 | 0.0 | 0.0009354540000000006 | 0.0 | 0.0 | 0.0024772920000000003 |
| 0.14000000000000001 | 0.00210084 | 0.0 | 0.0024570020000000002 | 0.001067236 | 0.008670520000000003 | 0.0018691590000000002 | 0.001477105 | 0.0 | 0.001015228 | 0.0008658010000000001 | 0.0 |
| 0.16 | 0.0 | 0.0 | 0.006142505999999999 | 0.002134472 | 0.002890173 | 0.0018691590000000002 | 0.001477105 | 0.0009354540000000006 | 0.002030457 | 0.0 | 0.0 |
| 0.18000000000000002 | 0.00210084 | 0.0 | 0.012285009999999999 | 0.002134472 | 0.008670520000000003 | 0.008411215000000001 | 0.005908419000000001 | 0.0037418150000000003 | 0.007106599000000001 | 0.001731602 | 0.0008257640000000005 |
| 0.2 | 0.0042016810000000005 | 0.0 | 0.013513510000000001 | 0.0149413 | 0.008670520000000003 | 0.015887850000000002 | 0.01772526 | 0.0074836300000000015 | 0.01218274 | 0.010389609999999999 | 0.004954583 |
| 0.22 | 0.008403362000000001 | 0.0 | 0.02948403 | 0.02027748 | 0.04046243000000002 | 0.04485981 | 0.03397341 | 0.01496726 | 0.014213199999999999 | 0.01298701 | 0.010734929999999998 |
| 0.24000000000000002 | 0.0042016810000000005 | 0.015384620000000002 | 0.06756756 | 0.059765210000000006 | 0.08092485000000002 | 0.06542056 | 0.09453471000000004 | 0.04022450999999999 | 0.04873097 | 0.03809524 | 0.03055326 |
| 0.26 | 0.03991597 | 0.019780220000000005 | 0.09459460000000003 | 0.10779080000000003 | 0.14739880000000002 | 0.1373832 | 0.140325 | 0.09728719 | 0.08730964 | 0.06839827000000001 | 0.06440958 |
| 0.28000000000000008 | 0.07142857 | 0.046153849999999996 | 0.1511057 | 0.14194240000000005 | 0.1965318 | 0.18224300000000002 | 0.18611520000000004 | 0.15996260000000004 | 0.14822340000000003 | 0.13246750000000002 | 0.1255161 |
| 0.30000000000000004 | 0.1701681 | 0.08791209 | 0.1658477 | 0.17929560000000003 | 0.1676301 | 0.164486 | 0.183161 | 0.20486440000000003 | 0.18883250000000001 | 0.18701300000000004 | 0.1717589 |
| 0.32000000000000006 | 0.2037815 | 0.15604400000000002 | 0.1461916 | 0.17289220000000002 | 0.1300578 | 0.1373832 | 0.127031 | 0.18989710000000004 | 0.1685279 | 0.20259740000000004 | 0.1750619 |
| 0.34 | 0.1827731 | 0.1208791 | 0.11793610000000002 | 0.11846320000000002 | 0.06358381 | 0.08691589000000001 | 0.10044309999999998 | 0.10944810000000002 | 0.1258883 | 0.1411255 | 0.15937240000000003 |
| 0.36000000000000004 | 0.1302521 | 0.15384620000000002 | 0.08476658000000004 | 0.07684098 | 0.046242769999999996 | 0.05514018999999999 | 0.045790250000000005 | 0.07296539000000002 | 0.09441625000000001 | 0.06753246000000002 | 0.10074320000000002 |
| 0.38000000000000006 | 0.07563026000000002 | 0.1186813 | 0.03808354 | 0.049092850000000014 | 0.03179191000000001 | 0.04299065 | 0.02215657 | 0.046772680000000004 | 0.05380711 | 0.04935065000000001 | 0.05367464999999999 |
| 0.4 | 0.04831933000000001 | 0.08131867999999998 | 0.024570019999999998 | 0.025613660000000003 | 0.011560690000000002 | 0.020560749999999996 | 0.01477105 | 0.018709070000000005 | 0.01725888 | 0.03809524 | 0.047068540000000006 |
| 0.42000000000000004 | 0.018907560000000004 | 0.08131867999999998 | 0.012285009999999999 | 0.01067236 | 0.008670520000000003 | 0.008411215000000001 | 0.008862630000000005 | 0.0121609 | 0.006091371000000001 | 0.019913420000000005 | 0.01321222 |
| 0.44 | 0.014705880000000001 | 0.04175824 | 0.009828010000000005 | 0.007470651000000003 | 0.005780347000000001 | 0.005607476 | 0.001477105 | 0.005612722 | 0.009137056000000001 | 0.01298701 | 0.01321222 |
| 0.46 | 0.0105042 | 0.02417582 | 0.0024570020000000002 | 0.0 | 0.0 | 0.0028037380000000005 | 0.001477105 | 0.0037418150000000003 | 0.004060914 | 0.006060606 | 0.005780347000000001 |
| 0.48000000000000004 | 0.0042016810000000005 | 0.01758242 | 0.0012285010000000001 | 0.001067236 | 0.002890173 | 0.0009345800000000004 | 0.001477105 | 0.0009354540000000006 | 0.002030457 | 0.001731602 | 0.004128818999999999 |
| 0.5 | 0.0 | 0.008791209 | 0.0 | 0.0 | 0.002890173 | 0.0 | 0.001477105 | 0.0009354540000000006 | 0.0 | 0.0008658010000000001 | 0.004128818999999999 |
| 0.52 | 0.00210084 | 0.002197802 | 0.0012285010000000001 | 0.002134472 | 0.0 | 0.0 | 0.0 | 0.0 | 0.001015228 | 0.0008658010000000001 | 0.0 |
| 0.54 | 0.0 | 0.002197802 | 0.0 | 0.0 | 0.0 | 0.0 | 0.001477105 | 0.0009354540000000006 | 0.001015228 | 0.0008658010000000001 | 0.0016515280000000002 |
| 0.56000000000000005 | 0.0 | 0.002197802 | 0.0 | 0.0 | 0.0 | 0.0 | 0.0 | 0.0 | 0.0 | 0.0008658010000000001 | 0.0 |
| 0.58000000000000007 | 0.0 | 0.002197802 | 0.0 | 0.001067236 | 0.0 | 0.0 | 0.0 | 0.0 | 0.0 | 0.0 | 0.0024772920000000003 |
| 0.60000000000000009 | 0.0 | 0.0 | 0.0 | 0.0 | 0.0 | 0.0009345800000000004 | 0.0 | 0.0 | 0.0 | 0.0 | 0.0 |
| 0.62000000000000011 | 0.0 | 0.0 | 0.0 | 0.0 | 0.0 | 0.0009345800000000004 | 0.0 | 0.0 | 0.0 | 0.0008658010000000001 | 0.0 |
| 0.64000000000000012 | 0.00210084 | 0.0 | 0.0 | 0.0 | 0.0 | 0.0 | 0.0 | 0.0 | 0.0 | 0.0 | 0.0 |
| 0.66000000000000014 | 0.0 | 0.0 | 0.0 | 0.0 | 0.0 | 0.0 | 0.0 | 0.0 | 0.0 | 0.0 | 0.0008257640000000005 |
| 0.68 | 0.0 | 0.0 | 0.0 | 0.0 | 0.0 | 0.0 | 0.0 | 0.0 | 0.0 | 0.0 | 0.0 |
| 0.70000000000000007 | 0.0 | 0.0 | 0.0 | 0.0 | 0.0 | 0.0 | 0.0 | 0.0 | 0.0 | 0.0 | 0.0 |
### Chart: w.t. A2
| Category | N2 A2 No.1 | N2 A2 No.2 | N2 A2 No.3 | N2 A2 No.4 | N2 A2 No.5 | N2 A2 No.6 | N2 A2 No.7 | N2 A2 No.8 | N2 A2 No.9 | N2 A2 No.10 |
|---|---|---|---|---|---|---|---|---|---|---|
| 0 | None | None | None | None | None | None | None | None | None | None |
| 2.0000000000000004E-2 | 0.01092896 | 0.01116071 | 0.02040816 | 0.003472222 | 0.0009823180000000003 | 0.002518892 | 0.0025706940000000005 | 0.0037950660000000006 | 0.0023201860000000006 | 0.0 |
| 4.0000000000000008E-2 | 0.005464481 | 0.004464286 | 0.0 | 0.0023148149999999996 | 0.0 | 0.003778338000000001 | 0.0012853470000000003 | 0.0056926 | 0.0011600930000000003 | 0.012738849999999998 |
| 6.0000000000000005E-2 | 0.0 | 0.002232143 | 0.005102041 | 0.003472222 | 0.0 | 0.0 | 0.003856041 | 0.0018975330000000003 | 0.0023201860000000006 | 0.003184713000000001 |
| 8.0000000000000016E-2 | 0.005464481 | 0.0 | 0.005102041 | 0.0023148149999999996 | 0.0009823180000000003 | 0.0 | 0.0025706940000000005 | 0.0 | 0.0011600930000000003 | 0.0 |
| 9.9999990000000025E-2 | 0.0 | 0.002232143 | 0.0 | 0.0 | 0.0019646370000000004 | 0.002518892 | 0.0 | 0.0 | 0.0011600930000000003 | 0.0 |
| 0.12000000000000001 | 0.0 | 0.0 | 0.0 | 0.0023148149999999996 | 0.0009823180000000003 | 0.001259446 | 0.0 | 0.0 | 0.0011600930000000003 | 0.0 |
| 0.14000000000000001 | 0.0 | 0.0 | 0.0 | 0.0 | 0.0029469550000000002 | 0.001259446 | 0.0012853470000000003 | 0.0037950660000000006 | 0.0 | 0.0 |
| 0.16 | 0.0 | 0.002232143 | 0.005102041 | 0.0011574070000000003 | 0.0 | 0.001259446 | 0.0 | 0.0056926 | 0.0 | 0.006369427000000001 |
| 0.18000000000000002 | 0.0 | 0.006696429000000001 | 0.0 | 0.003472222 | 0.0 | 0.0 | 0.005141388000000001 | 0.007590133 | 0.0023201860000000006 | 0.006369427000000001 |
| 0.2 | 0.005464481 | 0.002232143 | 0.01530612 | 0.010416669999999998 | 0.007858546 | 0.003778338000000001 | 0.008997429000000001 | 0.007590133 | 0.0034802780000000003 | 0.003184713000000001 |
| 0.22 | 0.005464481 | 0.008928572000000001 | 0.01020408 | 0.025462960000000003 | 0.022593320000000004 | 0.02518892 | 0.025706940000000005 | 0.0341556 | 0.010440840000000002 | 0.009554140000000004 |
| 0.24000000000000002 | 0.032786880000000004 | 0.03348214 | 0.09183674000000001 | 0.08796296 | 0.07072692000000001 | 0.05037783 | 0.06298201000000002 | 0.06641366 | 0.03944315000000001 | 0.025477710000000008 |
| 0.26 | 0.09289618000000002 | 0.08258927999999999 | 0.1173469 | 0.16550930000000003 | 0.1581532 | 0.1284635 | 0.11696660000000002 | 0.10626190000000003 | 0.1160093 | 0.05732484 |
| 0.28000000000000008 | 0.12568309999999996 | 0.1473214 | 0.1428571 | 0.2037037 | 0.21709230000000002 | 0.1939547 | 0.21850900000000004 | 0.1688805 | 0.19837589999999997 | 0.1496815 |
| 0.30000000000000004 | 0.2076503 | 0.140625 | 0.14795920000000004 | 0.17592590000000002 | 0.20726920000000001 | 0.2506297 | 0.21979430000000005 | 0.1650854 | 0.22853830000000003 | 0.20700640000000003 |
| 0.32000000000000006 | 0.22950820000000002 | 0.1741071 | 0.1326531 | 0.1377315 | 0.13948920000000004 | 0.13853900000000002 | 0.14781490000000003 | 0.12333970000000001 | 0.1635731 | 0.18471340000000006 |
| 0.34 | 0.15846990000000005 | 0.15625000000000003 | 0.11224489999999998 | 0.07060184999999998 | 0.07563851000000002 | 0.08564232000000001 | 0.07455013000000002 | 0.11574950000000002 | 0.09512761 | 0.14331210000000003 |
| 0.36000000000000004 | 0.07103825 | 0.10267859999999998 | 0.08163265 | 0.03587963000000001 | 0.03732809 | 0.05037783 | 0.05141388000000001 | 0.06451613000000002 | 0.05452436 | 0.08917198000000001 |
| 0.38000000000000006 | 0.0273224 | 0.04464285999999999 | 0.05102041 | 0.03356481 | 0.02357564 | 0.016372800000000003 | 0.032133680000000005 | 0.04933586000000001 | 0.03828306 | 0.047770700000000006 |
| 0.4 | 0.01092896 | 0.03571429 | 0.03571429 | 0.0150463 | 0.009823183000000004 | 0.027707810000000003 | 0.01413882 | 0.026565459999999996 | 0.02436195 | 0.025477710000000008 |
| 0.42000000000000004 | 0.005464481 | 0.015625 | 0.02040816 | 0.008101852 | 0.006876228000000002 | 0.0075566750000000005 | 0.0025706940000000005 | 0.026565459999999996 | 0.008120649000000002 | 0.012738849999999998 |
| 0.44 | 0.0 | 0.01785714 | 0.005102041 | 0.0023148149999999996 | 0.004911591000000001 | 0.005037783 | 0.0025706940000000005 | 0.0037950660000000006 | 0.0034802780000000003 | 0.003184713000000001 |
| 0.46 | 0.005464481 | 0.004464286 | 0.0 | 0.003472222 | 0.004911591000000001 | 0.0 | 0.0012853470000000003 | 0.0018975330000000003 | 0.0023201860000000006 | 0.009554140000000004 |
| 0.48000000000000004 | 0.0 | 0.0 | 0.0 | 0.0011574070000000003 | 0.0009823180000000003 | 0.001259446 | 0.0 | 0.0018975330000000003 | 0.0 | 0.0 |
| 0.5 | 0.0 | 0.0 | 0.0 | 0.0023148149999999996 | 0.0019646370000000004 | 0.002518892 | 0.0 | 0.0037950660000000006 | 0.0 | 0.0 |
| 0.52 | 0.0 | 0.0 | 0.0 | 0.0 | 0.0 | 0.0 | 0.0 | 0.0 | 0.0023201860000000006 | 0.003184713000000001 |
| 0.54 | 0.0 | 0.002232143 | 0.0 | 0.0 | 0.0009823180000000003 | 0.0 | 0.0 | 0.0 | 0.0 | 0.0 |
| 0.56000000000000005 | 0.0 | 0.0 | 0.0 | 0.0011574070000000003 | 0.0 | 0.0 | 0.0 | 0.0056926 | 0.0 | 0.0 |
| 0.58000000000000007 | 0.0 | 0.0 | 0.0 | 0.0 | 0.0 | 0.0 | 0.0 | 0.0 | 0.0 | 0.0 |
| 0.60000000000000009 | 0.0 | 0.002232143 | 0.0 | 0.0 | 0.0 | 0.0 | 0.0 | 0.0 | 0.0 | 0.0 |
| 0.62000000000000011 | 0.0 | 0.0 | 0.0 | 0.0011574070000000003 | 0.0 | 0.0 | 0.0 | 0.0 | 0.0 | 0.0 |
| 0.64000000000000012 | 0.0 | 0.0 | 0.0 | 0.0 | 0.0009823180000000003 | 0.0 | 0.0012853470000000003 | 0.0 | 0.0 | 0.0 |
| 0.66000000000000014 | 0.0 | 0.0 | 0.0 | 0.0 | 0.0009823180000000003 | 0.0 | 0.0012853470000000003 | 0.0 | 0.0 | 0.0 |
| 0.68 | 0.0 | 0.0 | 0.0 | 0.0 | 0.0 | 0.0 | 0.0 | 0.0 | 0.0 | 0.0 |
| 0.70000000000000007 | 0.0 | 0.0 | 0.0 | 0.0 | 0.0 | 0.0 | 0.0 | 0.0 | 0.0 | 0.0 |
### Chart: w.t. A3
| Category | N2 A3 No.1 | N2 A3 No.2 | N2 A3 No.3 | N2 A3 No.4 | N2 A3 No.5 | N2 A3 No.6 | N2 A3 No.7 | N2 A3 No.8 | N2 A3 No.9 | N2 A3 No.10 |
|---|---|---|---|---|---|---|---|---|---|---|
| 0 | None | None | None | None | None | None | None | None | None | None |
| 2.0000000000000007E-2 | 0.01077586 | 0.006818182 | 0.006430868000000004 | 0.00273224 | 0.020151130000000007 | 0.0 | 0.01444043 | 0.009463723000000004 | 0.008896797000000008 | 0.01203008 |
| 4.0000000000000015E-2 | 0.0 | 0.002272727000000001 | 0.0 | 0.005464481 | 0.01007557 | 0.0 | 0.01083033 | 0.004731861000000004 | 0.003558719000000001 | 0.009022556000000004 |
| 6.0000000000000019E-2 | 0.0 | 0.002272727000000001 | 0.0 | 0.00273224 | 0.005037783 | 0.0 | 0.0036101080000000008 | 0.0015772870000000004 | 0.001779359 | 0.003007519000000001 |
| 8.0000000000000029E-2 | 0.0 | 0.0 | 0.0 | 0.0 | 0.005037783 | 0.0 | 0.0 | 0.0015772870000000004 | 0.001779359 | 0.0015037590000000001 |
| 0.1 | 0.0 | 0.0 | 0.0 | 0.0 | 0.0 | 0.0 | 0.0 | 0.0 | 0.0 | 0.003007519000000001 |
| 0.12000000000000002 | 0.0 | 0.0 | 0.0 | 0.00273224 | 0.0 | 0.0 | 0.0 | 0.0 | 0.0 | 0.0015037590000000001 |
| 0.14000000000000001 | 0.0021551720000000008 | 0.0 | 0.0 | 0.0 | 0.0 | 0.0 | 0.0036101080000000008 | 0.0 | 0.001779359 | 0.0015037590000000001 |
| 0.16 | 0.0 | 0.0 | 0.0 | 0.00273224 | 0.0 | 0.0 | 0.0 | 0.0 | 0.0 | 0.0 |
| 0.18000000000000005 | 0.0021551720000000008 | 0.0 | 0.0 | 0.0 | 0.0 | 0.005434783000000002 | 0.007220217000000002 | 0.0 | 0.0 | 0.0 |
| 0.2 | 0.004310345000000002 | 0.0 | 0.0 | 0.00273224 | 0.0 | 0.008152174000000005 | 0.007220217000000002 | 0.0 | 0.001779359 | 0.003007519000000001 |
| 0.22 | 0.006465517 | 0.0 | 0.0 | 0.013661200000000004 | 0.0 | 0.027173910000000013 | 0.01444043 | 0.0015772870000000004 | 0.003558719000000001 | 0.003007519000000001 |
| 0.24000000000000005 | 0.02155172 | 0.0 | 0.03215434000000001 | 0.0273224 | 0.002518892 | 0.02989130000000001 | 0.05776173000000002 | 0.012618299999999997 | 0.023131670000000007 | 0.02857142999999999 |
| 0.26 | 0.09482758000000005 | 0.006818182 | 0.09003215 | 0.07923497000000003 | 0.02770781000000001 | 0.08152174000000004 | 0.1696751 | 0.05520504999999998 | 0.07295374 | 0.05263158 |
| 0.28000000000000008 | 0.14870690000000006 | 0.03863636 | 0.1672026 | 0.1775956 | 0.09823678000000002 | 0.1603261 | 0.2563177000000001 | 0.10567820000000003 | 0.13523130000000005 | 0.1503759 |
| 0.3000000000000001 | 0.21120690000000006 | 0.05909091000000003 | 0.1961415 | 0.2213115 | 0.1889169000000001 | 0.2065217 | 0.24909750000000005 | 0.1671924 | 0.19217079999999995 | 0.1924812 |
| 0.32000000000000012 | 0.1788793 | 0.12272730000000003 | 0.21221860000000006 | 0.1666667 | 0.1435768 | 0.18750000000000006 | 0.11913360000000005 | 0.1466877 | 0.18861210000000006 | 0.1849624000000001 |
| 0.34 | 0.1293103 | 0.1954546 | 0.1061093 | 0.12568309999999994 | 0.1914358 | 0.125 | 0.05054152 | 0.1735016 | 0.13879000000000005 | 0.1443609 |
| 0.3600000000000001 | 0.09482758000000005 | 0.18863640000000007 | 0.08681672 | 0.08469945000000005 | 0.09571788 | 0.08423913000000001 | 0.02166065 | 0.12302840000000002 | 0.08362989000000007 | 0.08421053000000002 |
| 0.38000000000000012 | 0.05387931 | 0.1386364 | 0.03858521 | 0.04371585 | 0.08816121 | 0.02989130000000001 | 0.0036101080000000008 | 0.07255521 | 0.08007117000000002 | 0.06015038 |
| 0.4 | 0.03232758 | 0.10909090000000003 | 0.03536978 | 0.02459016 | 0.05541562000000002 | 0.019021740000000006 | 0.007220217000000002 | 0.05047319 | 0.02491103 | 0.02706767 |
| 0.4200000000000001 | 0.004310345000000002 | 0.04545455 | 0.016077170000000005 | 0.00273224 | 0.03274559 | 0.010869570000000007 | 0.0 | 0.02996844999999999 | 0.01423488 | 0.021052629999999992 |
| 0.44 | 0.0 | 0.04545455 | 0.006430868000000004 | 0.00273224 | 0.01259446 | 0.010869570000000007 | 0.0036101080000000008 | 0.025236590000000007 | 0.01423488 | 0.007518797000000002 |
| 0.46 | 0.0 | 0.011363640000000001 | 0.0 | 0.0 | 0.01007557 | 0.0027173910000000017 | 0.0 | 0.003154574000000001 | 0.003558719000000001 | 0.006015038 |
| 0.48000000000000009 | 0.004310345000000002 | 0.018181820000000008 | 0.003215434000000001 | 0.008196721 | 0.005037783 | 0.008152174000000005 | 0.0 | 0.009463723000000004 | 0.005338078000000001 | 0.0015037590000000001 |
| 0.5 | 0.0 | 0.0 | 0.0 | 0.00273224 | 0.002518892 | 0.0027173910000000017 | 0.0 | 0.004731861000000004 | 0.0 | 0.0 |
| 0.52 | 0.0 | 0.004545454 | 0.0 | 0.0 | 0.002518892 | 0.0 | 0.0 | 0.0 | 0.0 | 0.0 |
| 0.54 | 0.0 | 0.002272727000000001 | 0.0 | 0.0 | 0.0 | 0.0 | 0.0 | 0.0 | 0.0 | 0.0 |
| 0.56000000000000005 | 0.0 | 0.0 | 0.0 | 0.0 | 0.002518892 | 0.0 | 0.0 | 0.0015772870000000004 | 0.0 | 0.0 |
| 0.58000000000000007 | 0.0 | 0.0 | 0.0 | 0.0 | 0.0 | 0.0 | 0.0 | 0.0 | 0.0 | 0.0 |
| 0.6000000000000002 | 0.0 | 0.0 | 0.003215434000000001 | 0.0 | 0.0 | 0.0 | 0.0 | 0.0 | 0.0 | 0.0 |
| 0.62000000000000022 | 0.0 | 0.0 | 0.0 | 0.0 | 0.0 | 0.0 | 0.0 | 0.0 | 0.0 | 0.0 |
| 0.64000000000000024 | 0.0 | 0.002272727000000001 | 0.0 | 0.0 | 0.0 | 0.0 | 0.0 | 0.0 | 0.0 | 0.0 |
| 0.66000000000000025 | 0.0 | 0.0 | 0.0 | 0.0 | 0.0 | 0.0 | 0.0 | 0.0 | 0.0 | 0.0 |
| 0.68 | 0.0 | 0.0 | 0.0 | 0.0 | 0.0 | 0.0 | 0.0 | 0.0 | 0.0 | 0.0015037590000000001 |
| 0.70000000000000018 | 0.0 | 0.0 | 0.0 | 0.0 | 0.0 | 0.0 | 0.0 | 0.0 | 0.0 | 0.0 |
### Chart: w.t. A5
| Category | N2 A5 No.1 | N2 A5 No.2 | N2 A5 No.3 | N2 A5 No.4 | N2 A5 No.5 | N2 A5 No.6 | N2 A5 No.7 | N2 A5 No.8 | N2 A5 No.9 | N2 A5 No.11 | N2 A5 No.12 | N2 A5 No.13 |
|---|---|---|---|---|---|---|---|---|---|---|---|---|
| 0 | None | None | None | None | None | None | None | None | None | None | None | None |
| 2.0000000000000004E-2 | 0.009876544000000004 | 0.052884620000000014 | 0.02087683 | 0.03921569 | 0.01438849 | 0.007662834999999999 | 0.03500000000000001 | 0.007393715 | 0.02072539 | 0.02434077 | 0.02902375 | 0.0 |
| 4.0000000000000008E-2 | 0.01728395 | 0.02403846 | 0.010438409999999999 | 0.0 | 0.01438849 | 0.007662834999999999 | 0.03500000000000001 | 0.004621072 | 0.005181347000000001 | 0.022312370000000005 | 0.018469660000000002 | 0.0 |
| 6.0000000000000005E-2 | 0.01481481 | 0.03365385 | 0.004175365000000001 | 0.01960784 | 0.0 | 0.0 | 0.014999999999999998 | 0.0018484290000000002 | 0.0077720210000000005 | 0.004056795000000001 | 0.013192609999999999 | 0.0 |
| 8.0000000000000016E-2 | 0.0024691360000000002 | 0.0 | 0.010438409999999999 | 0.0 | 0.0 | 0.003831418 | 0.010000000000000002 | 0.0009242140000000001 | 0.005181347000000001 | 0.008113590000000002 | 0.007915568000000001 | 0.0 |
| 0.1 | 0.0 | 0.0 | 0.006263048000000001 | 0.0 | 0.0 | 0.0 | 0.0 | 0.0018484290000000002 | 0.002590674 | 0.0020283980000000003 | 0.005277045000000001 | 0.0 |
| 0.12000000000000001 | 0.0024691360000000002 | 0.0 | 0.008350731000000004 | 0.0 | 0.0 | 0.0 | 0.0 | 0.0009242140000000001 | 0.0 | 0.0020283980000000003 | 0.005277045000000001 | 0.0 |
| 0.14000000000000001 | 0.0024691360000000002 | 0.0 | 0.002087683 | 0.0 | 0.0 | 0.0 | 0.005000000000000001 | 0.0009242140000000001 | 0.002590674 | 0.0020283980000000003 | 0.0 | 0.0 |
| 0.16 | 0.0024691360000000002 | 0.0 | 0.006263048000000001 | 0.0 | 0.0 | 0.0 | 0.005000000000000001 | 0.0009242140000000001 | 0.0 | 0.0 | 0.0 | 0.0 |
| 0.18000000000000002 | 0.0 | 0.0 | 0.004175365000000001 | 0.0 | 0.0 | 0.007662834999999999 | 0.0 | 0.004621072 | 0.002590674 | 0.0 | 0.005277045000000001 | 0.0 |
| 0.2 | 0.004938272000000001 | 0.0048076920000000006 | 0.010438409999999999 | 0.0 | 0.0 | 0.011494249999999998 | 0.0 | 0.01478743 | 0.01554404 | 0.006085193000000001 | 0.0 | 0.0 |
| 0.22 | 0.01481481 | 0.0 | 0.010438409999999999 | 0.01960784 | 0.01438849 | 0.007662834999999999 | 0.020000000000000004 | 0.0702403 | 0.018134720000000003 | 0.01419878 | 0.005277045000000001 | 0.0 |
| 0.24000000000000002 | 0.02469136 | 0.02884615 | 0.01461378 | 0.05882353 | 0.02517986 | 0.030651340000000003 | 0.05 | 0.11922370000000003 | 0.04404145 | 0.03042596 | 0.03166227 | 0.0 |
| 0.26 | 0.08641974999999998 | 0.057692310000000004 | 0.06263048000000002 | 0.07843138 | 0.05755396000000001 | 0.08429119000000002 | 0.14500000000000002 | 0.17744920000000006 | 0.1243523 | 0.07707911 | 0.07915568000000002 | 0.1 |
| 0.28000000000000008 | 0.10370370000000002 | 0.09615385000000004 | 0.08977035000000005 | 0.15686280000000002 | 0.12949640000000004 | 0.14559390000000003 | 0.165 | 0.1756007 | 0.1658031 | 0.1703854 | 0.14248020000000003 | 0.15000000000000002 |
| 0.30000000000000004 | 0.11358020000000002 | 0.1442308 | 0.125261 | 0.33333330000000005 | 0.21223020000000004 | 0.25287360000000003 | 0.24500000000000002 | 0.1691312 | 0.21243520000000002 | 0.18864100000000003 | 0.1952507 | 0.25 |
| 0.32000000000000006 | 0.1209877 | 0.14903850000000002 | 0.11691020000000002 | 0.07843138 | 0.17625900000000003 | 0.13026820000000003 | 0.125 | 0.10720890000000001 | 0.1994819 | 0.18458420000000003 | 0.1741425 | 0.05 |
| 0.34 | 0.10370370000000002 | 0.14903850000000002 | 0.125261 | 0.07843138 | 0.16187049999999997 | 0.11877390000000002 | 0.065 | 0.06192236999999999 | 0.10103630000000001 | 0.11359030000000002 | 0.1266491 | 0.2 |
| 0.36000000000000004 | 0.10864200000000002 | 0.08173077000000002 | 0.10229640000000002 | 0.01960784 | 0.10791370000000002 | 0.11877390000000002 | 0.030000000000000002 | 0.036968579999999994 | 0.02849741 | 0.08519270000000001 | 0.07651715 | 0.1 |
| 0.38000000000000006 | 0.08641974999999998 | 0.0625 | 0.06889353 | 0.1176471 | 0.05035971 | 0.05747126 | 0.010000000000000002 | 0.02310536 | 0.023316059999999996 | 0.02839757 | 0.05013192 | 0.1 |
| 0.4 | 0.0345679 | 0.04807692000000001 | 0.07933194000000002 | 0.0 | 0.01798561 | 0.0 | 0.014999999999999998 | 0.008317930000000001 | 0.0077720210000000005 | 0.01419878 | 0.018469660000000002 | 0.05 |
| 0.42000000000000004 | 0.027160489999999992 | 0.02403846 | 0.03131524000000001 | 0.0 | 0.01438849 | 0.015325670000000001 | 0.005000000000000001 | 0.008317930000000001 | 0.005181347000000001 | 0.008113590000000002 | 0.007915568000000001 | 0.0 |
| 0.44 | 0.02469136 | 0.019230770000000005 | 0.02922756 | 0.0 | 0.0 | 0.0 | 0.010000000000000002 | 0.0018484290000000002 | 0.005181347000000001 | 0.008113590000000002 | 0.0026385230000000003 | 0.0 |
| 0.46 | 0.04938272000000001 | 0.014423080000000001 | 0.016701460000000005 | 0.0 | 0.0035971220000000008 | 0.0 | 0.0 | 0.0 | 0.0 | 0.0020283980000000003 | 0.0 | 0.0 |
| 0.48000000000000004 | 0.01234568 | 0.009615385 | 0.010438409999999999 | 0.0 | 0.0 | 0.0 | 0.005000000000000001 | 0.0018484290000000002 | 0.002590674 | 0.0 | 0.0026385230000000003 | 0.0 |
| 0.5 | 0.007407407000000001 | 0.0 | 0.010438409999999999 | 0.0 | 0.0 | 0.0 | 0.0 | 0.0 | 0.0 | 0.0020283980000000003 | 0.0 | 0.0 |
| 0.52 | 0.004938272000000001 | 0.0 | 0.006263048000000001 | 0.0 | 0.0 | 0.0 | 0.0 | 0.0 | 0.0 | 0.0020283980000000003 | 0.0 | 0.0 |
| 0.54 | 0.009876544000000004 | 0.0 | 0.004175365000000001 | 0.0 | 0.0 | 0.0 | 0.005000000000000001 | 0.0 | 0.0 | 0.0 | 0.0026385230000000003 | 0.0 |
| 0.56000000000000005 | 0.0 | 0.0 | 0.004175365000000001 | 0.0 | 0.0 | 0.0 | 0.0 | 0.0 | 0.0 | 0.0 | 0.0 | 0.0 |
| 0.58000000000000007 | 0.0024691360000000002 | 0.0 | 0.0 | 0.0 | 0.0 | 0.0 | 0.0 | 0.0 | 0.0 | 0.0 | 0.0 | 0.0 |
| 0.60000000000000009 | 0.0024691360000000002 | 0.0 | 0.002087683 | 0.0 | 0.0 | 0.0 | 0.0 | 0.0 | 0.0 | 0.0 | 0.0 | 0.0 |
| 0.62000000000000011 | 0.0 | 0.0 | 0.002087683 | 0.0 | 0.0 | 0.0 | 0.0 | 0.0 | 0.0 | 0.0 | 0.0 | 0.0 |
| 0.64000000000000012 | 0.0024691360000000002 | 0.0 | 0.002087683 | 0.0 | 0.0 | 0.0 | 0.0 | 0.0 | 0.0 | 0.0 | 0.0 | 0.0 |
| 0.66000000000000014 | 0.0 | 0.0 | 0.0 | 0.0 | 0.0 | 0.0 | 0.0 | 0.0 | 0.0 | 0.0 | 0.0 | 0.0 |
| 0.68 | 0.0 | 0.0 | 0.0 | 0.0 | 0.0 | 0.0 | 0.0 | 0.0 | 0.0 | 0.0 | 0.0 | 0.0 |
| 0.70000000000000007 | 0.0 | 0.0 | 0.0 | 0.0 | 0.0 | 0.0 | 0.0 | 0.0 | 0.0 | 0.0 | 0.0 | 0.0 |
### Chart: unc-25 L4
| Category | unc-25 L4 No.1 | unc-25 L4 No.2 | unc-25 L4 No.3 | unc-25 L4 No.4 | unc-25 L4 No.5 | unc-25 L4 No.6 | unc-25 L4 No.7 | unc-25 L4 No.8 | unc-25 L4 No.9 | unc-25 L4 No.10 | unc-25 L4 No.11 | unc-25 L4 No.12 | unc-25 L4 No.13 | unc-25 L4 No.14 |
|---|---|---|---|---|---|---|---|---|---|---|---|---|---|---|
| 0 | None | None | None | None | None | None | None | None | None | None | None | None | None | None |
| 2.0000000000000007E-2 | 0.003512881 | 0.01008646 | 0.009153318 | 0.009740259 | 0.009310987000000008 | 0.01724138 | 0.048780490000000024 | 0.004451039000000002 | 0.0033333330000000017 | 0.0 | 0.006125574 | 0.011152420000000001 | 0.0033333330000000017 | 0.01385681 |
| 4.0000000000000015E-2 | 0.0011709600000000004 | 0.001440922 | 0.01601831 | 0.0 | 0.007448790000000004 | 0.03448276 | 0.12195120000000002 | 0.004451039000000002 | 0.0033333330000000017 | 0.0183727 | 0.009188361 | 0.02602231000000001 | 0.0033333330000000017 | 0.016166280000000005 |
| 6.0000000000000019E-2 | 0.003512881 | 0.005763689000000002 | 0.02745995000000001 | 0.0016233770000000005 | 0.005586592000000002 | 0.01724138 | 0.02439024 | 0.005934718000000002 | 0.0033333330000000017 | 0.01574803 | 0.015313939999999998 | 0.0037174720000000007 | 0.0016666670000000004 | 0.01154734 |
| 8.0000000000000029E-2 | 0.00234192 | 0.001440922 | 0.013729980000000001 | 0.006493506000000002 | 0.0 | 0.02155172 | 0.02439024 | 0.005934718000000002 | 0.0016666670000000004 | 0.002624672 | 0.007656968000000002 | 0.0 | 0.0016666670000000004 | 0.009237875000000001 |
| 0.1 | 0.00234192 | 0.007204611000000002 | 0.01601831 | 0.0 | 0.005586592000000002 | 0.004310345000000002 | 0.02439024 | 0.002967359000000001 | 0.0033333330000000017 | 0.007874016 | 0.006125574 | 0.0037174720000000007 | 0.0016666670000000004 | 0.009237875000000001 |
| 0.12000000000000002 | 0.008196721 | 0.001440922 | 0.01144165 | 0.0016233770000000005 | 0.0 | 0.004310345000000002 | 0.0 | 0.0014836800000000005 | 0.0 | 0.002624672 | 0.004594181 | 0.0 | 0.0 | 0.006928407000000002 |
| 0.14000000000000001 | 0.00234192 | 0.001440922 | 0.0 | 0.0 | 0.0 | 0.004310345000000002 | 0.02439024 | 0.005934718000000002 | 0.0016666670000000004 | 0.0 | 0.004594181 | 0.0037174720000000007 | 0.0016666670000000004 | 0.006928407000000002 |
| 0.16 | 0.0 | 0.007204611000000002 | 0.01144165 | 0.0 | 0.0 | 0.008620690000000004 | 0.07317073 | 0.004451039000000002 | 0.005000000000000002 | 0.002624672 | 0.009188361 | 0.0 | 0.0016666670000000004 | 0.006928407000000002 |
| 0.18000000000000005 | 0.005854801 | 0.011527380000000004 | 0.006864989 | 0.0 | 0.0 | 0.008620690000000004 | 0.02439024 | 0.016320470000000007 | 0.0016666670000000004 | 0.005249344000000002 | 0.007656968000000002 | 0.0037174720000000007 | 0.0 | 0.0 |
| 0.2 | 0.003512881 | 0.0129683 | 0.01144165 | 0.00487013 | 0.0018621970000000003 | 0.004310345000000002 | 0.02439024 | 0.011869440000000004 | 0.013333330000000001 | 0.007874016 | 0.007656968000000002 | 0.0 | 0.0016666670000000004 | 0.0 |
| 0.22 | 0.02693208 | 0.01008646 | 0.032036610000000014 | 0.0032467530000000007 | 0.003724395000000002 | 0.01293103 | 0.048780490000000024 | 0.023738869999999992 | 0.03500000000000001 | 0.02099738 | 0.01225115 | 0.0 | 0.0033333330000000017 | 0.009237875000000001 |
| 0.24000000000000005 | 0.03864169 | 0.040345819999999984 | 0.02745995000000001 | 0.01623377000000001 | 0.013035380000000001 | 0.004310345000000002 | 0.02439024 | 0.06824926 | 0.05 | 0.02624672 | 0.01990812000000001 | 0.0 | 0.011666670000000004 | 0.01154734 |
| 0.26 | 0.06908665000000003 | 0.07204611 | 0.0617849 | 0.03409091 | 0.022346370000000008 | 0.0 | 0.048780490000000024 | 0.06824926 | 0.1033333 | 0.03937008 | 0.038284840000000014 | 0.0037174720000000007 | 0.021666669999999992 | 0.023094689999999994 |
| 0.28000000000000008 | 0.12412180000000003 | 0.11815560000000003 | 0.07780320000000003 | 0.07305194 | 0.057728120000000015 | 0.01293103 | 0.0 | 0.09792285 | 0.15500000000000005 | 0.08136483000000004 | 0.04900460000000002 | 0.0037174720000000007 | 0.06166667000000002 | 0.03233256000000001 |
| 0.3000000000000001 | 0.1299766 | 0.11527380000000002 | 0.1281465 | 0.112013 | 0.11918060000000003 | 0.04310345 | 0.048780490000000024 | 0.13946590000000006 | 0.1366667 | 0.1312336 | 0.08882083000000006 | 0.02230483 | 0.08333334000000003 | 0.0369515 |
| 0.32000000000000012 | 0.14637 | 0.1268011 | 0.1395881 | 0.15097400000000005 | 0.13035379999999994 | 0.0862069 | 0.0 | 0.14243320000000007 | 0.15000000000000005 | 0.160105 | 0.1148545 | 0.059479549999999985 | 0.1683333 | 0.07390300000000001 |
| 0.34 | 0.1323185 | 0.11815560000000003 | 0.11670480000000003 | 0.1899351000000001 | 0.15083800000000006 | 0.1293103 | 0.048780490000000024 | 0.09495549000000007 | 0.09500000000000003 | 0.09448819 | 0.1332312 | 0.11152420000000003 | 0.17333330000000005 | 0.11316400000000003 |
| 0.3600000000000001 | 0.09953161 | 0.10951010000000003 | 0.07780320000000003 | 0.12337660000000003 | 0.1080074 | 0.0775862 | 0.09756097000000005 | 0.08160237000000005 | 0.08666667000000003 | 0.10236220000000003 | 0.12251150000000002 | 0.13754650000000004 | 0.1333333 | 0.09237875 |
| 0.38000000000000012 | 0.08313818 | 0.07348703000000002 | 0.07780320000000003 | 0.08279221 | 0.09869646000000008 | 0.12068970000000002 | 0.07317073 | 0.08011868999999999 | 0.07666667000000003 | 0.06036745000000003 | 0.09647779 | 0.1301115 | 0.1116667 | 0.10161660000000003 |
| 0.4 | 0.04566745 | 0.05763689000000002 | 0.048054920000000015 | 0.047077920000000016 | 0.07635009 | 0.1293103 | 0.02439024 | 0.047477740000000004 | 0.028333330000000007 | 0.06299213 | 0.08575804000000005 | 0.1561338 | 0.08166666000000004 | 0.07390300000000001 |
| 0.4200000000000001 | 0.02459016 | 0.03602305 | 0.02974828 | 0.050324670000000016 | 0.054003720000000026 | 0.09913793000000004 | 0.02439024 | 0.031157270000000008 | 0.020000000000000007 | 0.05511811 | 0.047473200000000014 | 0.09293681000000002 | 0.04333333000000002 | 0.07852194 |
| 0.44 | 0.01873536 | 0.027377520000000002 | 0.01830664 | 0.02922078 | 0.03910615 | 0.04741379000000002 | 0.07317073 | 0.025222549999999993 | 0.011666670000000004 | 0.02099738 | 0.03675345 | 0.07806692000000003 | 0.028333330000000007 | 0.04849885 |
| 0.46 | 0.009367681000000006 | 0.01729107 | 0.01601831 | 0.0211039 | 0.02979516 | 0.04310345 | 0.0 | 0.013353120000000001 | 0.0016666670000000004 | 0.02099738 | 0.02603369000000001 | 0.05204461 | 0.01666667 | 0.04849885 |
| 0.48000000000000009 | 0.010538639999999998 | 0.004322767 | 0.01601831 | 0.011363640000000001 | 0.027932960000000014 | 0.01724138 | 0.0 | 0.01038576 | 0.0016666670000000004 | 0.01312336 | 0.015313939999999998 | 0.04832714 | 0.014999999999999998 | 0.04849885 |
| 0.5 | 0.00234192 | 0.007204611000000002 | 0.0 | 0.01298701 | 0.013035380000000001 | 0.01724138 | 0.0 | 0.004451039000000002 | 0.0016666670000000004 | 0.01574803 | 0.01071975 | 0.01858736 | 0.008333334000000003 | 0.04618937999999999 |
| 0.52 | 0.003512881 | 0.002881844 | 0.004576659000000004 | 0.00487013 | 0.009310987000000008 | 0.01293103 | 0.0 | 0.002967359000000001 | 0.006666667000000002 | 0.01574803 | 0.009188361 | 0.01858736 | 0.010000000000000004 | 0.02540416 |
| 0.54 | 0.0011709600000000004 | 0.002881844 | 0.0022883290000000017 | 0.00487013 | 0.007448790000000004 | 0.008620690000000004 | 0.048780490000000024 | 0.0014836800000000005 | 0.0016666670000000004 | 0.007874016 | 0.007656968000000002 | 0.011152420000000001 | 0.005000000000000002 | 0.016166280000000005 |
| 0.56000000000000005 | 0.0011709600000000004 | 0.0 | 0.0 | 0.0016233770000000005 | 0.003724395000000002 | 0.004310345000000002 | 0.0 | 0.0014836800000000005 | 0.0 | 0.0 | 0.001531394 | 0.0 | 0.0016666670000000004 | 0.01154734 |
| 0.58000000000000007 | 0.0 | 0.0 | 0.0022883290000000017 | 0.0 | 0.0018621970000000003 | 0.004310345000000002 | 0.0 | 0.0 | 0.0 | 0.005249344000000002 | 0.0030627870000000017 | 0.0 | 0.0033333330000000017 | 0.006928407000000002 |
| 0.6000000000000002 | 0.0 | 0.0 | 0.0 | 0.0032467530000000007 | 0.0018621970000000003 | 0.0 | 0.02439024 | 0.0 | 0.0 | 0.0 | 0.0 | 0.0 | 0.0016666670000000004 | 0.004618937999999999 |
| 0.62000000000000022 | 0.0 | 0.0 | 0.0 | 0.0 | 0.0018621970000000003 | 0.004310345000000002 | 0.02439024 | 0.0 | 0.0 | 0.0 | 0.0 | 0.0 | 0.0 | 0.006928407000000002 |
| 0.64000000000000024 | 0.0 | 0.0 | 0.0 | 0.0 | 0.0 | 0.0 | 0.0 | 0.0 | 0.0 | 0.002624672 | 0.001531394 | 0.0 | 0.0 | 0.0 |
| 0.66000000000000025 | 0.0 | 0.0 | 0.0 | 0.0 | 0.0 | 0.0 | 0.0 | 0.0 | 0.0 | 0.0 | 0.0 | 0.0 | 0.0 | 0.0023094689999999993 |
| 0.68 | 0.0 | 0.0 | 0.0 | 0.0 | 0.0 | 0.0 | 0.0 | 0.0 | 0.0 | 0.0 | 0.001531394 | 0.0037174720000000007 | 0.0 | 0.0023094689999999993 |
| 0.70000000000000018 | 0.0 | 0.0 | 0.0 | 0.0 | 0.0 | 0.0 | 0.0 | 0.0 | 0.0016666670000000004 | 0.0 | 0.0 | 0.0 | 0.0 | 0.0 |
### Chart: unc-25 A1
| Category | unc-25 A1 No.1 | unc-25 A1 No.2 | unc-25 A1 No.3 | unc-25 A1 No.4 | unc-25 A1 No.5 | unc-25 A1 No.6 | unc-25 A1 No.7 | unc-25 A1 No.8 | unc-25 A1 No.9 | unc-25 A1 No.10 | unc-25 A1 No.11 | unc-25 A1 No.12 | unc-25 A1 No.13 | unc-25 A1 No.14 | unc-25 A1 No.15 | unc-25 A1 No.16 | unc-25 A1 No.17 | unc-25 A1 No.18 |
|---|---|---|---|---|---|---|---|---|---|---|---|---|---|---|---|---|---|---|
| 0 | None | None | None | None | None | None | None | None | None | None | None | None | None | None | None | None | None | None |
| 2.0000000000000007E-2 | 0.008205128 | 0.007123776000000002 | 0.012 | 0.007926024000000004 | 0.001573564 | 0.00234192 | 0.002849003 | 0.004892368000000002 | 0.006776379000000004 | 0.006220840000000002 | 0.008557457000000006 | 0.010554089999999999 | 0.003802281 | 0.008055236000000007 | 0.03448276 | 0.025899280000000007 | 0.01157025 | 0.007238883000000002 |
| 4.0000000000000015E-2 | 0.006153846000000002 | 0.005342832 | 0.012 | 0.007926024000000004 | 0.0070810390000000025 | 0.005854801 | 0.004273504000000002 | 0.0009784740000000002 | 0.004840271000000002 | 0.0015552100000000004 | 0.0073349630000000025 | 0.010554089999999999 | 0.01045627 | 0.005753740000000002 | 0.035632190000000015 | 0.01438849 | 0.01157025 | 0.004136505 |
| 6.0000000000000019E-2 | 0.009230769 | 0.0017809440000000005 | 0.010666670000000001 | 0.002642008 | 0.006294257000000002 | 0.012880560000000004 | 0.008547009000000003 | 0.0 | 0.004840271000000002 | 0.004665630000000001 | 0.006112469 | 0.007915568000000003 | 0.004752852000000002 | 0.005753740000000002 | 0.0183908 | 0.018705040000000003 | 0.0066115700000000015 | 0.007238883000000002 |
| 8.0000000000000029E-2 | 0.006153846000000002 | 0.003561888000000001 | 0.016000000000000007 | 0.001321004 | 0.002360346 | 0.003512881 | 0.004273504000000002 | 0.0 | 0.0009680540000000006 | 0.0 | 0.002444988000000001 | 0.0039577840000000015 | 0.003802281 | 0.006904488000000002 | 0.014942530000000004 | 0.008633094000000003 | 0.0016528930000000001 | 0.002068252 |
| 0.1 | 0.003076923000000001 | 0.003561888000000001 | 0.012 | 0.001321004 | 0.003933910000000001 | 0.00234192 | 0.004273504000000002 | 0.0 | 0.0 | 0.0 | 0.002444988000000001 | 0.001319261 | 0.0009505700000000008 | 0.002301496 | 0.010344830000000001 | 0.005755396000000002 | 0.004958678000000004 | 0.0031023780000000007 |
| 0.12000000000000002 | 0.003076923000000001 | 0.003561888000000001 | 0.013333330000000001 | 0.002642008 | 0.0031471280000000016 | 0.004683841000000002 | 0.004273504000000002 | 0.0 | 0.0009680540000000006 | 0.0 | 0.001222494 | 0.0039577840000000015 | 0.0 | 0.0011507480000000005 | 0.005747126000000002 | 0.004316547 | 0.0066115700000000015 | 0.002068252 |
| 0.14000000000000001 | 0.003076923000000001 | 0.0008904720000000003 | 0.012 | 0.0 | 0.0031471280000000016 | 0.004683841000000002 | 0.0014245010000000001 | 0.0 | 0.0019361080000000008 | 0.0015552100000000004 | 0.0 | 0.0 | 0.0 | 0.002301496 | 0.001149425 | 0.002877698 | 0.0033057850000000008 | 0.002068252 |
| 0.16 | 0.0041025639999999995 | 0.002671416 | 0.018666670000000007 | 0.003963012000000002 | 0.008654603000000004 | 0.0 | 0.002849003 | 0.0009784740000000002 | 0.0009680540000000006 | 0.0015552100000000004 | 0.002444988000000001 | 0.002638523000000001 | 0.0009505700000000008 | 0.0011507480000000005 | 0.002298851 | 0.0 | 0.0033057850000000008 | 0.001034126 |
| 0.18000000000000005 | 0.007179487000000002 | 0.002671416 | 0.02133333000000001 | 0.001321004 | 0.014948860000000001 | 0.010538639999999998 | 0.002849003 | 0.002935421 | 0.008712488000000006 | 0.0 | 0.004889976 | 0.0 | 0.0 | 0.004602992 | 0.001149425 | 0.0 | 0.0033057850000000008 | 0.0031023780000000007 |
| 0.2 | 0.009230769 | 0.01513802 | 0.032000000000000015 | 0.007926024000000004 | 0.03147128000000001 | 0.022248240000000006 | 0.008547009000000003 | 0.003913894 | 0.021297190000000008 | 0.0 | 0.009779952000000003 | 0.0 | 0.0009505700000000008 | 0.002301496 | 0.003448276000000001 | 0.0 | 0.0033057850000000008 | 0.004136505 |
| 0.22 | 0.0225641 | 0.01780944 | 0.046666670000000014 | 0.01717305 | 0.05586153000000002 | 0.03395785 | 0.009971510000000006 | 0.01369863 | 0.05033882 | 0.0 | 0.03422983000000001 | 0.001319261 | 0.005703422000000002 | 0.008055236000000007 | 0.001149425 | 0.001438849 | 0.0 | 0.006204757000000002 |
| 0.24000000000000005 | 0.03179487000000001 | 0.05877114999999998 | 0.076 | 0.01717305 | 0.1062156 | 0.06557377 | 0.019943020000000006 | 0.02837573 | 0.08131655000000002 | 0.0015552100000000004 | 0.052567240000000015 | 0.001319261 | 0.006653992000000002 | 0.008055236000000007 | 0.001149425 | 0.001438849 | 0.0066115700000000015 | 0.009307136000000008 |
| 0.26 | 0.06051282000000002 | 0.08103295 | 0.1093333 | 0.05548217 | 0.10149490000000003 | 0.11709600000000005 | 0.029914529999999995 | 0.05088063000000003 | 0.12100680000000003 | 0.004665630000000001 | 0.09290954000000003 | 0.014511870000000001 | 0.0256654 | 0.019562720000000006 | 0.006896552000000002 | 0.011510790000000003 | 0.01487603 | 0.02481903 |
| 0.28000000000000008 | 0.1015385 | 0.1024043 | 0.10400000000000002 | 0.1109643 | 0.10228170000000003 | 0.14519910000000005 | 0.09116809000000001 | 0.08610567000000002 | 0.1355276 | 0.027993780000000006 | 0.1430318 | 0.058047490000000014 | 0.055133080000000015 | 0.04602992 | 0.02068966 | 0.01582734 | 0.04628099 | 0.04033092000000002 |
| 0.3000000000000001 | 0.1241026 | 0.1166518 | 0.09066667000000005 | 0.17040950000000005 | 0.1062156 | 0.1358314 | 0.0982906 | 0.10958900000000002 | 0.1500484000000001 | 0.07465008 | 0.14180930000000005 | 0.10158310000000002 | 0.055133080000000015 | 0.09896433000000006 | 0.022988510000000007 | 0.03884892 | 0.0661157 | 0.05687694 |
| 0.32000000000000012 | 0.11384619999999998 | 0.11932320000000003 | 0.112 | 0.14795240000000007 | 0.1101495 | 0.12880559999999994 | 0.12393160000000003 | 0.09589041000000002 | 0.14908040000000006 | 0.09797823 | 0.1454768 | 0.1292876 | 0.11787069999999998 | 0.1173763 | 0.04942529000000001 | 0.0676259 | 0.10909090000000003 | 0.11582210000000003 |
| 0.34 | 0.10769230000000003 | 0.1148709 | 0.08800000000000004 | 0.13870540000000006 | 0.12273800000000003 | 0.11475410000000003 | 0.12962959999999996 | 0.09491194000000003 | 0.1219748 | 0.12130639999999998 | 0.1124694 | 0.15303430000000007 | 0.1359316 | 0.15074800000000008 | 0.06781609000000002 | 0.08201439000000003 | 0.1504132000000001 | 0.11271979999999998 |
| 0.3600000000000001 | 0.09948718 | 0.10774710000000003 | 0.07733333000000003 | 0.09775429000000004 | 0.08025177 | 0.058548009999999984 | 0.12678059999999997 | 0.09686889000000005 | 0.07454018 | 0.13841370000000006 | 0.07946210000000002 | 0.12532979999999994 | 0.14543730000000007 | 0.14154200000000006 | 0.1264368 | 0.09784172 | 0.18181820000000007 | 0.11892450000000003 |
| 0.38000000000000012 | 0.07897435999999997 | 0.09973286000000006 | 0.049333330000000036 | 0.07001321000000003 | 0.05822186999999998 | 0.049180330000000015 | 0.10113960000000002 | 0.12426610000000006 | 0.030977740000000014 | 0.1461897 | 0.05745721000000002 | 0.10686020000000003 | 0.1188213 | 0.1334868 | 0.1298851 | 0.1064748 | 0.1371901 | 0.11375390000000002 |
| 0.4 | 0.0574359 | 0.04452360000000002 | 0.02133333000000001 | 0.05416116 | 0.03855232 | 0.032786880000000004 | 0.05840456 | 0.09589041000000002 | 0.021297190000000008 | 0.1353033 | 0.03911981 | 0.06200528000000002 | 0.09885932000000006 | 0.09666283 | 0.1344828 | 0.11223020000000003 | 0.08760331 | 0.09513961000000001 |
| 0.4200000000000001 | 0.04307692 | 0.03829029 | 0.032000000000000015 | 0.03170409 | 0.01573564 | 0.02107728000000001 | 0.049857550000000014 | 0.07240704000000002 | 0.006776379000000004 | 0.1010887 | 0.02200489 | 0.07124009999999999 | 0.07034221000000003 | 0.06444189 | 0.09540230000000002 | 0.09496403000000006 | 0.05454545 | 0.08790072 |
| 0.44 | 0.04205128 | 0.01780944 | 0.016000000000000007 | 0.02377806999999999 | 0.011801730000000005 | 0.0117096 | 0.03418804 | 0.03522505 | 0.002904163000000001 | 0.059097980000000036 | 0.009779952000000003 | 0.04881267000000004 | 0.040874529999999985 | 0.02761795000000001 | 0.08735632000000003 | 0.08489209000000003 | 0.02975206999999999 | 0.06204757 |
| 0.46 | 0.020512819999999998 | 0.01335708 | 0.006666667000000002 | 0.01321004 | 0.004720693000000002 | 0.009367681000000006 | 0.03846154 | 0.02837573 | 0.002904163000000001 | 0.03421462 | 0.01100244 | 0.02902375 | 0.022813690000000008 | 0.02186421 | 0.04482759000000002 | 0.07625899 | 0.018181820000000008 | 0.04033092000000002 |
| 0.48000000000000009 | 0.015384620000000003 | 0.008904720000000005 | 0.006666667000000002 | 0.003963012000000002 | 0.000786782 | 0.00234192 | 0.014245009999999999 | 0.022504890000000007 | 0.0 | 0.02643857000000001 | 0.004889976 | 0.02902375 | 0.03136882 | 0.009205984000000004 | 0.02873563 | 0.06330935 | 0.019834710000000005 | 0.02895553 |
| 0.5 | 0.008205128 | 0.005342832 | 0.0013333330000000001 | 0.002642008 | 0.0 | 0.00234192 | 0.01139601 | 0.009784736000000004 | 0.0 | 0.007776050000000004 | 0.004889976 | 0.013192609999999999 | 0.01235741 | 0.008055236000000007 | 0.01609195 | 0.02158273 | 0.008264462000000005 | 0.025853150000000002 |
| 0.52 | 0.0041025639999999995 | 0.004452360000000002 | 0.0013333330000000001 | 0.003963012000000002 | 0.001573564 | 0.00234192 | 0.01139601 | 0.004892368000000002 | 0.0 | 0.006220840000000002 | 0.002444988000000001 | 0.006596306000000002 | 0.008555133000000006 | 0.002301496 | 0.008045977000000003 | 0.018705040000000003 | 0.004958678000000004 | 0.01034126 |
| 0.54 | 0.005128205 | 0.0008904720000000003 | 0.0013333330000000001 | 0.001321004 | 0.0 | 0.0 | 0.004273504000000002 | 0.007827789 | 0.0 | 0.0 | 0.001222494 | 0.0039577840000000015 | 0.01235741 | 0.002301496 | 0.012643680000000001 | 0.008633094000000003 | 0.0033057850000000008 | 0.004136505 |
| 0.56000000000000005 | 0.0020512819999999998 | 0.0008904720000000003 | 0.0 | 0.0 | 0.000786782 | 0.0 | 0.0 | 0.001956947000000001 | 0.0 | 0.0 | 0.0 | 0.0 | 0.0019011410000000005 | 0.0011507480000000005 | 0.009195402000000007 | 0.010071939999999998 | 0.0 | 0.002068252 |
| 0.58000000000000007 | 0.0 | 0.0008904720000000003 | 0.0 | 0.0 | 0.0 | 0.0 | 0.0 | 0.003913894 | 0.0 | 0.0015552100000000004 | 0.0 | 0.001319261 | 0.0019011410000000005 | 0.0 | 0.002298851 | 0.002877698 | 0.0 | 0.001034126 |
| 0.6000000000000002 | 0.0 | 0.0 | 0.0 | 0.001321004 | 0.0 | 0.0 | 0.0 | 0.001956947000000001 | 0.0 | 0.0 | 0.0 | 0.001319261 | 0.003802281 | 0.0011507480000000005 | 0.001149425 | 0.0 | 0.0 | 0.0031023780000000007 |
| 0.62000000000000022 | 0.0 | 0.0 | 0.0 | 0.0 | 0.0 | 0.0 | 0.0014245010000000001 | 0.0009784740000000002 | 0.0 | 0.0 | 0.0 | 0.0 | 0.0 | 0.0 | 0.001149425 | 0.002877698 | 0.0016528930000000001 | 0.0 |
| 0.64000000000000024 | 0.0 | 0.0 | 0.0 | 0.001321004 | 0.0 | 0.0 | 0.0 | 0.0 | 0.0 | 0.0 | 0.0 | 0.0 | 0.0009505700000000008 | 0.0 | 0.001149425 | 0.0 | 0.0016528930000000001 | 0.002068252 |
| 0.66000000000000025 | 0.0010256409999999999 | 0.0 | 0.0 | 0.0 | 0.0 | 0.0 | 0.0 | 0.0 | 0.0 | 0.0 | 0.0 | 0.0 | 0.0019011410000000005 | 0.0011507480000000005 | 0.002298851 | 0.0 | 0.0016528930000000001 | 0.002068252 |
| 0.68 | 0.0010256409999999999 | 0.0 | 0.0 | 0.0 | 0.0 | 0.0 | 0.0 | 0.0 | 0.0 | 0.0 | 0.0 | 0.0 | 0.0 | 0.0 | 0.0 | 0.0 | 0.0 | 0.0 |
| 0.70000000000000018 | 0.0 | 0.0 | 0.0 | 0.0 | 0.0 | 0.0 | 0.0 | 0.0 | 0.0 | 0.0 | 0.0 | 0.0 | 0.0 | 0.0 | 0.001149425 | 0.0 | 0.0 | 0.0 |
### Chart: unc-25 A2
| Category | unc-25 A2 No.1 | unc-25 A2 No.2 | unc-25 A2 No.3 | unc-25 A2 No.4 | unc-25 A2 No.5 | unc-25 A2 No.6 | unc-25 A2 No.7 | unc-25 A2 No.8 | unc-25 A2 No.9 | unc-25 A2 No.10 |
|---|---|---|---|---|---|---|---|---|---|---|
| 0 | None | None | None | None | None | None | None | None | None | None |
| 2.0000000000000007E-2 | 0.009765625000000003 | 0.005970149000000004 | 0.022522519999999997 | 0.01895735 | 0.02307692 | 0.0350665 | 0.023560209999999995 | 0.036866360000000015 | 0.022140220000000002 | 0.03036437 |
| 4.0000000000000015E-2 | 0.023437500000000007 | 0.008955223000000003 | 0.03603604000000001 | 0.009478673 | 0.02948718 | 0.03869408 | 0.01701571 | 0.021505380000000008 | 0.018450180000000007 | 0.02125506 |
| 6.0000000000000019E-2 | 0.001953125000000001 | 0.004477612000000002 | 0.009009009000000004 | 0.014218009999999998 | 0.01794872 | 0.03022975 | 0.023560209999999995 | 0.02304148 | 0.015990160000000003 | 0.02024291 |
| 8.0000000000000029E-2 | 0.00390625 | 0.007462686000000002 | 0.009009009000000004 | 0.005924171 | 0.015384620000000003 | 0.013301090000000003 | 0.009162304000000007 | 0.013824880000000005 | 0.013530140000000001 | 0.01821862 |
| 9.9999990000000039E-2 | 0.0078125 | 0.0029850750000000002 | 0.0 | 0.0023696679999999997 | 0.002564103000000001 | 0.007255139 | 0.006544502 | 0.007680492000000002 | 0.004920049000000002 | 0.01214575 |
| 0.12000000000000002 | 0.001953125000000001 | 0.0 | 0.004504504999999998 | 0.003554502000000001 | 0.005128205 | 0.0048367600000000024 | 0.003926701000000001 | 0.004608295000000002 | 0.007380074000000002 | 0.002024292000000001 |
| 0.14000000000000001 | 0.0078125 | 0.0 | 0.0 | 0.004739337000000002 | 0.005128205 | 0.007255139 | 0.002617801 | 0.004608295000000002 | 0.0024600250000000002 | 0.0010121460000000004 |
| 0.16 | 0.001953125000000001 | 0.001492537 | 0.0 | 0.001184834 | 0.007692308000000002 | 0.00120919 | 0.003926701000000001 | 0.0015360980000000005 | 0.004920049000000002 | 0.004048583 |
| 0.18000000000000005 | 0.001953125000000001 | 0.001492537 | 0.0 | 0.001184834 | 0.003846154000000001 | 0.00241838 | 0.002617801 | 0.0015360980000000005 | 0.0024600250000000002 | 0.004048583 |
| 0.2 | 0.00390625 | 0.0 | 0.004504504999999998 | 0.0023696679999999997 | 0.006410256000000002 | 0.00120919 | 0.009162304000000007 | 0.0015360980000000005 | 0.0012300120000000006 | 0.003036437 |
| 0.22 | 0.0078125 | 0.0 | 0.013513510000000001 | 0.003554502000000001 | 0.003846154000000001 | 0.003627569000000002 | 0.002617801 | 0.010752689999999999 | 0.0024600250000000002 | 0.005060729 |
| 0.24000000000000005 | 0.023437500000000007 | 0.005970149000000004 | 0.0 | 0.014218009999999998 | 0.012820510000000004 | 0.0048367600000000024 | 0.0013089010000000001 | 0.009216590000000005 | 0.0012300120000000006 | 0.006072875000000002 |
| 0.26 | 0.03515625 | 0.011940300000000003 | 0.01801802 | 0.023696679999999998 | 0.01794872 | 0.003627569000000002 | 0.009162304000000007 | 0.009216590000000005 | 0.009840098 | 0.017206480000000003 |
| 0.28000000000000008 | 0.05273438000000001 | 0.04626866 | 0.04954954999999998 | 0.06279621000000005 | 0.04230769000000004 | 0.008464329000000003 | 0.019633510000000007 | 0.03225806 | 0.019680200000000005 | 0.048583 |
| 0.3000000000000001 | 0.08007813000000001 | 0.10597020000000003 | 0.10810810000000003 | 0.13270140000000005 | 0.07307693 | 0.02297461 | 0.037958120000000005 | 0.061443930000000015 | 0.045510460000000016 | 0.0840081 |
| 0.32000000000000012 | 0.09375000000000004 | 0.16865669999999994 | 0.1621622 | 0.15284360000000005 | 0.07948718000000003 | 0.050785970000000014 | 0.06544503 | 0.08141320999999997 | 0.09225092000000003 | 0.09817814000000002 |
| 0.34 | 0.1269531 | 0.2343284 | 0.23873870000000005 | 0.1457346 | 0.1282051 | 0.08101572000000001 | 0.09685864000000004 | 0.10599080000000002 | 0.09225092000000003 | 0.1275304 |
| 0.3600000000000001 | 0.1347656 | 0.18358210000000005 | 0.12612609999999994 | 0.1149289 | 0.11666670000000005 | 0.11850060000000003 | 0.1623037 | 0.1029186 | 0.1291513 | 0.1133603 |
| 0.38000000000000012 | 0.1054688 | 0.1 | 0.04954954999999998 | 0.08886256000000003 | 0.125641 | 0.09431681 | 0.132199 | 0.11674350000000003 | 0.13776140000000006 | 0.08704454000000003 |
| 0.4 | 0.07226563 | 0.05820895 | 0.07207207000000003 | 0.08293839000000003 | 0.07948718000000003 | 0.1076179 | 0.1256544 | 0.09677419000000004 | 0.09840099000000004 | 0.07692308000000002 |
| 0.4200000000000001 | 0.0625 | 0.01791045 | 0.022522519999999997 | 0.04383886 | 0.06538462000000003 | 0.09915357000000002 | 0.07198953 | 0.07987712 | 0.08487085000000003 | 0.07894737 |
| 0.44 | 0.04101563 | 0.014925370000000004 | 0.013513510000000001 | 0.014218009999999998 | 0.041025639999999995 | 0.08464329 | 0.054973820000000014 | 0.05222734 | 0.06888069 | 0.05263158 |
| 0.46 | 0.03320313 | 0.005970149000000004 | 0.027027030000000007 | 0.017772510000000002 | 0.03076923 | 0.05441354000000002 | 0.037958120000000005 | 0.04147465999999998 | 0.045510460000000016 | 0.027327940000000005 |
| 0.48000000000000009 | 0.023437500000000007 | 0.004477612000000002 | 0.009009009000000004 | 0.009478673 | 0.01666667 | 0.042321640000000015 | 0.022251310000000017 | 0.013824880000000005 | 0.027060270000000008 | 0.023279350000000008 |
| 0.5 | 0.011718750000000003 | 0.001492537 | 0.004504504999999998 | 0.007109005 | 0.010256409999999999 | 0.021765419999999997 | 0.013089000000000003 | 0.021505380000000008 | 0.017220170000000007 | 0.0111336 |
| 0.52 | 0.013671880000000004 | 0.0029850750000000002 | 0.0 | 0.010663510000000001 | 0.008974359000000003 | 0.0120919 | 0.019633510000000007 | 0.016897080000000005 | 0.011070110000000001 | 0.01012146 |
| 0.54 | 0.00390625 | 0.0029850750000000002 | 0.0 | 0.003554502000000001 | 0.008974359000000003 | 0.01571947 | 0.007853403000000004 | 0.007680492000000002 | 0.008610087 | 0.005060729 |
| 0.56000000000000005 | 0.005859375000000002 | 0.001492537 | 0.0 | 0.005924171 | 0.01153846 | 0.008464329000000003 | 0.002617801 | 0.007680492000000002 | 0.008610087 | 0.004048583 |
| 0.58000000000000007 | 0.001953125000000001 | 0.0 | 0.0 | 0.0 | 0.002564103000000001 | 0.0120919 | 0.002617801 | 0.004608295000000002 | 0.0024600250000000002 | 0.0010121460000000004 |
| 0.6000000000000002 | 0.001953125000000001 | 0.0 | 0.0 | 0.0 | 0.0012820510000000004 | 0.0048367600000000024 | 0.002617801 | 0.003072197000000001 | 0.003690037000000002 | 0.002024292000000001 |
| 0.62000000000000022 | 0.001953125000000001 | 0.0 | 0.0 | 0.0 | 0.002564103000000001 | 0.00120919 | 0.0013089010000000001 | 0.004608295000000002 | 0.0 | 0.0010121460000000004 |
| 0.64000000000000024 | 0.001953125000000001 | 0.0 | 0.0 | 0.0 | 0.002564103000000001 | 0.00120919 | 0.0013089010000000001 | 0.0015360980000000005 | 0.0 | 0.0010121460000000004 |
| 0.66000000000000025 | 0.0 | 0.0 | 0.0 | 0.0 | 0.0012820510000000004 | 0.003627569000000002 | 0.0013089010000000001 | 0.0 | 0.0 | 0.0 |
| 0.68 | 0.0 | 0.0 | 0.0 | 0.001184834 | 0.0 | 0.00120919 | 0.0 | 0.0015360980000000005 | 0.0 | 0.0 |
| 0.70000000000000018 | 0.0 | 0.0 | 0.0 | 0.0 | 0.0 | 0.0 | 0.002617801 | 0.0 | 0.0 | 0.0010121460000000004 |
### Chart: unc-25 A3
| Category | unc-25 A3 No.1 | unc-25 A3 No.2 | unc-25 A3 No.3 | unc-25 A3 No.4 | unc-25 A3 No.5 | unc-25 A3 No.6 | unc-25 A3 No.7 | unc-25 A3 No.8 | unc-25 A3 No.9 | unc-25 A3 No.10 |
|---|---|---|---|---|---|---|---|---|---|---|
| 0 | None | None | None | None | None | None | None | None | None | None |
| 2.0000000000000007E-2 | 0.02411874 | 0.03527337000000001 | 0.03265306000000001 | 0.026645770000000013 | 0.027127 | 0.03485255 | 0.02923077000000001 | 0.061760840000000004 | 0.06751825000000003 | 0.03259452 |
| 4.0000000000000015E-2 | 0.01298701 | 0.03174603 | 0.02857142999999999 | 0.01567398 | 0.03822441 | 0.03351206 | 0.020000000000000007 | 0.03679369 | 0.05474453000000002 | 0.02998696 |
| 6.0000000000000019E-2 | 0.02597403 | 0.02116401999999999 | 0.02857142999999999 | 0.01724138 | 0.024660910000000008 | 0.04423593000000002 | 0.032307690000000014 | 0.042049929999999985 | 0.056569339999999996 | 0.026075620000000008 |
| 8.0000000000000029E-2 | 0.018552880000000008 | 0.02469136 | 0.0122449 | 0.01253918 | 0.013563500000000003 | 0.013404830000000005 | 0.015384620000000003 | 0.03810775 | 0.03467153 | 0.01564537 |
| 0.1 | 0.009276438000000005 | 0.005291005000000002 | 0.006122449 | 0.0047021940000000016 | 0.01849568 | 0.016085790000000003 | 0.01230769 | 0.02233903 | 0.01277372 | 0.0026075620000000016 |
| 0.12000000000000002 | 0.005565863 | 0.005291005000000002 | 0.0040816320000000034 | 0.0047021940000000016 | 0.003699137000000001 | 0.008042895000000005 | 0.004615384999999998 | 0.013140600000000004 | 0.0072992700000000035 | 0.0052151240000000015 |
| 0.14000000000000001 | 0.007421150000000002 | 0.01410935 | 0.002040816000000001 | 0.0047021940000000016 | 0.002466091000000001 | 0.008042895000000005 | 0.0015384620000000004 | 0.0065703020000000036 | 0.0072992700000000035 | 0.003911343 |
| 0.16 | 0.007421150000000002 | 0.003527337000000002 | 0.006122449 | 0.003134796000000001 | 0.002466091000000001 | 0.004021448000000002 | 0.003076923000000001 | 0.01445466 | 0.01277372 | 0.0026075620000000016 |
| 0.18000000000000005 | 0.005565863 | 0.01234568 | 0.002040816000000001 | 0.0047021940000000016 | 0.003699137000000001 | 0.006702413 | 0.01230769 | 0.009198423 | 0.0018248180000000008 | 0.003911343 |
| 0.2 | 0.0148423 | 0.02292769 | 0.01020408 | 0.01567398 | 0.004932182000000002 | 0.002680965000000001 | 0.0 | 0.0065703020000000036 | 0.009124087000000005 | 0.0013037810000000004 |
| 0.22 | 0.011131730000000001 | 0.015873020000000005 | 0.0 | 0.009404388000000005 | 0.002466091000000001 | 0.0 | 0.01230769 | 0.01445466 | 0.009124087000000005 | 0.01043025 |
| 0.24000000000000005 | 0.037105750000000014 | 0.04409171 | 0.022448980000000007 | 0.023510969999999992 | 0.01233046 | 0.0 | 0.006153846000000002 | 0.021024970000000007 | 0.005474452000000002 | 0.023468059999999985 |
| 0.26 | 0.07235622000000003 | 0.09171076 | 0.03673469 | 0.04545455 | 0.027127 | 0.004021448000000002 | 0.015384620000000003 | 0.03153745 | 0.014598539999999998 | 0.05084746 |
| 0.28000000000000008 | 0.08534322999999998 | 0.1358025 | 0.040816330000000026 | 0.08150470000000004 | 0.03452528 | 0.01206434 | 0.018461540000000005 | 0.05124835999999997 | 0.03102190000000001 | 0.06649283000000003 |
| 0.3000000000000001 | 0.12615949999999998 | 0.14462079999999997 | 0.1244898 | 0.12225709999999998 | 0.0567201 | 0.02546917 | 0.03846154 | 0.08409987000000005 | 0.0620438 | 0.136897 |
| 0.32000000000000012 | 0.1335807 | 0.15520280000000006 | 0.1387755 | 0.10501570000000003 | 0.10110970000000002 | 0.04423593000000002 | 0.04307692 | 0.07095926 | 0.09854015000000003 | 0.15123860000000006 |
| 0.34 | 0.12615949999999998 | 0.07936508000000005 | 0.08775510000000003 | 0.130094 | 0.1331689 | 0.07372654 | 0.09846154000000003 | 0.10906700000000003 | 0.09671533000000003 | 0.14341590000000007 |
| 0.3600000000000001 | 0.09647495 | 0.06349207000000003 | 0.09795918000000002 | 0.1128527 | 0.1183724 | 0.12198390000000002 | 0.11846150000000003 | 0.07358739 | 0.08576642000000004 | 0.10299870000000003 |
| 0.38000000000000012 | 0.06679035 | 0.03703704000000002 | 0.08367347000000003 | 0.06269593 | 0.1134402 | 0.10187670000000003 | 0.12307690000000003 | 0.08409987000000005 | 0.08211679 | 0.07822686 |
| 0.4 | 0.03525046 | 0.02292769 | 0.04897958999999999 | 0.07366771 | 0.10850799999999998 | 0.10991960000000002 | 0.09384616000000004 | 0.052562420000000026 | 0.06569343000000002 | 0.04302476999999998 |
| 0.4200000000000001 | 0.027829310000000013 | 0.019400350000000007 | 0.06326531 | 0.04702194 | 0.04932183 | 0.10187670000000003 | 0.09384616000000004 | 0.04599212000000001 | 0.052919710000000016 | 0.02737940000000001 |
| 0.44 | 0.02411874 | 0.005291005000000002 | 0.040816330000000026 | 0.03605016 | 0.036991370000000016 | 0.07238606000000003 | 0.05846154 | 0.039421810000000015 | 0.041970799999999996 | 0.01564537 |
| 0.46 | 0.009276438000000005 | 0.003527337000000002 | 0.022448980000000007 | 0.01253918 | 0.024660910000000008 | 0.04155496 | 0.052307690000000025 | 0.03022339 | 0.03102190000000001 | 0.009126467000000003 |
| 0.48000000000000009 | 0.009276438000000005 | 0.0 | 0.0244898 | 0.01097179 | 0.02219482 | 0.03619303 | 0.03846154 | 0.010512480000000001 | 0.023722629999999988 | 0.014341590000000001 |
| 0.5 | 0.0018552880000000005 | 0.003527337000000002 | 0.01428571 | 0.0047021940000000016 | 0.01233046 | 0.02815012999999999 | 0.02307692 | 0.0065703020000000036 | 0.014598539999999998 | 0.0013037810000000004 |
| 0.52 | 0.005565863 | 0.0017636680000000001 | 0.0122449 | 0.006269592000000002 | 0.001233046 | 0.021447720000000007 | 0.018461540000000005 | 0.0065703020000000036 | 0.01277372 | 0.0 |
| 0.54 | 0.0 | 0.0 | 0.002040816000000001 | 0.001567398 | 0.002466091000000001 | 0.008042895000000005 | 0.004615384999999998 | 0.002628121000000002 | 0.003649635000000001 | 0.0 |
| 0.56000000000000005 | 0.0 | 0.0 | 0.002040816000000001 | 0.0 | 0.0 | 0.01206434 | 0.009230769 | 0.0065703020000000036 | 0.0018248180000000008 | 0.0013037810000000004 |
| 0.58000000000000007 | 0.0 | 0.0 | 0.002040816000000001 | 0.001567398 | 0.002466091000000001 | 0.00536193 | 0.0 | 0.002628121000000002 | 0.0018248180000000008 | 0.0 |
| 0.6000000000000002 | 0.0 | 0.0 | 0.0 | 0.0 | 0.0 | 0.004021448000000002 | 0.0 | 0.0013140600000000001 | 0.0 | 0.0 |
| 0.62000000000000022 | 0.0 | 0.0 | 0.002040816000000001 | 0.001567398 | 0.0 | 0.0 | 0.003076923000000001 | 0.0 | 0.0 | 0.0 |
| 0.64000000000000024 | 0.0 | 0.0 | 0.0 | 0.0 | 0.0 | 0.002680965000000001 | 0.0 | 0.002628121000000002 | 0.0 | 0.0 |
| 0.66000000000000025 | 0.0 | 0.0 | 0.0 | 0.001567398 | 0.0 | 0.0 | 0.0 | 0.0 | 0.0 | 0.0 |
| 0.68 | 0.0 | 0.0 | 0.0 | 0.0 | 0.0 | 0.0 | 0.0 | 0.0 | 0.0 | 0.0 |
| 0.70000000000000018 | 0.0 | 0.0 | 0.0 | 0.0 | 0.001233046 | 0.0 | 0.0 | 0.0013140600000000001 | 0.0 | 0.0 |
### Chart: unc-25 A5
| Category | unc-25 A5 No.1 | unc-25 A5 No.2 | unc-25 A5 No.3 | unc-25 A5 No.4 | unc-25 A5 No.5 | unc-25 A5 No.6 | unc-25 A5 No.7 | unc-25 A5 No.8 | unc-25 A5 No.9 | unc-25 A5 No.10 | unc-25 A5 No.11 | unc-25 A5 No.12 | unc-25 A5 No.13 | unc-25 A5 No.14 | unc-25 A5 No.15 | unc-25 A5 No.16 | unc-25 A5 No.17 |
|---|---|---|---|---|---|---|---|---|---|---|---|---|---|---|---|---|---|
| 0 | None | None | None | None | None | None | None | None | None | None | None | None | None | None | None | None | None |
| 2.0000000000000007E-2 | 0.06004619 | 0.06534653000000003 | 0.04112148999999999 | 0.059748430000000026 | 0.05405406000000003 | 0.10256410000000005 | 0.03071672 | 0.06109325000000002 | 0.05042017000000001 | 0.03971963 | 0.05988024000000003 | 0.05230769000000004 | 0.07476635000000002 | 0.035443040000000016 | 0.046583849999999996 | 0.05714285999999999 | 0.03783784000000002 |
| 4.0000000000000015E-2 | 0.06004619 | 0.04752474999999999 | 0.046728970000000015 | 0.07232705000000003 | 0.07807808 | 0.05128205 | 0.05119454 | 0.1061093 | 0.05042017000000001 | 0.04439252000000003 | 0.03592815 | 0.049230770000000014 | 0.037383180000000016 | 0.035443040000000016 | 0.037267080000000015 | 0.06753246000000003 | 0.05405406000000003 |
| 6.0000000000000019E-2 | 0.046189379999999995 | 0.05148514999999999 | 0.046728970000000015 | 0.08176101000000002 | 0.06306306000000003 | 0.041025639999999995 | 0.03754266 | 0.04823150999999998 | 0.039915970000000016 | 0.049065420000000026 | 0.03892216 | 0.040000000000000015 | 0.06542056 | 0.040506330000000014 | 0.062111800000000016 | 0.05194804999999998 | 0.048648649999999995 |
| 8.0000000000000029E-2 | 0.036951500000000005 | 0.04752474999999999 | 0.03925234000000001 | 0.059748430000000026 | 0.06306306000000003 | 0.015384620000000003 | 0.040955630000000014 | 0.051446940000000004 | 0.037815130000000016 | 0.02803738000000001 | 0.020958079999999997 | 0.04307692000000002 | 0.07009346000000002 | 0.027848100000000015 | 0.018633540000000007 | 0.03636364000000001 | 0.03783784000000002 |
| 0.1 | 0.016166280000000005 | 0.04356436 | 0.02616822 | 0.04402516 | 0.03303303000000001 | 0.04615384999999998 | 0.020477820000000015 | 0.019292600000000003 | 0.018907560000000007 | 0.01635514 | 0.011976050000000004 | 0.01230769 | 0.023364489999999984 | 0.01772152000000001 | 0.02795031000000001 | 0.018181820000000008 | 0.008108108000000001 |
| 0.12000000000000002 | 0.025404160000000002 | 0.04158415999999999 | 0.02616822 | 0.059748430000000026 | 0.015015020000000002 | 0.010256409999999999 | 0.02730375000000001 | 0.02893891000000001 | 0.016806720000000008 | 0.01635514 | 0.01796407000000001 | 0.015384620000000003 | 0.018691590000000008 | 0.015189870000000003 | 0.018633540000000007 | 0.015584420000000005 | 0.021621620000000005 |
| 0.14000000000000001 | 0.018475750000000003 | 0.029702970000000002 | 0.01121495 | 0.031446540000000016 | 0.015015020000000002 | 0.0 | 0.017064850000000003 | 0.016077170000000005 | 0.006302521000000002 | 0.007009346000000003 | 0.0 | 0.01846154000000001 | 0.023364489999999984 | 0.012658229999999998 | 0.018633540000000007 | 0.0077922080000000045 | 0.005405406000000002 |
| 0.16000000000000003 | 0.016166280000000005 | 0.015841580000000008 | 0.01121495 | 0.02515723 | 0.021021020000000005 | 0.010256409999999999 | 0.006825938 | 0.016077170000000005 | 0.010504200000000002 | 0.007009346000000003 | 0.0029940120000000008 | 0.01846154000000001 | 0.009345794000000006 | 0.007594937 | 0.02795031000000001 | 0.010389609999999999 | 0.010810810000000002 |
| 0.18000000000000005 | 0.01154734 | 0.015841580000000008 | 0.0 | 0.02201258 | 0.003003003000000001 | 0.005128205 | 0.010238909999999997 | 0.009646302 | 0.008403362000000003 | 0.004672897000000001 | 0.008982036000000007 | 0.01230769 | 0.018691590000000008 | 0.007594937 | 0.018633540000000007 | 0.020779220000000008 | 0.0 |
| 0.2 | 0.018475750000000003 | 0.015841580000000008 | 0.01682243000000001 | 0.012578619999999999 | 0.015015020000000002 | 0.0 | 0.013651880000000005 | 0.0064308680000000045 | 0.01260504 | 0.011682240000000003 | 0.008982036000000007 | 0.01846154000000001 | 0.014018689999999997 | 0.007594937 | 0.024844720000000004 | 0.010389609999999999 | 0.002702703000000001 |
| 0.22000000000000003 | 0.036951500000000005 | 0.011881190000000005 | 0.014953269999999998 | 0.031446540000000016 | 0.015015020000000002 | 0.010256409999999999 | 0.017064850000000003 | 0.0032154340000000014 | 0.03361345000000001 | 0.009345794000000006 | 0.014970060000000002 | 0.01846154000000001 | 0.037383180000000016 | 0.007594937 | 0.031055900000000015 | 0.031168829999999998 | 0.005405406000000002 |
| 0.24000000000000005 | 0.020785220000000007 | 0.019801980000000007 | 0.029906539999999995 | 0.012578619999999999 | 0.015015020000000002 | 0.041025639999999995 | 0.05460751000000002 | 0.012861740000000003 | 0.048319330000000014 | 0.037383180000000016 | 0.04491018000000003 | 0.049230770000000014 | 0.05607476 | 0.027848100000000015 | 0.04968944000000001 | 0.023376619999999997 | 0.002702703000000001 |
| 0.26 | 0.055427250000000004 | 0.03168317000000001 | 0.052336450000000034 | 0.04402516 | 0.03303303000000001 | 0.03589744000000002 | 0.08873720000000003 | 0.025723469999999995 | 0.06722689 | 0.030373830000000015 | 0.11077840000000001 | 0.07384614999999999 | 0.06542056 | 0.04556962000000002 | 0.052795030000000034 | 0.044155839999999995 | 0.013513510000000003 |
| 0.28000000000000008 | 0.09468822000000002 | 0.05742574 | 0.08224298999999999 | 0.06603774000000001 | 0.03603604000000001 | 0.07179487000000001 | 0.061433450000000014 | 0.03536978 | 0.10924370000000005 | 0.06074766000000003 | 0.09880240000000007 | 0.08000000000000003 | 0.07009346000000002 | 0.09113924000000002 | 0.08695652000000004 | 0.06493507000000001 | 0.03243243000000001 |
| 0.3000000000000001 | 0.08314088000000003 | 0.055445539999999995 | 0.09158879 | 0.05031446000000002 | 0.07207207000000003 | 0.10256410000000005 | 0.10921500000000005 | 0.08681672 | 0.11134450000000001 | 0.09813084000000008 | 0.08982036000000007 | 0.10461540000000003 | 0.10280370000000003 | 0.11645570000000002 | 0.09937888000000002 | 0.0935064900000001 | 0.08378378 |
| 0.32000000000000012 | 0.07621247000000003 | 0.05742574 | 0.08224298999999999 | 0.059748430000000026 | 0.07807808 | 0.10256410000000005 | 0.10921500000000005 | 0.07395498000000003 | 0.09033614000000002 | 0.09112149000000005 | 0.11976050000000008 | 0.07692308000000002 | 0.07943925000000003 | 0.1265823 | 0.09627329999999999 | 0.11688310000000002 | 0.09729730000000003 |
| 0.34000000000000008 | 0.09006929000000002 | 0.07920792000000003 | 0.11028040000000001 | 0.06603774000000001 | 0.057057060000000014 | 0.08205127999999999 | 0.09215017000000002 | 0.06109325000000002 | 0.08823530000000006 | 0.11682240000000003 | 0.08383234000000006 | 0.058461540000000006 | 0.06074766000000003 | 0.1265823 | 0.07763974999999998 | 0.06493507000000001 | 0.11081079999999999 |
| 0.3600000000000001 | 0.07852194 | 0.08712871000000001 | 0.05794393000000002 | 0.05031446000000002 | 0.07807808 | 0.08205127999999999 | 0.07508533000000002 | 0.07717042000000003 | 0.04411765000000003 | 0.1004673 | 0.07485030000000002 | 0.05538461000000003 | 0.08411215000000001 | 0.08860759000000004 | 0.07763974999999998 | 0.09090909000000003 | 0.11621620000000007 |
| 0.38000000000000012 | 0.046189379999999995 | 0.05742574 | 0.06355139999999998 | 0.02201258 | 0.05405406000000003 | 0.06666667000000003 | 0.05119454 | 0.07717042000000003 | 0.046218490000000015 | 0.06308411 | 0.04790419 | 0.05230769000000004 | 0.04205608000000003 | 0.08354431000000008 | 0.04347825999999999 | 0.05714285999999999 | 0.08648649000000001 |
| 0.4 | 0.050808310000000016 | 0.04356436 | 0.046728970000000015 | 0.031446540000000016 | 0.04204204000000001 | 0.05128205 | 0.03412969 | 0.05466238000000003 | 0.046218490000000015 | 0.06074766000000003 | 0.05688623000000003 | 0.040000000000000015 | 0.018691590000000008 | 0.03291139000000001 | 0.031055900000000015 | 0.03636364000000001 | 0.08378378 |
| 0.4200000000000001 | 0.018475750000000003 | 0.03168317000000001 | 0.02803738000000001 | 0.028301890000000007 | 0.03903904000000001 | 0.041025639999999995 | 0.017064850000000003 | 0.041800640000000014 | 0.018907560000000007 | 0.030373830000000015 | 0.0239521 | 0.021538459999999992 | 0.009345794000000006 | 0.015189870000000003 | 0.018633540000000007 | 0.028571429999999995 | 0.05405406000000003 |
| 0.44000000000000006 | 0.009237875000000001 | 0.02178218 | 0.022429910000000015 | 0.02201258 | 0.021021020000000005 | 0.015384620000000003 | 0.010238909999999997 | 0.022508040000000007 | 0.014705880000000005 | 0.01635514 | 0.008982036000000007 | 0.04615384999999998 | 0.014018689999999997 | 0.015189870000000003 | 0.018633540000000007 | 0.02597403 | 0.035135140000000016 |
| 0.46 | 0.01154734 | 0.025742569999999996 | 0.013084110000000003 | 0.006289308000000004 | 0.01801802 | 0.005128205 | 0.006825938 | 0.03536978 | 0.01260504 | 0.02102804 | 0.005988024000000003 | 0.01846154000000001 | 0.0 | 0.010126580000000003 | 0.003105590000000002 | 0.018181820000000008 | 0.008108108000000001 |
| 0.48000000000000009 | 0.0023094689999999998 | 0.01782178000000001 | 0.01121495 | 0.0 | 0.03303303000000001 | 0.005128205 | 0.010238909999999997 | 0.012861740000000003 | 0.004201681000000002 | 0.02102804 | 0.0 | 0.01230769 | 0.0 | 0.002531645999999999 | 0.009316770000000004 | 0.0 | 0.013513510000000003 |
| 0.5 | 0.006928407000000003 | 0.009900990000000005 | 0.01121495 | 0.012578619999999999 | 0.015015020000000002 | 0.005128205 | 0.003412969 | 0.0064308680000000045 | 0.004201681000000002 | 0.007009346000000003 | 0.005988024000000003 | 0.003076923000000001 | 0.0 | 0.0 | 0.0 | 0.005194804999999999 | 0.008108108000000001 |
| 0.52 | 0.0046189379999999995 | 0.013861390000000005 | 0.0037383180000000013 | 0.009433962000000002 | 0.009009009000000005 | 0.0 | 0.0 | 0.0 | 0.004201681000000002 | 0.007009346000000003 | 0.005988024000000003 | 0.0 | 0.004672897000000001 | 0.002531645999999999 | 0.0 | 0.002597403 | 0.008108108000000001 |
| 0.54 | 0.0023094689999999998 | 0.0 | 0.0018691590000000007 | 0.009433962000000002 | 0.009009009000000005 | 0.0 | 0.0 | 0.0 | 0.0021008400000000005 | 0.0023364489999999987 | 0.0 | 0.0 | 0.0 | 0.0 | 0.0 | 0.0 | 0.002702703000000001 |
| 0.56000000000000005 | 0.0023094689999999998 | 0.0019801980000000007 | 0.007476636000000005 | 0.003144654000000001 | 0.006006006000000002 | 0.0 | 0.0 | 0.0064308680000000045 | 0.0021008400000000005 | 0.0 | 0.0 | 0.003076923000000001 | 0.0 | 0.0 | 0.0 | 0.0 | 0.002702703000000001 |
| 0.58000000000000007 | 0.0 | 0.0019801980000000007 | 0.0018691590000000007 | 0.0 | 0.003003003000000001 | 0.0 | 0.003412969 | 0.0 | 0.0 | 0.0023364489999999987 | 0.0 | 0.0 | 0.0 | 0.0 | 0.003105590000000002 | 0.0 | 0.002702703000000001 |
| 0.6000000000000002 | 0.0 | 0.0 | 0.0 | 0.003144654000000001 | 0.003003003000000001 | 0.0 | 0.0 | 0.0 | 0.0 | 0.0 | 0.0 | 0.003076923000000001 | 0.0 | 0.0 | 0.0 | 0.0 | 0.002702703000000001 |
| 0.62000000000000022 | 0.0 | 0.0 | 0.0018691590000000007 | 0.0 | 0.0 | 0.0 | 0.0 | 0.0 | 0.0 | 0.0 | 0.0 | 0.0 | 0.0 | 0.0 | 0.0 | 0.0 | 0.0 |
| 0.64000000000000024 | 0.0 | 0.0 | 0.0 | 0.0 | 0.003003003000000001 | 0.0 | 0.0 | 0.0032154340000000014 | 0.0 | 0.0 | 0.0 | 0.003076923000000001 | 0.0 | 0.0 | 0.0 | 0.0 | 0.0 |
| 0.66000000000000025 | 0.0 | 0.0 | 0.0 | 0.0 | 0.0 | 0.0 | 0.0 | 0.0 | 0.0 | 0.0 | 0.0 | 0.0 | 0.0 | 0.0 | 0.0 | 0.0 | 0.0 |
| 0.68000000000000027 | 0.0 | 0.0 | 0.0 | 0.0 | 0.0 | 0.0 | 0.0 | 0.0 | 0.0 | 0.0 | 0.0 | 0.0 | 0.0 | 0.0 | 0.0 | 0.0 | 0.0 |
| 0.70000000000000018 | 0.0 | 0.0 | 0.0 | 0.0 | 0.0 | 0.0 | 0.0 | 0.0 | 0.0 | 0.0 | 0.0 | 0.0 | 0.0 | 0.0 | 0.0 | 0.0 | 0.0 |

## Slide 2
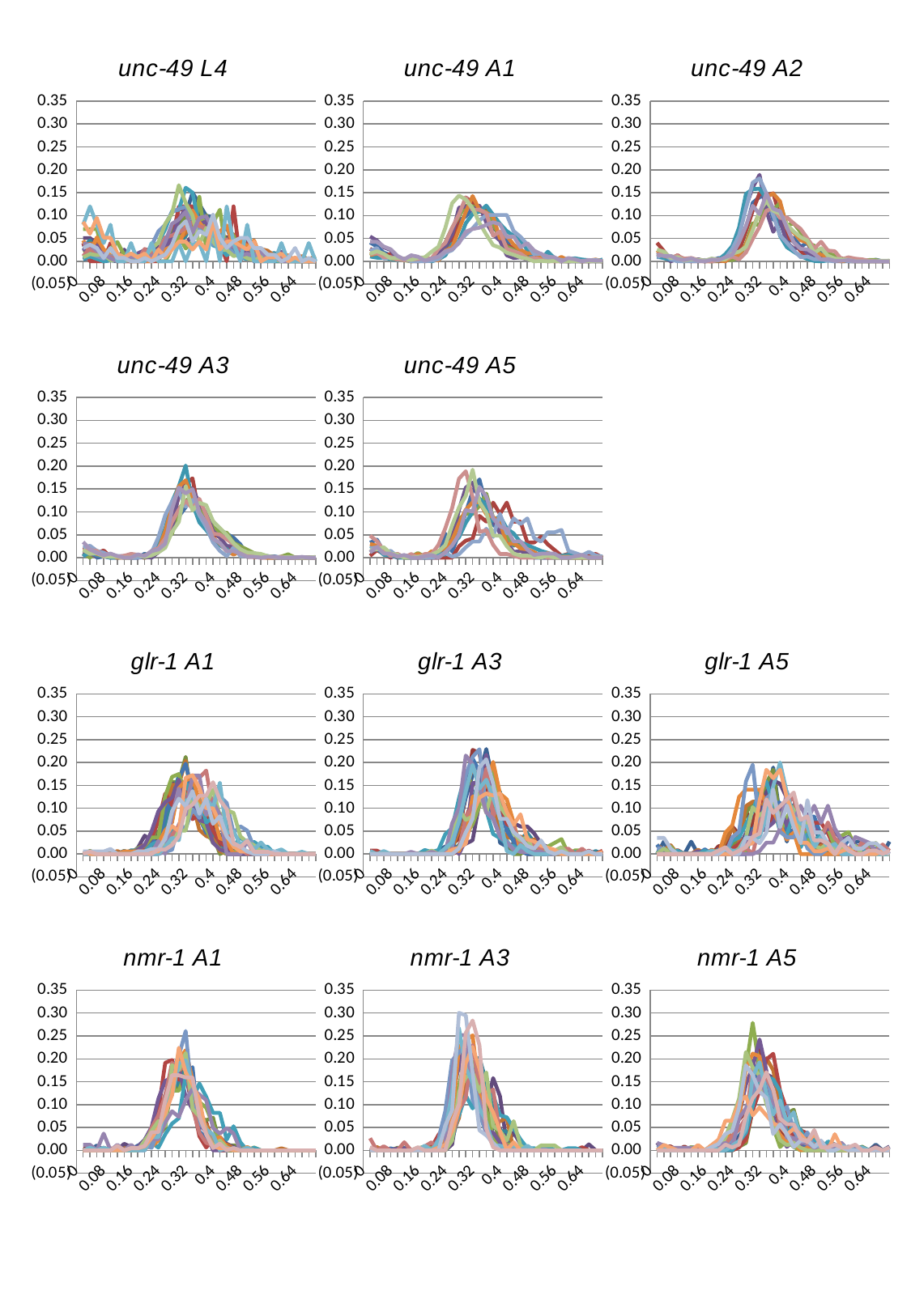

### Chart: unc-49 L4
| Category | unc-49 L4 No.1 | unc-49 L4 No.2 | unc-49 L4 No.3 | unc-49 L4 No.4 | unc-49 L4 No.5 | unc-49 L4 No.6 | unc-49 L4 No.7 | unc-49 L4 No.8 | unc-49 L4 No.9 | unc-49 L4 No.10 | unc-49 L4 No.11 | unc-49 L4 No.12 | unc-49 L4 No.13 | unc-49 L4 No.14 | unc-49 L4 No.15 | unc-49 L4 No.16 | unc-49 L4 No.17 | unc-49 L4 No.18 | unc-49 L4 No.19 |
|---|---|---|---|---|---|---|---|---|---|---|---|---|---|---|---|---|---|---|---|
| 0 | None | None | None | None | None | None | None | None | None | None | None | None | None | None | None | None | None | None | None |
| 2.0000000000000007E-2 | 0.0042283300000000015 | 0.009375000000000007 | 0.020710059999999992 | 0.050955410000000013 | 0.030674850000000007 | 0.03474903 | 0.029723989999999992 | 0.040000000000000015 | 0.07042254000000002 | 0.02807018 | 0.00536193 | 0.04575163000000003 | 0.01139601 | 0.011337870000000005 | 0.009111618 | 0.03934426 | 0.08000000000000003 | 0.0862069 | 0.029197079999999997 |
| 4.0000000000000015E-2 | 0.008456660000000006 | 0.006250000000000002 | 0.026627220000000007 | 0.050955410000000013 | 0.04601227000000002 | 0.03861004 | 0.014862000000000004 | 0.0 | 0.07042254000000002 | 0.01754386 | 0.010723860000000003 | 0.01960784 | 0.01709402 | 0.02947846 | 0.01594533 | 0.03606557 | 0.12000000000000002 | 0.06034483 | 0.03649635000000001 |
| 6.0000000000000019E-2 | 0.0021141650000000007 | 0.009375000000000007 | 0.011834320000000004 | 0.03821656 | 0.009202454000000004 | 0.03861004 | 0.010615709999999999 | 0.0 | 0.07042254000000002 | 0.03508772000000001 | 0.00536193 | 0.052287580000000014 | 0.01139601 | 0.015873020000000005 | 0.013667430000000001 | 0.02295082 | 0.08000000000000003 | 0.09482758000000005 | 0.029197079999999997 |
| 8.0000000000000029E-2 | 0.0 | 0.01875 | 0.020710059999999992 | 0.012738849999999998 | 0.012269939999999998 | 0.011583010000000001 | 0.010615709999999999 | 0.0 | 0.01408451 | 0.014035089999999998 | 0.0 | 0.026143790000000007 | 0.005698006 | 0.011337870000000005 | 0.01594533 | 0.016393440000000002 | 0.040000000000000015 | 0.051724139999999995 | 0.0072992700000000035 |
| 0.1 | 0.0021141650000000007 | 0.006250000000000002 | 0.005917160000000002 | 0.031847130000000015 | 0.02760736000000002 | 0.011583010000000001 | 0.006369427000000002 | 0.040000000000000015 | 0.02816901 | 0.01052632 | 0.002680965000000001 | 0.0130719 | 0.01139601 | 0.009070295 | 0.002277904000000001 | 0.006557377 | 0.08000000000000003 | 0.051724139999999995 | 0.029197079999999997 |
| 0.12000000000000002 | 0.0 | 0.003125000000000001 | 0.0 | 0.006369427000000002 | 0.009202454000000004 | 0.011583010000000001 | 0.004246285 | 0.0 | 0.04225352 | 0.01052632 | 0.002680965000000001 | 0.0 | 0.005698006 | 0.004535147 | 0.002277904000000001 | 0.0 | 0.0 | 0.01724138 | 0.0072992700000000035 |
| 0.14000000000000001 | 0.0021141650000000007 | 0.0 | 0.011834320000000004 | 0.025477710000000015 | 0.012269939999999998 | 0.007722008 | 0.002123142000000001 | 0.0 | 0.01408451 | 0.007017544000000001 | 0.0 | 0.006535948000000002 | 0.0 | 0.002267574000000002 | 0.002277904000000001 | 0.006557377 | 0.0 | 0.008620690000000004 | 0.0072992700000000035 |
| 0.16 | 0.0 | 0.0 | 0.002958580000000001 | 0.006369427000000002 | 0.012269939999999998 | 0.003861004 | 0.0 | 0.0 | 0.0 | 0.0035087720000000016 | 0.00536193 | 0.0 | 0.002849003 | 0.009070295 | 0.002277904000000001 | 0.009836066000000004 | 0.040000000000000015 | 0.01724138 | 0.0 |
| 0.18000000000000005 | 0.0021141650000000007 | 0.0 | 0.0 | 0.012738849999999998 | 0.003067485000000001 | 0.0 | 0.002123142000000001 | 0.0 | 0.0 | 0.01754386 | 0.002680965000000001 | 0.006535948000000002 | 0.005698006 | 0.002267574000000002 | 0.011389520000000007 | 0.006557377 | 0.0 | 0.008620690000000004 | 0.0 |
| 0.2 | 0.0021141650000000007 | 0.0 | 0.011834320000000004 | 0.025477710000000015 | 0.009202454000000004 | 0.003861004 | 0.002123142000000001 | 0.0 | 0.01408451 | 0.024561399999999997 | 0.016085790000000003 | 0.0 | 0.01139601 | 0.027210880000000007 | 0.011389520000000007 | 0.0 | 0.0 | 0.01724138 | 0.0072992700000000035 |
| 0.22 | 0.019027480000000006 | 0.003125000000000001 | 0.0147929 | 0.025477710000000015 | 0.012269939999999998 | 0.01544402 | 0.010615709999999999 | 0.0 | 0.01408451 | 0.024561399999999997 | 0.032171580000000005 | 0.0 | 0.03418804 | 0.01814059000000001 | 0.02733485 | 0.013114750000000001 | 0.040000000000000015 | 0.0 | 0.0 |
| 0.24000000000000005 | 0.01691332 | 0.028124999999999994 | 0.023668639999999994 | 0.031847130000000015 | 0.009202454000000004 | 0.007722008 | 0.010615709999999999 | 0.0 | 0.0 | 0.021052629999999992 | 0.04021448 | 0.006535948000000002 | 0.06552707000000002 | 0.022675740000000017 | 0.041002280000000016 | 0.009836066000000004 | 0.0 | 0.02586207 | 0.0072992700000000035 |
| 0.26 | 0.02536998 | 0.028124999999999994 | 0.04733728000000003 | 0.031847130000000015 | 0.02453988 | 0.027027030000000007 | 0.021231420000000008 | 0.040000000000000015 | 0.0 | 0.03157895 | 0.06970509000000003 | 0.026143790000000007 | 0.07977208000000002 | 0.05215419 | 0.08200455000000002 | 0.042622950000000014 | 0.0 | 0.008620690000000004 | 0.014598539999999998 |
| 0.28000000000000008 | 0.044397460000000034 | 0.05 | 0.07100592 | 0.05732484 | 0.058282210000000015 | 0.03861004 | 0.04670913 | 0.040000000000000015 | 0.02816901 | 0.05263158 | 0.10991960000000002 | 0.03267974000000001 | 0.10826210000000006 | 0.056689339999999984 | 0.10478360000000003 | 0.08196721000000001 | 0.0 | 0.02586207 | 0.03649635000000001 |
| 0.3000000000000001 | 0.08033827000000003 | 0.11562500000000005 | 0.07100592 | 0.07006370000000003 | 0.03987730000000001 | 0.046332050000000014 | 0.07006370000000003 | 0.12000000000000002 | 0.05633803000000001 | 0.09122807000000002 | 0.11260050000000002 | 0.03267974000000001 | 0.11396009999999998 | 0.06349207000000003 | 0.166287 | 0.09180328 | 0.040000000000000015 | 0.04310345 | 0.07299270000000002 |
| 0.32000000000000012 | 0.1014799 | 0.10937500000000003 | 0.10059170000000005 | 0.03821656 | 0.05521473000000001 | 0.061776060000000015 | 0.07430998000000003 | 0.12000000000000002 | 0.02816901 | 0.1157895 | 0.1608579 | 0.07843138 | 0.1196581 | 0.07256236000000003 | 0.1275626 | 0.10819670000000009 | 0.0 | 0.04310345 | 0.08759124000000003 |
| 0.34 | 0.1501057 | 0.11562500000000005 | 0.0739645 | 0.05732484 | 0.08895706000000007 | 0.08494209000000003 | 0.11252650000000003 | 0.12000000000000002 | 0.04225352 | 0.09824561000000002 | 0.150134 | 0.1111111 | 0.0940171 | 0.08616780000000003 | 0.10022780000000002 | 0.06229508000000002 | 0.040000000000000015 | 0.02586207 | 0.04379562000000002 |
| 0.3600000000000001 | 0.12473570000000006 | 0.08750000000000001 | 0.08579881000000003 | 0.10828030000000002 | 0.09202454000000004 | 0.06563707000000002 | 0.1104034 | 0.08000000000000003 | 0.14084510000000006 | 0.08070175 | 0.07506702000000003 | 0.08496732000000005 | 0.06837607 | 0.07709751 | 0.03644647 | 0.09508197 | 0.040000000000000015 | 0.04310345 | 0.06569343000000002 |
| 0.38000000000000012 | 0.09936575 | 0.084375 | 0.10059170000000005 | 0.050955410000000013 | 0.06134969 | 0.06949807000000002 | 0.09554140000000007 | 0.08000000000000003 | 0.02816901 | 0.0877193 | 0.06434316 | 0.06535948 | 0.05982906 | 0.07256236000000003 | 0.07289294000000003 | 0.09836066000000003 | 0.0 | 0.02586207 | 0.058394160000000014 |
| 0.4 | 0.09725159000000003 | 0.06875000000000002 | 0.062130180000000014 | 0.050955410000000013 | 0.058282210000000015 | 0.07335907 | 0.07006370000000003 | 0.08000000000000003 | 0.08450704000000006 | 0.045614030000000014 | 0.03485255 | 0.052287580000000014 | 0.03703704000000002 | 0.07256236000000003 | 0.04328017999999998 | 0.06885246000000003 | 0.08000000000000003 | 0.0862069 | 0.1021898 |
| 0.4200000000000001 | 0.04651163 | 0.05 | 0.062130180000000014 | 0.025477710000000015 | 0.058282210000000015 | 0.05019305000000002 | 0.06157111999999998 | 0.040000000000000015 | 0.11267610000000003 | 0.042105259999999985 | 0.02949062 | 0.06535948 | 0.03418804 | 0.040816330000000026 | 0.02505695 | 0.032786880000000004 | 0.0 | 0.02586207 | 0.05109489 |
| 0.44 | 0.04862578999999999 | 0.053125 | 0.03550296 | 0.031847130000000015 | 0.049079749999999984 | 0.046332050000000014 | 0.07430998000000003 | 0.0 | 0.01408451 | 0.02807018 | 0.013404830000000005 | 0.07843138 | 0.03418804 | 0.04761905 | 0.02505695 | 0.03606557 | 0.12000000000000002 | 0.04310345 | 0.029197079999999997 |
| 0.46 | 0.02325581 | 0.04062499999999999 | 0.0591716 | 0.031847130000000015 | 0.05521473000000001 | 0.05791506 | 0.03397028 | 0.12000000000000002 | 0.05633803000000001 | 0.02807018 | 0.024128689999999987 | 0.03267974000000001 | 0.01709402 | 0.03174603 | 0.011389520000000007 | 0.032786880000000004 | 0.040000000000000015 | 0.04310345 | 0.04379562000000002 |
| 0.48000000000000009 | 0.02114165 | 0.037500000000000006 | 0.0147929 | 0.04458599 | 0.04294479 | 0.03861004 | 0.025477710000000015 | 0.0 | 0.01408451 | 0.021052629999999992 | 0.008042895000000005 | 0.04575163000000003 | 0.008547009000000003 | 0.027210880000000007 | 0.01594533 | 0.013114750000000001 | 0.0 | 0.03448276 | 0.05109489 |
| 0.5 | 0.008456660000000006 | 0.015625 | 0.020710059999999992 | 0.04458599 | 0.03374233 | 0.03474903 | 0.029723989999999992 | 0.040000000000000015 | 0.01408451 | 0.021052629999999992 | 0.00536193 | 0.03267974000000001 | 0.008547009000000003 | 0.027210880000000007 | 0.0068337130000000025 | 0.016393440000000002 | 0.08000000000000003 | 0.02586207 | 0.05109489 |
| 0.52 | 0.02114165 | 0.025 | 0.0147929 | 0.0 | 0.009202454000000004 | 0.01544402 | 0.014862000000000004 | 0.040000000000000015 | 0.02816901 | 0.0 | 0.008042895000000005 | 0.04575163000000003 | 0.005698006 | 0.024943310000000014 | 0.002277904000000001 | 0.013114750000000001 | 0.0 | 0.04310345 | 0.029197079999999997 |
| 0.54 | 0.02536998 | 0.006250000000000002 | 0.0147929 | 0.006369427000000002 | 0.01533742 | 0.027027030000000007 | 0.008492569000000007 | 0.0 | 0.0 | 0.0 | 0.00536193 | 0.006535948000000002 | 0.014245009999999999 | 0.027210880000000007 | 0.009111618 | 0.02295082 | 0.0 | 0.0 | 0.029197079999999997 |
| 0.56000000000000005 | 0.01268499 | 0.009375000000000007 | 0.005917160000000002 | 0.0 | 0.01533742 | 0.02316601999999999 | 0.012738849999999998 | 0.0 | 0.01408451 | 0.007017544000000001 | 0.00536193 | 0.0 | 0.0 | 0.015873020000000005 | 0.009111618 | 0.003278689000000001 | 0.0 | 0.008620690000000004 | 0.014598539999999998 |
| 0.58000000000000007 | 0.0 | 0.003125000000000001 | 0.002958580000000001 | 0.006369427000000002 | 0.01840491 | 0.011583010000000001 | 0.008492569000000007 | 0.0 | 0.0 | 0.0 | 0.0 | 0.006535948000000002 | 0.005698006 | 0.011337870000000005 | 0.004555809000000002 | 0.009836066000000004 | 0.0 | 0.008620690000000004 | 0.014598539999999998 |
| 0.6000000000000002 | 0.0042283300000000015 | 0.006250000000000002 | 0.002958580000000001 | 0.0 | 0.003067485000000001 | 0.007722008 | 0.008492569000000007 | 0.0 | 0.0 | 0.021052629999999992 | 0.0 | 0.0130719 | 0.008547009000000003 | 0.004535147 | 0.0 | 0.0 | 0.040000000000000015 | 0.01724138 | 0.0 |
| 0.62000000000000022 | 0.0 | 0.003125000000000001 | 0.0 | 0.012738849999999998 | 0.006134969 | 0.003861004 | 0.006369427000000002 | 0.0 | 0.0 | 0.0 | 0.0 | 0.0130719 | 0.0 | 0.004535147 | 0.0 | 0.0 | 0.0 | 0.0 | 0.0072992700000000035 |
| 0.64000000000000024 | 0.0042283300000000015 | 0.003125000000000001 | 0.002958580000000001 | 0.0 | 0.006134969 | 0.003861004 | 0.006369427000000002 | 0.0 | 0.0 | 0.0035087720000000016 | 0.0 | 0.0 | 0.0 | 0.004535147 | 0.0 | 0.0 | 0.0 | 0.008620690000000004 | 0.029197079999999997 |
| 0.66000000000000025 | 0.0021141650000000007 | 0.003125000000000001 | 0.0 | 0.0 | 0.003067485000000001 | 0.011583010000000001 | 0.0 | 0.0 | 0.0 | 0.0035087720000000016 | 0.0 | 0.0 | 0.0 | 0.006802721000000002 | 0.0 | 0.0 | 0.0 | 0.0 | 0.0 |
| 0.68 | 0.0 | 0.0 | 0.0 | 0.006369427000000002 | 0.003067485000000001 | 0.003861004 | 0.0 | 0.0 | 0.0 | 0.0035087720000000016 | 0.0 | 0.0 | 0.0 | 0.0 | 0.0 | 0.0 | 0.040000000000000015 | 0.0 | 0.0072992700000000035 |
| 0.70000000000000018 | 0.0 | 0.0 | 0.0 | 0.0 | 0.003067485000000001 | 0.011583010000000001 | 0.0 | 0.0 | 0.0 | 0.0 | 0.0 | 0.0 | 0.0 | 0.004535147 | 0.0 | 0.0 | 0.0 | 0.0 | 0.0 |
### Chart: unc-49 A1
| Category | unc-49 A1 No.1 | unc-49 A1 No.2 | unc-49 A1 No.3 | unc-49 A1 No.4 | unc-49 A1 No.5 | unc-49 A1 No.6 | unc-49 A1 No.7 | unc-49 A1 No.8 | unc-49 A1 No.9 | unc-49 A1 No.10 |
|---|---|---|---|---|---|---|---|---|---|---|
| 0 | None | None | None | None | None | None | None | None | None | None |
| 2.0000000000000007E-2 | 0.04130809 | 0.02735978 | 0.017709560000000003 | 0.054172770000000016 | 0.010582010000000001 | 0.013487480000000001 | 0.02300614 | 0.015604680000000001 | 0.016574590000000007 | 0.04270987 |
| 4.0000000000000015E-2 | 0.032702240000000014 | 0.02051984 | 0.011806380000000003 | 0.04538799000000003 | 0.007936508000000004 | 0.017341040000000002 | 0.029141100000000007 | 0.018205460000000003 | 0.02209945 | 0.04270987 |
| 6.0000000000000019E-2 | 0.018932880000000006 | 0.01094391 | 0.01416765 | 0.030746710000000007 | 0.007936508000000004 | 0.013487480000000001 | 0.006134969 | 0.009102731000000003 | 0.016574590000000007 | 0.032400590000000014 |
| 8.0000000000000029E-2 | 0.013769360000000001 | 0.016415870000000006 | 0.008264462000000005 | 0.024890190000000006 | 0.005291005000000002 | 0.0077071290000000035 | 0.009202454000000004 | 0.009102731000000003 | 0.006629834 | 0.026509570000000007 |
| 0.1 | 0.006884682 | 0.006839945000000002 | 0.003541913000000001 | 0.005856514999999998 | 0.002645503000000001 | 0.003853565 | 0.006134969 | 0.0039011700000000007 | 0.007734807000000002 | 0.011782030000000004 |
| 0.12000000000000002 | 0.003442341 | 0.004103967000000002 | 0.002361275 | 0.005856514999999998 | 0.003968254000000002 | 0.0057803470000000025 | 0.004601227000000002 | 0.0026007800000000018 | 0.005524862000000002 | 0.0044182620000000035 |
| 0.14000000000000001 | 0.00172117 | 0.0 | 0.0 | 0.007320644000000002 | 0.0013227510000000005 | 0.0 | 0.0 | 0.0039011700000000007 | 0.003314917000000001 | 0.01325479 |
| 0.16 | 0.005163511 | 0.0 | 0.003541913000000001 | 0.001464129 | 0.0 | 0.001926782000000001 | 0.0 | 0.0052015600000000035 | 0.007734807000000002 | 0.010309279999999999 |
| 0.18000000000000005 | 0.003442341 | 0.0013679890000000004 | 0.002361275 | 0.004392385999999998 | 0.003968254000000002 | 0.0 | 0.0 | 0.006501951000000002 | 0.008839779 | 0.0014727540000000004 |
| 0.2 | 0.00172117 | 0.002735978000000001 | 0.00472255 | 0.004392385999999998 | 0.0 | 0.003853565 | 0.0015337420000000005 | 0.006501951000000002 | 0.020994479999999992 | 0.005891016 |
| 0.22 | 0.01204819 | 0.009575923000000004 | 0.01416765 | 0.016105420000000006 | 0.003968254000000002 | 0.001926782000000001 | 0.004601227000000002 | 0.027308190000000006 | 0.032044200000000016 | 0.010309279999999999 |
| 0.24000000000000005 | 0.02065405 | 0.01367989 | 0.041322310000000015 | 0.03513909 | 0.013227510000000001 | 0.02312138999999999 | 0.01840491 | 0.040312090000000037 | 0.07292818 | 0.022091310000000013 |
| 0.26 | 0.04819277000000002 | 0.04240766000000002 | 0.07319953 | 0.05856514999999999 | 0.03835979 | 0.030828519999999998 | 0.02453988 | 0.07282184999999998 | 0.12707179999999996 | 0.026509570000000007 |
| 0.28000000000000008 | 0.08950086000000007 | 0.09986320000000004 | 0.09917355000000004 | 0.11713030000000002 | 0.04365079 | 0.06936416 | 0.04294479 | 0.10273080000000002 | 0.14364640000000006 | 0.03976436 |
| 0.3000000000000001 | 0.09810671 | 0.1395349 | 0.12160570000000004 | 0.1200586 | 0.08333334000000003 | 0.1078998 | 0.058282210000000015 | 0.1391417 | 0.1348066 | 0.06480118000000001 |
| 0.32000000000000012 | 0.11531839999999996 | 0.127223 | 0.12042500000000003 | 0.1127379 | 0.10317460000000005 | 0.1425819 | 0.07208589000000001 | 0.11183360000000002 | 0.11602210000000003 | 0.07069220000000002 |
| 0.34 | 0.11359720000000002 | 0.1176471 | 0.10861870000000003 | 0.12152270000000005 | 0.10846560000000004 | 0.1117534 | 0.08895706000000007 | 0.11313390000000002 | 0.08397790000000005 | 0.0736377 |
| 0.3600000000000001 | 0.1084337 | 0.1108071 | 0.10861870000000003 | 0.08491948 | 0.12169310000000005 | 0.10597300000000003 | 0.10122700000000003 | 0.10533160000000003 | 0.058563530000000016 | 0.07952872 |
| 0.38000000000000012 | 0.07228914999999997 | 0.07934336000000003 | 0.08146399 | 0.05856514999999999 | 0.1018519 | 0.09826589000000006 | 0.10122700000000003 | 0.05591678000000002 | 0.03535911 | 0.08541974 |
| 0.4 | 0.06196214 | 0.05335157 | 0.05312869 | 0.04538799000000003 | 0.08201058 | 0.05009634000000002 | 0.10122700000000003 | 0.0650195 | 0.029834250000000007 | 0.08100148 |
| 0.4200000000000001 | 0.04302926 | 0.03419973 | 0.03187721000000003 | 0.01317716 | 0.06613757 | 0.05587669000000002 | 0.10122700000000003 | 0.027308190000000006 | 0.018784530000000008 | 0.05301915 |
| 0.44 | 0.02065405 | 0.03009576 | 0.02479339 | 0.007320644000000002 | 0.05555555999999998 | 0.036608860000000014 | 0.06595092 | 0.02210662999999999 | 0.01436464 | 0.054491900000000024 |
| 0.46 | 0.025817560000000007 | 0.017783860000000005 | 0.018890200000000003 | 0.008784773000000001 | 0.04629629000000001 | 0.02504817000000001 | 0.05368098 | 0.016905070000000008 | 0.008839779 | 0.03092784 |
| 0.48000000000000009 | 0.015490530000000004 | 0.01367989 | 0.008264462000000005 | 0.004392385999999998 | 0.02513227000000001 | 0.017341040000000002 | 0.036809820000000014 | 0.006501951000000002 | 0.005524862000000002 | 0.03976436 |
| 0.5 | 0.013769360000000001 | 0.009575923000000004 | 0.00472255 | 0.005856514999999998 | 0.005291005000000002 | 0.01926782000000001 | 0.01533742 | 0.007802341000000003 | 0.0 | 0.023564059999999987 |
| 0.52 | 0.006884682 | 0.0054719560000000035 | 0.01062574 | 0.004392385999999998 | 0.003968254000000002 | 0.009633912 | 0.016871170000000008 | 0.0039011700000000007 | 0.0011049720000000005 | 0.016200290000000003 |
| 0.54 | 0.003442341 | 0.002735978000000001 | 0.0 | 0.0 | 0.02116401999999999 | 0.0057803470000000025 | 0.003067485000000001 | 0.0 | 0.0 | 0.010309279999999999 |
| 0.56000000000000005 | 0.00172117 | 0.0 | 0.005903188000000002 | 0.001464129 | 0.006613757 | 0.001926782000000001 | 0.006134969 | 0.0 | 0.0011049720000000005 | 0.008836525000000005 |
| 0.58000000000000007 | 0.0 | 0.0 | 0.0011806380000000006 | 0.0 | 0.003968254000000002 | 0.009633912 | 0.003067485000000001 | 0.0013003900000000004 | 0.0 | 0.0 |
| 0.6000000000000002 | 0.0 | 0.0 | 0.0 | 0.0 | 0.005291005000000002 | 0.001926782000000001 | 0.0 | 0.0 | 0.0 | 0.007363770000000002 |
| 0.62000000000000022 | 0.0 | 0.0013679890000000004 | 0.0011806380000000006 | 0.0 | 0.006613757 | 0.001926782000000001 | 0.0 | 0.0 | 0.0 | 0.0044182620000000035 |
| 0.64000000000000024 | 0.0 | 0.0 | 0.0 | 0.0 | 0.003968254000000002 | 0.0 | 0.0 | 0.0 | 0.0 | 0.0 |
| 0.66000000000000025 | 0.0 | 0.0 | 0.002361275 | 0.0 | 0.002645503000000001 | 0.001926782000000001 | 0.0015337420000000005 | 0.0 | 0.0 | 0.0029455080000000008 |
| 0.68 | 0.0 | 0.0013679890000000004 | 0.0 | 0.0 | 0.0 | 0.003853565 | 0.003067485000000001 | 0.0 | 0.0 | 0.0014727540000000004 |
| 0.70000000000000018 | 0.0 | 0.0 | 0.0 | 0.0 | 0.003968254000000002 | 0.0 | 0.0 | 0.0 | 0.0 | 0.0014727540000000004 |
### Chart: unc-49 A2
| Category | unc-49 A2 No.1 | unc-49 A2 No.2 | unc-49 A2 No.3 | unc-49 A2 No.4 | unc-49 A2 No.5 | unc-49 A2 No.6 | unc-49 A2 No.7 | unc-49 A2 No.8 | unc-49 A2 No.9 | unc-49 A2 No.10 |
|---|---|---|---|---|---|---|---|---|---|---|
| 0 | None | None | None | None | None | None | None | None | None | None |
| 2.0000000000000007E-2 | 0.01953819 | 0.040540539999999986 | 0.01571429 | 0.009216590000000005 | 0.01057269 | 0.024271840000000013 | 0.008982036000000007 | 0.0147929 | 0.025718609999999992 | 0.01436031 |
| 4.0000000000000015E-2 | 0.012433389999999999 | 0.022522519999999997 | 0.010000000000000004 | 0.009216590000000005 | 0.007048458000000002 | 0.01213592 | 0.016467070000000007 | 0.016272190000000002 | 0.021180029999999992 | 0.011749350000000004 |
| 6.0000000000000019E-2 | 0.003552398000000001 | 0.004504504999999998 | 0.011428570000000008 | 0.0 | 0.003524229000000001 | 0.007281553000000003 | 0.004491018000000002 | 0.008875740000000007 | 0.007564296 | 0.011749350000000004 |
| 8.0000000000000029E-2 | 0.0017761990000000004 | 0.013513510000000001 | 0.007142857 | 0.004608295000000002 | 0.004405286000000001 | 0.009708738000000001 | 0.0 | 0.011834320000000004 | 0.009077156000000003 | 0.005221932 |
| 0.1 | 0.0 | 0.0022522519999999997 | 0.002857143 | 0.004608295000000002 | 0.002643172000000002 | 0.004854369 | 0.0 | 0.005917160000000002 | 0.0015128590000000005 | 0.002610966 |
| 0.12000000000000002 | 0.003552398000000001 | 0.0 | 0.002857143 | 0.0 | 0.0008810570000000006 | 0.002427184000000002 | 0.0 | 0.0073964500000000015 | 0.004538578 | 0.006527415 |
| 0.14000000000000001 | 0.0017761990000000004 | 0.0 | 0.004285714 | 0.004608295000000002 | 0.0008810570000000006 | 0.002427184000000002 | 0.0 | 0.002958580000000001 | 0.003025718000000001 | 0.001305483 |
| 0.16 | 0.0 | 0.0 | 0.001428571 | 0.0 | 0.0008810570000000006 | 0.0 | 0.0029940120000000008 | 0.00147929 | 0.0015128590000000005 | 0.0 |
| 0.18000000000000005 | 0.0017761990000000004 | 0.0 | 0.0 | 0.004608295000000002 | 0.0017621150000000005 | 0.0 | 0.0 | 0.0 | 0.006051437 | 0.003916449 |
| 0.2 | 0.007104796000000002 | 0.0 | 0.001428571 | 0.004608295000000002 | 0.003524229000000001 | 0.002427184000000002 | 0.0029940120000000008 | 0.004437870000000002 | 0.003025718000000001 | 0.002610966 |
| 0.22 | 0.005328597 | 0.0022522519999999997 | 0.002857143 | 0.0 | 0.01585903 | 0.0 | 0.007485030000000002 | 0.004437870000000002 | 0.004538578 | 0.009138381000000001 |
| 0.24000000000000005 | 0.01953819 | 0.004504504999999998 | 0.002857143 | 0.018433180000000007 | 0.03348017 | 0.01213592 | 0.02694611 | 0.01035503 | 0.016641450000000006 | 0.01436031 |
| 0.26 | 0.031971580000000006 | 0.022522519999999997 | 0.008571428 | 0.05529953999999999 | 0.07400881 | 0.01456311 | 0.056886230000000024 | 0.004437870000000002 | 0.021180029999999992 | 0.04177546 |
| 0.28000000000000008 | 0.07104795 | 0.06531531000000003 | 0.03857143 | 0.11981570000000002 | 0.1471366 | 0.03640777000000001 | 0.11526950000000002 | 0.019230770000000008 | 0.03177005 | 0.07702350000000002 |
| 0.3000000000000001 | 0.1278863 | 0.1103604 | 0.08285714000000004 | 0.16129030000000005 | 0.1577093000000001 | 0.07281554000000001 | 0.17215569999999997 | 0.04881657000000001 | 0.06807867 | 0.1214099 |
| 0.32000000000000012 | 0.1403197 | 0.1486486 | 0.07857143 | 0.1889401000000001 | 0.15859030000000007 | 0.10679610000000006 | 0.1811377 | 0.07544379 | 0.10892590000000005 | 0.1031332 |
| 0.34 | 0.1438721 | 0.14414410000000005 | 0.13 | 0.11059910000000002 | 0.1365639 | 0.1407767 | 0.1482036 | 0.11094670000000002 | 0.1316188 | 0.1488251 |
| 0.3600000000000001 | 0.09058615 | 0.1486486 | 0.11285709999999996 | 0.06451613000000003 | 0.11101320000000002 | 0.14805830000000006 | 0.10029940000000002 | 0.1153846 | 0.1104387 | 0.1148825 |
| 0.38000000000000012 | 0.09236234 | 0.09684685000000003 | 0.1271429 | 0.08755761000000002 | 0.055506610000000026 | 0.131068 | 0.056886230000000024 | 0.09467456000000006 | 0.10438729999999997 | 0.10966060000000004 |
| 0.4 | 0.04085258 | 0.054054060000000015 | 0.07857143 | 0.04147465999999998 | 0.030837010000000015 | 0.07281554000000001 | 0.04041916 | 0.09615385000000004 | 0.08018155 | 0.07310705 |
| 0.4200000000000001 | 0.05150976999999999 | 0.04279279000000003 | 0.08285714000000004 | 0.027649770000000018 | 0.021145370000000014 | 0.06310680000000002 | 0.0239521 | 0.08284023 | 0.06505295 | 0.04177546 |
| 0.44 | 0.031971580000000006 | 0.022522519999999997 | 0.06428572000000002 | 0.03225806 | 0.01145374 | 0.04611650000000001 | 0.008982036000000007 | 0.07100592 | 0.05295008 | 0.027415140000000015 |
| 0.46 | 0.04085258 | 0.01126126 | 0.040000000000000015 | 0.018433180000000007 | 0.005286343000000004 | 0.04126213 | 0.010479039999999998 | 0.05029585999999998 | 0.045385780000000014 | 0.026109660000000007 |
| 0.48000000000000009 | 0.01065719 | 0.01801802 | 0.03428571000000001 | 0.013824880000000005 | 0.0008810570000000006 | 0.01941748 | 0.004491018000000002 | 0.028106509999999994 | 0.01815431 | 0.016971280000000005 |
| 0.5 | 0.01953819 | 0.01126126 | 0.01285714 | 0.013824880000000005 | 0.0 | 0.01456311 | 0.004491018000000002 | 0.04289941000000002 | 0.02874432999999999 | 0.003916449 |
| 0.52 | 0.008880994000000007 | 0.004504504999999998 | 0.017142860000000003 | 0.004608295000000002 | 0.0017621150000000005 | 0.009708738000000001 | 0.0014970060000000004 | 0.023668639999999994 | 0.012102870000000005 | 0.005221932 |
| 0.54 | 0.005328597 | 0.0022522519999999997 | 0.01285714 | 0.0 | 0.0017621150000000005 | 0.002427184000000002 | 0.0 | 0.022189350000000007 | 0.006051437 | 0.001305483 |
| 0.56000000000000005 | 0.003552398000000001 | 0.006756757000000002 | 0.001428571 | 0.0 | 0.0 | 0.0 | 0.004491018000000002 | 0.004437870000000002 | 0.004538578 | 0.001305483 |
| 0.58000000000000007 | 0.003552398000000001 | 0.0 | 0.001428571 | 0.0 | 0.0008810570000000006 | 0.002427184000000002 | 0.0 | 0.008875740000000007 | 0.003025718000000001 | 0.002610966 |
| 0.6000000000000002 | 0.003552398000000001 | 0.0 | 0.004285714 | 0.0 | 0.0 | 0.0 | 0.0 | 0.005917160000000002 | 0.0 | 0.0 |
| 0.62000000000000022 | 0.0017761990000000004 | 0.0 | 0.001428571 | 0.0 | 0.0 | 0.0 | 0.0 | 0.004437870000000002 | 0.0 | 0.0 |
| 0.64000000000000024 | 0.0 | 0.0 | 0.002857143 | 0.0 | 0.0 | 0.0 | 0.0 | 0.00147929 | 0.0 | 0.0 |
| 0.66000000000000025 | 0.003552398000000001 | 0.0 | 0.002857143 | 0.0 | 0.0 | 0.0 | 0.0 | 0.0 | 0.0 | 0.0 |
| 0.68 | 0.0 | 0.0 | 0.0 | 0.0 | 0.0 | 0.0 | 0.0 | 0.0 | 0.0015128590000000005 | 0.0 |
| 0.70000000000000018 | 0.0 | 0.0 | 0.001428571 | 0.0 | 0.0 | 0.0 | 0.0 | 0.0 | 0.0015128590000000005 | 0.0 |
### Chart: unc-49 A3
| Category | unc-49 A3 No.1 | unc-49 A3 No.2 | unc-49 A3 No.3 | unc-49 A3 No.4 | unc-49 A3 No.5 | unc-49 A3 No.6 | unc-49 A3 No.7 | unc-49 A3 No.8 | unc-49 A3 No.9 | unc-49 A3 No.10 |
|---|---|---|---|---|---|---|---|---|---|---|
| 0 | None | None | None | None | None | None | None | None | None | None |
| 2.0000000000000007E-2 | 0.002906977 | 0.01771654 | 0.009950249000000006 | 0.01362862 | 0.003355705 | 0.01623816 | 0.02230483 | 0.01473684 | 0.01663893 | 0.03423423 |
| 4.0000000000000015E-2 | 0.02034884 | 0.02362205 | 0.0 | 0.008517887000000003 | 0.011744970000000006 | 0.01623816 | 0.02602231000000001 | 0.016842100000000006 | 0.008319467000000004 | 0.01801802 |
| 6.0000000000000019E-2 | 0.0 | 0.009842520000000004 | 0.014925370000000004 | 0.0034071550000000016 | 0.005033557000000002 | 0.008119080000000004 | 0.01486989 | 0.01473684 | 0.009983361000000001 | 0.01081081 |
| 8.0000000000000029E-2 | 0.002906977 | 0.01574803 | 0.007462686000000002 | 0.0034071550000000016 | 0.003355705 | 0.004059540000000002 | 0.011152420000000001 | 0.002105263000000001 | 0.0033277870000000018 | 0.005405406 |
| 0.1 | 0.0 | 0.0019685040000000007 | 0.002487562000000001 | 0.0 | 0.0 | 0.005412720000000002 | 0.0037174720000000007 | 0.00631579 | 0.001663893 | 0.009009009000000004 |
| 0.12000000000000002 | 0.002906977 | 0.0 | 0.002487562000000001 | 0.0 | 0.0 | 0.0 | 0.0037174720000000007 | 0.002105263000000001 | 0.001663893 | 0.003603604000000001 |
| 0.14000000000000001 | 0.0 | 0.0 | 0.0 | 0.0034071550000000016 | 0.0016778520000000007 | 0.0013531800000000005 | 0.0037174720000000007 | 0.004210526000000002 | 0.0 | 0.0 |
| 0.16 | 0.0 | 0.0 | 0.002487562000000001 | 0.0 | 0.0 | 0.0013531800000000005 | 0.0 | 0.008421052000000004 | 0.0 | 0.0 |
| 0.18000000000000005 | 0.0 | 0.0019685040000000007 | 0.002487562000000001 | 0.0 | 0.005033557000000002 | 0.0013531800000000005 | 0.0 | 0.00631579 | 0.006655574 | 0.007207207000000004 |
| 0.2 | 0.008720931000000001 | 0.0 | 0.0 | 0.0017035770000000004 | 0.003355705 | 0.005412720000000002 | 0.0037174720000000007 | 0.002105263000000001 | 0.001663893 | 0.003603604000000001 |
| 0.22 | 0.0 | 0.0019685040000000007 | 0.009950249000000006 | 0.0 | 0.013422820000000007 | 0.009472260000000005 | 0.011152420000000001 | 0.01473684 | 0.0049916800000000035 | 0.014414409999999999 |
| 0.24000000000000005 | 0.01453488 | 0.011811020000000004 | 0.014925370000000004 | 0.011925040000000001 | 0.031879190000000016 | 0.01759134 | 0.04460967 | 0.021052629999999992 | 0.011647250000000001 | 0.01801802 |
| 0.26 | 0.03197674000000001 | 0.04527558999999998 | 0.04228856 | 0.04258944 | 0.06375839 | 0.05818674 | 0.09293681000000002 | 0.042105259999999985 | 0.021630610000000012 | 0.03063063 |
| 0.28000000000000008 | 0.09883721000000002 | 0.08070866000000003 | 0.05223880999999998 | 0.061328790000000015 | 0.12248320000000003 | 0.11231389999999995 | 0.12267660000000004 | 0.07789474000000003 | 0.056572379999999985 | 0.10630630000000002 |
| 0.3000000000000001 | 0.09011628 | 0.1338583 | 0.10696520000000004 | 0.1413969 | 0.15604030000000008 | 0.15561570000000005 | 0.14498140000000007 | 0.10526320000000006 | 0.07820299000000003 | 0.1531532 |
| 0.32000000000000012 | 0.11046510000000002 | 0.16141730000000007 | 0.12437810000000002 | 0.15672910000000007 | 0.20134230000000006 | 0.1691475 | 0.11152420000000003 | 0.12421050000000003 | 0.15640600000000007 | 0.1405405 |
| 0.34 | 0.14244190000000007 | 0.17322840000000006 | 0.1343284 | 0.14991480000000004 | 0.1174497 | 0.1285521 | 0.126394 | 0.1073684 | 0.10316140000000003 | 0.14954950000000006 |
| 0.3600000000000001 | 0.11918600000000003 | 0.09251969 | 0.10696520000000004 | 0.11925040000000002 | 0.07718121000000001 | 0.09336942000000004 | 0.09293681000000002 | 0.1284211 | 0.1198003 | 0.09909910000000002 |
| 0.38000000000000012 | 0.0872093 | 0.07283465000000003 | 0.0920398 | 0.09710392 | 0.06040268000000003 | 0.07442490000000003 | 0.0669145 | 0.09263158 | 0.1148086 | 0.07567567 |
| 0.4 | 0.05232558 | 0.051181099999999986 | 0.06716418 | 0.05792164 | 0.04026844999999998 | 0.04330176 | 0.03345725 | 0.05263158 | 0.07986689000000002 | 0.041441439999999996 |
| 0.4200000000000001 | 0.061046509999999984 | 0.04527558999999998 | 0.054726370000000024 | 0.04429302000000002 | 0.026845640000000014 | 0.031123140000000007 | 0.01486989 | 0.05052632000000001 | 0.06489184000000002 | 0.03243243 |
| 0.44 | 0.05232558 | 0.02165354 | 0.054726370000000024 | 0.025553659999999992 | 0.01845638 | 0.01488498 | 0.0037174720000000007 | 0.040000000000000015 | 0.04991680000000002 | 0.014414409999999999 |
| 0.46 | 0.04360465 | 0.02165354 | 0.032338310000000016 | 0.025553659999999992 | 0.01006711 | 0.006765900000000002 | 0.02230483 | 0.029473680000000002 | 0.031613980000000014 | 0.014414409999999999 |
| 0.48000000000000009 | 0.02906977 | 0.011811020000000004 | 0.024875620000000008 | 0.010221470000000005 | 0.01006711 | 0.013531800000000004 | 0.0037174720000000007 | 0.01894737000000001 | 0.018302830000000006 | 0.009009009000000004 |
| 0.5 | 0.005813953000000002 | 0.0019685040000000007 | 0.01741293 | 0.010221470000000005 | 0.0016778520000000007 | 0.009472260000000005 | 0.0037174720000000007 | 0.008421052000000004 | 0.011647250000000001 | 0.001801802 |
| 0.52 | 0.008720931000000001 | 0.0019685040000000007 | 0.009950249000000006 | 0.005110733000000002 | 0.01006711 | 0.0013531800000000005 | 0.0074349440000000015 | 0.004210526000000002 | 0.009983361000000001 | 0.001801802 |
| 0.54 | 0.005813953000000002 | 0.0 | 0.002487562000000001 | 0.005110733000000002 | 0.0016778520000000007 | 0.0013531800000000005 | 0.0037174720000000007 | 0.0 | 0.008319467000000004 | 0.0 |
| 0.56000000000000005 | 0.002906977 | 0.0 | 0.0 | 0.0017035770000000004 | 0.003355705 | 0.0 | 0.0037174720000000007 | 0.002105263000000001 | 0.0033277870000000018 | 0.001801802 |
| 0.58000000000000007 | 0.002906977 | 0.0 | 0.0 | 0.0 | 0.0 | 0.0 | 0.0 | 0.0 | 0.001663893 | 0.001801802 |
| 0.6000000000000002 | 0.0 | 0.0 | 0.002487562000000001 | 0.0 | 0.0 | 0.0 | 0.0 | 0.002105263000000001 | 0.0 | 0.0 |
| 0.62000000000000022 | 0.002906977 | 0.0 | 0.007462686000000002 | 0.0 | 0.0 | 0.0 | 0.0 | 0.0 | 0.001663893 | 0.0 |
| 0.64000000000000024 | 0.0 | 0.0 | 0.0 | 0.0 | 0.0 | 0.0 | 0.0 | 0.0 | 0.0 | 0.0 |
| 0.66000000000000025 | 0.0 | 0.0 | 0.0 | 0.0 | 0.0 | 0.0 | 0.0 | 0.0 | 0.0 | 0.001801802 |
| 0.68 | 0.0 | 0.0 | 0.0 | 0.0 | 0.0 | 0.0 | 0.0 | 0.0 | 0.0 | 0.0 |
| 0.70000000000000018 | 0.0 | 0.0 | 0.0 | 0.0 | 0.0 | 0.0 | 0.0 | 0.0 | 0.001663893 | 0.0 |
### Chart: unc-49 A5
| Category | unc-49 A5 No.1 | unc-49 A5 No.2 | unc-49 A5 No.3 | unc-49 A5 No.4 | unc-49 A5 No.5 | unc-49 A5 No.6 | unc-49 A5 No.7 | unc-49 A5 No.8 | unc-49 A5 No.9 | unc-49 A5 No.10 |
|---|---|---|---|---|---|---|---|---|---|---|
| 0 | None | None | None | None | None | None | None | None | None | None |
| 2.0000000000000007E-2 | 0.03289474 | 0.004149378000000001 | 0.01217039 | 0.01233046 | 0.02542373 | 0.030241930000000014 | 0.02020202 | 0.04819277000000002 | 0.01601831 | 0.011869440000000004 |
| 4.0000000000000015E-2 | 0.03947368000000001 | 0.016597510000000003 | 0.020283980000000007 | 0.01849568 | 0.02118644 | 0.026209680000000006 | 0.022727270000000008 | 0.03614458 | 0.01830664 | 0.023738869999999992 |
| 6.0000000000000019E-2 | 0.01315789 | 0.008298756000000003 | 0.01419878 | 0.009864365000000007 | 0.006355931999999998 | 0.01209677 | 0.01010101 | 0.01606426 | 0.022883300000000013 | 0.008902078 |
| 8.0000000000000029E-2 | 0.0065789470000000025 | 0.0 | 0.01014199 | 0.002466091000000001 | 0.004237288000000002 | 0.01008064 | 0.015151520000000005 | 0.0040160640000000015 | 0.009153318 | 0.008902078 |
| 0.1 | 0.0 | 0.008298756000000003 | 0.002028398000000001 | 0.002466091000000001 | 0.002118644 | 0.002016129 | 0.002525253 | 0.008032128000000001 | 0.006864989 | 0.002967359000000001 |
| 0.12000000000000002 | 0.0 | 0.0 | 0.002028398000000001 | 0.004932182000000002 | 0.0 | 0.004032258000000002 | 0.005050505 | 0.0 | 0.0 | 0.005934718000000002 |
| 0.14000000000000001 | 0.0 | 0.0 | 0.002028398000000001 | 0.002466091000000001 | 0.0 | 0.002016129 | 0.0 | 0.008032128000000001 | 0.0 | 0.0 |
| 0.16 | 0.0 | 0.0 | 0.01014199 | 0.002466091000000001 | 0.002118644 | 0.008064516000000004 | 0.0 | 0.0 | 0.0022883290000000017 | 0.0 |
| 0.18000000000000005 | 0.0 | 0.0 | 0.002028398000000001 | 0.002466091000000001 | 0.0 | 0.002016129 | 0.0 | 0.008032128000000001 | 0.0022883290000000017 | 0.002967359000000001 |
| 0.2 | 0.0 | 0.0 | 0.006085193000000003 | 0.009864365000000007 | 0.002118644 | 0.0141129 | 0.0 | 0.008032128000000001 | 0.006864989 | 0.005934718000000002 |
| 0.22 | 0.0 | 0.0 | 0.01014199 | 0.00863132 | 0.01059322 | 0.0141129 | 0.002525253 | 0.02811244999999999 | 0.013729980000000001 | 0.0 |
| 0.24000000000000005 | 0.05263158 | 0.0 | 0.020283980000000007 | 0.02589396000000001 | 0.006355931999999998 | 0.02217742 | 0.01010101 | 0.06425703000000003 | 0.02745995000000001 | 0.011869440000000004 |
| 0.26 | 0.01315789 | 0.0 | 0.05679513 | 0.040690500000000004 | 0.02966102 | 0.03830645 | 0.002525253 | 0.1084337 | 0.07093821000000003 | 0.02670623 |
| 0.28000000000000008 | 0.05263158 | 0.02489627 | 0.08519270000000001 | 0.1048089 | 0.04449153000000003 | 0.07459678000000004 | 0.007575758000000004 | 0.1726908 | 0.1098398 | 0.050445099999999986 |
| 0.3000000000000001 | 0.09868421000000002 | 0.0373444 | 0.09533469000000003 | 0.15289770000000005 | 0.07627117999999997 | 0.10483870000000002 | 0.022727270000000008 | 0.18875500000000006 | 0.13501140000000006 | 0.10385759999999995 |
| 0.32000000000000012 | 0.14473680000000005 | 0.041493780000000015 | 0.09533469000000003 | 0.16399510000000006 | 0.09957627000000005 | 0.12096770000000003 | 0.03535353 | 0.1325301 | 0.1922197 | 0.10089020000000003 |
| 0.34 | 0.1710526 | 0.09128631000000001 | 0.11359030000000002 | 0.1270037 | 0.12923730000000005 | 0.12096770000000003 | 0.03535353 | 0.05622489999999998 | 0.12128150000000003 | 0.1543027 |
| 0.3600000000000001 | 0.1118421 | 0.07883818 | 0.13995940000000007 | 0.0974106 | 0.1144068 | 0.09475806000000007 | 0.06313131 | 0.060240969999999984 | 0.09839817000000005 | 0.1335312 |
| 0.38000000000000012 | 0.07236842000000003 | 0.12033199999999998 | 0.08722109 | 0.08384711 | 0.08050848000000005 | 0.08870967000000003 | 0.04545455 | 0.02811244999999999 | 0.048054920000000015 | 0.08605342 |
| 0.4 | 0.05921053000000001 | 0.09543569 | 0.07910751000000003 | 0.053020959999999985 | 0.0911017 | 0.07862903 | 0.0959596 | 0.008032128000000001 | 0.048054920000000015 | 0.06824926 |
| 0.4200000000000001 | 0.06578948 | 0.12033199999999998 | 0.04665313999999998 | 0.03329223 | 0.06355932 | 0.028225810000000007 | 0.0530303 | 0.008032128000000001 | 0.025171620000000002 | 0.062314540000000015 |
| 0.44 | 0.02631579 | 0.07883818 | 0.02839757 | 0.01233046 | 0.052966100000000016 | 0.03225806 | 0.08585858 | 0.0040160640000000015 | 0.009153318 | 0.03560831000000001 |
| 0.46 | 0.03289474 | 0.07883818 | 0.02636917 | 0.01479655 | 0.03389831 | 0.02016128999999999 | 0.07323232 | 0.0 | 0.004576659000000004 | 0.03560831000000001 |
| 0.48000000000000009 | 0.0065789470000000025 | 0.03319502 | 0.018255580000000007 | 0.009864365000000007 | 0.027542370000000014 | 0.018145160000000007 | 0.08585858 | 0.0 | 0.004576659000000004 | 0.017804160000000003 |
| 0.5 | 0.0 | 0.03319502 | 0.006085193000000003 | 0.003699137000000001 | 0.02118644 | 0.01209677 | 0.04292929 | 0.0 | 0.0022883290000000017 | 0.005934718000000002 |
| 0.52 | 0.0 | 0.04564314999999997 | 0.006085193000000003 | 0.0 | 0.01483051 | 0.004032258000000002 | 0.03535353 | 0.0040160640000000015 | 0.0 | 0.008902078 |
| 0.54 | 0.0 | 0.029045640000000008 | 0.0 | 0.0 | 0.01059322 | 0.01008064 | 0.05555555999999998 | 0.0 | 0.004576659000000004 | 0.008902078 |
| 0.56000000000000005 | 0.0 | 0.016597510000000003 | 0.004056795000000002 | 0.0 | 0.004237288000000002 | 0.002016129 | 0.05555555999999998 | 0.0 | 0.0 | 0.008902078 |
| 0.58000000000000007 | 0.0 | 0.004149378000000001 | 0.0 | 0.0 | 0.004237288000000002 | 0.002016129 | 0.06060606 | 0.0 | 0.0 | 0.0 |
| 0.6000000000000002 | 0.0 | 0.008298756000000003 | 0.0 | 0.0 | 0.006355931999999998 | 0.002016129 | 0.015151520000000005 | 0.0 | 0.0 | 0.0 |
| 0.62000000000000022 | 0.0 | 0.008298756000000003 | 0.0 | 0.0 | 0.004237288000000002 | 0.0 | 0.01010101 | 0.0 | 0.0 | 0.002967359000000001 |
| 0.64000000000000024 | 0.0 | 0.004149378000000001 | 0.0 | 0.0 | 0.004237288000000002 | 0.0 | 0.005050505 | 0.0 | 0.0 | 0.005934718000000002 |
| 0.66000000000000025 | 0.0 | 0.004149378000000001 | 0.0 | 0.0 | 0.0 | 0.0 | 0.01262626 | 0.0 | 0.0 | 0.0 |
| 0.68 | 0.0 | 0.008298756000000003 | 0.0 | 0.0 | 0.0 | 0.0 | 0.005050505 | 0.0 | 0.0 | 0.0 |
| 0.70000000000000018 | 0.0 | 0.0 | 0.0 | 0.0 | 0.0 | 0.0 | 0.002525253 | 0.0 | 0.0 | 0.0 |
### Chart: glr-1 A1
| Category | glr-1 A1 No.1 | glr-1 A1 No.2 | glr-1 A1 No.3 | glr-1 A1 No.4 | glr-1 A1 No.5 | glr-1 A1 No.6 | glr-1 A1 No.7 | glr-1 A1 No.8 | glr-1 A1 No.9 | glr-1 A1 No.10 | glr-1 A1 No.11 | glr-1 A1 No.12 | glr-1 A1 No.13 | glr-1 A1 No.14 | glr-1 A1 No.15 | glr-1 A1 No.16 | glr-1 A1 No.17 | glr-1 A1 No.18 | glr-1 A1 No.19 | glr-1 A1 No.20 |
|---|---|---|---|---|---|---|---|---|---|---|---|---|---|---|---|---|---|---|---|---|
| 0 | None | None | None | None | None | None | None | None | None | None | None | None | None | None | None | None | None | None | None | None |
| 2.0000000000000007E-2 | 0.0 | 0.0 | 0.0 | 0.0 | 0.0 | 0.0 | 0.0 | 0.0 | 0.0 | 0.004926108000000002 | 0.0 | 0.0 | 0.004651163000000002 | 0.0 | 0.0 | 0.0 | 0.0 | 0.005555556 | 0.005494506 | 0.0 |
| 4.0000000000000015E-2 | 0.0 | 0.0 | 0.006849315 | 0.0 | 0.0 | 0.0 | 0.0 | 0.0 | 0.0 | 0.0 | 0.0 | 0.0 | 0.0 | 0.0 | 0.0 | 0.0 | 0.0 | 0.0 | 0.005494506 | 0.004901961000000002 |
| 6.0000000000000019E-2 | 0.0 | 0.0 | 0.0 | 0.0 | 0.0 | 0.0 | 0.0 | 0.0 | 0.0 | 0.004926108000000002 | 0.0 | 0.0 | 0.0 | 0.0 | 0.0 | 0.0 | 0.0 | 0.0 | 0.005494506 | 0.0 |
| 8.0000000000000029E-2 | 0.0 | 0.0 | 0.0 | 0.0 | 0.0 | 0.0 | 0.0 | 0.0 | 0.0 | 0.0 | 0.0 | 0.0 | 0.0 | 0.0 | 0.0 | 0.0 | 0.0 | 0.0 | 0.005494506 | 0.0 |
| 0.1 | 0.0 | 0.0 | 0.0 | 0.0 | 0.0 | 0.0 | 0.0 | 0.0 | 0.0 | 0.0 | 0.0 | 0.0 | 0.0 | 0.0 | 0.0 | 0.0 | 0.0 | 0.0 | 0.01098901 | 0.0 |
| 0.12000000000000002 | 0.0 | 0.0 | 0.0 | 0.0 | 0.0 | 0.0065789470000000025 | 0.0 | 0.0 | 0.0 | 0.0 | 0.0 | 0.0 | 0.0 | 0.0 | 0.0 | 0.0 | 0.0 | 0.0 | 0.0 | 0.0 |
| 0.14000000000000001 | 0.0 | 0.0 | 0.006849315 | 0.0 | 0.0 | 0.0 | 0.0065789470000000025 | 0.0 | 0.0 | 0.004926108000000002 | 0.0 | 0.005747126000000002 | 0.0 | 0.0 | 0.0 | 0.0 | 0.0 | 0.0 | 0.0 | 0.0 |
| 0.16 | 0.0 | 0.0 | 0.0 | 0.0 | 0.008064516000000004 | 0.0 | 0.0 | 0.0 | 0.0 | 0.0 | 0.0 | 0.005747126000000002 | 0.0 | 0.0 | 0.0 | 0.0 | 0.0 | 0.0 | 0.0 | 0.0 |
| 0.18000000000000005 | 0.0 | 0.0 | 0.006849315 | 0.013422820000000007 | 0.008064516000000004 | 0.01315789 | 0.01315789 | 0.0 | 0.01204819 | 0.00985221700000001 | 0.0 | 0.005747126000000002 | 0.004651163000000002 | 0.0 | 0.0 | 0.0 | 0.0 | 0.005555556 | 0.005494506 | 0.0 |
| 0.2 | 0.005291005000000002 | 0.005347594 | 0.006849315 | 0.04026844999999998 | 0.016129030000000003 | 0.019736840000000005 | 0.01315789 | 0.015228430000000001 | 0.018072289999999998 | 0.01970443000000001 | 0.005291005000000002 | 0.0 | 0.004651163000000002 | 0.0052083330000000025 | 0.0 | 0.0 | 0.0 | 0.005555556 | 0.005494506 | 0.0 |
| 0.22 | 0.04761905 | 0.03208556 | 0.020547950000000002 | 0.026845640000000014 | 0.016129030000000003 | 0.03289474 | 0.03289474 | 0.035533 | 0.03614458 | 0.04926108 | 0.02116401999999999 | 0.005747126000000002 | 0.0 | 0.0 | 0.006410256000000002 | 0.0 | 0.0 | 0.005555556 | 0.01098901 | 0.0 |
| 0.24000000000000005 | 0.04761905 | 0.026737970000000017 | 0.04794521 | 0.04026844999999998 | 0.024193549999999998 | 0.07894737 | 0.019736840000000005 | 0.07106599 | 0.03614458 | 0.09359606000000008 | 0.026455030000000008 | 0.022988510000000007 | 0.0 | 0.0 | 0.006410256000000002 | 0.02857142999999999 | 0.0 | 0.005555556 | 0.01098901 | 0.009803922000000003 |
| 0.26 | 0.052910050000000014 | 0.08556150000000007 | 0.08219178 | 0.10067110000000003 | 0.05645161 | 0.09210526000000001 | 0.09210526000000001 | 0.1319797 | 0.126506 | 0.1133005 | 0.02116401999999999 | 0.051724139999999995 | 0.004651163000000002 | 0.010416669999999998 | 0.019230770000000008 | 0.0 | 0.01507538 | 0.03888889 | 0.03296703 | 0.009803922000000003 |
| 0.28000000000000008 | 0.08465608000000006 | 0.1336898 | 0.15753420000000007 | 0.15436240000000007 | 0.1290323 | 0.15131580000000006 | 0.11842110000000003 | 0.10659900000000003 | 0.16867469999999996 | 0.12315270000000003 | 0.05820105999999998 | 0.06896552 | 0.009302326000000007 | 0.04166667000000002 | 0.019230770000000008 | 0.08571429000000003 | 0.035175880000000014 | 0.06111110999999999 | 0.0934066 | 0.01960784 |
| 0.3000000000000001 | 0.1746032 | 0.1283422 | 0.15753420000000007 | 0.147651 | 0.1693548 | 0.15131580000000006 | 0.1644737 | 0.15228430000000007 | 0.1746988 | 0.1625616 | 0.04761905 | 0.1436782 | 0.06511628000000003 | 0.0625 | 0.05128205 | 0.1428571 | 0.030150749999999997 | 0.05 | 0.1208791 | 0.03921569 |
| 0.32000000000000012 | 0.1269841 | 0.15508020000000006 | 0.2123288 | 0.10738259999999998 | 0.1290323 | 0.2039474000000001 | 0.19736840000000005 | 0.142132 | 0.126506 | 0.11822660000000006 | 0.15873020000000007 | 0.16091950000000005 | 0.05581395000000003 | 0.10416670000000006 | 0.05128205 | 0.08571429000000003 | 0.1155779 | 0.1666667 | 0.10439560000000005 | 0.09803922000000002 |
| 0.34 | 0.15343910000000008 | 0.1497326 | 0.10273970000000003 | 0.14093960000000005 | 0.15322580000000005 | 0.1118421 | 0.131579 | 0.07614213 | 0.126506 | 0.1133005 | 0.11640209999999998 | 0.1666667 | 0.09767442000000004 | 0.1666667 | 0.1282051 | 0.1714286 | 0.12562809999999994 | 0.1722222 | 0.1373626 | 0.10784310000000003 |
| 0.3600000000000001 | 0.11640209999999998 | 0.1016043 | 0.08219178 | 0.09395973 | 0.10483870000000002 | 0.05263158 | 0.07894737 | 0.08629441 | 0.07831325000000003 | 0.07389162000000003 | 0.1269841 | 0.1149425 | 0.07906977 | 0.1666667 | 0.10256410000000005 | 0.1714286 | 0.09045226 | 0.14444450000000006 | 0.08791209 | 0.127451 |
| 0.38000000000000012 | 0.0952381 | 0.07486631000000005 | 0.05479452000000003 | 0.053691280000000015 | 0.12096770000000003 | 0.03947368000000001 | 0.04605263000000001 | 0.08121827000000001 | 0.05421687000000002 | 0.06896552 | 0.06878307000000003 | 0.0862069 | 0.1255814 | 0.18229170000000006 | 0.10897440000000003 | 0.08571429000000003 | 0.12060300000000003 | 0.1 | 0.1208791 | 0.127451 |
| 0.4 | 0.03703704000000002 | 0.06417112 | 0.04109589 | 0.03355705 | 0.024193549999999998 | 0.03289474 | 0.05263158 | 0.05076142000000003 | 0.02409638 | 0.02463054 | 0.07936508000000005 | 0.0862069 | 0.12093020000000003 | 0.09375000000000004 | 0.1410256 | 0.11428570000000003 | 0.10050249999999998 | 0.1 | 0.06593407 | 0.15686280000000005 |
| 0.4200000000000001 | 0.042328040000000004 | 0.016042780000000006 | 0.0 | 0.026845640000000014 | 0.03225806 | 0.0065789470000000025 | 0.03289474 | 0.02538071 | 0.01204819 | 0.00985221700000001 | 0.06349207000000003 | 0.03448276 | 0.1255814 | 0.078125 | 0.08333334000000003 | 0.08571429000000003 | 0.15577890000000005 | 0.06666667 | 0.08241757999999998 | 0.1176471 |
| 0.44 | 0.005291005000000002 | 0.016042780000000006 | 0.006849315 | 0.006711409000000002 | 0.008064516000000004 | 0.0 | 0.0 | 0.015228430000000001 | 0.0 | 0.004926108000000002 | 0.07407407000000003 | 0.02873563 | 0.1116279 | 0.05208333000000001 | 0.09615385000000004 | 0.0 | 0.04522613 | 0.03888889 | 0.03846154 | 0.09803922000000002 |
| 0.46 | 0.005291005000000002 | 0.005347594 | 0.006849315 | 0.006711409000000002 | 0.0 | 0.0 | 0.0 | 0.0 | 0.006024096 | 0.0 | 0.015873020000000005 | 0.0 | 0.04651163 | 0.020833330000000018 | 0.08974359000000003 | 0.0 | 0.05025125999999998 | 0.01666667 | 0.02197802 | 0.0245098 |
| 0.48000000000000009 | 0.0 | 0.0 | 0.0 | 0.006711409000000002 | 0.0 | 0.0065789470000000025 | 0.0 | 0.0 | 0.0 | 0.0 | 0.03703704000000002 | 0.005747126000000002 | 0.06046512 | 0.010416669999999998 | 0.03846154 | 0.0 | 0.0201005 | 0.005555556 | 0.01648352000000001 | 0.014705880000000001 |
| 0.5 | 0.005291005000000002 | 0.0 | 0.0 | 0.0 | 0.0 | 0.0 | 0.0 | 0.005076142000000002 | 0.0 | 0.0 | 0.02116401999999999 | 0.005747126000000002 | 0.05116279000000002 | 0.0 | 0.025641030000000006 | 0.02857142999999999 | 0.030150749999999997 | 0.005555556 | 0.005494506 | 0.02941176 |
| 0.52 | 0.0 | 0.0 | 0.0 | 0.0 | 0.0 | 0.0 | 0.0 | 0.0 | 0.0 | 0.0 | 0.026455030000000008 | 0.0 | 0.01395349 | 0.0052083330000000025 | 0.019230770000000008 | 0.0 | 0.01005025 | 0.005555556 | 0.0 | 0.004901961000000002 |
| 0.54 | 0.0 | 0.0 | 0.0 | 0.0 | 0.0 | 0.0 | 0.0 | 0.0 | 0.0 | 0.0 | 0.015873020000000005 | 0.0 | 0.009302326000000007 | 0.0 | 0.006410256000000002 | 0.0 | 0.02512562999999999 | 0.0 | 0.0 | 0.004901961000000002 |
| 0.56000000000000005 | 0.0 | 0.0 | 0.0 | 0.0 | 0.0 | 0.0 | 0.0 | 0.0 | 0.0 | 0.0 | 0.015873020000000005 | 0.0 | 0.0 | 0.0 | 0.006410256000000002 | 0.0 | 0.01005025 | 0.0 | 0.0 | 0.004901961000000002 |
| 0.58000000000000007 | 0.0 | 0.0 | 0.0 | 0.0 | 0.0 | 0.0 | 0.0 | 0.0 | 0.0 | 0.0 | 0.0 | 0.0 | 0.004651163000000002 | 0.0 | 0.0 | 0.0 | 0.005025126 | 0.0 | 0.005494506 | 0.0 |
| 0.6000000000000002 | 0.0 | 0.0 | 0.0 | 0.0 | 0.0 | 0.0 | 0.0 | 0.005076142000000002 | 0.0 | 0.0 | 0.0 | 0.0 | 0.004651163000000002 | 0.0 | 0.0 | 0.0 | 0.01005025 | 0.0 | 0.0 | 0.0 |
| 0.62000000000000022 | 0.0 | 0.0 | 0.0 | 0.0 | 0.0 | 0.0 | 0.0 | 0.0 | 0.0 | 0.0 | 0.0 | 0.0 | 0.0 | 0.0 | 0.0 | 0.0 | 0.0 | 0.0 | 0.0 | 0.0 |
| 0.64000000000000024 | 0.0 | 0.0 | 0.0 | 0.0 | 0.0 | 0.0 | 0.0 | 0.0 | 0.0 | 0.0 | 0.0 | 0.0 | 0.0 | 0.0 | 0.0 | 0.0 | 0.0 | 0.0 | 0.0 | 0.0 |
| 0.66000000000000025 | 0.0 | 0.0 | 0.0 | 0.0 | 0.0 | 0.0 | 0.0 | 0.0 | 0.0 | 0.0 | 0.0 | 0.0 | 0.0 | 0.0 | 0.0 | 0.0 | 0.005025126 | 0.0 | 0.0 | 0.0 |
| 0.68 | 0.0 | 0.0 | 0.0 | 0.0 | 0.0 | 0.0 | 0.0 | 0.0 | 0.0 | 0.0 | 0.0 | 0.0 | 0.0 | 0.0 | 0.0 | 0.0 | 0.0 | 0.0 | 0.0 | 0.0 |
| 0.70000000000000018 | 0.0 | 0.0 | 0.0 | 0.0 | 0.0 | 0.0 | 0.0 | 0.0 | 0.0 | 0.0 | 0.0 | 0.0 | 0.0 | 0.0 | 0.0 | 0.0 | 0.0 | 0.0 | 0.0 | 0.0 |
### Chart: glr-1 A3
| Category | glr-1 A3 No.1 | glr-1 A3 No.2 | glr-1 A3 No.3 | glr-1 A3 No.4 | glr-1 A3 No.5 | glr-1 A3 No.6 | glr-1 A3 No.7 | glr-1 A3 No.8 | glr-1 A3 No.9 | glr-1 A3 No.10 | glr-1 A3 No.11 | glr-1 A3 No.12 | glr-1 A3 No.13 | glr-1 A3 No.14 | glr-1 A3 No.15 | glr-1 A3 No.16 | glr-1 A3 No.17 | glr-1 A3 No.18 | glr-1 A3 No.19 |
|---|---|---|---|---|---|---|---|---|---|---|---|---|---|---|---|---|---|---|---|
| 0 | None | None | None | None | None | None | None | None | None | None | None | None | None | None | None | None | None | None | None |
| 2.0000000000000007E-2 | 0.0 | 0.0 | 0.0 | 0.0 | 0.0 | 0.0 | 0.0 | 0.0072463770000000035 | 0.0 | 0.0 | 0.0 | 0.0 | 0.0 | 0.0 | 0.0 | 0.0 | 0.006493506000000002 | 0.0 | 0.0 |
| 4.0000000000000015E-2 | 0.0 | 0.0 | 0.0 | 0.0 | 0.0 | 0.0 | 0.0 | 0.0072463770000000035 | 0.0 | 0.0 | 0.0 | 0.0 | 0.0 | 0.0 | 0.0 | 0.0 | 0.0 | 0.0 | 0.0 |
| 6.0000000000000019E-2 | 0.0 | 0.0 | 0.0 | 0.0 | 0.0 | 0.0 | 0.0 | 0.0 | 0.0 | 0.0 | 0.0 | 0.0 | 0.0 | 0.0 | 0.0 | 0.0 | 0.006493506000000002 | 0.0 | 0.0 |
| 8.0000000000000029E-2 | 0.0 | 0.0 | 0.0 | 0.0 | 0.0 | 0.0 | 0.0 | 0.0 | 0.0 | 0.0 | 0.0 | 0.0 | 0.0 | 0.0 | 0.0 | 0.0 | 0.0 | 0.0 | 0.0 |
| 0.1 | 0.0 | 0.0 | 0.0 | 0.0 | 0.0 | 0.0 | 0.0 | 0.0 | 0.0 | 0.0 | 0.0 | 0.0 | 0.0 | 0.0 | 0.0 | 0.0 | 0.0 | 0.0 | 0.0 |
| 0.12000000000000002 | 0.0 | 0.0 | 0.0 | 0.0 | 0.0 | 0.0 | 0.0 | 0.0 | 0.0 | 0.0 | 0.0 | 0.0 | 0.0 | 0.0 | 0.0 | 0.0 | 0.0 | 0.0 | 0.0 |
| 0.14000000000000001 | 0.0 | 0.0 | 0.0 | 0.0 | 0.0 | 0.0 | 0.0 | 0.0 | 0.0 | 0.0 | 0.0 | 0.0 | 0.0 | 0.0 | 0.0 | 0.004784689000000002 | 0.0 | 0.0 | 0.0 |
| 0.16 | 0.0 | 0.0 | 0.0 | 0.0 | 0.0 | 0.0 | 0.0 | 0.0 | 0.0 | 0.0 | 0.0 | 0.0 | 0.0 | 0.0 | 0.0 | 0.0 | 0.0 | 0.0 | 0.0 |
| 0.18000000000000005 | 0.0 | 0.0 | 0.0 | 0.0 | 0.0 | 0.0 | 0.0 | 0.0 | 0.0 | 0.0 | 0.008695652000000005 | 0.0 | 0.0 | 0.0 | 0.0 | 0.0 | 0.0 | 0.0 | 0.0 |
| 0.2 | 0.0 | 0.0 | 0.0 | 0.0 | 0.0 | 0.0 | 0.0 | 0.0 | 0.0 | 0.0 | 0.004347825999999998 | 0.0 | 0.0 | 0.0 | 0.007462686000000002 | 0.004784689000000002 | 0.0 | 0.0 | 0.0 |
| 0.22 | 0.008196721 | 0.0 | 0.0 | 0.0 | 0.0 | 0.0 | 0.0 | 0.0 | 0.0 | 0.0 | 0.008695652000000005 | 0.0 | 0.0 | 0.0 | 0.0 | 0.0 | 0.0 | 0.0 | 0.0 |
| 0.24000000000000005 | 0.016393440000000002 | 0.0 | 0.0 | 0.0 | 0.0 | 0.0 | 0.0 | 0.0 | 0.0 | 0.0 | 0.043478259999999984 | 0.0 | 0.0 | 0.0 | 0.0 | 0.004784689000000002 | 0.01298701 | 0.0 | 0.0 |
| 0.26 | 0.016393440000000002 | 0.02068966 | 0.0 | 0.0 | 0.006493506000000002 | 0.012820510000000004 | 0.02158273 | 0.0 | 0.0 | 0.02325581 | 0.05652173999999999 | 0.012578619999999997 | 0.016759780000000005 | 0.01162791 | 0.03731343 | 0.06698564 | 0.02597403 | 0.00913242 | 0.0 |
| 0.28000000000000008 | 0.05737705 | 0.03448276 | 0.07462686 | 0.01503759 | 0.01948052000000001 | 0.05128205 | 0.06474820000000003 | 0.0072463770000000035 | 0.016260160000000006 | 0.0 | 0.12173910000000003 | 0.006289308000000004 | 0.08379889000000007 | 0.01162791 | 0.09701493000000004 | 0.1004785 | 0.07142857 | 0.01369863 | 0.011834320000000004 |
| 0.3000000000000001 | 0.1229508 | 0.13793100000000005 | 0.1791045 | 0.02255639 | 0.04545455 | 0.13461540000000005 | 0.12949640000000007 | 0.07971015 | 0.0406504 | 0.031007750000000008 | 0.17826090000000006 | 0.037735850000000015 | 0.1731844 | 0.08139535000000005 | 0.07462686 | 0.21531100000000006 | 0.1363636 | 0.022831050000000012 | 0.04733728000000003 |
| 0.32000000000000012 | 0.1721312 | 0.2275862 | 0.1492537 | 0.030075190000000012 | 0.1168831 | 0.1923077 | 0.2086331 | 0.115942 | 0.06504065000000002 | 0.15503880000000006 | 0.1913043 | 0.1257862 | 0.21229050000000005 | 0.10465120000000003 | 0.08208955000000001 | 0.19617229999999997 | 0.1948052 | 0.08675799000000003 | 0.07692308000000002 |
| 0.34 | 0.1721312 | 0.22068969999999993 | 0.16417909999999997 | 0.11278199999999998 | 0.1623377 | 0.15384620000000007 | 0.1798561 | 0.10869560000000006 | 0.10569110000000004 | 0.15503880000000006 | 0.1521739 | 0.1257862 | 0.2290503 | 0.11627910000000002 | 0.1343284 | 0.1435407 | 0.1428571 | 0.12328770000000003 | 0.18934910000000008 |
| 0.3600000000000001 | 0.22950820000000005 | 0.13793100000000005 | 0.1791045 | 0.15789470000000005 | 0.1948052 | 0.10897440000000003 | 0.1151079 | 0.18840580000000007 | 0.16260159999999996 | 0.21705430000000006 | 0.09565217000000005 | 0.15094340000000012 | 0.09497207 | 0.18604650000000006 | 0.08955224000000006 | 0.09090909000000003 | 0.1623377 | 0.1324201 | 0.2071006 |
| 0.38000000000000012 | 0.15573770000000006 | 0.11724140000000002 | 0.1343284 | 0.16541350000000005 | 0.1168831 | 0.1153846 | 0.08633094000000002 | 0.1521739 | 0.18699190000000007 | 0.11627910000000002 | 0.043478259999999984 | 0.2012579 | 0.07821229000000003 | 0.08139535000000005 | 0.1492537 | 0.07655502 | 0.12337660000000003 | 0.12785389999999994 | 0.15384620000000007 |
| 0.4 | 0.02459016 | 0.05517241 | 0.05970149000000004 | 0.1203008 | 0.1363636 | 0.05128205 | 0.1007194 | 0.10869560000000006 | 0.11382109999999998 | 0.11627910000000002 | 0.03478261 | 0.13207549999999998 | 0.03910615 | 0.122093 | 0.1044776 | 0.038277510000000015 | 0.07792208000000003 | 0.1324201 | 0.07692308000000002 |
| 0.4200000000000001 | 0.016393440000000002 | 0.02068966 | 0.02985075 | 0.09774436000000006 | 0.058441559999999976 | 0.03846154 | 0.05035971 | 0.06521739000000001 | 0.10569110000000004 | 0.10077520000000004 | 0.02173913 | 0.11949690000000003 | 0.027932960000000014 | 0.09302326000000004 | 0.08208955000000001 | 0.023923449999999992 | 0.006493506000000002 | 0.09132420000000002 | 0.07692308000000002 |
| 0.44 | 0.0 | 0.02068966 | 0.0 | 0.06766918 | 0.02597403 | 0.032051280000000015 | 0.02877698 | 0.05797102 | 0.056910570000000014 | 0.05426357 | 0.0173913 | 0.06918239000000002 | 0.011173180000000001 | 0.058139539999999997 | 0.044776120000000016 | 0.009569378000000003 | 0.0 | 0.06392694 | 0.04733728000000003 |
| 0.46 | 0.008196721 | 0.006896552000000002 | 0.0 | 0.06015038 | 0.03246753 | 0.03846154 | 0.007194245000000002 | 0.01449275 | 0.016260160000000006 | 0.0077519380000000025 | 0.004347825999999998 | 0.006289308000000004 | 0.011173180000000001 | 0.02325581 | 0.02985075 | 0.004784689000000002 | 0.01948052000000001 | 0.08675799000000003 | 0.03550296 |
| 0.48000000000000009 | 0.0 | 0.0 | 0.014925370000000004 | 0.06015038 | 0.04545455 | 0.025641030000000006 | 0.0 | 0.02898551 | 0.02439024 | 0.0077519380000000025 | 0.008695652000000005 | 0.006289308000000004 | 0.011173180000000001 | 0.034883720000000014 | 0.03731343 | 0.004784689000000002 | 0.006493506000000002 | 0.03196347 | 0.01775148 |
| 0.5 | 0.0 | 0.0 | 0.0 | 0.04511278000000002 | 0.02597403 | 0.032051280000000015 | 0.0 | 0.0072463770000000035 | 0.008130081 | 0.0077519380000000025 | 0.0 | 0.0 | 0.0 | 0.017441860000000003 | 0.0 | 0.004784689000000002 | 0.0 | 0.02739726000000001 | 0.011834320000000004 |
| 0.52 | 0.0 | 0.0 | 0.014925370000000004 | 0.02255639 | 0.0 | 0.0 | 0.007194245000000002 | 0.01449275 | 0.008130081 | 0.0 | 0.0 | 0.006289308000000004 | 0.005586592000000002 | 0.01162791 | 0.0 | 0.004784689000000002 | 0.0 | 0.01826484 | 0.0295858 |
| 0.54 | 0.0 | 0.0 | 0.0 | 0.01503759 | 0.0 | 0.006410256000000002 | 0.0 | 0.0 | 0.016260160000000006 | 0.0 | 0.004347825999999998 | 0.0 | 0.0 | 0.01162791 | 0.007462686000000002 | 0.0 | 0.0 | 0.01369863 | 0.005917160000000002 |
| 0.56000000000000005 | 0.0 | 0.0 | 0.0 | 0.0 | 0.0 | 0.0 | 0.0 | 0.0072463770000000035 | 0.02439024 | 0.0 | 0.0 | 0.0 | 0.0 | 0.0 | 0.007462686000000002 | 0.004784689000000002 | 0.006493506000000002 | 0.00913242 | 0.0 |
| 0.58000000000000007 | 0.0 | 0.0 | 0.0 | 0.0 | 0.006493506000000002 | 0.0 | 0.0 | 0.0072463770000000035 | 0.03252032 | 0.0077519380000000025 | 0.0 | 0.0 | 0.005586592000000002 | 0.01162791 | 0.014925370000000004 | 0.0 | 0.0 | 0.0 | 0.005917160000000002 |
| 0.6000000000000002 | 0.0 | 0.0 | 0.0 | 0.007518797000000002 | 0.0 | 0.006410256000000002 | 0.0 | 0.0 | 0.0 | 0.0 | 0.0 | 0.0 | 0.0 | 0.01162791 | 0.0 | 0.0 | 0.0 | 0.0 | 0.0 |
| 0.62000000000000022 | 0.0 | 0.0 | 0.0 | 0.0 | 0.0 | 0.0 | 0.0 | 0.0 | 0.008130081 | 0.0 | 0.004347825999999998 | 0.0 | 0.0 | 0.0 | 0.0 | 0.0 | 0.0 | 0.0 | 0.0 |
| 0.64000000000000024 | 0.0 | 0.0 | 0.0 | 0.0 | 0.0 | 0.0 | 0.0 | 0.0 | 0.008130081 | 0.0 | 0.0 | 0.0 | 0.0 | 0.01162791 | 0.0 | 0.0 | 0.0 | 0.0 | 0.0 |
| 0.66000000000000025 | 0.0 | 0.0 | 0.0 | 0.0 | 0.0 | 0.0 | 0.0 | 0.0072463770000000035 | 0.0 | 0.0 | 0.0 | 0.0 | 0.0 | 0.0 | 0.0 | 0.0 | 0.0 | 0.0 | 0.005917160000000002 |
| 0.68 | 0.0 | 0.0 | 0.0 | 0.0 | 0.006493506000000002 | 0.0 | 0.0 | 0.0 | 0.0 | 0.0 | 0.0 | 0.0 | 0.0 | 0.0 | 0.0 | 0.0 | 0.0 | 0.0 | 0.0 |
| 0.70000000000000018 | 0.0 | 0.0 | 0.0 | 0.0 | 0.0 | 0.0 | 0.0 | 0.0072463770000000035 | 0.0 | 0.0 | 0.0 | 0.0 | 0.0 | 0.0 | 0.0 | 0.0 | 0.0 | 0.00456621 | 0.0 |
### Chart: glr-1 A5
| Category | glr-1 A5 No.1 | glr-1 A5 No.2 | glr-1 A5 No.3 | glr-1 A5 No.4 | glr-1 A5 No.5 | glr-1 A5 No.6 | glr-1 A5 No.7 | glr-1 A5 No.8 | glr-1 A5 No.9 | glr-1 A5 No.10 | glr-1 A5 No.11 | glr-1 A5 No.12 | glr-1 A5 No.13 | glr-1 A5 No.14 | glr-1 A5 No.15 | glr-1 A5 No.16 | glr-1 A5 No.17 | glr-1 A5 No.18 | glr-1 A5 No.19 |
|---|---|---|---|---|---|---|---|---|---|---|---|---|---|---|---|---|---|---|---|
| 0 | None | None | None | None | None | None | None | None | None | None | None | None | None | None | None | None | None | None | None |
| 2.0000000000000007E-2 | 0.0 | 0.0 | 0.0 | 0.0 | 0.01904762 | 0.02040816 | 0.0 | 0.008000000000000005 | 0.006211180000000002 | 0.007194245000000002 | 0.0 | 0.01219512 | 0.0 | 0.0 | 0.0 | 0.0 | 0.0 | 0.03529412 | 0.0 |
| 4.0000000000000015E-2 | 0.027027030000000007 | 0.0 | 0.0 | 0.0 | 0.00952381 | 0.0 | 0.0 | 0.0 | 0.006211180000000002 | 0.0 | 0.0 | 0.01219512 | 0.0 | 0.01204819 | 0.0 | 0.0 | 0.0 | 0.03529412 | 0.0 |
| 6.0000000000000019E-2 | 0.0 | 0.0 | 0.0 | 0.0 | 0.01904762 | 0.0 | 0.0 | 0.016000000000000007 | 0.0 | 0.0 | 0.0 | 0.0 | 0.0 | 0.0 | 0.0 | 0.0 | 0.0 | 0.011764710000000001 | 0.0 |
| 8.0000000000000029E-2 | 0.0 | 0.008620690000000004 | 0.0 | 0.0 | 0.0 | 0.0 | 0.0 | 0.0 | 0.0 | 0.007194245000000002 | 0.0 | 0.0 | 0.0 | 0.0 | 0.0 | 0.0 | 0.0 | 0.0 | 0.0 |
| 0.1 | 0.0 | 0.0 | 0.0 | 0.0 | 0.0 | 0.0 | 0.0 | 0.0 | 0.0 | 0.0 | 0.0 | 0.0 | 0.0 | 0.0 | 0.0 | 0.0 | 0.0 | 0.0 | 0.0 |
| 0.12000000000000002 | 0.027027030000000007 | 0.0 | 0.0 | 0.0 | 0.0 | 0.0 | 0.0 | 0.0 | 0.0 | 0.0 | 0.0 | 0.0 | 0.0 | 0.0 | 0.0 | 0.0 | 0.0 | 0.0 | 0.0 |
| 0.14000000000000001 | 0.0 | 0.008620690000000004 | 0.0 | 0.0 | 0.00952381 | 0.0 | 0.0 | 0.0 | 0.006211180000000002 | 0.0 | 0.0 | 0.0 | 0.0 | 0.0 | 0.0 | 0.0 | 0.0 | 0.0 | 0.0 |
| 0.16 | 0.0 | 0.0 | 0.0 | 0.0 | 0.0 | 0.01020408 | 0.0 | 0.0 | 0.0 | 0.007194245000000002 | 0.0 | 0.0 | 0.0 | 0.0 | 0.0 | 0.0 | 0.0 | 0.0 | 0.0 |
| 0.18000000000000005 | 0.0 | 0.0 | 0.0 | 0.0 | 0.00952381 | 0.0 | 0.0 | 0.0 | 0.0 | 0.0 | 0.0 | 0.0 | 0.0 | 0.006024096 | 0.0 | 0.007407407000000002 | 0.0 | 0.0 | 0.0 |
| 0.2 | 0.0 | 0.01724138 | 0.0 | 0.0 | 0.0 | 0.01020408 | 0.0 | 0.008000000000000005 | 0.0 | 0.0 | 0.0 | 0.0 | 0.0 | 0.006024096 | 0.0 | 0.0 | 0.0 | 0.0 | 0.007462686000000002 |
| 0.22 | 0.0 | 0.01724138 | 0.0 | 0.0 | 0.02857142999999999 | 0.0 | 0.011363640000000001 | 0.0 | 0.006211180000000002 | 0.0 | 0.046874999999999986 | 0.0 | 0.0 | 0.0 | 0.0 | 0.01481481 | 0.0 | 0.011764710000000001 | 0.014925370000000004 |
| 0.24000000000000005 | 0.0 | 0.06034483 | 0.01785714 | 0.0 | 0.05714285999999998 | 0.01020408 | 0.011363640000000001 | 0.0 | 0.0 | 0.02877698 | 0.0625 | 0.03658536000000001 | 0.0 | 0.0 | 0.0 | 0.007407407000000002 | 0.0 | 0.0 | 0.0 |
| 0.26 | 0.0 | 0.04310345 | 0.03571429 | 0.012820510000000004 | 0.02857142999999999 | 0.0 | 0.022727270000000008 | 0.0 | 0.0 | 0.03597122000000001 | 0.125 | 0.048780490000000024 | 0.0 | 0.01204819 | 0.0 | 0.007407407000000002 | 0.006134969 | 0.0 | 0.007462686000000002 |
| 0.28000000000000008 | 0.0 | 0.09482758000000005 | 0.08928572000000001 | 0.0448718 | 0.10476190000000005 | 0.0 | 0.03409091 | 0.008000000000000005 | 0.01863354 | 0.06474820000000003 | 0.140625 | 0.15853660000000006 | 0.013793100000000004 | 0.03614458 | 0.0 | 0.03703704000000002 | 0.01840491 | 0.03529412 | 0.014925370000000004 |
| 0.3000000000000001 | 0.08108108 | 0.06034483 | 0.11309520000000005 | 0.03846154 | 0.11428570000000003 | 0.05102041 | 0.022727270000000008 | 0.048 | 0.04968944 | 0.0935251800000001 | 0.140625 | 0.19512189999999993 | 0.0 | 0.1024096 | 0.0 | 0.02962963 | 0.02453988 | 0.03529412 | 0.03731343 |
| 0.32000000000000012 | 0.08108108 | 0.1293103 | 0.11309520000000005 | 0.08974359000000003 | 0.11428570000000003 | 0.08163265 | 0.11363640000000003 | 0.06400000000000003 | 0.04968944 | 0.1366906 | 0.140625 | 0.06097561000000001 | 0.06206897000000003 | 0.08433735000000005 | 0.006211180000000002 | 0.03703704000000002 | 0.11656440000000003 | 0.023529409999999994 | 0.044776120000000016 |
| 0.34 | 0.10810810000000003 | 0.051724139999999995 | 0.15476190000000006 | 0.13461540000000005 | 0.10476190000000005 | 0.061224489999999986 | 0.125 | 0.096 | 0.05590062000000002 | 0.15827340000000006 | 0.10937500000000003 | 0.12195120000000002 | 0.1103448 | 0.09638554 | 0.02484472 | 0.08148148 | 0.18404910000000008 | 0.04705882 | 0.11940300000000002 |
| 0.3600000000000001 | 0.18918920000000006 | 0.0862069 | 0.1845238 | 0.1602564 | 0.0952381 | 0.11224489999999998 | 0.06818182 | 0.112 | 0.06832298000000003 | 0.10791370000000003 | 0.09375000000000004 | 0.13414630000000005 | 0.1310345 | 0.1204819 | 0.02484472 | 0.14814810000000006 | 0.1656442 | 0.14117649999999998 | 0.08955224000000006 |
| 0.38000000000000012 | 0.08108108 | 0.06896552 | 0.07142857 | 0.15384620000000007 | 0.08571429000000003 | 0.11224489999999998 | 0.1022727 | 0.048 | 0.09937888 | 0.05035971 | 0.0625 | 0.048780490000000024 | 0.06896552 | 0.07831325000000003 | 0.05590062000000002 | 0.2 | 0.18404910000000008 | 0.05882353 | 0.1044776 |
| 0.4 | 0.027027030000000007 | 0.03448276 | 0.05952381 | 0.12179490000000005 | 0.03809524 | 0.05102041 | 0.11363640000000003 | 0.08000000000000003 | 0.0621118 | 0.04316547 | 0.03125 | 0.03658536000000001 | 0.1103448 | 0.11445779999999997 | 0.136646 | 0.1333333 | 0.12269940000000003 | 0.1058824 | 0.11940300000000002 |
| 0.4200000000000001 | 0.10810810000000003 | 0.04310345 | 0.06547619 | 0.08333334000000003 | 0.04761905 | 0.05102041 | 0.05681818 | 0.10400000000000002 | 0.09937888 | 0.08633094000000002 | 0.046874999999999986 | 0.03658536000000001 | 0.12413790000000002 | 0.09638554 | 0.07453416 | 0.08148148 | 0.07975460000000002 | 0.05882353 | 0.1343284 |
| 0.44 | 0.054054060000000015 | 0.06896552 | 0.0297619 | 0.05769231000000001 | 0.02857142999999999 | 0.040816330000000026 | 0.03409091 | 0.072 | 0.0621118 | 0.01438849 | 0.0 | 0.06097561000000001 | 0.05517241 | 0.04819277000000002 | 0.10559010000000005 | 0.06666667 | 0.02453988 | 0.023529409999999994 | 0.07462686 |
| 0.46 | 0.027027030000000007 | 0.04310345 | 0.01785714 | 0.025641030000000006 | 0.03809524 | 0.08163265 | 0.03409091 | 0.032000000000000015 | 0.08695652000000004 | 0.04316547 | 0.0 | 0.01219512 | 0.05517241 | 0.07228914999999997 | 0.07453416 | 0.05925926 | 0.02453988 | 0.1176471 | 0.08208955000000001 |
| 0.48000000000000009 | 0.08108108 | 0.01724138 | 0.011904760000000004 | 0.019230770000000008 | 0.0 | 0.08163265 | 0.06818182 | 0.016000000000000007 | 0.05590062000000002 | 0.02877698 | 0.0 | 0.0 | 0.02068966 | 0.018072289999999998 | 0.10559010000000005 | 0.01481481 | 0.006134969 | 0.04705882 | 0.02985075 |
| 0.5 | 0.0 | 0.02586207 | 0.011904760000000004 | 0.012820510000000004 | 0.01904762 | 0.040816330000000026 | 0.06818182 | 0.048 | 0.043478259999999984 | 0.04316547 | 0.0 | 0.0 | 0.03448276 | 0.01204819 | 0.06832298000000003 | 0.03703704000000002 | 0.006134969 | 0.04705882 | 0.02985075 |
| 0.52 | 0.0 | 0.02586207 | 0.0 | 0.025641030000000006 | 0.00952381 | 0.05102041 | 0.04545455 | 0.056 | 0.05590062000000002 | 0.01438849 | 0.0 | 0.01219512 | 0.06896552 | 0.02409638 | 0.10559010000000005 | 0.01481481 | 0.012269939999999998 | 0.023529409999999994 | 0.02238806 |
| 0.54 | 0.0 | 0.01724138 | 0.011904760000000004 | 0.006410256000000002 | 0.0 | 0.03061225 | 0.011363640000000001 | 0.016000000000000007 | 0.04968944 | 0.007194245000000002 | 0.0 | 0.01219512 | 0.03448276 | 0.01204819 | 0.05590062000000002 | 0.02222222 | 0.0 | 0.0 | 0.0 |
| 0.56000000000000005 | 0.0 | 0.03448276 | 0.005952381 | 0.0 | 0.00952381 | 0.01020408 | 0.022727270000000008 | 0.040000000000000015 | 0.031055900000000015 | 0.0 | 0.0 | 0.0 | 0.02068966 | 0.01204819 | 0.031055900000000015 | 0.0 | 0.006134969 | 0.023529409999999994 | 0.02238806 |
| 0.58000000000000007 | 0.0 | 0.0 | 0.0 | 0.006410256000000002 | 0.0 | 0.03061225 | 0.011363640000000001 | 0.048 | 0.03726708 | 0.007194245000000002 | 0.0 | 0.0 | 0.0 | 0.006024096 | 0.006211180000000002 | 0.0 | 0.012269939999999998 | 0.03529412 | 0.007462686000000002 |
| 0.6000000000000002 | 0.027027030000000007 | 0.008620690000000004 | 0.0 | 0.0 | 0.00952381 | 0.02040816 | 0.0 | 0.016000000000000007 | 0.01863354 | 0.0 | 0.0 | 0.0 | 0.0 | 0.018072289999999998 | 0.03726708 | 0.0 | 0.0 | 0.011764710000000001 | 0.0 |
| 0.62000000000000022 | 0.027027030000000007 | 0.0 | 0.0 | 0.0 | 0.0 | 0.01020408 | 0.011363640000000001 | 0.008000000000000005 | 0.006211180000000002 | 0.01438849 | 0.0 | 0.0 | 0.027586210000000014 | 0.0 | 0.031055900000000015 | 0.0 | 0.0 | 0.011764710000000001 | 0.0 |
| 0.64000000000000024 | 0.0 | 0.008620690000000004 | 0.0 | 0.0 | 0.0 | 0.02040816 | 0.0 | 0.016000000000000007 | 0.006211180000000002 | 0.0 | 0.0 | 0.0 | 0.006896552000000002 | 0.006024096 | 0.02484472 | 0.0 | 0.0 | 0.023529409999999994 | 0.007462686000000002 |
| 0.66000000000000025 | 0.0 | 0.008620690000000004 | 0.0 | 0.0 | 0.0 | 0.01020408 | 0.0 | 0.024 | 0.0 | 0.0 | 0.0 | 0.0 | 0.006896552000000002 | 0.0 | 0.006211180000000002 | 0.0 | 0.0 | 0.023529409999999994 | 0.007462686000000002 |
| 0.68 | 0.0 | 0.0 | 0.0 | 0.006410256000000002 | 0.0 | 0.0 | 0.0 | 0.0 | 0.01242236 | 0.0 | 0.0 | 0.0 | 0.02068966 | 0.0 | 0.01242236 | 0.0 | 0.006134969 | 0.011764710000000001 | 0.0 |
| 0.70000000000000018 | 0.027027030000000007 | 0.0 | 0.005952381 | 0.0 | 0.0 | 0.0 | 0.0 | 0.0 | 0.006211180000000002 | 0.0 | 0.0 | 0.0 | 0.006896552000000002 | 0.0 | 0.0 | 0.0 | 0.0 | 0.0 | 0.007462686000000002 |
### Chart: nmr-1 A1
| Category | nmr-1 A1 No.1 | nmr-1 A1 No.2 | nmr-1 A1 No.3 | nmr-1 A1 No.4 | nmr-1 A1 No.5 | nmr-1 A1 No.6 | nmr-1 A1 No.7 | nmr-1 A1 No.8 | nmr-1 A1 No.9 | nmr-1 A1 No.10 | nmr-1 A1 No.11 | nmr-1 A1 No.12 | nmr-1 A1 No.13 | nmr-1 A1 No.14 | nmr-1 A1 No.15 | nmr-1 A1 No.16 | nmr-1 A1 No.17 | nmr-1 A1 No.18 | nmr-1 A1 No.19 |
|---|---|---|---|---|---|---|---|---|---|---|---|---|---|---|---|---|---|---|---|
| 0 | None | None | None | None | None | None | None | None | None | None | None | None | None | None | None | None | None | None | None |
| 2.0000000000000007E-2 | 0.0 | 0.005649718 | 0.0 | 0.0 | 0.0 | 0.0 | 0.0 | 0.0 | 0.0 | 0.0 | 0.0 | 0.0 | 0.0 | 0.0 | 0.0 | 0.01219512 | 0.0 | 0.0 | 0.0 |
| 4.0000000000000015E-2 | 0.0 | 0.0 | 0.0 | 0.0 | 0.0 | 0.0 | 0.0 | 0.0 | 0.0 | 0.0 | 0.0 | 0.0 | 0.0 | 0.005464481 | 0.0 | 0.01219512 | 0.0057803470000000025 | 0.0 | 0.0 |
| 6.0000000000000019E-2 | 0.010582010000000001 | 0.0 | 0.0 | 0.0 | 0.0 | 0.0 | 0.0 | 0.0 | 0.0 | 0.0 | 0.0 | 0.0 | 0.0 | 0.0 | 0.0 | 0.0 | 0.0 | 0.0 | 0.0 |
| 8.0000000000000029E-2 | 0.0 | 0.005649718 | 0.0 | 0.0 | 0.0 | 0.0 | 0.0 | 0.0 | 0.0 | 0.0 | 0.0 | 0.0 | 0.0 | 0.005464481 | 0.005319149000000001 | 0.03658536000000001 | 0.0057803470000000025 | 0.0 | 0.0 |
| 0.1 | 0.0 | 0.0 | 0.0 | 0.0 | 0.0 | 0.004424779000000002 | 0.0 | 0.0 | 0.0 | 0.0 | 0.0 | 0.0 | 0.0 | 0.0 | 0.0 | 0.0 | 0.0 | 0.0 | 0.0 |
| 0.12000000000000002 | 0.0 | 0.0 | 0.0 | 0.0 | 0.0 | 0.0 | 0.0 | 0.0 | 0.0 | 0.003891051000000001 | 0.005847953 | 0.0 | 0.0 | 0.005464481 | 0.0 | 0.01219512 | 0.0 | 0.0 | 0.009950249000000006 |
| 0.14000000000000001 | 0.005291005000000002 | 0.0 | 0.005494506 | 0.01438849 | 0.0 | 0.004424779000000002 | 0.0 | 0.0 | 0.0 | 0.0 | 0.0 | 0.0 | 0.0 | 0.0 | 0.0 | 0.0 | 0.0 | 0.0 | 0.0 |
| 0.16 | 0.0 | 0.0 | 0.0 | 0.007194245000000002 | 0.0 | 0.004424779000000002 | 0.0 | 0.0 | 0.0 | 0.0 | 0.0 | 0.0 | 0.0 | 0.005464481 | 0.0 | 0.01219512 | 0.0 | 0.004484305000000002 | 0.004975124000000002 |
| 0.18000000000000005 | 0.010582010000000001 | 0.005649718 | 0.0 | 0.0 | 0.004672897 | 0.0 | 0.009090909000000003 | 0.00617284 | 0.0 | 0.007782101000000002 | 0.0 | 0.0 | 0.005434783000000002 | 0.0 | 0.0 | 0.0 | 0.0 | 0.004484305000000002 | 0.004975124000000002 |
| 0.2 | 0.010582010000000001 | 0.01129944 | 0.01648352000000001 | 0.007194245000000002 | 0.004672897 | 0.008849557000000006 | 0.022727270000000008 | 0.018518520000000007 | 0.01242236 | 0.0233463 | 0.005847953 | 0.0072992700000000035 | 0.010869570000000007 | 0.01092896 | 0.021276600000000007 | 0.0 | 0.0 | 0.008968610000000005 | 0.009950249000000006 |
| 0.22 | 0.015873020000000005 | 0.02824859 | 0.03846154 | 0.02877698 | 0.028037380000000008 | 0.03539823 | 0.04545455 | 0.018518520000000007 | 0.02484472 | 0.050583660000000016 | 0.01754386 | 0.0072992700000000035 | 0.005434783000000002 | 0.049180330000000015 | 0.04255319 | 0.02439024 | 0.02890173 | 0.02690583 | 0.039800990000000015 |
| 0.24000000000000005 | 0.05820105999999998 | 0.07344633 | 0.03296703 | 0.06474820000000003 | 0.07476635000000002 | 0.061946899999999985 | 0.08636363999999999 | 0.08641974999999998 | 0.05590062000000002 | 0.1128405 | 0.005847953 | 0.021897810000000014 | 0.043478259999999984 | 0.07650273000000003 | 0.06382979 | 0.02439024 | 0.02890173 | 0.04932735 | 0.039800990000000015 |
| 0.26 | 0.1111111 | 0.07344633 | 0.04945055 | 0.07913669000000004 | 0.12149530000000003 | 0.07522124 | 0.1 | 0.191358 | 0.08695652000000004 | 0.15175100000000005 | 0.03508772000000001 | 0.08759124000000003 | 0.06521739000000001 | 0.12021859999999998 | 0.053191490000000015 | 0.06097561000000001 | 0.09248555 | 0.08071749000000003 | 0.10945270000000003 |
| 0.28000000000000008 | 0.15343910000000008 | 0.13559320000000005 | 0.15384620000000007 | 0.1798561 | 0.1635514 | 0.15486730000000007 | 0.15000000000000005 | 0.1975309 | 0.1304348 | 0.15953310000000007 | 0.05847953 | 0.12408760000000002 | 0.17391300000000007 | 0.1912568 | 0.1861702 | 0.08536585000000005 | 0.12138729999999998 | 0.1210762 | 0.16417909999999997 |
| 0.3000000000000001 | 0.1322751 | 0.18644070000000004 | 0.1318681 | 0.15827340000000006 | 0.1401869 | 0.1681416 | 0.15454550000000006 | 0.154321 | 0.1304348 | 0.17898830000000007 | 0.07017544 | 0.1970803 | 0.2065217 | 0.17486340000000006 | 0.1755319 | 0.07317073 | 0.1907514 | 0.22421520000000006 | 0.16417909999999997 |
| 0.32000000000000012 | 0.21164020000000006 | 0.19774010000000006 | 0.1923077 | 0.1654676 | 0.18224300000000007 | 0.199115 | 0.15909090000000006 | 0.1604938 | 0.1677019 | 0.11673150000000003 | 0.1637427 | 0.21897810000000006 | 0.2608696000000001 | 0.1857924000000001 | 0.21276600000000007 | 0.10975610000000004 | 0.1965318 | 0.1704036 | 0.15920400000000007 |
| 0.34 | 0.14814810000000006 | 0.10169490000000003 | 0.1373626 | 0.0935251800000001 | 0.1308411 | 0.12389380000000003 | 0.18181820000000007 | 0.1049383 | 0.1180124 | 0.08949416000000004 | 0.1111111 | 0.15328470000000005 | 0.10326090000000003 | 0.09289618000000002 | 0.09042553 | 0.13414630000000005 | 0.1445087 | 0.14349780000000006 | 0.15920400000000007 |
| 0.3600000000000001 | 0.06878307000000003 | 0.07909604000000003 | 0.06043956 | 0.08633094000000002 | 0.08878504000000004 | 0.05309734 | 0.04090909 | 0.0308642 | 0.10559010000000005 | 0.058365760000000017 | 0.1461988 | 0.06569343000000002 | 0.05978261000000004 | 0.038251370000000014 | 0.07446808000000003 | 0.12195120000000002 | 0.09248555 | 0.1121076 | 0.07462686 |
| 0.38000000000000012 | 0.03703704000000002 | 0.06214689 | 0.06593407 | 0.03597122000000001 | 0.03738318 | 0.05752213 | 0.027272730000000005 | 0.00617284 | 0.0931677 | 0.0389105 | 0.1169591 | 0.05109489 | 0.043478259999999984 | 0.016393440000000002 | 0.03723404000000002 | 0.10975610000000004 | 0.04624276999999999 | 0.035874440000000014 | 0.03482587 |
| 0.4 | 0.026455030000000008 | 0.01129944 | 0.07142857 | 0.02877698 | 0.009345794000000004 | 0.02654867 | 0.01363636 | 0.02469136 | 0.05590062000000002 | 0.0 | 0.08187135000000002 | 0.021897810000000014 | 0.010869570000000007 | 0.01092896 | 0.0106383 | 0.048780490000000024 | 0.034682080000000004 | 0.008968610000000005 | 0.004975124000000002 |
| 0.4200000000000001 | 0.0 | 0.01129944 | 0.01098901 | 0.02877698 | 0.014018689999999995 | 0.0 | 0.004545454 | 0.0 | 0.01242236 | 0.0 | 0.08187135000000002 | 0.029197079999999997 | 0.005434783000000002 | 0.005464481 | 0.021276600000000007 | 0.03658536000000001 | 0.0 | 0.004484305000000002 | 0.014925370000000004 |
| 0.44 | 0.0 | 0.01129944 | 0.01098901 | 0.01438849 | 0.0 | 0.013274339999999999 | 0.004545454 | 0.0 | 0.0 | 0.003891051000000001 | 0.02339181 | 0.0072992700000000035 | 0.0 | 0.005464481 | 0.005319149000000001 | 0.048780490000000024 | 0.0057803470000000025 | 0.004484305000000002 | 0.0 |
| 0.46 | 0.0 | 0.0 | 0.01098901 | 0.007194245000000002 | 0.0 | 0.004424779000000002 | 0.0 | 0.0 | 0.0 | 0.003891051000000001 | 0.05263158 | 0.0 | 0.005434783000000002 | 0.0 | 0.0 | 0.03658536000000001 | 0.0 | 0.0 | 0.004975124000000002 |
| 0.48000000000000009 | 0.0 | 0.0 | 0.01098901 | 0.0 | 0.0 | 0.0 | 0.0 | 0.0 | 0.0 | 0.0 | 0.01754386 | 0.0 | 0.0 | 0.0 | 0.0 | 0.0 | 0.0 | 0.0 | 0.0 |
| 0.5 | 0.0 | 0.0 | 0.0 | 0.0 | 0.0 | 0.0 | 0.0 | 0.0 | 0.0 | 0.0 | 0.0 | 0.0072992700000000035 | 0.0 | 0.0 | 0.0 | 0.0 | 0.0057803470000000025 | 0.0 | 0.0 |
| 0.52 | 0.0 | 0.0 | 0.0 | 0.0 | 0.0 | 0.0 | 0.0 | 0.0 | 0.006211180000000002 | 0.0 | 0.005847953 | 0.0 | 0.0 | 0.0 | 0.0 | 0.0 | 0.0 | 0.0 | 0.0 |
| 0.54 | 0.0 | 0.0 | 0.0 | 0.0 | 0.0 | 0.0 | 0.0 | 0.0 | 0.0 | 0.0 | 0.0 | 0.0 | 0.0 | 0.0 | 0.0 | 0.0 | 0.0 | 0.0 | 0.0 |
| 0.56000000000000005 | 0.0 | 0.0 | 0.0 | 0.0 | 0.0 | 0.0 | 0.0 | 0.0 | 0.0 | 0.0 | 0.0 | 0.0 | 0.0 | 0.0 | 0.0 | 0.0 | 0.0 | 0.0 | 0.0 |
| 0.58000000000000007 | 0.0 | 0.0 | 0.0 | 0.0 | 0.0 | 0.0 | 0.0 | 0.0 | 0.0 | 0.0 | 0.0 | 0.0 | 0.0 | 0.0 | 0.0 | 0.0 | 0.0 | 0.0 | 0.0 |
| 0.6000000000000002 | 0.0 | 0.0 | 0.0 | 0.0 | 0.0 | 0.004424779000000002 | 0.0 | 0.0 | 0.0 | 0.0 | 0.0 | 0.0 | 0.0 | 0.0 | 0.0 | 0.0 | 0.0 | 0.0 | 0.0 |
| 0.62000000000000022 | 0.0 | 0.0 | 0.0 | 0.0 | 0.0 | 0.0 | 0.0 | 0.0 | 0.0 | 0.0 | 0.0 | 0.0 | 0.0 | 0.0 | 0.0 | 0.0 | 0.0 | 0.0 | 0.0 |
| 0.64000000000000024 | 0.0 | 0.0 | 0.0 | 0.0 | 0.0 | 0.0 | 0.0 | 0.0 | 0.0 | 0.0 | 0.0 | 0.0 | 0.0 | 0.0 | 0.0 | 0.0 | 0.0 | 0.0 | 0.0 |
| 0.66000000000000025 | 0.0 | 0.0 | 0.0 | 0.0 | 0.0 | 0.0 | 0.0 | 0.0 | 0.0 | 0.0 | 0.0 | 0.0 | 0.0 | 0.0 | 0.0 | 0.0 | 0.0 | 0.0 | 0.0 |
| 0.68 | 0.0 | 0.0 | 0.0 | 0.0 | 0.0 | 0.0 | 0.0 | 0.0 | 0.0 | 0.0 | 0.0 | 0.0 | 0.0 | 0.0 | 0.0 | 0.0 | 0.0 | 0.0 | 0.0 |
| 0.70000000000000018 | 0.0 | 0.0 | 0.0 | 0.0 | 0.0 | 0.0 | 0.0 | 0.0 | 0.0 | 0.0 | 0.0 | 0.0 | 0.0 | 0.0 | 0.0 | 0.0 | 0.0 | 0.0 | 0.0 |
### Chart: nmr-1 A3
| Category | nmr-1 A3 No.1 | nmr-1 A3 No.2 | nmr-1 A3 No.3 | nmr-1 A3 No.4 | nmr-1 A3 No.5 | nmr-1 A3 No.6 | nmr-1 A3 No.7 | nmr-1 A3 No.8 | nmr-1 A3 No.9 | nmr-1 A3 No.10 | nmr-1 A3 No.11 | nmr-1 A3 No.12 | nmr-1 A3 No.13 | nmr-1 A3 No.14 | nmr-1 A3 No.15 | nmr-1 A3 No.16 | nmr-1 A3 No.17 | nmr-1 A3 No.18 | nmr-1 A3 No.19 | nmr-1 A3 No.20 |
|---|---|---|---|---|---|---|---|---|---|---|---|---|---|---|---|---|---|---|---|---|
| 0 | None | None | None | None | None | None | None | None | None | None | None | None | None | None | None | None | None | None | None | None |
| 2.0000000000000007E-2 | 0.0 | 0.0 | 0.0 | 0.0 | 0.005952381 | 0.0 | 0.004504504999999998 | 0.0 | 0.0 | 0.0 | 0.0 | 0.0 | 0.0 | 0.02654867 | 0.0 | 0.0 | 0.0 | 0.0 | 0.0 | 0.006756757000000002 |
| 4.0000000000000015E-2 | 0.0 | 0.0 | 0.0 | 0.0 | 0.0 | 0.0070422530000000035 | 0.0 | 0.0 | 0.0 | 0.0 | 0.0 | 0.0 | 0.0 | 0.0 | 0.0 | 0.0 | 0.0 | 0.0 | 0.0 | 0.0 |
| 6.0000000000000019E-2 | 0.0 | 0.0 | 0.0 | 0.0 | 0.0 | 0.0 | 0.0 | 0.0 | 0.0 | 0.0 | 0.0 | 0.0 | 0.0 | 0.008849557000000006 | 0.0 | 0.0 | 0.0 | 0.0 | 0.0 | 0.0 |
| 8.0000000000000029E-2 | 0.0 | 0.0 | 0.0 | 0.0 | 0.0 | 0.0 | 0.004504504999999998 | 0.0 | 0.0 | 0.0 | 0.0 | 0.0 | 0.0 | 0.0 | 0.0 | 0.0 | 0.0 | 0.0 | 0.0 | 0.0 |
| 0.1 | 0.0 | 0.0 | 0.005586592000000002 | 0.0 | 0.0 | 0.0 | 0.0 | 0.0 | 0.0 | 0.004291845 | 0.0 | 0.0 | 0.0 | 0.0 | 0.0 | 0.0 | 0.0 | 0.0 | 0.0 | 0.0 |
| 0.12000000000000002 | 0.0 | 0.0 | 0.0 | 0.0 | 0.005952381 | 0.0 | 0.0 | 0.0 | 0.0 | 0.004291845 | 0.0 | 0.0 | 0.0 | 0.01769911 | 0.0 | 0.0 | 0.0 | 0.0 | 0.0 | 0.0 |
| 0.14000000000000001 | 0.0 | 0.0 | 0.0 | 0.0 | 0.0 | 0.0 | 0.0 | 0.0 | 0.0 | 0.0 | 0.0 | 0.0 | 0.0 | 0.0 | 0.0 | 0.0 | 0.0 | 0.0 | 0.0 | 0.0 |
| 0.16 | 0.0 | 0.0 | 0.005586592000000002 | 0.0 | 0.0 | 0.0 | 0.0 | 0.0 | 0.004273504000000002 | 0.0 | 0.0 | 0.0 | 0.0 | 0.0 | 0.0 | 0.0 | 0.0 | 0.0 | 0.0 | 0.006756757000000002 |
| 0.18000000000000005 | 0.0 | 0.0 | 0.0 | 0.0 | 0.005952381 | 0.0070422530000000035 | 0.0 | 0.0 | 0.008547009000000003 | 0.0 | 0.0 | 0.0 | 0.0 | 0.008849557000000006 | 0.0 | 0.0 | 0.010256409999999999 | 0.0 | 0.0 | 0.0 |
| 0.2 | 0.0 | 0.0 | 0.011173180000000001 | 0.0 | 0.005952381 | 0.0 | 0.0 | 0.006756757000000002 | 0.004273504000000002 | 0.0 | 0.0 | 0.0 | 0.0 | 0.01769911 | 0.0 | 0.0 | 0.0 | 0.0 | 0.0 | 0.0 |
| 0.22 | 0.004854369 | 0.0 | 0.022346370000000008 | 0.0 | 0.0 | 0.0 | 0.01801802 | 0.03378378 | 0.008547009000000003 | 0.01716738 | 0.0 | 0.01069519 | 0.02453988 | 0.0 | 0.0 | 0.0 | 0.020512819999999998 | 0.005952381 | 0.0 | 0.0 |
| 0.24000000000000005 | 0.01941748 | 0.027932960000000014 | 0.011173180000000001 | 0.0 | 0.01785714 | 0.02816901 | 0.045045039999999995 | 0.054054060000000015 | 0.03846154 | 0.03862661 | 0.014423080000000001 | 0.02139037 | 0.08588957000000001 | 0.008849557000000006 | 0.0 | 0.013605440000000003 | 0.04615384999999998 | 0.011904760000000004 | 0.05464481 | 0.0 |
| 0.26 | 0.038834950000000014 | 0.050279329999999976 | 0.06703911 | 0.01315789 | 0.02380952 | 0.03521127000000002 | 0.1711712 | 0.12162160000000004 | 0.08119658 | 0.1158798 | 0.03365385 | 0.06951872000000003 | 0.196319 | 0.02654867 | 0.021276600000000007 | 0.17006800000000005 | 0.1282051 | 0.04166667000000002 | 0.08743168999999999 | 0.06081081000000002 |
| 0.28000000000000008 | 0.1116505 | 0.11173180000000002 | 0.1340782 | 0.10526320000000006 | 0.07738096 | 0.07042254000000002 | 0.17567569999999993 | 0.19594590000000006 | 0.1410256 | 0.1845494000000001 | 0.07692308000000002 | 0.2245989 | 0.21472390000000005 | 0.08849557000000005 | 0.12765959999999996 | 0.2517007 | 0.2666667000000001 | 0.08928572000000001 | 0.30054640000000016 | 0.10135139999999995 |
| 0.3000000000000001 | 0.2427184000000001 | 0.20111730000000005 | 0.20670390000000005 | 0.15789470000000005 | 0.13095240000000005 | 0.1619718 | 0.24774770000000007 | 0.20945950000000005 | 0.2051282 | 0.22746780000000005 | 0.12019230000000003 | 0.2406417 | 0.22699390000000005 | 0.1150443 | 0.1595745 | 0.2517007 | 0.17948720000000007 | 0.1904762 | 0.2950820000000001 | 0.2567568 |
| 0.32000000000000012 | 0.1796117 | 0.21229050000000005 | 0.1675978 | 0.18421050000000005 | 0.1904762 | 0.1901408 | 0.13063059999999996 | 0.1418919 | 0.1923077 | 0.17596570000000006 | 0.09134615000000001 | 0.2513369000000001 | 0.1104295 | 0.2300885 | 0.17021280000000005 | 0.1428571 | 0.14871800000000007 | 0.2261905 | 0.1693989 | 0.2837838000000001 |
| 0.34 | 0.1601942 | 0.18994410000000012 | 0.15083800000000006 | 0.09210526000000001 | 0.202381 | 0.1619718 | 0.11261259999999998 | 0.1283784 | 0.15811970000000006 | 0.1287554 | 0.1634615 | 0.12299470000000005 | 0.07975460000000002 | 0.1415929 | 0.12765959999999996 | 0.10884350000000002 | 0.09230769 | 0.1785714 | 0.04371585 | 0.2297297 |
| 0.3600000000000001 | 0.15048540000000007 | 0.08938547000000002 | 0.08379889000000007 | 0.09210526000000001 | 0.1488095 | 0.1267606 | 0.04954954999999998 | 0.054054060000000015 | 0.08974359000000003 | 0.04291845 | 0.1298077 | 0.03743315000000001 | 0.036809820000000014 | 0.12389380000000003 | 0.17021280000000005 | 0.040816330000000026 | 0.06153846 | 0.11309520000000005 | 0.032786880000000004 | 0.047297300000000014 |
| 0.38000000000000012 | 0.024271840000000013 | 0.06145252 | 0.06145252 | 0.15789470000000005 | 0.08928572000000001 | 0.07042254000000002 | 0.013513510000000001 | 0.013513510000000001 | 0.029914529999999995 | 0.03862661 | 0.12019230000000003 | 0.016042780000000006 | 0.012269939999999998 | 0.13274340000000007 | 0.053191490000000015 | 0.013605440000000003 | 0.010256409999999999 | 0.08333334000000003 | 0.016393440000000002 | 0.006756757000000002 |
| 0.4 | 0.048543690000000014 | 0.027932960000000014 | 0.027932960000000014 | 0.11842110000000003 | 0.04166667000000002 | 0.06338028 | 0.009009009000000004 | 0.013513510000000001 | 0.008547009000000003 | 0.01716738 | 0.07692308000000002 | 0.0 | 0.0 | 0.03539823 | 0.04255319 | 0.006802721000000002 | 0.010256409999999999 | 0.04166667000000002 | 0.0 | 0.0 |
| 0.4200000000000001 | 0.004854369 | 0.016759780000000005 | 0.016759780000000005 | 0.01315789 | 0.04166667000000002 | 0.07042254000000002 | 0.009009009000000004 | 0.006756757000000002 | 0.012820510000000004 | 0.004291845 | 0.07211538000000002 | 0.0 | 0.0 | 0.008849557000000006 | 0.021276600000000007 | 0.0 | 0.005128205 | 0.0 | 0.0 | 0.0 |
| 0.44 | 0.009708738000000001 | 0.0 | 0.0 | 0.03947368000000001 | 0.005952381 | 0.0 | 0.004504504999999998 | 0.006756757000000002 | 0.012820510000000004 | 0.0 | 0.048076920000000016 | 0.005347594 | 0.006134969 | 0.008849557000000006 | 0.06382979 | 0.0 | 0.010256409999999999 | 0.01785714 | 0.0 | 0.0 |
| 0.46 | 0.0 | 0.005586592000000002 | 0.011173180000000001 | 0.01315789 | 0.0 | 0.0 | 0.0 | 0.0 | 0.0 | 0.0 | 0.02403846 | 0.0 | 0.0 | 0.0 | 0.0106383 | 0.0 | 0.0 | 0.0 | 0.0 | 0.0 |
| 0.48000000000000009 | 0.0 | 0.005586592000000002 | 0.005586592000000002 | 0.0 | 0.005952381 | 0.0 | 0.004504504999999998 | 0.006756757000000002 | 0.004273504000000002 | 0.0 | 0.004807692000000002 | 0.0 | 0.0 | 0.0 | 0.0 | 0.0 | 0.010256409999999999 | 0.0 | 0.0 | 0.0 |
| 0.5 | 0.0 | 0.0 | 0.0 | 0.0 | 0.0 | 0.0 | 0.0 | 0.0 | 0.0 | 0.0 | 0.004807692000000002 | 0.0 | 0.0 | 0.0 | 0.0 | 0.0 | 0.0 | 0.0 | 0.0 | 0.0 |
| 0.52 | 0.004854369 | 0.0 | 0.0 | 0.0 | 0.0 | 0.0070422530000000035 | 0.0 | 0.0 | 0.0 | 0.0 | 0.0 | 0.0 | 0.006134969 | 0.0 | 0.0106383 | 0.0 | 0.0 | 0.0 | 0.0 | 0.0 |
| 0.54 | 0.0 | 0.0 | 0.005586592000000002 | 0.0 | 0.0 | 0.0 | 0.0 | 0.0 | 0.0 | 0.0 | 0.009615385 | 0.0 | 0.0 | 0.0 | 0.0106383 | 0.0 | 0.0 | 0.0 | 0.0 | 0.0 |
| 0.56000000000000005 | 0.0 | 0.0 | 0.0 | 0.0 | 0.0 | 0.0 | 0.0 | 0.0 | 0.0 | 0.0 | 0.0 | 0.0 | 0.0 | 0.0 | 0.0106383 | 0.0 | 0.0 | 0.0 | 0.0 | 0.0 |
| 0.58000000000000007 | 0.0 | 0.0 | 0.0 | 0.0 | 0.0 | 0.0 | 0.0 | 0.0 | 0.0 | 0.0 | 0.0 | 0.0 | 0.0 | 0.0 | 0.0 | 0.0 | 0.0 | 0.0 | 0.0 | 0.0 |
| 0.6000000000000002 | 0.0 | 0.0 | 0.0 | 0.0 | 0.0 | 0.0 | 0.0 | 0.0 | 0.0 | 0.0 | 0.004807692000000002 | 0.0 | 0.0 | 0.0 | 0.0 | 0.0 | 0.0 | 0.0 | 0.0 | 0.0 |
| 0.62000000000000022 | 0.0 | 0.0 | 0.0 | 0.0 | 0.0 | 0.0 | 0.0 | 0.0 | 0.0 | 0.0 | 0.004807692000000002 | 0.0 | 0.0 | 0.0 | 0.0 | 0.0 | 0.0 | 0.0 | 0.0 | 0.0 |
| 0.64000000000000024 | 0.0 | 0.0 | 0.005586592000000002 | 0.0 | 0.0 | 0.0 | 0.0 | 0.006756757000000002 | 0.0 | 0.0 | 0.0 | 0.0 | 0.0 | 0.0 | 0.0 | 0.0 | 0.0 | 0.0 | 0.0 | 0.0 |
| 0.66000000000000025 | 0.0 | 0.0 | 0.0 | 0.01315789 | 0.0 | 0.0 | 0.0 | 0.0 | 0.0 | 0.0 | 0.0 | 0.0 | 0.0 | 0.0 | 0.0 | 0.0 | 0.0 | 0.0 | 0.0 | 0.0 |
| 0.68 | 0.0 | 0.0 | 0.0 | 0.0 | 0.0 | 0.0 | 0.0 | 0.0 | 0.0 | 0.0 | 0.0 | 0.0 | 0.0 | 0.0 | 0.0 | 0.0 | 0.0 | 0.0 | 0.0 | 0.0 |
| 0.70000000000000018 | 0.0 | 0.0 | 0.0 | 0.0 | 0.0 | 0.0 | 0.0 | 0.0 | 0.0 | 0.0 | 0.0 | 0.0 | 0.0 | 0.0 | 0.0 | 0.0 | 0.0 | 0.0 | 0.0 | 0.0 |
### Chart: nmr-1 A5
| Category | nmr-1 A5 No.1 | nmr-1 A5 No.2 | nmr-1 A5 No.3 | nmr-1 A5 No.4 | nmr-1 A5 No.6 | nmr-1 A5 No.7 | nmr-1 A5 No.8 | nmr-1 A5 No.9 | nmr-1 A5 No.10 | nmr-1 A5 No.11 | nmr-1 A5 No.12 | nmr-1 A5 No.13 | nmr-1 A5 No.14 | nmr-1 A5 No.15 | nmr-1 A5 No.16 | nmr-1 A5 No.17 | nmr-1 A5 No.18 | nmr-1 A5 No.19 | nmr-1 A5 No.20 |
|---|---|---|---|---|---|---|---|---|---|---|---|---|---|---|---|---|---|---|---|
| 0 | None | None | None | None | None | None | None | None | None | None | None | None | None | None | None | None | None | None | None |
| 2.0000000000000007E-2 | 0.0 | 0.008474576000000004 | 0.0 | 0.01666667 | 0.0 | 0.0 | 0.0 | 0.0 | 0.0 | 0.0 | 0.0 | 0.005617978 | 0.0 | 0.0 | 0.01621622 | 0.0 | 0.0 | 0.0 | 0.0 |
| 4.0000000000000015E-2 | 0.0 | 0.008474576000000004 | 0.0 | 0.0 | 0.0 | 0.0 | 0.0 | 0.007142857 | 0.007575758000000004 | 0.0 | 0.0 | 0.01123596 | 0.0 | 0.0 | 0.01081081 | 0.0052083330000000025 | 0.011764710000000001 | 0.0 | 0.0 |
| 6.0000000000000019E-2 | 0.0 | 0.008474576000000004 | 0.0 | 0.0 | 0.0 | 0.0 | 0.0 | 0.0 | 0.0 | 0.0 | 0.0 | 0.005617978 | 0.0 | 0.0 | 0.005405406 | 0.0 | 0.005882353 | 0.0 | 0.0 |
| 8.0000000000000029E-2 | 0.0 | 0.0 | 0.0 | 0.0 | 0.0 | 0.0 | 0.0 | 0.0 | 0.0 | 0.0 | 0.0 | 0.0 | 0.0 | 0.0 | 0.005405406 | 0.0 | 0.0 | 0.0 | 0.0 |
| 0.1 | 0.0 | 0.008474576000000004 | 0.0 | 0.0 | 0.0 | 0.0 | 0.0 | 0.0 | 0.0 | 0.0 | 0.0 | 0.0 | 0.0 | 0.0 | 0.005405406 | 0.0 | 0.0 | 0.0 | 0.0 |
| 0.12000000000000002 | 0.006289308000000004 | 0.0 | 0.0 | 0.0 | 0.0 | 0.0 | 0.0 | 0.007142857 | 0.0 | 0.0 | 0.0 | 0.0 | 0.0 | 0.0 | 0.005405406 | 0.0 | 0.0 | 0.0 | 0.0 |
| 0.14000000000000001 | 0.006289308000000004 | 0.0 | 0.0 | 0.0 | 0.0 | 0.0 | 0.0 | 0.0 | 0.0 | 0.0 | 0.0 | 0.0 | 0.004524886999999997 | 0.0 | 0.0 | 0.0 | 0.011764710000000001 | 0.0 | 0.0 |
| 0.16 | 0.0 | 0.0 | 0.0 | 0.0 | 0.0 | 0.0 | 0.0 | 0.0 | 0.0 | 0.0 | 0.0 | 0.0 | 0.0 | 0.0 | 0.0 | 0.0 | 0.0 | 0.0 | 0.0 |
| 0.18000000000000005 | 0.0 | 0.0 | 0.0 | 0.0 | 0.0 | 0.0 | 0.0 | 0.0 | 0.0 | 0.0 | 0.0 | 0.0 | 0.0 | 0.01129944 | 0.01081081 | 0.010416669999999998 | 0.011764710000000001 | 0.007142857 | 0.0 |
| 0.2 | 0.0 | 0.008474576000000004 | 0.0 | 0.008333334000000003 | 0.0 | 0.021052629999999992 | 0.006211180000000002 | 0.02142857 | 0.0 | 0.0 | 0.005714286 | 0.0 | 0.0 | 0.0 | 0.01081081 | 0.0 | 0.023529409999999994 | 0.01428571 | 0.0 |
| 0.22 | 0.0 | 0.008474576000000004 | 0.0 | 0.0 | 0.0 | 0.03157895 | 0.0 | 0.02142857 | 0.0 | 0.0 | 0.017142860000000003 | 0.01123596 | 0.01357466 | 0.02824859 | 0.01621622 | 0.0 | 0.06470589000000003 | 0.03571429 | 0.012658229999999998 |
| 0.24000000000000005 | 0.006289308000000004 | 0.059322030000000026 | 0.0 | 0.0 | 0.0 | 0.05789474000000002 | 0.0 | 0.02142857 | 0.022727270000000008 | 0.0 | 0.05714285999999998 | 0.02247191000000002 | 0.031674210000000015 | 0.06779661000000003 | 0.005405406 | 0.010416669999999998 | 0.06470589000000003 | 0.04285714 | 0.006329114 |
| 0.26 | 0.03144654 | 0.06779661000000003 | 0.008064516000000004 | 0.025 | 0.01948052000000001 | 0.08421053000000002 | 0.006211180000000002 | 0.09285714000000005 | 0.07575758000000003 | 0.019354840000000005 | 0.06857143 | 0.0505618 | 0.04524886999999997 | 0.11299439999999998 | 0.07027027 | 0.020833330000000018 | 0.1058824 | 0.09285714000000005 | 0.012658229999999998 |
| 0.28000000000000008 | 0.05031446000000002 | 0.08474576000000003 | 0.016129030000000003 | 0.03333334000000002 | 0.01948052000000001 | 0.2 | 0.02484472 | 0.1785714 | 0.1439394000000001 | 0.03870968 | 0.1771429 | 0.06179775 | 0.07239819000000003 | 0.21468930000000006 | 0.09189189000000003 | 0.046874999999999986 | 0.1176471 | 0.1857143000000001 | 0.07594935999999997 |
| 0.3000000000000001 | 0.16981130000000005 | 0.08474576000000003 | 0.08870967000000003 | 0.1416667 | 0.11038959999999996 | 0.1947368 | 0.08695652000000004 | 0.2785714 | 0.18181820000000007 | 0.09677419000000004 | 0.2114286 | 0.1292135 | 0.18099550000000006 | 0.18079100000000006 | 0.1351351 | 0.16145830000000005 | 0.07647059000000002 | 0.1642857 | 0.11392400000000003 |
| 0.32000000000000012 | 0.1257862 | 0.220339 | 0.18548390000000006 | 0.2 | 0.1688312 | 0.14736840000000007 | 0.1304348 | 0.1785714 | 0.24242420000000006 | 0.16774190000000005 | 0.20571430000000007 | 0.1348315 | 0.1628959 | 0.1638418 | 0.1621622 | 0.19270830000000005 | 0.09411765000000002 | 0.1285714 | 0.13291140000000007 |
| 0.34 | 0.1446541 | 0.14406780000000005 | 0.16129030000000005 | 0.1666667 | 0.20129870000000005 | 0.1263158 | 0.1987578 | 0.10714290000000003 | 0.17424240000000007 | 0.16129030000000005 | 0.12000000000000002 | 0.1629214 | 0.158371 | 0.10169490000000003 | 0.11891889999999995 | 0.125 | 0.07647059000000002 | 0.11428570000000003 | 0.1708861 |
| 0.3600000000000001 | 0.1446541 | 0.1101695 | 0.15322580000000005 | 0.15833330000000007 | 0.1753247 | 0.05789474000000002 | 0.2111801 | 0.05 | 0.07575758000000003 | 0.15483870000000005 | 0.05714285999999998 | 0.1011236 | 0.1312217 | 0.06214689 | 0.1027027 | 0.078125 | 0.05882353 | 0.03571429 | 0.13291140000000007 |
| 0.38000000000000012 | 0.0754717 | 0.05084746 | 0.10483870000000002 | 0.09166667000000003 | 0.09740259 | 0.015789470000000003 | 0.1304348 | 0.007142857 | 0.03030303 | 0.1290323 | 0.03428571000000001 | 0.07865169 | 0.054298640000000016 | 0.022598870000000007 | 0.09189189000000003 | 0.10937500000000003 | 0.052941179999999984 | 0.05714285999999998 | 0.06962025 |
| 0.4 | 0.06918239000000002 | 0.016949150000000007 | 0.08064516000000004 | 0.05 | 0.05194804999999998 | 0.02631579 | 0.08695652000000004 | 0.02142857 | 0.007575758000000004 | 0.03225806 | 0.017142860000000003 | 0.09550562000000005 | 0.0361991 | 0.01129944 | 0.027027030000000007 | 0.0625 | 0.03529412 | 0.03571429 | 0.05696202000000002 |
| 0.4200000000000001 | 0.037735850000000015 | 0.05084746 | 0.08870967000000003 | 0.03333334000000002 | 0.04545455 | 0.015789470000000003 | 0.01242236 | 0.007142857 | 0.022727270000000008 | 0.058064520000000015 | 0.017142860000000003 | 0.04494382 | 0.02262443 | 0.016949150000000007 | 0.027027030000000007 | 0.08333334000000003 | 0.04705882 | 0.007142857 | 0.05696202000000002 |
| 0.44 | 0.02515723 | 0.008474576000000004 | 0.03225806 | 0.008333334000000003 | 0.04545455 | 0.005263158 | 0.043478259999999984 | 0.0 | 0.007575758000000004 | 0.045161290000000014 | 0.0 | 0.01685393 | 0.02262443 | 0.005649718 | 0.01621622 | 0.02604167000000001 | 0.03529412 | 0.02142857 | 0.025316459999999985 |
| 0.46 | 0.02515723 | 0.008474576000000004 | 0.024193549999999998 | 0.025 | 0.02597403 | 0.01052632 | 0.03726708 | 0.0 | 0.0 | 0.01290323 | 0.005714286 | 0.03932584 | 0.01357466 | 0.0 | 0.01621622 | 0.020833330000000018 | 0.01764706 | 0.02142857 | 0.018987340000000002 |
| 0.48000000000000009 | 0.02515723 | 0.02542373 | 0.008064516000000004 | 0.008333334000000003 | 0.006493506000000002 | 0.0 | 0.006211180000000002 | 0.0 | 0.0 | 0.025806449999999998 | 0.0 | 0.005617978 | 0.01357466 | 0.0 | 0.005405406 | 0.010416669999999998 | 0.03529412 | 0.007142857 | 0.04430380000000002 |
| 0.5 | 0.012578619999999997 | 0.0 | 0.016129030000000003 | 0.0 | 0.01948052000000001 | 0.0 | 0.006211180000000002 | 0.0 | 0.0 | 0.0 | 0.0 | 0.0 | 0.004524886999999997 | 0.0 | 0.01081081 | 0.010416669999999998 | 0.011764710000000001 | 0.02142857 | 0.006329114 |
| 0.52 | 0.012578619999999997 | 0.0 | 0.008064516000000004 | 0.008333334000000003 | 0.006493506000000002 | 0.0 | 0.0 | 0.0 | 0.0 | 0.019354840000000005 | 0.0 | 0.0 | 0.009049774 | 0.0 | 0.005405406 | 0.0 | 0.0 | 0.0 | 0.006329114 |
| 0.54 | 0.012578619999999997 | 0.0 | 0.0 | 0.0 | 0.006493506000000002 | 0.0 | 0.0 | 0.0 | 0.0 | 0.01290323 | 0.005714286 | 0.0 | 0.01357466 | 0.0 | 0.005405406 | 0.0 | 0.03529412 | 0.0 | 0.012658229999999998 |
| 0.56000000000000005 | 0.006289308000000004 | 0.008474576000000004 | 0.008064516000000004 | 0.0 | 0.0 | 0.0 | 0.006211180000000002 | 0.0 | 0.0 | 0.0 | 0.0 | 0.005617978 | 0.0 | 0.0 | 0.01621622 | 0.010416669999999998 | 0.005882353 | 0.007142857 | 0.006329114 |
| 0.58000000000000007 | 0.0 | 0.0 | 0.0 | 0.008333334000000003 | 0.0 | 0.0 | 0.006211180000000002 | 0.0 | 0.007575758000000004 | 0.006451613000000004 | 0.0 | 0.0 | 0.004524886999999997 | 0.0 | 0.0 | 0.0052083330000000025 | 0.0 | 0.0 | 0.006329114 |
| 0.6000000000000002 | 0.0 | 0.0 | 0.0 | 0.0 | 0.0 | 0.0 | 0.0 | 0.0 | 0.0 | 0.006451613000000004 | 0.0 | 0.005617978 | 0.004524886999999997 | 0.0 | 0.005405406 | 0.0 | 0.0 | 0.0 | 0.012658229999999998 |
| 0.62000000000000022 | 0.0 | 0.0 | 0.008064516000000004 | 0.0 | 0.0 | 0.005263158 | 0.0 | 0.0 | 0.0 | 0.006451613000000004 | 0.0 | 0.005617978 | 0.0 | 0.0 | 0.0 | 0.0052083330000000025 | 0.0 | 0.0 | 0.0 |
| 0.64000000000000024 | 0.0 | 0.0 | 0.0 | 0.0 | 0.0 | 0.0 | 0.0 | 0.0 | 0.0 | 0.0 | 0.0 | 0.0 | 0.0 | 0.0 | 0.0 | 0.0 | 0.0 | 0.0 | 0.0 |
| 0.66000000000000025 | 0.012578619999999997 | 0.008474576000000004 | 0.008064516000000004 | 0.008333334000000003 | 0.0 | 0.0 | 0.0 | 0.0 | 0.0 | 0.0 | 0.0 | 0.005617978 | 0.0 | 0.0 | 0.0 | 0.0 | 0.0 | 0.0 | 0.006329114 |
| 0.68 | 0.0 | 0.0 | 0.0 | 0.0 | 0.0 | 0.0 | 0.0 | 0.0 | 0.0 | 0.0 | 0.0 | 0.0 | 0.0 | 0.0 | 0.0 | 0.0 | 0.0 | 0.0 | 0.0 |
| 0.70000000000000018 | 0.0 | 0.0 | 0.0 | 0.008333334000000003 | 0.0 | 0.0 | 0.0 | 0.0 | 0.0 | 0.006451613000000004 | 0.0 | 0.0 | 0.0 | 0.0 | 0.0 | 0.0052083330000000025 | 0.0 | 0.0 | 0.006329114 |

## Slide 3
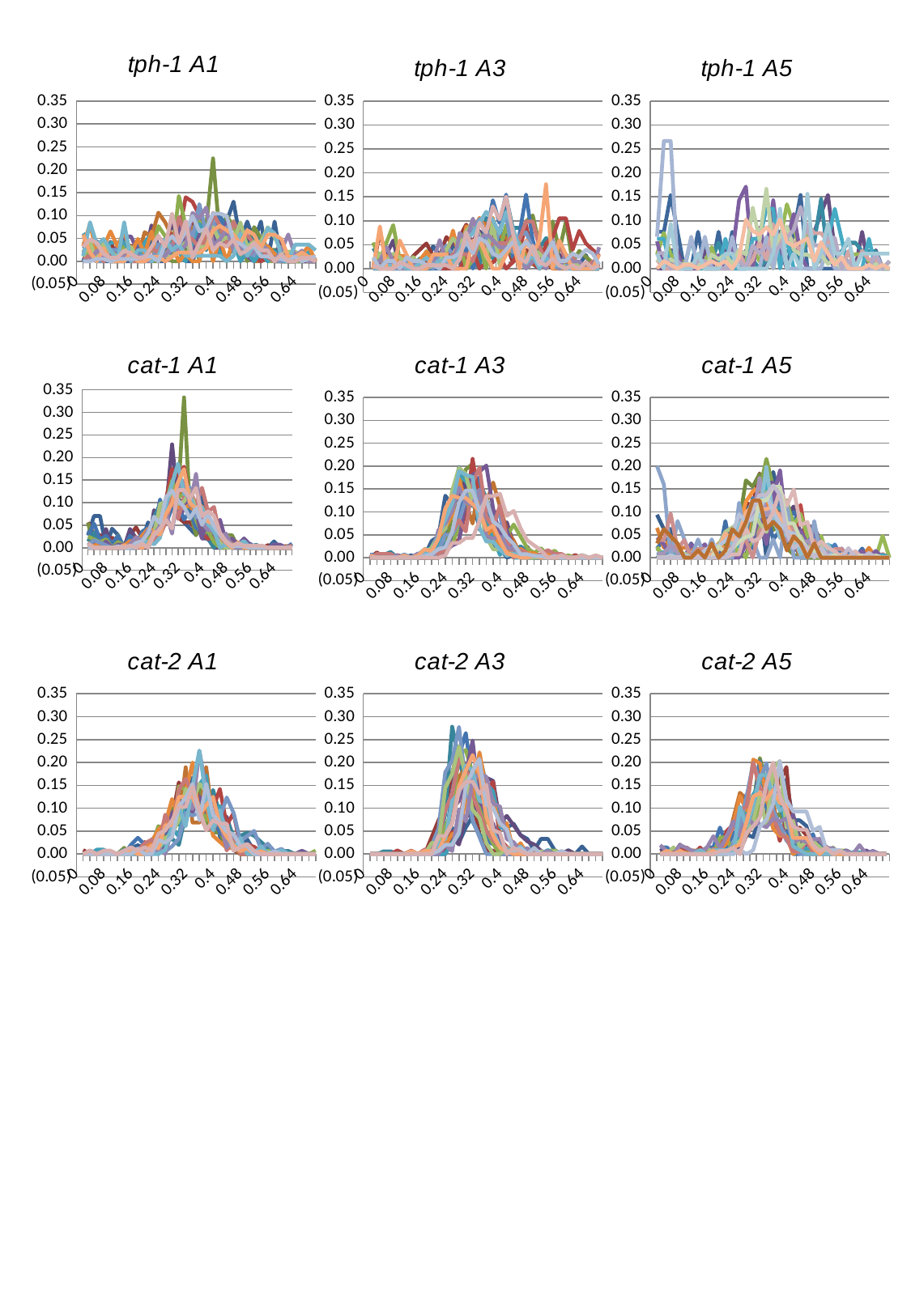

### Chart: tph-1 A1
| Category | tph-1 A1 No.1 | tph-1 A1 No.2 | tph-1 A1 No.3 | tph-1 A1 No.4 | tph-1 A1 No.5 | tph-1 A1 No.6 | tph-1 A1 No.7 | tph-1 A1 No.8 | tph-1 A1 No.9 | tph-1 A1 No.10 | tph-1 A1 No.11 | tph-1 A1 No.12 | tph-1 A1 No.13 | tph-1 A1 No.14 | tph-1 A1 No.15 | tph-1 A1 No.16 | tph-1 A1 No.17 | tph-1 A1 No.18 | tph-1 A1 No.19 | tph-1 A1 No.20 |
|---|---|---|---|---|---|---|---|---|---|---|---|---|---|---|---|---|---|---|---|---|
| 0 | None | None | None | None | None | None | None | None | None | None | None | None | None | None | None | None | None | None | None | None |
| 2.0000000000000007E-2 | 0.0 | 0.025641030000000006 | 0.025 | 0.03370786 | 0.01219512 | 0.021276600000000007 | 0.043478259999999984 | 0.010000000000000004 | 0.01098901 | 0.043478259999999984 | 0.061224489999999986 | 0.032786880000000004 | 0.0 | 0.009615385 | 0.0106383 | 0.0 | 0.01219512 | 0.05825243000000002 | 0.0 | 0.032000000000000015 |
| 4.0000000000000015E-2 | 0.043478259999999984 | 0.008547009000000003 | 0.025 | 0.02247191000000002 | 0.01219512 | 0.0106383 | 0.02898551 | 0.010000000000000004 | 0.02197802 | 0.043478259999999984 | 0.040816330000000026 | 0.08196721000000001 | 0.0125 | 0.009615385 | 0.04255319 | 0.0 | 0.08536585000000005 | 0.01941748 | 0.0 | 0.048 |
| 6.0000000000000019E-2 | 0.043478259999999984 | 0.0 | 0.0 | 0.0 | 0.0 | 0.0106383 | 0.01449275 | 0.010000000000000004 | 0.03296703 | 0.043478259999999984 | 0.040816330000000026 | 0.016393440000000002 | 0.0 | 0.019230770000000008 | 0.0 | 0.023529409999999994 | 0.03658536000000001 | 0.0 | 0.005494506 | 0.040000000000000015 |
| 8.0000000000000029E-2 | 0.0 | 0.01709402 | 0.0 | 0.01123596 | 0.006097561 | 0.0106383 | 0.02898551 | 0.010000000000000004 | 0.01098901 | 0.0326087 | 0.02040816 | 0.032786880000000004 | 0.0 | 0.0 | 0.04255319 | 0.011764710000000001 | 0.048780490000000024 | 0.02912621 | 0.005494506 | 0.016000000000000007 |
| 0.1 | 0.0 | 0.04273504 | 0.025 | 0.0 | 0.0 | 0.0 | 0.01449275 | 0.014999999999999998 | 0.0 | 0.0 | 0.040816330000000026 | 0.06557377 | 0.025 | 0.009615385 | 0.021276600000000007 | 0.0 | 0.02439024 | 0.0 | 0.0 | 0.008000000000000005 |
| 0.12000000000000002 | 0.0 | 0.008547009000000003 | 0.0 | 0.01123596 | 0.0 | 0.0106383 | 0.01449275 | 0.014999999999999998 | 0.0 | 0.0326087 | 0.040816330000000026 | 0.032786880000000004 | 0.025 | 0.0 | 0.0106383 | 0.0 | 0.0 | 0.0 | 0.005494506 | 0.008000000000000005 |
| 0.14000000000000001 | 0.0 | 0.008547009000000003 | 0.0 | 0.02247191000000002 | 0.01219512 | 0.04255319 | 0.05797102 | 0.010000000000000004 | 0.03296703 | 0.05434782 | 0.040816330000000026 | 0.06557377 | 0.0 | 0.019230770000000008 | 0.021276600000000007 | 0.011764710000000001 | 0.08536585000000005 | 0.0 | 0.005494506 | 0.024 |
| 0.16 | 0.0 | 0.03418804 | 0.025 | 0.01123596 | 0.01219512 | 0.021276600000000007 | 0.0 | 0.005000000000000002 | 0.02197802 | 0.05434782 | 0.02040816 | 0.016393440000000002 | 0.0125 | 0.0 | 0.03191489 | 0.011764710000000001 | 0.0 | 0.01941748 | 0.005494506 | 0.016000000000000007 |
| 0.18000000000000005 | 0.0 | 0.025641030000000006 | 0.0 | 0.03370786 | 0.006097561 | 0.0106383 | 0.043478259999999984 | 0.005000000000000002 | 0.01098901 | 0.02173913 | 0.0 | 0.049180330000000015 | 0.0125 | 0.0 | 0.0 | 0.011764710000000001 | 0.02439024 | 0.0 | 0.005494506 | 0.008000000000000005 |
| 0.2 | 0.0 | 0.03418804 | 0.0 | 0.01123596 | 0.006097561 | 0.06382979 | 0.01449275 | 0.005000000000000002 | 0.02197802 | 0.0 | 0.040816330000000026 | 0.016393440000000002 | 0.0125 | 0.0 | 0.0106383 | 0.0 | 0.02439024 | 0.0 | 0.01098901 | 0.008000000000000005 |
| 0.22 | 0.043478259999999984 | 0.025641030000000006 | 0.0 | 0.07865169 | 0.006097561 | 0.053191490000000015 | 0.02898551 | 0.005000000000000002 | 0.02197802 | 0.0326087 | 0.061224489999999986 | 0.06557377 | 0.0 | 0.02884615 | 0.0 | 0.0 | 0.0 | 0.009708738000000001 | 0.01098901 | 0.032000000000000015 |
| 0.24000000000000005 | 0.0 | 0.025641030000000006 | 0.0 | 0.03370786 | 0.01219512 | 0.10638300000000002 | 0.01449275 | 0.03500000000000001 | 0.07692308000000002 | 0.0326087 | 0.02040816 | 0.0 | 0.037500000000000006 | 0.019230770000000008 | 0.0106383 | 0.011764710000000001 | 0.01219512 | 0.009708738000000001 | 0.0 | 0.056 |
| 0.26 | 0.0 | 0.04273504 | 0.025 | 0.02247191000000002 | 0.02439024 | 0.08510638000000002 | 0.02898551 | 0.040000000000000015 | 0.05494505999999998 | 0.010869570000000007 | 0.040816330000000026 | 0.016393440000000002 | 0.0125 | 0.048076920000000016 | 0.0106383 | 0.011764710000000001 | 0.048780490000000024 | 0.0 | 0.03846154 | 0.032000000000000015 |
| 0.28000000000000008 | 0.043478259999999984 | 0.04273504 | 0.0 | 0.08988764 | 0.03658536000000001 | 0.053191490000000015 | 0.02898551 | 0.06000000000000002 | 0.01098901 | 0.02173913 | 0.040816330000000026 | 0.032786880000000004 | 0.025 | 0.02884615 | 0.0106383 | 0.03529412 | 0.02439024 | 0.009708738000000001 | 0.05494505999999998 | 0.10400000000000002 |
| 0.3000000000000001 | 0.043478259999999984 | 0.05128205 | 0.0 | 0.02247191000000002 | 0.02439024 | 0.08510638000000002 | 0.08695652000000004 | 0.06000000000000002 | 0.1428571 | 0.07608695000000001 | 0.02040816 | 0.0 | 0.025 | 0.09615385000000004 | 0.021276600000000007 | 0.023529409999999994 | 0.03658536000000001 | 0.01941748 | 0.03846154 | 0.040000000000000015 |
| 0.32000000000000012 | 0.0 | 0.04273504 | 0.025 | 0.05617977999999998 | 0.048780490000000024 | 0.04255319 | 0.05797102 | 0.14 | 0.07692308000000002 | 0.0326087 | 0.0 | 0.016393440000000002 | 0.08750000000000001 | 0.05769231000000001 | 0.04255319 | 0.03529412 | 0.01219512 | 0.01941748 | 0.06593407 | 0.08800000000000004 |
| 0.34 | 0.0 | 0.07692308000000002 | 0.07500000000000001 | 0.06741573 | 0.07317073 | 0.07446808000000003 | 0.05797102 | 0.13 | 0.04395605 | 0.02173913 | 0.040816330000000026 | 0.0 | 0.05 | 0.048076920000000016 | 0.0106383 | 0.1058824 | 0.01219512 | 0.01941748 | 0.0989011 | 0.008000000000000005 |
| 0.3600000000000001 | 0.043478259999999984 | 0.10256410000000005 | 0.0 | 0.02247191000000002 | 0.06097561000000001 | 0.03191489 | 0.01449275 | 0.1 | 0.08791209 | 0.06521739000000001 | 0.08163265 | 0.0 | 0.125 | 0.02884615 | 0.03191489 | 0.09411765000000002 | 0.01219512 | 0.01941748 | 0.06593407 | 0.040000000000000015 |
| 0.38000000000000012 | 0.043478259999999984 | 0.05128205 | 0.07500000000000001 | 0.03370786 | 0.02439024 | 0.053191490000000015 | 0.043478259999999984 | 0.07000000000000002 | 0.03296703 | 0.07608695000000001 | 0.061224489999999986 | 0.06557377 | 0.0625 | 0.048076920000000016 | 0.03191489 | 0.1176471 | 0.01219512 | 0.02912621 | 0.07142857 | 0.072 |
| 0.4 | 0.043478259999999984 | 0.05982906 | 0.225 | 0.06741573 | 0.09756097000000005 | 0.04255319 | 0.05797102 | 0.065 | 0.04395605 | 0.07608695000000001 | 0.040816330000000026 | 0.0 | 0.08750000000000001 | 0.1057692 | 0.07446808000000003 | 0.03529412 | 0.01219512 | 0.06796116 | 0.10439560000000005 | 0.024 |
| 0.4200000000000001 | 0.043478259999999984 | 0.05128205 | 0.05 | 0.03370786 | 0.09756097000000005 | 0.021276600000000007 | 0.1014493 | 0.03500000000000001 | 0.04395605 | 0.010869570000000007 | 0.02040816 | 0.049180330000000015 | 0.05 | 0.07692308000000002 | 0.04255319 | 0.04705882 | 0.01219512 | 0.07766990000000001 | 0.10439560000000005 | 0.040000000000000015 |
| 0.44 | 0.08695652000000004 | 0.05982906 | 0.1 | 0.06741573 | 0.07926829000000003 | 0.053191490000000015 | 0.05797102 | 0.040000000000000015 | 0.04395605 | 0.08695652000000004 | 0.0 | 0.0 | 0.07500000000000001 | 0.03846154 | 0.053191490000000015 | 0.07058824 | 0.0 | 0.06796116 | 0.0989011 | 0.032000000000000015 |
| 0.46 | 0.1304348 | 0.025641030000000006 | 0.07500000000000001 | 0.02247191000000002 | 0.07926829000000003 | 0.03191489 | 0.02898551 | 0.020000000000000007 | 0.08791209 | 0.02173913 | 0.040816330000000026 | 0.049180330000000015 | 0.05 | 0.08653846000000004 | 0.04255319 | 0.023529409999999994 | 0.01219512 | 0.05825243000000002 | 0.04395605 | 0.048 |
| 0.48000000000000009 | 0.043478259999999984 | 0.05128205 | 0.05 | 0.02247191000000002 | 0.06097561000000001 | 0.021276600000000007 | 0.043478259999999984 | 0.03500000000000001 | 0.01098901 | 0.02173913 | 0.0 | 0.016393440000000002 | 0.0625 | 0.05769231000000001 | 0.08510638000000002 | 0.03529412 | 0.06097561000000001 | 0.038834950000000014 | 0.03846154 | 0.016000000000000007 |
| 0.5 | 0.08695652000000004 | 0.008547009000000003 | 0.0 | 0.05617977999999998 | 0.030487810000000018 | 0.03191489 | 0.02898551 | 0.03500000000000001 | 0.01098901 | 0.010869570000000007 | 0.02040816 | 0.032786880000000004 | 0.025 | 0.05769231000000001 | 0.03191489 | 0.011764710000000001 | 0.01219512 | 0.06796116 | 0.027472530000000005 | 0.024 |
| 0.52 | 0.043478259999999984 | 0.05128205 | 0.07500000000000001 | 0.02247191000000002 | 0.05487805 | 0.0 | 0.01449275 | 0.010000000000000004 | 0.01098901 | 0.0 | 0.0 | 0.032786880000000004 | 0.0125 | 0.03846154 | 0.04255319 | 0.023529409999999994 | 0.03658536000000001 | 0.05825243000000002 | 0.03296703 | 0.040000000000000015 |
| 0.54 | 0.08695652000000004 | 0.0 | 0.05 | 0.02247191000000002 | 0.04268293000000003 | 0.0 | 0.0 | 0.0 | 0.03296703 | 0.02173913 | 0.02040816 | 0.032786880000000004 | 0.037500000000000006 | 0.02884615 | 0.04255319 | 0.03529412 | 0.02439024 | 0.02912621 | 0.01098901 | 0.024 |
| 0.56000000000000005 | 0.0 | 0.008547009000000003 | 0.025 | 0.03370786 | 0.01219512 | 0.0106383 | 0.01449275 | 0.010000000000000004 | 0.0 | 0.010869570000000007 | 0.0 | 0.032786880000000004 | 0.025 | 0.009615385 | 0.07446808000000003 | 0.023529409999999994 | 0.07317073 | 0.05825243000000002 | 0.01098901 | 0.024 |
| 0.58000000000000007 | 0.08695652000000004 | 0.0 | 0.025 | 0.01123596 | 0.02439024 | 0.0 | 0.0 | 0.005000000000000002 | 0.0 | 0.0 | 0.02040816 | 0.016393440000000002 | 0.0 | 0.0 | 0.053191490000000015 | 0.0 | 0.06097561000000001 | 0.05825243000000002 | 0.005494506 | 0.0 |
| 0.6000000000000002 | 0.0 | 0.0 | 0.025 | 0.04494382 | 0.01219512 | 0.0 | 0.0 | 0.0 | 0.0 | 0.02173913 | 0.0 | 0.0 | 0.0 | 0.009615385 | 0.021276600000000007 | 0.023529409999999994 | 0.01219512 | 0.048543690000000014 | 0.005494506 | 0.016000000000000007 |
| 0.62000000000000022 | 0.0 | 0.0 | 0.0 | 0.0 | 0.006097561 | 0.0 | 0.0 | 0.0 | 0.0 | 0.010869570000000007 | 0.0 | 0.016393440000000002 | 0.0125 | 0.0 | 0.021276600000000007 | 0.05882353 | 0.0 | 0.009708738000000001 | 0.0 | 0.0 |
| 0.64000000000000024 | 0.0 | 0.0 | 0.0 | 0.0 | 0.006097561 | 0.0 | 0.0 | 0.005000000000000002 | 0.0 | 0.010869570000000007 | 0.02040816 | 0.016393440000000002 | 0.0125 | 0.0 | 0.021276600000000007 | 0.011764710000000001 | 0.03658536000000001 | 0.009708738000000001 | 0.01098901 | 0.0 |
| 0.66000000000000025 | 0.0 | 0.01709402 | 0.0 | 0.01123596 | 0.006097561 | 0.0 | 0.0 | 0.005000000000000002 | 0.0 | 0.0 | 0.0 | 0.016393440000000002 | 0.0125 | 0.009615385 | 0.0106383 | 0.023529409999999994 | 0.03658536000000001 | 0.01941748 | 0.0 | 0.008000000000000005 |
| 0.68 | 0.0 | 0.0 | 0.0 | 0.0 | 0.0 | 0.0 | 0.0 | 0.0 | 0.0 | 0.0 | 0.02040816 | 0.032786880000000004 | 0.0 | 0.0 | 0.0 | 0.0 | 0.03658536000000001 | 0.009708738000000001 | 0.005494506 | 0.008000000000000005 |
| 0.70000000000000018 | 0.0 | 0.0 | 0.0 | 0.0 | 0.0 | 0.0 | 0.0 | 0.0 | 0.0 | 0.0 | 0.0 | 0.0 | 0.0 | 0.009615385 | 0.0 | 0.0 | 0.02439024 | 0.009708738000000001 | 0.01098901 | 0.0 |
### Chart: tph-1 A3
| Category | tph-1 A3 No.1 | tph-1 A3 No.2 | tph-1 A3 No.3 | tph-1 A3 No.4 | tph-1 A3 No.5 | tph-1 A3 No.6 | tph-1 A3 No.7 | tph-1 A3 No.8 | tph-1 A3 No.9 | tph-1 A3 No.10 | tph-1 A3 No.11 | tph-1 A3 No.12 | tph-1 A3 No.13 | tph-1 A3 No.14 | tph-1 A3 No.15 | tph-1 A3 No.16 | tph-1 A3 No.17 | tph-1 A3 No.18 |
|---|---|---|---|---|---|---|---|---|---|---|---|---|---|---|---|---|---|---|
| 0 | None | None | None | None | None | None | None | None | None | None | None | None | None | None | None | None | None | None |
| 2.0000000000000007E-2 | 0.009345794000000004 | 0.0 | 0.0 | 0.0 | 0.04255319 | 0.0 | 0.0 | 0.0 | 0.05454545 | 0.01769911 | 0.013793100000000004 | 0.0 | 0.01574803 | 0.014925370000000004 | 0.0 | 0.0 | 0.02380952 | 0.006849315 |
| 4.0000000000000015E-2 | 0.01869159 | 0.07894737 | 0.0 | 0.009803922000000003 | 0.021276600000000007 | 0.004273504000000002 | 0.0 | 0.02631579 | 0.018181820000000008 | 0.0 | 0.0 | 0.0 | 0.03149606 | 0.014925370000000004 | 0.0 | 0.08823530000000006 | 0.0 | 0.0 |
| 6.0000000000000019E-2 | 0.009345794000000004 | 0.01315789 | 0.01234568 | 0.02941176 | 0.021276600000000007 | 0.0 | 0.0 | 0.01315789 | 0.05454545 | 0.02654867 | 0.02068966 | 0.0 | 0.01574803 | 0.044776120000000016 | 0.005649718 | 0.0 | 0.015873020000000005 | 0.0 |
| 8.0000000000000029E-2 | 0.028037380000000008 | 0.02631579 | 0.01234568 | 0.05882353 | 0.0 | 0.0 | 0.0 | 0.0 | 0.09090909000000003 | 0.008849557000000006 | 0.0 | 0.007575758000000004 | 0.03149606 | 0.014925370000000004 | 0.005649718 | 0.0 | 0.015873020000000005 | 0.0 |
| 0.1 | 0.009345794000000004 | 0.01315789 | 0.0 | 0.0 | 0.0 | 0.004273504000000002 | 0.0 | 0.01315789 | 0.018181820000000008 | 0.02654867 | 0.0 | 0.0 | 0.02362205 | 0.0 | 0.01129944 | 0.05882353 | 0.0 | 0.01369863 |
| 0.12000000000000002 | 0.009345794000000004 | 0.0 | 0.0 | 0.01960784 | 0.021276600000000007 | 0.004273504000000002 | 0.0 | 0.0 | 0.018181820000000008 | 0.02654867 | 0.013793100000000004 | 0.007575758000000004 | 0.0 | 0.0 | 0.0 | 0.02941176 | 0.02380952 | 0.006849315 |
| 0.14000000000000001 | 0.0 | 0.02631579 | 0.02469136 | 0.01960784 | 0.0 | 0.008547009000000003 | 0.0 | 0.0 | 0.018181820000000008 | 0.008849557000000006 | 0.006896552000000002 | 0.0 | 0.02362205 | 0.014925370000000004 | 0.0 | 0.0 | 0.015873020000000005 | 0.0 |
| 0.16 | 0.009345794000000004 | 0.03947368000000001 | 0.0 | 0.009803922000000003 | 0.021276600000000007 | 0.004273504000000002 | 0.0 | 0.0 | 0.0 | 0.01769911 | 0.006896552000000002 | 0.007575758000000004 | 0.007874016 | 0.0 | 0.01129944 | 0.0 | 0.015873020000000005 | 0.0 |
| 0.18000000000000005 | 0.009345794000000004 | 0.05263158 | 0.0 | 0.0 | 0.0 | 0.012820510000000004 | 0.0 | 0.01315789 | 0.03636364000000001 | 0.03539823 | 0.006896552000000002 | 0.0 | 0.0 | 0.0 | 0.022598870000000007 | 0.0 | 0.007936508000000004 | 0.006849315 |
| 0.2 | 0.04672897 | 0.02631579 | 0.0 | 0.01960784 | 0.0 | 0.004273504000000002 | 0.0 | 0.01315789 | 0.018181820000000008 | 0.01769911 | 0.0 | 0.007575758000000004 | 0.007874016 | 0.014925370000000004 | 0.005649718 | 0.02941176 | 0.007936508000000004 | 0.006849315 |
| 0.22 | 0.028037380000000008 | 0.02631579 | 0.01234568 | 0.02941176 | 0.0 | 0.004273504000000002 | 0.0 | 0.0 | 0.0 | 0.008849557000000006 | 0.027586210000000014 | 0.0 | 0.01574803 | 0.05970149000000004 | 0.016949150000000007 | 0.02941176 | 0.0 | 0.006849315 |
| 0.24000000000000005 | 0.01869159 | 0.06578948 | 0.0 | 0.02941176 | 0.0 | 0.01709402 | 0.0 | 0.0 | 0.018181820000000008 | 0.02654867 | 0.04827585999999996 | 0.015151520000000005 | 0.03937008 | 0.014925370000000004 | 0.016949150000000007 | 0.02941176 | 0.02380952 | 0.006849315 |
| 0.26 | 0.04672897 | 0.05263158 | 0.01234568 | 0.05882353 | 0.021276600000000007 | 0.025641030000000006 | 0.0 | 0.0 | 0.018181820000000008 | 0.07964602 | 0.027586210000000014 | 0.03030303 | 0.06299213 | 0.0 | 0.01129944 | 0.0 | 0.02380952 | 0.0 |
| 0.28000000000000008 | 0.04672897 | 0.06578948 | 0.01234568 | 0.03921569 | 0.021276600000000007 | 0.05555555999999998 | 0.0 | 0.0 | 0.05454545 | 0.01769911 | 0.027586210000000014 | 0.03030303 | 0.04724409 | 0.07462686 | 0.03954802 | 0.0 | 0.03174603 | 0.020547950000000002 |
| 0.3000000000000001 | 0.06542056 | 0.09210526000000001 | 0.0 | 0.05882353 | 0.0 | 0.07692308000000002 | 0.04761905 | 0.02631579 | 0.0 | 0.08849557000000005 | 0.06206897000000003 | 0.03030303 | 0.07874016 | 0.05970149000000004 | 0.06214689 | 0.0 | 0.05555555999999998 | 0.07534247000000002 |
| 0.32000000000000012 | 0.05607476 | 0.03947368000000001 | 0.01234568 | 0.05882353 | 0.021276600000000007 | 0.0982906 | 0.0 | 0.03947368000000001 | 0.018181820000000008 | 0.07964602 | 0.08275862 | 0.015151520000000005 | 0.09448819 | 0.1044776 | 0.07344633 | 0.05882353 | 0.03174603 | 0.04109589 |
| 0.34 | 0.06542056 | 0.03947368000000001 | 0.02469136 | 0.07843138 | 0.0 | 0.0940171 | 0.03571429 | 0.0 | 0.05454545 | 0.10619470000000006 | 0.1034483 | 0.06818182 | 0.07086615 | 0.05970149000000004 | 0.09604520000000002 | 0.05882353 | 0.06349207000000003 | 0.09589041000000002 |
| 0.3600000000000001 | 0.05607476 | 0.03947368000000001 | 0.01234568 | 0.05882353 | 0.10638300000000002 | 0.0940171 | 0.07142857 | 0.01315789 | 0.0 | 0.07964602 | 0.06896552 | 0.04545455 | 0.02362205 | 0.07462686 | 0.11864410000000003 | 0.02941176 | 0.05555555999999998 | 0.07534247000000002 |
| 0.38000000000000012 | 0.04672897 | 0.06578948 | 0.01234568 | 0.07843138 | 0.021276600000000007 | 0.08119658 | 0.1428571 | 0.01315789 | 0.05454545 | 0.10619470000000006 | 0.027586210000000014 | 0.0530303 | 0.09448819 | 0.05970149000000004 | 0.10169490000000003 | 0.0 | 0.03174603 | 0.130137 |
| 0.4 | 0.03738318 | 0.07894737 | 0.049382720000000026 | 0.049019610000000026 | 0.06382979 | 0.06837607 | 0.0952381 | 0.02631579 | 0.03636364000000001 | 0.02654867 | 0.08275862 | 0.0530303 | 0.007874016 | 0.044776120000000016 | 0.07344633 | 0.0 | 0.02380952 | 0.10273970000000003 |
| 0.4200000000000001 | 0.07476635000000002 | 0.05263158 | 0.049382720000000026 | 0.049019610000000026 | 0.14893620000000007 | 0.06410257 | 0.15476190000000006 | 0.0 | 0.09090909000000003 | 0.04424779 | 0.1103448 | 0.0530303 | 0.03937008 | 0.044776120000000016 | 0.11864410000000003 | 0.02941176 | 0.03968254 | 0.1506849 |
| 0.44 | 0.028037380000000008 | 0.01315789 | 0.02469136 | 0.06862745 | 0.08510638000000002 | 0.05128205 | 0.02380952 | 0.01315789 | 0.03636364000000001 | 0.03539823 | 0.03448276 | 0.06818182 | 0.06299213 | 0.044776120000000016 | 0.08474576000000003 | 0.05882353 | 0.05555555999999998 | 0.07534247000000002 |
| 0.46 | 0.009345794000000004 | 0.02631579 | 0.0617284 | 0.049019610000000026 | 0.08510638000000002 | 0.05982906 | 0.07142857 | 0.03947368000000001 | 0.05454545 | 0.03539823 | 0.06896552 | 0.06060606 | 0.03937008 | 0.02985075 | 0.045197740000000014 | 0.0 | 0.03968254 | 0.020547950000000002 |
| 0.48000000000000009 | 0.028037380000000008 | 0.03947368000000001 | 0.09876543000000006 | 0.01960784 | 0.08510638000000002 | 0.029914529999999995 | 0.15476190000000006 | 0.09210526000000001 | 0.018181820000000008 | 0.01769911 | 0.07586207000000003 | 0.09848485000000007 | 0.007874016 | 0.0 | 0.01129944 | 0.05882353 | 0.05555555999999998 | 0.01369863 |
| 0.5 | 0.04672897 | 0.01315789 | 0.1111111 | 0.009803922000000003 | 0.04255319 | 0.008547009000000003 | 0.05952381 | 0.01315789 | 0.018181820000000008 | 0.02654867 | 0.04137930999999999 | 0.09848485000000007 | 0.03937008 | 0.014925370000000004 | 0.02824859 | 0.02941176 | 0.03968254 | 0.04794521 |
| 0.52 | 0.03738318 | 0.0 | 0.049382720000000026 | 0.0 | 0.04255319 | 0.008547009000000003 | 0.011904760000000004 | 0.0 | 0.03636364000000001 | 0.0 | 0.0 | 0.04545455 | 0.03149606 | 0.014925370000000004 | 0.0 | 0.05882353 | 0.007936508000000004 | 0.01369863 |
| 0.54 | 0.01869159 | 0.01315789 | 0.02469136 | 0.02941176 | 0.06382979 | 0.008547009000000003 | 0.02380952 | 0.05263158 | 0.018181820000000008 | 0.02654867 | 0.027586210000000014 | 0.015151520000000005 | 0.01574803 | 0.0 | 0.022598870000000007 | 0.1764706 | 0.03174603 | 0.006849315 |
| 0.56000000000000005 | 0.028037380000000008 | 0.0 | 0.09876543000000006 | 0.01960784 | 0.04255319 | 0.03418804 | 0.04761905 | 0.06578948 | 0.05454545 | 0.0 | 0.006896552000000002 | 0.04545455 | 0.01574803 | 0.05970149000000004 | 0.01129944 | 0.0 | 0.05555555999999998 | 0.020547950000000002 |
| 0.58000000000000007 | 0.01869159 | 0.0 | 0.03703704000000002 | 0.02941176 | 0.0 | 0.008547009000000003 | 0.011904760000000004 | 0.10526320000000006 | 0.018181820000000008 | 0.0 | 0.0 | 0.0530303 | 0.01574803 | 0.0 | 0.005649718 | 0.05882353 | 0.015873020000000005 | 0.006849315 |
| 0.6000000000000002 | 0.01869159 | 0.0 | 0.09876543000000006 | 0.009803922000000003 | 0.0 | 0.008547009000000003 | 0.02380952 | 0.10526320000000006 | 0.0 | 0.0 | 0.006896552000000002 | 0.015151520000000005 | 0.0 | 0.02985075 | 0.0 | 0.02941176 | 0.015873020000000005 | 0.0 |
| 0.62000000000000022 | 0.01869159 | 0.0 | 0.01234568 | 0.0 | 0.0 | 0.008547009000000003 | 0.0 | 0.03947368000000001 | 0.018181820000000008 | 0.008849557000000006 | 0.0 | 0.022727270000000008 | 0.007874016 | 0.014925370000000004 | 0.0 | 0.0 | 0.03174603 | 0.006849315 |
| 0.64000000000000024 | 0.009345794000000004 | 0.0 | 0.03703704000000002 | 0.0 | 0.0 | 0.008547009000000003 | 0.0 | 0.07894737 | 0.0 | 0.0 | 0.0 | 0.007575758000000004 | 0.007874016 | 0.014925370000000004 | 0.0 | 0.0 | 0.02380952 | 0.0 |
| 0.66000000000000025 | 0.028037380000000008 | 0.0 | 0.02469136 | 0.009803922000000003 | 0.0 | 0.008547009000000003 | 0.0 | 0.05263158 | 0.0 | 0.0 | 0.0 | 0.0 | 0.0 | 0.0 | 0.0 | 0.0 | 0.03968254 | 0.01369863 |
| 0.68 | 0.0 | 0.0 | 0.01234568 | 0.0 | 0.0 | 0.008547009000000003 | 0.0 | 0.03947368000000001 | 0.0 | 0.0 | 0.0 | 0.015151520000000005 | 0.0 | 0.0 | 0.0 | 0.0 | 0.03174603 | 0.0 |
| 0.70000000000000018 | 0.009345794000000004 | 0.0 | 0.01234568 | 0.0 | 0.0 | 0.004273504000000002 | 0.0 | 0.02631579 | 0.0 | 0.0 | 0.0 | 0.0 | 0.0 | 0.044776120000000016 | 0.0 | 0.02941176 | 0.007936508000000004 | 0.006849315 |
### Chart: tph-1 A5
| Category | tph-1 A5 No.1 | tph-1 A5 No.2 | tph-1 A5 No.3 | tph-1 A5 No.4 | tph-1 A5 No.5 | tph-1 A5 No.6 | tph-1 A5 No.7 | tph-1 A5 No.8 | tph-1 A5 No.9 | tph-1 A5 No.10 | tph-1 A5 No.11 | tph-1 A5 No.12 |
|---|---|---|---|---|---|---|---|---|---|---|---|---|
| 0 | None | None | None | None | None | None | None | None | None | None | None | None |
| 2.0000000000000007E-2 | 0.07692308000000002 | 0.0 | 0.018181820000000008 | 0.02985075 | 0.05714285999999998 | 0.0625 | 0.06666667 | 0.018518520000000007 | 0.01960784 | 0.03225806 | 0.03125 | 0.0 |
| 4.0000000000000015E-2 | 0.07692308000000002 | 0.0 | 0.018181820000000008 | 0.07462686 | 0.0 | 0.0625 | 0.2666667000000001 | 0.0 | 0.009803922000000003 | 0.03225806 | 0.0 | 0.01574803 |
| 6.0000000000000019E-2 | 0.15384620000000007 | 0.03846154 | 0.03636364000000001 | 0.02985075 | 0.0 | 0.0 | 0.2666667000000001 | 0.0 | 0.01960784 | 0.0 | 0.09375000000000004 | 0.007874016 |
| 8.0000000000000029E-2 | 0.07692308000000002 | 0.03846154 | 0.0 | 0.014925370000000004 | 0.02857142999999999 | 0.0 | 0.0 | 0.0 | 0.009803922000000003 | 0.016129030000000003 | 0.0 | 0.0 |
| 0.1 | 0.0 | 0.0 | 0.0 | 0.014925370000000004 | 0.0 | 0.0 | 0.0 | 0.0 | 0.009803922000000003 | 0.0 | 0.0 | 0.007874016 |
| 0.12000000000000002 | 0.0 | 0.0 | 0.0 | 0.0 | 0.0 | 0.0625 | 0.06666667 | 0.0 | 0.009803922000000003 | 0.0 | 0.0 | 0.007874016 |
| 0.14000000000000001 | 0.07692308000000002 | 0.0 | 0.018181820000000008 | 0.0 | 0.0 | 0.0 | 0.0 | 0.018518520000000007 | 0.009803922000000003 | 0.016129030000000003 | 0.03125 | 0.0 |
| 0.16 | 0.0 | 0.03846154 | 0.05454545 | 0.014925370000000004 | 0.0 | 0.0 | 0.06666667 | 0.0 | 0.0 | 0.016129030000000003 | 0.0 | 0.007874016 |
| 0.18000000000000005 | 0.0 | 0.0 | 0.018181820000000008 | 0.044776120000000016 | 0.0 | 0.0 | 0.0 | 0.0 | 0.02941176 | 0.0 | 0.0 | 0.01574803 |
| 0.2 | 0.07692308000000002 | 0.0 | 0.05454545 | 0.0 | 0.02857142999999999 | 0.0 | 0.0 | 0.0 | 0.01960784 | 0.0 | 0.0 | 0.007874016 |
| 0.22 | 0.0 | 0.03846154 | 0.0 | 0.0 | 0.0 | 0.0625 | 0.0 | 0.0 | 0.02941176 | 0.0 | 0.03125 | 0.01574803 |
| 0.24000000000000005 | 0.07692308000000002 | 0.0 | 0.018181820000000008 | 0.0 | 0.02857142999999999 | 0.0 | 0.06666667 | 0.0 | 0.009803922000000003 | 0.0 | 0.03125 | 0.0 |
| 0.26 | 0.0 | 0.0 | 0.0 | 0.014925370000000004 | 0.1428571 | 0.0 | 0.0 | 0.03703704000000002 | 0.03921569 | 0.016129030000000003 | 0.0 | 0.02362205 |
| 0.28000000000000008 | 0.0 | 0.03846154 | 0.0 | 0.014925370000000004 | 0.1714286 | 0.0 | 0.0 | 0.0 | 0.02941176 | 0.0 | 0.0 | 0.10236220000000003 |
| 0.3000000000000001 | 0.0 | 0.03846154 | 0.018181820000000008 | 0.044776120000000016 | 0.0 | 0.0625 | 0.0 | 0.018518520000000007 | 0.127451 | 0.0483871 | 0.0 | 0.07874016 |
| 0.32000000000000012 | 0.0 | 0.03846154 | 0.0 | 0.014925370000000004 | 0.08571429000000003 | 0.0625 | 0.0 | 0.05555555999999998 | 0.05882353 | 0.03225806 | 0.0 | 0.07086615 |
| 0.34 | 0.0 | 0.0 | 0.018181820000000008 | 0.044776120000000016 | 0.02857142999999999 | 0.125 | 0.06666667 | 0.018518520000000007 | 0.1666667 | 0.0483871 | 0.0 | 0.08661418000000004 |
| 0.3600000000000001 | 0.07692308000000002 | 0.03846154 | 0.018181820000000008 | 0.0 | 0.1428571 | 0.125 | 0.0 | 0.09259259000000003 | 0.03921569 | 0.03225806 | 0.03125 | 0.07086615 |
| 0.38000000000000012 | 0.07692308000000002 | 0.03846154 | 0.03636364000000001 | 0.05970149000000004 | 0.02857142999999999 | 0.0 | 0.06666667 | 0.05555555999999998 | 0.06862745 | 0.0483871 | 0.125 | 0.10236220000000003 |
| 0.4 | 0.0 | 0.0 | 0.03636364000000001 | 0.1343284 | 0.05714285999999998 | 0.0625 | 0.0 | 0.07407407000000003 | 0.08823530000000006 | 0.06451613000000003 | 0.0 | 0.05511811 |
| 0.4200000000000001 | 0.07692308000000002 | 0.03846154 | 0.03636364000000001 | 0.08955224000000006 | 0.11428570000000003 | 0.0 | 0.0 | 0.05555555999999998 | 0.03921569 | 0.08064516000000004 | 0.03125 | 0.04724409 |
| 0.44 | 0.15384620000000007 | 0.03846154 | 0.018181820000000008 | 0.02985075 | 0.02857142999999999 | 0.0 | 0.0 | 0.03703704000000002 | 0.02941176 | 0.1290323 | 0.0 | 0.05511811 |
| 0.46 | 0.0 | 0.07692308000000002 | 0.05454545 | 0.07462686 | 0.0 | 0.0 | 0.0 | 0.09259259000000003 | 0.02941176 | 0.08064516000000004 | 0.15625000000000006 | 0.06299213 |
| 0.48000000000000009 | 0.0 | 0.07692308000000002 | 0.05454545 | 0.014925370000000004 | 0.0 | 0.0 | 0.0 | 0.07407407000000003 | 0.01960784 | 0.0483871 | 0.0 | 0.01574803 |
| 0.5 | 0.0 | 0.1153846 | 0.14545450000000001 | 0.014925370000000004 | 0.02857142999999999 | 0.0 | 0.0 | 0.07407407000000003 | 0.02941176 | 0.0483871 | 0.03125 | 0.05511811 |
| 0.52 | 0.0 | 0.15384620000000007 | 0.018181820000000008 | 0.044776120000000016 | 0.02857142999999999 | 0.0625 | 0.06666667 | 0.05555555999999998 | 0.009803922000000003 | 0.06451613000000003 | 0.09375000000000004 | 0.03149606 |
| 0.54 | 0.0 | 0.03846154 | 0.018181820000000008 | 0.044776120000000016 | 0.0 | 0.125 | 0.0 | 0.05555555999999998 | 0.01960784 | 0.06451613000000003 | 0.03125 | 0.007874016 |
| 0.56000000000000005 | 0.0 | 0.0 | 0.03636364000000001 | 0.014925370000000004 | 0.0 | 0.0625 | 0.0 | 0.0 | 0.0 | 0.0 | 0.03125 | 0.02362205 |
| 0.58000000000000007 | 0.0 | 0.0 | 0.05454545 | 0.02985075 | 0.0 | 0.0 | 0.0 | 0.03703704000000002 | 0.009803922000000003 | 0.016129030000000003 | 0.0625 | 0.0 |
| 0.6000000000000002 | 0.0 | 0.0 | 0.05454545 | 0.02985075 | 0.0 | 0.0 | 0.0 | 0.0 | 0.0 | 0.0 | 0.0 | 0.0 |
| 0.62000000000000022 | 0.0 | 0.07692308000000002 | 0.03636364000000001 | 0.02985075 | 0.0 | 0.0 | 0.0 | 0.03703704000000002 | 0.0 | 0.0 | 0.03125 | 0.0 |
| 0.64000000000000024 | 0.0 | 0.0 | 0.03636364000000001 | 0.014925370000000004 | 0.0 | 0.0625 | 0.0 | 0.018518520000000007 | 0.0 | 0.0 | 0.03125 | 0.007874016 |
| 0.66000000000000025 | 0.0 | 0.03846154 | 0.03636364000000001 | 0.0 | 0.0 | 0.0 | 0.0 | 0.018518520000000007 | 0.009803922000000003 | 0.03225806 | 0.03125 | 0.0 |
| 0.68 | 0.0 | 0.0 | 0.0 | 0.0 | 0.0 | 0.0 | 0.0 | 0.0 | 0.0 | 0.0 | 0.03125 | 0.007874016 |
| 0.70000000000000018 | 0.0 | 0.0 | 0.0 | 0.014925370000000004 | 0.0 | 0.0 | 0.0 | 0.0 | 0.0 | 0.016129030000000003 | 0.03125 | 0.0 |
### Chart: cat-1 A1
| Category | cat-1 A1 No.1 | cat-1 A1 No.2 | cat-1 A1 No.3 | cat-1 A1 No.4 | cat-1 A1 No.5 | cat-1 A1 No.6 | cat-1 A1 No.7 | cat-1 A1 No.8 | cat-1 A1 No.9 | cat-1 A1 No.10 | cat-1 A1 No.11 | cat-1 A1 No.12 | cat-1 A1 No.13 | cat-1 A1 No.14 | cat-1 A1 No.15 | cat-1 A1 No.16 | cat-1 A1 No.17 | cat-1 A1 No.18 | cat-1 A1 No.19 |
|---|---|---|---|---|---|---|---|---|---|---|---|---|---|---|---|---|---|---|---|
| 0 | None | None | None | None | None | None | None | None | None | None | None | None | None | None | None | None | None | None | None |
| 2.0000000000000007E-2 | 0.02816901 | 0.056497180000000015 | 0.05555555999999998 | 0.020833330000000018 | 0.01369863 | 0.0 | 0.02678571000000001 | 0.0 | 0.02484472 | 0.006896552000000002 | 0.011450380000000001 | 0.005555556 | 0.0 | 0.0 | 0.0 | 0.0 | 0.0 | 0.0 | 0.009478673 |
| 4.0000000000000015E-2 | 0.07042254000000002 | 0.02824859 | 0.0 | 0.020833330000000018 | 0.03424658 | 0.0 | 0.05357143 | 0.0 | 0.01863354 | 0.006896552000000002 | 0.003816794000000001 | 0.01666667 | 0.0 | 0.0 | 0.0 | 0.0 | 0.0 | 0.0 | 0.0 |
| 6.0000000000000019E-2 | 0.07042254000000002 | 0.02824859 | 0.0 | 0.0 | 0.006849315 | 0.003968254000000002 | 0.01785714 | 0.0 | 0.01242236 | 0.0 | 0.011450380000000001 | 0.01111111 | 0.0 | 0.0 | 0.0 | 0.0 | 0.0 | 0.0 | 0.0 |
| 8.0000000000000029E-2 | 0.0 | 0.01129944 | 0.0 | 0.04166667000000002 | 0.01369863 | 0.0 | 0.02678571000000001 | 0.0 | 0.01863354 | 0.013793100000000004 | 0.0 | 0.005555556 | 0.0 | 0.0 | 0.0 | 0.0 | 0.0 | 0.0 | 0.0 |
| 0.1 | 0.04225352 | 0.022598870000000007 | 0.0 | 0.0 | 0.0 | 0.0 | 0.008928572000000003 | 0.0 | 0.0 | 0.0 | 0.0 | 0.0 | 0.0 | 0.0 | 0.0 | 0.0 | 0.0 | 0.0 | 0.0 |
| 0.12000000000000002 | 0.02816901 | 0.01129944 | 0.02777778000000001 | 0.0 | 0.0 | 0.007936508000000004 | 0.02678571000000001 | 0.0 | 0.01242236 | 0.006896552000000002 | 0.0 | 0.0 | 0.0 | 0.0 | 0.0 | 0.0 | 0.0 | 0.0036231880000000016 | 0.0 |
| 0.14000000000000001 | 0.0 | 0.005649718 | 0.0 | 0.0 | 0.0 | 0.003968254000000002 | 0.0 | 0.0 | 0.006211180000000002 | 0.006896552000000002 | 0.003816794000000001 | 0.0 | 0.0 | 0.0 | 0.0 | 0.0 | 0.005813953000000002 | 0.0 | 0.0 |
| 0.16 | 0.01408451 | 0.02824859 | 0.0 | 0.04166667000000002 | 0.0 | 0.003968254000000002 | 0.02678571000000001 | 0.009708738000000001 | 0.01242236 | 0.0 | 0.015267180000000003 | 0.0 | 0.0 | 0.004854369 | 0.0078125 | 0.0 | 0.0 | 0.0 | 0.004739337000000002 |
| 0.18000000000000005 | 0.0 | 0.045197740000000014 | 0.0 | 0.020833330000000018 | 0.0 | 0.02380952 | 0.0 | 0.0 | 0.006211180000000002 | 0.0 | 0.007633588000000002 | 0.0 | 0.0 | 0.009708738000000001 | 0.023437500000000007 | 0.0 | 0.005813953000000002 | 0.010869570000000007 | 0.0 |
| 0.2 | 0.01408451 | 0.022598870000000007 | 0.0 | 0.0 | 0.020547950000000002 | 0.015873020000000005 | 0.03571429 | 0.009708738000000001 | 0.0 | 0.027586210000000014 | 0.02671756 | 0.01666667 | 0.0 | 0.01941748 | 0.015625 | 0.007462686000000002 | 0.0 | 0.01449275 | 0.009478673 |
| 0.22 | 0.05633803000000001 | 0.03954802 | 0.0 | 0.0 | 0.02739726000000001 | 0.04365079 | 0.044642859999999986 | 0.01941748 | 0.01863354 | 0.0 | 0.04198473 | 0.005555556 | 0.005319149000000001 | 0.009708738000000001 | 0.0390625 | 0.014925370000000004 | 0.005813953000000002 | 0.0326087 | 0.0 |
| 0.24000000000000005 | 0.01408451 | 0.06214689 | 0.02777778000000001 | 0.08333334000000003 | 0.06849315 | 0.03571429 | 0.03571429 | 0.02912621 | 0.031055900000000015 | 0.03448276 | 0.03816794 | 0.02222222 | 0.01595745 | 0.02912621 | 0.03125 | 0.007462686000000002 | 0.034883720000000014 | 0.06884058 | 0.02843602 |
| 0.26 | 0.04225352 | 0.06214689 | 0.02777778000000001 | 0.0625 | 0.09589041000000002 | 0.05952381 | 0.10714290000000003 | 0.043689319999999976 | 0.08695652000000004 | 0.06206897000000003 | 0.07633588 | 0.03333334000000002 | 0.02659575 | 0.09708738 | 0.046874999999999986 | 0.02238806 | 0.02906977 | 0.05434782 | 0.04265403 |
| 0.28000000000000008 | 0.05633803000000001 | 0.10169490000000003 | 0.08333334000000003 | 0.08333334000000003 | 0.10273970000000003 | 0.0952381 | 0.05357143 | 0.10679610000000006 | 0.09937888 | 0.09655172000000001 | 0.06488550000000001 | 0.07222223 | 0.058510640000000017 | 0.10194170000000002 | 0.078125 | 0.09701493000000004 | 0.07558139 | 0.11231879999999997 | 0.06161136999999998 |
| 0.3000000000000001 | 0.09859155000000004 | 0.08474576000000003 | 0.08333334000000003 | 0.2291667 | 0.10958900000000002 | 0.1428571 | 0.08928572000000001 | 0.17475730000000006 | 0.14906830000000004 | 0.1034483 | 0.10687020000000003 | 0.11666670000000005 | 0.11170210000000003 | 0.1262136 | 0.03125 | 0.14179100000000006 | 0.09883721000000002 | 0.1268116 | 0.04265403 |
| 0.32000000000000012 | 0.11267610000000003 | 0.06779661000000003 | 0.1111111 | 0.10416670000000006 | 0.130137 | 0.1190476 | 0.09821428 | 0.1601942 | 0.1118012 | 0.07586207000000003 | 0.08778626 | 0.1333333 | 0.06382979 | 0.09708738 | 0.1328125 | 0.18656720000000007 | 0.14534880000000006 | 0.10507250000000003 | 0.12796210000000005 |
| 0.34 | 0.05633803000000001 | 0.056497180000000015 | 0.3333333000000001 | 0.10416670000000006 | 0.13698630000000006 | 0.12301590000000003 | 0.0625 | 0.1796117 | 0.1180124 | 0.08275862 | 0.08778626 | 0.1388889 | 0.11170210000000003 | 0.10194170000000002 | 0.14843750000000006 | 0.11940300000000002 | 0.17441860000000006 | 0.09057971 | 0.12796210000000005 |
| 0.3600000000000001 | 0.04225352 | 0.056497180000000015 | 0.08333334000000003 | 0.08333334000000003 | 0.06849315 | 0.09126984000000005 | 0.08035714000000001 | 0.10679610000000006 | 0.1180124 | 0.12413790000000002 | 0.09541985 | 0.1333333 | 0.12765959999999996 | 0.1262136 | 0.09375000000000004 | 0.1119403 | 0.09883721000000002 | 0.06521739000000001 | 0.10900470000000004 |
| 0.38000000000000012 | 0.02816901 | 0.03954802 | 0.02777778000000001 | 0.0625 | 0.061643839999999984 | 0.07142857 | 0.044642859999999986 | 0.07281554000000001 | 0.0621118 | 0.08275862 | 0.07633588 | 0.06666667 | 0.09574468000000004 | 0.08252427000000005 | 0.1640625 | 0.08208955000000001 | 0.0872093 | 0.08695652000000004 | 0.13270140000000005 |
| 0.4 | 0.1267606 | 0.06214689 | 0.05555555999999998 | 0.020833330000000018 | 0.04794521 | 0.06349207000000003 | 0.03571429 | 0.03398058 | 0.031055900000000015 | 0.02068966 | 0.09160306000000003 | 0.08333334000000003 | 0.1329787 | 0.08737864 | 0.054687500000000014 | 0.08955224000000006 | 0.07558139 | 0.050724640000000015 | 0.07109005 |
| 0.4200000000000001 | 0.04225352 | 0.022598870000000007 | 0.02777778000000001 | 0.020833330000000018 | 0.020547950000000002 | 0.04761905 | 0.01785714 | 0.01941748 | 0.03726708 | 0.06896552 | 0.06488550000000001 | 0.05 | 0.07978723000000004 | 0.05825243000000002 | 0.0390625 | 0.05223880999999998 | 0.058139539999999997 | 0.07608695000000001 | 0.08056872000000002 |
| 0.44 | 0.01408451 | 0.022598870000000007 | 0.0 | 0.0 | 0.0 | 0.02380952 | 0.02678571000000001 | 0.024271840000000013 | 0.01242236 | 0.03448276 | 0.05343511 | 0.03333334000000002 | 0.09042553 | 0.038834950000000014 | 0.0390625 | 0.03731343 | 0.05232558 | 0.04710145 | 0.07109005 |
| 0.46 | 0.01408451 | 0.022598870000000007 | 0.0 | 0.0 | 0.020547950000000002 | 0.007936508000000004 | 0.02678571000000001 | 0.0 | 0.0 | 0.06206897000000003 | 0.019083970000000006 | 0.03333334000000002 | 0.02659575 | 0.009708738000000001 | 0.023437500000000007 | 0.0 | 0.02906977 | 0.02898551 | 0.03317536 |
| 0.48000000000000009 | 0.01408451 | 0.005649718 | 0.02777778000000001 | 0.0 | 0.006849315 | 0.007936508000000004 | 0.01785714 | 0.0 | 0.0 | 0.013793100000000004 | 0.003816794000000001 | 0.005555556 | 0.02659575 | 0.0 | 0.03125 | 0.007462686000000002 | 0.01162791 | 0.01449275 | 0.014218009999999998 |
| 0.5 | 0.0 | 0.016949150000000007 | 0.02777778000000001 | 0.0 | 0.006849315 | 0.003968254000000002 | 0.0 | 0.004854369 | 0.01242236 | 0.02068966 | 0.003816794000000001 | 0.0 | 0.005319149000000001 | 0.0 | 0.0 | 0.0 | 0.0 | 0.0036231880000000016 | 0.004739337000000002 |
| 0.52 | 0.0 | 0.01129944 | 0.0 | 0.0 | 0.0 | 0.0 | 0.0 | 0.0 | 0.0 | 0.006896552000000002 | 0.007633588000000002 | 0.01111111 | 0.0106383 | 0.0 | 0.0 | 0.007462686000000002 | 0.01162791 | 0.0036231880000000016 | 0.009478673 |
| 0.54 | 0.0 | 0.0 | 0.0 | 0.0 | 0.0 | 0.0 | 0.008928572000000003 | 0.004854369 | 0.0 | 0.02068966 | 0.0 | 0.0 | 0.0 | 0.0 | 0.0 | 0.014925370000000004 | 0.0 | 0.0036231880000000016 | 0.004739337000000002 |
| 0.56000000000000005 | 0.0 | 0.0 | 0.0 | 0.0 | 0.0 | 0.0 | 0.008928572000000003 | 0.0 | 0.0 | 0.006896552000000002 | 0.0 | 0.005555556 | 0.0 | 0.0 | 0.0 | 0.0 | 0.0 | 0.0 | 0.004739337000000002 |
| 0.58000000000000007 | 0.0 | 0.0 | 0.0 | 0.0 | 0.006849315 | 0.0 | 0.0 | 0.0 | 0.0 | 0.0 | 0.0 | 0.0 | 0.0 | 0.0 | 0.0 | 0.0 | 0.0 | 0.0 | 0.004739337000000002 |
| 0.6000000000000002 | 0.0 | 0.0 | 0.0 | 0.0 | 0.0 | 0.0 | 0.0 | 0.0 | 0.0 | 0.0 | 0.0 | 0.0 | 0.0 | 0.0 | 0.0 | 0.0 | 0.0 | 0.0 | 0.004739337000000002 |
| 0.62000000000000022 | 0.0 | 0.005649718 | 0.0 | 0.0 | 0.0 | 0.0 | 0.0 | 0.0 | 0.0 | 0.0 | 0.0 | 0.0 | 0.0 | 0.0 | 0.0 | 0.0 | 0.0 | 0.0 | 0.0 |
| 0.64000000000000024 | 0.01408451 | 0.0 | 0.0 | 0.0 | 0.0 | 0.0 | 0.008928572000000003 | 0.0 | 0.0 | 0.006896552000000002 | 0.0 | 0.0 | 0.0 | 0.0 | 0.0 | 0.0 | 0.0 | 0.0 | 0.0 |
| 0.66000000000000025 | 0.0 | 0.0 | 0.0 | 0.0 | 0.0 | 0.0 | 0.0 | 0.0 | 0.0 | 0.006896552000000002 | 0.0 | 0.0 | 0.0 | 0.0 | 0.0 | 0.0 | 0.0 | 0.0 | 0.0 |
| 0.68 | 0.0 | 0.0 | 0.0 | 0.0 | 0.0 | 0.003968254000000002 | 0.0 | 0.0 | 0.0 | 0.0 | 0.0 | 0.0 | 0.0 | 0.0 | 0.0 | 0.0 | 0.0 | 0.0 | 0.0 |
| 0.70000000000000018 | 0.0 | 0.0 | 0.0 | 0.0 | 0.0 | 0.0 | 0.008928572000000003 | 0.0 | 0.0 | 0.0 | 0.0 | 0.0 | 0.005319149000000001 | 0.0 | 0.0 | 0.0 | 0.0 | 0.0 | 0.0 |
### Chart: cat-1 A3
| Category | cat-1 A3 No.1 | cat-1 A3 No.2 | cat-1 A3 No.3 | cat-1 A3 No.4 | cat-1 A3 No.5 | cat-1 A3 No.6 | cat-1 A3 No.7 | cat-1 A3 No.8 | cat-1 A3 No.9 | cat-1 A3 No.10 | cat-1 A3 No.11 | cat-1 A3 No.12 | cat-1 A3 No.13 | cat-1 A3 No.14 | cat-1 A3 No.15 | cat-1 A3 No.16 | cat-1 A3 No.17 | cat-1 A3 No.18 | cat-1 A3 No.19 | cat-1 A3 No.20 |
|---|---|---|---|---|---|---|---|---|---|---|---|---|---|---|---|---|---|---|---|---|
| 0 | None | None | None | None | None | None | None | None | None | None | None | None | None | None | None | None | None | None | None | None |
| 2.0000000000000007E-2 | 0.0 | 0.0 | 0.0 | 0.0 | 0.006250000000000002 | 0.0 | 0.006493506000000002 | 0.005681818 | 0.0 | 0.0 | 0.0 | 0.0 | 0.007142857 | 0.0 | 0.0 | 0.004651163000000002 | 0.0 | 0.004672897 | 0.0 | 0.0 |
| 4.0000000000000015E-2 | 0.0 | 0.011560690000000004 | 0.0 | 0.0 | 0.006250000000000002 | 0.0 | 0.006493506000000002 | 0.0 | 0.0 | 0.0 | 0.006211180000000002 | 0.0 | 0.0 | 0.008196721 | 0.0 | 0.0 | 0.0 | 0.0 | 0.0 | 0.0 |
| 6.0000000000000019E-2 | 0.0 | 0.0 | 0.0 | 0.0 | 0.006250000000000002 | 0.0 | 0.0 | 0.0 | 0.0 | 0.0 | 0.0 | 0.0 | 0.007142857 | 0.008196721 | 0.0 | 0.0 | 0.0 | 0.0 | 0.0 | 0.0 |
| 8.0000000000000029E-2 | 0.0 | 0.0 | 0.0 | 0.0 | 0.0125 | 0.0 | 0.0 | 0.0 | 0.0 | 0.0 | 0.0 | 0.0 | 0.007142857 | 0.008196721 | 0.0 | 0.0 | 0.0 | 0.0 | 0.0 | 0.0 |
| 0.1 | 0.0 | 0.0057803470000000025 | 0.0 | 0.0 | 0.0 | 0.0 | 0.0 | 0.0 | 0.0 | 0.0 | 0.0 | 0.0 | 0.0 | 0.0 | 0.0 | 0.004651163000000002 | 0.0 | 0.0 | 0.0 | 0.0 |
| 0.12000000000000002 | 0.0 | 0.0 | 0.0 | 0.0 | 0.006250000000000002 | 0.0 | 0.0 | 0.005681818 | 0.0 | 0.0 | 0.0 | 0.0 | 0.007142857 | 0.0 | 0.0 | 0.0 | 0.0 | 0.004672897 | 0.0 | 0.0 |
| 0.14000000000000001 | 0.0052083330000000025 | 0.0 | 0.0 | 0.0 | 0.0 | 0.0 | 0.0 | 0.0 | 0.0 | 0.0 | 0.0 | 0.0 | 0.0 | 0.0 | 0.0 | 0.0 | 0.0 | 0.0 | 0.0 | 0.0 |
| 0.16 | 0.0 | 0.0 | 0.0 | 0.0 | 0.0 | 0.0 | 0.0 | 0.0 | 0.0 | 0.0 | 0.006211180000000002 | 0.0 | 0.0 | 0.0 | 0.0 | 0.009302326000000007 | 0.0 | 0.004672897 | 0.0 | 0.0 |
| 0.18000000000000005 | 0.0052083330000000025 | 0.011560690000000004 | 0.0 | 0.0 | 0.0 | 0.0 | 0.0 | 0.005681818 | 0.0 | 0.0 | 0.01863354 | 0.004926108000000002 | 0.007142857 | 0.0 | 0.00456621 | 0.004651163000000002 | 0.013215860000000001 | 0.01869159 | 0.003759399000000002 | 0.0 |
| 0.2 | 0.03645833000000001 | 0.0057803470000000025 | 0.006024096 | 0.0 | 0.006250000000000002 | 0.0 | 0.01298701 | 0.005681818 | 0.0 | 0.0 | 0.01863354 | 0.01477833 | 0.0 | 0.016393440000000002 | 0.022831050000000012 | 0.004651163000000002 | 0.013215860000000001 | 0.014018689999999995 | 0.007518797000000002 | 0.0 |
| 0.22 | 0.046874999999999986 | 0.02890173 | 0.02409638 | 0.0 | 0.006250000000000002 | 0.0 | 0.01298701 | 0.005681818 | 0.0 | 0.0 | 0.02484472 | 0.04433497000000003 | 0.01428571 | 0.008196721 | 0.05022830999999998 | 0.06046512 | 0.04845814999999998 | 0.04205608000000002 | 0.01879699 | 0.0 |
| 0.24000000000000005 | 0.13541670000000006 | 0.05780347000000003 | 0.018072289999999998 | 0.005952381 | 0.03125 | 0.014925370000000004 | 0.006493506000000002 | 0.022727270000000008 | 0.02072539 | 0.013422820000000007 | 0.0621118 | 0.06403941000000003 | 0.05 | 0.016393440000000002 | 0.06392694 | 0.07441860000000004 | 0.08370044000000006 | 0.10747660000000005 | 0.02255639 | 0.005376344000000003 |
| 0.26 | 0.1145833 | 0.040462430000000035 | 0.09638554 | 0.03571429 | 0.04375 | 0.03731343 | 0.05194804999999998 | 0.02840909 | 0.02590674 | 0.026845640000000014 | 0.1180124 | 0.09359606000000008 | 0.05 | 0.05737705 | 0.1461187 | 0.10232560000000003 | 0.11894270000000003 | 0.13551400000000005 | 0.05639098000000002 | 0.03225806 |
| 0.28000000000000008 | 0.19791670000000006 | 0.12138729999999998 | 0.1385542 | 0.125 | 0.09375000000000004 | 0.07462686 | 0.09740259 | 0.03977273 | 0.07253886 | 0.03355705 | 0.1801242 | 0.17241380000000006 | 0.07857143 | 0.08196721000000001 | 0.196347 | 0.1534884000000001 | 0.18942730000000008 | 0.1308411 | 0.1203008 | 0.03225806 |
| 0.3000000000000001 | 0.15104170000000006 | 0.1445087 | 0.1927711 | 0.172619 | 0.13125 | 0.11940300000000002 | 0.1363636 | 0.1022727 | 0.1139896 | 0.1275168 | 0.1677019 | 0.11822660000000006 | 0.1285714 | 0.05737705 | 0.18264840000000007 | 0.12093020000000003 | 0.1806167 | 0.1308411 | 0.1466165 | 0.043010750000000014 |
| 0.32000000000000012 | 0.13020830000000005 | 0.1676301 | 0.20481930000000007 | 0.1190476 | 0.1125 | 0.07462686 | 0.1623377 | 0.21590910000000008 | 0.13989640000000006 | 0.18120810000000007 | 0.1428571 | 0.1576355 | 0.15714290000000006 | 0.16393440000000006 | 0.1187215 | 0.11627910000000002 | 0.17621140000000007 | 0.11682240000000002 | 0.1466165 | 0.043010750000000014 |
| 0.34 | 0.0625 | 0.10404620000000005 | 0.1325301 | 0.1369048 | 0.1625 | 0.16417909999999997 | 0.1753247 | 0.1193182 | 0.1243523 | 0.18791950000000007 | 0.08695652000000004 | 0.11822660000000006 | 0.1285714 | 0.1967213 | 0.10958900000000002 | 0.14883720000000006 | 0.06607929999999998 | 0.10280370000000003 | 0.10150380000000002 | 0.08064516000000004 |
| 0.3600000000000001 | 0.046874999999999986 | 0.12138729999999998 | 0.08433735000000005 | 0.13095240000000005 | 0.13125 | 0.11940300000000002 | 0.09740259 | 0.11363640000000003 | 0.1295337 | 0.20134230000000006 | 0.0931677 | 0.04926108 | 0.11428570000000003 | 0.09016393 | 0.045662100000000004 | 0.04651163 | 0.035242290000000016 | 0.060747660000000016 | 0.1278196 | 0.1344086 |
| 0.38000000000000012 | 0.02604167000000001 | 0.05202312 | 0.03614458 | 0.08333334000000003 | 0.08125 | 0.16417909999999997 | 0.07142857 | 0.09659091000000003 | 0.07772021000000003 | 0.10738259999999998 | 0.043478259999999984 | 0.06403941000000003 | 0.08571429000000003 | 0.06557377 | 0.01826484 | 0.041860460000000016 | 0.035242290000000016 | 0.07009346000000002 | 0.07894737 | 0.1344086 |
| 0.4 | 0.020833330000000018 | 0.02890173 | 0.02409638 | 0.04761905 | 0.06875000000000002 | 0.1119403 | 0.06493507 | 0.1022727 | 0.04663213 | 0.04697986999999997 | 0.006211180000000002 | 0.01970443000000001 | 0.09285714000000005 | 0.11475410000000003 | 0.01826484 | 0.04651163 | 0.01762114 | 0.03738318 | 0.06766918 | 0.1397849 |
| 0.4200000000000001 | 0.0 | 0.034682080000000004 | 0.02409638 | 0.07738096 | 0.04375 | 0.05970149000000004 | 0.03896104 | 0.05681818 | 0.04663213 | 0.06040268000000003 | 0.02484472 | 0.01970443000000001 | 0.02142857 | 0.04098361000000001 | 0.00913242 | 0.02325581 | 0.004405286000000001 | 0.009345794000000004 | 0.04511278000000002 | 0.09139785 |
| 0.44 | 0.0052083330000000025 | 0.02312138999999999 | 0.0 | 0.0297619 | 0.0125 | 0.03731343 | 0.01298701 | 0.02840909 | 0.07253886 | 0.013422820000000007 | 0.0 | 0.004926108000000002 | 0.02142857 | 0.0 | 0.00913242 | 0.018604650000000007 | 0.008810572000000004 | 0.004672897 | 0.030075190000000012 | 0.10215050000000002 |
| 0.46 | 0.0052083330000000025 | 0.017341040000000002 | 0.018072289999999998 | 0.005952381 | 0.025 | 0.007462686000000002 | 0.01948052000000001 | 0.005681818 | 0.04663213 | 0.0 | 0.0 | 0.01970443000000001 | 0.01428571 | 0.016393440000000002 | 0.00456621 | 0.009302326000000007 | 0.004405286000000001 | 0.0 | 0.007518797000000002 | 0.06451613000000003 |
| 0.48000000000000009 | 0.0052083330000000025 | 0.011560690000000004 | 0.0 | 0.02380952 | 0.0125 | 0.007462686000000002 | 0.01948052000000001 | 0.005681818 | 0.02072539 | 0.0 | 0.0 | 0.00985221700000001 | 0.007142857 | 0.008196721 | 0.0 | 0.004651163000000002 | 0.0 | 0.0 | 0.007518797000000002 | 0.037634410000000014 |
| 0.5 | 0.0 | 0.0057803470000000025 | 0.0 | 0.0 | 0.0 | 0.0 | 0.0 | 0.005681818 | 0.01036269 | 0.0 | 0.0 | 0.00985221700000001 | 0.0 | 0.0 | 0.0 | 0.004651163000000002 | 0.004405286000000001 | 0.004672897 | 0.003759399000000002 | 0.026881720000000008 |
| 0.52 | 0.0 | 0.0057803470000000025 | 0.0 | 0.0 | 0.0 | 0.0 | 0.0 | 0.0 | 0.02072539 | 0.0 | 0.0 | 0.0 | 0.0 | 0.008196721 | 0.0 | 0.0 | 0.0 | 0.0 | 0.003759399000000002 | 0.016129030000000003 |
| 0.54 | 0.0 | 0.0 | 0.0 | 0.005952381 | 0.0 | 0.007462686000000002 | 0.006493506000000002 | 0.005681818 | 0.005181347000000002 | 0.0 | 0.0 | 0.00985221700000001 | 0.0 | 0.016393440000000002 | 0.0 | 0.0 | 0.0 | 0.0 | 0.0 | 0.0 |
| 0.56000000000000005 | 0.0 | 0.0 | 0.0 | 0.0 | 0.0 | 0.0 | 0.0 | 0.005681818 | 0.01554404 | 0.0 | 0.0 | 0.0 | 0.0 | 0.008196721 | 0.0 | 0.0 | 0.0 | 0.0 | 0.003759399000000002 | 0.0 |
| 0.58000000000000007 | 0.0 | 0.0 | 0.0 | 0.0 | 0.0 | 0.0 | 0.0 | 0.005681818 | 0.0 | 0.0 | 0.0 | 0.004926108000000002 | 0.0 | 0.008196721 | 0.0 | 0.0 | 0.0 | 0.0 | 0.0 | 0.005376344000000003 |
| 0.6000000000000002 | 0.0 | 0.0 | 0.0 | 0.0 | 0.0 | 0.0 | 0.0 | 0.0 | 0.005181347000000002 | 0.0 | 0.0 | 0.0 | 0.0 | 0.0 | 0.0 | 0.0 | 0.0 | 0.0 | 0.0 | 0.0 |
| 0.62000000000000022 | 0.0 | 0.0 | 0.0 | 0.0 | 0.0 | 0.0 | 0.0 | 0.005681818 | 0.0 | 0.0 | 0.0 | 0.0 | 0.0 | 0.0 | 0.0 | 0.0 | 0.0 | 0.0 | 0.0 | 0.0 |
| 0.64000000000000024 | 0.0052083330000000025 | 0.0 | 0.0 | 0.0 | 0.0 | 0.0 | 0.0 | 0.0 | 0.005181347000000002 | 0.0 | 0.0 | 0.0 | 0.0 | 0.0 | 0.0 | 0.0 | 0.0 | 0.0 | 0.0 | 0.005376344000000003 |
| 0.66000000000000025 | 0.0 | 0.0 | 0.0 | 0.0 | 0.0 | 0.0 | 0.0 | 0.0 | 0.0 | 0.0 | 0.0 | 0.0 | 0.0 | 0.0 | 0.0 | 0.0 | 0.0 | 0.0 | 0.0 | 0.0 |
| 0.68 | 0.0 | 0.0 | 0.0 | 0.0 | 0.0 | 0.0 | 0.0 | 0.0 | 0.0 | 0.0 | 0.0 | 0.0 | 0.0 | 0.0 | 0.0 | 0.0 | 0.0 | 0.0 | 0.0 | 0.005376344000000003 |
| 0.70000000000000018 | 0.0 | 0.0 | 0.0 | 0.0 | 0.0 | 0.0 | 0.0 | 0.0 | 0.0 | 0.0 | 0.0 | 0.0 | 0.0 | 0.0 | 0.0 | 0.0 | 0.0 | 0.0 | 0.0 | 0.0 |
### Chart: cat-1 A5
| Category | cat-1 A5 No.1 | cat-1 A5 No.2 | cat-1 A5 No.3 | cat-1 A5 No.4 | cat-1 A5 No.6 | cat-1 A5 No.7 | cat-1 A5 No.8 | cat-1 A5 No.9 | cat-1 A5 No.10 | cat-1 A5 No.11 | cat-1 A5 No.12 | cat-1 A5 No.13 | cat-1 A5 No.14 | cat-1 A5 No.15 | cat-1 A5 No.16 | cat-1 A5 No.17 | cat-1 A5 No.18 | cat-1 A5 No.19 | cat-1 A5 No.20 | cat-1 A5 No.21 | cat-1 A5 No.22 | cat-1 A5 No.23 | cat-1 A5 No.24 | cat-1 A5 No.25 | cat-1 A5 No.26 | cat-1 A5 No.27 |
|---|---|---|---|---|---|---|---|---|---|---|---|---|---|---|---|---|---|---|---|---|---|---|---|---|---|---|
| 0 | None | None | None | None | None | None | None | None | None | None | None | None | None | None | None | None | None | None | None | None | None | None | None | None | None | None |
| 2.0000000000000007E-2 | 0.09375000000000004 | 0.0 | 0.020270270000000014 | 0.005882353 | 0.020000000000000007 | 0.0 | 0.006060606 | 0.005586592000000002 | 0.06521739000000001 | 0.0 | 0.0 | 0.02380952 | 0.04411765000000003 | 0.006250000000000002 | 0.0 | 0.2 | 0.03846154 | 0.0 | 0.009174312 | 0.0 | 0.0 | 0.0 | 0.0 | 0.0 | 0.0 | 0.03125 |
| 4.0000000000000015E-2 | 0.0625 | 0.0 | 0.006756757000000002 | 0.0 | 0.040000000000000015 | 0.0 | 0.0 | 0.0 | 0.02173913 | 0.0072463770000000035 | 0.0 | 0.0 | 0.02941176 | 0.0125 | 0.0 | 0.16 | 0.03846154 | 0.0 | 0.009174312 | 0.0 | 0.0 | 0.0 | 0.0 | 0.0 | 0.0 | 0.0625 |
| 6.0000000000000019E-2 | 0.0625 | 0.0 | 0.0 | 0.0 | 0.040000000000000015 | 0.0 | 0.0 | 0.0 | 0.043478259999999984 | 0.0 | 0.0 | 0.0 | 0.0 | 0.0125 | 0.004524886999999997 | 0.0 | 0.09615385000000004 | 0.0 | 0.02752294 | 0.0 | 0.0 | 0.005263158 | 0.0 | 0.0 | 0.0 | 0.046874999999999986 |
| 8.0000000000000029E-2 | 0.0 | 0.0 | 0.0 | 0.0 | 0.0 | 0.0 | 0.0 | 0.005586592000000002 | 0.02173913 | 0.0 | 0.0 | 0.02380952 | 0.0 | 0.0 | 0.0 | 0.08000000000000003 | 0.019230770000000008 | 0.0 | 0.009174312 | 0.0 | 0.0 | 0.0 | 0.0 | 0.0 | 0.0 | 0.03125 |
| 0.1 | 0.0 | 0.0 | 0.0 | 0.0 | 0.0 | 0.0 | 0.006060606 | 0.0 | 0.02173913 | 0.0 | 0.0 | 0.0 | 0.0 | 0.0 | 0.0 | 0.040000000000000015 | 0.03846154 | 0.009433962 | 0.0 | 0.0 | 0.0 | 0.005263158 | 0.0 | 0.0 | 0.0 | 0.0 |
| 0.12000000000000002 | 0.03125 | 0.0 | 0.0 | 0.0 | 0.0 | 0.0 | 0.0 | 0.005586592000000002 | 0.02173913 | 0.0 | 0.0 | 0.0 | 0.02941176 | 0.006250000000000002 | 0.004524886999999997 | 0.0 | 0.0 | 0.0 | 0.0 | 0.0 | 0.0 | 0.0 | 0.0 | 0.0 | 0.0 | 0.0 |
| 0.14000000000000001 | 0.0 | 0.0 | 0.006756757000000002 | 0.0 | 0.0 | 0.0 | 0.0 | 0.0 | 0.0 | 0.0072463770000000035 | 0.0 | 0.0 | 0.014705880000000001 | 0.0125 | 0.004524886999999997 | 0.040000000000000015 | 0.019230770000000008 | 0.0 | 0.0 | 0.007352941000000002 | 0.006622516999999998 | 0.0 | 0.0 | 0.0 | 0.0 | 0.015625 |
| 0.16 | 0.0 | 0.0 | 0.006756757000000002 | 0.0 | 0.020000000000000007 | 0.0 | 0.0 | 0.011173180000000001 | 0.0 | 0.0 | 0.0 | 0.0 | 0.02941176 | 0.0 | 0.004524886999999997 | 0.0 | 0.019230770000000008 | 0.009433962 | 0.0 | 0.007352941000000002 | 0.006622516999999998 | 0.015789470000000003 | 0.0 | 0.0 | 0.0 | 0.0 |
| 0.18000000000000005 | 0.0 | 0.0 | 0.006756757000000002 | 0.0 | 0.0 | 0.0 | 0.0 | 0.0 | 0.0 | 0.0072463770000000035 | 0.0 | 0.0 | 0.0 | 0.0 | 0.009049774 | 0.040000000000000015 | 0.0 | 0.009433962 | 0.009174312 | 0.0 | 0.006622516999999998 | 0.02631579 | 0.0 | 0.0 | 0.0 | 0.03125 |
| 0.2 | 0.0 | 0.0 | 0.027027030000000007 | 0.0 | 0.0 | 0.0 | 0.0 | 0.0 | 0.0 | 0.02173913 | 0.0 | 0.0 | 0.0 | 0.01875 | 0.031674210000000015 | 0.0 | 0.019230770000000008 | 0.028301890000000007 | 0.0 | 0.02205882 | 0.019867550000000008 | 0.02631579 | 0.0 | 0.025 | 0.0 | 0.0 |
| 0.22 | 0.0 | 0.0 | 0.027027030000000007 | 0.0 | 0.08000000000000003 | 0.0 | 0.006060606 | 0.005586592000000002 | 0.0 | 0.02173913 | 0.0 | 0.0 | 0.014705880000000001 | 0.01875 | 0.01809955 | 0.0 | 0.019230770000000008 | 0.056603769999999984 | 0.0 | 0.05147058999999998 | 0.052980130000000014 | 0.036842100000000017 | 0.0 | 0.010000000000000004 | 0.0 | 0.015625 |
| 0.24000000000000005 | 0.0625 | 0.039800990000000015 | 0.06081081000000002 | 0.0 | 0.0 | 0.0 | 0.012121209999999999 | 0.022346370000000008 | 0.043478259999999984 | 0.02173913 | 0.015384620000000003 | 0.0 | 0.0 | 0.037500000000000006 | 0.0361991 | 0.0 | 0.019230770000000008 | 0.06603774 | 0.02752294 | 0.02941176 | 0.0397351 | 0.05263158 | 0.007092198000000002 | 0.020000000000000007 | 0.021052629999999992 | 0.0625 |
| 0.26 | 0.03125 | 0.07462686 | 0.07432432 | 0.005882353 | 0.020000000000000007 | 0.02840909 | 0.024242419999999997 | 0.022346370000000008 | 0.043478259999999984 | 0.036231880000000015 | 0.05384614999999999 | 0.02380952 | 0.0 | 0.025 | 0.06787331000000002 | 0.12000000000000002 | 0.03846154 | 0.0754717 | 0.08256881 | 0.07352941 | 0.052980130000000014 | 0.09473684000000007 | 0.007092198000000002 | 0.03500000000000001 | 0.05263158 | 0.046874999999999986 |
| 0.28000000000000008 | 0.125 | 0.11940300000000002 | 0.16891890000000007 | 0.005882353 | 0.06000000000000002 | 0.03409091 | 0.06666667 | 0.033519549999999995 | 0.02173913 | 0.050724640000000015 | 0.08461539000000007 | 0.0 | 0.02941176 | 0.08750000000000001 | 0.12217190000000003 | 0.040000000000000015 | 0.03846154 | 0.06603774 | 0.08256881 | 0.08823530000000006 | 0.09933775 | 0.07894737 | 0.06382979 | 0.04500000000000001 | 0.07368421 | 0.078125 |
| 0.3000000000000001 | 0.03125 | 0.1144279 | 0.1554054000000001 | 0.04705882 | 0.020000000000000007 | 0.07386363000000003 | 0.1151515 | 0.050279329999999976 | 0.1304348 | 0.1304348 | 0.08461539000000007 | 0.04761905 | 0.04411765000000003 | 0.08125 | 0.14479640000000008 | 0.040000000000000015 | 0.0 | 0.12264150000000003 | 0.08256881 | 0.125 | 0.1125828 | 0.08421053000000002 | 0.03546099 | 0.05 | 0.10526320000000006 | 0.125 |
| 0.32000000000000012 | 0.125 | 0.1840796 | 0.18243240000000008 | 0.07058824 | 0.12000000000000002 | 0.14772730000000006 | 0.12121210000000003 | 0.09497207 | 0.10869560000000006 | 0.10869560000000006 | 0.13846150000000004 | 0.07142857 | 0.08823530000000006 | 0.1125 | 0.15384620000000007 | 0.0 | 0.05769231000000001 | 0.0754717 | 0.1743119 | 0.08823530000000006 | 0.1059603 | 0.10526320000000006 | 0.04255319 | 0.125 | 0.1368421 | 0.125 |
| 0.34 | 0.0 | 0.08955224000000006 | 0.10135139999999995 | 0.17058820000000005 | 0.1 | 0.21590910000000008 | 0.1636364 | 0.10614520000000004 | 0.1304348 | 0.1014493 | 0.10769230000000003 | 0.1666667 | 0.02941176 | 0.16875 | 0.1312217 | 0.0 | 0.07692308000000002 | 0.056603769999999984 | 0.09174312 | 0.1985294 | 0.1059603 | 0.1368421 | 0.07092199 | 0.125 | 0.1368421 | 0.0625 |
| 0.3600000000000001 | 0.18750000000000006 | 0.12437810000000002 | 0.06081081000000002 | 0.1294118 | 0.040000000000000015 | 0.15340910000000008 | 0.1636364 | 0.11731839999999996 | 0.10869560000000006 | 0.1014493 | 0.08461539000000007 | 0.1190476 | 0.1323529 | 0.09375000000000004 | 0.08597285 | 0.040000000000000015 | 0.09615385000000004 | 0.1132075 | 0.11009170000000003 | 0.08823530000000006 | 0.1125828 | 0.1 | 0.09929078000000002 | 0.15500000000000005 | 0.15789470000000005 | 0.078125 |
| 0.38000000000000012 | 0.125 | 0.09950249000000005 | 0.06756756 | 0.1176471 | 0.06000000000000002 | 0.11363640000000003 | 0.10909090000000003 | 0.10614520000000004 | 0.043478259999999984 | 0.1304348 | 0.09230769 | 0.07142857 | 0.19117649999999997 | 0.08750000000000001 | 0.054298640000000016 | 0.0 | 0.09615385000000004 | 0.08490566000000004 | 0.06422018 | 0.04411765000000003 | 0.08609272 | 0.07368421 | 0.14893620000000007 | 0.15500000000000005 | 0.1368421 | 0.0625 |
| 0.4 | 0.03125 | 0.05970149000000004 | 0.020270270000000014 | 0.09411765000000002 | 0.08000000000000003 | 0.125 | 0.07272727000000002 | 0.09497207 | 0.06521739000000001 | 0.05797102 | 0.08461539000000007 | 0.1190476 | 0.05882353 | 0.05624999999999998 | 0.0361991 | 0.12000000000000002 | 0.07692308000000002 | 0.056603769999999984 | 0.036697250000000015 | 0.05147058999999998 | 0.07284768000000003 | 0.05789474000000002 | 0.11347520000000003 | 0.07500000000000001 | 0.05263158 | 0.015625 |
| 0.4200000000000001 | 0.03125 | 0.039800990000000015 | 0.006756757000000002 | 0.1117647 | 0.1 | 0.0625 | 0.04848484999999999 | 0.08379889000000007 | 0.043478259999999984 | 0.02898551 | 0.03846154 | 0.07142857 | 0.014705880000000001 | 0.03125 | 0.04977376000000002 | 0.0 | 0.05769231000000001 | 0.028301890000000007 | 0.055045869999999976 | 0.04411765000000003 | 0.0397351 | 0.05263158 | 0.14893620000000007 | 0.07500000000000001 | 0.021052629999999992 | 0.046874999999999986 |
| 0.44 | 0.0 | 0.019900500000000015 | 0.0 | 0.052941179999999984 | 0.08000000000000003 | 0.03409091 | 0.03636364000000001 | 0.07821229000000003 | 0.0 | 0.05797102 | 0.1153846 | 0.0952381 | 0.05882353 | 0.037500000000000006 | 0.01357466 | 0.0 | 0.03846154 | 0.04716981 | 0.02752294 | 0.007352941000000002 | 0.03311258 | 0.02631579 | 0.07092199 | 0.040000000000000015 | 0.03157895 | 0.03125 |
| 0.46 | 0.0 | 0.009950249000000006 | 0.0 | 0.07058824 | 0.06000000000000002 | 0.011363640000000001 | 0.024242419999999997 | 0.06145252 | 0.02173913 | 0.02173913 | 0.02307692 | 0.04761905 | 0.02941176 | 0.025 | 0.004524886999999997 | 0.0 | 0.019230770000000008 | 0.018867920000000003 | 0.036697250000000015 | 0.014705880000000001 | 0.013245030000000001 | 0.0 | 0.07801419 | 0.020000000000000007 | 0.021052629999999992 | 0.0 |
| 0.48000000000000009 | 0.0 | 0.009950249000000006 | 0.0 | 0.06470589000000003 | 0.0 | 0.0 | 0.018181820000000008 | 0.027932960000000014 | 0.02173913 | 0.043478259999999984 | 0.03076923 | 0.02380952 | 0.05882353 | 0.006250000000000002 | 0.009049774 | 0.08000000000000003 | 0.03846154 | 0.028301890000000007 | 0.02752294 | 0.007352941000000002 | 0.013245030000000001 | 0.01052632 | 0.021276600000000007 | 0.014999999999999998 | 0.0 | 0.03125 |
| 0.5 | 0.0 | 0.009950249000000006 | 0.0 | 0.03529412 | 0.020000000000000007 | 0.0 | 0.006060606 | 0.011173180000000001 | 0.0 | 0.0072463770000000035 | 0.007692308000000002 | 0.04761905 | 0.02941176 | 0.025 | 0.0 | 0.0 | 0.0 | 0.028301890000000007 | 0.018348620000000003 | 0.007352941000000002 | 0.0 | 0.0 | 0.03546099 | 0.014999999999999998 | 0.021052629999999992 | 0.0 |
| 0.52 | 0.0 | 0.0 | 0.0 | 0.0 | 0.0 | 0.0 | 0.0 | 0.027932960000000014 | 0.0 | 0.0 | 0.015384620000000003 | 0.0 | 0.014705880000000001 | 0.006250000000000002 | 0.0 | 0.0 | 0.0 | 0.009433962 | 0.009174312 | 0.02941176 | 0.0 | 0.005263158 | 0.021276600000000007 | 0.0 | 0.0 | 0.0 |
| 0.54 | 0.0 | 0.0 | 0.0 | 0.0 | 0.0 | 0.0 | 0.0 | 0.022346370000000008 | 0.0 | 0.02898551 | 0.007692308000000002 | 0.0 | 0.014705880000000001 | 0.0125 | 0.0 | 0.0 | 0.019230770000000008 | 0.009433962 | 0.009174312 | 0.0 | 0.006622516999999998 | 0.005263158 | 0.014184400000000003 | 0.005000000000000002 | 0.0 | 0.0 |
| 0.56000000000000005 | 0.0 | 0.004975124000000002 | 0.0 | 0.0 | 0.020000000000000007 | 0.0 | 0.0 | 0.0 | 0.0 | 0.0 | 0.0 | 0.0 | 0.0 | 0.0 | 0.004524886999999997 | 0.0 | 0.019230770000000008 | 0.0 | 0.0 | 0.0 | 0.006622516999999998 | 0.0 | 0.0 | 0.0 | 0.0 | 0.0 |
| 0.58000000000000007 | 0.0 | 0.0 | 0.0 | 0.011764710000000001 | 0.0 | 0.0 | 0.0 | 0.005586592000000002 | 0.0 | 0.0 | 0.0 | 0.0 | 0.014705880000000001 | 0.006250000000000002 | 0.0 | 0.0 | 0.0 | 0.0 | 0.0 | 0.0 | 0.0 | 0.0 | 0.0 | 0.0 | 0.021052629999999992 | 0.0 |
| 0.6000000000000002 | 0.0 | 0.0 | 0.0 | 0.005882353 | 0.0 | 0.0 | 0.0 | 0.0 | 0.0 | 0.0 | 0.007692308000000002 | 0.0 | 0.0 | 0.0 | 0.004524886999999997 | 0.0 | 0.0 | 0.0 | 0.0 | 0.007352941000000002 | 0.0 | 0.0 | 0.014184400000000003 | 0.0 | 0.0 | 0.0 |
| 0.62000000000000022 | 0.0 | 0.0 | 0.0 | 0.0 | 0.020000000000000007 | 0.0 | 0.0 | 0.0 | 0.0 | 0.0072463770000000035 | 0.0 | 0.0 | 0.014705880000000001 | 0.0 | 0.004524886999999997 | 0.0 | 0.0 | 0.0 | 0.0 | 0.0 | 0.0 | 0.0 | 0.0 | 0.005000000000000002 | 0.0 | 0.0 |
| 0.64000000000000024 | 0.0 | 0.0 | 0.0 | 0.0 | 0.0 | 0.0 | 0.0 | 0.0 | 0.02173913 | 0.0 | 0.0 | 0.0 | 0.0 | 0.0 | 0.0 | 0.0 | 0.0 | 0.0 | 0.0 | 0.0 | 0.006622516999999998 | 0.0 | 0.0 | 0.0 | 0.01052632 | 0.0 |
| 0.66000000000000025 | 0.0 | 0.0 | 0.0 | 0.0 | 0.0 | 0.0 | 0.0 | 0.0 | 0.0 | 0.0 | 0.0 | 0.0 | 0.014705880000000001 | 0.0 | 0.0 | 0.0 | 0.0 | 0.0 | 0.0 | 0.0 | 0.0 | 0.0 | 0.0 | 0.005000000000000002 | 0.0 | 0.0 |
| 0.68 | 0.0 | 0.0 | 0.0 | 0.0 | 0.0 | 0.0 | 0.0 | 0.0 | 0.0 | 0.0 | 0.007692308000000002 | 0.04761905 | 0.0 | 0.006250000000000002 | 0.0 | 0.0 | 0.0 | 0.0 | 0.0 | 0.0 | 0.0 | 0.0 | 0.0 | 0.0 | 0.0 | 0.0 |
| 0.70000000000000018 | 0.0 | 0.0 | 0.0 | 0.0 | 0.0 | 0.0 | 0.0 | 0.0 | 0.0 | 0.0 | 0.0 | 0.0 | 0.0 | 0.0 | 0.0 | 0.0 | 0.0 | 0.0 | 0.0 | 0.0 | 0.0 | 0.0 | 0.0 | 0.0 | 0.0 | 0.0 |
### Chart: cat-2 A1
| Category | cat-2 A1 No.1 | cat-2 A1 No.2 | cat-2 A1 No.3 | cat-2 A1 No.4 | cat-2 A1 No.5 | cat-2 A1 No.6 | cat-2 A1 No.7 | cat-2 A1 No.8 | cat-2 A1 No.9 | cat-2 A1 No.10 | cat-2 A1 No.11 | cat-2 A1 No.12 | cat-2 A1 No.13 | cat-2 A1 No.14 | cat-2 A1 No.15 | cat-2 A1 No.16 | cat-2 A1 No.17 | cat-2 A1 No.18 | cat-2 A1 No.19 | cat-2 A1 No.20 |
|---|---|---|---|---|---|---|---|---|---|---|---|---|---|---|---|---|---|---|---|---|
| 0 | None | None | None | None | None | None | None | None | None | None | None | None | None | None | None | None | None | None | None | None |
| 2.0000000000000007E-2 | 0.0 | 0.008695652000000005 | 0.0 | 0.0 | 0.0 | 0.0 | 0.0 | 0.0 | 0.0 | 0.0 | 0.0 | 0.0 | 0.0 | 0.0 | 0.0 | 0.0 | 0.0 | 0.0 | 0.0 | 0.0 |
| 4.0000000000000015E-2 | 0.0 | 0.0 | 0.0 | 0.0 | 0.0 | 0.0 | 0.005076142000000002 | 0.0 | 0.0 | 0.0 | 0.0 | 0.0 | 0.0 | 0.0078125 | 0.0 | 0.0 | 0.0 | 0.0 | 0.0 | 0.007352941000000002 |
| 6.0000000000000019E-2 | 0.0 | 0.0 | 0.0 | 0.0 | 0.0 | 0.0 | 0.0 | 0.0 | 0.0 | 0.0 | 0.00952381 | 0.0 | 0.0 | 0.0 | 0.0 | 0.0 | 0.0 | 0.0 | 0.0 | 0.0 |
| 8.0000000000000029E-2 | 0.0 | 0.008695652000000005 | 0.0 | 0.0 | 0.0 | 0.0 | 0.005076142000000002 | 0.0 | 0.0 | 0.0 | 0.00952381 | 0.0 | 0.0 | 0.0 | 0.0 | 0.0 | 0.0 | 0.0 | 0.0 | 0.007352941000000002 |
| 0.1 | 0.0 | 0.0 | 0.0 | 0.0 | 0.0 | 0.0 | 0.0 | 0.0 | 0.0 | 0.0 | 0.0 | 0.0 | 0.0 | 0.0 | 0.0 | 0.0 | 0.0 | 0.0 | 0.0 | 0.007352941000000002 |
| 0.12000000000000002 | 0.0 | 0.0 | 0.0 | 0.0 | 0.0 | 0.0 | 0.0 | 0.0 | 0.0 | 0.0 | 0.0 | 0.0 | 0.0 | 0.0 | 0.0 | 0.0 | 0.0 | 0.0 | 0.0 | 0.0 |
| 0.14000000000000001 | 0.006410256000000002 | 0.008695652000000005 | 0.013513510000000001 | 0.0 | 0.0 | 0.0 | 0.0 | 0.0 | 0.0 | 0.0 | 0.00952381 | 0.0 | 0.0 | 0.0078125 | 0.0 | 0.0 | 0.0 | 0.0 | 0.0 | 0.007352941000000002 |
| 0.16 | 0.012820510000000004 | 0.0 | 0.0 | 0.0 | 0.0 | 0.0 | 0.02030457 | 0.0 | 0.0 | 0.0069930070000000025 | 0.0 | 0.0 | 0.0 | 0.0078125 | 0.0 | 0.0 | 0.0 | 0.006250000000000002 | 0.0 | 0.014705880000000001 |
| 0.18000000000000005 | 0.019230770000000008 | 0.0 | 0.0 | 0.006756757000000002 | 0.0 | 0.0 | 0.035533 | 0.005263158 | 0.0 | 0.0069930070000000025 | 0.0 | 0.0 | 0.0 | 0.0078125 | 0.0 | 0.0 | 0.0 | 0.0 | 0.012269939999999998 | 0.007352941000000002 |
| 0.2 | 0.012820510000000004 | 0.02608696 | 0.006756757000000002 | 0.0 | 0.0 | 0.01724138 | 0.02030457 | 0.0 | 0.0 | 0.02097902 | 0.01904762 | 0.013333330000000001 | 0.0 | 0.023437500000000007 | 0.005681818 | 0.01408451 | 0.0 | 0.0 | 0.0 | 0.014705880000000001 |
| 0.22 | 0.0 | 0.008695652000000005 | 0.013513510000000001 | 0.006756757000000002 | 0.006666667000000002 | 0.0 | 0.03045685 | 0.0 | 0.01481481 | 0.0069930070000000025 | 0.01904762 | 0.026666670000000007 | 0.0 | 0.03125 | 0.005681818 | 0.0 | 0.0 | 0.01875 | 0.0 | 0.007352941000000002 |
| 0.24000000000000005 | 0.032051280000000015 | 0.02608696 | 0.06081081000000002 | 0.013513510000000001 | 0.0 | 0.051724139999999995 | 0.04568528 | 0.005263158 | 0.05925926 | 0.013986010000000004 | 0.00952381 | 0.05333333000000002 | 0.005617978 | 0.015625 | 0.011363640000000001 | 0.01408451 | 0.0 | 0.0125 | 0.030674850000000007 | 0.04411765000000003 |
| 0.26 | 0.05128205 | 0.043478259999999984 | 0.047297300000000014 | 0.013513510000000001 | 0.026666670000000007 | 0.06896552 | 0.04568528 | 0.015789470000000003 | 0.02962963 | 0.013986010000000004 | 0.02857142999999999 | 0.06666667 | 0.005617978 | 0.08593750000000003 | 0.03409091 | 0.0 | 0.008695652000000005 | 0.0625 | 0.02453988 | 0.05147058999999998 |
| 0.28000000000000008 | 0.07051282000000003 | 0.07826087 | 0.09459460000000007 | 0.07432432 | 0.03333334000000002 | 0.0862069 | 0.06598983999999998 | 0.07368421 | 0.03703704000000002 | 0.09090909000000003 | 0.05714285999999998 | 0.12000000000000002 | 0.01685393 | 0.054687500000000014 | 0.04545455 | 0.05633803000000001 | 0.06086956 | 0.0625 | 0.036809820000000014 | 0.07352941 |
| 0.3000000000000001 | 0.06410257 | 0.1565217 | 0.12162160000000004 | 0.08783784000000003 | 0.020000000000000007 | 0.06896552 | 0.09137056 | 0.06842105 | 0.14814810000000006 | 0.0979021 | 0.03809524 | 0.08000000000000003 | 0.03370786 | 0.140625 | 0.07386363000000003 | 0.09859155000000004 | 0.10434779999999998 | 0.125 | 0.10429450000000005 | 0.10294120000000002 |
| 0.32000000000000012 | 0.07692308000000002 | 0.11304350000000002 | 0.16891890000000007 | 0.1283784 | 0.08666667000000003 | 0.18965520000000005 | 0.10152280000000002 | 0.08421053000000002 | 0.14074070000000005 | 0.1328671 | 0.1238095 | 0.1333333 | 0.08988764 | 0.1640625 | 0.14204550000000005 | 0.11267610000000003 | 0.06086956 | 0.125 | 0.12269940000000003 | 0.10294120000000002 |
| 0.34 | 0.1666667 | 0.09565217000000005 | 0.1283784 | 0.1418919 | 0.1 | 0.06896552 | 0.1370558 | 0.10526320000000006 | 0.1185185 | 0.09090909000000003 | 0.1238095 | 0.2 | 0.08426967 | 0.1328125 | 0.11363640000000003 | 0.09859155000000004 | 0.1478261 | 0.125 | 0.1533742 | 0.1470588 |
| 0.3600000000000001 | 0.13461540000000005 | 0.1478261 | 0.1486486 | 0.08783784000000003 | 0.1133333 | 0.06896552 | 0.08121827000000001 | 0.1 | 0.1185185 | 0.12587409999999993 | 0.152381 | 0.08000000000000003 | 0.08988764 | 0.08593750000000003 | 0.15340910000000008 | 0.2253521 | 0.226087 | 0.08750000000000001 | 0.07361963000000003 | 0.09558824 |
| 0.38000000000000012 | 0.10256410000000005 | 0.11304350000000002 | 0.07432432 | 0.12162160000000004 | 0.08000000000000003 | 0.18965520000000005 | 0.10152280000000002 | 0.08947369 | 0.07407407000000003 | 0.09090909000000003 | 0.1714286 | 0.10666670000000006 | 0.12359550000000004 | 0.054687500000000014 | 0.1363636 | 0.14084510000000006 | 0.12173910000000003 | 0.10625000000000002 | 0.1533742 | 0.05147058999999998 |
| 0.4 | 0.1153846 | 0.06086956 | 0.047297300000000014 | 0.09459460000000007 | 0.14 | 0.06896552 | 0.08629441 | 0.1 | 0.08148148 | 0.09090909000000003 | 0.04761905 | 0.040000000000000015 | 0.08988764 | 0.054687500000000014 | 0.07386363000000003 | 0.07042254000000002 | 0.05217391 | 0.125 | 0.08588957000000001 | 0.07352941 |
| 0.4200000000000001 | 0.03846154 | 0.05217391 | 0.047297300000000014 | 0.07432432 | 0.08666667000000003 | 0.051724139999999995 | 0.04568528 | 0.1421053 | 0.06666667 | 0.0979021 | 0.10476190000000005 | 0.026666670000000007 | 0.07865169 | 0.078125 | 0.08522727000000002 | 0.07042254000000002 | 0.09565217000000005 | 0.0625 | 0.06748466000000003 | 0.06617647 |
| 0.44 | 0.019230770000000008 | 0.043478259999999984 | 0.013513510000000001 | 0.08108108 | 0.07333333000000003 | 0.051724139999999995 | 0.035533 | 0.06315788999999998 | 0.05185184999999997 | 0.04895104999999999 | 0.00952381 | 0.013333330000000001 | 0.12359550000000004 | 0.0078125 | 0.05681818 | 0.01408451 | 0.043478259999999984 | 0.037500000000000006 | 0.049079749999999984 | 0.06617647 |
| 0.46 | 0.012820510000000004 | 0.008695652000000005 | 0.013513510000000001 | 0.03378378 | 0.040000000000000015 | 0.01724138 | 0.015228430000000001 | 0.08947369 | 0.02962963 | 0.02097902 | 0.03809524 | 0.013333330000000001 | 0.08988764 | 0.0390625 | 0.017045460000000002 | 0.04225352 | 0.008695652000000005 | 0.01875 | 0.04294479 | 0.007352941000000002 |
| 0.48000000000000009 | 0.019230770000000008 | 0.0 | 0.0 | 0.013513510000000001 | 0.040000000000000015 | 0.0 | 0.01015228 | 0.015789470000000003 | 0.007407407000000002 | 0.02097902 | 0.01904762 | 0.026666670000000007 | 0.02808989 | 0.0 | 0.022727270000000008 | 0.02816901 | 0.03478261 | 0.006250000000000002 | 0.01840491 | 0.014705880000000001 |
| 0.5 | 0.032051280000000015 | 0.0 | 0.0 | 0.013513510000000001 | 0.046666670000000014 | 0.0 | 0.005076142000000002 | 0.021052629999999992 | 0.007407407000000002 | 0.0069930070000000025 | 0.00952381 | 0.0 | 0.03932584 | 0.0 | 0.005681818 | 0.01408451 | 0.0 | 0.0125 | 0.01840491 | 0.02205882 |
| 0.52 | 0.006410256000000002 | 0.0 | 0.0 | 0.0 | 0.040000000000000015 | 0.0 | 0.005076142000000002 | 0.015789470000000003 | 0.0 | 0.0069930070000000025 | 0.0 | 0.0 | 0.0505618 | 0.0 | 0.005681818 | 0.0 | 0.0 | 0.0 | 0.0 | 0.007352941000000002 |
| 0.54 | 0.0 | 0.0 | 0.0 | 0.0 | 0.026666670000000007 | 0.0 | 0.0 | 0.005263158 | 0.0 | 0.0 | 0.0 | 0.0 | 0.01123596 | 0.0 | 0.005681818 | 0.0 | 0.0173913 | 0.006250000000000002 | 0.0 | 0.0 |
| 0.56000000000000005 | 0.0 | 0.0 | 0.0 | 0.0 | 0.013333330000000001 | 0.0 | 0.0 | 0.0 | 0.007407407000000002 | 0.0 | 0.0 | 0.0 | 0.02247191000000002 | 0.0 | 0.005681818 | 0.0 | 0.0 | 0.0 | 0.0 | 0.0 |
| 0.58000000000000007 | 0.0 | 0.0 | 0.0 | 0.006756757000000002 | 0.006666667000000002 | 0.0 | 0.005076142000000002 | 0.0 | 0.0 | 0.0 | 0.0 | 0.0 | 0.005617978 | 0.0 | 0.0 | 0.0 | 0.0 | 0.0 | 0.006134969 | 0.0 |
| 0.6000000000000002 | 0.0 | 0.0 | 0.0 | 0.0 | 0.006666667000000002 | 0.0 | 0.0 | 0.0 | 0.0 | 0.0 | 0.0 | 0.0 | 0.01123596 | 0.0 | 0.0 | 0.0 | 0.008695652000000005 | 0.0 | 0.0 | 0.0 |
| 0.62000000000000022 | 0.0 | 0.0 | 0.0 | 0.0 | 0.006666667000000002 | 0.0 | 0.0 | 0.0 | 0.0 | 0.0 | 0.0 | 0.0 | 0.0 | 0.0 | 0.0 | 0.0 | 0.0 | 0.0 | 0.0 | 0.0 |
| 0.64000000000000024 | 0.0 | 0.0 | 0.0 | 0.0 | 0.0 | 0.0 | 0.0 | 0.0 | 0.0 | 0.0 | 0.0 | 0.0 | 0.0 | 0.0 | 0.0 | 0.0 | 0.0 | 0.0 | 0.0 | 0.0 |
| 0.66000000000000025 | 0.0 | 0.0 | 0.0 | 0.0 | 0.006666667000000002 | 0.0 | 0.005076142000000002 | 0.0 | 0.0 | 0.0069930070000000025 | 0.0 | 0.0 | 0.0 | 0.0 | 0.0 | 0.0 | 0.0 | 0.0 | 0.0 | 0.0 |
| 0.68 | 0.0 | 0.0 | 0.0 | 0.0 | 0.0 | 0.0 | 0.0 | 0.0 | 0.0 | 0.0 | 0.0 | 0.0 | 0.0 | 0.0 | 0.0 | 0.0 | 0.0 | 0.0 | 0.0 | 0.0 |
| 0.70000000000000018 | 0.0 | 0.0 | 0.0 | 0.0 | 0.0 | 0.0 | 0.0 | 0.0 | 0.007407407000000002 | 0.0 | 0.0 | 0.0 | 0.0 | 0.0 | 0.0 | 0.0 | 0.0 | 0.0 | 0.0 | 0.0 |
### Chart: cat-2 A3
| Category | cat-2 A3 No.1 | cat-2 A3 No.2 | cat-2 A3 No.3 | cat-2 A3 No.4 | cat-2 A3 No.5 | cat-2 A3 No.6 | cat-2 A3 No.7 | cat-2 A3 No.8 | cat-2 A3 No.9 | cat-2 A3 No.10 | cat-2 A3 No.11 | cat-2 A3 No.12 | cat-2 A3 No.13 | cat-2 A3 No.14 | cat-2 A3 No.15 | cat-2 A3 No.16 | cat-2 A3 No.17 | cat-2 A3 No.18 | cat-2 A3 No.19 |
|---|---|---|---|---|---|---|---|---|---|---|---|---|---|---|---|---|---|---|---|
| 0 | None | None | None | None | None | None | None | None | None | None | None | None | None | None | None | None | None | None | None |
| 2.0000000000000007E-2 | 0.0 | 0.0 | 0.0 | 0.0 | 0.0 | 0.0 | 0.0 | 0.0 | 0.0 | 0.0 | 0.0 | 0.0 | 0.0 | 0.0 | 0.0 | 0.0 | 0.0 | 0.0 | 0.0 |
| 4.0000000000000015E-2 | 0.0 | 0.0 | 0.0 | 0.0 | 0.0 | 0.0 | 0.0 | 0.0 | 0.0 | 0.0 | 0.0 | 0.0 | 0.0 | 0.0 | 0.0 | 0.0 | 0.0 | 0.0 | 0.0 |
| 6.0000000000000019E-2 | 0.0 | 0.0 | 0.0 | 0.005917160000000002 | 0.0 | 0.0 | 0.0 | 0.0 | 0.0 | 0.0 | 0.0 | 0.0 | 0.0 | 0.0 | 0.0 | 0.0 | 0.0 | 0.0 | 0.0 |
| 8.0000000000000029E-2 | 0.0 | 0.0 | 0.0 | 0.005917160000000002 | 0.0 | 0.0 | 0.0 | 0.0 | 0.0 | 0.0 | 0.0 | 0.0 | 0.0 | 0.0 | 0.0 | 0.0 | 0.0 | 0.0 | 0.0 |
| 0.1 | 0.0 | 0.0 | 0.0 | 0.0 | 0.0 | 0.0 | 0.007874016 | 0.0 | 0.0 | 0.0 | 0.0 | 0.0 | 0.0 | 0.0 | 0.0 | 0.0 | 0.0 | 0.0 | 0.0 |
| 0.12000000000000002 | 0.0 | 0.0 | 0.0 | 0.0 | 0.0 | 0.0 | 0.0 | 0.0 | 0.0 | 0.0 | 0.0 | 0.0 | 0.0 | 0.0 | 0.0 | 0.0 | 0.0 | 0.0 | 0.0 |
| 0.14000000000000001 | 0.0 | 0.007352941000000002 | 0.0 | 0.0 | 0.0 | 0.0 | 0.0 | 0.0 | 0.0 | 0.0 | 0.0 | 0.0 | 0.0 | 0.0 | 0.0 | 0.0 | 0.006756757000000002 | 0.0 | 0.0 |
| 0.16 | 0.0 | 0.0 | 0.0 | 0.0 | 0.0 | 0.0 | 0.0 | 0.0 | 0.0 | 0.0 | 0.0 | 0.0 | 0.0 | 0.0 | 0.0 | 0.0 | 0.0 | 0.0 | 0.0 |
| 0.18000000000000005 | 0.0 | 0.0 | 0.0 | 0.0 | 0.0 | 0.0 | 0.0 | 0.0 | 0.0 | 0.0 | 0.003968254000000002 | 0.0 | 0.01010101 | 0.0 | 0.0 | 0.0 | 0.006756757000000002 | 0.0 | 0.0 |
| 0.2 | 0.008264462000000005 | 0.03676471 | 0.0 | 0.011834320000000004 | 0.006493506000000002 | 0.0 | 0.0 | 0.0 | 0.0 | 0.0 | 0.0 | 0.018518520000000007 | 0.005050505 | 0.02409638 | 0.0 | 0.0 | 0.006756757000000002 | 0.0 | 0.0 |
| 0.22 | 0.0 | 0.07352941 | 0.006944444000000002 | 0.023668639999999994 | 0.006493506000000002 | 0.02142857 | 0.0 | 0.015151520000000005 | 0.01652892 | 0.0 | 0.003968254000000002 | 0.05555555999999998 | 0.03535353 | 0.02409638 | 0.0 | 0.02209945 | 0.006756757000000002 | 0.006711409000000002 | 0.03947368000000001 |
| 0.24000000000000005 | 0.0 | 0.10294120000000002 | 0.0 | 0.08875740000000006 | 0.01948052000000001 | 0.02142857 | 0.01574803 | 0.06060606 | 0.03305785 | 0.0 | 0.019841270000000008 | 0.17901230000000007 | 0.0959596 | 0.1445783 | 0.01948052000000001 | 0.03314917 | 0.040540539999999986 | 0.020134229999999993 | 0.03947368000000001 |
| 0.26 | 0.05785124000000001 | 0.1764706 | 0.03472222 | 0.2781065000000001 | 0.08441558 | 0.13571430000000007 | 0.04724409 | 0.09090909000000003 | 0.09917355000000004 | 0.025641030000000006 | 0.04365079 | 0.2037037 | 0.1313131 | 0.1746988 | 0.006493506000000002 | 0.11602210000000003 | 0.06756756 | 0.020134229999999993 | 0.06578948 |
| 0.28000000000000008 | 0.02479339 | 0.23529410000000006 | 0.020833330000000018 | 0.2071006 | 0.1688312 | 0.21428570000000005 | 0.05511811 | 0.21969700000000006 | 0.11570250000000003 | 0.04273504 | 0.04761905 | 0.2777778 | 0.2070707 | 0.23493980000000006 | 0.09740259 | 0.1325967 | 0.1486486 | 0.04697986999999997 | 0.1381579 |
| 0.3000000000000001 | 0.05785124000000001 | 0.1838235 | 0.08333334000000003 | 0.14792900000000006 | 0.18831170000000005 | 0.2642857 | 0.15748030000000007 | 0.22727269999999997 | 0.1735537 | 0.1282051 | 0.1190476 | 0.154321 | 0.18686870000000005 | 0.16867469999999996 | 0.07142857 | 0.19337019999999994 | 0.18243240000000008 | 0.147651 | 0.15789470000000005 |
| 0.32000000000000012 | 0.08264463000000002 | 0.1176471 | 0.08333334000000003 | 0.07100592 | 0.1753247 | 0.15714290000000006 | 0.18110240000000005 | 0.17424240000000007 | 0.24793390000000007 | 0.15384620000000007 | 0.1190476 | 0.07407407000000003 | 0.1262626 | 0.09638554 | 0.1623377 | 0.1823204 | 0.2162162000000001 | 0.18120810000000007 | 0.15789470000000005 |
| 0.34 | 0.14049590000000006 | 0.04411765000000003 | 0.11805560000000002 | 0.041420119999999984 | 0.1493506 | 0.11428570000000003 | 0.1653543 | 0.09848485000000007 | 0.11570250000000003 | 0.2136752 | 0.2222222 | 0.03703704000000002 | 0.09090909000000003 | 0.07831325000000003 | 0.1753247 | 0.11602210000000003 | 0.17567569999999993 | 0.2080537 | 0.131579 |
| 0.3600000000000001 | 0.11570250000000003 | 0.007352941000000002 | 0.1666667 | 0.03550296 | 0.07142857 | 0.02857142999999999 | 0.14173230000000006 | 0.0530303 | 0.09090909000000003 | 0.15384620000000007 | 0.1428571 | 0.0 | 0.05050504999999999 | 0.02409638 | 0.1688312 | 0.1104972 | 0.08108108 | 0.10738259999999998 | 0.15131580000000006 |
| 0.38000000000000012 | 0.11570250000000003 | 0.007352941000000002 | 0.15972220000000006 | 0.0295858 | 0.058441559999999976 | 0.02142857 | 0.15748030000000007 | 0.022727270000000008 | 0.0661157 | 0.1367521 | 0.1150794 | 0.0 | 0.03030303 | 0.0 | 0.11038959999999996 | 0.04972376000000002 | 0.040540539999999986 | 0.10067110000000003 | 0.05263158 |
| 0.4 | 0.09090909000000003 | 0.0 | 0.06944445000000002 | 0.0295858 | 0.03896104 | 0.01428571 | 0.03149606 | 0.0 | 0.02479339 | 0.05982906 | 0.06349207000000003 | 0.0 | 0.015151520000000005 | 0.0 | 0.10389610000000005 | 0.016574590000000007 | 0.013513510000000001 | 0.06711409000000003 | 0.04605263000000001 |
| 0.4200000000000001 | 0.05785124000000001 | 0.0 | 0.08333334000000003 | 0.0 | 0.0 | 0.0 | 0.01574803 | 0.015151520000000005 | 0.008264462000000005 | 0.05128205 | 0.06746032000000003 | 0.0 | 0.015151520000000005 | 0.01204819 | 0.03246753 | 0.011049720000000004 | 0.006756757000000002 | 0.053691280000000015 | 0.0 |
| 0.44 | 0.0661157 | 0.0 | 0.0625 | 0.011834320000000004 | 0.006493506000000002 | 0.007142857 | 0.0 | 0.007575758000000004 | 0.0 | 0.008547009000000003 | 0.003968254000000002 | 0.0 | 0.0 | 0.0 | 0.02597403 | 0.0 | 0.0 | 0.006711409000000002 | 0.01315789 |
| 0.46 | 0.041322310000000015 | 0.0 | 0.04166667000000002 | 0.011834320000000004 | 0.01298701 | 0.0 | 0.01574803 | 0.0 | 0.008264462000000005 | 0.008547009000000003 | 0.02380952 | 0.0 | 0.0 | 0.006024096 | 0.006493506000000002 | 0.016574590000000007 | 0.0 | 0.013422820000000007 | 0.0065789470000000025 |
| 0.48000000000000009 | 0.03305785 | 0.0 | 0.02777778000000001 | 0.0 | 0.0 | 0.0 | 0.0 | 0.0 | 0.0 | 0.008547009000000003 | 0.0 | 0.0 | 0.0 | 0.0 | 0.01298701 | 0.0 | 0.0 | 0.0 | 0.0 |
| 0.5 | 0.008264462000000005 | 0.0 | 0.020833330000000018 | 0.0 | 0.006493506000000002 | 0.0 | 0.007874016 | 0.0 | 0.0 | 0.0 | 0.003968254000000002 | 0.0 | 0.0 | 0.0 | 0.0 | 0.0 | 0.0 | 0.013422820000000007 | 0.0 |
| 0.52 | 0.03305785 | 0.0 | 0.006944444000000002 | 0.0 | 0.0 | 0.0 | 0.0 | 0.0 | 0.0 | 0.0 | 0.0 | 0.0 | 0.0 | 0.006024096 | 0.0 | 0.0 | 0.0 | 0.0 | 0.0 |
| 0.54 | 0.03305785 | 0.0 | 0.0 | 0.0 | 0.006493506000000002 | 0.0 | 0.0 | 0.007575758000000004 | 0.0 | 0.008547009000000003 | 0.0 | 0.0 | 0.0 | 0.0 | 0.006493506000000002 | 0.0 | 0.0 | 0.0 | 0.0 |
| 0.56000000000000005 | 0.008264462000000005 | 0.0 | 0.006944444000000002 | 0.0 | 0.0 | 0.0 | 0.0 | 0.007575758000000004 | 0.0 | 0.0 | 0.0 | 0.0 | 0.0 | 0.006024096 | 0.0 | 0.0 | 0.0 | 0.0 | 0.0 |
| 0.58000000000000007 | 0.0 | 0.0 | 0.0 | 0.0 | 0.0 | 0.0 | 0.0 | 0.0 | 0.0 | 0.0 | 0.0 | 0.0 | 0.0 | 0.0 | 0.0 | 0.0 | 0.0 | 0.006711409000000002 | 0.0 |
| 0.6000000000000002 | 0.008264462000000005 | 0.007352941000000002 | 0.006944444000000002 | 0.0 | 0.0 | 0.0 | 0.0 | 0.0 | 0.0 | 0.0 | 0.0 | 0.0 | 0.0 | 0.0 | 0.0 | 0.0 | 0.0 | 0.0 | 0.0 |
| 0.62000000000000022 | 0.0 | 0.0 | 0.0 | 0.0 | 0.0 | 0.0 | 0.0 | 0.0 | 0.0 | 0.0 | 0.0 | 0.0 | 0.0 | 0.0 | 0.0 | 0.0 | 0.0 | 0.0 | 0.0 |
| 0.64000000000000024 | 0.01652892 | 0.0 | 0.0 | 0.0 | 0.0 | 0.0 | 0.0 | 0.0 | 0.0 | 0.0 | 0.0 | 0.0 | 0.0 | 0.0 | 0.0 | 0.0 | 0.0 | 0.0 | 0.0 |
| 0.66000000000000025 | 0.0 | 0.0 | 0.0 | 0.0 | 0.0 | 0.0 | 0.0 | 0.0 | 0.0 | 0.0 | 0.0 | 0.0 | 0.0 | 0.0 | 0.0 | 0.0 | 0.0 | 0.0 | 0.0 |
| 0.68 | 0.0 | 0.0 | 0.0 | 0.0 | 0.0 | 0.0 | 0.0 | 0.0 | 0.0 | 0.0 | 0.0 | 0.0 | 0.0 | 0.0 | 0.0 | 0.0 | 0.0 | 0.0 | 0.0 |
| 0.70000000000000018 | 0.0 | 0.0 | 0.0 | 0.0 | 0.0 | 0.0 | 0.0 | 0.0 | 0.0 | 0.0 | 0.0 | 0.0 | 0.0 | 0.0 | 0.0 | 0.0 | 0.0 | 0.0 | 0.0 |
### Chart: cat-2 A5
| Category | cat-2 A5 No.1 | cat-2 A5 No.2 | cat-2 A5 No.3 | cat-2 A5 No.4 | cat-2 A5 No.5 | cat-2 A5 No.6 | cat-2 A5 No.7 | cat-2 A5 No.8 | cat-2 A5 No.9 | cat-2 A5 No.10 | cat-2 A5 No.11 | cat-2 A5 No.12 | cat-2 A5 No.13 | cat-2 A5 No.14 | cat-2 A5 No.15 | cat-2 A5 No.16 | cat-2 A5 No.17 | cat-2 A5 No.18 | cat-2 A5 No.19 | cat-2 A5 No.20 |
|---|---|---|---|---|---|---|---|---|---|---|---|---|---|---|---|---|---|---|---|---|
| 0 | None | None | None | None | None | None | None | None | None | None | None | None | None | None | None | None | None | None | None | None |
| 2.0000000000000007E-2 | 0.0 | 0.0 | 0.0 | 0.006849315 | 0.0 | 0.0 | 0.01449275 | 0.0 | 0.0 | 0.0 | 0.0 | 0.0 | 0.0 | 0.0 | 0.0 | 0.01948052000000001 | 0.0 | 0.0 | 0.0 | 0.0 |
| 4.0000000000000015E-2 | 0.0 | 0.0 | 0.0 | 0.01369863 | 0.0 | 0.0 | 0.01449275 | 0.0 | 0.0 | 0.0 | 0.0 | 0.0 | 0.0 | 0.0 | 0.0 | 0.006493506000000002 | 0.0 | 0.0069930070000000025 | 0.0 | 0.0 |
| 6.0000000000000019E-2 | 0.0 | 0.0 | 0.0 | 0.0 | 0.0 | 0.0 | 0.0 | 0.007142857 | 0.0 | 0.0 | 0.0 | 0.0 | 0.0 | 0.0 | 0.01449275 | 0.0 | 0.0 | 0.0 | 0.0 | 0.0 |
| 8.0000000000000029E-2 | 0.0 | 0.0 | 0.0 | 0.020547950000000002 | 0.0 | 0.0 | 0.0 | 0.007142857 | 0.0 | 0.0 | 0.0 | 0.0 | 0.0 | 0.0 | 0.0 | 0.01948052000000001 | 0.0 | 0.0069930070000000025 | 0.0 | 0.0 |
| 0.1 | 0.0 | 0.0 | 0.0 | 0.0 | 0.0 | 0.0070422530000000035 | 0.0 | 0.01428571 | 0.0 | 0.0 | 0.0 | 0.0 | 0.0 | 0.0 | 0.0 | 0.01298701 | 0.0 | 0.0 | 0.0 | 0.0 |
| 0.12000000000000002 | 0.0 | 0.0 | 0.0 | 0.0 | 0.0 | 0.0 | 0.0 | 0.0 | 0.0072992700000000035 | 0.0 | 0.0 | 0.007936508000000004 | 0.0 | 0.0 | 0.0 | 0.006493506000000002 | 0.0 | 0.0 | 0.0 | 0.0 |
| 0.14000000000000001 | 0.0 | 0.008264462000000005 | 0.0 | 0.0 | 0.0 | 0.0070422530000000035 | 0.0 | 0.01428571 | 0.0072992700000000035 | 0.0 | 0.0 | 0.0 | 0.0 | 0.0 | 0.0 | 0.006493506000000002 | 0.008620690000000004 | 0.0 | 0.0 | 0.0 |
| 0.16 | 0.0 | 0.008264462000000005 | 0.008064516000000004 | 0.006849315 | 0.0 | 0.0070422530000000035 | 0.01449275 | 0.0 | 0.0 | 0.0 | 0.0 | 0.0 | 0.0 | 0.0 | 0.01449275 | 0.01298701 | 0.0 | 0.0 | 0.0 | 0.0 |
| 0.18000000000000005 | 0.0 | 0.0 | 0.008064516000000004 | 0.006849315 | 0.0 | 0.0070422530000000035 | 0.01449275 | 0.007142857 | 0.0 | 0.0 | 0.0 | 0.0 | 0.0 | 0.011428570000000008 | 0.0 | 0.03896104 | 0.008620690000000004 | 0.0069930070000000025 | 0.0 | 0.0 |
| 0.2 | 0.0 | 0.0 | 0.0 | 0.0 | 0.0 | 0.02816901 | 0.05797102 | 0.007142857 | 0.03649635000000001 | 0.007874016 | 0.0 | 0.007936508000000004 | 0.007352941000000002 | 0.005714286 | 0.0 | 0.01298701 | 0.008620690000000004 | 0.0 | 0.0 | 0.006666667000000002 |
| 0.22 | 0.0 | 0.0 | 0.024193549999999998 | 0.020547950000000002 | 0.0 | 0.021126759999999994 | 0.01449275 | 0.05 | 0.03649635000000001 | 0.0 | 0.007633588000000002 | 0.02380952 | 0.0 | 0.040000000000000015 | 0.0 | 0.03896104 | 0.008620690000000004 | 0.0069930070000000025 | 0.0 | 0.006666667000000002 |
| 0.24000000000000005 | 0.006134969 | 0.008264462000000005 | 0.016129030000000003 | 0.020547950000000002 | 0.015267180000000003 | 0.07746479000000003 | 0.043478259999999984 | 0.06428572000000002 | 0.0 | 0.01574803 | 0.019083970000000006 | 0.06349207000000003 | 0.02205882 | 0.05714285999999998 | 0.01449275 | 0.07142857 | 0.0 | 0.013986010000000004 | 0.0 | 0.013333330000000001 |
| 0.26 | 0.006134969 | 0.008264462000000005 | 0.04032258000000001 | 0.006849315 | 0.030534350000000002 | 0.1338028 | 0.05797102 | 0.06428572000000002 | 0.05109489 | 0.0 | 0.03816794 | 0.1269841 | 0.06617647 | 0.09714286000000003 | 0.02898551 | 0.03896104 | 0.1034483 | 0.02097902 | 0.008474576000000004 | 0.0 |
| 0.28000000000000008 | 0.04294479 | 0.041322310000000015 | 0.07258064000000003 | 0.06849315 | 0.05343511 | 0.1197183 | 0.1304348 | 0.07142857 | 0.1021898 | 0.07874016 | 0.08778626 | 0.0952381 | 0.125 | 0.1028571 | 0.043478259999999984 | 0.08441558 | 0.06034483 | 0.06993007 | 0.0 | 0.046666670000000014 |
| 0.3000000000000001 | 0.036809820000000014 | 0.0661157 | 0.10483870000000002 | 0.08904110000000003 | 0.09923664000000003 | 0.09154929 | 0.08695652000000004 | 0.1 | 0.058394160000000014 | 0.08661418000000004 | 0.129771 | 0.20634920000000007 | 0.1985294 | 0.2 | 0.07246377000000002 | 0.06493507 | 0.1034483 | 0.12587409999999993 | 0.008474576000000004 | 0.06000000000000002 |
| 0.32000000000000012 | 0.08588957000000001 | 0.10743800000000002 | 0.2096774 | 0.10273970000000003 | 0.1984733 | 0.1338028 | 0.2028985 | 0.1785714 | 0.10948910000000002 | 0.1259843 | 0.1526718 | 0.19841270000000005 | 0.1617647 | 0.1428571 | 0.1304348 | 0.06493507 | 0.17241380000000006 | 0.1328671 | 0.059322030000000026 | 0.08000000000000003 |
| 0.34 | 0.12269940000000003 | 0.08264463000000002 | 0.15322580000000005 | 0.13698630000000006 | 0.1374046 | 0.11267610000000003 | 0.1014493 | 0.15000000000000005 | 0.1167883 | 0.1338583 | 0.1717557 | 0.1269841 | 0.1985294 | 0.1371429 | 0.05797102 | 0.058441559999999976 | 0.17241380000000006 | 0.1188811 | 0.07627117999999997 | 0.15333330000000006 |
| 0.3600000000000001 | 0.12269940000000003 | 0.13223140000000005 | 0.07258064000000003 | 0.13698630000000006 | 0.16793890000000006 | 0.11267610000000003 | 0.05797102 | 0.09285714000000005 | 0.1751825 | 0.14173230000000006 | 0.11832060000000003 | 0.05555555999999998 | 0.09558824 | 0.08000000000000003 | 0.18840580000000007 | 0.07792208000000003 | 0.1293103 | 0.1398601 | 0.1101695 | 0.2 |
| 0.38000000000000012 | 0.1656442 | 0.1570248 | 0.08870967000000003 | 0.130137 | 0.10687020000000003 | 0.04225352 | 0.05797102 | 0.02857142999999999 | 0.1167883 | 0.1338583 | 0.1145038 | 0.03968254 | 0.06617647 | 0.045714290000000025 | 0.2028985 | 0.07142857 | 0.09482758000000005 | 0.11188809999999996 | 0.2033898 | 0.12666669999999997 |
| 0.4 | 0.15950920000000007 | 0.19008259999999993 | 0.05645161 | 0.08219178 | 0.061068700000000004 | 0.07042254000000002 | 0.05797102 | 0.07857143 | 0.08029197 | 0.05511811 | 0.06488550000000001 | 0.03968254 | 0.02941176 | 0.03428571000000001 | 0.05797102 | 0.07792208000000003 | 0.1034483 | 0.10489510000000005 | 0.11864410000000003 | 0.09333333 |
| 0.4200000000000001 | 0.07361963000000003 | 0.03305785 | 0.06451613000000003 | 0.061643839999999984 | 0.02290076 | 0.01408451 | 0.0 | 0.04285714 | 0.05109489 | 0.08661418000000004 | 0.04198473 | 0.0 | 0.014705880000000001 | 0.005714286 | 0.07246377000000002 | 0.02597403 | 0.01724138 | 0.03496503 | 0.09322034000000008 | 0.06000000000000002 |
| 0.44 | 0.07361963000000003 | 0.05785124000000001 | 0.04032258000000001 | 0.020547950000000002 | 0.007633588000000002 | 0.0070422530000000035 | 0.0 | 0.01428571 | 0.014598539999999998 | 0.03149606 | 0.015267180000000003 | 0.007936508000000004 | 0.0 | 0.005714286 | 0.01449275 | 0.03246753 | 0.008620690000000004 | 0.03496503 | 0.09322034000000008 | 0.05333333000000002 |
| 0.46 | 0.06134969 | 0.041322310000000015 | 0.008064516000000004 | 0.020547950000000002 | 0.030534350000000002 | 0.0 | 0.02898551 | 0.0 | 0.0072992700000000035 | 0.03149606 | 0.011450380000000001 | 0.0 | 0.0 | 0.02857142999999999 | 0.02898551 | 0.02597403 | 0.0 | 0.03496503 | 0.09322034000000008 | 0.05333333000000002 |
| 0.48000000000000009 | 0.012269939999999998 | 0.01652892 | 0.008064516000000004 | 0.020547950000000002 | 0.015267180000000003 | 0.0 | 0.043478259999999984 | 0.007142857 | 0.0072992700000000035 | 0.007874016 | 0.011450380000000001 | 0.0 | 0.0 | 0.005714286 | 0.02898551 | 0.03246753 | 0.0 | 0.013986010000000004 | 0.05084746 | 0.020000000000000007 |
| 0.5 | 0.006134969 | 0.03305785 | 0.016129030000000003 | 0.0 | 0.02290076 | 0.0 | 0.0 | 0.0 | 0.0072992700000000035 | 0.03149606 | 0.003816794000000001 | 0.0 | 0.007352941000000002 | 0.0 | 0.01449275 | 0.03246753 | 0.0 | 0.0 | 0.059322030000000026 | 0.006666667000000002 |
| 0.52 | 0.006134969 | 0.008264462000000005 | 0.0 | 0.0 | 0.015267180000000003 | 0.0 | 0.0 | 0.0 | 0.0 | 0.01574803 | 0.0 | 0.0 | 0.007352941000000002 | 0.0 | 0.0 | 0.01298701 | 0.0 | 0.013986010000000004 | 0.008474576000000004 | 0.013333330000000001 |
| 0.54 | 0.012269939999999998 | 0.0 | 0.0 | 0.0 | 0.007633588000000002 | 0.0070422530000000035 | 0.0 | 0.0 | 0.0 | 0.0 | 0.003816794000000001 | 0.0 | 0.0 | 0.0 | 0.01449275 | 0.01298701 | 0.0 | 0.0 | 0.008474576000000004 | 0.0 |
| 0.56000000000000005 | 0.0 | 0.0 | 0.008064516000000004 | 0.006849315 | 0.0 | 0.0 | 0.0 | 0.0 | 0.0072992700000000035 | 0.0 | 0.0 | 0.0 | 0.0 | 0.0 | 0.0 | 0.0 | 0.0 | 0.0 | 0.008474576000000004 | 0.006666667000000002 |
| 0.58000000000000007 | 0.006134969 | 0.0 | 0.0 | 0.006849315 | 0.007633588000000002 | 0.0 | 0.0 | 0.0 | 0.0072992700000000035 | 0.007874016 | 0.0 | 0.0 | 0.0 | 0.0 | 0.0 | 0.006493506000000002 | 0.0 | 0.0 | 0.0 | 0.0 |
| 0.6000000000000002 | 0.0 | 0.0 | 0.0 | 0.0 | 0.0 | 0.0 | 0.0 | 0.0 | 0.0 | 0.0 | 0.003816794000000001 | 0.0 | 0.0 | 0.0 | 0.0 | 0.0 | 0.0 | 0.0 | 0.0 | 0.0 |
| 0.62000000000000022 | 0.0 | 0.0 | 0.0 | 0.0 | 0.0 | 0.0 | 0.0 | 0.0 | 0.0 | 0.0 | 0.003816794000000001 | 0.0 | 0.0 | 0.0 | 0.0 | 0.01948052000000001 | 0.0 | 0.0 | 0.0 | 0.0 |
| 0.64000000000000024 | 0.0 | 0.0 | 0.0 | 0.0 | 0.0 | 0.0 | 0.0 | 0.0 | 0.0 | 0.007874016 | 0.0 | 0.0 | 0.0 | 0.0 | 0.0 | 0.0 | 0.0 | 0.0 | 0.0 | 0.0 |
| 0.66000000000000025 | 0.0 | 0.0 | 0.0 | 0.006849315 | 0.0 | 0.0 | 0.0 | 0.0 | 0.0 | 0.0 | 0.0 | 0.0 | 0.0 | 0.0 | 0.0 | 0.006493506000000002 | 0.0 | 0.0 | 0.0 | 0.0 |
| 0.68 | 0.0 | 0.0 | 0.0 | 0.0 | 0.0 | 0.0 | 0.0 | 0.0 | 0.0 | 0.0 | 0.0 | 0.0 | 0.0 | 0.0 | 0.0 | 0.0 | 0.0 | 0.0 | 0.0 | 0.0 |
| 0.70000000000000018 | 0.0 | 0.0 | 0.0 | 0.0 | 0.0 | 0.0 | 0.0 | 0.0 | 0.0 | 0.0 | 0.0 | 0.0 | 0.0 | 0.0 | 0.0 | 0.0 | 0.0 | 0.0 | 0.0 | 0.0 |
