# Supplementary material for: Evaluation of Head Movement Periodicity and Irregularity during Locomotion of Caenorhabditis elegans
Source: Front Behav Neurosci. 2013 Mar 21;7:20. doi: 10.3389/fnbeh.2013.00020 (PMC3604732; doi:10.3389/fnbeh.2013.00020)
Supplement: Supplementary Figure S2 — nSL-2 histograms (5%) for each worms. [file 42064_Shingai_Presentation2.PPTX]

## Slide 1
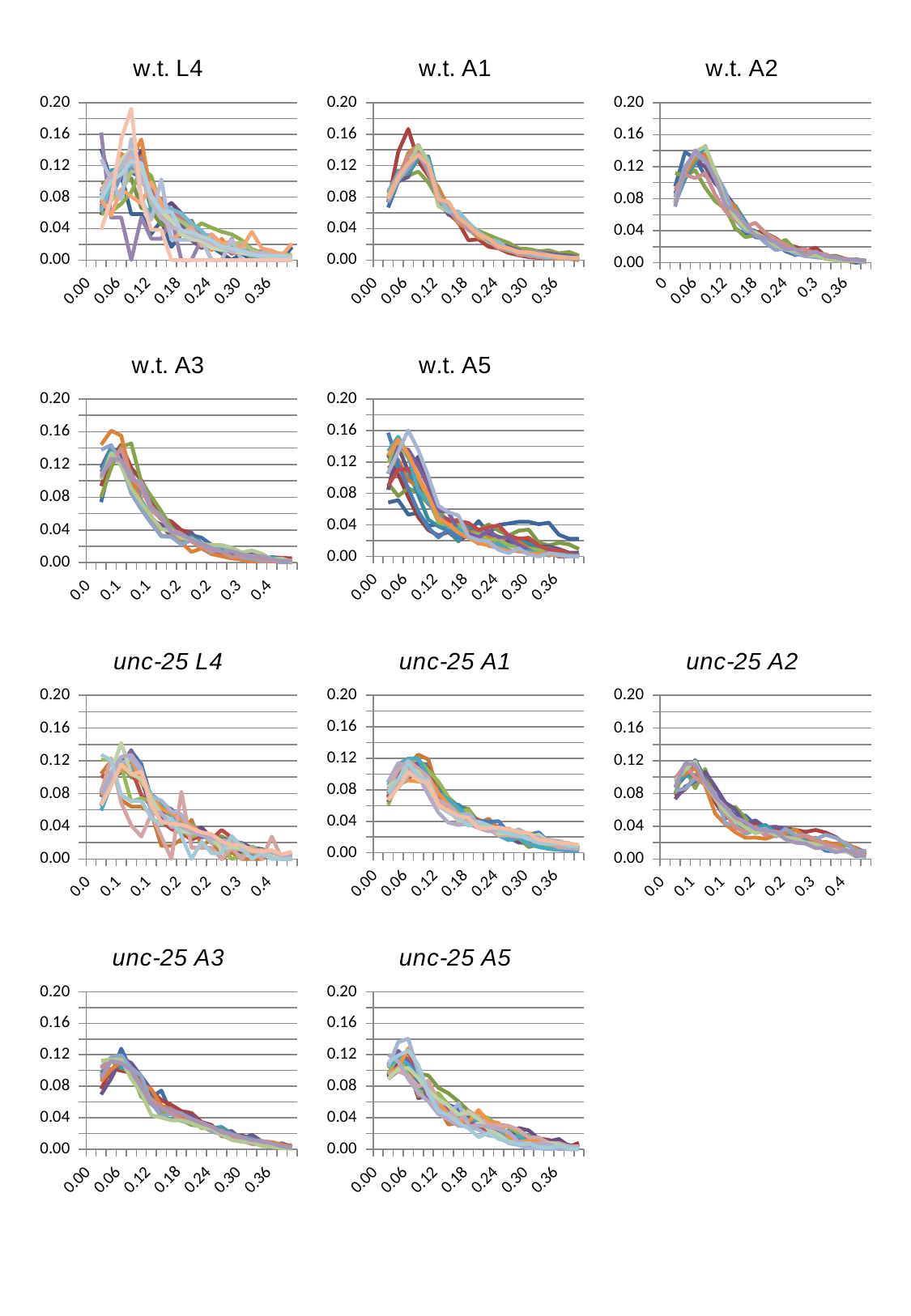

### Chart: w.t. L4
| Category | N2_L4_0601 | N2_L4_0602 | N2_L4_0603 | N2_L4_0604 | N2_L4_0605 | N2_L4_0606 | N2_L4_0607 | N2_L4_0608 | N2_L4_0609 | N2_L4_0611 | N2_L4_0612 | N2_L4_0613 | N2_L4_0614 | N2_L4_0615 | N2_L4_0616 | N2_L4_0617 | N2_L4_0618 | N2_L4_0619 | N2_L4_0621 | N2_L4_0622 | N2_L4_0623 | N2_L4_0624 | N2_L4_0625 | N2_L4_0626 |
|---|---|---|---|---|---|---|---|---|---|---|---|---|---|---|---|---|---|---|---|---|---|---|---|---|
| 0 | None | None | None | None | None | None | None | None | None | None | None | None | None | None | None | None | None | None | None | None | None | None | None | None |
| 2.0000000000000011E-2 | 0.141666666666667 | 0.06833878589603784 | 0.09838998211091245 | 0.0571428571428571 | 0.062040816326530614 | 0.0632046463956269 | None | 0.08666666666666678 | 0.0590551181102362 | 0.07252440725244075 | 0.0818767249310028 | 0.0680851063829787 | 0.0712486505937387 | 0.06637767542671363 | 0.07782515991471221 | 0.1621621621621621 | 0.06245141228297488 | 0.07784431137724551 | 0.128205128205128 | 0.08931698774080558 | 0.08405438813349832 | 0.0785358919687278 | 0.07738693467336684 | 0.0384615384615385 |
| 4.0000000000000022E-2 | 0.1 | 0.09560159941839336 | 0.09838998211091245 | 0.0920634920634922 | 0.10530612244898009 | 0.0983942603348139 | None | 0.10416666666666705 | 0.06130483689538811 | 0.09483960948396095 | 0.11407543698252104 | 0.08085106382978723 | 0.0780856423173804 | 0.09807640205906272 | 0.07569296375266533 | 0.05405405405405411 | 0.09613889608706926 | 0.056886227544910246 | 0.10256410256410305 | 0.108581436077058 | 0.10341985990935296 | 0.10589907604833004 | 0.10016750418760502 | 0.07692307692307691 |
| 6.0000000000000026E-2 | 0.108333333333333 | 0.113413304252999 | 0.11091234347048302 | 0.11519274376417209 | 0.115918367346939 | 0.10796036897847602 | None | 0.10666666666666708 | 0.07142857142857141 | 0.13249651324965092 | 0.11683532658693704 | 0.13475177304964492 | 0.10831234256927004 | 0.115144947168789 | 0.08635394456289987 | 0.05405405405405411 | 0.11609225187872504 | 0.0898203592814372 | 0.07692307692307691 | 0.11908931698774096 | 0.127729707457767 | 0.11869225302061112 | 0.10954773869346696 | 0.15384615384615413 |
| 8.0000000000000043E-2 | 0.05833333333333335 | 0.124318429661941 | 0.10375670840787105 | 0.118367346938776 | 0.12952380952380993 | 0.116843184147591 | None | 0.11666666666666704 | 0.08605174353205851 | 0.111576011157601 | 0.12327506899724004 | 0.12765957446808485 | 0.11586901763224196 | 0.128691411541588 | 0.11194029850746295 | 0.0 | 0.120497538222337 | 0.0808383233532935 | 0.15384615384615413 | 0.13091068301225908 | 0.1400906468891641 | 0.1382373845060412 | 0.12562814070351785 | 0.192307692307692 |
| 0.1 | 0.05833333333333335 | 0.10069065794256604 | 0.0661896243291592 | 0.1392290249433109 | 0.12163265306122412 | 0.1322172873249061 | None | 0.11666666666666704 | 0.11642294713160908 | 0.10460251046025108 | 0.11315547378104907 | 0.15319148936170213 | 0.129543001079525 | 0.12679490652939612 | 0.11407249466951 | 0.05405405405405411 | 0.122829748639544 | 0.07185628742514974 | 0.07692307692307691 | 0.10070052539404598 | 0.11742892459826898 | 0.10341151385927498 | 0.12294807370184302 | 0.07692307692307691 |
| 0.12000000000000002 | 0.0333333333333333 | 0.08796801163213373 | 0.06261180679785329 | 0.07301587301587302 | 0.08190476190476188 | 0.07106252135292117 | None | 0.08416666666666676 | 0.10686164229471302 | 0.09065550906555099 | 0.06347746090156392 | 0.0822695035460993 | 0.07916516732637642 | 0.0839880791113519 | 0.07142857142857141 | 0.02702702702702702 | 0.07877688520342063 | 0.09880239520958087 | 0.0512820512820513 | 0.08143607705779328 | 0.08158220024721874 | 0.07746979388770434 | 0.0800670016750419 | 0.0384615384615385 |
| 0.14000000000000001 | 0.05 | 0.0734278444202108 | 0.044722719141323856 | 0.0621315192743764 | 0.0661224489795918 | 0.0676460539801845 | None | 0.06333333333333337 | 0.0646794150731159 | 0.0669456066945607 | 0.0570377184912604 | 0.0581560283687943 | 0.05289672544080606 | 0.0777567054998645 | 0.0522388059701493 | 0.02702702702702702 | 0.07178025395180104 | 0.07185628742514974 | 0.10256410256410305 | 0.0630472854640981 | 0.06139266584260404 | 0.053304904051172726 | 0.0619765494137353 | 0.0384615384615385 |
| 0.16 | 0.0166666666666667 | 0.06397673573246089 | 0.042933810375670796 | 0.072562358276644 | 0.05959183673469391 | 0.061154765971984965 | None | 0.04583333333333335 | 0.052868391451068655 | 0.04602510460251047 | 0.05151793928242873 | 0.04539007092198584 | 0.06045340050377833 | 0.060417231102682246 | 0.0554371002132196 | 0.05405405405405411 | 0.06478362270018144 | 0.02694610778443112 | 0.025641025641025616 | 0.05122591943957967 | 0.05026782035434693 | 0.041222459132906904 | 0.06130653266331663 | 0.0 |
| 0.18000000000000008 | 0.0333333333333333 | 0.04798255179934573 | 0.0339892665474061 | 0.05941043083900238 | 0.05659863945578233 | 0.05534677143833283 | None | 0.0366666666666667 | 0.04105736782902143 | 0.044630404463040424 | 0.05151793928242873 | 0.053900709219858185 | 0.05037783375314865 | 0.054456786778650804 | 0.04904051172707891 | 0.0 | 0.05908266390256545 | 0.03293413173652692 | 0.025641025641025616 | 0.03633975481611211 | 0.0325504738360115 | 0.0383795309168444 | 0.0368509212730318 | 0.0 |
| 0.2 | 0.05 | 0.0348964013086151 | 0.03041144901610021 | 0.04489795918367352 | 0.047346938775510224 | 0.0379227878373762 | None | 0.0316666666666667 | 0.0371203599550056 | 0.025104602510460317 | 0.0331186752529899 | 0.048226950354609895 | 0.0449802087081684 | 0.0333243023570848 | 0.04051172707889133 | 0.0 | 0.04664420834413057 | 0.0419161676646707 | 0.025641025641025616 | 0.0385288966725044 | 0.026782035434693013 | 0.03162757640369581 | 0.03517587939698491 | 0.0 |
| 0.22 | 0.025 | 0.032715376226826624 | 0.025044722719141314 | 0.036281179138322024 | 0.029931972789115635 | 0.033481380252818604 | None | 0.02416666666666669 | 0.046681664791901 | 0.015341701534170206 | 0.02759889604415819 | 0.025531914893617006 | 0.031666066930550595 | 0.03278244378217292 | 0.025586353944562885 | 0.02702702702702702 | 0.03731536667530452 | 0.01796407185628741 | 0.025641025641025616 | 0.020577933450087602 | 0.0238978162340338 | 0.0277185501066098 | 0.029815745393634806 | 0.0 |
| 0.24000000000000007 | 0.0166666666666667 | 0.023264267539076712 | 0.016100178890876612 | 0.018140589569161022 | 0.0247619047619048 | 0.0239152716091561 | None | 0.025 | 0.04105736782902143 | 0.01952580195258021 | 0.0211591536338546 | 0.012765957446808501 | 0.0313062252608852 | 0.019777837984286107 | 0.01705756929637531 | 0.0 | 0.02254470069966312 | 0.03293413173652692 | 0.0 | 0.0188266199649737 | 0.01524515863205601 | 0.022388059701492487 | 0.02546063651591291 | 0.0 |
| 0.26 | 0.00833333333333333 | 0.014176663031624891 | 0.014311270125223598 | 0.020861678004535113 | 0.017959183673469402 | 0.0239152716091561 | None | 0.0108333333333333 | 0.035995500562429734 | 0.0167364016736402 | 0.01195952161913522 | 0.026950354609929124 | 0.017632241813602 | 0.017339474397182312 | 0.0181236673773987 | 0.0 | 0.019175952319253703 | 0.020958083832335276 | 0.0 | 0.01576182136602451 | 0.0127729707457767 | 0.01670220326936742 | 0.017085427135678403 | 0.0 |
| 0.28000000000000008 | 0.0 | 0.0138131588513268 | 0.016100178890876612 | 0.00816326530612245 | 0.009795918367346945 | 0.016740690126409302 | None | 0.011666666666666707 | 0.0326209223847019 | 0.012552301255230106 | 0.0128794848206072 | 0.009929078014184403 | 0.020870816840590115 | 0.00921159577350312 | 0.0202558635394456 | 0.02702702702702702 | 0.010365379632029006 | 0.023952095808383197 | 0.025641025641025616 | 0.010945709281961513 | 0.0127729707457767 | 0.01172707889125801 | 0.014740368509212703 | 0.0 |
| 0.30000000000000016 | 0.00833333333333333 | 0.00981461286804799 | 0.014311270125223598 | 0.011337868480725601 | 0.009251700680272106 | 0.010249402118209799 | None | 0.0108333333333333 | 0.0253093363329584 | 0.0097629009762901 | 0.01195952161913522 | 0.012765957446808501 | 0.013314141777617807 | 0.008669737198591175 | 0.022388059701492487 | 0.0 | 0.00984711065042757 | 0.0149700598802395 | 0.0 | 0.008756567425569187 | 0.00906468891635765 | 0.013503909026297101 | 0.0110552763819095 | 0.0 |
| 0.32000000000000017 | 0.0 | 0.0072700836059614755 | 0.0017889087656529506 | 0.0090702947845805 | 0.005442176870748297 | 0.006149641270925863 | None | 0.00916666666666668 | 0.014060742407199098 | 0.008368200836820086 | 0.005519779208831654 | 0.012765957446808501 | 0.0104354084202951 | 0.006773232186399357 | 0.012793176972281398 | 0.0 | 0.00544182430681524 | 0.035928143712574814 | 0.0 | 0.009194395796847646 | 0.007004532344458182 | 0.00710732054015636 | 0.00737018425460636 | 0.0 |
| 0.34 | 0.00833333333333333 | 0.006906579425663408 | 0.00536672629695885 | 0.004081632653061223 | 0.0032653061224489814 | 0.00785787495729416 | None | 0.005833333333333336 | 0.009561304836895396 | 0.011157601115760106 | 0.006439742410303599 | 0.0056737588652482325 | 0.00539762504498021 | 0.0037930100243836412 | 0.008528784648187637 | 0.0 | 0.004405286343612343 | 0.0149700598802395 | 0.0 | 0.006129597197898423 | 0.006180469715698396 | 0.009950248756218921 | 0.0050251256281406975 | 0.0 |
| 0.36000000000000015 | 0.00833333333333333 | 0.005452562704471107 | 0.0017889087656529506 | 0.005442176870748297 | 0.005170068027210883 | 0.0037581141100102517 | None | 0.005000000000000003 | 0.0101237345331834 | 0.002789400278940034 | 0.00367985280588776 | 0.0014184397163120599 | 0.006117308384310903 | 0.0029802221620157114 | 0.00959488272921109 | 0.0 | 0.003627882871210162 | 0.011976047904191593 | 0.0 | 0.0021891418563922934 | 0.00412031314379893 | 0.0060412224591329155 | 0.00569514237855946 | 0.0 |
| 0.38000000000000017 | 0.0 | 0.00399854598327881 | 0.0017889087656529506 | 0.00317460317460317 | 0.0038095238095238113 | 0.0027331738981892727 | None | 0.005833333333333336 | 0.005624296962879642 | 0.0013947001394700113 | 0.00367985280588776 | 0.0014184397163120599 | 0.004318100035984173 | 0.0016255757247358416 | 0.008528784648187637 | 0.0 | 0.0038870173620108826 | 0.005988023952095813 | 0.0 | 0.0021891418563922934 | 0.0028842192006592513 | 0.0028429282160625413 | 0.00536013400335008 | 0.0 |
| 0.4 | 0.0166666666666667 | 0.0014540167211922912 | 0.0017889087656529506 | 0.00317460317460317 | 0.002993197278911564 | 0.0037581141100102517 | None | 0.0033333333333333314 | 0.005624296962879642 | 0.002789400278940034 | 0.00183992640294388 | 0.007092198581560283 | 0.0046779417056495125 | 0.0021674342996477933 | 0.005330490405117274 | 0.0 | 0.0015548069448043512 | 0.020958083832335276 | 0.0 | 0.0035026269702276716 | 0.00164812525751957 | 0.00355366027007818 | 0.00402010050251256 | 0.0 |
### Chart: w.t. A1
| Category | N2_A1_40_30_01 | N2_A1_40_30_06 | N2_A1_40_30_07 | N2_REX_A1_01 | N2_REX_A1_03 | N2_REX_A1_05 | N2_REX_A1_07 | N2_REX_A1_08 | N2_REX_A1_09 | N2_REX_A1_10 | N2_REX_A1_11 | N2_REX_A1_12 |
|---|---|---|---|---|---|---|---|---|---|---|---|---|
| 0 | None | None | None | None | None | None | None | None | None | None | None | None |
| 2.0000000000000011E-2 | 0.06679174484052536 | 0.07340206185567015 | 0.07708161582852437 | 0.08518145161290314 | 0.07886372665872077 | 0.08624229979466119 | 0.08714744131058944 | 0.07673199006348334 | 0.0814412635735439 | 0.073290517567057 | 0.07677235482721777 | 0.07841811728916684 |
| 4.0000000000000022E-2 | 0.0998123827392121 | 0.137319587628866 | 0.10181368507831802 | 0.09979838709677424 | 0.11362733412793001 | 0.10215605749486698 | 0.10994764397905804 | 0.106817554512835 | 0.10562685093780808 | 0.10162448054401207 | 0.10135375846099004 | 0.10512083826263308 |
| 6.0000000000000026E-2 | 0.10806754221388407 | 0.1665979381443302 | 0.10717230008244004 | 0.10559475806451608 | 0.10945570123162508 | 0.136036960985626 | 0.12514777909137 | 0.131934860612752 | 0.11829549193813811 | 0.1161692482055161 | 0.12166013537584602 | 0.12320432651681607 |
| 8.0000000000000043E-2 | 0.140337711069418 | 0.125773195876289 | 0.11253091508656203 | 0.140120967741935 | 0.12872467222884384 | 0.145790554414784 | 0.133930079378483 | 0.146287606955562 | 0.145771635406384 | 0.135058556856819 | 0.133594584966156 | 0.13351360486733113 |
| 0.1 | 0.1163227016885551 | 0.107216494845361 | 0.09851607584501255 | 0.11214717741935501 | 0.131704410011919 | 0.10985626283367604 | 0.11974328660699207 | 0.12061827215015204 | 0.125534715366897 | 0.11220249338874203 | 0.12397577484859308 | 0.12151428088558408 |
| 0.12000000000000002 | 0.0735459662288931 | 0.0870103092783505 | 0.07708161582852437 | 0.07510080645161292 | 0.08124751688518077 | 0.09240246406570839 | 0.07904070258402303 | 0.07673199006348334 | 0.06860809476801581 | 0.0768794862108047 | 0.08282864267901675 | 0.07706608078418128 |
| 0.14000000000000001 | 0.06904315196998125 | 0.059381443298969105 | 0.05935696619950545 | 0.05771169354838715 | 0.06853396901072711 | 0.06673511293634504 | 0.06536058098294215 | 0.06872757383383944 | 0.06153339914445542 | 0.06403475632791844 | 0.06180976131100824 | 0.07402399864796352 |
| 0.16000000000000006 | 0.047654784240150114 | 0.04783505154639184 | 0.05317394888705689 | 0.05342741935483875 | 0.05403257846642834 | 0.04620123203285419 | 0.053031582502955595 | 0.0488545404361027 | 0.05610398157288581 | 0.05213449187759736 | 0.06180976131100824 | 0.05357444651005578 |
| 0.18000000000000008 | 0.043527204502814304 | 0.0251546391752577 | 0.04492992580379233 | 0.0395665322580645 | 0.03833929280889953 | 0.039527720739219716 | 0.03833811856105394 | 0.0402980955009661 | 0.04540967423494576 | 0.042689837551945636 | 0.047381546134663326 | 0.043772181848909936 |
| 0.2 | 0.036022514071294615 | 0.02639175257731963 | 0.03709810387469082 | 0.029737903225806526 | 0.03178386968613433 | 0.028234086242299797 | 0.031413612565445025 | 0.03146563621308311 | 0.032082922013820334 | 0.03607857952398943 | 0.03562522265764162 | 0.0327868852459016 |
| 0.22000000000000006 | 0.027016885553470916 | 0.016907216494845403 | 0.0317394888705688 | 0.02721774193548391 | 0.026023043305522412 | 0.0251540041067762 | 0.02600912008106742 | 0.02428926304167821 | 0.023198420533070082 | 0.02436720816018131 | 0.02654079087994301 | 0.027378739225959126 |
| 0.24000000000000007 | 0.0165103189493433 | 0.014845360824742299 | 0.02638087386644681 | 0.018397177419354812 | 0.01807707588398891 | 0.015400410677618112 | 0.015875696672859298 | 0.014628760695556207 | 0.0184271141822968 | 0.01870041556479031 | 0.023334520840755296 | 0.017238465438566813 |
| 0.26 | 0.019512195121951202 | 0.009072164948453618 | 0.0218466611706513 | 0.016633064516129007 | 0.0145013905442988 | 0.011293634496919901 | 0.012666779260260119 | 0.0140767319900635 | 0.013984863441921706 | 0.014922553834529706 | 0.013003206270039199 | 0.014872401554842 |
| 0.28000000000000008 | 0.0135084427767355 | 0.006185567010309282 | 0.014839241549876299 | 0.0123487903225806 | 0.010131108462455306 | 0.008726899383983569 | 0.009288971457524069 | 0.006624344465912232 | 0.010365251727542001 | 0.011144692104268999 | 0.00997506234413966 | 0.009971269224269068 |
| 0.30000000000000016 | 0.012757973733583505 | 0.0037113402061855734 | 0.014014839241549901 | 0.007812500000000003 | 0.008144616607071917 | 0.006673511293634505 | 0.005066711704104046 | 0.006348330113165883 | 0.008555445870352105 | 0.010389119758216806 | 0.008371927324545786 | 0.009971269224269068 |
| 0.32000000000000017 | 0.007879924953095693 | 0.002886597938144333 | 0.01112943116240731 | 0.009828629032258066 | 0.006356773937226864 | 0.003593429158110882 | 0.00489782131396724 | 0.0035881865857024645 | 0.006416584402764074 | 0.009633547412164717 | 0.005165657285358033 | 0.0072671962142977905 |
| 0.34000000000000019 | 0.010881801125703605 | 0.00247422680412371 | 0.0123660346248969 | 0.008316532258064531 | 0.00476758045292014 | 0.0025667351129363427 | 0.0027022462421888212 | 0.0033121722329561114 | 0.005758473181967754 | 0.0066112580279561834 | 0.003918774492340582 | 0.005915159709312155 |
| 0.36000000000000015 | 0.006754221388367734 | 0.00247422680412371 | 0.00865622423742787 | 0.006552419354838712 | 0.005363528009535163 | 0.0015400410677618114 | 0.003040027022462424 | 0.0024841291747170926 | 0.0026324448831852587 | 0.0045334340763128105 | 0.004096900605628792 | 0.004056109514956901 |
| 0.38000000000000017 | 0.0033771106941838597 | 0.0020618556701030885 | 0.010305028854080803 | 0.0055443548387096794 | 0.0033770361541517716 | 0.0015400410677618114 | 0.001688903901368012 | 0.002760143527463433 | 0.002796972688384342 | 0.003400075557234613 | 0.003206270039187741 | 0.0025350684468480596 |
| 0.4 | 0.00375234521575985 | 0.0 | 0.0057708161582852415 | 0.003528225806451613 | 0.0021851410409217333 | 0.0005133470225872694 | 0.0015200135112312123 | 0.0024841291747170926 | 0.002303389272787103 | 0.002644503211182474 | 0.0024937655860349122 | 0.00304208213621768 |
### Chart: w.t. A2
| Category | N2_A2_40_30_02 | N2_A2_40_30_03 | N2_A2_40_30_04 | N2_REX_A2_01 | N2_REX_A2_02 | N2_REX_A2_03 | N2_REX_A2_04 | N2_REX_A2_08 | N2_REX_A2_09 | N2_REX_A2_10 |
|---|---|---|---|---|---|---|---|---|---|---|
| 0 | None | None | None | None | None | None | None | None | None | None |
| 2.0000000000000011E-2 | 0.09506618531889299 | 0.07582697201017813 | 0.112 | 0.08624971776924804 | 0.07398155265180631 | 0.08203483043079746 | 0.08148148148148145 | 0.09128289473684215 | 0.07434640522875824 | 0.0698795180722892 |
| 4.0000000000000022E-2 | 0.13838748495788208 | 0.10890585241730302 | 0.10857142857142908 | 0.11695642357191206 | 0.10491929285165302 | 0.110678276810266 | 0.12037037037037 | 0.10978618421052606 | 0.11540032679738596 | 0.11686746987951796 |
| 6.0000000000000026E-2 | 0.12996389891696708 | 0.12468193384223906 | 0.11542857142857108 | 0.12824565364642113 | 0.124519600307456 | 0.13015582034830392 | 0.13634259259259307 | 0.10567434210526302 | 0.138684640522876 | 0.1403614457831331 |
| 8.0000000000000043E-2 | 0.10830324909747302 | 0.1302798982188299 | 0.09371428571428571 | 0.11989162339128505 | 0.14584934665641813 | 0.13588450962419793 | 0.12615740740740708 | 0.11184210526315802 | 0.145220588235294 | 0.128313253012048 |
| 0.1 | 0.110709987966306 | 0.10687022900763404 | 0.0765714285714286 | 0.09866787085120801 | 0.10991544965411204 | 0.112511457378552 | 0.11273148148148109 | 0.08470394736842118 | 0.114174836601307 | 0.104819277108434 |
| 0.12000000000000002 | 0.07460890493381474 | 0.07531806615776084 | 0.06742857142857142 | 0.0871528561752088 | 0.0749423520368947 | 0.0815765352887259 | 0.08912037037037052 | 0.06455592105263168 | 0.0757761437908497 | 0.07228915662650597 |
| 0.14000000000000001 | 0.04211793020457283 | 0.0549618320610687 | 0.043428571428571414 | 0.0693158726574848 | 0.0710991544965411 | 0.0689734188817599 | 0.05393518518518517 | 0.0534539473684211 | 0.056781045751633986 | 0.063855421686747 |
| 0.16 | 0.036101083032491 | 0.0412213740458015 | 0.03200000000000002 | 0.0438022126890946 | 0.05169100691775558 | 0.04491292392300649 | 0.04004629629629633 | 0.045230263157894704 | 0.04309640522875822 | 0.04879518072289163 |
| 0.18000000000000008 | 0.0385078219013237 | 0.03765903307888042 | 0.03428571428571433 | 0.039286520659291 | 0.031706379707917016 | 0.0348304307974335 | 0.03680555555555561 | 0.04975328947368423 | 0.03778594771241833 | 0.0337349397590361 |
| 0.2 | 0.036101083032491 | 0.03715012722646314 | 0.028571428571428602 | 0.02551365996839019 | 0.0307455803228286 | 0.027497708524289628 | 0.02476851851851849 | 0.03741776315789472 | 0.030841503267973924 | 0.0307228915662651 |
| 0.22 | 0.024067388688327324 | 0.030534351145038188 | 0.022857142857142913 | 0.0223526755475277 | 0.02209838585703311 | 0.021539871677360222 | 0.0159722222222222 | 0.029194078947368397 | 0.0218545751633987 | 0.025301204819277112 |
| 0.24000000000000007 | 0.0144404332129964 | 0.02188295165394401 | 0.028571428571428602 | 0.01941747572815531 | 0.01633358954650271 | 0.016727772685609512 | 0.0175925925925926 | 0.022615131578947414 | 0.0163398692810458 | 0.01686746987951811 |
| 0.26 | 0.00962695547533093 | 0.019847328244274817 | 0.01600000000000001 | 0.015127568299842008 | 0.0151806302843966 | 0.016040329972502303 | 0.011111111111111101 | 0.015625 | 0.014297385620915 | 0.01686746987951811 |
| 0.28000000000000008 | 0.0120336943441637 | 0.014249363867684498 | 0.01600000000000001 | 0.0124181530819598 | 0.013835511145272907 | 0.0126031164069661 | 0.00810185185185185 | 0.016447368421052603 | 0.009191176470588246 | 0.009036144578313256 |
| 0.30000000000000016 | 0.00962695547533093 | 0.018829516539440202 | 0.008000000000000007 | 0.006999322646195534 | 0.008839354342813225 | 0.009853345554537126 | 0.006944444444444444 | 0.011513157894736807 | 0.009191176470588246 | 0.013855421686747011 |
| 0.32000000000000017 | 0.00962695547533093 | 0.008142493638676846 | 0.008000000000000007 | 0.005870399638744647 | 0.00691775557263643 | 0.004812098991750693 | 0.00601851851851852 | 0.0106907894736842 | 0.005514705882352942 | 0.007831325301204829 |
| 0.34 | 0.0036101083032491 | 0.00865139949109415 | 0.008000000000000007 | 0.0063219688417249925 | 0.005956956187548043 | 0.0032080659945004602 | 0.00439814814814815 | 0.004111842105263169 | 0.0028594771241830085 | 0.00662650602409639 |
| 0.36000000000000015 | 0.0024067388688327326 | 0.00458015267175573 | 0.003428571428571433 | 0.0036125536238428486 | 0.0036510376633359016 | 0.00206232813932172 | 0.00416666666666667 | 0.0049342105263157875 | 0.00245098039215686 | 0.0036144578313253013 |
| 0.38000000000000017 | 0.0 | 0.00305343511450382 | 0.004571428571428573 | 0.00203206141341161 | 0.0036510376633359016 | 0.001833180568285981 | 0.0020833333333333316 | 0.0032894736842105313 | 0.0030637254901960814 | 0.0036144578313253013 |
| 0.4 | 0.0012033694344163706 | 0.0020356234096692086 | 0.002285714285714292 | 0.0027094152178821426 | 0.0015372790161414301 | 0.0006874427131072414 | 0.0006944444444444446 | 0.0020559210526315827 | 0.0008169934640522886 | 0.00180722891566265 |
### Chart: w.t. A3
| Category | N2_A3_40_30_03 | N2_A3_40_30_2 | N2_REX_A3_01 | N2_REX_A3_02 | N2_REX_A3_03 | N2_REX_A3_04 | N2_REX_A3_05 | N2_REX_A3_07 | N2_REX_A3_08 | N2_REX_A3_09 |
|---|---|---|---|---|---|---|---|---|---|---|
| 0 | None | None | None | None | None | None | None | None | None | None |
| 2.0000000000000007E-2 | 0.07386363636363644 | 0.09362460989745885 | 0.08023598820059002 | 0.115884115884116 | 0.11074144486692003 | 0.14408361328853994 | 0.13799621928166406 | 0.10706638115631703 | 0.101966496722505 | 0.104684788895315 |
| 4.0000000000000015E-2 | 0.12448347107438003 | 0.12706197057512306 | 0.11622418879056003 | 0.14085914085914106 | 0.13925855513308 | 0.161254199328108 | 0.14366729678638906 | 0.12419700214132802 | 0.13401310997815 | 0.127241179872759 |
| 6.0000000000000019E-2 | 0.12448347107438003 | 0.144003566651806 | 0.14159292035398197 | 0.132367632367632 | 0.121197718631179 | 0.15528182157521506 | 0.12161310649023305 | 0.13888039155705106 | 0.11798980335032797 | 0.125216888374783 |
| 8.0000000000000029E-2 | 0.09917355371900828 | 0.11591618368256798 | 0.145722713864307 | 0.09940059940059942 | 0.111692015209125 | 0.102650242627846 | 0.08443604284814113 | 0.10676047721015604 | 0.09104151493080838 | 0.10121457489878502 |
| 0.1 | 0.0888429752066116 | 0.10075791350869398 | 0.0985250737463128 | 0.0669330669330669 | 0.0760456273764259 | 0.0742814483016051 | 0.06490233144297423 | 0.08993576017130624 | 0.07319737800437 | 0.09861191440138808 |
| 0.12000000000000002 | 0.0681818181818182 | 0.0784663397235845 | 0.08082595870206495 | 0.051948051948052 | 0.0560836501901141 | 0.051511758118700984 | 0.04788909892879652 | 0.06485163658611202 | 0.05644573925710122 | 0.06824754193175249 |
| 0.14000000000000001 | 0.05733471074380173 | 0.054391440035666525 | 0.06253687315634222 | 0.046453546453546504 | 0.0413498098859316 | 0.032848077640910814 | 0.032136105860113416 | 0.0526154787396757 | 0.0411507647487254 | 0.05639097744360902 |
| 0.16 | 0.04442148760330582 | 0.050378956754346914 | 0.04306784660766958 | 0.0314685314685315 | 0.040399239543726234 | 0.032474804031355005 | 0.0315059861373661 | 0.0403793208932395 | 0.0386016023306628 | 0.037015615962984416 |
| 0.18000000000000005 | 0.0392561983471074 | 0.0396790013374944 | 0.028908554572271393 | 0.0324675324675325 | 0.026615969581749013 | 0.0246360582306831 | 0.0220541902961563 | 0.03548485775466501 | 0.029861616897305206 | 0.034412955465587 |
| 0.2 | 0.0335743801652893 | 0.0365581810075791 | 0.02654867256637171 | 0.035964035964035995 | 0.031844106463878315 | 0.013064576334453206 | 0.027725267800882202 | 0.02600183542367702 | 0.029133284777858707 | 0.028050896471949114 |
| 0.22 | 0.03047520661157022 | 0.0182790905037896 | 0.0171091445427729 | 0.022977022977023018 | 0.023764258555133092 | 0.017543859649122813 | 0.019533711405167006 | 0.019271948608137007 | 0.022942461762563707 | 0.023134759976865198 |
| 0.24000000000000005 | 0.021694214876033114 | 0.012929112795363401 | 0.019469026548672608 | 0.017982017982018 | 0.014733840304182504 | 0.010451661067562505 | 0.013232514177693798 | 0.015601101254206209 | 0.02148579752367082 | 0.0179294389820706 |
| 0.26 | 0.019111570247933914 | 0.011591618368256803 | 0.018879056047197605 | 0.013486513486513505 | 0.0180608365019011 | 0.007838745800671893 | 0.020163831127914308 | 0.0134597736310798 | 0.02148579752367082 | 0.0133024869866975 |
| 0.28000000000000008 | 0.016528925619834708 | 0.0089166295140437 | 0.007079646017699114 | 0.011488511488511505 | 0.0128326996197719 | 0.00522583053378126 | 0.015752993068683 | 0.007035790761700834 | 0.0182083029861617 | 0.01417004048583 |
| 0.3000000000000001 | 0.010330578512396701 | 0.00534997770842621 | 0.010619469026548698 | 0.006993006993006992 | 0.0104562737642586 | 0.00373273609555804 | 0.006301197227473222 | 0.007953502600183543 | 0.012381646030589898 | 0.010121457489878501 |
| 0.32000000000000012 | 0.0129132231404959 | 0.008470798038341516 | 0.0058997050147492625 | 0.00999000999001 | 0.006653992395437262 | 0.00149309443822322 | 0.005040957781978582 | 0.007953502600183543 | 0.014930808448652605 | 0.008386350491613653 |
| 0.34 | 0.005681818181818184 | 0.004012483281319662 | 0.004719764011799412 | 0.003996003996004 | 0.006178707224334602 | 0.00149309443822322 | 0.0018903591682419712 | 0.004588559192413584 | 0.010924981791697005 | 0.00809716599190283 |
| 0.3600000000000001 | 0.005165289256198348 | 0.006687472135532773 | 0.004719764011799412 | 0.002497502497502501 | 0.006653992395437262 | 0.00186636804777902 | 0.003150598613736611 | 0.003059039461609051 | 0.0040058266569555695 | 0.004048582995951422 |
| 0.38000000000000012 | 0.004132231404958682 | 0.005795809184128403 | 0.0017699115044247805 | 0.001998001998002001 | 0.003802281368821292 | 0.00037327360955580414 | 0.0006301197227473222 | 0.00336494340776996 | 0.005098324836125268 | 0.002024291497975711 |
| 0.4 | 0.0015495867768595005 | 0.00534997770842621 | 0.00294985250737463 | 0.0009990009990009999 | 0.0 | 0.0007465472191116092 | 0.00126023944549464 | 0.00183542367696543 | 0.0010924981791697005 | 0.002602660497397341 |
### Chart: w.t. A5
| Category | N2_A5_001 | N2_A5_003 | N2_A5_004 | N2_A5_40_30_01 | N2_A5_40_30_02 | N2_A5_40_30_04 | N2_REX_A5_01 | N2_REX_A5_02 | N2_REX_A5_04 | N2_REX_A5_05 | N2_REX_A5_06 | N2_REX_A5_08 | N2_REX_A5_09 |
|---|---|---|---|---|---|---|---|---|---|---|---|---|---|
| 0 | None | None | None | None | None | None | None | None | None | None | None | None | None |
| 2.0000000000000011E-2 | 0.0684371807967314 | 0.128865979381443 | 0.09266666666666677 | 0.08450704225352122 | 0.13021939136588812 | 0.1121212121212121 | 0.1572438162544171 | 0.08972460763991719 | 0.11867152693398111 | 0.105008077544426 | 0.1335637963544941 | 0.128205128205128 | 0.106029106029106 |
| 4.0000000000000022E-2 | 0.07150153217568947 | 0.1039518900343639 | 0.0766666666666667 | 0.139280125195618 | 0.11252653927813211 | 0.12272727272727312 | 0.11778563015312107 | 0.110453064850459 | 0.14337788578371788 | 0.138933764135703 | 0.15210559396605897 | 0.14851814851814918 | 0.13305613305613312 |
| 6.0000000000000032E-2 | 0.05311542390194086 | 0.0756013745704467 | 0.08666666666666681 | 0.10798122065727705 | 0.10898796886058 | 0.09696969696969707 | 0.0859835100117785 | 0.111045306485046 | 0.12515188335358382 | 0.135702746365105 | 0.1282212445003141 | 0.12987012987013 | 0.16008316008316 |
| 8.0000000000000043E-2 | 0.055158324821246274 | 0.049828178694158065 | 0.08066666666666678 | 0.12676056338028197 | 0.0778485491861288 | 0.08863636363636374 | 0.05712603062426382 | 0.09712762807225357 | 0.100445524503848 | 0.1147011308562201 | 0.08390949088623517 | 0.10256410256410307 | 0.13513513513513511 |
| 0.1 | 0.038815117466802926 | 0.0335051546391753 | 0.0666666666666667 | 0.09389671361502352 | 0.046709129511677286 | 0.08333333333333334 | 0.036513545347467605 | 0.06781166716020146 | 0.0729040097205346 | 0.08885298869143793 | 0.0688246385920804 | 0.07692307692307691 | 0.10187110187110206 |
| 0.12000000000000002 | 0.0408580183861083 | 0.02663230240549834 | 0.0593333333333334 | 0.05164319248826284 | 0.0382165605095541 | 0.053030303030303 | 0.0241460541813899 | 0.050044418122594 | 0.042932361279870414 | 0.05815831987075928 | 0.05279698302954134 | 0.0486180486180486 | 0.0644490644490645 |
| 0.14000000000000001 | 0.0326864147088866 | 0.0309278350515464 | 0.04333333333333336 | 0.0469483568075117 | 0.032554847841472015 | 0.0439393939393939 | 0.03415783274440521 | 0.04530648504589878 | 0.03888213851761853 | 0.0565428109854604 | 0.03708359522313013 | 0.0406260406260406 | 0.0561330561330561 |
| 0.16 | 0.0326864147088866 | 0.020618556701030875 | 0.046 | 0.043818466353677636 | 0.019108280254777114 | 0.046969696969697 | 0.025323910482921142 | 0.04501036422860534 | 0.040097205346294004 | 0.032310177705977425 | 0.03519798868636083 | 0.0303030303030303 | 0.051975051975051985 |
| 0.1800000000000001 | 0.030643513789581227 | 0.02663230240549834 | 0.0386666666666667 | 0.0422535211267606 | 0.03750884642604395 | 0.03106060606060611 | 0.025323910482921142 | 0.0423452768729642 | 0.02875658161198866 | 0.02907915993537961 | 0.025455688246385888 | 0.023643023643023616 | 0.024948024948024887 |
| 0.2 | 0.0449438202247191 | 0.01632302405498282 | 0.0326666666666667 | 0.028169014084506998 | 0.0339702760084926 | 0.0303030303030303 | 0.02944640753828031 | 0.033757773171454 | 0.028351559335763474 | 0.024232633279483 | 0.019798868636077917 | 0.0166500166500167 | 0.020790020790020798 |
| 0.22 | 0.0296220633299285 | 0.018900343642611714 | 0.04066666666666671 | 0.028169014084506998 | 0.02406227883934888 | 0.024242424242424197 | 0.013545347467609001 | 0.037015102161682 | 0.020656136087484806 | 0.032310177705977425 | 0.014770584538026401 | 0.014319014319014299 | 0.018711018711018716 |
| 0.2400000000000001 | 0.03983656792645563 | 0.0128865979381443 | 0.0326666666666667 | 0.0250391236306729 | 0.021231422505307924 | 0.0189393939393939 | 0.011189634864546499 | 0.03938406870002961 | 0.021871202916160442 | 0.024232633279483 | 0.016027655562539305 | 0.010323010323010301 | 0.008316008316008336 |
| 0.26 | 0.041879468845760985 | 0.0197594501718213 | 0.026666666666666717 | 0.0203442879499218 | 0.024769992922859217 | 0.025757575757575812 | 0.01531213191990581 | 0.0263547527391176 | 0.013770757391656509 | 0.024232633279483 | 0.008170961659333752 | 0.00666000666000666 | 0.0041580041580041634 |
| 0.28000000000000008 | 0.043922369765066395 | 0.01632302405498282 | 0.0326666666666667 | 0.015649452269170614 | 0.019815994338287315 | 0.018181818181818212 | 0.012367491166077701 | 0.0225051821143026 | 0.011745646010530601 | 0.014539579967689805 | 0.006285355122564437 | 0.00732600732600733 | 0.0103950103950104 |
| 0.30000000000000021 | 0.043922369765066395 | 0.020618556701030875 | 0.034 | 0.014084507042253508 | 0.016277423920736 | 0.010606060606060601 | 0.00824499411071849 | 0.023689665383476523 | 0.009720534629404635 | 0.008077544426494358 | 0.006599622878692654 | 0.0036630036630036617 | 0.00207900207900208 |
| 0.32000000000000023 | 0.0408580183861083 | 0.015463917525773198 | 0.0173333333333333 | 0.012519561815336503 | 0.009907997169143675 | 0.007575757575757584 | 0.00647820965842168 | 0.013325436778205501 | 0.008100445524503855 | 0.0016155088852988701 | 0.00345694531741043 | 0.00333000333000333 | 0.0 |
| 0.34 | 0.04290091930541374 | 0.00945017182130584 | 0.014 | 0.012519561815336503 | 0.0106157112526539 | 0.00833333333333333 | 0.001177856301531212 | 0.00858750370151022 | 0.003240178209801544 | 0.004846526655896613 | 0.002199874292897554 | 0.00233100233100233 | 0.0041580041580041634 |
| 0.36000000000000021 | 0.027579162410623165 | 0.007731958762886606 | 0.0173333333333333 | 0.009389671361502353 | 0.006369426751592364 | 0.00681818181818182 | 0.004711425206124852 | 0.00799526206692331 | 0.0028351559335763484 | 0.0 | 0.0018856065367693312 | 0.001332001332001331 | 0.00207900207900208 |
| 0.38000000000000023 | 0.022471910112359654 | 0.004295532646048109 | 0.015333333333333301 | 0.00469483568075117 | 0.00141542816702052 | 0.00303030303030303 | 0.001177856301531212 | 0.0035534498075214735 | 0.00121506682867558 | 0.0 | 0.0012570710245128909 | 0.0 | 0.0 |
| 0.4 | 0.022471910112359654 | 0.0008591065292096228 | 0.009333333333333338 | 0.00469483568075117 | 0.002830856334041048 | 0.0015151515151515217 | 0.00235571260306243 | 0.0014806040864672801 | 0.00121506682867558 | 0.0016155088852988701 | 0.0 | 0.0006660006660006661 | 0.0 |
### Chart: unc-25 L4
| Category | unc-25_L4_06001 | unc-25_L4_06002 | unc-25_L4_06003 | unc-25_L4_06004 | unc-25_L4_06005 | unc-25_L4_06006 | unc-25_L4_06007 | unc-25_L4_06008 | unc-25_L4_06009 | unc-25_L4_06011 | unc-25_L4_06012 | unc-25_L4_06013 | unc-25_L4_06014 | unc-25_L4_06015 | unc-25_L4_06016 | unc-25_L4_06017 | unc-25_L4_06018 | unc-25_L4_06019 |
|---|---|---|---|---|---|---|---|---|---|---|---|---|---|---|---|---|---|---|
| 0 | None | None | None | None | None | None | None | None | None | None | None | None | None | None | None | None | None | None |
| 2.0000000000000007E-2 | 0.06490455212922172 | 0.07836153161175423 | 0.07972440944881894 | 0.08324125230202578 | None | 0.10400000000000002 | 0.0770502645502645 | 0.09982638888888895 | 0.12109375000000006 | 0.0756556825823806 | 0.05923043666234333 | 0.0778508771929825 | 0.0664760543245175 | 0.08219178082191773 | 0.08066666666666675 | 0.08087504143188602 | 0.1276595744680849 | 0.0656565656565657 |
| 4.0000000000000015E-2 | 0.10014684287812003 | 0.104778866132383 | 0.09104330708661419 | 0.10349907918968702 | None | 0.12000000000000002 | 0.10152116402116403 | 0.10590277777777803 | 0.12109375000000006 | 0.09952925353059851 | 0.0912235192390834 | 0.09539473684210523 | 0.09721229449606854 | 0.12328767123287702 | 0.105333333333333 | 0.104076897580378 | 0.12056737588652502 | 0.08922558922558921 |
| 6.0000000000000019E-2 | 0.105433186490455 | 0.12318195310181103 | 0.11466535433070903 | 0.113812154696133 | None | 0.072 | 0.12301587301587302 | 0.12065972222222203 | 0.11328125000000003 | 0.11264290517821103 | 0.12019022913964503 | 0.10964912280701802 | 0.11686919228020003 | 0.0684931506849315 | 0.14200000000000004 | 0.12495856811402095 | 0.0780141843971631 | 0.11560044893378203 |
| 8.0000000000000029E-2 | 0.13186490455212904 | 0.11932324131789802 | 0.09990157480314958 | 0.119705340699816 | None | 0.06400000000000003 | 0.12599206349206307 | 0.11024305555555602 | 0.07031250000000003 | 0.13315400134499 | 0.11197578901859102 | 0.12225877192982502 | 0.12294496068620403 | 0.041095890410958895 | 0.10400000000000002 | 0.12727875372886993 | 0.0709219858156028 | 0.10325476992143705 |
| 0.1 | 0.115418502202643 | 0.11279311368358604 | 0.09547244094488193 | 0.09907918968692449 | None | 0.06400000000000003 | 0.116402116402116 | 0.078125 | 0.07421875000000001 | 0.09952925353059851 | 0.10073497622135805 | 0.09375000000000004 | 0.10686204431737 | 0.027397260273972608 | 0.0973333333333333 | 0.11037454424925403 | 0.0709219858156028 | 0.10662177328844008 |
| 0.12000000000000002 | 0.0740088105726872 | 0.07301869991095283 | 0.0639763779527559 | 0.058563535911602224 | None | 0.056 | 0.0621693121693122 | 0.0651041666666667 | 0.07031250000000003 | 0.0716207128446537 | 0.0786856895806312 | 0.07839912280701751 | 0.07576840600428883 | 0.054794520547945244 | 0.074 | 0.05767318528339408 | 0.049645390070921995 | 0.0656565656565657 |
| 0.14000000000000001 | 0.057268722466960395 | 0.06411398040961713 | 0.06643700787401582 | 0.05193370165745862 | None | 0.016000000000000007 | 0.05555555555555558 | 0.04600694444444441 | 0.042968750000000014 | 0.06691324815063891 | 0.06960657155209685 | 0.06140350877192978 | 0.0714796283059328 | 0.027397260273972608 | 0.05466666666666672 | 0.05601590984421613 | 0.042553191489361715 | 0.0454545454545455 |
| 0.16 | 0.05198237885462563 | 0.05936479667557142 | 0.04921259842519692 | 0.044935543278084696 | None | 0.016000000000000007 | 0.04563492063492062 | 0.0364583333333333 | 0.054687500000000014 | 0.060860793544048435 | 0.05101599654128842 | 0.055921052631578885 | 0.058255897069335184 | 0.0 | 0.048 | 0.0391117003646006 | 0.049645390070921995 | 0.04264870931537599 |
| 0.18000000000000005 | 0.047283406754772404 | 0.04927278124072423 | 0.036417322834645716 | 0.040883977900552516 | None | 0.024 | 0.04728835978835976 | 0.0321180555555556 | 0.03515625 | 0.0464021519838601 | 0.04366623432771292 | 0.0482456140350877 | 0.05432451751250892 | 0.08219178082191773 | 0.034 | 0.04043752071594301 | 0.028368794326241082 | 0.0420875420875421 |
| 0.2 | 0.0422907488986784 | 0.034431582071831404 | 0.033956692913385794 | 0.0331491712707182 | None | 0.048 | 0.036706349206349215 | 0.023437500000000007 | 0.027343750000000007 | 0.04135843981170143 | 0.04236921746649372 | 0.040570175438596486 | 0.0339528234453181 | 0.0136986301369863 | 0.032000000000000015 | 0.03314550878355981 | 0.0 | 0.0375982042648709 |
| 0.22 | 0.0290748898678414 | 0.03383793410507571 | 0.0334645669291339 | 0.03867403314917131 | None | 0.016000000000000007 | 0.03240740740740741 | 0.02951388888888889 | 0.03125 | 0.026563550773369207 | 0.029831387808041513 | 0.03125 | 0.032523230879199415 | 0.0136986301369863 | 0.030666666666666707 | 0.0291680477295326 | 0.0212765957446809 | 0.0325476992143659 |
| 0.24000000000000005 | 0.026431718061674027 | 0.0225586227367171 | 0.026574803149606308 | 0.025046040515653813 | None | 0.024 | 0.01984126984126981 | 0.0243055555555556 | 0.019531250000000003 | 0.027908540685944918 | 0.026372676178123614 | 0.0246710526315789 | 0.020729092208720507 | 0.0136986301369863 | 0.026666666666666707 | 0.024859131587669916 | 0.007092198581560282 | 0.0303030303030303 |
| 0.26 | 0.017327459618208512 | 0.019887206886316403 | 0.027559055118110208 | 0.023941068139963207 | None | 0.0 | 0.0165343915343915 | 0.03559027777777781 | 0.015625 | 0.015803631472764 | 0.01859057501080851 | 0.021929824561403508 | 0.0175125089349535 | 0.0 | 0.012 | 0.023201856148491892 | 0.007092198581560282 | 0.02132435465768799 |
| 0.28000000000000008 | 0.018208516886931002 | 0.0121697833184921 | 0.0211614173228346 | 0.021731123388582 | None | 0.008000000000000005 | 0.016865079365079413 | 0.026041666666666713 | 0.0 | 0.01546738399462 | 0.021184608733246894 | 0.0213815789473684 | 0.014295925661186601 | 0.0136986301369863 | 0.014666666666666701 | 0.02121312562147831 | 0.028368794326241082 | 0.017396184062850702 |
| 0.3000000000000001 | 0.013215859030837008 | 0.008311071534579995 | 0.014271653543307101 | 0.019521178637200712 | None | 0.0 | 0.0115740740740741 | 0.0164930555555556 | 0.00390625 | 0.0107599193006052 | 0.0190229139645482 | 0.0142543859649123 | 0.010721944245889901 | 0.0 | 0.012666666666666701 | 0.0155783891282731 | 0.014184397163120598 | 0.0168350168350168 |
| 0.32000000000000012 | 0.009397944199706318 | 0.006530127634312853 | 0.0127952755905512 | 0.013627992633517504 | None | 0.0 | 0.0109126984126984 | 0.008680555555555565 | 0.015625 | 0.0097511768661735 | 0.00605274535235625 | 0.008223684210526327 | 0.010721944245889901 | 0.0136986301369863 | 0.010000000000000004 | 0.012263838249917114 | 0.0 | 0.010662177328844 |
| 0.34 | 0.00646108663729809 | 0.00267141585040071 | 0.006889763779527564 | 0.01215469613259669 | None | 0.0 | 0.007605820105820113 | 0.008680555555555565 | 0.0078125 | 0.008406186953597856 | 0.00994379593601383 | 0.010964912280701799 | 0.006433166547533952 | 0.0 | 0.010666666666666701 | 0.008949287371561155 | 0.007092198581560282 | 0.00897867564534231 |
| 0.3600000000000001 | 0.00499265785609398 | 0.0038587117839121417 | 0.00541338582677165 | 0.006998158379373852 | None | 0.008000000000000005 | 0.009920634920634924 | 0.010416666666666699 | 0.00390625 | 0.003698722259583052 | 0.00691742325983571 | 0.008223684210526327 | 0.005003573981415302 | 0.027397260273972608 | 0.002666666666666671 | 0.00861783228372556 | 0.0 | 0.010662177328844 |
| 0.38000000000000012 | 0.00499265785609398 | 0.002077767883645001 | 0.004921259842519692 | 0.004419889502762432 | None | 0.0 | 0.002645502645502652 | 0.0017361111111111108 | 0.0 | 0.0016812373907195701 | 0.004755728491137052 | 0.003289473684210531 | 0.002859185132237311 | 0.0 | 0.003333333333333331 | 0.00430891614186278 | 0.0 | 0.00561167227833894 |
| 0.4 | 0.004111600587371512 | 0.0017809439002671405 | 0.00393700787401575 | 0.00331491712707182 | None | 0.0 | 0.0009920634920634924 | 0.0017361111111111108 | 0.00390625 | 0.0033624747814391402 | 0.0030263726761781207 | 0.00219298245614035 | 0.002144388849177982 | 0.0 | 0.002666666666666671 | 0.003977461054027182 | 0.0 | 0.00897867564534231 |
### Chart: unc-25 A1
| Category | control_unc-25_A1_001 | control_unc-25_A1_002 | control_unc-25_A1_003 | control_unc-25_A1_004 | control_unc-25_A1_005 | control_unc-25_A1_006 | control_unc-25_A1_007 | control_unc-25_A1_008 | unc-25_A1_M101 | unc-25_A1_M102 | unc-25_A1_M104 | unc-25_A1_M106 | unc-25_REX_A1_01 | unc-25_REX_A1_02 | unc-25_REX_A1_04 | unc-25_REX_A1_06 | unc-25_REX_A1_07 | unc-25_REX_A1_09 |
|---|---|---|---|---|---|---|---|---|---|---|---|---|---|---|---|---|---|---|
| 0 | None | None | None | None | None | None | None | None | None | None | None | None | None | None | None | None | None | None |
| 2.0000000000000011E-2 | 0.08932268559597754 | 0.07609805924412676 | 0.075077399380805 | 0.0710951526032316 | 0.06959152798789715 | 0.0636140135218193 | 0.07224025974025969 | 0.07123854566952403 | 0.06064474944142986 | 0.06466809421841548 | 0.08546712802768171 | 0.06792918896665286 | 0.07489775051124747 | 0.08104238258877434 | 0.08441558441558435 | 0.09035369774919615 | 0.0769528228924981 | 0.0668806848582131 |
| 4.0000000000000022E-2 | 0.11180124223602506 | 0.096782431052094 | 0.10371517027863815 | 0.09982046678635562 | 0.091780131114473 | 0.08727719729563613 | 0.09415584415584437 | 0.08956547443097848 | 0.09383977018831798 | 0.09593147751606007 | 0.112456747404844 | 0.08645533141210375 | 0.0828220858895705 | 0.105670103092784 | 0.0928030303030303 | 0.11414790996784598 | 0.09280742459396753 | 0.08293204922418408 |
| 6.0000000000000032E-2 | 0.117420881396037 | 0.10852911133810007 | 0.1099071207430341 | 0.10520646319569102 | 0.10993444276349011 | 0.11186232329440694 | 0.0974025974025973 | 0.0931126219331954 | 0.115863389722311 | 0.10963597430406906 | 0.11972318339100307 | 0.0922190201729107 | 0.09815950920245409 | 0.117124856815578 | 0.1087662337662342 | 0.109967845659164 | 0.11562258313998502 | 0.10299625468164811 |
| 8.0000000000000043E-2 | 0.10854776693286 | 0.11644535240040906 | 0.111455108359133 | 0.11741472172351912 | 0.11396873424104906 | 0.12476951444376205 | 0.09943181818181825 | 0.10966597694354112 | 0.11841685285668706 | 0.113490364025696 | 0.11972318339100307 | 0.09057225195553728 | 0.10761758691206498 | 0.10681557846506302 | 0.102813852813853 | 0.09774919614147921 | 0.09590100541376645 | 0.09202782236490108 |
| 0.1 | 0.09553386572020124 | 0.10776302349336107 | 0.093266253869969 | 0.100538599640934 | 0.1122037317196171 | 0.11862323294406911 | 0.08360389610389628 | 0.107301211942063 | 0.10437280561761902 | 0.09122055674518205 | 0.09619377162629758 | 0.0959242486620009 | 0.0846114519427403 | 0.0959335624284078 | 0.08360389610389628 | 0.0736334405144694 | 0.0924207269914927 | 0.0890850722311397 |
| 0.12000000000000002 | 0.0698018337769891 | 0.0735444330949949 | 0.07546439628482973 | 0.0703770197486535 | 0.08421583459404952 | 0.0759065765212047 | 0.0641233766233766 | 0.07064735441915454 | 0.09064794127034795 | 0.06338329764453968 | 0.0761245674740484 | 0.0753396459448334 | 0.06646216768916162 | 0.06386025200458191 | 0.059253246753246835 | 0.05112540192926041 | 0.059938128383603996 | 0.06072766185125746 |
| 0.14000000000000001 | 0.060928719313812496 | 0.0638406537282942 | 0.07043343653250778 | 0.05206463195691212 | 0.06227937468482096 | 0.0611555009219422 | 0.053165584415584395 | 0.053207212533254465 | 0.07085860197893395 | 0.05695931477516055 | 0.0678200692041522 | 0.0613421160971593 | 0.0605828220858896 | 0.060423825887743435 | 0.052218614718614734 | 0.0382636655948553 | 0.05143078112915701 | 0.05377207062600324 |
| 0.16 | 0.044661342797988765 | 0.052860061287027665 | 0.0588235294117647 | 0.052423698384201134 | 0.061018658598083704 | 0.05439459127228037 | 0.04991883116883128 | 0.047590895654744336 | 0.052026811362911 | 0.04668094218415413 | 0.057093425605536395 | 0.0494030465212021 | 0.0480572597137014 | 0.038659793814433 | 0.0416666666666667 | 0.03536977491961413 | 0.04447022428460942 | 0.047084002140181914 |
| 0.1800000000000001 | 0.05146406388642411 | 0.0490296220633299 | 0.055727554179566624 | 0.041292639138240675 | 0.05194150277357533 | 0.04978488014751084 | 0.048701298701298704 | 0.047590895654744336 | 0.05458027449728693 | 0.0453961456102784 | 0.044636678200692004 | 0.043227665706051875 | 0.04959100204498981 | 0.0403780068728522 | 0.03652597402597404 | 0.036655948553054755 | 0.034416086620263 | 0.04440877474585343 |
| 0.2 | 0.03667553978112985 | 0.03855975485188968 | 0.037925696594427204 | 0.03698384201077204 | 0.0403429147755925 | 0.0368776889981561 | 0.041396103896103924 | 0.0416789831510494 | 0.034152569422279 | 0.03940042826552461 | 0.039100346020761206 | 0.0321119802387814 | 0.03629856850715753 | 0.03264604810996564 | 0.0327380952380952 | 0.0315112540192926 | 0.03944315545243623 | 0.031300160513643725 |
| 0.22 | 0.03223898254954163 | 0.0326864147088866 | 0.0297987616099071 | 0.0351885098743268 | 0.03328290468986383 | 0.043331284572833424 | 0.0393668831168831 | 0.027785988767366224 | 0.033833386530482 | 0.0312633832976445 | 0.031487889273356405 | 0.0341704405104981 | 0.031186094069529713 | 0.030068728522336798 | 0.033008658008657994 | 0.02733118971061092 | 0.0348027842227378 | 0.031300160513643725 |
| 0.2400000000000001 | 0.028098195800059202 | 0.0265577119509704 | 0.0286377708978328 | 0.0333931777378815 | 0.0237014624306606 | 0.03042409342347881 | 0.040178571428571404 | 0.028968371268105197 | 0.03223747207149703 | 0.03169164882226984 | 0.02179930795847753 | 0.02840675174969119 | 0.02709611451942742 | 0.02205040091638029 | 0.024350649350649397 | 0.0286173633440514 | 0.02900232018561482 | 0.0321027287319422 |
| 0.26 | 0.01952085181898853 | 0.020684371807967318 | 0.019736842105263205 | 0.023339317773788212 | 0.01840645486636412 | 0.02427781192378612 | 0.0251623376623377 | 0.02187407626367133 | 0.019150973507820003 | 0.02141327623126342 | 0.015916955017301 | 0.03087690407575133 | 0.026329243353783206 | 0.02205040091638029 | 0.024621212121212134 | 0.024758842443729934 | 0.023975251353441588 | 0.0296950240770466 |
| 0.28000000000000008 | 0.013013901212659001 | 0.01481103166496421 | 0.018575851393188923 | 0.0226211849192101 | 0.0196671709531014 | 0.0202827289489859 | 0.02069805194805187 | 0.02571681939107299 | 0.022981168209384017 | 0.023554603854389684 | 0.018339100346020803 | 0.02305475504322768 | 0.026840490797546 | 0.020332187857961117 | 0.0219155844155844 | 0.029903536977492 | 0.022428460943542183 | 0.0267522739432852 |
| 0.30000000000000021 | 0.012422360248447213 | 0.0112359550561798 | 0.00735294117647059 | 0.0226211849192101 | 0.0143721633888048 | 0.01352181929932392 | 0.023133116883116926 | 0.0150753768844221 | 0.010213852537503999 | 0.018843683083511805 | 0.0110726643598616 | 0.025113215314944412 | 0.021216768916155402 | 0.0197594501718213 | 0.020562770562770602 | 0.0228295819935691 | 0.017401392111368902 | 0.02487961476725522 |
| 0.32000000000000023 | 0.0082815734989648 | 0.009193054136874367 | 0.010448916408668699 | 0.014003590664272909 | 0.011598587997982909 | 0.008604794099569773 | 0.025974025974026017 | 0.017440141885900103 | 0.011809766996489 | 0.0184154175588865 | 0.007266435986159173 | 0.019349526554137512 | 0.017638036809816 | 0.014891179839633414 | 0.022997835497835527 | 0.0189710610932476 | 0.0127610208816705 | 0.016318887105404 |
| 0.34 | 0.007394262052647154 | 0.0071501532175689475 | 0.008900928792569667 | 0.009694793536804318 | 0.005295007564296524 | 0.004917025199754157 | 0.01379870129870131 | 0.014779781259237412 | 0.007979572294925 | 0.0141327623126338 | 0.005190311418685123 | 0.016467682173734 | 0.016615541922290398 | 0.012313860252004599 | 0.0140692640692641 | 0.012540192926044993 | 0.011214230471771091 | 0.015516318887105399 |
| 0.36000000000000021 | 0.0053238686779059395 | 0.0056179775280898875 | 0.0046439628482972065 | 0.007540394973070025 | 0.0068078668683812385 | 0.004302397049784881 | 0.00974025974025974 | 0.010937038131835601 | 0.005745292052345994 | 0.0107066381156317 | 0.00380622837370242 | 0.0119390695759572 | 0.014826175869120712 | 0.00687285223367698 | 0.013528138528138505 | 0.013504823151125401 | 0.01198762567672081 | 0.012573568753343999 |
| 0.38000000000000023 | 0.002661934338952976 | 0.0056179775280898875 | 0.003869969040247682 | 0.00538599640933573 | 0.003530005042864354 | 0.002765826674861712 | 0.006899350649350653 | 0.007389890629618686 | 0.003830194701564004 | 0.009850107066381168 | 0.0031141868512110757 | 0.012350761630300501 | 0.011758691206544 | 0.006013745704467356 | 0.0110930735930736 | 0.006752411575562702 | 0.008507347254447028 | 0.0120385232744783 |
| 0.4 | 0.0023661638568470925 | 0.00408580183861083 | 0.002708978328173372 | 0.004308797127468581 | 0.003025718608169443 | 0.003073140749846344 | 0.007305194805194814 | 0.00413833875258646 | 0.002553463134376 | 0.00642398286937901 | 0.00242214532871972 | 0.006587072869493624 | 0.005623721881390592 | 0.00486827033218786 | 0.007305194805194814 | 0.006752411575562702 | 0.007733952049497291 | 0.00989834135901552 |
### Chart: unc-25 A2
| Category | unc-25_A2_40_30_01 | unc-25_A2_40_30_02 | unc-25_A2_40_30_03 | unc-25_REX_A2_03 | unc-25_REX_A2_04 | unc-25_REX_A2_05 | unc-25_REX_A2_06 | unc-25_REX_A2_08 | unc-25_REX_A2_09 | unc-25_REX_A2_10 |
|---|---|---|---|---|---|---|---|---|---|---|
| 0 | None | None | None | None | None | None | None | None | None | None |
| 2.0000000000000007E-2 | 0.08695652173913045 | 0.0768491834774256 | 0.08000000000000003 | 0.07310493660642031 | 0.09374064091045224 | 0.1 | 0.0834890965732087 | 0.0977306002928258 | 0.0949227373068433 | 0.08762626262626266 |
| 4.0000000000000015E-2 | 0.10111223458038403 | 0.085014409221902 | 0.11125 | 0.0882114917723226 | 0.100928421683139 | 0.10375000000000002 | 0.08598130841121501 | 0.11493411420205002 | 0.10871964679911703 | 0.116919191919192 |
| 6.0000000000000019E-2 | 0.12082912032355904 | 0.09990393852065325 | 0.08625000000000001 | 0.09495548961424334 | 0.10272536687631004 | 0.11093749999999995 | 0.0984423676012461 | 0.09956076134699854 | 0.11920529801324503 | 0.11540404040404002 |
| 8.0000000000000029E-2 | 0.09807886754297275 | 0.09269932756964462 | 0.11 | 0.10628540598867009 | 0.09134471398622343 | 0.08937500000000002 | 0.09096573208722744 | 0.09077598828696934 | 0.09271523178807953 | 0.09393939393939398 |
| 0.1 | 0.08645096056622854 | 0.06916426512968303 | 0.07375 | 0.0882114917723226 | 0.0763701707097934 | 0.05531250000000002 | 0.0803738317757009 | 0.07540263543191802 | 0.07339955849889622 | 0.0757575757575758 |
| 0.12000000000000002 | 0.0662285136501517 | 0.050432276657060515 | 0.05875 | 0.06878877798759103 | 0.05959868224019174 | 0.0425 | 0.04423676012461063 | 0.05636896046852121 | 0.057671081677704183 | 0.0638888888888889 |
| 0.14000000000000001 | 0.053083923154701736 | 0.0499519692603266 | 0.06375 | 0.060695980577286215 | 0.047319556753519 | 0.0328125 | 0.040809968847352 | 0.038067349926793614 | 0.0469094922737307 | 0.05025252525252532 |
| 0.16 | 0.053083923154701736 | 0.042747358309318 | 0.05 | 0.042352306447261914 | 0.0359389038634322 | 0.0259375 | 0.030841121495327115 | 0.0336749633967789 | 0.0389072847682119 | 0.0454545454545455 |
| 0.18000000000000005 | 0.04145601617795749 | 0.04322766570605188 | 0.03375 | 0.04720798489344482 | 0.037436358191075214 | 0.026250000000000002 | 0.0348909657320872 | 0.0395314787701318 | 0.031456953642384114 | 0.0366161616161616 |
| 0.2 | 0.03185035389282101 | 0.036503362151777116 | 0.03875 | 0.038575667655786405 | 0.04162923030847559 | 0.0246875 | 0.037383177570093525 | 0.0285505124450952 | 0.034768211920529805 | 0.034595959595959604 |
| 0.22 | 0.03185035389282101 | 0.03746397694524502 | 0.027500000000000007 | 0.039384947396816805 | 0.03204552261156041 | 0.028749999999999998 | 0.028037383177570117 | 0.03404099560761351 | 0.029801324503311313 | 0.0333333333333333 |
| 0.24000000000000005 | 0.02780586450960572 | 0.03746397694524502 | 0.0325 | 0.037226868087402214 | 0.03204552261156041 | 0.0315625 | 0.03707165109034271 | 0.027086383601757007 | 0.0259381898454746 | 0.023989898989899 |
| 0.26 | 0.025278058645096108 | 0.03554274735830931 | 0.03625000000000001 | 0.0267062314540059 | 0.0260557053009883 | 0.0353125 | 0.028660436137071685 | 0.0248901903367496 | 0.02455849889624719 | 0.01994949494949501 |
| 0.28000000000000008 | 0.023761375126390306 | 0.03314121037463981 | 0.023749999999999997 | 0.025627191799298597 | 0.022761305780173723 | 0.027187500000000007 | 0.028660436137071685 | 0.0285505124450952 | 0.0201434878587196 | 0.01868686868686871 |
| 0.3000000000000001 | 0.018705763397371105 | 0.03554274735830931 | 0.026250000000000002 | 0.01996223361208521 | 0.0212638514525307 | 0.021562499999999988 | 0.02429906542056071 | 0.02379209370424599 | 0.0182119205298013 | 0.013383838383838408 |
| 0.32000000000000012 | 0.0101112234580384 | 0.03218059558117201 | 0.01625 | 0.020771513353115705 | 0.011081162024558304 | 0.020625 | 0.02959501557632402 | 0.017935578330893105 | 0.013245033112582804 | 0.013636363636363601 |
| 0.34 | 0.009605662285136504 | 0.026897214217098907 | 0.011250000000000001 | 0.012139196115457199 | 0.0152740341419587 | 0.0178125 | 0.02585669781931461 | 0.015007320644216707 | 0.010485651214128007 | 0.00808080808080808 |
| 0.3600000000000001 | 0.0101112234580384 | 0.0172910662824208 | 0.017500000000000005 | 0.0097113568923658 | 0.0107816711590297 | 0.020000000000000007 | 0.01900311526479751 | 0.011346998535871199 | 0.00883002207505519 | 0.011111111111111101 |
| 0.38000000000000012 | 0.003538928210313451 | 0.013448607108549499 | 0.0125 | 0.008632317237658485 | 0.005989817310572032 | 0.0125 | 0.009968847352024924 | 0.0069546120058565225 | 0.004690949227373072 | 0.005050505050505048 |
| 0.4 | 0.003538928210313451 | 0.008165225744476456 | 0.005000000000000002 | 0.0040463987051524136 | 0.003893381251871822 | 0.0071875 | 0.009968847352024924 | 0.005124450951683748 | 0.003035320088300221 | 0.00303030303030303 |
### Chart: unc-25 A3
| Category | unc-25_A3_40_30_01 | unc-25_A3_40_30_03 | unc-25_A3_40_30_2 | unc-25_A3_40_30_4 | unc-25_REX_A3_01 | unc-25_REX_A3_03 | unc-25_REX_A3_04 | unc-25_REX_A3_05 | unc-25_REX_A3_06 | unc-25_REX_A3_08 |
|---|---|---|---|---|---|---|---|---|---|---|
| 0 | None | None | None | None | None | None | None | None | None | None |
| 2.0000000000000011E-2 | 0.0877712031558185 | 0.07654320987654321 | 0.0929423459244534 | 0.0694891854578923 | 0.09670143783479 | 0.08583066627873152 | 0.10416666666666707 | 0.10218767990788702 | 0.112340131351538 | 0.09017805858701908 |
| 4.0000000000000022E-2 | 0.09418145956607497 | 0.10222222222222214 | 0.11232604373757506 | 0.08973768982972849 | 0.115590639977446 | 0.101251091067792 | 0.11666666666666706 | 0.11370178468624108 | 0.11406844106463908 | 0.11229178632969605 |
| 6.0000000000000032E-2 | 0.1277120315581851 | 0.0997530864197531 | 0.10337972166998002 | 0.11596870685688006 | 0.10487736115026793 | 0.11114343904567905 | 0.119345238095238 | 0.11283822682786394 | 0.11406844106463908 | 0.109419873635841 |
| 8.0000000000000043E-2 | 0.09960552268244592 | 0.0967901234567901 | 0.09840954274353884 | 0.10952600092038711 | 0.09585565266422343 | 0.100669188245563 | 0.10327380952381007 | 0.0926885434657455 | 0.0895264431386106 | 0.10022975301550806 |
| 0.1 | 0.0902366863905325 | 0.08296296296296307 | 0.0666003976143141 | 0.09249884951679717 | 0.07809416408232311 | 0.082048297934245 | 0.093154761904762 | 0.0768566493955095 | 0.0712063601797442 | 0.0784032165422171 |
| 0.12000000000000002 | 0.0660749506903353 | 0.0740740740740741 | 0.0571570576540755 | 0.07501150483202955 | 0.0614603890611785 | 0.07622926971195805 | 0.058928571428571414 | 0.063615428900403 | 0.043207742827514696 | 0.0574382538770821 |
| 0.14000000000000001 | 0.0744575936883629 | 0.06222222222222224 | 0.0457256461232604 | 0.05292222733548094 | 0.041725401747956 | 0.055280768111725304 | 0.0419642857142857 | 0.053252734599884895 | 0.04009678534393368 | 0.050832854681217704 |
| 0.16 | 0.044871794871794914 | 0.05629629629629634 | 0.04721669980119286 | 0.05062126092959041 | 0.0377784042853115 | 0.05033459412278155 | 0.0449404761904762 | 0.0463442717328728 | 0.03664016591773253 | 0.049684089603675985 |
| 0.1800000000000001 | 0.042406311637080904 | 0.048395061728395104 | 0.03727634194831013 | 0.04463874827427516 | 0.0462362559909783 | 0.0418970032004655 | 0.0357142857142857 | 0.03684513529073113 | 0.03664016591773253 | 0.0450890292935095 |
| 0.2 | 0.03747534516765295 | 0.04592592592592591 | 0.0308151093439364 | 0.042797975149562836 | 0.0335494784324782 | 0.03665987780040735 | 0.0333333333333333 | 0.037996545768566536 | 0.0331835464915313 | 0.038483630097645 |
| 0.22 | 0.030078895463510835 | 0.034567901234567884 | 0.029324055666004 | 0.0317533364012885 | 0.03101212292077812 | 0.026476578411405337 | 0.030952380952381 | 0.0325273459988486 | 0.027307293466989336 | 0.0338885697874785 |
| 0.2400000000000001 | 0.025147928994082788 | 0.0306172839506173 | 0.0228628230616302 | 0.02807179015186382 | 0.027065125458133624 | 0.027931335466977052 | 0.02261904761904763 | 0.02389176741508354 | 0.026615969581749034 | 0.028431935669155743 |
| 0.26 | 0.020710059171597583 | 0.016790123456790103 | 0.020377733598409525 | 0.022089277496548626 | 0.0281928390188892 | 0.022985161478033235 | 0.01875 | 0.02360391479562462 | 0.017974421016246113 | 0.021539345203905828 |
| 0.28000000000000008 | 0.01923076923076922 | 0.014320987654320999 | 0.0193836978131213 | 0.02300966405890471 | 0.019453058923033603 | 0.0183299389002037 | 0.012797619047619007 | 0.012665515256188812 | 0.011752506049084012 | 0.018954623779437112 |
| 0.30000000000000021 | 0.0138067061143984 | 0.017777777777777802 | 0.012922465208747515 | 0.012425218591808599 | 0.015787989850578003 | 0.013674716322374198 | 0.0101190476190476 | 0.0109383995394358 | 0.009332872450743173 | 0.015221137277426801 |
| 0.32000000000000023 | 0.009368836291913228 | 0.012839506172839498 | 0.009940357852882708 | 0.018407731247123818 | 0.01381449111925571 | 0.0116380564445738 | 0.007440476190476197 | 0.0066206102475532495 | 0.008987210508123072 | 0.011487650775416403 |
| 0.34 | 0.007396449704142019 | 0.00592592592592593 | 0.005964214711729624 | 0.009664058904739998 | 0.00789399492528898 | 0.01018329938900201 | 0.00654761904761905 | 0.005757052389176742 | 0.003802281368821294 | 0.0103388856978748 |
| 0.36000000000000021 | 0.004930966469428011 | 0.006419753086419757 | 0.006958250497017894 | 0.005982512655315232 | 0.005920496193966736 | 0.009019493744544669 | 0.006250000000000004 | 0.004605641911341394 | 0.003110957483581061 | 0.007466973004020689 |
| 0.38000000000000023 | 0.002958579881656801 | 0.002469135802469142 | 0.00149105367793241 | 0.007363092498849529 | 0.004792782633211164 | 0.005819028222286884 | 0.00386904761904762 | 0.00316637881404721 | 0.001382647770480471 | 0.0034462952326249335 |
| 0.4 | 0.003451676528599612 | 0.002962962962962962 | 0.005467196819085485 | 0.003221352968246662 | 0.0036650690724556022 | 0.002909514111143442 | 0.0014880952380952408 | 0.0014392630972941882 | 0.000345661942620118 | 0.002010338885697872 |
### Chart: unc-25 A5
| Category | unc-25_A5_001 | unc-25_A5_002 | unc-25_A5_003 | unc-25_A5_004 | unc-25_A5_006 | unc-25_A5_40_30_01 | unc-25_A5_40_30_03 | unc-25_A5_40_30_04 | unc-25_A5_M102 | unc-25_A5_M103 | unc-25_A5_M105 | unc-25_A5_M108 | unc-25_A5_M109 | unc-25_A5_M110 | unc-25_A5_M111 | unc-25_REX_A5_01 | unc-25_REX_A5_02 |
|---|---|---|---|---|---|---|---|---|---|---|---|---|---|---|---|---|---|
| 0 | None | None | None | None | None | None | None | None | None | None | None | None | None | None | None | None | None |
| 2.0000000000000011E-2 | 0.09364548494983292 | 0.08942558746736293 | 0.0958829902491874 | 0.09163346613545831 | 0.10871302957633912 | 0.10303587856485702 | 0.111111111111111 | 0.106111484217596 | 0.09599528857479402 | 0.10721982758620706 | 0.1045056320400501 | 0.09496810772501772 | 0.10475161987041005 | 0.095572733661279 | 0.08900523560209422 | 0.120383036935705 | 0.11056910569105698 |
| 4.0000000000000022E-2 | 0.11304347826087002 | 0.0998694516971279 | 0.10130010834236194 | 0.111553784860558 | 0.11350919264588298 | 0.119595216191352 | 0.11187739463601494 | 0.11349899261249193 | 0.11896348645465306 | 0.125 | 0.11639549436796 | 0.10347271438696005 | 0.1360691144708421 | 0.09908643710470838 | 0.1017202692595361 | 0.111263109895121 | 0.11910569105691111 |
| 6.0000000000000032E-2 | 0.09899665551839479 | 0.09595300261096618 | 0.0942578548212351 | 0.105577689243028 | 0.11031175059951999 | 0.11591536338546495 | 0.09118773946360148 | 0.12357286769644098 | 0.09481743227326281 | 0.112068965517241 | 0.10763454317897411 | 0.1282778171509569 | 0.140388768898488 | 0.09346451159522148 | 0.10321615557217707 | 0.0889192886456908 | 0.1252032520325201 |
| 8.0000000000000043E-2 | 0.071571906354515 | 0.08485639686684082 | 0.0958829902491874 | 0.06474103585657377 | 0.07993605115907275 | 0.06715731370745172 | 0.08659003831417628 | 0.10073875083949002 | 0.0742049469964664 | 0.09806034482758627 | 0.09011264080100119 | 0.10347271438696005 | 0.09827213822894168 | 0.07940969782150399 | 0.0905011219147345 | 0.07159142726858192 | 0.107317073170732 |
| 0.1 | 0.0729096989966555 | 0.0789817232375979 | 0.0937161430119177 | 0.06872509960159362 | 0.06314948041566748 | 0.08003679852805898 | 0.07586206896551725 | 0.073875083948959 | 0.06949352179034171 | 0.061422413793103404 | 0.08197747183979978 | 0.06945428773919211 | 0.07775377969762426 | 0.08713984539704861 | 0.07404637247569182 | 0.06064751481988143 | 0.07073170731707326 |
| 0.12000000000000002 | 0.0682274247491639 | 0.059399477806788586 | 0.07800650054171186 | 0.052788844621513904 | 0.0511590727418066 | 0.053357865685372555 | 0.0651340996168582 | 0.058428475486904 | 0.07126030624263842 | 0.061422413793103404 | 0.052565707133917436 | 0.058114812189936214 | 0.05075593952483804 | 0.060435699226985246 | 0.06357516828721015 | 0.04468764249885997 | 0.04796747967479674 |
| 0.14000000000000001 | 0.0561872909698997 | 0.0489556135770235 | 0.07096424702058511 | 0.0468127490039841 | 0.04636290967226218 | 0.031278748850046015 | 0.05363984674329494 | 0.0510409670920081 | 0.0559481743227326 | 0.0490301724137931 | 0.045056320400500637 | 0.045357902197023424 | 0.04211663066954648 | 0.05621925509486995 | 0.0553477935676889 | 0.03739170086639315 | 0.043495934959349704 |
| 0.16 | 0.051505016722407974 | 0.0443864229765014 | 0.06067172264355356 | 0.04183266932270924 | 0.0399680255795364 | 0.0331186752529899 | 0.052107279693486636 | 0.0402955003357958 | 0.040047114252061235 | 0.040948275862068964 | 0.0431789737171464 | 0.03685329553508153 | 0.058315334773218104 | 0.03513703443429373 | 0.0441286462228871 | 0.02963976288189693 | 0.033739837398374016 |
| 0.1800000000000001 | 0.0334448160535117 | 0.04699738903394264 | 0.0487540628385699 | 0.0338645418326693 | 0.03437250199840128 | 0.029438822447102116 | 0.0367816091954023 | 0.0322364002686367 | 0.04122497055359248 | 0.0398706896551724 | 0.03692115143929911 | 0.034727143869596 | 0.0302375809935205 | 0.0491918482080112 | 0.04712041884816751 | 0.029183766529867802 | 0.02642276422764228 |
| 0.2 | 0.040133779264214 | 0.0365535248041775 | 0.03900325027085593 | 0.0388446215139442 | 0.038369304556354906 | 0.024839006439742402 | 0.0367816091954023 | 0.030221625251846893 | 0.04652532391048288 | 0.038254310344827604 | 0.0387984981226533 | 0.04961020552799431 | 0.024838012958963342 | 0.040056219255094914 | 0.03814510097232613 | 0.03055175558595533 | 0.015447154471544699 |
| 0.22 | 0.03210702341137123 | 0.035900783289817204 | 0.03954496208017343 | 0.02788844621513941 | 0.0303756994404476 | 0.0239190432382705 | 0.0383141762452107 | 0.023505708529214228 | 0.0382803297997644 | 0.02909482758620693 | 0.029411764705882398 | 0.034727143869596 | 0.0172786177105832 | 0.031623330990864416 | 0.024682124158564002 | 0.028271773825809438 | 0.02073170731707324 |
| 0.2400000000000001 | 0.0254180602006689 | 0.0306788511749347 | 0.0184182015167931 | 0.03087649402390441 | 0.02078337330135891 | 0.016559337626494898 | 0.020689655172413828 | 0.016118200134318302 | 0.0306242638398115 | 0.021012931034482797 | 0.025657071339174 | 0.033309709425939 | 0.016198704103671697 | 0.030217849613492602 | 0.018698578908003013 | 0.025535795713634325 | 0.0130081300813008 |
| 0.26 | 0.02073578595317731 | 0.029373368146214135 | 0.022751895991332597 | 0.013944223107569705 | 0.02318145483613106 | 0.0147194112235511 | 0.0168582375478927 | 0.010745466756212208 | 0.028268551236749064 | 0.0204741379310345 | 0.015018773466833503 | 0.016300496102055303 | 0.007559395248380136 | 0.029515108924806712 | 0.00972326103216156 | 0.021431828545371626 | 0.009349593495934968 |
| 0.28000000000000008 | 0.0193979933110368 | 0.0156657963446475 | 0.013542795232936116 | 0.02689243027888451 | 0.023980815347721802 | 0.011039558417663315 | 0.013793103448275909 | 0.007387508394895903 | 0.020612485276796197 | 0.0129310344827586 | 0.016896120150187714 | 0.009213323883770374 | 0.005399568034557244 | 0.024595924104005597 | 0.00822737471952132 | 0.00911992704058368 | 0.00650406504065041 |
| 0.30000000000000021 | 0.016722408026755915 | 0.011096605744125309 | 0.007042253521126766 | 0.023904382470119535 | 0.0159872102318145 | 0.005519779208831656 | 0.009961685823754802 | 0.00537273337810611 | 0.0159010600706714 | 0.0113146551724138 | 0.008135168961201499 | 0.010630758327427403 | 0.00107991360691145 | 0.014757554462403401 | 0.007479431563201207 | 0.0063839489284085735 | 0.006910569105691071 |
| 0.32000000000000023 | 0.0107023411371237 | 0.013707571801566617 | 0.00866738894907909 | 0.012948207171314698 | 0.014388489208633115 | 0.007359705611775532 | 0.0038314176245210717 | 0.0026863666890530598 | 0.011189634864546499 | 0.008620689655172419 | 0.00563204005006258 | 0.010630758327427403 | 0.00107991360691145 | 0.014757554462403401 | 0.005235602094240851 | 0.00729594163246694 | 0.0028455284552845518 |
| 0.34 | 0.011371237458193998 | 0.011749347258485601 | 0.01083423618634889 | 0.009960159362549808 | 0.007194244604316552 | 0.005519779208831656 | 0.00459770114942529 | 0.0026863666890530598 | 0.0070671378091872765 | 0.0037715517241379347 | 0.0050062578222778535 | 0.0035435861091424547 | 0.0 | 0.007730147575544632 | 0.00822737471952132 | 0.0063839489284085735 | 0.00203252032520325 |
| 0.36000000000000021 | 0.00735785953177258 | 0.0045691906005221935 | 0.0005417118093174436 | 0.012948207171314698 | 0.0031974420463629135 | 0.00275988960441582 | 0.0038314176245210717 | 0.002014775016789788 | 0.00353356890459364 | 0.0037715517241379347 | 0.0 | 0.0007087172218284901 | 0.00107991360691145 | 0.007730147575544632 | 0.005235602094240851 | 0.0018239854081167418 | 0.0040650406504065 |
| 0.38000000000000023 | 0.00535117056856187 | 0.0032637075718015755 | 0.002708559046587223 | 0.003984063745019924 | 0.004796163069544369 | 0.0009199632014719427 | 0.001532567049808431 | 0.0006715916722632646 | 0.00235571260306243 | 0.002155172413793098 | 0.0018773466833541899 | 0.0014174344436569798 | 0.0 | 0.0035137034434293735 | 0.00149588631264024 | 0.002279981760145924 | 0.0016260162601626012 |
| 0.4 | 0.002675585284280943 | 0.007832898172323768 | 0.001625135427952331 | 0.002988047808764942 | 0.0023980815347721812 | 0.00183992640294388 | 0.000766283524904215 | 0.0006715916722632646 | 0.00235571260306243 | 0.0016163793103448308 | 0.003128911138923654 | 0.002126151665485472 | 0.0 | 0.0035137034434293735 | 0.00149588631264024 | 0.0018239854081167418 | 0.000813008130081301 |

## Slide 2
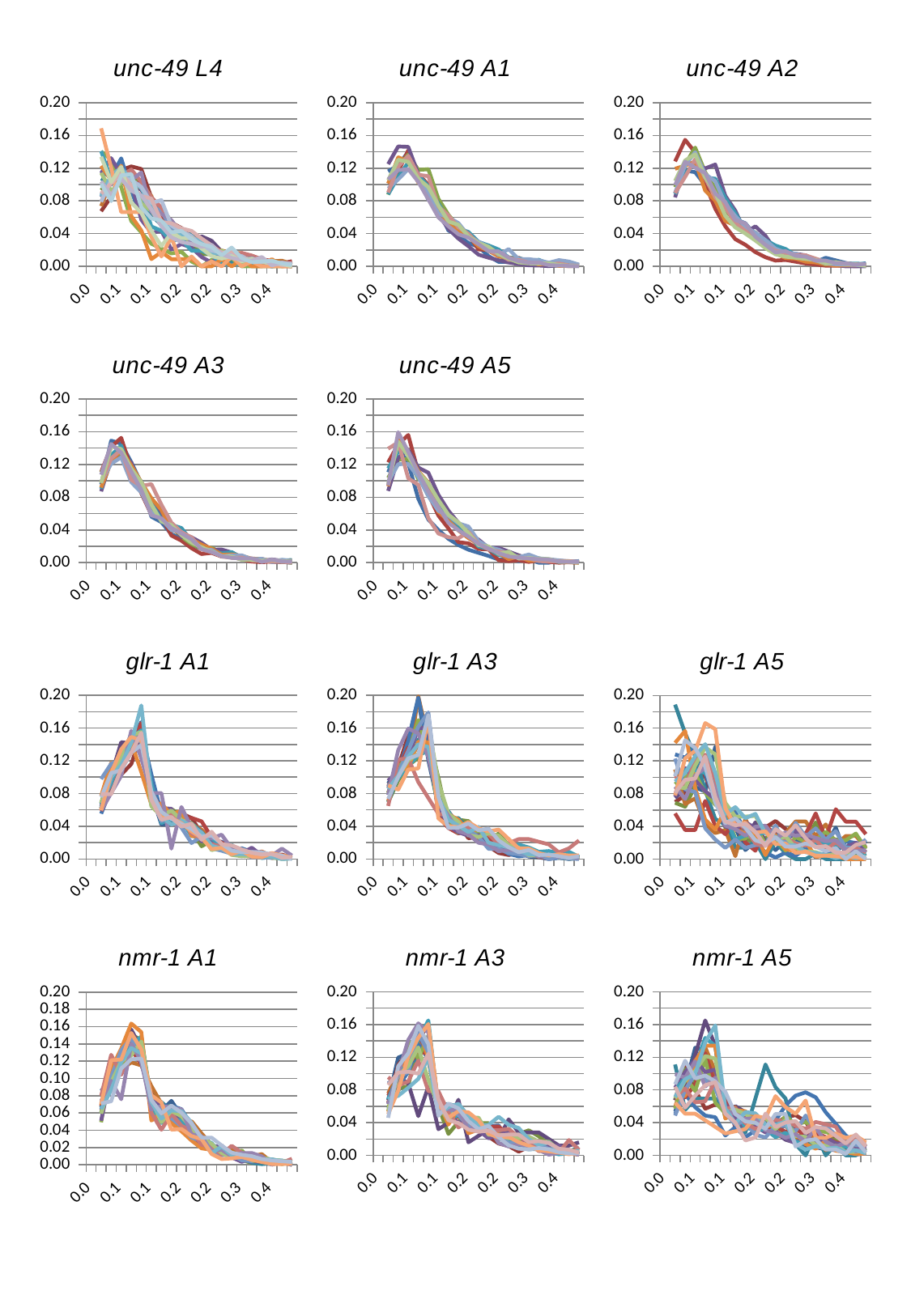

### Chart: unc-49 L4
| Category | unc-49_L4_0601 | unc-49_L4_0602 | unc-49_L4_06021 | unc-49_L4_06022 | unc-49_L4_06023 | unc-49_L4_06024 | unc-49_L4_06025 | unc-49_L4_06026 | unc-49_L4_0603 | unc-49_L4_0604 | unc-49_L4_0605 | unc-49_L4_0606 | unc-49_L4_0607 | unc-49_L4_0608 | unc-49_L4_0609 | unc-49_L4_0611 | unc-49_L4_0612 | unc-49_L4_0613 | unc-49_L4_0614 | unc-49_L4_0615 | unc-49_L4_0616 | unc-49_L4_0617 | unc-49_L4_0618 | unc-49_L4_0619 |
|---|---|---|---|---|---|---|---|---|---|---|---|---|---|---|---|---|---|---|---|---|---|---|---|---|
| 0 | None | None | None | None | None | None | None | None | None | None | None | None | None | None | None | None | None | None | None | None | None | None | None | None |
| 2.0000000000000007E-2 | None | 0.0673493501378496 | 0.09299655568312283 | 0.09969969969969976 | 0.107344632768362 | 0.0735202492211838 | 0.11602209944751403 | 0.08459770114942533 | 0.14031620553359705 | 0.11389521640091105 | 0.14086956521739105 | 0.12280701754385999 | 0.08731241473396996 | 0.0789041095890411 | 0.114213197969543 | 0.08442330558858503 | 0.08541973490427096 | 0.16867469879518093 | 0.0788381742738589 | 0.08894044856921897 | 0.13395638629283507 | 0.10376282782212103 | 0.10093299406276497 | None |
| 4.0000000000000015E-2 | None | 0.08507286333202053 | 0.10619977037887506 | 0.10030030030029999 | 0.10056497175141209 | 0.10467289719626198 | 0.10865561694291004 | 0.10160919540229903 | 0.130434782608696 | 0.132118451025057 | 0.10608695652173904 | 0.105263157894737 | 0.102319236016371 | 0.10356164383561606 | 0.0922165820642978 | 0.10344827586206902 | 0.111438389788905 | 0.120481927710843 | 0.10529045643153509 | 0.10131477184841503 | 0.102803738317757 | 0.08665906499429873 | 0.07972858354537743 | None |
| 6.0000000000000019E-2 | None | 0.117762898779047 | 0.11768082663605102 | 0.11171171171171203 | 0.10508474576271205 | 0.10404984423676 | 0.13198281154082306 | 0.11862068965517203 | 0.09683794466403163 | 0.11161731207289295 | 0.111304347826087 | 0.12280701754385999 | 0.11869031377899003 | 0.10958904109589 | 0.10236886632825698 | 0.11593341260404297 | 0.120274914089347 | 0.0662650602409639 | 0.108921161825726 | 0.11291569992266 | 0.121495327102804 | 0.10718358038768504 | 0.111111111111111 | None |
| 8.0000000000000029E-2 | None | 0.12209531311540002 | 0.10964408725602805 | 0.09489489489489498 | 0.102824858757062 | 0.10155763239875397 | 0.09085328422345 | 0.100229885057471 | 0.05533596837944662 | 0.10250569476082003 | 0.09565217391304354 | 0.06140350877192978 | 0.09481582537517057 | 0.118356164383562 | 0.109137055837563 | 0.10523186682520802 | 0.10554737358861103 | 0.0662650602409639 | 0.10580912863070498 | 0.09899458623356544 | 0.07788161993769473 | 0.09236031927023951 | 0.11280746395250198 | None |
| 0.1 | None | 0.11894446632532497 | 0.10390355912744 | 0.09429429429429434 | 0.0971751412429379 | 0.09595015576323995 | 0.06384284837323508 | 0.102068965517241 | 0.04150197628458497 | 0.0569476082004556 | 0.0852173913043478 | 0.043859649122807 | 0.10163710777626203 | 0.103013698630137 | 0.07698815566835868 | 0.11474435196195006 | 0.09523809523809525 | 0.0662650602409639 | 0.09699170124481328 | 0.08584686774942006 | 0.0654205607476635 | 0.08665906499429873 | 0.07209499575911793 | None |
| 0.12000000000000002 | None | 0.08389129578574248 | 0.0838117106773823 | 0.0612612612612613 | 0.0621468926553672 | 0.08598130841121501 | 0.061387354205033814 | 0.08000000000000003 | 0.027667984189723313 | 0.041002277904328026 | 0.048695652173913 | 0.0087719298245614 | 0.07571623465211466 | 0.08438356164383558 | 0.0676818950930626 | 0.0665873959571938 | 0.07511045655375552 | 0.036144578313253 | 0.07728215767634863 | 0.08275328692962099 | 0.043613707165109 | 0.0695553021664766 | 0.0585241730279898 | None |
| 0.14000000000000001 | None | 0.059472233162662515 | 0.07060849598163033 | 0.0588588588588589 | 0.05084745762711862 | 0.053582554517134014 | 0.057704112952731736 | 0.06160919540229888 | 0.0217391304347826 | 0.0432801822323462 | 0.0434782608695652 | 0.017543859649122813 | 0.06616643929058663 | 0.07178082191780823 | 0.05076142131979703 | 0.06420927467300831 | 0.0510554737358861 | 0.012048192771084298 | 0.08091286307053944 | 0.05413766434648114 | 0.0249221183800623 | 0.0490307867730901 | 0.055979643765903295 | None |
| 0.16 | None | 0.053170539582512784 | 0.05510907003444323 | 0.0510510510510511 | 0.03276836158192092 | 0.0479750778816199 | 0.047268262737876 | 0.04597701149425289 | 0.0158102766798419 | 0.02050113895216401 | 0.031304347826087014 | 0.0087719298245614 | 0.048431105047749 | 0.0542465753424658 | 0.03637901861252121 | 0.0564803804994055 | 0.0510554737358861 | 0.036144578313253 | 0.049792531120331954 | 0.052590873936581635 | 0.040498442367601216 | 0.0353477765108324 | 0.0415606446140797 | None |
| 0.18000000000000005 | None | 0.046868846002363095 | 0.04190585533869118 | 0.039039039039039 | 0.0361581920903955 | 0.0392523364485981 | 0.030079803560466516 | 0.0427586206896552 | 0.017786561264822108 | 0.0273348519362187 | 0.03304347826086961 | 0.0087719298245614 | 0.0361527967257844 | 0.03671232876712331 | 0.040609137055837616 | 0.03983353151010701 | 0.038782523318605794 | 0.0 | 0.0430497925311203 | 0.0464037122969838 | 0.034267912772585715 | 0.029646522234891694 | 0.043256997455470715 | None |
| 0.2 | None | 0.035447026388341914 | 0.0292766934557979 | 0.028828828828828798 | 0.039548022598870115 | 0.03613707165109031 | 0.0282381829343155 | 0.0271264367816092 | 0.005928853754940712 | 0.022779043280182206 | 0.019130434782608705 | 0.0087719298245614 | 0.034106412005457 | 0.035616438356164404 | 0.027918781725888294 | 0.0309155766944114 | 0.0353460972017673 | 0.012048192771084298 | 0.0368257261410788 | 0.0433101314771848 | 0.034267912772585715 | 0.0273660205245154 | 0.033078880407124714 | None |
| 0.22 | None | 0.0319023237495077 | 0.014925373134328401 | 0.0366366366366366 | 0.02824858757062151 | 0.02990654205607481 | 0.02332719459791279 | 0.02896551724137931 | 0.0 | 0.011389521640091107 | 0.019130434782608705 | 0.0 | 0.030695770804911315 | 0.030684931506849318 | 0.0228426395939086 | 0.0309155766944114 | 0.02994599901816401 | 0.0 | 0.0238589211618257 | 0.0340293890177881 | 0.021806853582554513 | 0.023945267958951 | 0.02714164546225612 | None |
| 0.24000000000000005 | None | 0.0232374950768019 | 0.014925373134328401 | 0.031231231231231206 | 0.016949152542372906 | 0.021806853582554513 | 0.020871700429711523 | 0.02528735632183911 | 0.00790513833992095 | 0.004555808656036448 | 0.0139130434782609 | 0.0 | 0.02387448840381991 | 0.0235616438356164 | 0.014382402707275801 | 0.0178359096313912 | 0.01620029455081 | 0.006024096385542173 | 0.019190871369294607 | 0.0255220417633411 | 0.012461059190031204 | 0.026225769669327312 | 0.023748939779474114 | None |
| 0.26 | None | 0.014572666404096095 | 0.013777267508610799 | 0.018018018018018007 | 0.007909604519774013 | 0.019314641744548305 | 0.006752608962553712 | 0.0174712643678161 | 0.00395256916996047 | 0.01594533029612761 | 0.00347826086956522 | 0.0087719298245614 | 0.011596180081855404 | 0.01424657534246581 | 0.019458544839255507 | 0.015457788347205707 | 0.015709376534118805 | 0.0 | 0.0171161825726141 | 0.0162412993039443 | 0.009345794392523367 | 0.0148232611174458 | 0.013570822731128104 | None |
| 0.28000000000000008 | None | 0.014178810555336699 | 0.009758897818599318 | 0.0132132132132132 | 0.00451977401129944 | 0.0161993769470405 | 0.0128913443830571 | 0.012873563218390801 | 0.009881422924901195 | 0.013667425968109305 | 0.0017391304347826105 | 0.0 | 0.010914051841746203 | 0.0104109589041096 | 0.0126903553299492 | 0.0101070154577883 | 0.01620029455081 | 0.012048192771084298 | 0.0134854771784232 | 0.018561484918793506 | 0.012461059190031204 | 0.0102622576966933 | 0.0229007633587786 | None |
| 0.3000000000000001 | None | 0.011421819614021307 | 0.006314580941446608 | 0.008408408408408414 | 0.00903954802259887 | 0.0161993769470405 | 0.0036832412523020324 | 0.0119540229885057 | 0.0 | 0.002277904328018221 | 0.00347826086956522 | 0.0087719298245614 | 0.004774897680763982 | 0.0164383561643836 | 0.00423011844331641 | 0.0071343638525564815 | 0.010800196367206704 | 0.0 | 0.012448132780083 | 0.0100541376643465 | 0.006230529595015582 | 0.007981755986316993 | 0.010178117048346098 | None |
| 0.32000000000000012 | None | 0.010634107916502603 | 0.008036739380022964 | 0.0036036036036036 | 0.006779661016949154 | 0.009968847352024924 | 0.0049109883364027 | 0.012873563218390801 | 0.0 | 0.004555808656036448 | 0.0017391304347826105 | 0.0 | 0.006139154160982262 | 0.013150684931506899 | 0.00676818950930626 | 0.0101070154577883 | 0.008345606283750617 | 0.006024096385542173 | 0.006224066390041492 | 0.007733952049497291 | 0.009345794392523367 | 0.006841505131128852 | 0.005089058524173034 | None |
| 0.34 | None | 0.008270972823946444 | 0.008610792192881744 | 0.006606606606606611 | 0.00112994350282486 | 0.006230529595015582 | 0.004297114794352362 | 0.008735632183908054 | 0.0 | 0.004555808656036448 | 0.0017391304347826105 | 0.0 | 0.006139154160982262 | 0.007671232876712332 | 0.005076142131979704 | 0.005945303210463734 | 0.008345606283750617 | 0.0 | 0.005186721991701242 | 0.008507347254447024 | 0.006230529595015582 | 0.011402508551881404 | 0.005937234944868532 | None |
| 0.3600000000000001 | None | 0.005907837731390312 | 0.00459242250287026 | 0.0030030030030030012 | 0.00225988700564972 | 0.006230529595015582 | 0.003069367710251692 | 0.0064367816091954015 | 0.0 | 0.0 | 0.0 | 0.0087719298245614 | 0.0027285129604365617 | 0.003287671232876712 | 0.00676818950930626 | 0.005945303210463734 | 0.001963672066764851 | 0.0 | 0.004149377593361002 | 0.00464037122969838 | 0.0031152647975077907 | 0.0011402508551881406 | 0.006785411365564043 | None |
| 0.38000000000000012 | None | 0.0035447026388341907 | 0.003444316877152702 | 0.005405405405405412 | 0.00112994350282486 | 0.006853582554517131 | 0.0049109883364027 | 0.0018390804597701105 | 0.0 | 0.002277904328018221 | 0.0017391304347826105 | 0.0 | 0.0027285129604365617 | 0.004383561643835622 | 0.003384094754653132 | 0.002378121284185491 | 0.00147275405007364 | 0.0 | 0.004149377593361002 | 0.00154679040989946 | 0.0031152647975077907 | 0.0045610034207525735 | 0.003392705682782021 | None |
| 0.4 | None | 0.006301693580149675 | 0.0022962112514351312 | 0.0036036036036036 | 0.0 | 0.0031152647975077907 | 0.0012277470841006801 | 0.0059770114942528756 | 0.001976284584980242 | 0.0 | 0.0 | 0.0 | 0.0027285129604365617 | 0.0016438356164383604 | 0.003384094754653132 | 0.00297265160523187 | 0.00294550810014728 | 0.0 | 0.0020746887966805 | 0.0007733952049497292 | 0.0 | 0.003420752565564421 | 0.0016963528413910117 | None |
### Chart: unc-49 A1
| Category | unc-49_REX_A1_01 | unc-49_REX_A1_02 | unc-49_REX_A1_03 | unc-49_REX_A1_04 | unc-49_REX_A1_05 | unc-49_REX_A1_06 | unc-49_REX_A1_07 | unc-49_REX_A1_08 | unc-49_REX_A1_09 | unc-49_REX_A1_10 |
|---|---|---|---|---|---|---|---|---|---|---|
| 0 | None | None | None | None | None | None | None | None | None | None |
| 2.0000000000000007E-2 | 0.11600221483942398 | 0.10119343610144202 | 0.0874747020463234 | 0.12490594431903702 | 0.08803088803088806 | 0.10117789187556597 | 0.09504253446758576 | 0.09012345679012353 | 0.10527589545014503 | 0.10626773278308005 |
| 4.0000000000000015E-2 | 0.12347729789590298 | 0.11611138736946795 | 0.12187991904654798 | 0.14647604715324805 | 0.10939510939510905 | 0.13319238900634306 | 0.106189498386624 | 0.12197530864197502 | 0.130203291384318 | 0.11761671395408803 |
| 6.0000000000000019E-2 | 0.139811738648948 | 0.141720537046246 | 0.13649651450416006 | 0.145974416854778 | 0.12689832689832706 | 0.128360012080942 | 0.11880316808448202 | 0.135061728395062 | 0.12778315585672806 | 0.11890637090533904 |
| 8.0000000000000029E-2 | 0.10769656699889303 | 0.11263053207359502 | 0.11783224645828605 | 0.11261600200652103 | 0.111454311454311 | 0.102385986106916 | 0.10853622763273703 | 0.11185185185185198 | 0.10696999031945797 | 0.103172556100077 |
| 0.1 | 0.081672203765227 | 0.09970164097463957 | 0.11873172925567806 | 0.08377225984449463 | 0.09781209781209778 | 0.0930232558139535 | 0.0906424171311235 | 0.110123456790123 | 0.09680542110358183 | 0.0817642507093113 |
| 0.12000000000000002 | 0.0686600221483942 | 0.07881650919940333 | 0.08252754666067015 | 0.0632054176072235 | 0.0764478764478764 | 0.06916339474479012 | 0.06424171311234972 | 0.07308641975308643 | 0.07454017424975802 | 0.0598400825380449 |
| 0.14000000000000001 | 0.04374307862679959 | 0.05395325708602691 | 0.062963795817405 | 0.04589917231000749 | 0.056885456885456896 | 0.052552099063727015 | 0.059841595775887386 | 0.06222222222222222 | 0.054695062923523725 | 0.049006964147536844 |
| 0.16 | 0.037375415282392015 | 0.04873197414221782 | 0.04834720035979311 | 0.033860045146726914 | 0.04633204633204634 | 0.04228329809725161 | 0.05280140803754772 | 0.0476543209876543 | 0.050822846079380396 | 0.03920557131802942 |
| 0.18000000000000005 | 0.028516057585825007 | 0.038538040775733515 | 0.03867776028783451 | 0.024579884625031398 | 0.04195624195624202 | 0.0362428269405014 | 0.03754766793781172 | 0.039753086419753114 | 0.036544046466602115 | 0.03507866907402631 |
| 0.2 | 0.023809523809523808 | 0.020636499254102398 | 0.0245109062289184 | 0.014296463506395796 | 0.029858429858429892 | 0.0244639081848384 | 0.02992079788794371 | 0.027407407407407415 | 0.028557599225556597 | 0.027082795976270318 |
| 0.22 | 0.012458471760797304 | 0.0201392342118349 | 0.019114009444569407 | 0.0105342362678706 | 0.025482625482625518 | 0.019027484143763207 | 0.02376063361689649 | 0.018765432098765408 | 0.023717328170377507 | 0.018571060098013906 |
| 0.24000000000000005 | 0.005537098560354372 | 0.014420686225758301 | 0.013267371261524604 | 0.007524454477050408 | 0.0205920205920206 | 0.012080942313500499 | 0.013787034320915204 | 0.015061728395061709 | 0.015004840271055201 | 0.018313128707763707 |
| 0.26 | 0.005260243632336664 | 0.00596718050721034 | 0.007645603777827752 | 0.00476548783546526 | 0.010810810810810801 | 0.0108728480821504 | 0.0211205632150191 | 0.007901234567901231 | 0.009438528557599234 | 0.010575187000257901 |
| 0.28000000000000008 | 0.006090808416389812 | 0.010442565887618107 | 0.005621767483696874 | 0.002758966641585151 | 0.00978120978120978 | 0.0072485653881002735 | 0.00880023467292462 | 0.007901234567901231 | 0.0048402710551790915 | 0.006448284756254842 |
| 0.3000000000000001 | 0.00221483942414175 | 0.004972650422675292 | 0.005172026085001124 | 0.0012540757461750704 | 0.00720720720720721 | 0.005436424041075202 | 0.00880023467292462 | 0.004444444444444442 | 0.003630203291384321 | 0.00438483363425329 |
| 0.32000000000000012 | 0.0016611295681063108 | 0.00422675285927399 | 0.002023836294130871 | 0.00100326059694006 | 0.007979407979407983 | 0.00241618846270009 | 0.007333528894103844 | 0.00395061728395062 | 0.003388189738625362 | 0.004642765024503482 |
| 0.34 | 0.0008305647840531565 | 0.002237692690203879 | 0.00044974139869575016 | 0.0002508151492350141 | 0.003088803088803091 | 0.00332225913621262 | 0.004400117336462311 | 0.002469135802469141 | 0.003630203291384321 | 0.0015475883415011604 |
| 0.3600000000000001 | 0.00110741971207087 | 0.0014917951268025905 | 0.00134922419608725 | 0.0012540757461750704 | 0.00592020592020592 | 0.0006040471156750232 | 0.007626870049868 | 0.003456790123456789 | 0.0016940948693126808 | 0.0010317255610007704 |
| 0.38000000000000012 | 0.0008305647840531565 | 0.0014917951268025905 | 0.00044974139869575016 | 0.0002508151492350141 | 0.00231660231660232 | 0.0003020235578375112 | 0.005866823115283074 | 0.0012345679012345705 | 0.0007260406582768644 | 0.0005158627805003875 |
| 0.4 | 0.0005537098560354373 | 0.0012431626056688201 | 0.0008994827973915003 | 0.0002508151492350141 | 0.0018018018018018005 | 0.0006040471156750232 | 0.00205338809034908 | 0.0007407407407407411 | 0.00048402710551790924 | 0.00025793139025019314 |
### Chart: unc-49 A2
| Category | unc-49_REX_A2_01 | unc-49_REX_A2_02 | unc-49_REX_A2_03 | unc-49_REX_A2_04 | unc-49_REX_A2_05 | unc-49_REX_A2_06 | unc-49_REX_A2_07 | unc-49_REX_A2_08 | unc-49_REX_A2_09 | unc-49_REX_A2_10 |
|---|---|---|---|---|---|---|---|---|---|---|
| 0 | None | None | None | None | None | None | None | None | None | None |
| 2.0000000000000007E-2 | 0.08953341740226993 | 0.128226025536539 | 0.09666586711441595 | 0.08421052631578953 | 0.08830950378469306 | 0.11926605504587204 | 0.08957373271889403 | 0.08954845256215124 | 0.10438047559449297 | 0.09925238463521528 |
| 4.0000000000000015E-2 | 0.11696090794451403 | 0.154577560445531 | 0.12473015111537503 | 0.12727272727272695 | 0.11333052985702298 | 0.12300373768263703 | 0.12355990783410098 | 0.10933536276002002 | 0.129411764705882 | 0.12786800721835495 |
| 6.0000000000000019E-2 | 0.115069356872636 | 0.13909263787014406 | 0.14511873350923507 | 0.124401913875598 | 0.126156433978133 | 0.12810057764186195 | 0.13940092165898593 | 0.13089802130897996 | 0.13692115143929906 | 0.12064965197215803 |
| 8.0000000000000029E-2 | 0.09899117276166465 | 0.100516164085846 | 0.11489565843127804 | 0.11961722488038302 | 0.10807401177460105 | 0.09242269792728508 | 0.11261520737327205 | 0.10755961440892903 | 0.108886107634543 | 0.11394689352925999 |
| 0.1 | 0.09615384615384627 | 0.0703613148600924 | 0.09474694171264096 | 0.124401913875598 | 0.10702270815811606 | 0.0801902820251444 | 0.10397465437788003 | 0.0842212075088787 | 0.08986232790988745 | 0.08894044856921897 |
| 0.12000000000000002 | 0.06904161412358133 | 0.0488997555012225 | 0.06836171743823463 | 0.08612440191387565 | 0.0834735071488646 | 0.055385660890248024 | 0.07632488479262671 | 0.07179096905124305 | 0.06157697121401751 | 0.07862851250322249 |
| 0.14000000000000001 | 0.0573770491803279 | 0.0331431676174952 | 0.055408970976253316 | 0.0679425837320574 | 0.06497056349873842 | 0.0499490316004077 | 0.061347926267281104 | 0.053018772196854386 | 0.0475594493116396 | 0.0564578499613302 |
| 0.16 | 0.052648171500630496 | 0.0263515349089921 | 0.0448548812664908 | 0.041148325358851684 | 0.04478553406223723 | 0.0455317703024125 | 0.048099078341013804 | 0.04337899543379 | 0.040050062578222786 | 0.05233307553493174 |
| 0.18000000000000005 | 0.037515762925599015 | 0.01738657973376801 | 0.04149676181338452 | 0.04880382775119622 | 0.039949537426408714 | 0.03669724770642203 | 0.04118663594470052 | 0.03221714865550482 | 0.0315394242803504 | 0.0360917762309874 |
| 0.2 | 0.0324716267339218 | 0.011138277641945101 | 0.025905492923962607 | 0.0373205741626794 | 0.031959629941127 | 0.022426095820591206 | 0.031970046082949316 | 0.0256215119228818 | 0.022277847309136418 | 0.0262954369682908 |
| 0.22 | 0.024590163934426198 | 0.007063298016843253 | 0.0206284480690813 | 0.019138755980861205 | 0.025441547518923534 | 0.0159700985389059 | 0.021601382488479336 | 0.019025875190258813 | 0.0147684605757196 | 0.017014694508893993 |
| 0.24000000000000005 | 0.018600252206809605 | 0.007878293941863625 | 0.010554089709762505 | 0.012440191387559803 | 0.021236333052985706 | 0.010193679918450601 | 0.016705069124424002 | 0.016235413495687506 | 0.011764705882352905 | 0.018561484918793506 |
| 0.26 | 0.013556116015132404 | 0.005704971475142624 | 0.0119932837610938 | 0.015311004784689 | 0.0134566862910008 | 0.00951410125722052 | 0.015552995391705104 | 0.0136986301369863 | 0.0105131414267835 | 0.0128899200824955 |
| 0.28000000000000008 | 0.011664564943253507 | 0.00353164900842162 | 0.006956104581434402 | 0.013397129186602904 | 0.00820016820857864 | 0.005776418620455324 | 0.010656682027649795 | 0.013444951801116201 | 0.009011264080100126 | 0.011858726475895798 |
| 0.3000000000000001 | 0.005359394703657 | 0.002173322466721001 | 0.00551691053010314 | 0.0066985645933014416 | 0.00820016820857864 | 0.004757050628610258 | 0.007200460829493092 | 0.00938609842719432 | 0.007759699624530666 | 0.006960556844547562 |
| 0.32000000000000012 | 0.010403530895334204 | 0.0008149959250203754 | 0.0021587910769968817 | 0.003827751196172251 | 0.006938603868797313 | 0.002718314644920152 | 0.003456221198156681 | 0.00532724505327245 | 0.003504380475594492 | 0.006960556844547562 |
| 0.34 | 0.00725094577553594 | 0.0008149959250203754 | 0.0031182537778843816 | 0.0019138755980861206 | 0.00504625735912532 | 0.0010193679918450605 | 0.002592165898617511 | 0.0050735667174023336 | 0.005006257822277852 | 0.00386697602474865 |
| 0.3600000000000001 | 0.00409836065573771 | 0.0008149959250203754 | 0.00119932837610938 | 0.0 | 0.0035744322960471015 | 0.0013591573224600705 | 0.00201612903225806 | 0.0035514967021816318 | 0.00300375469336671 | 0.00360917762309874 |
| 0.38000000000000012 | 0.0015762925598991205 | 0.000271665308340125 | 0.00119932837610938 | 0.0 | 0.0033641715727502123 | 0.0013591573224600705 | 0.001728110599078341 | 0.0025367833587011707 | 0.00225281602002503 | 0.00206238721319928 |
| 0.4 | 0.003783102143757882 | 0.0010866612333605 | 0.000719597025665627 | 0.0 | 0.002943650126156431 | 0.00169894665307509 | 0.0008640552995391713 | 0.0020294266869609317 | 0.0007509386733416775 | 0.00283578241814901 |
### Chart: unc-49 A3
| Category | unc-49_REX_A3_01 | unc-49_REX_A3_02 | unc-49_REX_A3_03 | unc-49_REX_A3_04 | unc-49_REX_A3_05 | unc-49_REX_A3_06 | unc-49_REX_A3_07 | unc-49_REX_A3_08 | unc-49_REX_A3_09 | unc-49_REX_A3_10 |
|---|---|---|---|---|---|---|---|---|---|---|
| 0 | None | None | None | None | None | None | None | None | None | None |
| 2.0000000000000007E-2 | 0.0954930834448907 | 0.11076130509444797 | 0.110148514851485 | 0.08715457030063774 | 0.09028177113283499 | 0.09166235111341275 | 0.09892729439809307 | 0.0977312390924956 | 0.09721839713577528 | 0.10738653218629503 |
| 4.0000000000000015E-2 | 0.14904060687193207 | 0.14167143674871197 | 0.128712871287129 | 0.12632857576677795 | 0.13110983323749306 | 0.123511134127395 | 0.12097735399284895 | 0.12739965095986 | 0.14210961167722405 | 0.145060812815188 |
| 6.0000000000000019E-2 | 0.145470771976796 | 0.1525472238122501 | 0.129950495049505 | 0.128454296993623 | 0.14318573893042005 | 0.13775245986535506 | 0.1287246722288439 | 0.13821989528795806 | 0.138804736987056 | 0.13438148917235204 |
| 8.0000000000000029E-2 | 0.12271307452030303 | 0.11419576416714403 | 0.103960396039604 | 0.12238080777406597 | 0.10897067280046002 | 0.11885033661315397 | 0.09833134684147804 | 0.1012216404886561 | 0.114844395483338 | 0.111242954612875 |
| 0.1 | 0.09683177153056671 | 0.08471665712650263 | 0.08622112211221122 | 0.09019131491041603 | 0.09919493962047153 | 0.09839461418953914 | 0.08581644815256267 | 0.093542757417103 | 0.09832002203249798 | 0.08899436369029971 |
| 0.12000000000000002 | 0.05622489959839358 | 0.0586720091585575 | 0.0680693069306931 | 0.0786516853932584 | 0.0721679125934445 | 0.07871569135163135 | 0.060786650774731825 | 0.09598603839441544 | 0.071054805838612 | 0.057846336398694714 |
| 0.14000000000000001 | 0.04953145917001341 | 0.05638236977676018 | 0.05445544554455448 | 0.057090798663832404 | 0.0529039677975848 | 0.0644743656136717 | 0.05303933253873662 | 0.07085514834205928 | 0.050399339025062 | 0.054286561851082826 |
| 0.16 | 0.03480589022757701 | 0.033199771036061795 | 0.0441419141914191 | 0.0467658669905861 | 0.04629097182288673 | 0.040134645261522495 | 0.03992848629320619 | 0.04886561954624782 | 0.045992839438171326 | 0.0424206466923761 |
| 0.18000000000000005 | 0.029451137884872816 | 0.026903262736119118 | 0.03176567656765681 | 0.03127846948071671 | 0.04226566992524442 | 0.0359917141377525 | 0.0375446960667461 | 0.037347294938918 | 0.03359955935004128 | 0.0358943933550875 |
| 0.2 | 0.0218652387327086 | 0.017744705208929606 | 0.022689768976897715 | 0.0309747950197388 | 0.0264519838987924 | 0.028482651475919208 | 0.0292014302741359 | 0.031064572425829013 | 0.02451115395207931 | 0.0284781963808959 |
| 0.22 | 0.0169567157518965 | 0.010589582140812804 | 0.0173267326732673 | 0.024293956878226514 | 0.020989074180563515 | 0.022009321595028514 | 0.01668653158522051 | 0.0160558464223386 | 0.019003029468466014 | 0.0166122812221893 |
| 0.24000000000000005 | 0.013386880856760404 | 0.012020606754436199 | 0.0181518151815182 | 0.015791071970847302 | 0.015526164462334704 | 0.0170895908855515 | 0.015494636471990494 | 0.0129144851657941 | 0.0134949049848527 | 0.013052506674577304 |
| 0.26 | 0.010263275323516305 | 0.007441327990841444 | 0.0123762376237624 | 0.0160947464318251 | 0.011213341000575 | 0.00958052822371828 | 0.007747318235995232 | 0.007678883071553232 | 0.008262186725419996 | 0.008009492732126977 |
| 0.28000000000000008 | 0.008478357875948249 | 0.00715512306811677 | 0.00618811881188119 | 0.012450652900091093 | 0.012938470385278901 | 0.0100983946141895 | 0.008939213349225275 | 0.005933682373472952 | 0.007435968052877992 | 0.00593295757935331 |
| 0.3000000000000001 | 0.0049085229808121435 | 0.0040068689181453915 | 0.00618811881188119 | 0.006377163680534473 | 0.006037952846463478 | 0.008285862247540134 | 0.008939213349225275 | 0.006282722513089013 | 0.00358028091434866 | 0.005043013942450312 |
| 0.32000000000000012 | 0.0040160642570281095 | 0.0014310246136233498 | 0.00412541254125413 | 0.0036440935317339817 | 0.003162737205290402 | 0.004919730709476962 | 0.004171632896305132 | 0.0027923211169284512 | 0.00358028091434866 | 0.005339661821417982 |
| 0.34 | 0.00312360553324409 | 0.0005724098454493422 | 0.00247524752475248 | 0.002125721226844821 | 0.004312823461759632 | 0.004660797514241331 | 0.00476758045292014 | 0.002094240837696341 | 0.002203249793445331 | 0.0008899436369029973 |
| 0.3600000000000001 | 0.000892458723784026 | 0.0014310246136233498 | 0.0020627062706270616 | 0.003947767992711812 | 0.0014376078205865408 | 0.0015535991714137804 | 0.00119189511323004 | 0.0013961605584642204 | 0.003304874690168 | 0.0038564224265796487 |
| 0.38000000000000012 | 0.000892458723784026 | 0.000858614768174013 | 0.00123762376237624 | 0.0024293956878226517 | 0.002875215641173089 | 0.002330398757120661 | 0.00357568533969011 | 0.00104712041884817 | 0.0019278435692646708 | 0.0014832393948383301 |
| 0.4 | 0.0 | 0.00028620492272467106 | 0.0 | 0.002125721226844821 | 0.003162737205290402 | 0.002330398757120661 | 0.0017878426698450505 | 0.00034904013961605614 | 0.0013770311209033309 | 0.00118659151587066 |
### Chart: unc-49 A5
| Category | unc-49_A5_40_30_03 | unc-49_A5_40_30_04 | unc-49_REX_A5_01 | unc-49_REX_A5_02 | unc-49_REX_A5_03 | unc-49_REX_A5_04 | unc-49_REX_A5_05 | unc-49_REX_A5_06 | unc-49_REX_A5_09 | unc-49_REX_A5_10 |
|---|---|---|---|---|---|---|---|---|---|---|
| 0 | None | None | None | None | None | None | None | None | None | None |
| 2.0000000000000007E-2 | 0.110406091370558 | 0.12253937007874002 | 0.10357934748178703 | 0.08762886597938147 | 0.115229885057471 | 0.09337539432176666 | 0.09864672364672371 | 0.139248704663212 | 0.09631391200951249 | 0.0944366526414212 |
| 4.0000000000000015E-2 | 0.139593908629442 | 0.144685039370079 | 0.125752296484004 | 0.128163074039363 | 0.13563218390804593 | 0.155520504731861 | 0.12037037037037 | 0.14702072538860095 | 0.14783987316686506 | 0.15942028985507212 |
| 6.0000000000000019E-2 | 0.121827411167513 | 0.15600393700787407 | 0.12670256572695596 | 0.13776944704779812 | 0.135057471264368 | 0.13470031545741307 | 0.12072649572649605 | 0.10297927461139902 | 0.13040031708283806 | 0.135577372604021 |
| 8.0000000000000029E-2 | 0.0780456852791878 | 0.11023622047244105 | 0.10231232182451698 | 0.11621368322399303 | 0.10862068965517205 | 0.11198738170346997 | 0.10470085470085502 | 0.0952072538860104 | 0.11058263971462498 | 0.109864422627396 |
| 0.1 | 0.052664974619289304 | 0.0836614173228347 | 0.09534368070953436 | 0.10988753514526697 | 0.08735632183908053 | 0.09779179810725552 | 0.0811965811965812 | 0.054404145077720185 | 0.097106619104241 | 0.08695652173913045 |
| 0.12000000000000002 | 0.03997461928934012 | 0.0575787401574803 | 0.07507127019322142 | 0.08294283036551078 | 0.0683908045977012 | 0.06214511041009458 | 0.06339031339031342 | 0.0356217616580311 | 0.07649623464130002 | 0.06778868630201033 |
| 0.14000000000000001 | 0.029187817258883208 | 0.04183070866141728 | 0.0582831802343997 | 0.0634957825679475 | 0.05459770114942535 | 0.0511041009463722 | 0.05733618233618232 | 0.031088082901554407 | 0.0586603250099088 | 0.04862085086489008 |
| 0.16 | 0.021573604060913715 | 0.025098425196850398 | 0.04751346214760853 | 0.048734770384254895 | 0.0439655172413793 | 0.03943217665615142 | 0.048076923076923114 | 0.0297927461139896 | 0.0491478398731669 | 0.03973819541841982 |
| 0.18000000000000005 | 0.015862944162436502 | 0.0236220472440945 | 0.03769401330376941 | 0.035613870665417116 | 0.0339080459770115 | 0.02996845425867509 | 0.0441595441595442 | 0.0375647668393782 | 0.0360681728101467 | 0.03179055633473588 |
| 0.2 | 0.012055837563451795 | 0.016732283464566903 | 0.024390243902439 | 0.02811621368322401 | 0.01982758620689661 | 0.022712933753943208 | 0.026353276353276407 | 0.022668393782383413 | 0.021403091557669413 | 0.02290790088826552 |
| 0.22 | 0.00824873096446701 | 0.016240157480315008 | 0.018371872030408607 | 0.018041237113402105 | 0.0166666666666667 | 0.017034700315457403 | 0.018874643874643892 | 0.0161917098445596 | 0.01981767736821241 | 0.0172978027115475 |
| 0.24000000000000005 | 0.0038071065989847717 | 0.0029527559055118092 | 0.014887551472917307 | 0.018041237113402105 | 0.009482758620689667 | 0.011671924290220804 | 0.016025641025641 | 0.013601036269430109 | 0.010701545778834701 | 0.011687704534829401 |
| 0.26 | 0.0038071065989847717 | 0.001968503937007871 | 0.012987012987013 | 0.013589503280224903 | 0.007471264367816093 | 0.004416403785488963 | 0.010683760683760705 | 0.011658031088082901 | 0.013476020610384503 | 0.007480130902290791 |
| 0.28000000000000008 | 0.0019035532994923904 | 0.0029527559055118092 | 0.008869179600886927 | 0.008903467666354274 | 0.007183908045977011 | 0.007570977917981072 | 0.0067663817663817715 | 0.005181347150259074 | 0.00515259611573524 | 0.006077606358111272 |
| 0.3000000000000001 | 0.0038071065989847717 | 0.0009842519685039376 | 0.002850807728856511 | 0.00515463917525773 | 0.00402298850574713 | 0.00252365930599369 | 0.009971509971509977 | 0.005181347150259074 | 0.005945303210463734 | 0.005142589995324922 |
| 0.32000000000000012 | 0.0 | 0.0029527559055118092 | 0.005384859043395631 | 0.002577319587628871 | 0.00258620689655172 | 0.002839116719242901 | 0.00534188034188034 | 0.0019430051813471509 | 0.00435988902100674 | 0.004207573632538572 |
| 0.34 | 0.0 | 0.0009842519685039376 | 0.0022172949002217312 | 0.002811621368322401 | 0.0028735632183908015 | 0.002839116719242901 | 0.004273504273504271 | 0.00129533678756477 | 0.00435988902100674 | 0.0037400654511454017 |
| 0.3600000000000001 | 0.002538071065989851 | 0.0 | 0.001583782071586951 | 0.0014058106841612 | 0.0008620689655172415 | 0.0012618296529968499 | 0.002849002849002851 | 0.0019430051813471509 | 0.00118906064209275 | 0.00140252454417952 |
| 0.38000000000000012 | 0.00126903553299492 | 0.0004921259842519694 | 0.0012670256572695595 | 0.0011715089034676704 | 0.0011494252873563199 | 0.0006309148264984234 | 0.0017806267806267804 | 0.0019430051813471509 | 0.00118906064209275 | 0.0009350163627863497 |
| 0.4 | 0.0006345177664974623 | 0.0 | 0.0009502692429521708 | 0.0018744142455482705 | 0.0014367816091954001 | 0.0 | 0.0014245014245014205 | 0.0006476683937823836 | 0.0 | 0.0009350163627863497 |
### Chart: glr-1 A1
| Category | glr-1(n2461)_A1_01 | glr-1(n2461)_A1_02 | glr-1(n2461)_A1_03 | glr-1(n2461)_A1_04 | glr-1(n2461)_A1_05 | glr-1(n2461)_A1_06 | glr-1(n2461)_A1_07 | glr-1(n2461)_A1_08 | glr-1(n2461)_A1_09 | glr-1(n2461)_A1_10 | glr-1(n2461)_A1_11 | glr-1(n2461)_A1_12 | glr-1(n2461)_A1_13 | glr-1(n2461)_A1_14 | glr-1(n2461)_A1_15 | glr-1(n2461)_A1_16 | glr-1(n2461)_A1_17 | glr-1(n2461)_A1_18 | glr-1(n2461)_A1_19 | glr-1(n2461)_A1_20 |
|---|---|---|---|---|---|---|---|---|---|---|---|---|---|---|---|---|---|---|---|---|
| 0 | None | None | None | None | None | None | None | None | None | None | None | None | None | None | None | None | None | None | None | None |
| 2.0000000000000007E-2 | 0.0681818181818182 | 0.059023066485753096 | 0.06845238095238104 | 0.06174819566960707 | 0.06641509433962263 | 0.06092916984006089 | 0.05539358600583092 | 0.05861828332170271 | 0.062403697996918354 | 0.06390328151986181 | 0.06795469686875423 | 0.07700312174817901 | 0.09768451519536904 | 0.0609919571045576 | 0.0683903252710592 | 0.059322033898305135 | 0.0619362597714973 | 0.0595408895265423 | 0.0755451713395639 | 0.07916666666666673 |
| 4.0000000000000015E-2 | 0.08264462809917364 | 0.09769335142469471 | 0.107142857142857 | 0.101844426623897 | 0.09358490566037747 | 0.0913937547600914 | 0.08600583090379015 | 0.0823447313328681 | 0.10862865947611708 | 0.0984455958549223 | 0.09260493004663563 | 0.11758584807492202 | 0.11649782923299598 | 0.09919571045576413 | 0.10091743119266094 | 0.08050847457627124 | 0.09981960312687915 | 0.10473457675753209 | 0.10514018691588803 | 0.07916666666666673 |
| 6.0000000000000019E-2 | 0.11639118457300303 | 0.10312075983717803 | 0.121279761904762 | 0.14274258219727315 | 0.12377358490566005 | 0.123381568926123 | 0.12026239067055403 | 0.11235170969992997 | 0.11864406779661005 | 0.123488773747841 | 0.126582278481013 | 0.112382934443288 | 0.12156295224312604 | 0.122654155495979 | 0.12427022518765606 | 0.10169491525423703 | 0.11545399879735396 | 0.13414634146341506 | 0.10825545171339603 | 0.110416666666667 |
| 8.0000000000000029E-2 | 0.14462809917355393 | 0.11601085481682498 | 0.13392857142857095 | 0.141138732959102 | 0.14490566037735805 | 0.1439451637471441 | 0.13265306122449 | 0.13677599441730606 | 0.14560862865947594 | 0.13644214162348905 | 0.1479013990672881 | 0.14255983350676407 | 0.15340086830680205 | 0.14611260053619307 | 0.15095913261050906 | 0.156779661016949 | 0.14191220685508107 | 0.14921090387374505 | 0.13006230529595 | 0.129861111111111 |
| 0.1 | 0.167355371900826 | 0.152645861601085 | 0.150297619047619 | 0.14354450681635905 | 0.15245283018867906 | 0.144706778370145 | 0.15597667638484 | 0.166085136078158 | 0.148690292758089 | 0.13126079447323005 | 0.15722851432378396 | 0.106139438085328 | 0.14254703328509408 | 0.14611260053619307 | 0.15512927439532906 | 0.12288135593220302 | 0.187612748045701 | 0.14490674318507907 | 0.13940809968847406 | 0.154166666666667 |
| 0.12000000000000002 | 0.08057851239669424 | 0.08005427408412483 | 0.078125 | 0.07778668805132326 | 0.09509433962264154 | 0.08149276466108149 | 0.10349854227405203 | 0.06629448709002092 | 0.07935285053929123 | 0.07512953367875648 | 0.09193870752831448 | 0.06659729448491163 | 0.07742402315484803 | 0.0824396782841823 | 0.0642201834862385 | 0.08050847457627124 | 0.0799759470835839 | 0.07890961262553801 | 0.08255451713395645 | 0.07222222222222223 |
| 0.14000000000000001 | 0.04201101928374663 | 0.06512890094979652 | 0.0498511904761905 | 0.04811547714514843 | 0.04603773584905663 | 0.0502665651180503 | 0.05539358600583092 | 0.05373342637822752 | 0.04699537750385208 | 0.062176165803108814 | 0.0439706862091939 | 0.06763787721123833 | 0.0463096960926194 | 0.05965147453083112 | 0.0525437864887406 | 0.08050847457627124 | 0.051713770294648236 | 0.050215208034433315 | 0.0576323987538941 | 0.04791666666666672 |
| 0.16 | 0.04269972451790632 | 0.05563093622795122 | 0.061011904761904795 | 0.05854049719326381 | 0.04603773584905663 | 0.05331302361005332 | 0.053206997084548124 | 0.0502442428471738 | 0.058551617873651804 | 0.06131260794473235 | 0.049300466355762816 | 0.054110301768990614 | 0.05209840810419682 | 0.0542895442359249 | 0.05921601334445373 | 0.0127118644067797 | 0.042092603728202026 | 0.050215208034433315 | 0.04906542056074772 | 0.0513888888888889 |
| 0.18000000000000005 | 0.05165289256198348 | 0.05563093622795122 | 0.052827380952381035 | 0.0417000801924619 | 0.0445283018867925 | 0.0525514089870526 | 0.04591836734693882 | 0.048150732728541513 | 0.050077041602465296 | 0.039723661485319514 | 0.0473017988007995 | 0.05931321540062432 | 0.038350217076700416 | 0.04825737265415548 | 0.047539616346955804 | 0.0635593220338983 | 0.0469031870114251 | 0.043758967001434716 | 0.04283489096573211 | 0.0395833333333333 |
| 0.2 | 0.0392561983471074 | 0.050203527815468114 | 0.034226190476190514 | 0.03448275862068972 | 0.040000000000000015 | 0.04265041888804262 | 0.043731778425656 | 0.0502442428471738 | 0.0431432973805855 | 0.037996545768566516 | 0.027981345769487017 | 0.028095733610822106 | 0.0195369030390738 | 0.031501340482573734 | 0.043369474562135114 | 0.038135593220339 | 0.04028863499699343 | 0.0337159253945481 | 0.036604361370716515 | 0.04305555555555557 |
| 0.22 | 0.0351239669421488 | 0.031207598371777515 | 0.015625 | 0.02485966319166 | 0.030188679245282988 | 0.035795887281035804 | 0.032798833819242 | 0.0460572226099093 | 0.02311248073959939 | 0.021588946459412808 | 0.027315123251165908 | 0.027055150884495317 | 0.027496382054992802 | 0.025469168900804313 | 0.03002502085070889 | 0.0296610169491525 | 0.03247143716175591 | 0.0258249641319943 | 0.027258566978193108 | 0.023611111111111114 |
| 0.24000000000000005 | 0.019972451790633613 | 0.02442333785617372 | 0.0223214285714286 | 0.02004811547714512 | 0.0188679245283019 | 0.0236100533130236 | 0.018950437317784306 | 0.026517794836008392 | 0.0161787365177196 | 0.025043177892918808 | 0.025316455696202493 | 0.0156087408949011 | 0.013024602026049199 | 0.018096514745308306 | 0.010842368640533805 | 0.02542372881355931 | 0.0162357185808779 | 0.0121951219512195 | 0.028816199376947 | 0.0333333333333333 |
| 0.26 | 0.012396694214876 | 0.014925373134328401 | 0.013392857142857107 | 0.012830793905372898 | 0.0158490566037736 | 0.0144706778370145 | 0.021137026239067092 | 0.02023726448011172 | 0.0161787365177196 | 0.0120898100172712 | 0.0159893404397069 | 0.013527575442247709 | 0.010130246020260495 | 0.015415549597855205 | 0.014178482068390298 | 0.0296610169491525 | 0.012627781118460607 | 0.013629842180774695 | 0.018691588785046703 | 0.018055555555555606 |
| 0.28000000000000008 | 0.008264462809917364 | 0.0128900949796472 | 0.008184523809523813 | 0.0168404170008019 | 0.008301886792452834 | 0.007616146230007621 | 0.010932944606414 | 0.012561060711793401 | 0.012326656394453003 | 0.0146804835924007 | 0.017321785476349102 | 0.009365244536940702 | 0.006512301013024604 | 0.009383378016085795 | 0.005004170141784822 | 0.0127118644067797 | 0.0060132291040288655 | 0.00573888091822095 | 0.010124610591900299 | 0.0166666666666667 |
| 0.3000000000000001 | 0.008264462809917364 | 0.011533242876526495 | 0.00669642857142857 | 0.005613472333600643 | 0.0060377358490566 | 0.00380807311500381 | 0.013119533527696795 | 0.011165387299371901 | 0.008474576271186448 | 0.006908462867012092 | 0.004663557628247828 | 0.006243496357960462 | 0.007959479015918968 | 0.008042895442359253 | 0.003336113427856551 | 0.008474576271186448 | 0.0060132291040288655 | 0.010043041606886701 | 0.00856697819314642 | 0.011805555555555609 |
| 0.32000000000000012 | 0.007575757575757582 | 0.0074626865671641816 | 0.005208333333333334 | 0.00641539695268645 | 0.00452830188679245 | 0.00304645849200305 | 0.008746355685131197 | 0.004884856943475228 | 0.0061633281972265 | 0.0138169257340242 | 0.0066622251832111935 | 0.00416233090530697 | 0.002170767004341531 | 0.006032171581769442 | 0.004170141784820684 | 0.008474576271186448 | 0.005411906193625982 | 0.00286944045911047 | 0.00856697819314642 | 0.00763888888888889 |
| 0.34 | 0.005509641873278242 | 0.004748982360922662 | 0.002976190476190481 | 0.0024057738572574217 | 0.00150943396226415 | 0.006854531607006848 | 0.00291545189504373 | 0.004884856943475228 | 0.003852080123266559 | 0.004317789291882562 | 0.001332445036642241 | 0.009365244536940702 | 0.002170767004341531 | 0.006032171581769442 | 0.004170141784820684 | 0.008474576271186448 | 0.003006614552014432 | 0.00215208034433286 | 0.007788161993769474 | 0.00555555555555556 |
| 0.3600000000000001 | 0.002066115702479341 | 0.00407055630936228 | 0.0014880952380952404 | 0.0024057738572574217 | 0.0022641509433962317 | 0.00304645849200305 | 0.0021865889212828 | 0.00139567341242149 | 0.0015408320493066308 | 0.006908462867012092 | 0.002664890073284481 | 0.003121748178980231 | 0.00144717800289436 | 0.004021447721179624 | 0.0025020850708924107 | 0.004237288135593224 | 0.003006614552014432 | 0.007173601147776184 | 0.005451713395638635 | 0.006250000000000002 |
| 0.38000000000000012 | 0.0013774104683195608 | 0.005427408412483043 | 0.0007440476190476194 | 0.0016038492381716101 | 0.0022641509433962317 | 0.002284843869002291 | 0.00145772594752187 | 0.0034891835310537312 | 0.003081664098613251 | 0.004317789291882562 | 0.0006662225183211195 | 0.00416233090530697 | 0.002170767004341531 | 0.002010723860589811 | 0.0 | 0.0127118644067797 | 0.0 | 0.00430416068866571 | 0.00389408099688474 | 0.001388888888888891 |
| 0.4 | 0.0013774104683195608 | 0.0013568521031207613 | 0.0014880952380952404 | 0.0024057738572574217 | 0.00150943396226415 | 0.0007616146230007622 | 0.00291545189504373 | 0.0006978367062107474 | 0.002311248073959939 | 0.00259067357512953 | 0.001332445036642241 | 0.00416233090530697 | 0.00144717800289436 | 0.0013404825737265414 | 0.0008340283569641374 | 0.004237288135593224 | 0.0006013229104028867 | 0.00143472022955524 | 0.0 | 0.0027777777777777827 |
### Chart: glr-1 A3
| Category | glr-1(n2461)_A3_01 | glr-1(n2461)_A3_02 | glr-1(n2461)_A3_03 | glr-1(n2461)_A3_04 | glr-1(n2461)_A3_05 | glr-1(n2461)_A3_06 | glr-1(n2461)_A3_07 | glr-1(n2461)_A3_08 | glr-1(n2461)_A3_09 | glr-1(n2461)_A3_10 | glr-1(n2461)_A3_11 | glr-1(n2461)_A3_12 | glr-1(n2461)_A3_13 | glr-1(n2461)_A3_14 | glr-1(n2461)_A3_15 | glr-1(n2461)_A3_16 | glr-1(n2461)_A3_17 | glr-1(n2461)_A3_18 | glr-1(n2461)_A3_19 |
|---|---|---|---|---|---|---|---|---|---|---|---|---|---|---|---|---|---|---|---|
| 0 | None | None | None | None | None | None | None | None | None | None | None | None | None | None | None | None | None | None | None |
| 2.0000000000000007E-2 | 0.0920754716981132 | 0.0807504078303426 | 0.06942148760330583 | 0.0858895705521472 | 0.0696969696969697 | 0.08782742681047773 | 0.08660785886126704 | 0.0889309366130558 | 0.08076225045372051 | 0.09560723514211893 | 0.0784140969162996 | 0.07084468664850142 | 0.08845029239766088 | 0.0648298217179903 | 0.07916986933128363 | 0.0782280867106503 | 0.0856685348278623 | 0.089863407620417 | 0.07348901098901103 |
| 4.0000000000000015E-2 | 0.113207547169811 | 0.11256117455138705 | 0.09586776859504136 | 0.102760736196319 | 0.100757575757576 | 0.105546995377504 | 0.10665597433841204 | 0.10974456007568606 | 0.11070780399273998 | 0.10766580534022405 | 0.09867841409691633 | 0.10717529518619404 | 0.105263157894737 | 0.12155591572123205 | 0.10069177555726404 | 0.13289349670122508 | 0.10168134507606102 | 0.08483105679367367 | 0.10027472527472503 |
| 6.0000000000000019E-2 | 0.145660377358491 | 0.15089722675367 | 0.11404958677686 | 0.11579754601227005 | 0.112878787878788 | 0.11710323574730402 | 0.14434643143544515 | 0.13623462630085095 | 0.12885662431941888 | 0.12489233419465999 | 0.113656387665198 | 0.11171662125340605 | 0.13230994152046807 | 0.12317666126418206 | 0.12067640276710204 | 0.15928369462771005 | 0.12489991993594902 | 0.10927390366642703 | 0.12293956043956 |
| 8.0000000000000029E-2 | 0.195471698113208 | 0.15742251223490997 | 0.12396694214876003 | 0.141104294478528 | 0.145454545454545 | 0.198767334360555 | 0.197273456295108 | 0.13718070009460695 | 0.169691470054446 | 0.15934539190353106 | 0.126872246696035 | 0.143505903723887 | 0.16154970760233905 | 0.0940032414910859 | 0.128362797847809 | 0.153628652214892 | 0.140112089671737 | 0.110711718188354 | 0.1270604395604401 |
| 0.1 | 0.12075471698113205 | 0.14437194127243105 | 0.1504132231404961 | 0.174846625766871 | 0.16136363636363593 | 0.144838212634823 | 0.13712910986367297 | 0.165562913907285 | 0.12976406533575294 | 0.161068044788975 | 0.13215859030836993 | 0.14259763851044507 | 0.17909356725146205 | 0.07455429497568883 | 0.16525749423520406 | 0.12441093308199802 | 0.136909527622098 | 0.17181883537023707 | 0.175824175824176 |
| 0.12000000000000002 | 0.0649056603773585 | 0.0693311582381729 | 0.09917355371900828 | 0.0567484662576687 | 0.0674242424242424 | 0.058551617873651804 | 0.057738572574177985 | 0.0520340586565752 | 0.06261343012704175 | 0.0654608096468562 | 0.07048458149779742 | 0.0663033605812897 | 0.07236842105263165 | 0.05510534846029168 | 0.09377401998462725 | 0.07068803016022623 | 0.07606084867894318 | 0.04960460100647021 | 0.0762362637362637 |
| 0.14000000000000001 | 0.05056603773584912 | 0.05791190864600332 | 0.0446280991735537 | 0.04677914110429448 | 0.049242424242424226 | 0.04083204930662562 | 0.045709703287890896 | 0.0378429517502365 | 0.04537205081669693 | 0.0404823428079242 | 0.05814977973568278 | 0.05449591280653952 | 0.05190058479532162 | 0.05348460291734202 | 0.0553420445810915 | 0.05278039585296892 | 0.04803843074459572 | 0.0402588066139468 | 0.03777472527472532 |
| 0.16 | 0.03698113207547172 | 0.0424143556280587 | 0.04793388429752072 | 0.0414110429447853 | 0.05 | 0.036209553158705714 | 0.040096230954290345 | 0.0312204351939451 | 0.0317604355716878 | 0.0327304048234281 | 0.03876651982378849 | 0.048138056312443216 | 0.04020467836257308 | 0.04213938411669372 | 0.0453497309761722 | 0.0339302544769086 | 0.04083266613290634 | 0.0388209920920201 | 0.0384615384615385 |
| 0.18000000000000005 | 0.029433962264150917 | 0.0252854812398042 | 0.04628099173553722 | 0.04217791411042941 | 0.0363636363636364 | 0.04083204930662562 | 0.0376904570970329 | 0.0359508041627247 | 0.039019963702359314 | 0.02842377260981912 | 0.03788546255506611 | 0.032697547683923744 | 0.035087719298245605 | 0.0388978930307942 | 0.0292083013066872 | 0.03016022620169649 | 0.032826261008807 | 0.045291157440690184 | 0.043269230769230796 |
| 0.2 | 0.021132075471698115 | 0.0277324632952692 | 0.0330578512396694 | 0.03680981595092022 | 0.034848484848484886 | 0.02542372881355931 | 0.02967121090617482 | 0.033112582781457 | 0.0317604355716878 | 0.029285099052540908 | 0.03700440528634361 | 0.03723887375113531 | 0.027046783625731007 | 0.0356564019448947 | 0.026902382782475018 | 0.019792648444863305 | 0.03923138510808651 | 0.037383177570093525 | 0.03502747252747252 |
| 0.22 | 0.0173584905660377 | 0.0154975530179445 | 0.019834710743801706 | 0.0291411042944785 | 0.0318181818181818 | 0.026194144838212592 | 0.02004811547714512 | 0.023651844843897797 | 0.017241379310344803 | 0.016365202411714002 | 0.0317180616740088 | 0.0299727520435967 | 0.012426900584795298 | 0.0307941653160454 | 0.0315142198308993 | 0.01790763430725731 | 0.0200160128102482 | 0.0337886412652768 | 0.03708791208791213 |
| 0.24000000000000005 | 0.013584905660377405 | 0.007340946166394782 | 0.031404958677686015 | 0.02453987730061352 | 0.023484848484848515 | 0.013867488443759604 | 0.014434643143544494 | 0.01892147587511831 | 0.01814882032667881 | 0.015503875968992208 | 0.022026431718061693 | 0.02179836512261581 | 0.012426900584795298 | 0.0178282009724473 | 0.0284396617986164 | 0.00942507068803016 | 0.016813450760608507 | 0.03594536304816681 | 0.022664835164835202 |
| 0.26 | 0.00528301886792453 | 0.00489396411092985 | 0.016528925619834708 | 0.009202453987730064 | 0.0128787878787879 | 0.011556240369799698 | 0.00641539695268645 | 0.014191106906338699 | 0.012704174228675109 | 0.011197243755383298 | 0.013215859030837008 | 0.01544050862851951 | 0.007309941520467842 | 0.0194489465153971 | 0.016910069177555703 | 0.011310084825636203 | 0.014411529223378704 | 0.024442846872753412 | 0.014423076923076898 |
| 0.28000000000000008 | 0.008301886792452834 | 0.007340946166394782 | 0.00991735537190083 | 0.006901840490797554 | 0.00833333333333333 | 0.011556240369799698 | 0.0032076984763432202 | 0.010406811731315007 | 0.007259528130671513 | 0.012058570198105105 | 0.0176211453744493 | 0.0118074477747502 | 0.00584795321637427 | 0.024311183144246393 | 0.013835511145272905 | 0.010367577756833205 | 0.0104083266613291 | 0.012940330697340005 | 0.00824175824175824 |
| 0.3000000000000001 | 0.0030188679245283 | 0.007340946166394782 | 0.0066115702479338815 | 0.008435582822085898 | 0.012121212121212099 | 0.0053929121725731916 | 0.004811547714514843 | 0.004730368968779562 | 0.008166969147005444 | 0.003445305770887172 | 0.014096916299559498 | 0.009990917347865584 | 0.003654970760233922 | 0.024311183144246393 | 0.0046118370484242895 | 0.00942507068803016 | 0.005604483586869499 | 0.012940330697340005 | 0.010989010989011 |
| 0.32000000000000012 | 0.0022641509433962317 | 0.002446982055464931 | 0.0049586776859504135 | 0.008435582822085898 | 0.0045454545454545504 | 0.003081664098613251 | 0.004811547714514843 | 0.005676442762535482 | 0.0045372050816696935 | 0.006029285099052542 | 0.008810572687224674 | 0.0063578564940962815 | 0.0043859649122807 | 0.0210696920583468 | 0.0046118370484242895 | 0.002827521206409052 | 0.005604483586869499 | 0.007189072609633364 | 0.004807692307692311 |
| 0.34 | 0.0 | 0.0008156606851549771 | 0.008264462809917364 | 0.00383435582822086 | 0.00303030303030303 | 0.003081664098613251 | 0.0008019246190858065 | 0.006622516556291386 | 0.007259528130671513 | 0.0025839793281653726 | 0.009691629955947145 | 0.002724795640326982 | 0.0 | 0.0178282009724473 | 0.0046118370484242895 | 0.00471253534401508 | 0.004003202562049642 | 0.0043134435657800115 | 0.00549450549450549 |
| 0.3600000000000001 | 0.00150943396226415 | 0.003262642740619901 | 0.0049586776859504135 | 0.0023006134969325207 | 0.003787878787878792 | 0.0015408320493066308 | 0.0016038492381716101 | 0.003784295175023651 | 0.0018148820326678809 | 0.00430663221360896 | 0.007048458149779742 | 0.004541326067211632 | 0.00219298245614035 | 0.00810372771474878 | 0.0046118370484242895 | 0.002827521206409052 | 0.004803843074459573 | 0.005751258087706694 | 0.00343406593406593 |
| 0.38000000000000012 | 0.0007547169811320752 | 0.0016313213703099498 | 0.0 | 0.0023006134969325207 | 0.00681818181818182 | 0.002311248073959939 | 0.0 | 0.005676442762535482 | 0.0009074410163339384 | 0.0008613264427217922 | 0.008810572687224674 | 0.002724795640326982 | 0.0007309941520467842 | 0.012965964343598101 | 0.0030745580322828602 | 0.003770028275212062 | 0.00240192153722978 | 0.0043134435657800115 | 0.00137362637362637 |
| 0.4 | 0.0007547169811320752 | 0.0016313213703099498 | 0.0016528925619834713 | 0.000766871165644172 | 0.0015151515151515208 | 0.0007704160246533132 | 0.0016038492381716101 | 0.0009460737937559138 | 0.0027223230490018117 | 0.0017226528854435805 | 0.002643171806167403 | 0.0018165304268846505 | 0.0 | 0.022690437601296607 | 0.0015372790161414301 | 0.0009425070688030163 | 0.003202562049639711 | 0.0043134435657800115 | 0.00343406593406593 |
### Chart: glr-1 A5
| Category | glr-1(n2461)_A5_01 | glr-1(n2461)_A5_02 | glr-1(n2461)_A5_03 | glr-1(n2461)_A5_04 | glr-1(n2461)_A5_05 | glr-1(n2461)_A5_06 | glr-1(n2461)_A5_07 | glr-1(n2461)_A5_08 | glr-1(n2461)_A5_09 | glr-1(n2461)_A5_10 | glr-1(n2461)_A5_11 | glr-1(n2461)_A5_12 | glr-1(n2461)_A5_13 | glr-1(n2461)_A5_14 | glr-1(n2461)_A5_15 | glr-1(n2461)_A5_16 | glr-1(n2461)_A5_17 | glr-1(n2461)_A5_18 | glr-1(n2461)_A5_19 | glr-1(n2461)_A5_20 |
|---|---|---|---|---|---|---|---|---|---|---|---|---|---|---|---|---|---|---|---|---|
| 0 | None | None | None | None | None | None | None | None | None | None | None | None | None | None | None | None | None | None | None | None |
| 2.0000000000000007E-2 | 0.10439560439560402 | 0.06976744186046513 | 0.0688 | 0.07485029940119763 | 0.1888111888111891 | 0.109540636042403 | 0.12795275590551194 | 0.05583756345177672 | 0.0914760914760915 | 0.0764119601328904 | 0.105263157894737 | 0.14216867469879493 | 0.122866894197952 | 0.10150891632373098 | 0.08783068783068777 | 0.08843537414965998 | 0.09469302809573364 | 0.0777502067824648 | 0.10133843212237095 | 0.08095238095238103 |
| 4.0000000000000015E-2 | 0.0824175824175824 | 0.07751937984496123 | 0.06400000000000003 | 0.08532934131736533 | 0.15384615384615408 | 0.0671378091872792 | 0.12401574803149604 | 0.0355329949238579 | 0.10187110187110203 | 0.11129568106312306 | 0.10889292196007304 | 0.156626506024096 | 0.08532423208191128 | 0.08916323731138548 | 0.08677248677248683 | 0.07346938775510202 | 0.105098855359001 | 0.12324234904880105 | 0.14531548757170215 | 0.09682539682539684 |
| 6.0000000000000019E-2 | 0.08791208791208788 | 0.116279069767442 | 0.08960000000000007 | 0.11976047904191606 | 0.125874125874126 | 0.0742049469964664 | 0.1358267716535429 | 0.0355329949238579 | 0.10395010395010403 | 0.0963455149501661 | 0.10889292196007304 | 0.0867469879518072 | 0.0819112627986348 | 0.120713305898491 | 0.11428571428571403 | 0.102040816326531 | 0.125910509885536 | 0.132340777502068 | 0.13575525812619507 | 0.0984126984126984 |
| 8.0000000000000029E-2 | 0.0824175824175824 | 0.10077519379845003 | 0.12160000000000003 | 0.11826347305389204 | 0.08391608391608396 | 0.04240282685512375 | 0.10433070866141703 | 0.07106598984771573 | 0.0810810810810811 | 0.08305647840531565 | 0.105263157894737 | 0.04819277108433742 | 0.037542662116041 | 0.12757201646090494 | 0.1365079365079361 | 0.11156462585034002 | 0.14047866805411 | 0.16625310173697305 | 0.11281070745697905 | 0.125396825396825 |
| 0.1 | 0.137362637362637 | 0.07364341085271323 | 0.09440000000000003 | 0.08532934131736533 | 0.048951048951049 | 0.0318021201413428 | 0.07283464566929132 | 0.040609137055837616 | 0.06652806652806649 | 0.0730897009966777 | 0.0907441016333938 | 0.036144578313253 | 0.023890784982935197 | 0.0973936899862826 | 0.12804232804232807 | 0.0843537414965986 | 0.105098855359001 | 0.1588089330024811 | 0.06883365200764822 | 0.0761904761904762 |
| 0.12000000000000002 | 0.054945054945054896 | 0.05038759689922482 | 0.0384 | 0.05389221556886231 | 0.041958041958042015 | 0.0353356890459364 | 0.04330708661417322 | 0.0304568527918782 | 0.0582120582120582 | 0.0398671096345515 | 0.052631578947368404 | 0.06024096385542172 | 0.013651877133105804 | 0.0438957475994513 | 0.0687830687830688 | 0.04081632653061222 | 0.0551508844953174 | 0.06203473945409432 | 0.043977055449330796 | 0.046031746031746014 |
| 0.14000000000000001 | 0.0384615384615385 | 0.04651162790697674 | 0.04480000000000002 | 0.038922155688622805 | 0.013986013986014 | 0.00353356890459364 | 0.05708661417322834 | 0.0456852791878173 | 0.024948024948024894 | 0.0365448504983389 | 0.023593466424682397 | 0.036144578313253 | 0.023890784982935197 | 0.04663923182441702 | 0.0518518518518519 | 0.03673469387755101 | 0.0634755463059313 | 0.041356492969396225 | 0.049713193116634836 | 0.0412698412698413 |
| 0.16 | 0.010989010989011 | 0.0387596899224806 | 0.024 | 0.023952095808383197 | 0.013986013986014 | 0.04946996466431102 | 0.0334645669291339 | 0.0203045685279188 | 0.024948024948024894 | 0.04485049833887042 | 0.029038112522686017 | 0.031325301204819314 | 0.013651877133105804 | 0.037037037037037014 | 0.0518518518518519 | 0.028571428571428602 | 0.049947970863683716 | 0.0446650124069479 | 0.03824091778202682 | 0.04285714285714291 |
| 0.18000000000000005 | 0.021978021978022 | 0.0348837209302326 | 0.02240000000000001 | 0.044910179640718625 | 0.020979020979021008 | 0.021201413427561818 | 0.0216535433070866 | 0.0101522842639594 | 0.03950103950103952 | 0.03820598006644521 | 0.029038112522686017 | 0.031325301204819314 | 0.017064846416382305 | 0.019204389574759905 | 0.0529100529100529 | 0.039455782312925215 | 0.0551508844953174 | 0.03225806451612901 | 0.022944550669216107 | 0.0333333333333333 |
| 0.2 | 0.021978021978022 | 0.0387596899224806 | 0.025600000000000008 | 0.020958083832335286 | 0.0 | 0.0353356890459364 | 0.007874015748031498 | 0.040609137055837616 | 0.01871101871101871 | 0.0315614617940199 | 0.03266787658802183 | 0.004819277108433742 | 0.017064846416382305 | 0.016460905349794205 | 0.0317460317460317 | 0.04081632653061222 | 0.031217481789802298 | 0.03391232423490489 | 0.01912045889101341 | 0.0158730158730159 |
| 0.22 | 0.010989010989011 | 0.04651162790697674 | 0.030400000000000007 | 0.020958083832335286 | 0.020979020979021008 | 0.021201413427561818 | 0.001968503937007871 | 0.0304568527918782 | 0.024948024948024894 | 0.024916943521594705 | 0.029038112522686017 | 0.0289156626506024 | 0.04095563139931742 | 0.0233196159122085 | 0.023280423280423308 | 0.024489795918367314 | 0.027055150884495317 | 0.0181968569065343 | 0.0267686424474187 | 0.03650793650793652 |
| 0.24000000000000005 | 0.021978021978022 | 0.03682170542635662 | 0.0288 | 0.023952095808383197 | 0.006993006993006992 | 0.0318021201413428 | 0.007874015748031498 | 0.0304568527918782 | 0.0103950103950104 | 0.028239202657807317 | 0.021778584392014494 | 0.012048192771084298 | 0.017064846416382305 | 0.026063100137174208 | 0.02751322751322752 | 0.032653061224489806 | 0.0176899063475546 | 0.019023986765922207 | 0.0152963671128107 | 0.028571428571428602 |
| 0.26 | 0.021978021978022 | 0.031007751937984492 | 0.040000000000000015 | 0.03143712574850301 | 0.0 | 0.045936395759717315 | 0.00393700787401575 | 0.0355329949238579 | 0.027027027027027015 | 0.0348837209302326 | 0.0199637023593466 | 0.009638554216867474 | 0.04436860068259392 | 0.019204389574759905 | 0.0137566137566138 | 0.028571428571428602 | 0.016649323621227903 | 0.0074441687344913255 | 0.0152963671128107 | 0.0412698412698413 |
| 0.28000000000000008 | 0.021978021978022 | 0.02906976744186051 | 0.0208 | 0.025449101796407202 | 0.0 | 0.045936395759717315 | 0.013779527559055104 | 0.0304568527918782 | 0.020790020790020798 | 0.024916943521594705 | 0.01633393829401091 | 0.021686746987951807 | 0.0273037542662116 | 0.016460905349794205 | 0.009523809523809523 | 0.02993197278911562 | 0.008324661810613945 | 0.00909842845326716 | 0.01912045889101341 | 0.025396825396825397 |
| 0.3000000000000001 | 0.021978021978022 | 0.025193798449612406 | 0.04480000000000002 | 0.025449101796407202 | 0.006993006993006992 | 0.02826855123674908 | 0.001968503937007871 | 0.05583756345177672 | 0.022869022869022912 | 0.019933554817275708 | 0.0199637023593466 | 0.00240963855421687 | 0.037542662116041 | 0.0233196159122085 | 0.008465608465608473 | 0.02585034013605441 | 0.007284079084287204 | 0.004135649296939624 | 0.0152963671128107 | 0.014285714285714301 |
| 0.32000000000000012 | 0.0164835164835165 | 0.013565891472868205 | 0.02240000000000001 | 0.017964071856287407 | 0.0 | 0.04240282685512375 | 0.007874015748031498 | 0.0253807106598985 | 0.0332640332640333 | 0.016611295681063103 | 0.0199637023593466 | 0.004819277108433742 | 0.023890784982935197 | 0.0109739368998628 | 0.004232804232804231 | 0.02176870748299321 | 0.0052029136316337115 | 0.004135649296939624 | 0.009560229445506694 | 0.0158730158730159 |
| 0.34 | 0.0384615384615385 | 0.00387596899224806 | 0.024 | 0.020958083832335286 | 0.0 | 0.017667844522968202 | 0.007874015748031498 | 0.06091370558375631 | 0.022869022869022912 | 0.018272425249169413 | 0.01633393829401091 | 0.004819277108433742 | 0.0341296928327645 | 0.020576131687242802 | 0.006349206349206352 | 0.02312925170068028 | 0.0010405827263267413 | 0.003308519437551701 | 0.013384321223709405 | 0.007936507936507941 |
| 0.3600000000000001 | 0.00549450549450549 | 0.00968992248062015 | 0.02240000000000001 | 0.022455089820359306 | 0.0 | 0.02826855123674908 | 0.0216535433070866 | 0.0456852791878173 | 0.022869022869022912 | 0.0149501661129568 | 0.014519056261342998 | 0.0 | 0.013651877133105804 | 0.0109739368998628 | 0.008465608465608473 | 0.010884353741496601 | 0.00416233090530697 | 0.0024813895781637717 | 0.0 | 0.007936507936507941 |
| 0.38000000000000012 | 0.00549450549450549 | 0.011627906976744195 | 0.016000000000000007 | 0.016467065868263506 | 0.0 | 0.02826855123674908 | 0.01968503937007871 | 0.0456852791878173 | 0.0311850311850312 | 0.019933554817275708 | 0.012704174228675109 | 0.0 | 0.013651877133105804 | 0.0137174211248285 | 0.00317460317460317 | 0.009523809523809523 | 0.0010405827263267413 | 0.0024813895781637717 | 0.009560229445506694 | 0.017460317460317506 |
| 0.4 | 0.021978021978022 | 0.017441860465116307 | 0.012800000000000004 | 0.013473053892215604 | 0.006993006993006992 | 0.0141342756183746 | 0.0177165354330709 | 0.0304568527918782 | 0.012474012474012499 | 0.008305647840531564 | 0.007259528130671513 | 0.0 | 0.023890784982935197 | 0.00823045267489712 | 0.00317460317460317 | 0.010884353741496601 | 0.003121748178980231 | 0.0008271298593879244 | 0.0 | 0.020634920634920607 |
### Chart: nmr-1 A1
| Category | nmr-1(ak4)_A1_01 | nmr-1(ak4)_A1_02 | nmr-1(ak4)_A1_03 | nmr-1(ak4)_A1_04 | nmr-1(ak4)_A1_05 | nmr-1(ak4)_A1_06 | nmr-1(ak4)_A1_07 | nmr-1(ak4)_A1_08 | nmr-1(ak4)_A1_09 | nmr-1(ak4)_A1_10 | nmr-1(ak4)_A1_11 | nmr-1(ak4)_A1_12 | nmr-1(ak4)_A1_13 | nmr-1(ak4)_A1_14 | nmr-1(ak4)_A1_15 | nmr-1(ak4)_A1_16 | nmr-1(ak4)_A1_17 | nmr-1(ak4)_A1_18 | nmr-1(ak4)_A1_20 |
|---|---|---|---|---|---|---|---|---|---|---|---|---|---|---|---|---|---|---|---|
| 0 | None | None | None | None | None | None | None | None | None | None | None | None | None | None | None | None | None | None | None |
| 2.0000000000000007E-2 | 0.059712773998488344 | 0.0666666666666667 | 0.060478199718706 | 0.06384742951907131 | 0.0665680473372781 | 0.07085916740478299 | 0.06441476826394343 | 0.06821963394342764 | 0.049242424242424226 | 0.05103806228373699 | 0.0718321226795803 | 0.06942148760330583 | 0.08554319931565443 | 0.07995365005793745 | 0.05922551252847379 | 0.0739299610894942 | 0.06451612903225813 | 0.07053941908713693 | 0.07107438016528929 |
| 4.0000000000000015E-2 | 0.10052910052910102 | 0.10406504065040703 | 0.0977496483825598 | 0.10033167495854105 | 0.0976331360946746 | 0.09123117803365813 | 0.0879811468970935 | 0.10981697171381004 | 0.118181818181818 | 0.106401384083045 | 0.09362389023405977 | 0.110743801652893 | 0.10179640718562906 | 0.12746234067207404 | 0.0956719817767654 | 0.0972762645914397 | 0.08654602675059016 | 0.12116182572614104 | 0.0735537190082645 |
| 6.0000000000000019E-2 | 0.12169312169312205 | 0.11869918699187003 | 0.11954992967651203 | 0.12023217247097806 | 0.111686390532544 | 0.110717449069973 | 0.111547525530244 | 0.11397670549084903 | 0.112878787878788 | 0.11937716262975796 | 0.12429378531073405 | 0.134710743801653 | 0.132591958939264 | 0.10428736964078797 | 0.11996962794229303 | 0.07587548638132302 | 0.118804091266719 | 0.12199170124481305 | 0.11157024793388404 |
| 8.0000000000000029E-2 | 0.13907785336356795 | 0.1447154471544721 | 0.1504922644163151 | 0.15671641791044807 | 0.13461538461538505 | 0.11868910540301203 | 0.1358994501178321 | 0.133943427620632 | 0.14090909090909107 | 0.143598615916955 | 0.138014527845036 | 0.16363636363636405 | 0.1471343028229261 | 0.12862108922363788 | 0.126803340926348 | 0.14007782101167293 | 0.135326514555468 | 0.152697095435685 | 0.12231404958677702 |
| 0.1 | 0.136054421768707 | 0.12276422764227606 | 0.14486638537271407 | 0.13432835820895495 | 0.12869822485207105 | 0.11514614703277203 | 0.125687352710134 | 0.11647254575707203 | 0.12045454545454502 | 0.13235294117647106 | 0.14850686037126706 | 0.1537190082644631 | 0.118049615055603 | 0.13441483198146012 | 0.14274867122247506 | 0.120622568093385 | 0.12903225806451593 | 0.13360995850622406 | 0.123140495867769 |
| 0.12000000000000002 | 0.0763416477702192 | 0.08292682926829277 | 0.0759493670886076 | 0.07545605306799341 | 0.08136094674556216 | 0.09123117803365813 | 0.07227022780832684 | 0.0815307820299501 | 0.0734848484848485 | 0.07871972318339103 | 0.06860371267150929 | 0.05123966942148759 | 0.0710008554319932 | 0.0602549246813441 | 0.0751708428246014 | 0.06420233463035023 | 0.07238394964594813 | 0.08049792531120334 | 0.07520661157024792 |
| 0.14000000000000001 | 0.0619803476946334 | 0.06910569105691064 | 0.06469760900140653 | 0.049751243781094495 | 0.07248520710059173 | 0.07085916740478299 | 0.06834249803613512 | 0.064891846921797 | 0.0659090909090909 | 0.0674740484429066 | 0.048426150121065395 | 0.059504132231405014 | 0.0633019674935843 | 0.040556199304750885 | 0.04935459377372823 | 0.0680933852140078 | 0.053501180173092085 | 0.07219917012448132 | 0.059504132231405014 |
| 0.16 | 0.0740740740740741 | 0.06178861788617892 | 0.0534458509142053 | 0.0588723051409619 | 0.0628698224852071 | 0.049601417183348116 | 0.06834249803613512 | 0.0640599001663894 | 0.0681818181818182 | 0.0666089965397924 | 0.0540758676351897 | 0.04793388429752072 | 0.05389221556886231 | 0.05909617612977982 | 0.0622627182991648 | 0.06614785992217899 | 0.06294256490952013 | 0.040663900414937816 | 0.068595041322314 |
| 0.18000000000000005 | 0.05668934240362808 | 0.05609756097560981 | 0.048523206751054884 | 0.05140961857379769 | 0.0414201183431953 | 0.0460584588131089 | 0.06441476826394343 | 0.05823627287853582 | 0.046969696969697 | 0.0467128027681661 | 0.05084745762711862 | 0.03884297520661161 | 0.04448246364414034 | 0.0451911935110081 | 0.056188306757782795 | 0.06420233463035023 | 0.06215578284815112 | 0.04149377593361002 | 0.06033057851239672 |
| 0.2 | 0.037037037037037014 | 0.031707317073170725 | 0.037974683544303806 | 0.0406301824212272 | 0.0443786982248521 | 0.05048715677590788 | 0.04556166535742342 | 0.032445923460898515 | 0.04318181818181819 | 0.03200692041522492 | 0.0387409200968523 | 0.02809917355371901 | 0.0316509837467921 | 0.0393974507531866 | 0.04252088078967348 | 0.0350194552529183 | 0.04878048780487813 | 0.03319502074688799 | 0.04793388429752072 |
| 0.22 | 0.02267573696145121 | 0.030894308943089407 | 0.019690576652602015 | 0.022388059701492494 | 0.0273668639053254 | 0.03631532329495131 | 0.025923016496465008 | 0.02662229617304491 | 0.0318181818181818 | 0.02249134948096892 | 0.030669895076674718 | 0.019008264462809902 | 0.02737382378100942 | 0.02317497103128619 | 0.0311313591495824 | 0.0330739299610895 | 0.029897718332022 | 0.029875518672199217 | 0.031404958677686015 |
| 0.24000000000000005 | 0.018140589569161016 | 0.016260162601626 | 0.0225035161744023 | 0.0190713101160862 | 0.01997041420118341 | 0.023029229406554507 | 0.022780832678711713 | 0.0207986688851913 | 0.0212121212121212 | 0.0198961937716263 | 0.025020177562550414 | 0.0173553719008264 | 0.013686911890504699 | 0.0208574739281576 | 0.024297646165527705 | 0.0155642023346304 | 0.013375295043273 | 0.012448132780083 | 0.031404958677686015 |
| 0.26 | 0.0158730158730159 | 0.016260162601626 | 0.015471167369901501 | 0.013266998341625204 | 0.0155325443786982 | 0.011514614703277203 | 0.01885310290652 | 0.0158069883527454 | 0.0143939393939394 | 0.02249134948096892 | 0.0121065375302663 | 0.016528925619834708 | 0.011120615911035105 | 0.011587485515643104 | 0.013667425968109305 | 0.017509727626459103 | 0.021243115656963022 | 0.0066390041493775915 | 0.022314049586776918 |
| 0.28000000000000008 | 0.00982615268329554 | 0.008943089430894315 | 0.014767932489451498 | 0.009950248756218917 | 0.0103550295857988 | 0.020372010628875118 | 0.00864100549882168 | 0.008319467554076549 | 0.0090909090909091 | 0.008650519031141874 | 0.010492332526230795 | 0.013223140495867806 | 0.011976047904191595 | 0.02201622247972192 | 0.008352315869400155 | 0.00972762645914397 | 0.011014948859165999 | 0.00746887966804979 | 0.0140495867768595 |
| 0.3000000000000001 | 0.00529100529100529 | 0.008943089430894315 | 0.005625879043600562 | 0.010779436152570498 | 0.009615384615384628 | 0.005314437555358718 | 0.006284367635506684 | 0.00499168053244592 | 0.0166666666666667 | 0.003460207612456752 | 0.008878127522195323 | 0.0066115702479338815 | 0.00513259195893926 | 0.0139049826187717 | 0.0129081245254366 | 0.0136186770428016 | 0.010228166797797 | 0.009128630705394188 | 0.011570247933884299 |
| 0.32000000000000012 | 0.006046863189720335 | 0.0040650406504065 | 0.0021097046413502112 | 0.00580431177446103 | 0.002218934911242602 | 0.009743135518157666 | 0.003142183817753342 | 0.00499168053244592 | 0.003787878787878792 | 0.007785467128027684 | 0.00484261501210654 | 0.0066115702479338815 | 0.004277159965782722 | 0.005793742757821552 | 0.00531511009870919 | 0.0136186770428016 | 0.009441384736428015 | 0.005809128630705392 | 0.00991735537190083 |
| 0.34 | 0.00151171579743008 | 0.0016260162601626005 | 0.00281293952180028 | 0.004975124378109448 | 0.0007396449704142015 | 0.01240035429583701 | 0.003927729772191672 | 0.003327787021630622 | 0.00681818181818182 | 0.005190311418685122 | 0.00726392251815981 | 0.0049586776859504135 | 0.0076988879384089 | 0.008111239860950168 | 0.008352315869400155 | 0.00972762645914397 | 0.005507474429583012 | 0.003319502074688799 | 0.0066115702479338815 |
| 0.3600000000000001 | 0.0007558578987150422 | 0.0016260162601626005 | 0.00281293952180028 | 0.004975124378109448 | 0.002218934911242602 | 0.0026572187776793617 | 0.002356637863315 | 0.0016638935108153105 | 0.003787878787878792 | 0.006055363321799312 | 0.00564971751412429 | 0.0008264462809917365 | 0.004277159965782722 | 0.005793742757821552 | 0.00607441154138193 | 0.0 | 0.006294256490952013 | 0.000829875518672199 | 0.0049586776859504135 |
| 0.38000000000000012 | 0.003023431594860172 | 0.0032520325203252 | 0.0021097046413502112 | 0.00414593698175788 | 0.0029585798816568008 | 0.004428697962798942 | 0.004713275726630012 | 0.0016638935108153105 | 0.0007575757575757582 | 0.003460207612456752 | 0.00484261501210654 | 0.0024793388429752107 | 0.00256629597946963 | 0.0023174971031286198 | 0.004555808656036448 | 0.00389105058365759 | 0.003933910306845001 | 0.000829875518672199 | 0.004132231404958682 |
| 0.4 | 0.00151171579743008 | 0.000813008130081301 | 0.00140646976090014 | 0.0 | 0.002218934911242602 | 0.004428697962798942 | 0.002356637863315 | 0.0008319467554076548 | 0.00303030303030303 | 0.002595155709342561 | 0.002421307506053271 | 0.0 | 0.0008554319931565447 | 0.006952491309385862 | 0.003037205770690961 | 0.0 | 0.0007867820613690012 | 0.0016597510373444 | 0.0033057851239669408 |
### Chart: nmr-1 A3
| Category | nmr-1(ak4)_A3_01 | nmr-1(ak4)_A3_02 | nmr-1(ak4)_A3_03 | nmr-1(ak4)_A3_04 | nmr-1(ak4)_A3_05 | nmr-1(ak4)_A3_06 | nmr-1(ak4)_A3_07 | nmr-1(ak4)_A3_08 | nmr-1(ak4)_A3_09 | nmr-1(ak4)_A3_10 | nmr-1(ak4)_A3_11 | nmr-1(ak4)_A3_12 | nmr-1(ak4)_A3_13 | nmr-1(ak4)_A3_14 | nmr-1(ak4)_A3_15 | nmr-1(ak4)_A3_16 | nmr-1(ak4)_A3_17 | nmr-1(ak4)_A3_18 | nmr-1(ak4)_A3_19 | nmr-1(ak4)_A3_20 |
|---|---|---|---|---|---|---|---|---|---|---|---|---|---|---|---|---|---|---|---|---|
| 0 | None | None | None | None | None | None | None | None | None | None | None | None | None | None | None | None | None | None | None | None |
| 2.0000000000000007E-2 | 0.06766917293233081 | 0.0641221374045802 | 0.05830903790087462 | 0.08800000000000004 | 0.057553956834532426 | 0.0756302521008403 | 0.0732177263969171 | 0.07286432160804023 | 0.0590614886731392 | 0.0626086956521739 | 0.06841046277666 | 0.053415061295972004 | 0.0878438331854481 | 0.0955631399317406 | 0.05450733752620552 | 0.0630252100840336 | 0.06889763779527562 | 0.0504908835904628 | 0.04593929450369163 | 0.08604651162790698 |
| 4.0000000000000015E-2 | 0.119548872180451 | 0.11068702290076297 | 0.0816326530612245 | 0.096 | 0.0781089414182939 | 0.102040816326531 | 0.0847784200385356 | 0.09422110552763824 | 0.0792880258899676 | 0.09130434782608704 | 0.09859154929577466 | 0.106830122591944 | 0.09582963620230699 | 0.08532423208191128 | 0.09853249475890997 | 0.1 | 0.07283464566929132 | 0.07924263674614314 | 0.10910582444626706 | 0.101162790697674 |
| 6.0000000000000019E-2 | 0.12481203007518803 | 0.126717557251908 | 0.0845481049562682 | 0.08800000000000004 | 0.11408016443987698 | 0.11404561824729903 | 0.10211946050096297 | 0.11055276381909497 | 0.140776699029126 | 0.12173913043478303 | 0.12206572769953103 | 0.117338003502627 | 0.102040816326531 | 0.09044368600682583 | 0.10901467505241105 | 0.141176470588235 | 0.08267716535433073 | 0.12131837307152903 | 0.12469237079573403 | 0.102325581395349 |
| 8.0000000000000029E-2 | 0.14360902255639107 | 0.142748091603053 | 0.141399416909621 | 0.048 | 0.117163412127441 | 0.14165666266506594 | 0.135838150289017 | 0.12688442211055295 | 0.11812297734627804 | 0.14695652173913 | 0.141515761234071 | 0.15761821366024506 | 0.14995563442768406 | 0.116040955631399 | 0.13207547169811296 | 0.16134453781512606 | 0.09350393700787403 | 0.143758765778401 | 0.15832649712879407 | 0.1 |
| 0.1 | 0.15789473684210512 | 0.158778625954198 | 0.116618075801749 | 0.08400000000000005 | 0.14388489208633107 | 0.11644657863145302 | 0.11849710982659002 | 0.12437185929648202 | 0.14644012944983806 | 0.126086956521739 | 0.16498993963782707 | 0.11821366024518404 | 0.12599822537710706 | 0.07849829351535843 | 0.0922431865828092 | 0.15042016806722708 | 0.12007874015748003 | 0.16058906030855488 | 0.13617719442165693 | 0.124418604651163 |
| 0.12000000000000002 | 0.054887218045112825 | 0.05954198473282441 | 0.059766763848396555 | 0.032000000000000015 | 0.061664953751284696 | 0.05522208883553422 | 0.056840077071290886 | 0.06532663316582923 | 0.0558252427184466 | 0.0565217391304348 | 0.05700871898055 | 0.0665499124343257 | 0.05767524401064771 | 0.07849829351535843 | 0.06079664570230612 | 0.0571428571428571 | 0.0501968503937008 | 0.06241234221598882 | 0.0484003281378179 | 0.0686046511627907 |
| 0.14000000000000001 | 0.04511278195488724 | 0.049618320610687 | 0.026239067055393618 | 0.040000000000000015 | 0.0626927029804728 | 0.0528211284513806 | 0.0481695568400771 | 0.052763819095477414 | 0.05097087378640778 | 0.0504347826086957 | 0.04560697518444 | 0.03765323992994751 | 0.055013309671694786 | 0.052901023890785014 | 0.0440251572327044 | 0.05630252100840343 | 0.0590551181102362 | 0.046283309957924304 | 0.06316652994257593 | 0.0430232558139535 |
| 0.16 | 0.0541353383458647 | 0.0473282442748092 | 0.04081632653061222 | 0.06800000000000002 | 0.059609455292908495 | 0.039615846338535404 | 0.0529865125240848 | 0.04899497487437192 | 0.05744336569579288 | 0.040000000000000015 | 0.048960429242119415 | 0.05779334500875662 | 0.0496894409937888 | 0.0341296928327645 | 0.058700209643605915 | 0.05462184873949578 | 0.06299212598425201 | 0.053997194950911646 | 0.0574241181296144 | 0.0395348837209302 |
| 0.18000000000000005 | 0.04511278195488724 | 0.03969465648854961 | 0.051020408163265286 | 0.016000000000000007 | 0.032887975334018514 | 0.0384153661464586 | 0.0481695568400771 | 0.04773869346733674 | 0.0501618122977346 | 0.0426086956521739 | 0.03688799463447352 | 0.027145359019264414 | 0.0354924578527063 | 0.04266211604095562 | 0.04612159329140457 | 0.0310924369747899 | 0.04822834645669287 | 0.052594670406732116 | 0.041837571780147714 | 0.03139534883720931 |
| 0.2 | 0.030075187969924817 | 0.04503816793893133 | 0.0393586005830904 | 0.024 | 0.032887975334018514 | 0.03241296518607442 | 0.0366088631984586 | 0.03266331658291463 | 0.034789644012945 | 0.030434782608695705 | 0.0355466130114017 | 0.031523642732049 | 0.0354924578527063 | 0.029010238907849806 | 0.04612159329140457 | 0.03445378151260501 | 0.036417322834645716 | 0.0427769985974755 | 0.0319934372436423 | 0.02906976744186051 |
| 0.22 | 0.030075187969924817 | 0.0229007633587786 | 0.0393586005830904 | 0.032000000000000015 | 0.02980472764645431 | 0.0288115246098439 | 0.03564547206165702 | 0.03517587939698491 | 0.029126213592233 | 0.020869565217391292 | 0.029510395707578806 | 0.0341506129597198 | 0.029281277728482713 | 0.030716723549488085 | 0.02515723270440251 | 0.0352941176470588 | 0.037401574803149616 | 0.02664796633941091 | 0.0360951599671862 | 0.0302325581395349 |
| 0.24000000000000005 | 0.0240601503759399 | 0.0145038167938931 | 0.021865889212828 | 0.020000000000000007 | 0.0205549845837616 | 0.025210084033613397 | 0.0366088631984586 | 0.03517587939698491 | 0.027508090614886706 | 0.026086956521739115 | 0.02213279678068411 | 0.0253940455341506 | 0.0239574090505768 | 0.030716723549488085 | 0.02515723270440251 | 0.015126050420168104 | 0.047244094488189 | 0.0217391304347826 | 0.022149302707137008 | 0.024418604651162797 |
| 0.26 | 0.0112781954887218 | 0.011450381679389304 | 0.026239067055393618 | 0.044 | 0.016443987667009306 | 0.0132052821128451 | 0.017341040462427702 | 0.0251256281407035 | 0.0323624595469256 | 0.0278260869565217 | 0.018108651911468803 | 0.024518388791593692 | 0.0150842945874002 | 0.03242320819112631 | 0.020964360587002108 | 0.010924369747899204 | 0.037401574803149616 | 0.022440392706872418 | 0.012305168170631698 | 0.0255813953488372 |
| 0.28000000000000008 | 0.012030075187969901 | 0.00458015267175573 | 0.026239067055393618 | 0.028 | 0.0205549845837616 | 0.015606242496998799 | 0.026011560693641602 | 0.0125628140703518 | 0.019417475728155307 | 0.012173913043478299 | 0.010060362173038198 | 0.014886164623467606 | 0.0150842945874002 | 0.0221843003412969 | 0.020964360587002108 | 0.013445378151260505 | 0.0334645669291339 | 0.016129032258064505 | 0.008203445447087785 | 0.0255813953488372 |
| 0.3000000000000001 | 0.0105263157894737 | 0.0106870229007634 | 0.030612244897959207 | 0.028 | 0.0102774922918808 | 0.0120048019207683 | 0.013487475915221604 | 0.02135678391959799 | 0.0121359223300971 | 0.0217391304347826 | 0.007377598926894702 | 0.011383537653239904 | 0.0106477373558119 | 0.017064846416382305 | 0.023060796645702285 | 0.011764705882352905 | 0.01968503937007871 | 0.011921458625525906 | 0.007383100902379 | 0.0162790697674419 |
| 0.32000000000000012 | 0.006766917293233082 | 0.006870229007633594 | 0.0233236151603499 | 0.028 | 0.012332990750256895 | 0.0120048019207683 | 0.01252408477842 | 0.008793969849246235 | 0.008090614886731394 | 0.01304347826086961 | 0.008048289738430577 | 0.006129597197898422 | 0.013309671694764907 | 0.005119453924914683 | 0.016771488469601706 | 0.006722689075630252 | 0.0127952755905512 | 0.006311360448807852 | 0.009023789991796564 | 0.017441860465116307 |
| 0.34 | 0.003759398496240601 | 0.006106870229007632 | 0.013119533527696795 | 0.020000000000000007 | 0.0102774922918808 | 0.0120048019207683 | 0.010597302504817 | 0.005025125628140698 | 0.0105177993527508 | 0.012173913043478299 | 0.004024144869215286 | 0.007005253940455343 | 0.007985803016858924 | 0.006825938566552898 | 0.016771488469601706 | 0.0008403361344537821 | 0.015748031496063006 | 0.004908835904628332 | 0.007383100902379 | 0.015116279069767405 |
| 0.3600000000000001 | 0.003759398496240601 | 0.00381679389312977 | 0.0058309037900874635 | 0.012 | 0.0102774922918808 | 0.0036014405762304917 | 0.00867052023121387 | 0.005025125628140698 | 0.004854368932038832 | 0.012173913043478299 | 0.0013413816230717608 | 0.004378283712784594 | 0.007098491570541262 | 0.003412969283276451 | 0.006289308176100634 | 0.003361344537815131 | 0.005905511811023622 | 0.0035063113604488112 | 0.0041017227235438936 | 0.008139534883720937 |
| 0.38000000000000012 | 0.002255639097744361 | 0.00534351145038168 | 0.0058309037900874635 | 0.012 | 0.005138746145940392 | 0.00600240096038415 | 0.0067437379576107915 | 0.00251256281407035 | 0.00323624595469256 | 0.00347826086956522 | 0.0026827632461435312 | 0.0026269702276707518 | 0.0053238686779059395 | 0.0187713310580205 | 0.006289308176100634 | 0.003361344537815131 | 0.00393700787401575 | 0.00140252454417952 | 0.002461033634126331 | 0.006976744186046513 |
| 0.4 | 0.003759398496240601 | 0.00381679389312977 | 0.007288629737609335 | 0.016000000000000007 | 0.0092497430626927 | 0.00600240096038415 | 0.0019267822736030809 | 0.00125628140703518 | 0.0024271844660194216 | 0.0052173913043478334 | 0.002012072434607648 | 0.0008756567425569185 | 0.000887311446317658 | 0.006825938566552898 | 0.00419287211740042 | 0.0008403361344537821 | 0.005905511811023622 | 0.0035063113604488112 | 0.002461033634126331 | 0.00348837209302326 |
### Chart: nmr-1 A5
| Category | nmr-1(ak4)_A5_01 | nmr-1(ak4)_A5_02 | nmr-1(ak4)_A5_03 | nmr-1(ak4)_A5_04 | nmr-1(ak4)_A5_05 | nmr-1(ak4)_A5_06 | nmr-1(ak4)_A5_07 | nmr-1(ak4)_A5_08 | nmr-1(ak4)_A5_09 | nmr-1(ak4)_A5_10 | | nmr-1(ak4)_A5_12 | nmr-1(ak4)_A5_13 | nmr-1(ak4)_A5_14 | nmr-1(ak4)_A5_15 | nmr-1(ak4)_A5_16 | nmr-1(ak4)_A5_17 | nmr-1(ak4)_A5_18 | nmr-1(ak4)_A5_19 | nmr-1(ak4)_A5_20 |
|---|---|---|---|---|---|---|---|---|---|---|---|---|---|---|---|---|---|---|---|---|
| 0 | None | None | None | None | None | None | None | None | None | None | None | None | None | None | None | None | None | None | None | None |
| 2.0000000000000007E-2 | 0.05369127516778518 | 0.06735751295336792 | 0.06791907514450873 | 0.0674567000911577 | 0.111111111111111 | 0.059050064184852404 | 0.05070993914807303 | 0.0722733245729304 | 0.0571428571428571 | 0.0827178729689808 | 0.07978723404255325 | 0.07031888798037612 | 0.0486656200941915 | 0.07183098591549303 | 0.06989247311827965 | 0.09954058192955588 | 0.07142857142857141 | 0.064343163538874 | 0.0867992766726944 | 0.0839416058394161 |
| 4.0000000000000015E-2 | 0.08456375838926185 | 0.113989637305699 | 0.10549132947976902 | 0.08386508659981776 | 0.05555555555555558 | 0.09756097560975616 | 0.07505070993914813 | 0.07095926412614982 | 0.0964285714285714 | 0.10192023633678003 | 0.09468085106382984 | 0.0727718724448078 | 0.08006279434850858 | 0.08169014084507045 | 0.0913978494623656 | 0.0949464012251149 | 0.09309791332263247 | 0.05093833780160863 | 0.11573236889692598 | 0.05839416058394161 |
| 6.0000000000000019E-2 | 0.131543624161074 | 0.08031088082901548 | 0.08959537572254338 | 0.12215132178669105 | 0.06944444444444443 | 0.10397946084724 | 0.0588235294117647 | 0.08672798948751642 | 0.0785714285714286 | 0.11669128508124109 | 0.108510638297872 | 0.11529026982829105 | 0.09890109890109895 | 0.0647887323943662 | 0.10322580645161308 | 0.11485451761102597 | 0.09951845906902096 | 0.05093833780160863 | 0.09403254972875236 | 0.07299270072992703 |
| 8.0000000000000029E-2 | 0.12885906040268497 | 0.0569948186528497 | 0.11849710982659002 | 0.16499544211485906 | 0.06944444444444443 | 0.13222079589216906 | 0.04868154158215007 | 0.12483574244415209 | 0.11785714285714298 | 0.10192023633678003 | 0.143617021276596 | 0.13409648405560107 | 0.0926216640502355 | 0.0661971830985915 | 0.121505376344086 | 0.08422664624808583 | 0.1380417335473521 | 0.04289544235924932 | 0.09764918625678118 | 0.08576642335766425 |
| 0.1 | 0.102013422818792 | 0.062176165803108814 | 0.09971098265895953 | 0.13400182315405695 | 0.06944444444444443 | 0.102695763799743 | 0.0466531440162272 | 0.08672798948751642 | 0.0642857142857143 | 0.10487444608567204 | 0.13404255319148906 | 0.13409648405560107 | 0.0816326530612245 | 0.09154929577464796 | 0.11827956989247297 | 0.0949464012251149 | 0.158908507223114 | 0.0348525469168901 | 0.09041591320072327 | 0.08941605839416064 |
| 0.12000000000000002 | 0.05906040268456381 | 0.07512953367875648 | 0.04624277456647398 | 0.04922515952597987 | 0.05555555555555558 | 0.05648267008985882 | 0.0243407707910751 | 0.06964520367936917 | 0.0517857142857143 | 0.062038404726735635 | 0.045744680851063826 | 0.04497138184791502 | 0.0518053375196232 | 0.05774647887323942 | 0.0526881720430108 | 0.05513016845329249 | 0.056982343499197396 | 0.026809651474530807 | 0.07775768535262213 | 0.05109489051094891 |
| 0.14000000000000001 | 0.04295302013422823 | 0.0336787564766839 | 0.04624277456647398 | 0.06016408386508662 | 0.0416666666666667 | 0.059050064184852404 | 0.03448275862068972 | 0.038107752956636 | 0.03928571428571431 | 0.047267355982274696 | 0.0563829787234043 | 0.055600981193785814 | 0.03767660910518052 | 0.05774647887323942 | 0.05591397849462372 | 0.05053598774885151 | 0.03932584269662921 | 0.02949061662198391 | 0.05063291139240512 | 0.04014598540145987 |
| 0.16 | 0.0442953020134228 | 0.0414507772020725 | 0.0419075144508671 | 0.05287146763901551 | 0.02777777777777782 | 0.033376123234916594 | 0.022312373225152105 | 0.0459921156373193 | 0.037500000000000006 | 0.03249630723781392 | 0.0340425531914894 | 0.053965658217497985 | 0.04238618524332812 | 0.03943661971830991 | 0.050537634408602226 | 0.04441041347626342 | 0.0529695024077047 | 0.032171581769437 | 0.0415913200723327 | 0.018248175182481806 |
| 0.18000000000000005 | 0.03624161073825501 | 0.04922279792746113 | 0.04335260115606942 | 0.04375569735642665 | 0.06944444444444443 | 0.03594351732991012 | 0.030425963488843816 | 0.04467805519053879 | 0.037500000000000006 | 0.035450516986706114 | 0.030851063829787202 | 0.04415372035977112 | 0.02511773940345371 | 0.0323943661971831 | 0.0419354838709677 | 0.0459418070444104 | 0.051364365971107495 | 0.04825737265415548 | 0.03978300180831831 | 0.021897810218978117 |
| 0.2 | 0.03758389261744973 | 0.0336787564766839 | 0.04479768786127175 | 0.0391978122151322 | 0.111111111111111 | 0.0320924261874198 | 0.03651115618661261 | 0.0341655716162943 | 0.0482142857142857 | 0.02806499261447561 | 0.031914893617021316 | 0.03515944399018808 | 0.021978021978022 | 0.03661971830985922 | 0.046236559139784895 | 0.030627871362940307 | 0.0377207062600321 | 0.0455764075067024 | 0.032549728752260414 | 0.05109489051094891 |
| 0.22 | 0.03221476510067112 | 0.03886010362694301 | 0.0346820809248555 | 0.028258887876025506 | 0.08333333333333333 | 0.02310654685494219 | 0.04259634888438132 | 0.03547963206307493 | 0.0321428571428571 | 0.02954209748892172 | 0.0223404255319149 | 0.03761242845461982 | 0.0361067503924647 | 0.04366197183098591 | 0.03225806451612901 | 0.0275650842266462 | 0.0377207062600321 | 0.07238605898123333 | 0.05063291139240512 | 0.03467153284671532 |
| 0.24000000000000005 | 0.03624161073825501 | 0.046632124352331626 | 0.027456647398843913 | 0.019143117593436603 | 0.06944444444444443 | 0.024390243902439 | 0.0608519269776876 | 0.02890932982917211 | 0.0482142857142857 | 0.0206794682422452 | 0.027659574468085115 | 0.035977105478332015 | 0.0361067503924647 | 0.0408450704225352 | 0.043010752688171984 | 0.0214395099540582 | 0.04093097913322633 | 0.05898123324396781 | 0.05063291139240512 | 0.04014598540145987 |
| 0.26 | 0.0228187919463087 | 0.04922279792746113 | 0.021676300578034716 | 0.015496809480401101 | 0.013888888888888907 | 0.0205391527599487 | 0.07302231237322522 | 0.03285151116951381 | 0.037500000000000006 | 0.014771048744460901 | 0.0170212765957447 | 0.026982829108748992 | 0.02982731554160131 | 0.03661971830985922 | 0.02365591397849461 | 0.026033690658499215 | 0.013643659711075408 | 0.05093833780160863 | 0.0108499095840868 | 0.04197080291970798 |
| 0.28000000000000008 | 0.013422818791946301 | 0.0414507772020725 | 0.027456647398843913 | 0.00911577028258888 | 0.0 | 0.01668806161745831 | 0.077079107505071 | 0.0236530880420499 | 0.04107142857142858 | 0.025110782865583492 | 0.013829787234042604 | 0.013900245298446405 | 0.0486656200941915 | 0.0323943661971831 | 0.02258064516129031 | 0.01684532924961722 | 0.007223113964687 | 0.0670241286863271 | 0.0180831826401447 | 0.027372262773722615 |
| 0.3000000000000001 | 0.0161073825503356 | 0.0207253886010363 | 0.018786127167630107 | 0.0100273473108478 | 0.02777777777777782 | 0.024390243902439 | 0.07099391480730224 | 0.027595269382391607 | 0.0303571428571429 | 0.013293943870014794 | 0.00957446808510638 | 0.008176614881439085 | 0.0313971742543171 | 0.0408450704225352 | 0.0129032258064516 | 0.0336906584992343 | 0.0112359550561798 | 0.021447721179624717 | 0.0198915009041591 | 0.03467153284671532 |
| 0.32000000000000012 | 0.013422818791946301 | 0.03626943005181352 | 0.021676300578034716 | 0.0072926162260711 | 0.0 | 0.00641848523748395 | 0.052738336713995915 | 0.022339027595269418 | 0.028571428571428602 | 0.0221565731166913 | 0.011702127659574506 | 0.017988552739166008 | 0.0361067503924647 | 0.0380281690140845 | 0.011827956989247301 | 0.01684532924961722 | 0.008025682182985553 | 0.021447721179624717 | 0.007233273056057874 | 0.03284671532846722 |
| 0.34 | 0.0147651006711409 | 0.0207253886010363 | 0.018786127167630107 | 0.006381039197812224 | 0.013888888888888907 | 0.012836970474967901 | 0.0385395537525355 | 0.015768725361366608 | 0.025 | 0.014771048744460901 | 0.0074468085106383025 | 0.005723630417007362 | 0.02511773940345371 | 0.03521126760563382 | 0.011827956989247301 | 0.010719754977029098 | 0.009630818619582664 | 0.026809651474530807 | 0.007233273056057874 | 0.0237226277372263 |
| 0.3600000000000001 | 0.0120805369127517 | 0.012953367875647699 | 0.005780346820809252 | 0.006381039197812224 | 0.0 | 0.015404364569961504 | 0.0243407707910751 | 0.014454664914586099 | 0.021428571428571408 | 0.013293943870014794 | 0.010638297872340394 | 0.004088307440719542 | 0.021978021978022 | 0.014084507042253504 | 0.004301075268817198 | 0.0153139356814701 | 0.004012841091492782 | 0.021447721179624717 | 0.0018083182640144704 | 0.014598540145985401 |
| 0.38000000000000012 | 0.013422818791946301 | 0.015544041450777204 | 0.011560693641618505 | 0.000911577028258888 | 0.0 | 0.012836970474967901 | 0.014198782961460396 | 0.007884362680683314 | 0.008928571428571423 | 0.00738552437223043 | 0.00531914893617021 | 0.0016353229762878208 | 0.0125588697017268 | 0.022535211267605614 | 0.004301075268817198 | 0.00459418070444104 | 0.0064205457463884395 | 0.024128686327077688 | 0.0180831826401447 | 0.02554744525547451 |
| 0.4 | 0.00536912751677852 | 0.015544041450777204 | 0.005780346820809252 | 0.0027347310847766616 | 0.013888888888888907 | 0.0115532734274711 | 0.010141987829614599 | 0.005256241787122214 | 0.008928571428571423 | 0.011816838995568707 | 0.006382978723404264 | 0.00245298446443173 | 0.010989010989011 | 0.011267605633802807 | 0.006451612903225813 | 0.010719754977029098 | 0.002407704654895671 | 0.016085790884718506 | 0.0036166365280289312 | 0.010948905109489104 |

## Slide 3
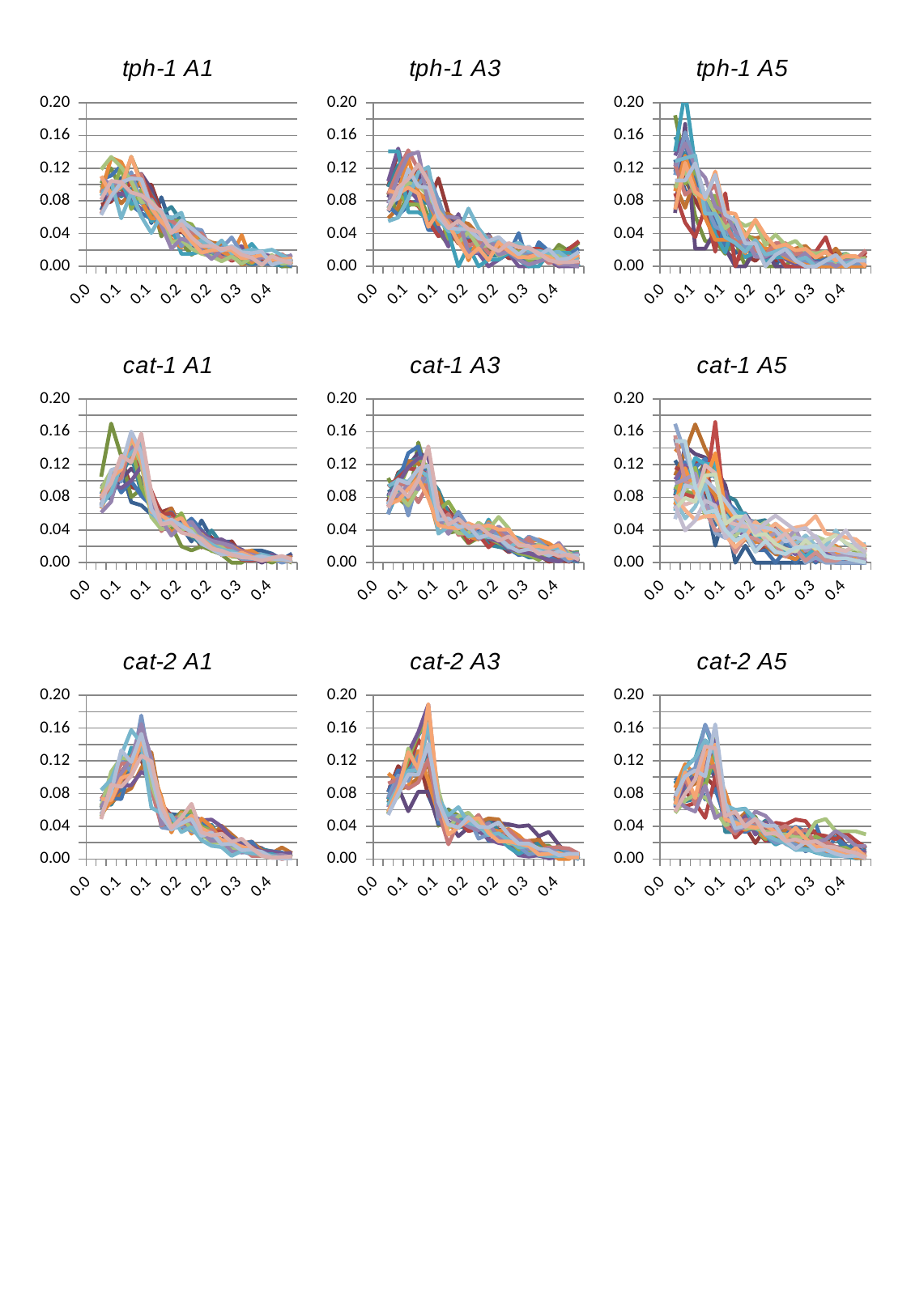

### Chart: tph-1 A1
| Category | thp-1(mg280)_A1_01 | thp-1(mg280)_A1_02 | thp-1(mg280)_A1_03 | thp-1(mg280)_A1_04 | thp-1(mg280)_A1_05 | thp-1(mg280)_A1_06 | thp-1(mg280)_A1_07 | thp-1(mg280)_A1_08 | thp-1(mg280)_A1_09 | thp-1(mg280)_A1_10 | tph-1(mg280)_A1_11 | tph-1(mg280)_A1_12 | tph-1(mg280)_A1_13 | tph-1(mg280)_A1_14 | tph-1(mg280)_A1_15 | tph-1(mg280)_A1_16 | tph-1(mg280)_A1_17 | tph-1(mg280)_A1_18 | tph-1(mg280)_A1_19 | tph-1(mg280)_A1_20 |
|---|---|---|---|---|---|---|---|---|---|---|---|---|---|---|---|---|---|---|---|---|
| 0 | None | None | None | None | None | None | None | None | None | None | None | None | None | None | None | None | None | None | None | None |
| 2.0000000000000007E-2 | 0.0686015831134565 | 0.07532956685499063 | 0.10141987829614597 | 0.08263069139966274 | 0.09122807017543866 | 0.09979209979209987 | 0.10602409638554203 | 0.09151785714285715 | 0.09926470588235292 | 0.09429280397022334 | 0.0945121951219512 | 0.08928571428571425 | 0.09292035398230093 | 0.0643712574850299 | 0.11827956989247297 | 0.10733452593917703 | 0.08577878103837466 | 0.110181311018131 | 0.06273764258555131 | 0.08141592920353978 |
| 4.0000000000000015E-2 | 0.09498680738786287 | 0.07909604519774013 | 0.11967545638945203 | 0.08600337268128164 | 0.09941520467836258 | 0.0914760914760915 | 0.11084337349397598 | 0.09151785714285715 | 0.08823529411764716 | 0.0818858560794045 | 0.100609756097561 | 0.13265306122449 | 0.08628318584070793 | 0.08832335329341323 | 0.133640552995392 | 0.10017889087656498 | 0.10383747178329603 | 0.08786610878661094 | 0.08555133079847921 | 0.10442477876106206 |
| 6.0000000000000019E-2 | 0.10817941952506598 | 0.10169491525423703 | 0.09330628803245443 | 0.08600337268128164 | 0.08538011695906428 | 0.07692307692307691 | 0.12289156626506002 | 0.08816964285714296 | 0.12132352941176502 | 0.0893300248138958 | 0.10365853658536597 | 0.12755102040816294 | 0.0973451327433628 | 0.10329341317365304 | 0.121351766513057 | 0.10375670840787103 | 0.05869074492099323 | 0.09762900976290102 | 0.10266159695817505 | 0.10265486725663703 |
| 8.0000000000000029E-2 | 0.10290237467018502 | 0.105461393596987 | 0.133874239350913 | 0.10623946037099502 | 0.09473684210526329 | 0.08939708939708947 | 0.0746987951807229 | 0.10267857142857104 | 0.06985294117647063 | 0.0818858560794045 | 0.076219512195122 | 0.102040816326531 | 0.11504424778761109 | 0.109281437125749 | 0.10445468509984597 | 0.08765652951699464 | 0.090293453724605 | 0.133891213389121 | 0.10741444866920202 | 0.09026548672566373 |
| 0.1 | 0.09762532981530346 | 0.09981167608286254 | 0.10344827586206902 | 0.11298482293423302 | 0.105263157894737 | 0.10395010395010403 | 0.0650602409638554 | 0.11049107142857104 | 0.09375000000000004 | 0.0694789081885856 | 0.07926829268292683 | 0.07908163265306124 | 0.0774336283185841 | 0.112275449101796 | 0.0783410138248848 | 0.08765652951699464 | 0.0609480812641084 | 0.10041841004184097 | 0.10646387832699603 | 0.08672566371681424 |
| 0.12000000000000002 | 0.0527704485488127 | 0.09981167608286254 | 0.08316430020283985 | 0.0944350758853288 | 0.087719298245614 | 0.06237006237006239 | 0.0578313253012048 | 0.08593750000000003 | 0.06617647058823531 | 0.07196029776674942 | 0.05487804878048778 | 0.058673469387755084 | 0.08185840707964603 | 0.07185628742514973 | 0.07987711213517663 | 0.0715563506261181 | 0.040632054176072185 | 0.0711297071129707 | 0.07224334600760457 | 0.07787610619469032 |
| 0.14000000000000001 | 0.08443271767810023 | 0.06591337099811682 | 0.03651115618661261 | 0.059021922428330514 | 0.06432748538011702 | 0.043659043659043696 | 0.0554216867469879 | 0.0658482142857143 | 0.0588235294117647 | 0.05459057071960302 | 0.0670731707317073 | 0.058673469387755084 | 0.0619469026548673 | 0.062874251497006 | 0.06298003072196623 | 0.0500894454382827 | 0.0632054176072235 | 0.05439330543933052 | 0.0684410646387833 | 0.06548672566371681 |
| 0.16 | 0.04749340369393144 | 0.0470809792843691 | 0.05476673427991894 | 0.05564924114671162 | 0.07251461988304093 | 0.03742203742203742 | 0.04337349397590363 | 0.0513392857142857 | 0.055147058823529396 | 0.0397022332506203 | 0.04878048780487813 | 0.0331632653061225 | 0.059734513274336334 | 0.05538922155688623 | 0.0291858678955453 | 0.02146690518783541 | 0.05869074492099323 | 0.041841004184100396 | 0.040874524714828914 | 0.0407079646017699 |
| 0.18000000000000005 | 0.0448548812664908 | 0.041431261770244796 | 0.0283975659229209 | 0.05733558178752112 | 0.054970760233918115 | 0.047817047817047834 | 0.02650602409638549 | 0.0513392857142857 | 0.055147058823529396 | 0.0496277915632754 | 0.015243902439024399 | 0.02295918367346941 | 0.0243362831858407 | 0.04790419161676651 | 0.03225806451612901 | 0.0339892665474061 | 0.06546275395033865 | 0.04463040446304042 | 0.0560836501901141 | 0.05132743362831862 |
| 0.2 | 0.023746701846965694 | 0.0470809792843691 | 0.014198782961460396 | 0.04721753794266443 | 0.035087719298245605 | 0.0353430353430353 | 0.02650602409638549 | 0.027901785714285705 | 0.05147058823529408 | 0.02729528535980151 | 0.015243902439024399 | 0.0433673469387755 | 0.0464601769911504 | 0.04041916167664671 | 0.021505376344086 | 0.028622540250447193 | 0.027088036117381507 | 0.030683403068340314 | 0.04657794676806076 | 0.0353982300884956 |
| 0.22 | 0.0263852242744063 | 0.03201506591337101 | 0.022312373225152105 | 0.0185497470489039 | 0.0233918128654971 | 0.0353430353430353 | 0.0289156626506024 | 0.024553571428571407 | 0.0330882352941176 | 0.02481389578163771 | 0.0182926829268293 | 0.025510204081632692 | 0.044247787610619496 | 0.04041916167664671 | 0.0153609831029186 | 0.0196779964221825 | 0.03160270880361171 | 0.0167364016736402 | 0.0361216730038023 | 0.024778761061946892 |
| 0.24000000000000005 | 0.013192612137203198 | 0.02448210922787191 | 0.014198782961460396 | 0.0185497470489039 | 0.0116959064327485 | 0.0291060291060291 | 0.016867469879518107 | 0.021205357142857106 | 0.0183823529411765 | 0.02481389578163771 | 0.0182926829268293 | 0.017857142857142898 | 0.0176991150442478 | 0.022455089820359306 | 0.012288786482334899 | 0.008944543828264765 | 0.020316027088036107 | 0.019525801952580208 | 0.0275665399239544 | 0.024778761061946892 |
| 0.26 | 0.013192612137203198 | 0.0225988700564972 | 0.014198782961460396 | 0.0185497470489039 | 0.0233918128654971 | 0.027027027027027015 | 0.016867469879518107 | 0.016741071428571407 | 0.0183823529411765 | 0.0148883374689826 | 0.009146341463414627 | 0.017857142857142898 | 0.02212389380530971 | 0.0104790419161677 | 0.00614439324116743 | 0.0196779964221825 | 0.03160270880361171 | 0.020920502092050198 | 0.019011406844106505 | 0.021238938053097314 |
| 0.28000000000000008 | 0.010554089709762505 | 0.009416195856873824 | 0.010141987829614599 | 0.0168634064080944 | 0.0105263157894737 | 0.014553014553014595 | 0.016867469879518107 | 0.00669642857142857 | 0.011029411764705904 | 0.01985111662531021 | 0.015243902439024399 | 0.0127551020408163 | 0.0353982300884956 | 0.008982035928143717 | 0.012288786482334899 | 0.0196779964221825 | 0.0158013544018059 | 0.019525801952580208 | 0.024714828897338385 | 0.0230088495575221 |
| 0.3000000000000001 | 0.0052770448548812715 | 0.013182674199623405 | 0.006085192697768763 | 0.015177065767285 | 0.016374269005848 | 0.00207900207900208 | 0.0072289156626506 | 0.012276785714285704 | 0.009191176470588244 | 0.02481389578163771 | 0.015243902439024399 | 0.038265306122449015 | 0.015486725663716809 | 0.017964071856287407 | 0.00307219662058372 | 0.014311270125223598 | 0.018058690744921 | 0.0097629009762901 | 0.017110266159695794 | 0.014159292035398192 |
| 0.32000000000000012 | 0.013192612137203198 | 0.018832391713747603 | 0.0020283975659229226 | 0.005059021922428332 | 0.00935672514619883 | 0.00623700623700624 | 0.00240963855421687 | 0.012276785714285704 | 0.003676470588235291 | 0.0074441687344913255 | 0.0274390243902439 | 0.0051020408163265285 | 0.015486725663716809 | 0.007485029940119763 | 0.010752688172042998 | 0.008944543828264765 | 0.020316027088036107 | 0.011157601115760104 | 0.0161596958174905 | 0.010619469026548698 |
| 0.34 | 0.002638522427440631 | 0.00564971751412429 | 0.006085192697768763 | 0.011804384485666107 | 0.0105263157894737 | 0.008316008316008329 | 0.004819277108433742 | 0.008928571428571423 | 0.014705882352941199 | 0.0148883374689826 | 0.0121951219512195 | 0.0127551020408163 | 0.0022123893805309713 | 0.011976047904191595 | 0.0015360983102918604 | 0.010733452593917704 | 0.018058690744921 | 0.013947001394700108 | 0.0180608365019011 | 0.0017699115044247805 |
| 0.3600000000000001 | 0.0052770448548812715 | 0.009416195856873824 | 0.008113590263691683 | 0.011804384485666107 | 0.0023391812865497107 | 0.00623700623700624 | 0.00240963855421687 | 0.008928571428571423 | 0.009191176470588244 | 0.0124069478908189 | 0.009146341463414627 | 0.00255102040816327 | 0.008849557522123897 | 0.0104790419161677 | 0.013824884792626705 | 0.010733452593917704 | 0.020316027088036107 | 0.008368200836820085 | 0.0019011406844106505 | 0.012389380530973498 |
| 0.38000000000000012 | 0.0 | 0.00564971751412429 | 0.0 | 0.008431703204047224 | 0.00116959064327485 | 0.004158004158004162 | 0.004819277108433742 | 0.005580357142857142 | 0.009191176470588244 | 0.0074441687344913255 | 0.0121951219512195 | 0.010204081632653106 | 0.00442477876106195 | 0.0149700598802395 | 0.00307219662058372 | 0.00715563506261181 | 0.0135440180586907 | 0.008368200836820085 | 0.005703422053231942 | 0.007079646017699114 |
| 0.4 | 0.002638522427440631 | 0.00564971751412429 | 0.0 | 0.0016863406408094404 | 0.008187134502923988 | 0.00623700623700624 | 0.0 | 0.00669642857142857 | 0.003676470588235291 | 0.0074441687344913255 | 0.0121951219512195 | 0.00255102040816327 | 0.0022123893805309713 | 0.005988023952095812 | 0.00460829493087558 | 0.014311270125223598 | 0.006772009029345373 | 0.0097629009762901 | 0.006653992395437262 | 0.003539823008849561 |
### Chart: tph-1 A3
| Category | tph-1(mg280)_A3_01 | tph-1(mg280)_A3_02 | tph-1(mg280)_A3_03 | tph-1(mg280)_A3_04 | tph-1(mg280)_A3_05 | tph-1(mg280)_A3_06 | tph-1(mg280)_A3_07 | tph-1(mg280)_A3_08 | tph-1(mg280)_A3_09 | tph-1(mg280)_A3_10 | tph-1(mg280)_A3_11 | tph-1(mg280)_A3_12 | tph-1(mg280)_A3_13 | tph-1(mg280)_A3_14 | tph-1(mg280)_A3_15 | tph-1(mg280)_A3_16 | tph-1(mg280)_A3_17 | tph-1(mg280)_A3_18 | tph-1(mg280)_A3_19 | tph-1(mg280)_A3_20 |
|---|---|---|---|---|---|---|---|---|---|---|---|---|---|---|---|---|---|---|---|---|
| 0 | None | None | None | None | None | None | None | None | None | None | None | None | None | None | None | None | None | None | None | None |
| 2.0000000000000007E-2 | 0.0669642857142857 | 0.09750000000000003 | 0.08970976253298153 | 0.08401639344262297 | 0.096989966555184 | 0.058563535911602224 | 0.0738007380073801 | 0.08895705521472394 | 0.09352517985611514 | 0.10400000000000002 | 0.140495867768595 | 0.0906862745098039 | 0.07099697885196382 | 0.0880195599022005 | 0.07049608355091384 | 0.07692307692307691 | 0.05518763796909492 | 0.09259259259259267 | 0.0780487804878049 | 0.0679851668726823 |
| 4.0000000000000015E-2 | 0.08035714285714296 | 0.14 | 0.06332453825857523 | 0.10860655737704904 | 0.123745819397993 | 0.0718232044198895 | 0.06273062730627313 | 0.09815950920245403 | 0.08992805755395683 | 0.14400000000000004 | 0.140495867768595 | 0.0906862745098039 | 0.09365558912386718 | 0.11735941320293397 | 0.08616187989556143 | 0.10931174089068803 | 0.059602649006622516 | 0.08888888888888893 | 0.08292682926829277 | 0.09641532756489495 |
| 6.0000000000000019E-2 | 0.11607142857142905 | 0.0825 | 0.0870712401055409 | 0.08401639344262297 | 0.11036789297658903 | 0.114917127071823 | 0.0738007380073801 | 0.07975460122699393 | 0.0755395683453237 | 0.096 | 0.0661157024793388 | 0.13235294117647106 | 0.09818731117824771 | 0.141809290953545 | 0.10182767624020905 | 0.135627530364372 | 0.08278145695364243 | 0.09629629629629634 | 0.11869918699187003 | 0.10630407911001202 |
| 8.0000000000000029E-2 | 0.0982142857142857 | 0.12000000000000002 | 0.08970976253298153 | 0.112704918032787 | 0.0802675585284281 | 0.103867403314917 | 0.08118081180811804 | 0.07361963190184054 | 0.0755395683453237 | 0.08000000000000003 | 0.0661157024793388 | 0.0906862745098039 | 0.09969788519637467 | 0.11858190709046497 | 0.0992167101827676 | 0.139676113360324 | 0.11479028697571705 | 0.08888888888888893 | 0.09756097560975616 | 0.12484548825710802 |
| 0.1 | 0.0982142857142857 | 0.07750000000000001 | 0.06068601583113463 | 0.0881147540983607 | 0.07692307692307691 | 0.103867403314917 | 0.04428044280442802 | 0.05521472392638042 | 0.057553956834532426 | 0.08800000000000004 | 0.0578512396694215 | 0.0784313725490196 | 0.11480362537764402 | 0.116136919315403 | 0.08616187989556143 | 0.07692307692307691 | 0.12141280353200902 | 0.048148148148148086 | 0.09593495934959355 | 0.103831891223733 |
| 0.12000000000000002 | 0.0580357142857143 | 0.10750000000000003 | 0.0422163588390501 | 0.07581967213114753 | 0.03678929765886292 | 0.0651933701657459 | 0.04428044280442802 | 0.03680981595092022 | 0.0719424460431655 | 0.048 | 0.08264462809917364 | 0.05147058823529408 | 0.08006042296072514 | 0.05745721271393642 | 0.06788511749347262 | 0.058704453441295504 | 0.0573951434878587 | 0.0666666666666667 | 0.06016260162601635 | 0.0667490729295426 |
| 0.14000000000000001 | 0.0625 | 0.065 | 0.0263852242744063 | 0.055327868852459 | 0.0434782608695652 | 0.0640883977900552 | 0.033210332103321014 | 0.04601226993865032 | 0.03237410071942452 | 0.024 | 0.04132231404958682 | 0.0563725490196078 | 0.049848942598187285 | 0.055012224938875344 | 0.05221932114882512 | 0.060728744939271335 | 0.043046357615893996 | 0.0518518518518519 | 0.04715447154471552 | 0.046971569839307795 |
| 0.16 | 0.044642857142857095 | 0.030000000000000002 | 0.0422163588390501 | 0.061475409836065614 | 0.0334448160535117 | 0.055248618784530384 | 0.040590405904059004 | 0.02760736196319021 | 0.04676258992805763 | 0.06400000000000003 | 0.0 | 0.0416666666666667 | 0.04833836858006038 | 0.04523227383863083 | 0.02872062663185379 | 0.058704453441295504 | 0.04194260485651212 | 0.02962962962962961 | 0.04552845528455278 | 0.0556242274412855 |
| 0.18000000000000005 | 0.0334821428571429 | 0.040000000000000015 | 0.0316622691292876 | 0.04303278688524593 | 0.0334448160535117 | 0.05193370165745862 | 0.018450184501845 | 0.030674846625766916 | 0.043165467625899304 | 0.016000000000000007 | 0.024793388429752112 | 0.00735294117647059 | 0.036253776435045314 | 0.024449877750611217 | 0.04177545691906009 | 0.0161943319838057 | 0.0706401766004415 | 0.011111111111111101 | 0.04552845528455278 | 0.0457354758961681 |
| 0.2 | 0.0357142857142857 | 0.025 | 0.023746701846965694 | 0.02663934426229512 | 0.0234113712374582 | 0.03867403314917131 | 0.025830258302583 | 0.03680981595092022 | 0.03237410071942452 | 0.016000000000000007 | 0.0 | 0.0343137254901961 | 0.04078549848942602 | 0.031784841075794615 | 0.023498694516971307 | 0.0182186234817814 | 0.04635761589403972 | 0.022222222222222202 | 0.034146341463414616 | 0.0420271940667491 |
| 0.22 | 0.024553571428571407 | 0.020000000000000007 | 0.021108179419525117 | 0.0389344262295082 | 0.013377926421404698 | 0.030939226519337008 | 0.03690036900369 | 0.02453987730061352 | 0.025179856115107892 | 0.0 | 0.008264462809917364 | 0.0367647058823529 | 0.027190332326284008 | 0.015892420537897307 | 0.026109660574412514 | 0.03643724696356281 | 0.030905077262693217 | 0.00740740740740741 | 0.030894308943089407 | 0.024721878862793607 |
| 0.24000000000000005 | 0.03125 | 0.020000000000000007 | 0.0263852242744063 | 0.0204918032786885 | 0.013377926421404698 | 0.025414364640884 | 0.018450184501845 | 0.015337423312883404 | 0.025179856115107892 | 0.008000000000000005 | 0.008264462809917364 | 0.0245098039215686 | 0.0317220543806647 | 0.01833740831295841 | 0.013054830287206304 | 0.01417004048583 | 0.033112582781457 | 0.02962962962962961 | 0.03577235772357722 | 0.018541409147095213 |
| 0.26 | 0.015625 | 0.010000000000000004 | 0.0263852242744063 | 0.02663934426229512 | 0.02006688963210699 | 0.022099447513812213 | 0.014760147601476 | 0.012269938650306698 | 0.0179856115107914 | 0.016000000000000007 | 0.016528925619834708 | 0.0196078431372549 | 0.025679758308157108 | 0.024449877750611217 | 0.0182767624020888 | 0.0182186234817814 | 0.025386313465783718 | 0.018518518518518507 | 0.024390243902439 | 0.028430160692212606 |
| 0.28000000000000008 | 0.008928571428571423 | 0.010000000000000004 | 0.0263852242744063 | 0.024590163934426198 | 0.013377926421404698 | 0.018784530386740307 | 0.040590405904059004 | 0.015337423312883404 | 0.0179856115107914 | 0.0 | 0.008264462809917364 | 0.012254901960784298 | 0.010574018126888199 | 0.011002444987775101 | 0.013054830287206304 | 0.00809716599190283 | 0.028697571743929402 | 0.011111111111111101 | 0.0146341463414634 | 0.023485784919653908 |
| 0.3000000000000001 | 0.00223214285714286 | 0.0125 | 0.007915567282321904 | 0.0163934426229508 | 0.003344481605351171 | 0.019889502762430906 | 0.0 | 0.021472392638036807 | 0.0071942446043165515 | 0.0 | 0.0 | 0.017156862745098 | 0.016616314199395802 | 0.012224938875305598 | 0.010443864229765006 | 0.002024291497975711 | 0.0187637969094923 | 0.011111111111111101 | 0.024390243902439 | 0.022249690976514216 |
| 0.32000000000000012 | 0.024553571428571407 | 0.007500000000000003 | 0.013192612137203198 | 0.00819672131147541 | 0.026755852842809402 | 0.0176795580110497 | 0.029520295202951997 | 0.021472392638036807 | 0.0071942446043165515 | 0.008000000000000005 | 0.0 | 0.00980392156862745 | 0.013595166163142 | 0.0146699266503667 | 0.010443864229765006 | 0.006072874493927133 | 0.0187637969094923 | 0.0148148148148148 | 0.0130081300813008 | 0.016069221260815808 |
| 0.34 | 0.013392857142857107 | 0.007500000000000003 | 0.010554089709762505 | 0.0163934426229508 | 0.0100334448160535 | 0.01215469613259669 | 0.018450184501845 | 0.015337423312883404 | 0.0071942446043165515 | 0.008000000000000005 | 0.016528925619834708 | 0.00980392156862745 | 0.016616314199395802 | 0.0036674816625916927 | 0.013054830287206304 | 0.010121457489878501 | 0.016556291390728502 | 0.011111111111111101 | 0.02113821138211381 | 0.007416563658838074 |
| 0.3600000000000001 | 0.011160714285714305 | 0.0 | 0.0263852242744063 | 0.00409836065573771 | 0.006688963210702342 | 0.011049723756906101 | 0.011070110701107005 | 0.012269938650306698 | 0.021582733812949607 | 0.0 | 0.016528925619834708 | 0.00980392156862745 | 0.013595166163142 | 0.009779951100244499 | 0.013054830287206304 | 0.002024291497975711 | 0.0176600441501104 | 0.0037037037037037017 | 0.00813008130081301 | 0.004944375772558711 |
| 0.38000000000000012 | 0.0223214285714286 | 0.002500000000000001 | 0.0184696569920844 | 0.00614754098360656 | 0.003344481605351171 | 0.01215469613259669 | 0.014760147601476 | 0.021472392638036807 | 0.014388489208633106 | 0.0 | 0.016528925619834708 | 0.00735294117647059 | 0.007552870090634443 | 0.012224938875305598 | 0.005221932114882512 | 0.002024291497975711 | 0.00883002207505519 | 0.00740740740740741 | 0.009756097560975615 | 0.004944375772558711 |
| 0.4 | 0.015625 | 0.007500000000000003 | 0.029023746701847 | 0.00409836065573771 | 0.003344481605351171 | 0.007734806629834252 | 0.02214022140221401 | 0.030674846625766916 | 0.010791366906474795 | 0.0 | 0.0 | 0.012254901960784298 | 0.007552870090634443 | 0.0024449877750611216 | 0.007832898172323764 | 0.0 | 0.00883002207505519 | 0.0148148148148148 | 0.01788617886178861 | 0.006180469715698394 |
### Chart: tph-1 A5
| Category | tph-1(mg280)_A5_01 | tph-1(mg280)_A5_02 | tph-1(mg280)_A5_03 | tph-1(mg280)_A5_04 | tph-1(mg280)_A5_05 | tph-1(mg280)_A5_06 | tph-1(mg280)_A5_07 | tph-1(mg280)_A5_08 | tph-1(mg280)_A5_10 | tph-1(mg280)_A5_11 | tph-1(mg280)_A5_12 | tph-1(mg280)_A5_13 | tph-1(mg280)_A5_14 | tph-1(mg280)_A5_15 | tph-1(mg280)_A5_16 | tph-1(mg280)_A5_17 | tph-1(mg280)_A5_18 | tph-1(mg280)_A5_19 | tph-1(mg280)_A5_20 |
|---|---|---|---|---|---|---|---|---|---|---|---|---|---|---|---|---|---|---|---|
| 0 | None | None | None | None | None | None | None | None | None | None | None | None | None | None | None | None | None | None | None |
| 2.0000000000000007E-2 | 0.130434782608696 | 0.12666666666666695 | 0.18461538461538504 | 0.06521739130434782 | 0.158301158301158 | 0.0993788819875776 | 0.115555555555556 | 0.08928571428571425 | 0.10126582278481006 | 0.1354466858789631 | 0.14044943820224712 | 0.09189189189189194 | 0.11149825783972098 | 0.12429378531073405 | 0.09585492227979278 | 0.12235294117647103 | 0.12857142857142906 | 0.0695187165775401 | 0.10489510489510502 |
| 4.0000000000000015E-2 | 0.09239130434782614 | 0.10666666666666705 | 0.12307692307692306 | 0.17391304347826111 | 0.13513513513513506 | 0.07142857142857141 | 0.15111111111111106 | 0.0535714285714286 | 0.10126582278481006 | 0.161383285302594 | 0.2134831460674161 | 0.13513513513513506 | 0.16376306620209105 | 0.08757062146892658 | 0.116580310880829 | 0.150588235294118 | 0.132142857142857 | 0.12655971479500897 | 0.10489510489510502 |
| 6.0000000000000019E-2 | 0.0815217391304348 | 0.08000000000000003 | 0.061538461538461514 | 0.0217391304347826 | 0.10038610038610005 | 0.10869565217391308 | 0.10666666666666705 | 0.0357142857142857 | 0.116455696202532 | 0.126801152737752 | 0.12359550561797802 | 0.0972972972972973 | 0.12543554006968594 | 0.096045197740113 | 0.0880829015544041 | 0.12235294117647103 | 0.13571428571428606 | 0.09090909090909094 | 0.125874125874126 |
| 8.0000000000000029E-2 | 0.06521739130434782 | 0.06000000000000002 | 0.030769230769230802 | 0.0217391304347826 | 0.07722007722007722 | 0.077639751552795 | 0.07555555555555557 | 0.07142857142857141 | 0.08607594936708864 | 0.0778097982708934 | 0.0842696629213483 | 0.0594594594594595 | 0.07665505226480838 | 0.08757062146892658 | 0.0880829015544041 | 0.10823529411764703 | 0.0642857142857143 | 0.0819964349376114 | 0.08391608391608396 |
| 0.1 | 0.03260869565217391 | 0.0333333333333333 | 0.030769230769230802 | 0.0434782608695652 | 0.0579150579150579 | 0.077639751552795 | 0.05333333333333334 | 0.017857142857142898 | 0.08607594936708864 | 0.060518731988472636 | 0.03932584269662921 | 0.0324324324324324 | 0.04878048780487813 | 0.09887005649717513 | 0.06476683937823834 | 0.0729411764705882 | 0.0642857142857143 | 0.115864527629234 | 0.111888111888112 |
| 0.12000000000000002 | 0.0380434782608696 | 0.020000000000000007 | 0.015384615384615405 | 0.0217391304347826 | 0.0347490347490347 | 0.031055900621118016 | 0.026666666666666707 | 0.08928571428571425 | 0.0455696202531646 | 0.0634005763688761 | 0.0168539325842697 | 0.0324324324324324 | 0.04181184668989548 | 0.053672316384180796 | 0.046632124352331626 | 0.06588235294117653 | 0.0357142857142857 | 0.06595365418894833 | 0.0629370629370629 |
| 0.14000000000000001 | 0.016304347826087 | 0.006666666666666671 | 0.030769230769230802 | 0.0 | 0.027027027027027015 | 0.031055900621118016 | 0.026666666666666707 | 0.0 | 0.05569620253164563 | 0.048991354466858796 | 0.06179775280898882 | 0.0324324324324324 | 0.038327526132404185 | 0.02824858757062151 | 0.0569948186528497 | 0.04235294117647062 | 0.028571428571428602 | 0.0641711229946524 | 0.055944055944055895 |
| 0.16 | 0.0217391304347826 | 0.013333333333333301 | 0.0 | 0.0 | 0.019305019305019308 | 0.0372670807453416 | 0.022222222222222202 | 0.0357142857142857 | 0.0405063291139241 | 0.0172910662824208 | 0.0112359550561798 | 0.0162162162162162 | 0.024390243902439 | 0.031073446327683614 | 0.04922279792746113 | 0.0352941176470588 | 0.017857142857142898 | 0.035650623885918005 | 0.02797202797202801 |
| 0.18000000000000005 | 0.027173913043478312 | 0.006666666666666671 | 0.030769230769230802 | 0.0217391304347826 | 0.0308880308880309 | 0.0341614906832298 | 0.013333333333333301 | 0.017857142857142898 | 0.030379746835443 | 0.0288184438040346 | 0.0168539325842697 | 0.0324324324324324 | 0.0174216027874564 | 0.01977401129943501 | 0.054404145077720185 | 0.011764705882352905 | 0.0321428571428571 | 0.0570409982174688 | 0.02797202797202801 |
| 0.2 | 0.0217391304347826 | 0.020000000000000007 | 0.0 | 0.0217391304347826 | 0.019305019305019308 | 0.0372670807453416 | 0.013333333333333301 | 0.017857142857142898 | 0.015189873417721501 | 0.00864553314121038 | 0.005617977528089888 | 0.021621621621621602 | 0.013937282229965204 | 0.0141242937853107 | 0.025906735751295307 | 0.011764705882352905 | 0.014285714285714301 | 0.03743315508021393 | 0.0 |
| 0.22 | 0.0 | 0.006666666666666671 | 0.0 | 0.0 | 0.011583011583011601 | 0.012422360248447206 | 0.013333333333333301 | 0.017857142857142898 | 0.020253164556962 | 0.023054755043227692 | 0.0112359550561798 | 0.021621621621621602 | 0.0174216027874564 | 0.02824858757062151 | 0.03886010362694301 | 0.01882352941176471 | 0.017857142857142898 | 0.023172905525846714 | 0.013986013986014 |
| 0.24000000000000005 | 0.0217391304347826 | 0.013333333333333301 | 0.0 | 0.0 | 0.011583011583011601 | 0.012422360248447206 | 0.0 | 0.0 | 0.012658227848101299 | 0.00864553314121038 | 0.0112359550561798 | 0.010810810810810801 | 0.013937282229965204 | 0.02824858757062151 | 0.025906735751295307 | 0.014117647058823493 | 0.025 | 0.026737967914438505 | 0.020979020979021008 |
| 0.26 | 0.010869565217391306 | 0.006666666666666671 | 0.015384615384615405 | 0.0 | 0.011583011583011601 | 0.0217391304347826 | 0.0 | 0.0 | 0.005063291139240512 | 0.0028818443804034598 | 0.005617977528089888 | 0.005405405405405412 | 0.006968641114982585 | 0.0141242937853107 | 0.031088082901554407 | 0.007058823529411772 | 0.007142857142857142 | 0.0196078431372549 | 0.006993006993006992 |
| 0.28000000000000008 | 0.0 | 0.006666666666666671 | 0.015384615384615405 | 0.0 | 0.0038610038610038602 | 0.0248447204968944 | 0.0 | 0.0 | 0.0177215189873418 | 0.0028818443804034598 | 0.005617977528089888 | 0.005405405405405412 | 0.006968641114982585 | 0.01977401129943501 | 0.0181347150259067 | 0.0164705882352941 | 0.010714285714285704 | 0.021390374331550797 | 0.0 |
| 0.3000000000000001 | 0.016304347826087 | 0.013333333333333301 | 0.0 | 0.0 | 0.011583011583011601 | 0.012422360248447206 | 0.004444444444444442 | 0.017857142857142898 | 0.012658227848101299 | 0.00576368876080692 | 0.0 | 0.0 | 0.0034843205574912927 | 0.016949152542372906 | 0.015544041450777204 | 0.0 | 0.0 | 0.0106951871657754 | 0.0 |
| 0.32000000000000012 | 0.0 | 0.0 | 0.0 | 0.0 | 0.0 | 0.0093167701863354 | 0.008888888888888894 | 0.0357142857142857 | 0.0177215189873418 | 0.0 | 0.005617977528089888 | 0.0 | 0.0034843205574912927 | 0.016949152542372906 | 0.0181347150259067 | 0.007058823529411772 | 0.007142857142857142 | 0.017825311942959002 | 0.006993006993006992 |
| 0.34 | 0.016304347826087 | 0.020000000000000007 | 0.0 | 0.0 | 0.011583011583011601 | 0.0217391304347826 | 0.008888888888888894 | 0.0 | 0.005063291139240512 | 0.0028818443804034598 | 0.0 | 0.0 | 0.0034843205574912927 | 0.008474576271186448 | 0.00259067357512953 | 0.0 | 0.007142857142857142 | 0.007130124777183602 | 0.013986013986014 |
| 0.3600000000000001 | 0.010869565217391306 | 0.006666666666666671 | 0.0 | 0.0 | 0.015444015444015408 | 0.006211180124223604 | 0.0 | 0.0 | 0.010126582278481 | 0.0028818443804034598 | 0.0 | 0.0 | 0.0034843205574912927 | 0.0141242937853107 | 0.015544041450777204 | 0.007058823529411772 | 0.003571428571428571 | 0.012477718360071298 | 0.0 |
| 0.38000000000000012 | 0.0 | 0.0 | 0.0 | 0.0 | 0.007722007722007722 | 0.0093167701863354 | 0.004444444444444442 | 0.0 | 0.0025316455696202493 | 0.0028818443804034598 | 0.0 | 0.0 | 0.0034843205574912927 | 0.008474576271186448 | 0.007772020725388605 | 0.007058823529411772 | 0.007142857142857142 | 0.012477718360071298 | 0.006993006993006992 |
| 0.4 | 0.0 | 0.013333333333333301 | 0.0 | 0.0 | 0.007722007722007722 | 0.012422360248447206 | 0.004444444444444442 | 0.017857142857142898 | 0.0 | 0.0 | 0.0 | 0.0 | 0.0034843205574912927 | 0.01977401129943501 | 0.010362694300518104 | 0.004705882352941182 | 0.007142857142857142 | 0.0 | 0.006993006993006992 |
### Chart: cat-1 A1
| Category | cat-1(CB1111)_A1_01 | cat-1(CB1111)_A1_02 | cat-1(CB1111)_A1_03 | cat-1(CB1111)_A1_04 | cat-1(CB1111)_A1_05 | cat-1(CB1111)_A1_06 | cat-1(CB1111)_A1_07 | cat-1(CB1111)_A1_08 | cat-1(CB1111)_A1_09 | cat-1(CB1111)_A1_10 | cat-1(CB1111)_A1_11 | cat-1(CB1111)_A1_12 | cat-1(CB1111)_A1_13 | cat-1(CB1111)_A1_14 | cat-1(CB1111)_A1_15 | cat-1(CB1111)_A1_16 | cat-1(CB1111)_A1_17 | cat-1(CB1111)_A1_18 | cat-1(CB1111)_A1_19 | cat-1(CB1111)_A1_20 |
|---|---|---|---|---|---|---|---|---|---|---|---|---|---|---|---|---|---|---|---|---|
| 0 | None | None | None | None | None | None | None | None | None | None | None | None | None | None | None | None | None | None | None | None |
| 2.0000000000000007E-2 | 0.08487084870848718 | 0.07561728395061734 | 0.10500000000000002 | 0.09584664536741216 | 0.09014084507042255 | 0.07125307125307133 | 0.08366533864541835 | 0.08608058608058608 | 0.08531317494600424 | 0.0705882352941176 | None | 0.0661290322580645 | 0.0695187165775401 | 0.07613814756671897 | 0.08996815286624205 | 0.0611814345991561 | 0.07005838198498751 | 0.08068459657701715 | 0.0671798460461861 | 0.0792483660130719 |
| 4.0000000000000015E-2 | 0.07749077490774914 | 0.08641975308641968 | 0.17 | 0.0862619808306709 | 0.09014084507042255 | 0.09500409500409507 | 0.10756972111553803 | 0.08791208791208788 | 0.09611231101511873 | 0.096078431372549 | None | 0.0951612903225806 | 0.11140819964349395 | 0.0957613814756672 | 0.11226114649681505 | 0.07454289732770751 | 0.08590492076730617 | 0.10350448247758805 | 0.11336599020293903 | 0.09722222222222222 |
| 6.0000000000000019E-2 | 0.11439114391143905 | 0.126543209876543 | 0.13 | 0.10223642172524006 | 0.10704225352112705 | 0.125307125307125 | 0.08565737051792832 | 0.12362637362637405 | 0.128509719222462 | 0.09117647058823533 | None | 0.125 | 0.09893048128342254 | 0.103610675039246 | 0.11863057324840805 | 0.127988748241913 | 0.12010008340283602 | 0.114099429502852 | 0.116864940517845 | 0.130718954248366 |
| 8.0000000000000029E-2 | 0.0738007380073801 | 0.09259259259259267 | 0.08000000000000003 | 0.115015974440895 | 0.130985915492958 | 0.1384111384111381 | 0.09960159362549803 | 0.137362637362637 | 0.14254859611231108 | 0.1 | None | 0.12822580645161297 | 0.157754010695187 | 0.14756671899529006 | 0.14888535031847105 | 0.139943741209564 | 0.134278565471226 | 0.15321923390383008 | 0.160251924422673 | 0.12254901960784295 |
| 0.1 | 0.07011070110701113 | 0.08796296296296303 | 0.09000000000000002 | 0.10223642172524006 | 0.08591549295774656 | 0.11957411957412002 | 0.0816733067729084 | 0.115384615384615 | 0.09287257019438452 | 0.11764705882352898 | None | 0.12741935483871006 | 0.13190730837789708 | 0.12166405023547905 | 0.12022292993630604 | 0.149085794655415 | 0.14512093411176005 | 0.12632436837815794 | 0.133659902029391 | 0.1576797385620911 |
| 0.12000000000000002 | 0.0590405904059041 | 0.08333333333333333 | 0.065 | 0.07987220447284345 | 0.08591549295774656 | 0.07862407862407857 | 0.0697211155378486 | 0.08791208791208788 | 0.06803455723542123 | 0.08039215686274508 | None | 0.06532258064516133 | 0.07486631016042783 | 0.07299843014128733 | 0.055732484076433136 | 0.07454289732770751 | 0.0600500417014178 | 0.06193969030154852 | 0.06508047585724283 | 0.08578431372549024 |
| 0.14000000000000001 | 0.0479704797047971 | 0.0617283950617284 | 0.05 | 0.05750798722044734 | 0.05774647887323942 | 0.055692055692055695 | 0.055776892430278904 | 0.05860805860805857 | 0.0561555075593952 | 0.04313725490196081 | None | 0.05080645161290324 | 0.05614973262032088 | 0.0384615384615385 | 0.04060509554140131 | 0.05203938115330522 | 0.0508757297748123 | 0.057864710676446635 | 0.0468859342197341 | 0.04656862745098042 |
| 0.16 | 0.0479704797047971 | 0.066358024691358 | 0.04500000000000001 | 0.047923322683706096 | 0.0549295774647887 | 0.06633906633906632 | 0.06175298804780882 | 0.0604395604395604 | 0.047516198704103715 | 0.0509803921568627 | None | 0.0370967741935484 | 0.053475935828877004 | 0.054160125588697 | 0.04697452229299358 | 0.0330520393811533 | 0.0508757297748123 | 0.04971475142624293 | 0.0524842547235829 | 0.049019607843137344 |
| 0.18000000000000005 | 0.04428044280442802 | 0.0416666666666667 | 0.020000000000000007 | 0.05111821086261981 | 0.03943661971830991 | 0.040950040950040914 | 0.0438247011952191 | 0.042124542124542086 | 0.060475161987041 | 0.040196078431372614 | None | 0.04516129032258062 | 0.04456327985739753 | 0.04474097331240192 | 0.04060509554140131 | 0.04571026722925462 | 0.04670558798999172 | 0.05460472697636511 | 0.044786564030790836 | 0.0367647058823529 |
| 0.2 | 0.025830258302583 | 0.040123456790123496 | 0.014999999999999998 | 0.0383386581469649 | 0.0323943661971831 | 0.03521703521703521 | 0.0537848605577689 | 0.03479853479853479 | 0.0323974082073434 | 0.04509803921568633 | None | 0.04112903225806448 | 0.0454545454545455 | 0.040031397174254295 | 0.0414012738853503 | 0.049929676511954985 | 0.03669724770642203 | 0.035859820700896515 | 0.037788663400979715 | 0.03349673202614381 |
| 0.22 | 0.05166051660516609 | 0.018518518518518507 | 0.020000000000000007 | 0.0319488817891374 | 0.022535211267605614 | 0.029484029484029513 | 0.039840637450199216 | 0.02747252747252752 | 0.0323974082073434 | 0.03823529411764711 | None | 0.0346774193548387 | 0.025846702317290606 | 0.0259026687598116 | 0.03423566878980892 | 0.0330520393811533 | 0.03002502085070889 | 0.029339853300733507 | 0.023093072078376513 | 0.02859477124183008 |
| 0.24000000000000005 | 0.029520295202951997 | 0.03086419753086421 | 0.014999999999999998 | 0.0223642172523962 | 0.03943661971830991 | 0.017199017199017202 | 0.0219123505976096 | 0.0164835164835165 | 0.0140388768898488 | 0.0274509803921569 | None | 0.0241935483870968 | 0.0196078431372549 | 0.022762951334379892 | 0.0207006369426752 | 0.0267229254571027 | 0.0225187656380317 | 0.0187449062754686 | 0.0167949615115465 | 0.017156862745098 |
| 0.26 | 0.025830258302583 | 0.00925925925925927 | 0.010000000000000004 | 0.019169329073482407 | 0.022535211267605614 | 0.017199017199017202 | 0.027888446215139407 | 0.014652014652014699 | 0.015118790496760301 | 0.02843137254901962 | None | 0.009677419354838717 | 0.011586452762923404 | 0.019623233908948202 | 0.019108280254777107 | 0.023909985935302393 | 0.015846538782318606 | 0.012224938875305598 | 0.009797060881735476 | 0.014705882352941199 |
| 0.28000000000000008 | 0.025830258302583 | 0.026234567901234608 | 0.0 | 0.0223642172523962 | 0.016901408450704206 | 0.012285012285012303 | 0.0199203187250996 | 0.011904761904761904 | 0.007559395248380133 | 0.0215686274509804 | None | 0.0201612903225806 | 0.012477718360071298 | 0.0164835164835165 | 0.00955414012738853 | 0.020393811533052 | 0.014178482068390298 | 0.008149959250203754 | 0.0111966410076977 | 0.00980392156862745 |
| 0.3000000000000001 | 0.003690036900369001 | 0.00925925925925927 | 0.0 | 0.00958466453674122 | 0.008450704225352117 | 0.00819000819000819 | 0.0159362549800797 | 0.003663003663003661 | 0.006479481641468683 | 0.0156862745098039 | None | 0.0137096774193548 | 0.004456327985739753 | 0.0141287284144427 | 0.007961783439490453 | 0.00914205344585091 | 0.00917431192660551 | 0.012224938875305598 | 0.009797060881735476 | 0.0065359477124183035 |
| 0.32000000000000012 | 0.014760147601476 | 0.004629629629629632 | 0.014999999999999998 | 0.00638977635782748 | 0.008450704225352117 | 0.007371007371007372 | 0.007968127490039844 | 0.0027472527472527527 | 0.008639308855291579 | 0.008823529411764714 | None | 0.014516129032258105 | 0.004456327985739753 | 0.010204081632653106 | 0.008757961783439487 | 0.009845288326300997 | 0.005004170141784822 | 0.007334963325183374 | 0.005598320503848852 | 0.0057189542483660075 |
| 0.34 | 0.014760147601476 | 0.003086419753086421 | 0.005000000000000002 | 0.0 | 0.004225352112676062 | 0.00491400491400491 | 0.009960159362549804 | 0.00549450549450549 | 0.007559395248380133 | 0.003921568627450981 | None | 0.007258064516129032 | 0.004456327985739753 | 0.00706436420722135 | 0.007165605095541402 | 0.00492264416315049 | 0.0100083402835696 | 0.003259983700081502 | 0.007697690692792163 | 0.00245098039215686 |
| 0.3600000000000001 | 0.011070110701107005 | 0.0015432098765432104 | 0.0 | 0.00638977635782748 | 0.004225352112676062 | 0.00491400491400491 | 0.007968127490039844 | 0.003663003663003661 | 0.005399568034557242 | 0.00980392156862745 | None | 0.006451612903225813 | 0.0053475935828877 | 0.006279434850863422 | 0.0031847133757961815 | 0.00492264416315049 | 0.006672226855713092 | 0.004074979625101873 | 0.00489853044086774 | 0.0057189542483660075 |
| 0.38000000000000012 | 0.003690036900369001 | 0.004629629629629632 | 0.005000000000000002 | 0.00319488817891374 | 0.0028169014084507 | 0.00327600327600328 | 0.00199203187250996 | 0.003663003663003661 | 0.003239740820734341 | 0.004901960784313736 | None | 0.006451612903225813 | 0.0 | 0.006279434850863422 | 0.003980891719745221 | 0.006329113924050632 | 0.0058381984987489624 | 0.004074979625101873 | 0.00419874037788663 | 0.00816993464052288 |
| 0.4 | 0.011070110701107005 | 0.00617283950617284 | 0.0 | 0.00958466453674122 | 0.0028169014084507 | 0.002457002457002461 | 0.00199203187250996 | 0.0027472527472527527 | 0.003239740820734341 | 0.00686274509803922 | None | 0.006451612903225813 | 0.003565062388591801 | 0.0047095761381475715 | 0.0015923566878980901 | 0.003516174402250352 | 0.0008340283569641374 | 0.00162999185004075 | 0.002799160251924422 | 0.00408496732026144 |
### Chart: cat-1 A3
| Category | cat-1(CB1111)_A3_01 | cat-1(CB1111)_A3_02 | cat-1(CB1111)_A3_03 | cat-1(CB1111)_A3_04 | cat-1(CB1111)_A3_05 | cat-1(CB1111)_A3_06 | cat-1(CB1111)_A3_07 | cat-1(CB1111)_A3_08 | cat-1(CB1111)_A3_09 | cat-1(CB1111)_A3_10 | cat-1(CB1111)_A3_11 | cat-1(CB1111)_A3_12 | cat-1(CB1111)_A3_13 | cat-1(CB1111)_A3_14 | cat-1(CB1111)_A3_15 | cat-1(CB1111)_A3_16 | cat-1(CB1111)_A3_17 | cat-1(CB1111)_A3_18 | cat-1(CB1111)_A3_19 | cat-1(CB1111)_A3_20 |
|---|---|---|---|---|---|---|---|---|---|---|---|---|---|---|---|---|---|---|---|---|
| 0 | None | None | None | None | None | None | None | None | None | None | None | None | None | None | None | None | None | None | None | None |
| 2.0000000000000007E-2 | 0.0860849056603774 | 0.07502930832356389 | 0.103407755581669 | 0.07822410147991539 | 0.08097686375321347 | 0.07329842931937172 | 0.0715935334872979 | 0.0928176795580111 | 0.07912844036697249 | 0.08251633986928103 | 0.059829059829059804 | 0.07792207792207793 | 0.059011164274322216 | 0.0689102564102564 | 0.08265424912689176 | 0.08450704225352115 | 0.09344790547798071 | 0.07088607594936713 | 0.0706190061028771 | 0.0670241286863271 |
| 4.0000000000000015E-2 | 0.09433962264150943 | 0.110199296600234 | 0.07873090481786135 | 0.101479915433404 | 0.10796915167095103 | 0.09293193717277488 | 0.09815242494226335 | 0.103867403314917 | 0.0894495412844037 | 0.09640522875816994 | 0.08831908831908833 | 0.07674144037780399 | 0.10207336523126002 | 0.0977564102564103 | 0.08381839348079163 | 0.08148893360160966 | 0.10096670247046204 | 0.0759493670886076 | 0.10113339145597203 | 0.0929401251117069 |
| 6.0000000000000019E-2 | 0.0766509433962264 | 0.116060961313013 | 0.0681551116333725 | 0.118393234672304 | 0.12339331619537297 | 0.12434554973822005 | 0.13394919168591207 | 0.114917127071823 | 0.11697247706422002 | 0.11683006535947697 | 0.07692307692307691 | 0.0755608028335301 | 0.057416267942583754 | 0.09294871794871794 | 0.06984866123399303 | 0.09557344064386324 | 0.09881847475832443 | 0.08987341772151898 | 0.09677419354838712 | 0.0804289544235925 |
| 8.0000000000000029E-2 | 0.09787735849056603 | 0.126611957796014 | 0.14688601645123406 | 0.13424947145877406 | 0.12724935732647807 | 0.121727748691099 | 0.14203233256351006 | 0.113812154696133 | 0.12729357798165095 | 0.127450980392157 | 0.09971509971509977 | 0.113341204250295 | 0.105263157894737 | 0.07371794871794873 | 0.09080325960419089 | 0.08953722334004026 | 0.11385606874328702 | 0.10253164556962004 | 0.11508282476024402 | 0.10098302055406598 |
| 0.1 | 0.11792452830188703 | 0.09847596717467767 | 0.103407755581669 | 0.127906976744186 | 0.105398457583548 | 0.09424083769633508 | 0.092378752886836 | 0.10718232044198908 | 0.11697247706422002 | 0.129084967320261 | 0.09971509971509977 | 0.08382526564344757 | 0.09728867623604466 | 0.09615384615384627 | 0.10942956926658909 | 0.111670020120724 | 0.105263157894737 | 0.0784810126582279 | 0.11857018308631204 | 0.14209115281501306 |
| 0.12000000000000002 | 0.0613207547169811 | 0.07854630715123102 | 0.0669800235017626 | 0.07505285412262162 | 0.08868894601542421 | 0.0850785340314136 | 0.05311778290993073 | 0.06740331491712713 | 0.0642201834862385 | 0.071078431372549 | 0.058404558404558396 | 0.037780401416765114 | 0.059011164274322216 | 0.0496794871794872 | 0.04656577415599532 | 0.0533199195171026 | 0.035445757250268516 | 0.0455696202531646 | 0.052310374891020035 | 0.0688114387846291 |
| 0.14000000000000001 | 0.0542452830188679 | 0.06447831184056273 | 0.03642773207990602 | 0.03911205073995769 | 0.06041131105398461 | 0.04842931937172778 | 0.05311778290993073 | 0.059668508287292796 | 0.0745412844036697 | 0.045751633986928136 | 0.0427350427350427 | 0.042502951593860715 | 0.0414673046251994 | 0.05929487179487181 | 0.04656577415599532 | 0.03521126760563382 | 0.04403866809881852 | 0.043037974683544304 | 0.05405405405405411 | 0.046470062555853384 |
| 0.16 | 0.056603773584905696 | 0.04689331770222741 | 0.038777908343125715 | 0.0496828752642706 | 0.048843187660668384 | 0.056282722513088995 | 0.04503464203233262 | 0.04972375690607732 | 0.055045871559633 | 0.05555555555555558 | 0.0512820512820513 | 0.050767414403778036 | 0.062200956937799014 | 0.0336538461538462 | 0.0360884749708964 | 0.05231388329979882 | 0.04511278195488724 | 0.0417721518987342 | 0.043591979075849996 | 0.05361930294906171 |
| 0.18000000000000005 | 0.0389150943396226 | 0.0351699882766706 | 0.023501762632197398 | 0.03594080338266381 | 0.0334190231362468 | 0.043193717277486915 | 0.0415704387990762 | 0.024309392265193408 | 0.03096330275229361 | 0.0392156862745098 | 0.04131054131054132 | 0.03187721369539553 | 0.043062200956937836 | 0.036858974358974415 | 0.0360884749708964 | 0.04527162977867198 | 0.03222341568206232 | 0.048101265822784796 | 0.045335658238884004 | 0.04200178731009832 |
| 0.2 | 0.041273584905660396 | 0.0386869871043376 | 0.0305522914218566 | 0.029598308668076112 | 0.035989717223650415 | 0.03926701570680632 | 0.0392609699769053 | 0.0342541436464088 | 0.034403669724770616 | 0.0449346405228758 | 0.0284900284900285 | 0.028335301062573814 | 0.035087719298245605 | 0.043269230769230796 | 0.0488940628637951 | 0.0291750503018109 | 0.034371643394199805 | 0.043037974683544304 | 0.030514385353095 | 0.0455764075067024 |
| 0.22 | 0.03183962264150941 | 0.0257913247362251 | 0.0352526439482961 | 0.029598308668076112 | 0.0218508997429306 | 0.0301047120418848 | 0.036951501154734404 | 0.018784530386740307 | 0.0298165137614679 | 0.029411764705882398 | 0.0527065527065527 | 0.04958677685950413 | 0.04784688995215312 | 0.04006410256410259 | 0.04074505238649588 | 0.03420523138833001 | 0.031149301825993615 | 0.0455696202531646 | 0.03400174367916302 | 0.0348525469168901 |
| 0.24000000000000005 | 0.035377358490566016 | 0.022274325908558008 | 0.031727379553466516 | 0.0285412262156448 | 0.019280205655527006 | 0.026178010471204216 | 0.0242494226327945 | 0.029834254143646398 | 0.024082568807339493 | 0.026143790849673214 | 0.0284900284900285 | 0.036599763872491115 | 0.0303030303030303 | 0.0416666666666667 | 0.0558789289871944 | 0.0442655935613682 | 0.030075187969924817 | 0.0405063291139241 | 0.030514385353095 | 0.026809651474530807 |
| 0.26 | 0.017688679245283008 | 0.012895662368112501 | 0.016451233842538205 | 0.013742071881606805 | 0.016709511568123406 | 0.0170157068062827 | 0.0173210161662818 | 0.01657458563535911 | 0.018348623853211 | 0.017156862745098 | 0.0341880341880342 | 0.027154663518299905 | 0.025518341307815 | 0.02564102564102561 | 0.041909196740395796 | 0.0331991951710262 | 0.0182599355531686 | 0.0405063291139241 | 0.020052310374891007 | 0.032171581769437 |
| 0.28000000000000008 | 0.023584905660377402 | 0.0175849941383353 | 0.021151586368977692 | 0.0221987315010571 | 0.012853470437018007 | 0.015706806282722505 | 0.0092378752886836 | 0.019889502762430906 | 0.011467889908256904 | 0.013888888888888907 | 0.02991452991452989 | 0.0212514757969303 | 0.020733652312599708 | 0.019230769230769208 | 0.02095459837019789 | 0.028169014084506998 | 0.02577873254564981 | 0.024050632911392398 | 0.013949433304272004 | 0.021447721179624717 |
| 0.3000000000000001 | 0.02004716981132081 | 0.021101992966002306 | 0.0152761457109283 | 0.011627906976744195 | 0.006426735218509 | 0.00916230366492147 | 0.0115473441108545 | 0.015469613259668504 | 0.00917431192660551 | 0.011437908496732 | 0.0227920227920228 | 0.024793388429752112 | 0.03189792663476871 | 0.03044871794871791 | 0.018626309662398106 | 0.02213279678068411 | 0.017185821697099906 | 0.0189873417721519 | 0.016564952048823006 | 0.0205540661304736 |
| 0.32000000000000012 | 0.0224056603773585 | 0.009378663540445495 | 0.0105757931844888 | 0.008456659619450328 | 0.006426735218509 | 0.005235602094240845 | 0.0138568129330254 | 0.0143646408839779 | 0.003440366972477062 | 0.00735294117647059 | 0.019943019943019908 | 0.028335301062573814 | 0.027113237639553416 | 0.011217948717948699 | 0.022118742724097806 | 0.0191146881287726 | 0.016111707841031202 | 0.020253164556962 | 0.013949433304272004 | 0.010723860589812305 |
| 0.34 | 0.02004716981132081 | 0.0011723329425556905 | 0.0141010575793184 | 0.0073995771670190315 | 0.0077120822622108 | 0.013089005235602108 | 0.005773672055427254 | 0.01215469613259669 | 0.011467889908256904 | 0.0032679738562091526 | 0.01566951566951571 | 0.0236127508854782 | 0.0159489633173844 | 0.016025641025641 | 0.0162980209545984 | 0.015090543259557304 | 0.017185821697099906 | 0.0177215189873418 | 0.00871839581517001 | 0.01340482573726541 |
| 0.3600000000000001 | 0.0106132075471698 | 0.0035169988276670602 | 0.0152761457109283 | 0.009513742071881619 | 0.005141388174807202 | 0.00654450261780105 | 0.0092378752886836 | 0.00662983425414365 | 0.010321100917431204 | 0.0016339869281045804 | 0.014245014245014204 | 0.0153482880755608 | 0.009569377990430622 | 0.011217948717948699 | 0.022118742724097806 | 0.024144869215291694 | 0.017185821697099906 | 0.021518987341772197 | 0.013949433304272004 | 0.008042895442359253 |
| 0.38000000000000012 | 0.00471698113207547 | 0.0011723329425556905 | 0.011750881316098707 | 0.00317124735729387 | 0.0038560411311053997 | 0.00654450261780105 | 0.0046189376443418 | 0.0132596685082873 | 0.006880733944954134 | 0.0065359477124183035 | 0.011396011396011404 | 0.008264462809917364 | 0.0015948963317384405 | 0.008012820512820514 | 0.0116414435389988 | 0.008048289738430577 | 0.00966702470461869 | 0.005063291139240512 | 0.00871839581517001 | 0.009830205540661314 |
| 0.4 | 0.0035377358490566017 | 0.00468933177022274 | 0.012925969447708604 | 0.005285412262156448 | 0.001285347043701801 | 0.0013089005235602108 | 0.0011547344110854501 | 0.008839779005524864 | 0.002293577981651382 | 0.00245098039215686 | 0.011396011396011404 | 0.009445100354191265 | 0.011164274322169104 | 0.011217948717948699 | 0.006984866123399303 | 0.011066398390342104 | 0.004296455424274972 | 0.00759493670886076 | 0.00871839581517001 | 0.00357462019660411 |
### Chart: cat-1 A5
| Category | cat-1(CB1111)_A5_01 | cat-1(CB1111)_A5_02 | cat-1(CB1111)_A5_03 | cat-1(CB1111)_A5_04 | cat-1(CB1111)_A5_05 | cat-1(CB1111)_A5_06 | cat-1(CB1111)_A5_07 | cat-1(CB1111)_A5_08 | cat-1(CB1111)_A5_10 | cat-1(CB1111)_A5_11 | cat-1(CB1111)_A5_12 | cat-1(CB1111)_A5_13 | cat-1(CB1111)_A5_14 | cat-1(CB1111)_A5_15 | cat-1(CB1111)_A5_16 | cat-1(CB1111)_A5_17 | cat-1(CB1111)_A5_18 | cat-1(CB1111)_A5_19 | cat-1(CB1111)_A5_20 | cat-1(CB1111)_A5_21 | cat-1(CB1111)_A5_22 | cat-1(CB1111)_A5_23 | cat-1(CB1111)_A5_24 | cat-1(CB1111)_A5_25 | cat-1(CB1111)_A5_26 | cat-1(CB1111)_A5_27 | cat-1(CB1111)_A5_28 | cat-1(CB1111)_A5_29 | cat-1(CB1111)_A5_30 |
|---|---|---|---|---|---|---|---|---|---|---|---|---|---|---|---|---|---|---|---|---|---|---|---|---|---|---|---|---|---|
| 0 | None | None | None | None | None | None | None | None | None | None | None | None | None | None | None | None | None | None | None | None | None | None | None | None | None | None | None | None | None |
| 2.0000000000000007E-2 | 0.125 | 0.10857142857142905 | 0.08156028368794328 | 0.08192090395480235 | 0.07052023121387281 | 0.10648148148148104 | 0.15151515151515207 | 0.11634349030470896 | 0.0654761904761905 | 0.0656620021528525 | 0.0831435079726651 | 0.135802469135802 | 0.08850726552179668 | 0.07823129251700682 | 0.0898876404494382 | 0.101522842639594 | 0.06335403726708072 | 0.0708050436469447 | 0.16969696969697 | 0.155555555555556 | 0.07673267326732673 | 0.0968523002421307 | 0.0788381742738589 | 0.08095238095238103 | 0.05326460481099664 | 0.0823863636363636 | 0.06534954407294831 | 0.0696969696969697 | 0.148541114058355 |
| 4.0000000000000015E-2 | 0.10416666666666703 | 0.11428571428571403 | 0.10283687943262403 | 0.14265536723163794 | 0.0971098265895954 | 0.135416666666667 | 0.10606060606060604 | 0.11080332409972295 | 0.08779761904761903 | 0.10010764262648003 | 0.09908883826879274 | 0.14814814814814806 | 0.12285336856010598 | 0.08333333333333333 | 0.104868913857678 | 0.11928934010152302 | 0.0993788819875776 | 0.11542192046556703 | 0.13333333333333305 | 0.09876543209876547 | 0.08910891089108916 | 0.10169491525423703 | 0.05394190871369288 | 0.0642857142857143 | 0.09450171821305843 | 0.07102272727272732 | 0.0790273556231003 | 0.0393939393939394 | 0.148541114058355 |
| 6.0000000000000019E-2 | 0.10416666666666703 | 0.0914285714285714 | 0.10283687943262403 | 0.13276836158192115 | 0.10173410404624306 | 0.16898148148148115 | 0.12121212121212104 | 0.124653739612188 | 0.08333333333333333 | 0.0914962325080732 | 0.11617312072892909 | 0.09876543209876547 | 0.116248348745046 | 0.0799319727891157 | 0.116104868913858 | 0.08375634517766513 | 0.127950310559006 | 0.08438409311348218 | 0.103030303030303 | 0.09382716049382726 | 0.09900990099009907 | 0.10895883777239697 | 0.06846473029045642 | 0.052380952380952396 | 0.09106529209621997 | 0.07528409090909091 | 0.07446808510638303 | 0.051515151515151486 | 0.09283819628647214 |
| 8.0000000000000029E-2 | 0.08333333333333333 | 0.0914285714285714 | 0.12174940898345203 | 0.12853107344632805 | 0.10289017341040503 | 0.138888888888889 | 0.0606060606060606 | 0.11911357340720204 | 0.11011904761904796 | 0.12163616792249703 | 0.09794988610478358 | 0.09876543209876547 | 0.12681638044914106 | 0.086734693877551 | 0.056179775280898875 | 0.0939086294416244 | 0.120496894409938 | 0.0989330746847721 | 0.07272727272727271 | 0.06419753086419752 | 0.09405940594059417 | 0.07021791767554482 | 0.0954356846473029 | 0.0571428571428571 | 0.10137457044673504 | 0.119318181818182 | 0.10638297872340402 | 0.0757575757575758 | 0.06100795755968172 |
| 0.1 | 0.02083333333333331 | 0.0514285714285714 | 0.124113475177305 | 0.11581920903954797 | 0.10867052023121408 | 0.116898148148148 | 0.0454545454545455 | 0.0692520775623269 | 0.11607142857142905 | 0.12378902045209904 | 0.127562642369021 | 0.08230452674897121 | 0.104359313077939 | 0.1717687074829931 | 0.0599250936329588 | 0.0685279187817259 | 0.120496894409938 | 0.133850630455868 | 0.0363636363636364 | 0.0395061728395062 | 0.05445544554455448 | 0.06295399515738503 | 0.05394190871369288 | 0.0571428571428571 | 0.0893470790378007 | 0.10937500000000003 | 0.10790273556231003 | 0.0696969696969697 | 0.0636604774535809 |
| 0.12000000000000002 | 0.0625 | 0.0742857142857143 | 0.08274231678487003 | 0.09463276836158195 | 0.08208092485549127 | 0.07754629629629632 | 0.0757575757575758 | 0.049861495844875335 | 0.0773809523809524 | 0.0785791173304629 | 0.0660592255125285 | 0.049382716049382734 | 0.07529722589167771 | 0.0561224489795918 | 0.056179775280898875 | 0.08629441624365483 | 0.06956521739130432 | 0.06789524733268673 | 0.0303030303030303 | 0.0395061728395062 | 0.0346534653465347 | 0.036319612590799015 | 0.0580912863070539 | 0.0333333333333333 | 0.0756013745704467 | 0.051136363636363584 | 0.04711246200607901 | 0.0303030303030303 | 0.0397877984084881 |
| 0.14000000000000001 | 0.0 | 0.0571428571428571 | 0.042553191489361715 | 0.0451977401129944 | 0.0763005780346821 | 0.05439814814814812 | 0.0303030303030303 | 0.041551246537396086 | 0.061011904761904795 | 0.06135629709364912 | 0.0432801822323462 | 0.016460905349794205 | 0.06472919418758262 | 0.042517006802721136 | 0.0411985018726592 | 0.040609137055837616 | 0.06211180124223603 | 0.0533462657613967 | 0.048484848484848485 | 0.012345679012345704 | 0.032178217821782214 | 0.0532687651331719 | 0.039419087136929515 | 0.019047619047619008 | 0.058419243986254296 | 0.046874999999999986 | 0.05623100303951371 | 0.0393939393939394 | 0.026525198938992 |
| 0.16 | 0.02083333333333331 | 0.03428571428571432 | 0.04373522458628842 | 0.0466101694915254 | 0.053179190751445095 | 0.03125 | 0.0303030303030303 | 0.03601108033241 | 0.04910714285714291 | 0.0602798708288482 | 0.037585421412300715 | 0.02880658436213989 | 0.04491413474240423 | 0.032312925170068 | 0.0449438202247191 | 0.032994923857868 | 0.05962732919254663 | 0.04364694471386996 | 0.0363636363636364 | 0.0320987654320988 | 0.047029702970296995 | 0.0532687651331719 | 0.039419087136929515 | 0.030952380952381 | 0.04639175257731959 | 0.044034090909090925 | 0.05623100303951371 | 0.05757575757575758 | 0.03448275862068972 |
| 0.18000000000000005 | 0.0 | 0.01714285714285711 | 0.05082742316784873 | 0.03389830508474581 | 0.04971098265895951 | 0.02662037037037041 | 0.015151515151515204 | 0.013850415512465405 | 0.044642857142857095 | 0.0409041980624327 | 0.035307517084282515 | 0.02469135802469141 | 0.03963011889035671 | 0.0357142857142857 | 0.03745318352059932 | 0.040609137055837616 | 0.028571428571428602 | 0.0358874878758487 | 0.018181818181818205 | 0.02962962962962961 | 0.0420792079207921 | 0.048426150121065395 | 0.0435684647302905 | 0.05 | 0.0446735395189003 | 0.04261363636363644 | 0.033434650455927 | 0.0363636363636364 | 0.013262599469496004 |
| 0.2 | 0.0 | 0.01714285714285711 | 0.024822695035460998 | 0.01977401129943501 | 0.05202312138728321 | 0.02083333333333331 | 0.015151515151515204 | 0.0221606648199446 | 0.0327380952380952 | 0.032292787944025805 | 0.0341685649202733 | 0.02880658436213989 | 0.03698811096433291 | 0.034013605442176915 | 0.02621722846441952 | 0.0304568527918782 | 0.0347826086956522 | 0.04849660523763343 | 0.024242424242424197 | 0.022222222222222202 | 0.0495049504950495 | 0.029055690072639202 | 0.03734439834024901 | 0.0380952380952381 | 0.042955326460481086 | 0.0284090909090909 | 0.0349544072948328 | 0.048484848484848485 | 0.026525198938992 |
| 0.22 | 0.0 | 0.03428571428571432 | 0.027186761229314408 | 0.02542372881355931 | 0.020809248554913323 | 0.010416666666666699 | 0.0 | 0.0221606648199446 | 0.0357142857142857 | 0.030139935414424123 | 0.031890660592255114 | 0.020576131687242802 | 0.025099075297225906 | 0.04591836734693882 | 0.018726591760299605 | 0.0228426395939086 | 0.0322981366459627 | 0.026188166828322007 | 0.012121212121212099 | 0.022222222222222202 | 0.0371287128712871 | 0.036319612590799015 | 0.0477178423236514 | 0.047619047619047616 | 0.0395189003436426 | 0.02698863636363639 | 0.02279635258358661 | 0.05757575757575758 | 0.013262599469496004 |
| 0.24000000000000005 | 0.0 | 0.01714285714285711 | 0.027186761229314408 | 0.016949152542372906 | 0.020809248554913323 | 0.00810185185185185 | 0.015151515151515204 | 0.013850415512465405 | 0.0357142857142857 | 0.031216361679225018 | 0.028473804100227807 | 0.00823045267489712 | 0.025099075297225906 | 0.030612244897959207 | 0.0112359550561798 | 0.0177664974619289 | 0.01118012422360251 | 0.0271580989330747 | 0.0303030303030303 | 0.00740740740740741 | 0.032178217821782214 | 0.026634382566586012 | 0.03319502074688799 | 0.0380952380952381 | 0.02577319587628871 | 0.03551136363636361 | 0.016717325227963507 | 0.048484848484848485 | 0.010610079575596799 |
| 0.26 | 0.0 | 0.01714285714285711 | 0.0177304964539007 | 0.0127118644067797 | 0.025433526011560705 | 0.004629629629629632 | 0.0 | 0.0277008310249307 | 0.02827380952380952 | 0.025834230355220717 | 0.026195899772209607 | 0.00411522633744856 | 0.013210039630118905 | 0.020408163265306107 | 0.0149812734082397 | 0.0101522842639594 | 0.0161490683229814 | 0.025218234723569315 | 0.018181818181818205 | 0.017283950617283907 | 0.0346534653465347 | 0.041162227602905624 | 0.02282157676348551 | 0.04285714285714291 | 0.0309278350515464 | 0.01988636363636361 | 0.013677811550152 | 0.0393939393939394 | 0.0185676392572944 |
| 0.28000000000000008 | 0.0 | 0.01714285714285711 | 0.023640661938534285 | 0.0112994350282486 | 0.013872832369942205 | 0.00578703703703704 | 0.015151515151515204 | 0.002770083102493071 | 0.02083333333333331 | 0.0129171151776103 | 0.022779043280182206 | 0.016460905349794205 | 0.00660501981505945 | 0.017006802721088402 | 0.018726591760299605 | 0.0304568527918782 | 0.007453416149068322 | 0.026188166828322007 | 0.0 | 0.002469135802469141 | 0.022277227722772325 | 0.014527845036319601 | 0.03319502074688799 | 0.045238095238095216 | 0.027491408934707907 | 0.01988636363636361 | 0.027355623100304 | 0.0424242424242424 | 0.0185676392572944 |
| 0.3000000000000001 | 0.02083333333333331 | 0.00571428571428571 | 0.014184397163120598 | 0.0028248587570621512 | 0.0104046242774566 | 0.002314814814814809 | 0.0 | 0.008310249307479227 | 0.019345238095238106 | 0.0129171151776103 | 0.007972665148063784 | 0.00823045267489712 | 0.010568031704095104 | 0.018707482993197303 | 0.007490636704119854 | 0.002538071065989851 | 0.0173913043478261 | 0.015518913676042698 | 0.00606060606060606 | 0.012345679012345704 | 0.032178217821782214 | 0.021791767554479417 | 0.020746887966805006 | 0.0571428571428571 | 0.03264604810996562 | 0.01988636363636361 | 0.016717325227963507 | 0.0303030303030303 | 0.021220159151193598 |
| 0.32000000000000012 | 0.02083333333333331 | 0.00571428571428571 | 0.0130023640661939 | 0.004237288135593224 | 0.008092485549132958 | 0.002314814814814809 | 0.015151515151515204 | 0.011080332409972301 | 0.011904761904761904 | 0.009687836383207754 | 0.011389521640091107 | 0.020576131687242802 | 0.005284015852047558 | 0.018707482993197303 | 0.0149812734082397 | 0.0126903553299492 | 0.0161490683229814 | 0.016488845780795305 | 0.0 | 0.002469135802469141 | 0.027227722772277214 | 0.021791767554479417 | 0.020746887966805006 | 0.0357142857142857 | 0.015463917525773198 | 0.017045454545454503 | 0.027355623100304 | 0.015151515151515204 | 0.007957559681697613 |
| 0.34 | 0.0 | 0.00571428571428571 | 0.007092198581560282 | 0.0 | 0.0092485549132948 | 0.0 | 0.0 | 0.005540166204986152 | 0.013392857142857107 | 0.003229278794402581 | 0.00341685649202733 | 0.020576131687242802 | 0.00264200792602378 | 0.015306122448979604 | 0.007490636704119854 | 0.007614213197969542 | 0.01118012422360251 | 0.010669253152279299 | 0.0 | 0.002469135802469141 | 0.0173267326732673 | 0.00726392251815981 | 0.039419087136929515 | 0.0333333333333333 | 0.015463917525773198 | 0.017045454545454503 | 0.033434650455927 | 0.027272727272727313 | 0.00530503978779841 |
| 0.3600000000000001 | 0.0 | 0.00571428571428571 | 0.01182033096926711 | 0.00141242937853107 | 0.004624277456647398 | 0.0034722222222222207 | 0.0 | 0.0 | 0.0074404761904761935 | 0.009687836383207754 | 0.011389521640091107 | 0.012345679012345704 | 0.005284015852047558 | 0.0051020408163265285 | 0.007490636704119854 | 0.005076142131979704 | 0.01118012422360251 | 0.0126091173617847 | 0.0 | 0.00740740740740741 | 0.014851485148514901 | 0.009685230024213084 | 0.02282157676348551 | 0.030952380952381 | 0.0120274914089347 | 0.014204545454545501 | 0.024316109422492398 | 0.0393939393939394 | 0.00530503978779841 |
| 0.38000000000000012 | 0.0 | 0.011428571428571404 | 0.0011820330969267109 | 0.0 | 0.003468208092485551 | 0.0 | 0.0 | 0.005540166204986152 | 0.013392857142857107 | 0.00753498385360603 | 0.00341685649202733 | 0.00411522633744856 | 0.005284015852047558 | 0.008503401360544225 | 0.0112359550561798 | 0.0101522842639594 | 0.008695652173913045 | 0.007759456838021342 | 0.0 | 0.002469135802469141 | 0.0173267326732673 | 0.009685230024213084 | 0.020746887966805006 | 0.028571428571428602 | 0.010309278350515504 | 0.022727272727272717 | 0.016717325227963507 | 0.0212121212121212 | 0.0026525198938991998 |
| 0.4 | 0.0 | 0.0 | 0.007092198581560282 | 0.0 | 0.003468208092485551 | 0.002314814814814809 | 0.0 | 0.002770083102493071 | 0.008928571428571423 | 0.002152852529601719 | 0.007972665148063784 | 0.00411522633744856 | 0.00264200792602378 | 0.018707482993197303 | 0.0112359550561798 | 0.0 | 0.006211180124223604 | 0.006789524733268674 | 0.0 | 0.004938271604938274 | 0.0074257425742574315 | 0.002421307506053271 | 0.02282157676348551 | 0.019047619047619008 | 0.00859106529209622 | 0.012784090909090898 | 0.016717325227963507 | 0.012121212121212099 | 0.0 |
### Chart: cat-2 A1
| Category | cat-2(CB1112)_A1_01 | cat-2(CB1112)_A1_02 | cat-2(CB1112)_A1_03 | cat-2(CB1112)_A1_04 | cat-2(CB1112)_A1_05 | cat-2(CB1112)_A1_06 | cat-2(CB1112)_A1_07 | cat-2(CB1112)_A1_08 | cat-2(CB1112)_A1_09 | cat-2(CB1112)_A1_10 | cat-2(CB1112)_A1_11 | cat-2(CB1112)_A1_12 | cat-2(CB1112)_A1_13 | cat-2(CB1112)_A1_14 | cat-2(CB1112)_A1_15 | cat-2(CB1112)_A1_16 | cat-2(CB1112)_A1_17 | cat-2(CB1112)_A1_18 | cat-2(CB1112)_A1_19 | cat-2(CB1112)_A1_20 |
|---|---|---|---|---|---|---|---|---|---|---|---|---|---|---|---|---|---|---|---|---|
| 0 | None | None | None | None | None | None | None | None | None | None | None | None | None | None | None | None | None | None | None | None |
| 2.0000000000000007E-2 | 0.06491499227202471 | 0.07077205882352942 | 0.0651277823577906 | 0.06062819576333088 | 0.0671768707482993 | 0.0617283950617284 | 0.06614173228346455 | 0.0765832106038292 | 0.0517363571934798 | 0.055124223602484486 | 0.0625 | 0.05482625482625482 | 0.07000000000000002 | 0.0732628398791541 | 0.06319947333772223 | 0.063 | 0.0841622035195103 | 0.07344632768361581 | 0.0538793103448276 | 0.04879938032532921 |
| 4.0000000000000015E-2 | 0.08114374034003088 | 0.0716911764705882 | 0.08408903544929934 | 0.0737764791818846 | 0.0663265306122449 | 0.0687830687830688 | 0.0740157480314961 | 0.07142857142857141 | 0.07583274273564852 | 0.0722049689440994 | 0.08779761904761903 | 0.0702702702702703 | 0.09800000000000003 | 0.0898791540785499 | 0.106649111257406 | 0.085 | 0.0971690895179801 | 0.0720338983050847 | 0.08189655172413793 | 0.09062742060418287 |
| 6.0000000000000019E-2 | 0.09119010819165385 | 0.10569852941176502 | 0.09563066776586976 | 0.10007304601899203 | 0.08588435374149665 | 0.0793650793650794 | 0.07322834645669288 | 0.09351988217967606 | 0.10063784549964598 | 0.08928571428571425 | 0.09523809523809525 | 0.0965250965250966 | 0.12066666666666705 | 0.11631419939576995 | 0.12376563528637305 | 0.10500000000000002 | 0.127008416220352 | 0.09887005649717513 | 0.13290229885057506 | 0.08830364058869097 |
| 8.0000000000000029E-2 | 0.112828438948995 | 0.12316176470588205 | 0.118713932399011 | 0.11468224981738502 | 0.108843537414966 | 0.08641975308641968 | 0.11338582677165403 | 0.10162002945508106 | 0.11622962437987203 | 0.0900621118012422 | 0.13616071428571394 | 0.12586872586872597 | 0.10666666666666705 | 0.11782477341389704 | 0.12179065174456906 | 0.12200000000000003 | 0.15761285386381 | 0.10310734463276795 | 0.11853448275862104 | 0.10147172734314502 |
| 0.1 | 0.13214837712519306 | 0.12132352941176502 | 0.12448474855729603 | 0.126369612856099 | 0.14965986394557795 | 0.111111111111111 | 0.13543307086614206 | 0.135493372606775 | 0.148830616583983 | 0.106366459627329 | 0.13020833333333307 | 0.13436293436293406 | 0.17533333333333306 | 0.1555891238670691 | 0.14944042132982208 | 0.165 | 0.14154552410099505 | 0.14759887005649708 | 0.1530172413793101 | 0.12625871417505793 |
| 0.12000000000000002 | 0.08964451313755803 | 0.08731617647058816 | 0.09563066776586976 | 0.09276844411979553 | 0.09523809523809525 | 0.13051146384479706 | 0.08740157480314954 | 0.0883652430044183 | 0.10276399716513103 | 0.09860248447204974 | 0.09449404761904764 | 0.10965250965251003 | 0.10466666666666703 | 0.08534743202416922 | 0.09084924292297558 | 0.12300000000000003 | 0.0627390971690895 | 0.10310734463276795 | 0.10344827586206902 | 0.119287374128582 |
| 0.14000000000000001 | 0.053323029366306014 | 0.05790441176470591 | 0.04534212695795552 | 0.0620891161431702 | 0.0416666666666667 | 0.0634920634920635 | 0.062204724409448825 | 0.0670103092783505 | 0.0517363571934798 | 0.06211180124223603 | 0.0669642857142857 | 0.07567567567567568 | 0.0386666666666667 | 0.04078549848942602 | 0.05595786701777489 | 0.042000000000000016 | 0.055853098699311404 | 0.0720338983050847 | 0.0531609195402299 | 0.06429124709527502 |
| 0.16 | 0.05486862442040194 | 0.04871323529411763 | 0.04534212695795552 | 0.046018991964937916 | 0.04421768707482992 | 0.0529100529100529 | 0.04645669291338582 | 0.05301914580265098 | 0.044649184975194885 | 0.0372670807453416 | 0.046874999999999986 | 0.0324324324324324 | 0.0366666666666667 | 0.049093655589123916 | 0.0368663594470046 | 0.049000000000000016 | 0.053557765876051995 | 0.03884180790960452 | 0.0359195402298851 | 0.03795507358636722 |
| 0.18000000000000005 | 0.053323029366306014 | 0.04871323529411763 | 0.0577081615828524 | 0.054784514243973736 | 0.047619047619047616 | 0.03527336860670193 | 0.0480314960629921 | 0.0390279823269514 | 0.055279943302622286 | 0.04736024844720502 | 0.052083333333333336 | 0.0571428571428571 | 0.04066666666666671 | 0.052114803625377584 | 0.054641211323239014 | 0.048 | 0.03289977046671772 | 0.042372881355932236 | 0.04382183908045978 | 0.05034856700232382 |
| 0.2 | 0.0479134466769706 | 0.05147058823529408 | 0.05605935696619948 | 0.042366691015339755 | 0.045068027210884404 | 0.03880070546737211 | 0.04881889763779532 | 0.04197349042709872 | 0.0588235294117647 | 0.052795031055900644 | 0.052827380952381035 | 0.0308880308880309 | 0.034 | 0.043051359516616296 | 0.0355497037524687 | 0.046 | 0.0390206579954093 | 0.05296610169491532 | 0.048850574712643716 | 0.06738962044926412 |
| 0.22 | 0.0324574961360124 | 0.0330882352941176 | 0.027205276174773328 | 0.048940832724616495 | 0.0357142857142857 | 0.03527336860670193 | 0.04409448818897642 | 0.0346097201767305 | 0.03968816442239551 | 0.04736024844720502 | 0.034226190476190514 | 0.0494208494208494 | 0.02933333333333331 | 0.0309667673716012 | 0.03094140882159321 | 0.033 | 0.02295332823259371 | 0.03742937853107343 | 0.028017241379310314 | 0.0325329202168861 |
| 0.24000000000000005 | 0.028593508500772802 | 0.022058823529411808 | 0.026380873866446802 | 0.027027027027027015 | 0.0416666666666667 | 0.0282186948853616 | 0.0346456692913386 | 0.032400589101620005 | 0.034727143869596 | 0.0481366459627329 | 0.0223214285714286 | 0.0393822393822394 | 0.028 | 0.0354984894259819 | 0.019749835418038215 | 0.022 | 0.01606732976281561 | 0.031073446327683614 | 0.03232758620689659 | 0.028659953524399713 |
| 0.26 | 0.033230293663060316 | 0.024816176470588206 | 0.023083264633140997 | 0.02483564645726812 | 0.021258503401360505 | 0.040564373897707215 | 0.022047244094488206 | 0.03387334315169371 | 0.0191353649893692 | 0.0395962732919255 | 0.0230654761904762 | 0.0223938223938224 | 0.0206666666666667 | 0.021148036253776398 | 0.019749835418038215 | 0.023 | 0.014537107880642698 | 0.023305084745762684 | 0.0179597701149425 | 0.030983733539891607 |
| 0.28000000000000008 | 0.012364760432766601 | 0.0183823529411765 | 0.021434460016488 | 0.015339663988312598 | 0.021258503401360505 | 0.0299823633156966 | 0.026771653543307107 | 0.02209131075110462 | 0.011339475549255807 | 0.025621118012422412 | 0.0126488095238095 | 0.017760617760617808 | 0.018 | 0.010574018126888199 | 0.011849901250822904 | 0.009000000000000003 | 0.0038255547054322912 | 0.02048022598870061 | 0.0186781609195402 | 0.0209140201394268 |
| 0.3000000000000001 | 0.016228748068006203 | 0.0128676470588235 | 0.021434460016488 | 0.01826150474799121 | 0.015306122448979604 | 0.019400352733686108 | 0.0181102362204724 | 0.0169366715758468 | 0.012756909992912801 | 0.0170807453416149 | 0.008184523809523813 | 0.0131274131274131 | 0.010000000000000004 | 0.01586102719033231 | 0.010533245556286996 | 0.008000000000000005 | 0.009946442234123958 | 0.0148305084745763 | 0.010775862068965504 | 0.0247869868319132 |
| 0.32000000000000012 | 0.009273570324574964 | 0.015625 | 0.014839241549876299 | 0.016800584368151912 | 0.016156462585034 | 0.021164021164021198 | 0.021259842519685018 | 0.009572901325478653 | 0.011339475549255807 | 0.015527950310559004 | 0.008184523809523813 | 0.0138996138996139 | 0.005333333333333334 | 0.0037764350453172216 | 0.00921658986175115 | 0.010000000000000004 | 0.0068859984697781235 | 0.0120056497175141 | 0.0114942528735632 | 0.0131680867544539 |
| 0.34 | 0.00772797527047913 | 0.00735294117647059 | 0.00659521846661171 | 0.0131482834185537 | 0.011904761904761904 | 0.0105820105820106 | 0.0055118110236220515 | 0.009572901325478653 | 0.00850460666194188 | 0.0093167701863354 | 0.005208333333333334 | 0.004633204633204634 | 0.008666666666666677 | 0.0037764350453172216 | 0.003949967083607641 | 0.005000000000000002 | 0.00612088752869166 | 0.0070621468926553715 | 0.008620689655172415 | 0.003872966692486441 |
| 0.3600000000000001 | 0.003863987635239571 | 0.0055147058823529415 | 0.00329760923330585 | 0.00511322132943755 | 0.009353741496598648 | 0.007054673721340392 | 0.0094488188976378 | 0.00883652430044183 | 0.003543586109142452 | 0.0093167701863354 | 0.00595238095238095 | 0.0038610038610038602 | 0.003333333333333331 | 0.0037764350453172216 | 0.001974983541803822 | 0.004000000000000002 | 0.0038255547054322912 | 0.002118644067796611 | 0.0014367816091954001 | 0.0015491866769945801 |
| 0.38000000000000012 | 0.006955177743431224 | 0.0009191176470588243 | 0.00247320692497939 | 0.004382761139517901 | 0.0042517006802721136 | 0.0141093474426808 | 0.007874015748031498 | 0.00515463917525773 | 0.0028348688873139597 | 0.006211180124223604 | 0.002976190476190481 | 0.0038610038610038602 | 0.002666666666666671 | 0.0 | 0.001974983541803822 | 0.005000000000000002 | 0.00153022188217292 | 0.00141242937853107 | 0.0007183908045977012 | 0.002323780015491871 |
| 0.4 | 0.00463678516228748 | 0.0055147058823529415 | 0.00659521846661171 | 0.0014609203798393 | 0.0042517006802721136 | 0.007054673721340392 | 0.003149606299212601 | 0.00515463917525773 | 0.003543586109142452 | 0.007763975155279502 | 0.00223214285714286 | 0.0007722007722007722 | 0.0006666666666666672 | 0.0022658610271903312 | 0.0032916392363397007 | 0.002000000000000001 | 0.00153022188217292 | 0.00141242937853107 | 0.0007183908045977012 | 0.0030983733539891602 |
### Chart: cat-2 A3
| Category | cat-2(CB1112)_A3_01 | cat-2(CB1112)_A3_02 | cat-2(CB1112)_A3_04 | cat-2(CB1112)_A3_05 | cat-2(CB1112)_A3_06 | cat-2(CB1112)_A3_07 | cat-2(CB1112)_A3_08 | cat-2(CB1112)_A3_09 | cat-2(CB1112)_A3_10 | cat-2(CB1112)_A3_11 | cat-2(CB1112)_A3_12 | cat-2(CB1112)_A3_13 | cat-2(CB1112)_A3_14 | cat-2(CB1112)_A3_15 | cat-2(CB1112)_A3_16 | cat-2(CB1112)_A3_17 | cat-2(CB1112)_A3_18 | cat-2(CB1112)_A3_19 | cat-2(CB1112)_A3_20 |
|---|---|---|---|---|---|---|---|---|---|---|---|---|---|---|---|---|---|---|---|
| 0 | None | None | None | None | None | None | None | None | None | None | None | None | None | None | None | None | None | None | None |
| 2.0000000000000007E-2 | 0.0729483282674772 | 0.08201892744479498 | 0.0554913294797688 | 0.0875331564986737 | 0.068678459937565 | 0.07049608355091384 | 0.0814873417721519 | 0.074 | 0.057692307692307716 | 0.07293233082706774 | 0.07279693486590046 | 0.10443864229765003 | 0.07536764705882355 | 0.09230769230769234 | 0.067238912732475 | 0.06332453825857523 | 0.0655270655270655 | 0.0576388888888889 | 0.0538599640933573 |
| 4.0000000000000015E-2 | 0.10790273556231003 | 0.11356466876971602 | 0.092485549132948 | 0.0875331564986737 | 0.0978147762747138 | 0.08485639686684075 | 0.108386075949367 | 0.09500000000000003 | 0.08230769230769236 | 0.0954887218045112 | 0.09118773946360148 | 0.09399477806788517 | 0.102022058823529 | 0.09743589743589744 | 0.07582260371959941 | 0.09762532981530346 | 0.08831908831908833 | 0.08541666666666672 | 0.07899461400359073 |
| 6.0000000000000019E-2 | 0.10942249240121606 | 0.0993690851735016 | 0.10867052023121408 | 0.0583554376657825 | 0.104058272632674 | 0.08877284595300267 | 0.102848101265823 | 0.115 | 0.12307692307692306 | 0.12932330827067695 | 0.10804597701149403 | 0.08746736292428207 | 0.0955882352941176 | 0.0858974358974359 | 0.13519313304721006 | 0.10290237467018502 | 0.107549857549858 | 0.13055555555555595 | 0.10323159784560103 |
| 8.0000000000000029E-2 | 0.13069908814589706 | 0.12460567823343806 | 0.13179190751445105 | 0.08222811671087528 | 0.10718002081165504 | 0.0992167101827676 | 0.151107594936709 | 0.14600000000000005 | 0.15230769230769206 | 0.154887218045113 | 0.12413793103448302 | 0.13185378590078295 | 0.125 | 0.09358974358974365 | 0.106580829756795 | 0.11697449428320103 | 0.10897435897435903 | 0.10972222222222208 | 0.10323159784560103 |
| 0.1 | 0.0775075987841945 | 0.0772870662460568 | 0.11907514450867106 | 0.08222811671087528 | 0.13215400624349594 | 0.116187989556136 | 0.17009493670886106 | 0.129 | 0.17230769230769205 | 0.18872180451127807 | 0.13793103448275906 | 0.09530026109660569 | 0.13694852941176505 | 0.12179487179487203 | 0.163090128755365 | 0.160070360598065 | 0.16524216524216506 | 0.188888888888889 | 0.140933572710952 |
| 0.12000000000000002 | 0.0486322188449848 | 0.06624605678233438 | 0.04046242774566472 | 0.04244031830238729 | 0.0530697190426639 | 0.049608355091383796 | 0.04351265822784812 | 0.056 | 0.06000000000000002 | 0.06691729323308272 | 0.05440613026819922 | 0.054830287206266336 | 0.04871323529411763 | 0.05897435897435897 | 0.08440629470672388 | 0.051890941072999096 | 0.07478632478632483 | 0.0743055555555556 | 0.0700179533213645 |
| 0.14000000000000001 | 0.06079027355623103 | 0.042586750788643504 | 0.06011560693641624 | 0.046419098143236116 | 0.04474505723204991 | 0.04308093994778072 | 0.0522151898734177 | 0.05300000000000001 | 0.042307692307692324 | 0.04436090225563913 | 0.04061302681992343 | 0.03916449086161879 | 0.05238970588235289 | 0.017948717948717906 | 0.03791130185979971 | 0.051890941072999096 | 0.0534188034188034 | 0.029861111111111106 | 0.0421903052064632 |
| 0.16 | 0.03951367781155021 | 0.042586750788643504 | 0.053179190751445095 | 0.027851458885941607 | 0.0385015608740895 | 0.0483028720626632 | 0.04667721518987338 | 0.047000000000000014 | 0.0492307692307692 | 0.04436090225563913 | 0.04597701149425289 | 0.03916449086161879 | 0.05238970588235289 | 0.044871794871794914 | 0.05221745350500722 | 0.0466138962181179 | 0.06339031339031342 | 0.0388888888888889 | 0.0385996409335727 |
| 0.18000000000000005 | 0.042553191489361715 | 0.03470031545741322 | 0.041618497109826624 | 0.0384615384615385 | 0.04058272632674302 | 0.04699738903394262 | 0.0482594936708861 | 0.034 | 0.05 | 0.0421052631578947 | 0.05517241379310341 | 0.04699738903394262 | 0.05147058823529408 | 0.042307692307692324 | 0.056509298998569386 | 0.03957783641160952 | 0.0441595441595442 | 0.04722222222222222 | 0.0511669658886894 |
| 0.2 | 0.033434650455927 | 0.041009463722397485 | 0.0335260115606936 | 0.0397877984084881 | 0.03225806451612901 | 0.04438642297650135 | 0.04351265822784812 | 0.037 | 0.03692307692307691 | 0.0345864661654135 | 0.04061302681992343 | 0.04046997389033942 | 0.024816176470588206 | 0.0538461538461538 | 0.04291845493562234 | 0.035180299032541794 | 0.0398860398860399 | 0.05 | 0.039497307001795316 |
| 0.22 | 0.031914893617021316 | 0.03943217665615142 | 0.032369942196531803 | 0.04907161803713532 | 0.0468262226847034 | 0.049608355091383796 | 0.0221518987341772 | 0.028 | 0.03461538461538461 | 0.0240601503759399 | 0.03524904214559391 | 0.0365535248041775 | 0.030330882352941197 | 0.0333333333333333 | 0.0357653791130186 | 0.04397537379067717 | 0.02920227920227921 | 0.0319444444444444 | 0.0385996409335727 |
| 0.24000000000000005 | 0.027355623100304 | 0.03627760252365933 | 0.0335260115606936 | 0.04376657824933693 | 0.0343392299687825 | 0.0483028720626632 | 0.022943037974683517 | 0.032000000000000015 | 0.0223076923076923 | 0.019548872180451107 | 0.026819923371647507 | 0.03394255874673629 | 0.029411764705882398 | 0.0243589743589744 | 0.02789699570815452 | 0.0255057167985928 | 0.031339031339031286 | 0.02152777777777781 | 0.04488330341113112 |
| 0.26 | 0.030395136778115506 | 0.03312302839116719 | 0.017341040462427702 | 0.04244031830238729 | 0.021852237252861607 | 0.027415143603133216 | 0.018196202531645594 | 0.019000000000000006 | 0.0207692307692308 | 0.017293233082706798 | 0.014559386973180094 | 0.0365535248041775 | 0.023897058823529414 | 0.0358974358974359 | 0.01931330472103 | 0.021108179419525117 | 0.0227920227920228 | 0.02152777777777781 | 0.02962298025134649 |
| 0.28000000000000008 | 0.013677811550152 | 0.0236593059936909 | 0.015028901734104 | 0.0397877984084881 | 0.026014568158168598 | 0.0182767624020888 | 0.009493670886075953 | 0.017 | 0.010000000000000004 | 0.0045112781954887255 | 0.006130268199233725 | 0.02872062663185379 | 0.015625 | 0.02307692307692311 | 0.0178826895565093 | 0.0228671943711522 | 0.014245014245014204 | 0.01875 | 0.018850987432675003 |
| 0.3000000000000001 | 0.015197568389057801 | 0.015772870662460612 | 0.015028901734104 | 0.0411140583554377 | 0.0145681581685744 | 0.02219321148825072 | 0.007120253164556962 | 0.016000000000000007 | 0.010769230769230803 | 0.003007518796992482 | 0.013026819923371598 | 0.016971279373368106 | 0.0055147058823529415 | 0.0217948717948718 | 0.01645207439198861 | 0.012313104661389601 | 0.01566951566951571 | 0.0159722222222222 | 0.018850987432675003 |
| 0.32000000000000012 | 0.016717325227963507 | 0.0252365930599369 | 0.01618497109826591 | 0.027851458885941607 | 0.01977107180020811 | 0.023498694516971307 | 0.003955696202531652 | 0.006000000000000002 | 0.007692307692307692 | 0.0045112781954887255 | 0.0114942528735632 | 0.011749347258485601 | 0.008272058823529415 | 0.010256410256410303 | 0.005007153075822602 | 0.010554089709762505 | 0.0049857549857549935 | 0.00555555555555556 | 0.0125673249551167 |
| 0.34 | 0.013677811550152 | 0.0015772870662460613 | 0.01618497109826591 | 0.0331564986737401 | 0.011446409989594201 | 0.010443864229765006 | 0.002373417721518991 | 0.010000000000000004 | 0.005384615384615382 | 0.0007518796992481203 | 0.003831417624521071 | 0.007832898172323764 | 0.0101102941176471 | 0.014102564102564101 | 0.004291845493562234 | 0.012313104661389601 | 0.003561253561253561 | 0.006250000000000002 | 0.010771992818671498 |
| 0.3600000000000001 | 0.010638297872340394 | 0.012618296529968496 | 0.006936416184971107 | 0.017241379310344803 | 0.008324661810613945 | 0.0156657963446475 | 0.003955696202531652 | 0.004000000000000002 | 0.004615384615384622 | 0.002255639097744361 | 0.013793103448275905 | 0.0 | 0.014705882352941199 | 0.014102564102564101 | 0.0035765379113018602 | 0.0070360598065083635 | 0.003561253561253561 | 0.00763888888888889 | 0.004488330341113112 |
| 0.38000000000000012 | 0.010638297872340394 | 0.0015772870662460613 | 0.008092485549132958 | 0.00397877984084881 | 0.010405827263267413 | 0.005221932114882512 | 0.003164556962025322 | 0.006000000000000002 | 0.003076923076923081 | 0.003759398496240601 | 0.006896551724137932 | 0.0 | 0.009191176470588244 | 0.012820512820512801 | 0.0035765379113018602 | 0.0052770448548812715 | 0.004273504273504271 | 0.002083333333333331 | 0.006283662477558354 |
| 0.4 | 0.003039513677811552 | 0.0031545741324921117 | 0.005780346820809252 | 0.00397877984084881 | 0.0 | 0.002610966057441251 | 0.0015822784810126606 | 0.002000000000000001 | 0.0015384615384615408 | 0.0 | 0.006896551724137932 | 0.007832898172323764 | 0.002757352941176472 | 0.006410256410256412 | 0.004291845493562234 | 0.0017590149516270904 | 0.0 | 0.002083333333333331 | 0.006283662477558354 |
### Chart: cat-2 A5
| Category | cat-2(CB1112)_A5_01 | cat-2(CB1112)_A5_02 | cat-2(CB1112)_A5_03 | cat-2(CB1112)_A5_04 | cat-2(CB1112)_A5_05 | cat-2(CB1112)_A5_06 | cat-2(CB1112)_A5_07 | cat-2(CB1112)_A5_08 | cat-2(CB1112)_A5_09 | cat-2(CB1112)_A5_10 | cat-2(CB1112)_A5_11 | cat-2(CB1112)_A5_12 | cat-2(CB1112)_A5_13 | cat-2(CB1112)_A5_14 | cat-2(CB1112)_A5_15 | cat-2(CB1112)_A5_16 | cat-2(CB1112)_A5_17 | cat-2(CB1112)_A5_18 | cat-2(CB1112)_A5_19 | cat-2(CB1112)_A5_20 |
|---|---|---|---|---|---|---|---|---|---|---|---|---|---|---|---|---|---|---|---|---|
| 0 | None | None | None | None | None | None | None | None | None | None | None | None | None | None | None | None | None | None | None | None |
| 2.0000000000000007E-2 | 0.0699658703071672 | 0.086038961038961 | 0.07135016465422611 | 0.09555854643337823 | 0.0954616588419405 | 0.0829493087557604 | 0.09900990099009907 | 0.07645875251509049 | 0.07089947089947093 | 0.062624254473161 | 0.0735411670663469 | 0.08695652173913045 | 0.06435643564356443 | 0.07416563658838073 | 0.05639097744360902 | 0.07387862796833773 | 0.0656851642129105 | 0.0665163472378805 | 0.07708333333333334 | 0.057860262008733634 |
| 4.0000000000000015E-2 | 0.08788395904436856 | 0.08116883116883122 | 0.10428100987925402 | 0.08479138627187084 | 0.0704225352112676 | 0.0864055299539171 | 0.0858085808580858 | 0.06438631790744476 | 0.091005291005291 | 0.09244532803180916 | 0.11350919264588298 | 0.11594202898550703 | 0.102970297029703 | 0.0988875154511743 | 0.07142857142857141 | 0.06332453825857523 | 0.11098527746319403 | 0.10597519729425002 | 0.101041666666667 | 0.07969432314410482 |
| 6.0000000000000019E-2 | 0.10068259385665503 | 0.07142857142857141 | 0.10976948408342503 | 0.10767160161507403 | 0.07355242566510171 | 0.09216589861751152 | 0.10891089108910898 | 0.06841046277666 | 0.10158730158730198 | 0.10039761431411498 | 0.12230215827338105 | 0.10276679841897206 | 0.110891089108911 | 0.0667490729295426 | 0.07894736842105257 | 0.0580474934036939 | 0.12344280860702198 | 0.0744081172491545 | 0.108333333333333 | 0.0971615720524018 |
| 8.0000000000000029E-2 | 0.14505119453924906 | 0.10064935064935097 | 0.106476399560922 | 0.12516823687752404 | 0.100156494522692 | 0.11290322580645203 | 0.0726072607260726 | 0.0503018108651912 | 0.08888888888888893 | 0.11133200795228602 | 0.16306954436450793 | 0.13702239789196308 | 0.164356435643564 | 0.111248454882571 | 0.07518796992481203 | 0.08970976253298153 | 0.14496036240090607 | 0.12739571589628 | 0.101041666666667 | 0.13755458515283805 |
| 0.1 | 0.12883959044368595 | 0.08766233766233769 | 0.147091108671789 | 0.09825033647375503 | 0.111111111111111 | 0.12903225806451593 | 0.0858085808580858 | 0.10060362173038204 | 0.13015873015873 | 0.145129224652087 | 0.128697042366107 | 0.10935441370224 | 0.133663366336634 | 0.11372064276885005 | 0.06015037593984962 | 0.050131926121372 | 0.12797281993204995 | 0.13979706877113907 | 0.16458333333333305 | 0.13537117903930093 |
| 0.12000000000000002 | 0.052901023890785014 | 0.051948051948052 | 0.0406147091108672 | 0.05787348586810232 | 0.03286384976525821 | 0.06566820276497698 | 0.0495049504950495 | 0.0583501006036217 | 0.0666666666666667 | 0.05566600397614312 | 0.056754596322941726 | 0.0816864295125165 | 0.06237623762376242 | 0.040791100123609404 | 0.041353383458646635 | 0.0580474934036939 | 0.0656851642129105 | 0.04847801578353999 | 0.06875000000000002 | 0.0524017467248908 |
| 0.14000000000000001 | 0.040102389078498314 | 0.030844155844155802 | 0.0307354555433589 | 0.05114401076716017 | 0.03286384976525821 | 0.0449308755760369 | 0.0363036303630363 | 0.0261569416498994 | 0.0412698412698413 | 0.03777335984095431 | 0.059952038369304614 | 0.04216073781291174 | 0.047524752475247484 | 0.030902348578492014 | 0.04511278195488724 | 0.0343007915567282 | 0.06002265005662513 | 0.0439684329199549 | 0.037500000000000006 | 0.057860262008733634 |
| 0.16 | 0.04436860068259392 | 0.038961038961039 | 0.0570801317233809 | 0.047106325706594884 | 0.04381846635367762 | 0.05645161290322582 | 0.033003300330033 | 0.038229376257545314 | 0.047619047619047616 | 0.0526838966202783 | 0.06155075939248598 | 0.03557312252964431 | 0.044554455445544615 | 0.0482076637824475 | 0.037593984962406 | 0.03957783641160952 | 0.061155152887882175 | 0.040586245772266084 | 0.04062499999999999 | 0.03930131004366812 |
| 0.18000000000000005 | 0.04351535836177466 | 0.019480519480519508 | 0.0515916575192097 | 0.03499327052489913 | 0.03599374021909231 | 0.03225806451612901 | 0.03960396039603961 | 0.038229376257545314 | 0.035978835978836006 | 0.02982107355864811 | 0.037569944044764214 | 0.04084321475625822 | 0.04356435643564362 | 0.04449938195302845 | 0.037593984962406 | 0.0580474934036939 | 0.04869762174405441 | 0.0383314543404735 | 0.04895833333333332 | 0.049126637554585136 |
| 0.2 | 0.04095563139931742 | 0.0373376623376623 | 0.026344676180022 | 0.022880215343203218 | 0.0469483568075117 | 0.0449308755760369 | 0.04290429042904292 | 0.02213279678068411 | 0.024338624338624288 | 0.044731610337972225 | 0.028776978417266213 | 0.026350461133069797 | 0.029702970297029712 | 0.03955500618046971 | 0.033834586466165405 | 0.0527704485488127 | 0.02944507361268401 | 0.04171364148816231 | 0.04062499999999999 | 0.03056768558951961 |
| 0.22 | 0.0383959044368601 | 0.0324675324675325 | 0.020856201975850707 | 0.02153432032301481 | 0.02347417840375591 | 0.026497695852534607 | 0.033003300330033 | 0.0442655935613682 | 0.02962962962962961 | 0.03280318091451291 | 0.017585931254996003 | 0.034255599472990804 | 0.01980198019801981 | 0.034610630407911014 | 0.030075187969924817 | 0.03957783641160952 | 0.02378255945639859 | 0.03269447576099211 | 0.04062499999999999 | 0.0316593886462882 |
| 0.24000000000000005 | 0.03242320819112631 | 0.019480519480519508 | 0.02414928649835351 | 0.0309555854643338 | 0.034428794992175285 | 0.0357142857142857 | 0.029702970297029712 | 0.0422535211267606 | 0.02751322751322752 | 0.0228628230616302 | 0.023181454836131078 | 0.02503293807641632 | 0.0207920792079208 | 0.040791100123609404 | 0.030075187969924817 | 0.0184696569920844 | 0.0192525481313703 | 0.025930101465614412 | 0.021875000000000006 | 0.021834061135371202 |
| 0.26 | 0.023890784982935197 | 0.025974025974026007 | 0.02414928649835351 | 0.022880215343203218 | 0.037558685446009404 | 0.031105990783410118 | 0.03960396039603961 | 0.048289738430583484 | 0.02962962962962961 | 0.0387673956262425 | 0.0111910471622702 | 0.019762845849802414 | 0.017821782178217803 | 0.030902348578492014 | 0.026315789473684202 | 0.013192612137203198 | 0.011325028312570805 | 0.0383314543404735 | 0.0125 | 0.0240174672489083 |
| 0.28000000000000008 | 0.009385665529010248 | 0.02922077922077921 | 0.0285400658616905 | 0.020188425302826392 | 0.03286384976525821 | 0.016129032258064505 | 0.033003300330033 | 0.046277665995975895 | 0.021164021164021198 | 0.021868787276342 | 0.012789768185451598 | 0.019762845849802414 | 0.009900990099009906 | 0.0358467243510507 | 0.026315789473684202 | 0.021108179419525117 | 0.011325028312570805 | 0.024802705749718202 | 0.02083333333333331 | 0.019650655021834103 |
| 0.3000000000000001 | 0.017064846416382305 | 0.0340909090909091 | 0.017563117453348 | 0.010767160161507404 | 0.0219092331768388 | 0.013824884792626705 | 0.04290429042904292 | 0.030181086519114705 | 0.02751322751322752 | 0.011928429423459204 | 0.007993605115907274 | 0.013175230566534898 | 0.013861386138613901 | 0.018541409147095213 | 0.04511278195488724 | 0.021108179419525117 | 0.009060022650056634 | 0.0146561443066516 | 0.010416666666666699 | 0.021834061135371202 |
| 0.32000000000000012 | 0.008532423208191134 | 0.019480519480519508 | 0.021953896816685 | 0.0134589502018843 | 0.0250391236306729 | 0.011520737327188904 | 0.009900990099009906 | 0.028169014084506998 | 0.021164021164021198 | 0.012922465208747506 | 0.004796163069544364 | 0.00790513833992095 | 0.004950495049504951 | 0.02101359703337451 | 0.0488721804511278 | 0.023746701846965694 | 0.004530011325028312 | 0.0146561443066516 | 0.011458333333333301 | 0.0152838427947598 |
| 0.34 | 0.009385665529010248 | 0.0340909090909091 | 0.013172338090011 | 0.014804845222072701 | 0.026604068857590008 | 0.005760368663594474 | 0.02640264026402641 | 0.024144869215291694 | 0.012698412698412704 | 0.010934393638171 | 0.004796163069544364 | 0.009222661396574443 | 0.00594059405940594 | 0.014833127317676104 | 0.033834586466165405 | 0.0343007915567282 | 0.00339750849377123 | 0.010146561443066507 | 0.006250000000000002 | 0.0141921397379913 |
| 0.3600000000000001 | 0.006825938566552898 | 0.0162337662337662 | 0.010976948408342499 | 0.0134589502018843 | 0.009389671361502353 | 0.013824884792626705 | 0.016501650165016507 | 0.0321931589537223 | 0.0137566137566138 | 0.007952286282306164 | 0.0031974420463629118 | 0.010540184453227904 | 0.002970297029702971 | 0.007416563658838074 | 0.033834586466165405 | 0.0263852242744063 | 0.004530011325028312 | 0.00450958286358512 | 0.003125000000000001 | 0.009825327510917034 |
| 0.38000000000000012 | 0.004266211604095562 | 0.011363636363636404 | 0.008781558726673978 | 0.014804845222072701 | 0.0219092331768388 | 0.006912442396313365 | 0.009900990099009906 | 0.02213279678068411 | 0.006349206349206352 | 0.007952286282306164 | 0.0007993605115907275 | 0.00131752305665349 | 0.004950495049504951 | 0.006180469715698394 | 0.033834586466165405 | 0.0158311345646438 | 0.00339750849377123 | 0.013528748590755401 | 0.00416666666666667 | 0.007641921397379911 |
| 0.4 | 0.005972696245733792 | 0.00974025974025974 | 0.003293084522502742 | 0.0067294751009421335 | 0.012519561815336503 | 0.00921658986175115 | 0.009900990099009906 | 0.014084507042253504 | 0.002116402116402119 | 0.008946322067594428 | 0.0015987210231814505 | 0.00131752305665349 | 0.000990099009900991 | 0.006180469715698394 | 0.030075187969924817 | 0.0158311345646438 | 0.00339750849377123 | 0.0022547914317925617 | 0.002083333333333331 | 0.0010917030567685604 |
