# Supplementary material for: Evaluation of Head Movement Periodicity and Irregularity during Locomotion of Caenorhabditis elegans
Source: Front Behav Neurosci. 2013 Mar 21;7:20. doi: 10.3389/fnbeh.2013.00020 (PMC3604732; doi:10.3389/fnbeh.2013.00020)
Supplement: Supplementary Figure S3 — nSL-2 histograms (50%) for each worms. [file 42064_Shingai_Presentation3.PPTX]

## Slide 1
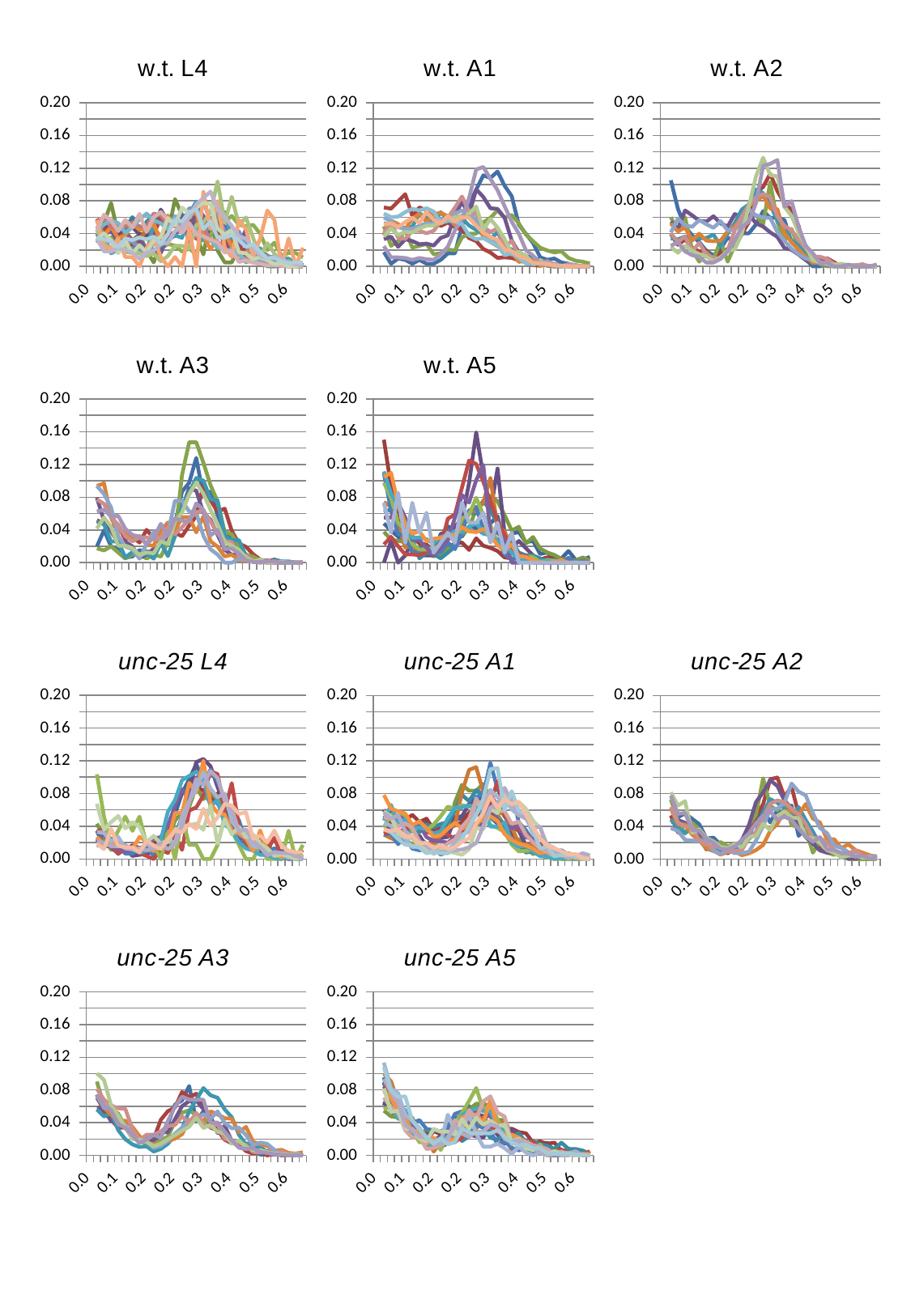

### Chart: w.t. L4
| Category | N2_L4_0601 | N2_L4_0602 | N2_L4_0603 | N2_L4_0604 | N2_L4_0605 | N2_L4_0606 | N2_L4_0607 | N2_L4_0608 | N2_L4_0609 | N2_L4_0611 | N2_L4_0612 | N2_L4_0613 | N2_L4_0614 | N2_L4_0615 | N2_L4_0616 | N2_L4_0617 | N2_L4_0618 | N2_L4_0619 | N2_L4_0621 | N2_L4_0622 | N2_L4_0623 | N2_L4_0624 | N2_L4_0625 |
|---|---|---|---|---|---|---|---|---|---|---|---|---|---|---|---|---|---|---|---|---|---|---|---|
| 0 | None | None | None | None | None | None | None | None | None | None | None | None | None | None | None | None | None | None | None | None | None | None | None |
| 2.0000000000000007E-2 | None | 0.0429268292682927 | 0.05314009661835748 | 0.0379888268156425 | 0.04651162790697674 | 0.0393096836049856 | None | 0.0581113801452785 | 0.056159420289855086 | 0.0286885245901639 | 0.04113924050632908 | 0.031034482758620714 | 0.049549549549549585 | 0.0528010302640052 | 0.0440251572327044 | None | 0.043936731107205626 | 0.05681818181818183 | None | 0.043112513144058916 | 0.03304597701149433 | 0.03389830508474581 | 0.0341614906832298 |
| 4.0000000000000015E-2 | None | 0.05073170731707323 | 0.05314009661835748 | 0.041340782122904984 | 0.04392764857881139 | 0.026845637583892617 | None | 0.0435835351089588 | 0.036231884057971016 | 0.03688524590163932 | 0.025316455696202493 | 0.03793103448275861 | 0.0371621621621622 | 0.059884095299420514 | 0.028301886792452793 | None | 0.0509666080843585 | 0.022727272727272717 | None | 0.06309148264984232 | 0.0402298850574713 | 0.018252933507170797 | 0.033126293995859195 |
| 6.0000000000000019E-2 | None | 0.04390243902439023 | 0.07729468599033824 | 0.0558659217877095 | 0.04134366925064601 | 0.035474592521572416 | None | 0.048426150121065395 | 0.0344202898550725 | 0.045081967213114804 | 0.031645569620253215 | 0.04827586206896546 | 0.015765765765765806 | 0.0476497102382486 | 0.0188679245283019 | None | 0.056239015817223216 | 0.022727272727272717 | None | 0.0557308096740273 | 0.031609195402298916 | 0.018252933507170797 | 0.03002070393374739 |
| 8.0000000000000029E-2 | None | 0.03024390243902442 | 0.03864734299516912 | 0.0379888268156425 | 0.0452196382428941 | 0.03163950143815921 | None | 0.03147699757869253 | 0.0289855072463768 | 0.0286885245901639 | 0.0189873417721519 | 0.03448275862068972 | 0.020270270270270313 | 0.04571796522858981 | 0.03144654088050313 | None | 0.05272407732864683 | 0.0340909090909091 | None | 0.043112513144058916 | 0.0158045977011494 | 0.022164276401564514 | 0.023809523809523808 |
| 0.1 | None | 0.0429268292682927 | 0.0289855072463768 | 0.04469273743016763 | 0.04586563307493543 | 0.04218600191754553 | None | 0.041162227602905624 | 0.018115942028985497 | 0.03688524590163932 | 0.0221518987341772 | 0.03793103448275861 | 0.029279279279279317 | 0.0489375402446877 | 0.022012578616352207 | None | 0.041007615700058585 | 0.011363636363636404 | None | 0.05678233438485802 | 0.031609195402298916 | 0.019556714471968703 | 0.0341614906832298 |
| 0.12000000000000002 | None | 0.03121951219512201 | 0.0289855072463768 | 0.04804469273743022 | 0.060077519379844985 | 0.0287631831255992 | None | 0.021791767554479417 | 0.016304347826087 | 0.0327868852459016 | 0.0221518987341772 | 0.05172413793103452 | 0.030405405405405414 | 0.05344494526722468 | 0.0188679245283019 | None | 0.0474516695957821 | 0.011363636363636404 | None | 0.04731861198738175 | 0.0158045977011494 | 0.016949152542372906 | 0.0320910973084886 |
| 0.14000000000000001 | None | 0.0390243902439024 | 0.03864734299516912 | 0.0391061452513966 | 0.04392764857881139 | 0.0335570469798658 | None | 0.029055690072639202 | 0.01992753623188411 | 0.0327868852459016 | 0.0221518987341772 | 0.04482758620689663 | 0.02364864864864869 | 0.0489375402446877 | 0.0157232704402516 | None | 0.05741066198008203 | 0.0 | None | 0.06414300736067298 | 0.0258620689655172 | 0.009126466753585404 | 0.0351966873706004 |
| 0.16 | None | 0.040000000000000015 | 0.01932367149758451 | 0.03687150837988831 | 0.0542635658914729 | 0.0441035474592522 | None | 0.021791767554479417 | 0.018115942028985497 | 0.0204918032786885 | 0.0158227848101266 | 0.04482758620689663 | 0.014639639639639601 | 0.0476497102382486 | 0.012578616352201295 | None | 0.06444053895723492 | 0.022727272727272717 | None | 0.042060988433228225 | 0.017241379310344803 | 0.016949152542372906 | 0.033126293995859195 |
| 0.18000000000000005 | None | 0.04195121951219508 | 0.0048309178743961385 | 0.046927374301675984 | 0.06330749354005173 | 0.046021093000958795 | None | 0.0387409200968523 | 0.0289855072463768 | 0.0204918032786885 | 0.05379746835443043 | 0.05172413793103452 | 0.029279279279279317 | 0.06439150032195752 | 0.0157232704402516 | None | 0.056239015817223216 | 0.0340909090909091 | None | 0.059936908517350215 | 0.031609195402298916 | 0.019556714471968703 | 0.036231884057971016 |
| 0.2 | None | 0.0575609756097561 | 0.0289855072463768 | 0.0692737430167598 | 0.0542635658914729 | 0.03643336529242572 | None | 0.060532687651331754 | 0.0217391304347826 | 0.0163934426229508 | 0.025316455696202493 | 0.031034482758620714 | 0.0281531531531532 | 0.0624597553122988 | 0.006289308176100634 | None | 0.05272407732864683 | 0.011363636363636404 | None | 0.06624605678233438 | 0.0359195402298851 | 0.027379400260756206 | 0.027950310559006215 |
| 0.22 | None | 0.0390243902439024 | 0.03864734299516912 | 0.05474860335195532 | 0.06136950904392762 | 0.047938638542665404 | None | 0.0387409200968523 | 0.027173913043478312 | 0.03688524590163932 | 0.031645569620253215 | 0.06206896551724142 | 0.029279279279279317 | 0.0502253702511269 | 0.012578616352201295 | None | 0.04510837727006438 | 0.0 | None | 0.05678233438485802 | 0.0589080459770115 | 0.028683181225554105 | 0.039337474120082795 |
| 0.24000000000000005 | None | 0.05951219512195124 | 0.0821256038647343 | 0.04916201117318442 | 0.059431524547803635 | 0.049856184084372014 | None | 0.041162227602905624 | 0.025362318840579712 | 0.03688524590163932 | 0.037974683544303806 | 0.04827586206896546 | 0.04279279279279282 | 0.06117192530585956 | 0.02515723270440251 | None | 0.0609256004686585 | 0.011363636363636404 | None | 0.052576235541535225 | 0.050287356321839095 | 0.043024771838331234 | 0.056935817805383 |
| 0.26 | None | 0.0546341463414634 | 0.0628019323671498 | 0.06033519553072632 | 0.06266149870801031 | 0.057526366251198516 | None | 0.04600484261501207 | 0.018115942028985497 | 0.061475409836065614 | 0.034810126582278514 | 0.05172413793103452 | 0.04729729729729732 | 0.05215711526078558 | 0.02515723270440251 | None | 0.05038078500292911 | 0.0 | None | 0.041009463722397485 | 0.0718390804597701 | 0.04823989569752278 | 0.054865424430641845 |
| 0.28000000000000008 | None | 0.06731707317073175 | 0.0579710144927536 | 0.0424581005586592 | 0.058139534883720916 | 0.0393096836049856 | None | 0.05569007263922522 | 0.0217391304347826 | 0.0286885245901639 | 0.06329113924050633 | 0.06206896551724142 | 0.0709459459459459 | 0.05344494526722468 | 0.022012578616352207 | None | 0.0515524311657879 | 0.0454545454545455 | None | 0.052576235541535225 | 0.06465517241379308 | 0.059973924380704015 | 0.06625258799171843 |
| 0.3000000000000001 | None | 0.06439024390243903 | 0.03864734299516912 | 0.0424581005586592 | 0.05038759689922482 | 0.057526366251198516 | None | 0.03389830508474581 | 0.0144927536231884 | 0.07377049180327874 | 0.07911392405063294 | 0.06551724137931042 | 0.06981981981981981 | 0.035415325177076615 | 0.050314465408805 | None | 0.04276508494434685 | 0.0 | None | 0.0389064143007361 | 0.0718390804597701 | 0.07561929595827903 | 0.06935817805383022 |
| 0.32000000000000012 | None | 0.05560975609756097 | 0.0144927536231884 | 0.05139664804469272 | 0.0381136950904393 | 0.050814956855225336 | None | 0.041162227602905624 | 0.0416666666666667 | 0.061475409836065614 | 0.031645569620253215 | 0.03793103448275861 | 0.0810810810810811 | 0.0489375402446877 | 0.053459119496855285 | None | 0.03632103104862332 | 0.09090909090909094 | None | 0.03680336487907473 | 0.07758620689655173 | 0.0834419817470665 | 0.045548654244306416 |
| 0.34 | None | 0.04195121951219508 | 0.0579710144927536 | 0.0379888268156425 | 0.032299741602067215 | 0.047938638542665404 | None | 0.048426150121065395 | 0.036231884057971016 | 0.045081967213114804 | 0.0569620253164557 | 0.020689655172413814 | 0.052927927927927935 | 0.030907920154539602 | 0.06603773584905663 | None | 0.032220269478617516 | 0.0454545454545455 | None | 0.032597266035751804 | 0.0761494252873563 | 0.09126466753585404 | 0.054865424430641845 |
| 0.3600000000000001 | None | 0.040975609756097583 | 0.01932367149758451 | 0.046927374301675984 | 0.0297157622739018 | 0.049856184084372014 | None | 0.0581113801452785 | 0.06340579710144932 | 0.0491803278688525 | 0.06645569620253163 | 0.03448275862068972 | 0.0630630630630631 | 0.030264005151320002 | 0.10377358490566004 | None | 0.022847100175746912 | 0.0795454545454545 | None | 0.029442691903259707 | 0.04597701149425289 | 0.0704041720990874 | 0.0351966873706004 |
| 0.38000000000000012 | None | 0.029268292682926814 | 0.0048309178743961385 | 0.027932960893854705 | 0.021963824289405708 | 0.03068072866730581 | None | 0.021791767554479417 | 0.0543478260869565 | 0.0491803278688525 | 0.06645569620253163 | 0.0413793103448276 | 0.04279279279279282 | 0.02060528010302641 | 0.053459119496855285 | None | 0.01933216168717051 | 0.022727272727272717 | None | 0.0168243953732913 | 0.03879310344827591 | 0.059973924380704015 | 0.0351966873706004 |
| 0.4 | None | 0.029268292682926814 | 0.0048309178743961385 | 0.017877094972067 | 0.015503875968992208 | 0.0325982742090125 | None | 0.041162227602905624 | 0.0615942028985507 | 0.0122950819672131 | 0.034810126582278514 | 0.024137931034482793 | 0.039414414414414414 | 0.01867353509336771 | 0.08490566037735855 | None | 0.020503807850029313 | 0.011363636363636404 | None | 0.018927444794952706 | 0.0445402298850575 | 0.049543676662320714 | 0.0383022774327122 |
| 0.4200000000000001 | None | 0.0214634146341463 | 0.024154589371980683 | 0.017877094972067 | 0.0148578811369509 | 0.02492809204218599 | None | 0.026634382566586012 | 0.0507246376811594 | 0.024590163934426198 | 0.04113924050632908 | 0.013793103448275905 | 0.04279279279279282 | 0.007083065035415333 | 0.050314465408805 | None | 0.015817223198593997 | 0.05681818181818183 | None | 0.005257623554153524 | 0.017241379310344803 | 0.036505867014341616 | 0.0320910973084886 |
| 0.44 | None | 0.0126829268292683 | 0.0048309178743961385 | 0.021229050279329607 | 0.0103359173126615 | 0.026845637583892617 | None | 0.0121065375302663 | 0.048913043478260886 | 0.045081967213114804 | 0.028481012658227816 | 0.017241379310344803 | 0.02364864864864869 | 0.0103026400515132 | 0.05974842767295602 | None | 0.005272407732864684 | 0.0454545454545455 | None | 0.007360672975814934 | 0.0158045977011494 | 0.03389830508474581 | 0.023809523809523808 |
| 0.46 | None | 0.00585365853658537 | 0.00966183574879227 | 0.0100558659217877 | 0.00904392764857881 | 0.015340364333652904 | None | 0.026634382566586012 | 0.0507246376811594 | 0.00409836065573771 | 0.009493670886075953 | 0.024137931034482793 | 0.0191441441441441 | 0.00965872504829363 | 0.034591194968553514 | None | 0.008201523140011718 | 0.011363636363636404 | None | 0.004206098843322823 | 0.008620689655172415 | 0.022164276401564514 | 0.02691511387163561 |
| 0.48000000000000009 | None | 0.004878048780487803 | 0.0144927536231884 | 0.00335195530726257 | 0.0025839793281653726 | 0.01917545541706621 | None | 0.00726392251815981 | 0.03985507246376811 | 0.0122950819672131 | 0.0221518987341772 | 0.0103448275862069 | 0.013513513513513504 | 0.0051513200257566035 | 0.012578616352201295 | None | 0.00878734622144113 | 0.0340909090909091 | None | 0.0031545741324921117 | 0.0114942528735632 | 0.013037809647979107 | 0.0175983436853002 |
| 0.5 | None | 0.00390243902439024 | 0.0 | 0.00670391061452514 | 0.0006459948320413443 | 0.013422818791946301 | None | 0.0121065375302663 | 0.01992753623188411 | 0.0122950819672131 | 0.009493670886075953 | 0.0034482758620689707 | 0.012387387387387401 | 0.002575660012878301 | 0.028301886792452793 | None | 0.0070298769771529 | 0.0681818181818182 | None | 0.0010515247108306995 | 0.007183908045977011 | 0.009126466753585404 | 0.0093167701863354 |
| 0.52 | None | 0.00585365853658537 | 0.0 | 0.002234636871508381 | 0.0 | 0.00767018216682646 | None | 0.002421307506053271 | 0.0289855072463768 | 0.00819672131147541 | 0.012658227848101299 | 0.0034482758620689707 | 0.005630630630630632 | 0.001931745009658731 | 0.0157232704402516 | None | 0.0023432923257176307 | 0.05681818181818183 | None | 0.0010515247108306995 | 0.0028735632183908015 | 0.011734028683181205 | 0.010351966873705995 |
| 0.54 | None | 0.0019512195121951204 | 0.0048309178743961385 | 0.00335195530726257 | 0.0006459948320413443 | 0.00767018216682646 | None | 0.00484261501210654 | 0.012681159420289901 | 0.00409836065573771 | 0.009493670886075953 | 0.0034482758620689707 | 0.0045045045045045 | 0.0006439150032195752 | 0.003144654088050312 | None | 0.003514938488576452 | 0.0 | None | 0.0010515247108306995 | 0.004310344827586208 | 0.009126466753585404 | 0.006211180124223604 |
| 0.56000000000000005 | None | 0.0009756097560975617 | 0.0048309178743961385 | 0.002234636871508381 | 0.0006459948320413443 | 0.005752636625119852 | None | 0.002421307506053271 | 0.016304347826087 | 0.0 | 0.009493670886075953 | 0.0 | 0.002252252252252251 | 0.0 | 0.006289308176100634 | None | 0.0023432923257176307 | 0.0340909090909091 | None | 0.0 | 0.0 | 0.00391134289439374 | 0.003105590062111802 |
| 0.58000000000000007 | None | 0.0009756097560975617 | 0.0 | 0.00111731843575419 | 0.0006459948320413443 | 0.0019175455417066213 | None | 0.0 | 0.016304347826087 | 0.0 | 0.003164556962025322 | 0.0 | 0.002252252252252251 | 0.0006439150032195752 | 0.0 | None | 0.00175746924428822 | 0.0 | None | 0.0 | 0.0 | 0.002607561929595831 | 0.005175983436853 |
| 0.6000000000000002 | None | 0.0 | 0.0048309178743961385 | 0.0 | 0.0 | 0.000958772770853308 | None | 0.0 | 0.010869565217391306 | 0.00409836065573771 | 0.0 | 0.0034482758620689707 | 0.0 | 0.0 | 0.0 | None | 0.0005858230814294082 | 0.022727272727272717 | None | 0.0010515247108306995 | 0.0028735632183908015 | 0.0 | 0.0041407867494824 |
### Chart: w.t. A1
| Category | N2_A1_40_30_01 | N2_A1_40_30_06 | N2_A1_40_30_07 | N2_REX_A1_01 | N2_REX_A1_03 | N2_REX_A1_05 | N2_REX_A1_07 | N2_REX_A1_08 | N2_REX_A1_09 | N2_REX_A1_10 | N2_REX_A1_11 | N2_REX_A1_12 |
|---|---|---|---|---|---|---|---|---|---|---|---|---|
| 0 | None | None | None | None | None | None | None | None | None | None | None | None |
| 2.0000000000000007E-2 | 0.017460317460317506 | 0.0719530102790015 | 0.0490307867730901 | 0.033153430994602884 | 0.05270723526593202 | 0.0380952380952381 | 0.05955977557186018 | 0.040365575019040416 | 0.0365193868349865 | 0.0247093023255814 | 0.06452714236940939 | 0.05137751303052872 |
| 4.0000000000000015E-2 | 0.00317460317460317 | 0.07048458149779742 | 0.02508551881413912 | 0.03623747108712413 | 0.05126976521322468 | 0.05578231292517012 | 0.0552438498057833 | 0.05331302361005332 | 0.0464382326420198 | 0.010901162790697704 | 0.0600887674974394 | 0.0524944154877141 |
| 6.0000000000000019E-2 | 0.009523809523809523 | 0.079295154185022 | 0.030786773090079808 | 0.02390131071703932 | 0.047915668423574496 | 0.05170068027210882 | 0.047906776003452715 | 0.035034272658035014 | 0.0383228133453562 | 0.010901162790697704 | 0.060771594400819404 | 0.048026805658972486 |
| 8.0000000000000029E-2 | 0.007936507936507941 | 0.08810572687224673 | 0.04446978335233752 | 0.033153430994602884 | 0.046957355055102984 | 0.044897959183673515 | 0.0457488131204143 | 0.033511043412033516 | 0.04192966636609559 | 0.0101744186046512 | 0.0662342096278593 | 0.0539836187639613 |
| 0.1 | 0.00317460317460317 | 0.061674008810572695 | 0.022805017103762818 | 0.030840400925212 | 0.04839482510781032 | 0.058503401360544216 | 0.0457488131204143 | 0.04341203351104343 | 0.04147880973850319 | 0.00799418604651163 | 0.07033117104813932 | 0.05919583023082653 |
| 0.12000000000000002 | 0.007936507936507941 | 0.0719530102790015 | 0.02508551881413912 | 0.026985350809560518 | 0.0646861523718256 | 0.06394557823129247 | 0.0496331463098835 | 0.04341203351104343 | 0.0464382326420198 | 0.0101744186046512 | 0.06828269033799933 | 0.053239017125837715 |
| 0.14000000000000001 | 0.00317460317460317 | 0.06828193832599122 | 0.0273660205245154 | 0.0277563608326908 | 0.05941542884523244 | 0.05442176870748298 | 0.052654294346137234 | 0.040365575019040416 | 0.04959422903516683 | 0.008720930232558145 | 0.07101399795151934 | 0.06664184661206246 |
| 0.16 | 0.00317460317460317 | 0.058002936857562436 | 0.0148232611174458 | 0.025443330763299913 | 0.05270723526593202 | 0.06394557823129247 | 0.052654294346137234 | 0.04341203351104343 | 0.05004508566275916 | 0.00799418604651163 | 0.06691703653123933 | 0.0569620253164557 |
| 0.18000000000000005 | 0.007936507936507941 | 0.049192364170337725 | 0.015963511972634 | 0.0346954510408635 | 0.06612362242453282 | 0.0653061224489796 | 0.05783340526542941 | 0.05483625285605482 | 0.0536519386834987 | 0.0145348837209302 | 0.0525776715602595 | 0.0524944154877141 |
| 0.2 | 0.0158730158730159 | 0.0535976505139501 | 0.02052451539338649 | 0.0377794911333847 | 0.0570196454240537 | 0.0612244897959184 | 0.0574018126888218 | 0.059405940594059396 | 0.055455365193868386 | 0.02398255813953488 | 0.05633321952884942 | 0.061429635145197316 |
| 0.22 | 0.0158730158730159 | 0.04772393538913364 | 0.0193842645381984 | 0.0624518118735544 | 0.05174892189746051 | 0.05034013605442183 | 0.0647388864911524 | 0.0715917745620716 | 0.059963931469792627 | 0.044331395348837226 | 0.05462615227039948 | 0.0547282204020849 |
| 0.24000000000000005 | 0.0492063492063492 | 0.044787077826725454 | 0.0353477765108324 | 0.0786430223592907 | 0.05845711547676088 | 0.0612244897959184 | 0.06819162710401383 | 0.08530083777608528 | 0.0622182146077547 | 0.057412790697674417 | 0.0518948446568795 | 0.059940431868950116 |
| 0.26 | 0.0761904761904762 | 0.0345080763582966 | 0.0399087799315849 | 0.06322282189668471 | 0.049832295160517515 | 0.05986394557823132 | 0.06603366422097542 | 0.06702208682406703 | 0.06447249774571694 | 0.08357558139534885 | 0.03755547968589961 | 0.05919583023082653 |
| 0.28000000000000008 | 0.09365079365079373 | 0.030837004405286306 | 0.0399087799315849 | 0.0940632228218967 | 0.04456157163392432 | 0.0666666666666667 | 0.0552438498057833 | 0.06397562833206398 | 0.073038773669973 | 0.118459302325581 | 0.03311710481392972 | 0.04951600893521973 |
| 0.3000000000000001 | 0.111111111111111 | 0.021292217327459617 | 0.05359179019384262 | 0.08481110254433313 | 0.0388116914230954 | 0.03537414965986392 | 0.046180405697021996 | 0.04874333587204872 | 0.044634806131650086 | 0.12136627906976702 | 0.03448275862068972 | 0.04095309009679822 |
| 0.32000000000000012 | 0.10793650793650804 | 0.0168869309838473 | 0.0592930444697834 | 0.07093292212798773 | 0.0330618112122664 | 0.02993197278911562 | 0.0349589987052223 | 0.0418888042650419 | 0.0577096483318305 | 0.106831395348837 | 0.029702970297029712 | 0.03574087862993301 |
| 0.34 | 0.115873015873016 | 0.0102790014684288 | 0.0684150513112885 | 0.0701619121048574 | 0.041686631528509814 | 0.024489795918367314 | 0.025463962019853317 | 0.04645849200304652 | 0.0473399458972047 | 0.0930232558139535 | 0.025606008876749714 | 0.0323901712583768 |
| 0.3600000000000001 | 0.0984126984126984 | 0.0110132158590308 | 0.0592930444697834 | 0.05936777178103324 | 0.024916147580258705 | 0.02585034013605441 | 0.021148036253776398 | 0.0236100533130236 | 0.0333633904418395 | 0.07340116279069768 | 0.0136565380675999 | 0.02047654504839911 |
| 0.38000000000000012 | 0.0857142857142857 | 0.0102790014684288 | 0.06271379703534785 | 0.031611410948342314 | 0.020603737422137013 | 0.017687074829932006 | 0.0146741476046612 | 0.0312261995430312 | 0.015329125338142508 | 0.05450581395348842 | 0.0160464322294298 | 0.0186150409530901 |
| 0.4 | 0.050793650793650814 | 0.007342143906020562 | 0.0524515393386545 | 0.01696222050886661 | 0.016291327264015307 | 0.005442176870748298 | 0.012947777298230509 | 0.013709063214013705 | 0.017583408476104602 | 0.0399709302325581 | 0.010242403550699898 | 0.0122859270290395 |
| 0.4200000000000001 | 0.039682539682539715 | 0.005139500734214393 | 0.03876852907639679 | 0.011565150346954507 | 0.0124580737901294 | 0.006802721088435372 | 0.009926629261976695 | 0.008377760853008388 | 0.013074842200180301 | 0.019622093023255807 | 0.0034141345168999726 | 0.009679821295606853 |
| 0.44 | 0.028571428571428602 | 0.00146842878120411 | 0.030786773090079808 | 0.011565150346954507 | 0.00718735026353618 | 0.00816326530612245 | 0.001726370306430731 | 0.004569687738004572 | 0.003155996393146981 | 0.01308139534883721 | 0.003755547968589962 | 0.004095309009679822 |
| 0.46 | 0.011111111111111101 | 0.0007342143906020563 | 0.022805017103762818 | 0.006168080185042411 | 0.005270723526593202 | 0.00136054421768707 | 0.005179110919292192 | 0.00304645849200305 | 0.00766456266907124 | 0.0036337209302325616 | 0.003072721065209971 | 0.004095309009679822 |
| 0.48000000000000009 | 0.007936507936507941 | 0.00220264317180617 | 0.0193842645381984 | 0.0007710100231303012 | 0.00383325347388596 | 0.00136054421768707 | 0.00258955545964609 | 0.00304645849200305 | 0.003155996393146981 | 0.006540697674418614 | 0.002389894161829981 | 0.0033507073715562208 |
| 0.5 | 0.009523809523809523 | 0.0 | 0.0171037628278221 | 0.0 | 0.00287494010541447 | 0.0 | 0.000863185153215365 | 0.00304645849200305 | 0.0013525698827772804 | 0.00145348837209302 | 0.00102424035506999 | 0.0014892032762472101 |
| 0.52 | 0.00476190476190476 | 0.0007342143906020563 | 0.0182440136830103 | 0.0 | 0.0009583133684714904 | 0.0 | 0.000863185153215365 | 0.0 | 0.0 | 0.0 | 0.0 | 0.0007446016381236044 |
| 0.54 | 0.00317460317460317 | 0.0 | 0.0102622576966933 | 0.0015420200462606005 | 0.0009583133684714904 | 0.00136054421768707 | 0.0004315925766076819 | 0.0 | 0.0 | 0.0029069767441860512 | 0.0 | 0.0003723008190618021 |
| 0.56000000000000005 | 0.0 | 0.0 | 0.006841505131128852 | 0.0 | 0.0009583133684714904 | 0.00136054421768707 | 0.0004315925766076819 | 0.0 | 0.00045085662759242624 | 0.00145348837209302 | 0.00034141345168999725 | 0.0 |
| 0.58000000000000007 | 0.0 | 0.0 | 0.0057012542759407115 | 0.0 | 0.0 | 0.0 | 0.0 | 0.0 | 0.00045085662759242624 | 0.0 | 0.0 | 0.0 |
| 0.6000000000000002 | 0.0015873015873015901 | 0.0 | 0.003420752565564421 | 0.0 | 0.000479156684235745 | 0.0 | 0.0 | 0.0 | 0.0 | 0.0 | 0.0 | 0.0 |
### Chart: w.t. A2
| Category | N2_A2_40_30_02 | N2_A2_40_30_03 | N2_A2_40_30_04 | N2_REX_A2_01 | N2_REX_A2_02 | N2_REX_A2_03 | N2_REX_A2_04 | N2_REX_A2_08 | N2_REX_A2_09 | N2_REX_A2_10 |
|---|---|---|---|---|---|---|---|---|---|---|
| 0 | None | None | None | None | None | None | None | None | None | None |
| 2.0000000000000007E-2 | 0.10512129380053906 | 0.0394088669950739 | 0.06000000000000002 | 0.0546149645002731 | 0.034149117814456516 | 0.05241090146750522 | 0.04129606099110551 | 0.040312093628088436 | 0.0237154150197628 | 0.025056947608200517 |
| 4.0000000000000015E-2 | 0.0700808625336927 | 0.026272577996715913 | 0.04285714285714291 | 0.0458765701802294 | 0.033010813887307915 | 0.0426275331935709 | 0.05844980940279538 | 0.0299089726918075 | 0.016600790513834007 | 0.029612756264236893 |
| 6.0000000000000019E-2 | 0.04851752021563343 | 0.03284072249589491 | 0.06000000000000002 | 0.06826870562534132 | 0.036425725668753614 | 0.04751921733053812 | 0.05019059720457431 | 0.036410923276983115 | 0.025296442687747007 | 0.018223234624145802 |
| 8.0000000000000029E-2 | 0.03773584905660382 | 0.022988505747126402 | 0.020000000000000007 | 0.062261059530311316 | 0.035287421741605 | 0.03144654088050313 | 0.04828462515883103 | 0.018205461638491505 | 0.018972332015810306 | 0.013667425968109305 |
| 0.1 | 0.029649595687331512 | 0.0180623973727422 | 0.00571428571428571 | 0.0557072637902785 | 0.034149117814456516 | 0.03983228511530401 | 0.056543837357052096 | 0.0286085825747724 | 0.015019762845849795 | 0.011389521640091107 |
| 0.12000000000000002 | 0.0188679245283019 | 0.019704433497536908 | 0.01714285714285711 | 0.0546149645002731 | 0.0347182697780307 | 0.03144654088050313 | 0.05146124523506988 | 0.0143042912873862 | 0.0118577075098814 | 0.004555808656036448 |
| 0.14000000000000001 | 0.013477088948787106 | 0.00656814449917898 | 0.00857142857142857 | 0.06116876024030581 | 0.03870233352305071 | 0.0307477288609364 | 0.047013977128335535 | 0.010403120936280904 | 0.006324110671936756 | 0.004555808656036448 |
| 0.16 | 0.021563342318059318 | 0.01642036124794751 | 0.03142857142857142 | 0.0524303659202622 | 0.025042686397268085 | 0.03144654088050313 | 0.0552731893265565 | 0.009102730819245777 | 0.0110671936758893 | 0.00911161731207289 |
| 0.18000000000000005 | 0.0350404312668464 | 0.0213464696223317 | 0.00571428571428571 | 0.048607318405243 | 0.039840637450199216 | 0.0426275331935709 | 0.043837357052096626 | 0.01950585175552671 | 0.018972332015810306 | 0.018223234624145802 |
| 0.2 | 0.029649595687331512 | 0.022988505747126402 | 0.0257142857142857 | 0.06389950846531949 | 0.0529311326124075 | 0.048916841369671615 | 0.045108005082592086 | 0.04421326397919382 | 0.02055335968379451 | 0.031890660592255114 |
| 0.22 | 0.04043126684636122 | 0.0394088669950739 | 0.0514285714285714 | 0.060076460950300434 | 0.06943653955606152 | 0.05939902166317262 | 0.05400254129606102 | 0.061118335500650184 | 0.04031620553359682 | 0.04783599088838272 |
| 0.24000000000000005 | 0.04043126684636122 | 0.05418719211822662 | 0.0628571428571429 | 0.06389950846531949 | 0.07569721115537853 | 0.0740740740740741 | 0.06925031766200758 | 0.07542262678803643 | 0.0735177865612648 | 0.054669703872437414 |
| 0.26 | 0.05121293800539081 | 0.09031198686371096 | 0.0628571428571429 | 0.0524303659202622 | 0.09391007398975533 | 0.07966457023060802 | 0.062261753494282084 | 0.08452535760728228 | 0.10988142292490102 | 0.0774487471526196 |
| 0.28000000000000008 | 0.06738544474393533 | 0.09688013136289 | 0.0514285714285714 | 0.048607318405243 | 0.08935685828116116 | 0.0859538784067086 | 0.059720457433291026 | 0.08842652795838755 | 0.132806324110672 | 0.12300683371298403 |
| 0.3000000000000001 | 0.0592991913746631 | 0.11330049261083695 | 0.10285714285714298 | 0.041507373020207496 | 0.07569721115537853 | 0.07058001397624043 | 0.05908513341804324 | 0.08452535760728228 | 0.111462450592885 | 0.125284738041002 |
| 0.32000000000000012 | 0.0592991913746631 | 0.09195402298850573 | 0.05428571428571431 | 0.03604587657018021 | 0.055776892430278904 | 0.06988120195667374 | 0.041931385006353177 | 0.061118335500650184 | 0.10988142292490102 | 0.129840546697039 |
| 0.34 | 0.056603773584905696 | 0.077175697865353 | 0.03142857142857142 | 0.022392135445112007 | 0.04553215708594192 | 0.040531097134870714 | 0.031130876747141007 | 0.049414824447334235 | 0.07272727272727271 | 0.07289293849658313 |
| 0.3600000000000001 | 0.029649595687331512 | 0.06896551724137931 | 0.045714285714285714 | 0.020207536865101006 | 0.03187250996015941 | 0.02935010482180289 | 0.021601016518424422 | 0.04421326397919382 | 0.061660079051383404 | 0.07972665148063783 |
| 0.38000000000000012 | 0.02695417789757412 | 0.049261083743842415 | 0.022857142857142906 | 0.014199890770070995 | 0.025042686397268085 | 0.020265548567435402 | 0.014612452350698898 | 0.032509752925877815 | 0.0411067193675889 | 0.04783599088838272 |
| 0.4 | 0.00808625336927224 | 0.01642036124794751 | 0.0257142857142857 | 0.007646095030038232 | 0.014797951052931099 | 0.011879804332634504 | 0.009529860228716654 | 0.022106631989596892 | 0.02450592885375491 | 0.025056947608200517 |
| 0.4200000000000001 | 0.0 | 0.013136288998358 | 0.00857142857142857 | 0.005461496450027312 | 0.007968127490039844 | 0.00419287211740042 | 0.008259212198221094 | 0.011703511053316008 | 0.00711462450592885 | 0.01594533029612761 |
| 0.44 | 0.0 | 0.008210180623973726 | 0.00285714285714286 | 0.0038230475150191202 | 0.00512236767216847 | 0.00419287211740042 | 0.00381194409148666 | 0.011703511053316008 | 0.0055335968379446624 | 0.004555808656036448 |
| 0.46 | 0.0026954177897574112 | 0.009852216748768476 | 0.0 | 0.001638448935008191 | 0.002276607854297101 | 0.002096436058700211 | 0.002541296060991111 | 0.009102730819245777 | 0.00316205533596838 | 0.004555808656036448 |
| 0.48000000000000009 | 0.0 | 0.004926108374384242 | 0.0 | 0.0010922992900054595 | 0.0028457598178713707 | 0.002096436058700211 | 0.0 | 0.0026007802340702207 | 0.00237154150197628 | 0.0 |
| 0.5 | 0.0 | 0.0 | 0.0 | 0.0010922992900054595 | 0.0 | 0.0006988120195667376 | 0.0006353240152477763 | 0.0026007802340702207 | 0.00316205533596838 | 0.0 |
| 0.52 | 0.0 | 0.0 | 0.0 | 0.001638448935008191 | 0.0005691519635742742 | 0.0 | 0.0006353240152477763 | 0.00130039011703511 | 0.0007905138339920952 | 0.002277904328018221 |
| 0.54 | 0.0 | 0.0 | 0.0 | 0.0 | 0.0 | 0.00139762403913347 | 0.0006353240152477763 | 0.00130039011703511 | 0.0 | 0.0 |
| 0.56000000000000005 | 0.0 | 0.0 | 0.0 | 0.0 | 0.0005691519635742742 | 0.0 | 0.0006353240152477763 | 0.0026007802340702207 | 0.0 | 0.0 |
| 0.58000000000000007 | 0.0 | 0.0 | 0.0 | 0.0 | 0.0 | 0.0 | 0.0 | 0.0 | 0.0 | 0.0 |
| 0.6000000000000002 | 0.0 | 0.0 | 0.0 | 0.0 | 0.0 | 0.0 | 0.0 | 0.00130039011703511 | 0.0 | 0.002277904328018221 |
### Chart: w.t. A3
| Category | N2_A3_40_30_03 | N2_A3_40_30_2 | N2_REX_A3_01 | N2_REX_A3_02 | N2_REX_A3_03 | N2_REX_A3_04 | N2_REX_A3_05 | N2_REX_A3_07 | N2_REX_A3_08 | N2_REX_A3_09 |
|---|---|---|---|---|---|---|---|---|---|---|
| 0 | None | None | None | None | None | None | None | None | None | None |
| 2.0000000000000007E-2 | 0.0198170731707317 | 0.05181347150259072 | 0.0174563591022444 | 0.07943262411347525 | 0.0500807754442649 | 0.093681917211329 | 0.09375000000000004 | 0.0773480662983425 | 0.0416666666666667 | 0.0625 |
| 4.0000000000000015E-2 | 0.039634146341463415 | 0.04792746113989643 | 0.0149625935162095 | 0.051063829787234 | 0.04684975767366716 | 0.09694989106753808 | 0.08388157894736843 | 0.0718232044198895 | 0.05520833333333332 | 0.06475903614457833 |
| 6.0000000000000019E-2 | 0.0198170731707317 | 0.04404145077720208 | 0.019950124688279308 | 0.03829787234042551 | 0.025848142164781908 | 0.05446623093681926 | 0.06743421052631583 | 0.055248618784530384 | 0.04375 | 0.05647590361445778 |
| 8.0000000000000029E-2 | 0.0167682926829268 | 0.04533678756476682 | 0.012468827930174599 | 0.024113475177305006 | 0.025848142164781908 | 0.0381263616557734 | 0.03289473684210532 | 0.04656669297553282 | 0.02083333333333331 | 0.05722891566265057 |
| 0.1 | 0.00609756097560976 | 0.0284974093264249 | 0.004987531172069832 | 0.025531914893617006 | 0.006462035541195478 | 0.0381263616557734 | 0.019736842105263205 | 0.032359905288082115 | 0.02083333333333331 | 0.041415662650602404 |
| 0.12000000000000002 | 0.0076219512195122 | 0.031088082901554407 | 0.009975062344139654 | 0.019858156028368806 | 0.012924071082391 | 0.029411764705882398 | 0.016447368421052603 | 0.02604577742699292 | 0.017708333333333305 | 0.03388554216867472 |
| 0.14000000000000001 | 0.015243902439024399 | 0.024611398963730602 | 0.009975062344139654 | 0.005673758865248232 | 0.008077544426494354 | 0.03267973856209151 | 0.004934210526315788 | 0.030781373322809818 | 0.009375000000000007 | 0.0316265060240964 |
| 0.16 | 0.013719512195122 | 0.0401554404145078 | 0.004987531172069832 | 0.009929078014184403 | 0.0113085621970921 | 0.0217864923747277 | 0.02302631578947372 | 0.030781373322809818 | 0.0125 | 0.02334337349397591 |
| 0.18000000000000005 | 0.0121951219512195 | 0.0297927461139896 | 0.009975062344139654 | 0.0170212765957447 | 0.004846526655896612 | 0.020697167755991314 | 0.03782894736842112 | 0.028413575374901308 | 0.011458333333333301 | 0.03012048192771079 |
| 0.2 | 0.024390243902439 | 0.024611398963730602 | 0.007481296758104744 | 0.0212765957446809 | 0.024232633279483 | 0.0392156862745098 | 0.0246710526315789 | 0.030781373322809818 | 0.026041666666666713 | 0.0474397590361446 |
| 0.22 | 0.032012195121951206 | 0.0284974093264249 | 0.024937655860349107 | 0.0354609929078014 | 0.008077544426494354 | 0.049019607843137344 | 0.03618421052631582 | 0.04262036306235198 | 0.022916666666666707 | 0.0331325301204819 |
| 0.24000000000000005 | 0.0472560975609756 | 0.03626943005181352 | 0.03241895261845392 | 0.039716312056737604 | 0.03231017770597741 | 0.0392156862745098 | 0.07565789473684212 | 0.0347277032359905 | 0.05 | 0.0542168674698795 |
| 0.26 | 0.08689024390243903 | 0.03238341968911923 | 0.107231920199501 | 0.0666666666666667 | 0.06946688206785141 | 0.05555555555555558 | 0.07565789473684212 | 0.04340962904498822 | 0.061458333333333316 | 0.051204819277108383 |
| 0.28000000000000008 | 0.09756097560975616 | 0.04533678756476682 | 0.14713216957606007 | 0.09078014184397158 | 0.08562197092084017 | 0.05555555555555558 | 0.0625 | 0.05288082083662193 | 0.08333333333333333 | 0.050451807228915714 |
| 0.3000000000000001 | 0.12804878048780508 | 0.0569948186528497 | 0.14713216957606007 | 0.08794326241134749 | 0.103392568659128 | 0.037037037037037014 | 0.060855263157894704 | 0.061562746645619615 | 0.09791666666666671 | 0.07228915662650597 |
| 0.32000000000000012 | 0.0807926829268293 | 0.09455958549222804 | 0.12219451371571104 | 0.060992907801418444 | 0.10016155088853006 | 0.05664488017429188 | 0.03289473684210532 | 0.063930544593528 | 0.084375 | 0.06475903614457833 |
| 0.34 | 0.08689024390243903 | 0.0764248704663212 | 0.09476309226932673 | 0.052482269503546126 | 0.07754442649434573 | 0.026143790849673214 | 0.016447368421052603 | 0.05051302288871352 | 0.06458333333333334 | 0.0331325301204819 |
| 0.3600000000000001 | 0.0579268292682927 | 0.062176165803108814 | 0.07481296758104744 | 0.0340425531914894 | 0.07592891760904692 | 0.0196078431372549 | 0.00986842105263158 | 0.0355169692186267 | 0.046874999999999986 | 0.0399096385542169 |
| 0.38000000000000012 | 0.038109756097561 | 0.0660621761658031 | 0.0374064837905237 | 0.015602836879432603 | 0.03231017770597741 | 0.007625272331154683 | 0.0 | 0.02604577742699292 | 0.025 | 0.016566265060241 |
| 0.4 | 0.0274390243902439 | 0.03886010362694301 | 0.0349127182044888 | 0.009929078014184403 | 0.0274636510500808 | 0.00980392156862745 | 0.0 | 0.025256511444356707 | 0.02083333333333331 | 0.0173192771084337 |
| 0.4200000000000001 | 0.010670731707317105 | 0.0233160621761658 | 0.019950124688279308 | 0.009929078014184403 | 0.0274636510500808 | 0.0032679738562091526 | 0.004934210526315788 | 0.00789265982636148 | 0.015625 | 0.0112951807228916 |
| 0.44 | 0.00609756097560976 | 0.019430051813471506 | 0.009975062344139654 | 0.0028368794326241093 | 0.008077544426494354 | 0.00217864923747277 | 0.003289473684210531 | 0.00868192580899763 | 0.006250000000000002 | 0.0030120481927710797 |
| 0.46 | 0.004573170731707322 | 0.010362694300518104 | 0.007481296758104744 | 0.0014184397163120599 | 0.0016155088852988701 | 0.00217864923747277 | 0.0016447368421052605 | 0.003157063930544591 | 0.0010416666666666701 | 0.0007530120481927713 |
| 0.48000000000000009 | 0.0030487804878048808 | 0.0038860103626943017 | 0.002493765586034911 | 0.0014184397163120599 | 0.0016155088852988701 | 0.0010893246187363799 | 0.0 | 0.0 | 0.00416666666666667 | 0.0007530120481927713 |
| 0.5 | 0.00152439024390244 | 0.00129533678756477 | 0.0 | 0.0028368794326241093 | 0.0 | 0.0 | 0.0016447368421052605 | 0.002367797947908452 | 0.0 | 0.00150602409638554 |
| 0.52 | 0.0 | 0.0038860103626943017 | 0.002493765586034911 | 0.0 | 0.0032310177705977415 | 0.0 | 0.0 | 0.0 | 0.0010416666666666701 | 0.00150602409638554 |
| 0.54 | 0.0 | 0.00129533678756477 | 0.0 | 0.0 | 0.0016155088852988701 | 0.0 | 0.0 | 0.0 | 0.0010416666666666701 | 0.0 |
| 0.56000000000000005 | 0.0 | 0.0 | 0.0 | 0.0 | 0.0016155088852988701 | 0.0 | 0.0 | 0.0 | 0.0 | 0.0 |
| 0.58000000000000007 | 0.0 | 0.0 | 0.0 | 0.0 | 0.0 | 0.0 | 0.0 | 0.0 | 0.0 | 0.0 |
| 0.6000000000000002 | 0.0 | 0.0 | 0.0 | 0.0 | 0.0 | 0.0 | 0.0 | 0.0007892659826361482 | 0.0 | 0.0 |
### Chart: w.t. A5
| Category | N2_A5_001 | N2_A5_003 | N2_A5_004 | N2_A5_40_30_01 | N2_A5_40_30_02 | N2_A5_40_30_04 | N2_REX_A5_01 | N2_REX_A5_02 | N2_REX_A5_04 | N2_REX_A5_05 | N2_REX_A5_06 | N2_REX_A5_08 | N2_REX_A5_09 |
|---|---|---|---|---|---|---|---|---|---|---|---|---|---|
| 0 | None | None | None | None | None | None | None | None | None | None | None | None | None |
| 2.0000000000000007E-2 | 0.04795737122557731 | 0.150259067357513 | 0.0376175548589342 | 0.0 | 0.0617283950617284 | 0.0739371534195934 | 0.109053497942387 | 0.02223816355810621 | 0.09770114942528743 | 0.055045871559633 | 0.111455108359133 | 0.10483870967741903 | 0.0731707317073171 |
| 4.0000000000000015E-2 | 0.03907637655417412 | 0.09326424870466327 | 0.0282131661442006 | 0.02654867256637171 | 0.06525573192239863 | 0.06839186691312393 | 0.0637860082304527 | 0.031563845050215214 | 0.07931034482758624 | 0.055045871559633 | 0.08436532507739947 | 0.109879032258065 | 0.036585365853658514 |
| 6.0000000000000019E-2 | 0.0319715808170515 | 0.05181347150259072 | 0.026645768025078415 | 0.0 | 0.045855379188712485 | 0.048059149722735686 | 0.0349794238683128 | 0.0200860832137733 | 0.04827586206896546 | 0.07339449541284403 | 0.0696594427244582 | 0.07157258064516131 | 0.08536585365853663 |
| 8.0000000000000029E-2 | 0.02309058614564828 | 0.03626943005181352 | 0.02507836990595611 | 0.008849557522123897 | 0.02645502645502651 | 0.018484288354898303 | 0.0329218106995885 | 0.010043041606886701 | 0.0413793103448276 | 0.055045871559633 | 0.04411764705882354 | 0.047379032258064495 | 0.024390243902439 |
| 0.1 | 0.01953818827708701 | 0.03238341968911923 | 0.0156739811912226 | 0.02654867256637171 | 0.0282186948853616 | 0.0203327171903882 | 0.012345679012345704 | 0.010043041606886701 | 0.022988505747126402 | 0.027522935779816515 | 0.030185758513931907 | 0.03528225806451611 | 0.0731707317073171 |
| 0.12000000000000002 | 0.017761989342806407 | 0.0233160621761658 | 0.0125391849529781 | 0.008849557522123897 | 0.00529100529100529 | 0.014787430683918705 | 0.012345679012345704 | 0.00932568149210905 | 0.016091954022988502 | 0.027522935779816515 | 0.0309597523219814 | 0.0383064516129032 | 0.036585365853658514 |
| 0.14000000000000001 | 0.017761989342806407 | 0.022020725388601 | 0.0141065830721003 | 0.008849557522123897 | 0.012345679012345704 | 0.018484288354898303 | 0.018518518518518507 | 0.012912482065997101 | 0.016091954022988502 | 0.018348623853211 | 0.0286377708978328 | 0.02620967741935482 | 0.0609756097560976 |
| 0.16 | 0.0159857904085258 | 0.014248704663212406 | 0.007836990595611283 | 0.008849557522123897 | 0.0105820105820106 | 0.01663585951940851 | 0.016460905349794205 | 0.01936872309899571 | 0.017241379310344803 | 0.00917431192660551 | 0.0232198142414861 | 0.029233870967741913 | 0.0121951219512195 |
| 0.18000000000000005 | 0.0319715808170515 | 0.014248704663212406 | 0.00940438871473354 | 0.0353982300884956 | 0.00529100529100529 | 0.022181146025878017 | 0.012345679012345704 | 0.0200860832137733 | 0.021839080459770118 | 0.018348623853211 | 0.031733746130031 | 0.030241935483871028 | 0.024390243902439 |
| 0.2 | 0.044404973357016035 | 0.019430051813471506 | 0.017241379310344803 | 0.0353982300884956 | 0.012345679012345704 | 0.0295748613678373 | 0.02469135802469141 | 0.05380200860832142 | 0.03218390804597701 | 0.027522935779816515 | 0.0325077399380805 | 0.033266129032258084 | 0.036585365853658514 |
| 0.22 | 0.03907637655417412 | 0.0233160621761658 | 0.02194357366771161 | 0.044247787610619496 | 0.019400352733686108 | 0.0295748613678373 | 0.016460905349794205 | 0.0588235294117647 | 0.03218390804597701 | 0.055045871559633 | 0.03869969040247681 | 0.0342741935483871 | 0.024390243902439 |
| 0.24000000000000005 | 0.0319715808170515 | 0.024611398963730602 | 0.03448275862068972 | 0.053097345132743404 | 0.03880070546737211 | 0.046210720887245815 | 0.037037037037037014 | 0.0918220946915352 | 0.05402298850574712 | 0.08256880733944956 | 0.03715170278637772 | 0.04334677419354842 | 0.0731707317073171 |
| 0.26 | 0.044404973357016035 | 0.015544041450777204 | 0.040752351097178716 | 0.0973451327433628 | 0.0617283950617284 | 0.0628465804066543 | 0.0637860082304527 | 0.12482065997130604 | 0.0586206896551724 | 0.07339449541284403 | 0.043343653250774 | 0.0383064516129032 | 0.04878048780487813 |
| 0.28000000000000008 | 0.04262877442273531 | 0.0297927461139896 | 0.0673981191222571 | 0.15929203539823011 | 0.05996472663139333 | 0.0628465804066543 | 0.06790123456790123 | 0.12051649928264002 | 0.07931034482758624 | 0.10091743119266094 | 0.04876160990712074 | 0.037298387096774216 | 0.04878048780487813 |
| 0.3000000000000001 | 0.035523978685612814 | 0.0207253886010363 | 0.0721003134796238 | 0.10619469026548706 | 0.0758377425044092 | 0.0739371534195934 | 0.04115226337448558 | 0.0989956958393113 | 0.0586206896551724 | 0.11926605504587204 | 0.03560371517027862 | 0.0413306451612903 | 0.0609756097560976 |
| 0.32000000000000012 | 0.04795737122557731 | 0.0181347150259067 | 0.08150470219435747 | 0.0619469026548673 | 0.0634920634920635 | 0.103512014787431 | 0.0349794238683128 | 0.07460545193687233 | 0.04597701149425289 | 0.03669724770642203 | 0.033281733746130006 | 0.033266129032258084 | 0.024390243902439 |
| 0.34 | 0.0568383658969805 | 0.014248704663212406 | 0.0752351097178683 | 0.11504424778761109 | 0.047619047619047616 | 0.0425138632162662 | 0.02880658436213989 | 0.05093256814921091 | 0.024137931034482793 | 0.03669724770642203 | 0.02554179566563471 | 0.0211693548387097 | 0.04878048780487813 |
| 0.3600000000000001 | 0.02309058614564828 | 0.0064766839378238355 | 0.057993730407523536 | 0.0353982300884956 | 0.0299823633156966 | 0.0388170055452865 | 0.010288065843621401 | 0.0337159253945481 | 0.024137931034482793 | 0.027522935779816515 | 0.02089783281733751 | 0.015120967741935505 | 0.0121951219512195 |
| 0.38000000000000012 | 0.035523978685612814 | 0.005181347150259074 | 0.0391849529780564 | 0.0176991150442478 | 0.02645502645502651 | 0.014787430683918705 | 0.00411522633744856 | 0.0179340028694405 | 0.0114942528735632 | 0.0 | 0.012383900928792598 | 0.0221774193548387 | 0.036585365853658514 |
| 0.4 | 0.03730017761989342 | 0.005181347150259074 | 0.0438871473354232 | 0.02654867256637171 | 0.012345679012345704 | 0.00369685767097967 | 0.00617283950617284 | 0.010760401721664304 | 0.005747126436781612 | 0.0 | 0.005417956656346748 | 0.008064516129032265 | 0.0 |
| 0.4200000000000001 | 0.02486678507992901 | 0.005181347150259074 | 0.0235109717868339 | 0.0176991150442478 | 0.008818342151675476 | 0.007393715341959332 | 0.00823045267489712 | 0.00573888091822095 | 0.008045977011494253 | 0.00917431192660551 | 0.00154798761609907 | 0.007056451612903233 | 0.0 |
| 0.44 | 0.026642984014209607 | 0.005181347150259074 | 0.03134796238244512 | 0.008849557522123897 | 0.008818342151675476 | 0.0018484288354898301 | 0.00617283950617284 | 0.005021520803443332 | 0.006896551724137932 | 0.0 | 0.00154798761609907 | 0.0030241935483871028 | 0.0 |
| 0.46 | 0.0106571936056838 | 0.0038860103626943017 | 0.017241379310344803 | 0.0 | 0.00529100529100529 | 0.0018484288354898301 | 0.00205761316872428 | 0.00143472022955524 | 0.0011494252873563199 | 0.0 | 0.00232198142414861 | 0.00100806451612903 | 0.0 |
| 0.48000000000000009 | 0.012433392539964498 | 0.0064766839378238355 | 0.0125391849529781 | 0.0 | 0.0 | 0.0018484288354898301 | 0.0 | 0.00215208034433286 | 0.0034482758620689707 | 0.0 | 0.00232198142414861 | 0.0 | 0.0 |
| 0.5 | 0.00532859680284192 | 0.0038860103626943017 | 0.00940438871473354 | 0.0 | 0.003527336860670192 | 0.0 | 0.00205761316872428 | 0.0 | 0.00229885057471264 | 0.0 | 0.0 | 0.00100806451612903 | 0.0 |
| 0.52 | 0.00532859680284192 | 0.00129533678756477 | 0.00470219435736677 | 0.0 | 0.0017636684303351004 | 0.0 | 0.0 | 0.0007173601147776184 | 0.0011494252873563199 | 0.0 | 0.0 | 0.0030241935483871028 | 0.0 |
| 0.54 | 0.0142095914742451 | 0.00129533678756477 | 0.003134796238244512 | 0.0 | 0.0017636684303351004 | 0.0 | 0.00617283950617284 | 0.00143472022955524 | 0.0 | 0.0 | 0.0 | 0.0 | 0.0 |
| 0.56000000000000005 | 0.0035523978685612816 | 0.0038860103626943017 | 0.003134796238244512 | 0.0 | 0.0017636684303351004 | 0.0 | 0.0 | 0.0007173601147776184 | 0.0 | 0.0 | 0.0007739938080495364 | 0.0 | 0.0 |
| 0.58000000000000007 | 0.0035523978685612816 | 0.00129533678756477 | 0.0062695924764890314 | 0.0 | 0.0 | 0.0 | 0.00205761316872428 | 0.0 | 0.0 | 0.0 | 0.0 | 0.0 | 0.0 |
| 0.6000000000000002 | 0.00710479573712256 | 0.0 | 0.003134796238244512 | 0.0 | 0.0 | 0.0018484288354898301 | 0.0 | 0.0 | 0.0 | 0.0 | 0.0 | 0.0 | 0.0 |
### Chart: unc-25 L4
| Category | unc-25_L4_06001 | unc-25_L4_06002 | unc-25_L4_06003 | unc-25_L4_06004 | unc-25_L4_06005 | unc-25_L4_06006 | unc-25_L4_06007 | unc-25_L4_06008 | unc-25_L4_06009 | unc-25_L4_06011 | unc-25_L4_06012 | unc-25_L4_06013 | unc-25_L4_06014 | unc-25_L4_06015 | unc-25_L4_06016 | unc-25_L4_06017 | unc-25_L4_06018 | unc-25_L4_06019 |
|---|---|---|---|---|---|---|---|---|---|---|---|---|---|---|---|---|---|---|
| 0 | None | None | None | None | None | None | None | None | None | None | None | None | None | None | None | None | None | None |
| 2.0000000000000007E-2 | 0.03448275862068972 | 0.02400835073068889 | 0.043151969981238304 | 0.016560509554140106 | None | None | 0.025 | 0.0333333333333333 | 0.10344827586206902 | 0.019955654101995603 | 0.02567394094993581 | 0.027131782945736406 | 0.03294117647058822 | None | 0.06754221388367733 | 0.021712907117008406 | None | 0.018367346938775498 |
| 4.0000000000000015E-2 | 0.014146772767462405 | 0.026096033402922807 | 0.02626641651031901 | 0.016560509554140106 | None | None | 0.0144736842105263 | 0.037037037037037014 | 0.05172413793103452 | 0.0321507760532151 | 0.019255455712451908 | 0.017441860465116307 | 0.03176470588235291 | None | 0.035647279549718615 | 0.019300361881785306 | None | 0.012244897959183699 |
| 6.0000000000000019E-2 | 0.0203359858532272 | 0.014613778705636701 | 0.011257035647279505 | 0.015286624203821701 | None | None | 0.025 | 0.0148148148148148 | 0.017241379310344803 | 0.014412416851441194 | 0.02310654685494219 | 0.0193798449612403 | 0.021176470588235314 | None | 0.04502814258911818 | 0.015681544028950507 | None | 0.03673469387755101 |
| 8.0000000000000029E-2 | 0.0194518125552608 | 0.014613778705636701 | 0.011257035647279505 | 0.006369426751592362 | None | None | 0.017105263157894693 | 0.00740740740740741 | 0.03448275862068972 | 0.013303769401330405 | 0.0205391527599487 | 0.017441860465116307 | 0.02705882352941181 | None | 0.05253283302063792 | 0.015681544028950507 | None | 0.016326530612244903 |
| 0.1 | 0.015030946065428798 | 0.01148225469728601 | 0.009380863039399634 | 0.012738853503184698 | None | None | 0.006578947368421051 | 0.011111111111111101 | 0.05172413793103452 | 0.008869179600886927 | 0.01668806161745831 | 0.013565891472868205 | 0.012941176470588204 | None | 0.0375234521575985 | 0.016887816646562113 | None | 0.014285714285714301 |
| 0.12000000000000002 | 0.014146772767462405 | 0.016701461377870607 | 0.011257035647279505 | 0.003821656050955411 | None | None | 0.006578947368421051 | 0.011111111111111101 | 0.03448275862068972 | 0.011086474501108601 | 0.0115532734274711 | 0.011627906976744195 | 0.011764705882352905 | None | 0.04502814258911818 | 0.0120627261761158 | None | 0.014285714285714301 |
| 0.14000000000000001 | 0.012378426171529594 | 0.0135699373695198 | 0.00562851782363977 | 0.005095541401273892 | None | None | 0.013157894736842101 | 0.00740740740740741 | 0.05172413793103452 | 0.011086474501108601 | 0.007702182284980742 | 0.027131782945736406 | 0.009411764705882354 | None | 0.0393996247654784 | 0.013268998793727404 | None | 0.00816326530612245 |
| 0.16 | 0.013262599469496004 | 0.015657620041753702 | 0.007504690431519703 | 0.006369426751592362 | None | None | 0.015789473684210506 | 0.0037037037037037017 | 0.017241379310344803 | 0.014412416851441194 | 0.0102695763799743 | 0.02131782945736431 | 0.01882352941176471 | None | 0.020637898686679222 | 0.010856453558504198 | None | 0.0224489795918367 |
| 0.18000000000000005 | 0.017683465959328 | 0.0187891440501044 | 0.007504690431519703 | 0.008917197452229306 | None | None | 0.003947368421052632 | 0.0 | 0.017241379310344803 | 0.0254988913525499 | 0.0102695763799743 | 0.00968992248062015 | 0.012941176470588204 | None | 0.028142589118198884 | 0.013268998793727404 | None | 0.016326530612244903 |
| 0.2 | 0.0238726790450928 | 0.02505219206680581 | 0.0131332082551595 | 0.007643312101910834 | None | None | 0.013157894736842101 | 0.011111111111111101 | 0.0 | 0.02660753880266081 | 0.02310654685494219 | 0.0193798449612403 | 0.015294117647058804 | None | 0.0187617260787993 | 0.019300361881785306 | None | 0.012244897959183699 |
| 0.22 | 0.03271441202475692 | 0.034446764091858 | 0.022514071294559092 | 0.016560509554140106 | None | None | 0.0210526315789474 | 0.00740740740740741 | 0.03448275862068972 | 0.041019955654102 | 0.057766367137355626 | 0.02131782945736431 | 0.02352941176470589 | None | 0.020637898686679222 | 0.0120627261761158 | None | 0.024489795918367314 |
| 0.24000000000000005 | 0.054818744473916915 | 0.06471816283924844 | 0.046904315196998086 | 0.029299363057324817 | None | None | 0.02763157894736841 | 0.02962962962962961 | 0.0 | 0.0643015521064302 | 0.07188703465982033 | 0.05620155038759688 | 0.020000000000000007 | None | 0.035647279549718615 | 0.0180940892641737 | None | 0.03469387755102042 |
| 0.26 | 0.0804597701149425 | 0.08350730688935287 | 0.056285178236397684 | 0.05095541401273892 | None | None | 0.05 | 0.011111111111111101 | 0.05172413793103452 | 0.08536585365853663 | 0.09627727856225934 | 0.05620155038759688 | 0.051764705882352886 | None | 0.0393996247654784 | 0.04101326899879372 | None | 0.032653061224489806 |
| 0.28000000000000008 | 0.09725906277630424 | 0.07620041753653442 | 0.06378986866791737 | 0.0828025477707006 | None | None | 0.0763157894736842 | 0.059259259259259296 | 0.017241379310344803 | 0.0731707317073171 | 0.101412066752246 | 0.0930232558139535 | 0.0705882352941176 | None | 0.041275797373358285 | 0.0687575392038601 | None | 0.04285714285714291 |
| 0.3000000000000001 | 0.11582670203359906 | 0.08872651356993745 | 0.0881801125703565 | 0.118471337579618 | None | None | 0.0907894736842105 | 0.062962962962963 | 0.017241379310344803 | 0.09977827050997778 | 0.10654685494223404 | 0.08139534883720934 | 0.0858823529411765 | None | 0.043151969981238304 | 0.097708082026538 | None | 0.03673469387755101 |
| 0.32000000000000012 | 0.0963748894783378 | 0.08350730688935287 | 0.0731707317073171 | 0.12229299363057303 | None | None | 0.0907894736842105 | 0.0777777777777778 | 0.0 | 0.09866962305986704 | 0.09884467265725298 | 0.12015503875969002 | 0.10588235294117603 | None | 0.035647279549718615 | 0.09047044632086851 | None | 0.0612244897959184 |
| 0.34 | 0.07780725022104334 | 0.06784968684759923 | 0.08067542213883688 | 0.11337579617834398 | None | None | 0.109210526315789 | 0.10740740740740698 | 0.0 | 0.0698447893569845 | 0.06418485237483948 | 0.06782945736434112 | 0.0858823529411765 | None | 0.0656660412757974 | 0.10735826296743102 | None | 0.04081632653061222 |
| 0.3600000000000001 | 0.0680813439434129 | 0.0803757828810021 | 0.0731707317073171 | 0.0878980891719745 | None | None | 0.09473684210526329 | 0.10370370370370405 | 0.017241379310344803 | 0.0698447893569845 | 0.07188703465982033 | 0.05620155038759688 | 0.0752941176470588 | None | 0.0187617260787993 | 0.09891435464414966 | None | 0.051020408163265286 |
| 0.38000000000000012 | 0.0512820512820513 | 0.05741127348643012 | 0.058161350844277704 | 0.0662420382165605 | None | None | 0.07105263157894742 | 0.059259259259259296 | 0.05172413793103452 | 0.04878048780487813 | 0.0423620025673941 | 0.0542635658914729 | 0.08000000000000003 | None | 0.04878048780487813 | 0.0711700844390832 | None | 0.06734693877551023 |
| 0.4 | 0.045092838196286504 | 0.04070981210855948 | 0.058161350844277704 | 0.047133757961783415 | None | None | 0.05921052631578951 | 0.09259259259259267 | 0.03448275862068972 | 0.036585365853658514 | 0.03465982028241341 | 0.0348837209302326 | 0.04588235294117652 | None | 0.0300187617260788 | 0.0615199034981906 | None | 0.0653061224489796 |
| 0.4200000000000001 | 0.021220159151193598 | 0.029227557411273513 | 0.04502814258911818 | 0.024203821656051002 | None | None | 0.04736842105263167 | 0.0407407407407407 | 0.017241379310344803 | 0.0288248337028825 | 0.0205391527599487 | 0.031007751937984492 | 0.03411764705882352 | None | 0.0393996247654784 | 0.033775633293124205 | None | 0.055102040816326525 |
| 0.44 | 0.0185676392572944 | 0.029227557411273513 | 0.022514071294559092 | 0.029299363057324817 | None | None | 0.0210526315789474 | 0.0148148148148148 | 0.0 | 0.022172949002217314 | 0.012836970474967901 | 0.025193798449612406 | 0.029411764705882398 | None | 0.02626641651031901 | 0.0361881785283474 | None | 0.0571428571428571 |
| 0.46 | 0.0114942528735632 | 0.016701461377870607 | 0.0187617260787993 | 0.019108280254777107 | None | None | 0.017105263157894693 | 0.0148148148148148 | 0.0 | 0.016629711751663008 | 0.0102695763799743 | 0.00968992248062015 | 0.015294117647058804 | None | 0.0300187617260788 | 0.021712907117008406 | None | 0.024489795918367314 |
| 0.48000000000000009 | 0.009725906277630423 | 0.01148225469728601 | 0.0131332082551595 | 0.015286624203821701 | None | None | 0.015789473684210506 | 0.02962962962962961 | 0.017241379310344803 | 0.013303769401330405 | 0.0051347881899871635 | 0.0348837209302326 | 0.014117647058823493 | None | 0.0187617260787993 | 0.014475271411339 | None | 0.028571428571428602 |
| 0.5 | 0.0026525198938991998 | 0.00626304801670146 | 0.0187617260787993 | 0.007643312101910834 | None | None | 0.006578947368421051 | 0.011111111111111101 | 0.0 | 0.00665188470066519 | 0.0051347881899871635 | 0.00968992248062015 | 0.010588235294117607 | None | 0.0131332082551595 | 0.00482509047044632 | None | 0.020408163265306107 |
| 0.52 | 0.0017683465959328008 | 0.003131524008350732 | 0.007504690431519703 | 0.010191082802547798 | None | None | 0.013157894736842101 | 0.025925925925925915 | 0.0 | 0.0022172949002217312 | 0.0012836970474967904 | 0.005813953488372092 | 0.004705882352941182 | None | 0.00375234521575985 | 0.00844390832328106 | None | 0.03469387755102042 |
| 0.54 | 0.0026525198938991998 | 0.0010438413361169101 | 0.007504690431519703 | 0.003821656050955411 | None | None | 0.007894736842105256 | 0.0037037037037037017 | 0.0 | 0.009977827050997784 | 0.0 | 0.0019379844961240301 | 0.007058823529411772 | None | 0.00562851782363977 | 0.007237635705669482 | None | 0.016326530612244903 |
| 0.56000000000000005 | 0.0 | 0.0010438413361169101 | 0.0018761726078799204 | 0.002547770700636942 | None | None | 0.005263157894736842 | 0.00740740740740741 | 0.03448275862068972 | 0.0 | 0.0025673940949935818 | 0.007751937984496123 | 0.0011764705882352905 | None | 0.00375234521575985 | 0.007237635705669482 | None | 0.010204081632653106 |
| 0.58000000000000007 | 0.0008841732979664018 | 0.0010438413361169101 | 0.0018761726078799204 | 0.0 | None | None | 0.003947368421052632 | 0.0037037037037037017 | 0.0 | 0.0011086474501108604 | 0.0 | 0.00387596899224806 | 0.0011764705882352905 | None | 0.0 | 0.003618817852834741 | None | 0.00816326530612245 |
| 0.6000000000000002 | 0.0008841732979664018 | 0.0020876826722338207 | 0.00562851782363977 | 0.00127388535031847 | None | None | 0.0 | 0.011111111111111101 | 0.017241379310344803 | 0.0 | 0.0 | 0.0 | 0.0 | None | 0.00375234521575985 | 0.0012062726176115801 | None | 0.00816326530612245 |
### Chart: unc-25 A1
| Category | control_unc-25_A1_001 | control_unc-25_A1_002 | control_unc-25_A1_003 | control_unc-25_A1_004 | control_unc-25_A1_005 | control_unc-25_A1_006 | control_unc-25_A1_007 | control_unc-25_A1_008 | unc-25_A1_M101 | unc-25_A1_M102 | unc-25_A1_M104 | unc-25_A1_M106 | unc-25_REX_A1_01 | unc-25_REX_A1_02 | unc-25_REX_A1_04 | unc-25_REX_A1_06 | unc-25_REX_A1_07 | unc-25_REX_A1_09 |
|---|---|---|---|---|---|---|---|---|---|---|---|---|---|---|---|---|---|---|
| 0 | None | None | None | None | None | None | None | None | None | None | None | None | None | None | None | None | None | None |
| 2.0000000000000007E-2 | 0.04991192014092783 | 0.060559796437659 | 0.0346585117227319 | 0.0413153456998314 | 0.0343347639484979 | 0.029812606473594506 | 0.0299465240641711 | 0.03094777562862672 | 0.038212815990593806 | 0.0600315955766193 | 0.04694167852062588 | 0.0782493368700265 | 0.0439706862091939 | 0.0507757404795487 | 0.0583775587566338 | 0.0565022421524664 | 0.036257309941520516 | 0.036276522929500316 |
| 4.0000000000000015E-2 | 0.0575455079271873 | 0.0422391857506361 | 0.0316004077471967 | 0.0370994940978078 | 0.0343347639484979 | 0.0255536626916525 | 0.031016042780748706 | 0.033526756931012286 | 0.05467372134038801 | 0.048183254344391815 | 0.06614509246088188 | 0.0616710875331565 | 0.0406395736175883 | 0.04936530324400562 | 0.0515542077331312 | 0.049327354260089704 | 0.0327485380116959 | 0.0342231348391513 |
| 6.0000000000000019E-2 | 0.0504991192014093 | 0.050381679389313 | 0.0336391437308868 | 0.0295109612141653 | 0.0343347639484979 | 0.02129471890971041 | 0.018181818181818205 | 0.033526756931012286 | 0.0529100529100529 | 0.052922590837282825 | 0.04338549075391182 | 0.0583554376657825 | 0.0299800133244504 | 0.03949224259520452 | 0.03108415466262321 | 0.053811659192825115 | 0.0245614035087719 | 0.036276522929500316 |
| 8.0000000000000029E-2 | 0.04462712859659418 | 0.047837150127226516 | 0.022426095820591206 | 0.0269814502529511 | 0.035944206008583716 | 0.019591141396933607 | 0.026737967914438505 | 0.0361057382333978 | 0.05349794238683132 | 0.047393364928909935 | 0.0490753911806543 | 0.05570291777188332 | 0.0246502331778814 | 0.04372355430183362 | 0.0272934040940106 | 0.03408071748878923 | 0.017543859649122813 | 0.02737850787132101 |
| 0.1 | 0.042865531415149725 | 0.0539440203562341 | 0.018348623853211 | 0.0269814502529511 | 0.033798283261802585 | 0.0178875638841567 | 0.0128342245989305 | 0.041908446163765295 | 0.0340975896531452 | 0.03712480252764611 | 0.03769559032716932 | 0.04177718832891249 | 0.0146568954030646 | 0.03808180535966151 | 0.025018953752843107 | 0.026905829596412602 | 0.016374269005848 | 0.028062970568104008 |
| 0.12000000000000002 | 0.036406341749853216 | 0.045292620865140035 | 0.024464831804281294 | 0.0286677908937605 | 0.032725321888412005 | 0.013628620102214701 | 0.0106951871657754 | 0.03997421018697612 | 0.046443268665490874 | 0.0418641390205371 | 0.03840682788051211 | 0.045755968169761296 | 0.0153231179213857 | 0.02186177715091681 | 0.015921152388172908 | 0.021524663677130004 | 0.014035087719298199 | 0.0191649555099247 |
| 0.14000000000000001 | 0.04521432765707571 | 0.04936386768447842 | 0.019367991845056106 | 0.015177065767285 | 0.04238197424892702 | 0.0187393526405451 | 0.011764705882352905 | 0.03288201160541592 | 0.03527336860670193 | 0.02685624012638231 | 0.03627311522048362 | 0.03779840848806371 | 0.010659560293137905 | 0.015514809590973204 | 0.012888551933282805 | 0.016143497757847503 | 0.007017543859649122 | 0.01984941820670771 |
| 0.16 | 0.027598355842630692 | 0.03613231552162852 | 0.012232415902140695 | 0.0244519392917369 | 0.03862660944206011 | 0.0187393526405451 | 0.013903743315508005 | 0.03094777562862672 | 0.04232804232804231 | 0.0221169036334913 | 0.0312944523470839 | 0.0271883289124668 | 0.010659560293137905 | 0.015514809590973204 | 0.006065200909780142 | 0.008071748878923774 | 0.00935672514619883 | 0.0136892539356605 |
| 0.18000000000000005 | 0.026423957721667612 | 0.037150127226463124 | 0.0336391437308868 | 0.026138279932546398 | 0.04291845493562234 | 0.028960817717206114 | 0.0053475935828877 | 0.0361057382333978 | 0.0523221634332745 | 0.0221169036334913 | 0.0334281650071124 | 0.029177718832891202 | 0.007994670219853433 | 0.011988716502115701 | 0.011372251705837805 | 0.00896860986547085 | 0.007017543859649122 | 0.0171115674195756 |
| 0.2 | 0.04345273047563123 | 0.037150127226463124 | 0.0336391437308868 | 0.0269814502529511 | 0.0450643776824034 | 0.0383304940374787 | 0.00962566844919786 | 0.0296582849774339 | 0.06407995296884192 | 0.019747235387045807 | 0.04978662873399724 | 0.03713527851458892 | 0.011325782811459 | 0.0190409026798307 | 0.009097801364670196 | 0.00896860986547085 | 0.007017543859649122 | 0.0123203285420945 |
| 0.22 | 0.0487375220199648 | 0.0412213740458015 | 0.06931702344546384 | 0.051433389544688 | 0.05901287553648072 | 0.0417376490630324 | 0.018181818181818205 | 0.02514506769825921 | 0.06114050558494996 | 0.027646129541864115 | 0.0625889046941678 | 0.04442970822281171 | 0.019320453031312513 | 0.023977433004231292 | 0.007581501137225172 | 0.010762331838565007 | 0.014035087719298199 | 0.014373716632443499 |
| 0.24000000000000005 | 0.0487375220199648 | 0.04478371501272264 | 0.09072375127421001 | 0.0522765598650927 | 0.07832618025751073 | 0.08177172061328793 | 0.028877005347593607 | 0.033526756931012286 | 0.065843621399177 | 0.028436018957346 | 0.0633001422475107 | 0.03713527851458892 | 0.0299800133244504 | 0.03949224259520452 | 0.00530705079605762 | 0.0116591928251121 | 0.0245614035087719 | 0.021218343600273817 |
| 0.26 | 0.05578391074574282 | 0.0564885496183206 | 0.08358817533129467 | 0.06745362563237772 | 0.07081545064377683 | 0.109028960817717 | 0.04491978609625672 | 0.0438426821405545 | 0.06407995296884192 | 0.0355450236966825 | 0.06543385490753911 | 0.04310344827586208 | 0.033311125916056 | 0.04936530324400562 | 0.012130401819560306 | 0.014349775784753403 | 0.039766081871345 | 0.025325119780971905 |
| 0.28000000000000008 | 0.0616559013505578 | 0.0564885496183206 | 0.08358817533129467 | 0.0741989881956155 | 0.0842274678111588 | 0.11243611584327103 | 0.0759358288770054 | 0.0567375886524823 | 0.0634920634920635 | 0.0442338072669826 | 0.0625889046941678 | 0.04177718832891249 | 0.07395069953364423 | 0.07193229901269393 | 0.03790750568612593 | 0.0197309417040359 | 0.0538011695906433 | 0.0438056125941136 |
| 0.3000000000000001 | 0.06341749853200233 | 0.050381679389313 | 0.09174311926605508 | 0.07335581787521081 | 0.07886266094420606 | 0.08177172061328793 | 0.079144385026738 | 0.05931656995486784 | 0.065843621399177 | 0.0418641390205371 | 0.056187766714082495 | 0.051061007957559704 | 0.060626249167221896 | 0.06488011283497883 | 0.03639120545868081 | 0.04035874439461881 | 0.07368421052631581 | 0.0561259411362081 |
| 0.32000000000000012 | 0.051086318261890785 | 0.0549618320610687 | 0.0744138634046891 | 0.0775716694772344 | 0.04989270386266092 | 0.0809199318568995 | 0.11764705882352898 | 0.06898774983881373 | 0.04115226337448558 | 0.06477093206951033 | 0.039829302987197716 | 0.05238726790450934 | 0.0846102598267822 | 0.07827926657263747 | 0.0614101592115239 | 0.06278026905829605 | 0.10994152046783603 | 0.0766598220396988 |
| 0.34 | 0.04345273047563123 | 0.04885496183206112 | 0.054026503567788 | 0.06576728499156834 | 0.046673819742489284 | 0.05792163543441232 | 0.08556149732620325 | 0.09477756286266938 | 0.038212815990593806 | 0.048183254344391815 | 0.039829302987197716 | 0.046419098143236116 | 0.0752831445702865 | 0.05923836389280682 | 0.0561031084154663 | 0.06726457399103143 | 0.111111111111111 | 0.06297056810403831 |
| 0.3600000000000001 | 0.0317087492660012 | 0.037150127226463124 | 0.0407747196738022 | 0.0522765598650927 | 0.040772532188841235 | 0.05451448040885862 | 0.0684491978609626 | 0.054158607350096734 | 0.0329218106995885 | 0.042654028436018995 | 0.044807965860597404 | 0.030503978779840814 | 0.0752831445702865 | 0.058533145275035295 | 0.07960576194086431 | 0.08699551569506736 | 0.059649122807017486 | 0.0732375085557837 |
| 0.38000000000000012 | 0.0229007633587786 | 0.0315521628498728 | 0.0203873598369011 | 0.039629005059021914 | 0.023068669527896997 | 0.03918228279386711 | 0.081283422459893 | 0.042553191489361715 | 0.017636684303351 | 0.03712480252764611 | 0.019914651493598903 | 0.023209549071618006 | 0.060626249167221896 | 0.04301833568406208 | 0.0697498104624716 | 0.0699551569506726 | 0.0830409356725146 | 0.06707734428473651 |
| 0.4 | 0.02407516147974161 | 0.02239185750636132 | 0.024464831804281294 | 0.0345699831365936 | 0.016630901287553606 | 0.0187393526405451 | 0.04491978609625672 | 0.0354609929078014 | 0.00940623162845385 | 0.04107424960505528 | 0.014935988620199098 | 0.0185676392572944 | 0.05329780146568948 | 0.04583921015514813 | 0.0697498104624716 | 0.053811659192825115 | 0.0584795321637427 | 0.06981519507186863 |
| 0.4200000000000001 | 0.015267175572519104 | 0.013231552162849899 | 0.0152905198776758 | 0.0168634064080944 | 0.008047210300429179 | 0.0178875638841567 | 0.0352941176470588 | 0.0315925209542231 | 0.009994121105232219 | 0.026066350710900507 | 0.0163584637268848 | 0.01657824933687 | 0.0519653564290473 | 0.0296191819464034 | 0.0614101592115239 | 0.049327354260089704 | 0.054970760233918115 | 0.05475701574264202 |
| 0.44 | 0.007633587786259542 | 0.009160305343511449 | 0.006116207951070342 | 0.019392917369308607 | 0.009120171673819736 | 0.017035775127768306 | 0.021390374331550797 | 0.0212765957446809 | 0.0105820105820106 | 0.027646129541864115 | 0.00924608819345661 | 0.0125994694960212 | 0.0299800133244504 | 0.020451339915373817 | 0.04700530705079612 | 0.038565022421524715 | 0.035087719298245605 | 0.039014373716632404 |
| 0.46 | 0.0064591896652965415 | 0.00559796437659033 | 0.008154943934760453 | 0.013490725126475504 | 0.006974248927038634 | 0.0051107325383304885 | 0.026737967914438505 | 0.018052869116698903 | 0.00529100529100529 | 0.015797788309636705 | 0.0035561877667140817 | 0.007294429708222814 | 0.0246502331778814 | 0.013399153737658704 | 0.03790750568612593 | 0.03946188340807171 | 0.0128654970760234 | 0.025325119780971905 |
| 0.48000000000000009 | 0.007633587786259542 | 0.005089058524173034 | 0.003058103975535171 | 0.005059021922428332 | 0.0037553648068669528 | 0.004258943781942083 | 0.016042780748663114 | 0.008381689232753066 | 0.0017636684303351004 | 0.00868878357030016 | 0.002844950213371271 | 0.009283819628647215 | 0.0179880079946702 | 0.008462623413258114 | 0.017437452615617906 | 0.014349775784753403 | 0.015204678362573104 | 0.0164271047227926 |
| 0.5 | 0.002348796241926012 | 0.002035623409669209 | 0.0 | 0.005059021922428332 | 0.00321888412017167 | 0.00255536626916525 | 0.007486631016042782 | 0.007092198581560282 | 0.00235155790711346 | 0.005529225908372832 | 0.0007112375533428172 | 0.006631299734748015 | 0.013324450366422408 | 0.003526093088857552 | 0.012888551933282805 | 0.008071748878923774 | 0.003508771929824561 | 0.013004791238877508 |
| 0.52 | 0.0017615971814445104 | 0.0025445292620865124 | 0.0010193679918450605 | 0.004215851602023612 | 0.002145922746781122 | 0.00255536626916525 | 0.0106951871657754 | 0.003868471953578341 | 0.0 | 0.003949447077409162 | 0.0007112375533428172 | 0.00397877984084881 | 0.0066622251832111935 | 0.005641748942172073 | 0.008339651250947698 | 0.00896860986547085 | 0.004678362573099422 | 0.009582477754962354 |
| 0.54 | 0.0005871990604815033 | 0.0015267175572519105 | 0.00203873598369011 | 0.00337268128161889 | 0.00107296137339056 | 0.00340715502555366 | 0.0053475935828877 | 0.0032237266279819534 | 0.0 | 0.00710900473933649 | 0.0007112375533428172 | 0.007294429708222814 | 0.003997335109926722 | 0.000705218617771509 | 0.006065200909780142 | 0.00269058295964126 | 0.004678362573099422 | 0.005475701574264203 |
| 0.56000000000000005 | 0.0 | 0.0 | 0.0010193679918450605 | 0.0 | 0.0005364806866952788 | 0.0 | 0.00427807486631016 | 0.0006447453255963893 | 0.0 | 0.002369668246445501 | 0.0 | 0.0006631299734748015 | 0.004663557628247828 | 0.0049365303244005634 | 0.002274450341167551 | 0.0035874439461883417 | 0.003508771929824561 | 0.006160164271047228 |
| 0.58000000000000007 | 0.0 | 0.0005089058524173033 | 0.0 | 0.0008431703204047225 | 0.0016094420600858408 | 0.0008517887563884164 | 0.0010695187165775401 | 0.0025789813023855616 | 0.0 | 0.002369668246445501 | 0.0007112375533428172 | 0.0006631299734748015 | 0.0033311125916056002 | 0.0 | 0.003032600454890069 | 0.007174887892376682 | 0.0 | 0.0013689253935660504 |
| 0.6000000000000002 | 0.0005871990604815033 | 0.0005089058524173033 | 0.0010193679918450605 | 0.0008431703204047225 | 0.0 | 0.0008517887563884164 | 0.0010695187165775401 | 0.0006447453255963893 | 0.0 | 0.0007898894154818334 | 0.0014224751066856305 | 0.0006631299734748015 | 0.0006662225183211195 | 0.0 | 0.0037907505686125926 | 0.00448430493273543 | 0.0 | 0.000684462696783025 |
### Chart: unc-25 A2
| Category | unc-25_A2_40_30_01 | unc-25_A2_40_30_02 | unc-25_A2_40_30_03 | unc-25_REX_A2_03 | unc-25_REX_A2_04 | unc-25_REX_A2_05 | unc-25_REX_A2_06 | unc-25_REX_A2_08 | unc-25_REX_A2_09 | unc-25_REX_A2_10 |
|---|---|---|---|---|---|---|---|---|---|---|
| 0 | None | None | None | None | None | None | None | None | None | None |
| 2.0000000000000007E-2 | 0.0513100436681223 | 0.053254437869822514 | 0.07272727272727271 | 0.0450070323488045 | 0.04850088183421523 | 0.0619122257053292 | 0.038009049773755715 | 0.06361829025844933 | 0.08158368326334726 | 0.0774487471526196 |
| 4.0000000000000015E-2 | 0.0513100436681223 | 0.03254437869822492 | 0.04675324675324682 | 0.04781997187060482 | 0.037918871252204604 | 0.04231974921630091 | 0.034389140271493215 | 0.044731610337972225 | 0.06538692261547693 | 0.055239179954441914 |
| 6.0000000000000019E-2 | 0.0556768558951965 | 0.04339250493096654 | 0.031168831168831197 | 0.0443037974683544 | 0.0317460317460317 | 0.04231974921630091 | 0.02352941176470589 | 0.039761431411530795 | 0.07078584283143373 | 0.05637813211845099 |
| 8.0000000000000029E-2 | 0.049126637554585136 | 0.0335305719921105 | 0.0441558441558442 | 0.0330520393811533 | 0.037918871252204604 | 0.029780564263322894 | 0.02262443438914029 | 0.0407554671968191 | 0.031793641271745714 | 0.035307517084282515 |
| 0.1 | 0.042576419213973815 | 0.02662721893491121 | 0.0363636363636364 | 0.031645569620253215 | 0.0229276895943563 | 0.0235109717868339 | 0.02262443438914029 | 0.021868787276342 | 0.0365926814637073 | 0.0347380410022779 |
| 0.12000000000000002 | 0.02510917030567691 | 0.011834319526627201 | 0.015584415584415605 | 0.025316455696202493 | 0.013227513227513204 | 0.013322884012539208 | 0.020814479638009 | 0.0178926441351889 | 0.025794841031793598 | 0.024487471526195913 |
| 0.14000000000000001 | 0.026200873362445407 | 0.0157790927021696 | 0.015584415584415605 | 0.020393811533052 | 0.00970017636684303 | 0.010188087774294698 | 0.010859728506787304 | 0.014910536779324099 | 0.0215956808638272 | 0.0199316628701595 |
| 0.16 | 0.017467248908296894 | 0.0108481262327416 | 0.020779220779220814 | 0.009845288326300997 | 0.014991181657848305 | 0.005485893416927902 | 0.00633484162895928 | 0.014910536779324099 | 0.014997000599880001 | 0.012528473804100201 |
| 0.18000000000000005 | 0.0141921397379913 | 0.0108481262327416 | 0.015584415584415605 | 0.007032348804500704 | 0.011463844797178107 | 0.008620689655172415 | 0.010859728506787304 | 0.009940357852882704 | 0.013197360527894395 | 0.011958997722095698 |
| 0.2 | 0.0185589519650655 | 0.008875739644970416 | 0.018181818181818205 | 0.012658227848101299 | 0.013227513227513204 | 0.00940438871473354 | 0.007239819004524894 | 0.009940357852882704 | 0.018596280743851203 | 0.015375854214123007 |
| 0.22 | 0.0163755458515284 | 0.023668639053254392 | 0.020779220779220814 | 0.023206751054852294 | 0.016754850088183407 | 0.00470219435736677 | 0.007239819004524894 | 0.010934393638171 | 0.014397120575884798 | 0.02334851936218679 |
| 0.24000000000000005 | 0.026200873362445407 | 0.0335305719921105 | 0.04155844155844162 | 0.0414908579465541 | 0.02469135802469141 | 0.007053291536050162 | 0.009049773755656106 | 0.025844930417495013 | 0.026994601079784 | 0.0313211845102506 |
| 0.26 | 0.0502183406113537 | 0.0522682445759369 | 0.05454545454545449 | 0.069620253164557 | 0.03262786596119932 | 0.0109717868338558 | 0.02262443438914029 | 0.03280318091451291 | 0.028194361127774407 | 0.04384965831435078 |
| 0.28000000000000008 | 0.0611353711790393 | 0.0670611439842209 | 0.09870129870129873 | 0.0815752461322082 | 0.0564373897707231 | 0.017241379310344803 | 0.04072398190045252 | 0.05069582504970181 | 0.04319136172765448 | 0.05296127562642372 |
| 0.3000000000000001 | 0.0447598253275109 | 0.0976331360946746 | 0.0649350649350649 | 0.0963431786216596 | 0.07319223985890655 | 0.03448275862068972 | 0.06063348416289591 | 0.06759443339960243 | 0.0353929214157169 | 0.0586560364464692 |
| 0.32000000000000012 | 0.0600436681222707 | 0.09960552268244585 | 0.0597402597402597 | 0.0893108298171589 | 0.0687830687830688 | 0.04310344827586208 | 0.061538461538461514 | 0.0715705765407555 | 0.052789442111577704 | 0.04783599088838272 |
| 0.34 | 0.0513100436681223 | 0.07593688362919133 | 0.07272727272727271 | 0.0717299578059072 | 0.0617283950617284 | 0.058777429467084585 | 0.06877828054298643 | 0.06958250497017893 | 0.058188362327534486 | 0.0518223234624146 |
| 0.3600000000000001 | 0.06550218340611351 | 0.08875739644970415 | 0.04155844155844162 | 0.0618846694796062 | 0.0529100529100529 | 0.058777429467084585 | 0.09230769230769234 | 0.06163021868787278 | 0.05098980203959208 | 0.04783599088838272 |
| 0.38000000000000012 | 0.0283842794759825 | 0.054240631163708114 | 0.038961038961039 | 0.038677918424753925 | 0.0634920634920635 | 0.0540752351097179 | 0.08235294117647066 | 0.05467196819085487 | 0.04979004199160175 | 0.03701594533029611 |
| 0.4 | 0.03930131004366812 | 0.0335305719921105 | 0.0363636363636364 | 0.0267229254571027 | 0.047619047619047616 | 0.0673981191222571 | 0.0778280542986425 | 0.0427435387673956 | 0.026994601079784 | 0.027904328018223217 |
| 0.4200000000000001 | 0.0185589519650655 | 0.031558185404339315 | 0.007792207792207792 | 0.0189873417721519 | 0.0335097001763668 | 0.04937304075235111 | 0.056108597285067896 | 0.025844930417495013 | 0.025194961007798392 | 0.021070615034168606 |
| 0.44 | 0.0141921397379913 | 0.0138067061143984 | 0.020779220779220814 | 0.010548523206751105 | 0.018518518518518507 | 0.04467084639498428 | 0.038009049773755715 | 0.025844930417495013 | 0.0197960407918416 | 0.026765375854214117 |
| 0.46 | 0.010917030567685601 | 0.011834319526627201 | 0.007792207792207792 | 0.007735583684950774 | 0.022045855379188708 | 0.0235109717868339 | 0.031674208144796406 | 0.01988071570576541 | 0.0107978404319136 | 0.011958997722095698 |
| 0.48000000000000009 | 0.010917030567685601 | 0.005917159763313612 | 0.005194805194805191 | 0.008438818565400838 | 0.016754850088183407 | 0.024294670846395 | 0.016289592760181 | 0.010934393638171 | 0.007798440311937612 | 0.0108200455580866 |
| 0.5 | 0.006550218340611352 | 0.005917159763313612 | 0.007792207792207792 | 0.00281293952180028 | 0.0141093474426808 | 0.013322884012539208 | 0.013574660633484201 | 0.006958250497017892 | 0.0017996400719856006 | 0.00740318906605923 |
| 0.52 | 0.00436681222707424 | 0.0029585798816568008 | 0.0 | 0.0007032348804500703 | 0.007936507936507941 | 0.01802507836990601 | 0.008144796380090498 | 0.006958250497017892 | 0.0041991601679664085 | 0.006833712984054676 |
| 0.54 | 0.00545851528384279 | 0.0019723865877712015 | 0.0 | 0.0007032348804500703 | 0.007936507936507941 | 0.0109717868338558 | 0.007239819004524894 | 0.010934393638171 | 0.0041991601679664085 | 0.002847380410022781 |
| 0.56000000000000005 | 0.003275109170305681 | 0.0 | 0.0 | 0.00281293952180028 | 0.00529100529100529 | 0.007836990595611283 | 0.0018099547511312205 | 0.00397614314115308 | 0.0017996400719856006 | 0.003986332574031892 |
| 0.58000000000000007 | 0.002183406113537121 | 0.0 | 0.0 | 0.0021097046413502112 | 0.004409171075837742 | 0.00470219435736677 | 0.003619909502262442 | 0.002982107355864811 | 0.0017996400719856006 | 0.000569476082004556 |
| 0.6000000000000002 | 0.0 | 0.0 | 0.0 | 0.0007032348804500703 | 0.002645502645502652 | 0.0015673981191222605 | 0.003619909502262442 | 0.0009940357852882705 | 0.0 | 0.001138952164009111 |
### Chart: unc-25 A3
| Category | unc-25_A3_40_30_01 | unc-25_A3_40_30_03 | unc-25_A3_40_30_2 | unc-25_A3_40_30_4 | unc-25_REX_A3_01 | unc-25_REX_A3_03 | unc-25_REX_A3_04 | unc-25_REX_A3_05 | unc-25_REX_A3_06 | unc-25_REX_A3_08 |
|---|---|---|---|---|---|---|---|---|---|---|
| 0 | None | None | None | None | None | None | None | None | None | None |
| 2.0000000000000007E-2 | 0.07032057911065151 | 0.0743099787685775 | 0.09036144578313256 | 0.05752212389380532 | 0.056170886075949396 | 0.0735963581183612 | 0.0736145574855252 | 0.08140350877192978 | 0.10023866348448703 | 0.07173601147776183 |
| 4.0000000000000015E-2 | 0.056876938986556415 | 0.052016985138004235 | 0.06024096385542172 | 0.05752212389380532 | 0.0474683544303798 | 0.0584218512898331 | 0.05789909015715472 | 0.0680701754385965 | 0.09228321400159101 | 0.0616929698708752 |
| 6.0000000000000019E-2 | 0.047569803516029 | 0.0435244161358811 | 0.05722891566265057 | 0.04159292035398232 | 0.0522151898734177 | 0.050075872534142585 | 0.05789909015715472 | 0.06105263157894742 | 0.0612569610182975 | 0.055236728837876635 |
| 8.0000000000000029E-2 | 0.04136504653567739 | 0.0339702760084926 | 0.051204819277108383 | 0.04690265486725663 | 0.0308544303797468 | 0.04097116843702578 | 0.04631927212572372 | 0.0575438596491228 | 0.0485282418456643 | 0.0380200860832138 |
| 0.1 | 0.034126163391933785 | 0.0339702760084926 | 0.035140562248996 | 0.0345132743362832 | 0.01977848101265821 | 0.042488619119878626 | 0.03639371381306871 | 0.05684210526315787 | 0.034208432776451914 | 0.0337159253945481 |
| 0.12000000000000002 | 0.024819027921406406 | 0.02547770700636942 | 0.027108433734939798 | 0.0292035398230088 | 0.0134493670886076 | 0.03490136570561461 | 0.02646815550041361 | 0.032280701754386 | 0.019888623707239508 | 0.024390243902439 |
| 0.14000000000000001 | 0.015511892450878998 | 0.02229299363057321 | 0.023092369477911608 | 0.019469026548672608 | 0.0102848101265823 | 0.023520485584218494 | 0.0181968569065343 | 0.018947368421052605 | 0.019093078758949902 | 0.01506456241033 |
| 0.16 | 0.0196483971044467 | 0.015923566878980902 | 0.011044176706827308 | 0.021238938053097314 | 0.0110759493670886 | 0.015933232169954497 | 0.016542597187758506 | 0.0252631578947368 | 0.0167064439140811 | 0.01936872309899571 |
| 0.18000000000000005 | 0.016546018614270908 | 0.020169851380042493 | 0.014056224899598398 | 0.01858407079646021 | 0.004746835443037971 | 0.012898330804248898 | 0.00909842845326716 | 0.0252631578947368 | 0.011137629276054101 | 0.025107604017216602 |
| 0.2 | 0.025853154084798307 | 0.0435244161358811 | 0.0180722891566265 | 0.028318584070796484 | 0.007911392405063295 | 0.0144157814871017 | 0.011579818031430903 | 0.029473684210526308 | 0.014319809069212406 | 0.0215208034433286 |
| 0.22 | 0.038262668045501595 | 0.054140127388534985 | 0.023092369477911608 | 0.028318584070796484 | 0.014240506329113901 | 0.0174506828528073 | 0.0181968569065343 | 0.03578947368421052 | 0.019888623707239508 | 0.04232424677187948 |
| 0.24000000000000005 | 0.06204756980351602 | 0.058386411889596646 | 0.04116465863453821 | 0.044247787610619496 | 0.025316455696202493 | 0.0204855842185129 | 0.02646815550041361 | 0.03719298245614041 | 0.024661893396976907 | 0.0595408895265423 |
| 0.26 | 0.0672182006204757 | 0.07749469214437375 | 0.05220883534136549 | 0.06017699115044253 | 0.033227848101265806 | 0.025796661608497688 | 0.03391232423490489 | 0.04771929824561402 | 0.03023070803500401 | 0.07173601147776183 |
| 0.28000000000000008 | 0.08479834539813867 | 0.0711252653927813 | 0.055220883534136504 | 0.06637168141592922 | 0.05379746835443043 | 0.043247344461304967 | 0.042183622828784115 | 0.040000000000000015 | 0.03500397772474142 | 0.0681492109038737 |
| 0.3000000000000001 | 0.04136504653567739 | 0.0753715498938429 | 0.049196787148594434 | 0.06725663716814165 | 0.06803797468354433 | 0.04400606980273144 | 0.04383788254755998 | 0.05192982456140348 | 0.0461416070007955 | 0.0681492109038737 |
| 0.32000000000000012 | 0.0537745604963806 | 0.058386411889596646 | 0.040160642570281097 | 0.056637168141592885 | 0.08227848101265818 | 0.042488619119878626 | 0.05045492142266342 | 0.0385964912280702 | 0.0334128878281623 | 0.0681492109038737 |
| 0.34 | 0.04136504653567739 | 0.0339702760084926 | 0.04518072289156631 | 0.04778761061946902 | 0.07357594936708857 | 0.05386949924127472 | 0.0446650124069479 | 0.0343859649122807 | 0.03739061256961022 | 0.0401721664275466 |
| 0.3600000000000001 | 0.03205791106514992 | 0.0307855626326964 | 0.0331325301204819 | 0.027433628318584116 | 0.07041139240506332 | 0.050075872534142585 | 0.05376344086021512 | 0.0385964912280702 | 0.028639618138424812 | 0.0401721664275466 |
| 0.38000000000000012 | 0.028955532574974113 | 0.019108280254777107 | 0.022088353413654616 | 0.036283185840708006 | 0.056170886075949396 | 0.0455235204855842 | 0.0397022332506203 | 0.020350877192982494 | 0.02704852824184569 | 0.0286944045911047 |
| 0.4 | 0.016546018614270908 | 0.014861995753715504 | 0.0180722891566265 | 0.0176991150442478 | 0.04667721518987338 | 0.04476479514415782 | 0.033085194375517 | 0.017543859649122813 | 0.014319809069212406 | 0.0186513629842181 |
| 0.4200000000000001 | 0.0124095139607032 | 0.013800424628450108 | 0.015060240963855399 | 0.014159292035398192 | 0.025316455696202493 | 0.028072837632776914 | 0.03391232423490489 | 0.0154385964912281 | 0.018297533810660307 | 0.00932568149210905 |
| 0.44 | 0.007238883143743544 | 0.0053078556263269575 | 0.011044176706827308 | 0.014159292035398192 | 0.0134493670886076 | 0.03490136570561461 | 0.02481389578163771 | 0.012631578947368407 | 0.010342084327764501 | 0.007890961262553804 |
| 0.46 | 0.0062047569803516025 | 0.002123142250530791 | 0.013052208835341398 | 0.0150442477876106 | 0.015031645569620299 | 0.0166919575113809 | 0.0124069478908189 | 0.004210526315789472 | 0.0063643595863166315 | 0.008608321377331418 |
| 0.48000000000000009 | 0.0010341261633919304 | 0.004246284501061572 | 0.006024096385542173 | 0.00619469026548673 | 0.0102848101265823 | 0.0136570561456753 | 0.0157154673283706 | 0.003508771929824561 | 0.0063643595863166315 | 0.00430416068866571 |
| 0.5 | 0.0010341261633919304 | 0.0 | 0.0040160642570281095 | 0.005309734513274342 | 0.003955696202531652 | 0.011380880121396101 | 0.014061207609594699 | 0.004210526315789472 | 0.003977724741447894 | 0.00286944045911047 |
| 0.52 | 0.0010341261633919304 | 0.002123142250530791 | 0.0040160642570281095 | 0.0008849557522123897 | 0.003955696202531652 | 0.006069802731411232 | 0.00661703887510339 | 0.0014035087719298201 | 0.00238663484486874 | 0.00143472022955524 |
| 0.54 | 0.00206825232678387 | 0.0 | 0.0010040160642570308 | 0.0017699115044247805 | 0.003164556962025322 | 0.00682852807283763 | 0.003308519437551701 | 0.004912280701754392 | 0.00159108989657916 | 0.0007173601147776184 |
| 0.56000000000000005 | 0.0 | 0.0 | 0.002008032128514061 | 0.0 | 0.0015822784810126606 | 0.003034901365705611 | 0.0 | 0.0007017543859649122 | 0.0007955449482895785 | 0.0 |
| 0.58000000000000007 | 0.0010341261633919304 | 0.0 | 0.0 | 0.0 | 0.0007911392405063296 | 0.00227617602427921 | 0.0024813895781637717 | 0.0 | 0.00159108989657916 | 0.0007173601147776184 |
| 0.6000000000000002 | 0.0010341261633919304 | 0.0 | 0.002008032128514061 | 0.0008849557522123897 | 0.0015822784810126606 | 0.0037936267071320227 | 0.0 | 0.0007017543859649122 | 0.0 | 0.0 |
### Chart: unc-25 A5
| Category | unc-25_A5_001 | unc-25_A5_002 | unc-25_A5_003 | unc-25_A5_004 | unc-25_A5_006 | unc-25_A5_40_30_01 | unc-25_A5_40_30_03 | unc-25_A5_40_30_04 | unc-25_A5_M102 | unc-25_A5_M103 | unc-25_A5_M105 | unc-25_A5_M108 | unc-25_A5_M109 | unc-25_A5_M110 | unc-25_A5_M111 | unc-25_REX_A5_01 | unc-25_REX_A5_02 |
|---|---|---|---|---|---|---|---|---|---|---|---|---|---|---|---|---|---|
| 0 | None | None | None | None | None | None | None | None | None | None | None | None | None | None | None | None | None |
| 2.0000000000000007E-2 | 0.08964451313755803 | 0.0640113798008535 | 0.054698457223001436 | 0.0610412926391382 | 0.09551656920077974 | 0.0997679814385151 | 0.0876923076923077 | 0.08421052631578953 | 0.05585106382978721 | 0.07995365005793745 | 0.0780254777070064 | 0.0792792792792793 | 0.11347517730496498 | 0.0706605222734255 | 0.0819112627986348 | 0.0933165195460277 | 0.10769230769230798 |
| 4.0000000000000015E-2 | 0.0510046367851623 | 0.06116642958748222 | 0.0476858345021038 | 0.08438061041292634 | 0.05653021442495132 | 0.09048723897911824 | 0.0784615384615385 | 0.06842105263157897 | 0.06117021276595737 | 0.07184241019698731 | 0.07165605095541401 | 0.0702702702702703 | 0.08156028368794328 | 0.05837173579109058 | 0.052901023890785014 | 0.07692307692307691 | 0.0788461538461538 |
| 6.0000000000000019E-2 | 0.054095826893353904 | 0.04694167852062588 | 0.046283309957924304 | 0.055655296229802496 | 0.0662768031189084 | 0.06496519721577733 | 0.061538461538461514 | 0.04912280701754392 | 0.0531914893617021 | 0.05561993047508691 | 0.04458598726114652 | 0.05405405405405411 | 0.07624113475177297 | 0.0522273425499232 | 0.054607508532423216 | 0.06431273644388404 | 0.07115384615384623 |
| 8.0000000000000029E-2 | 0.040185471406491514 | 0.04125177809388338 | 0.0406732117812062 | 0.04488330341113112 | 0.04483430799220272 | 0.0324825986078886 | 0.0446153846153846 | 0.04912280701754392 | 0.0412234042553191 | 0.05330243337195833 | 0.055732484076433136 | 0.045045045045045 | 0.049645390070921995 | 0.030721966205837198 | 0.03242320819112631 | 0.03656998738965951 | 0.0721153846153846 |
| 0.1 | 0.0370942812982998 | 0.025604551920341397 | 0.046283309957924304 | 0.039497307001795316 | 0.027290448343079914 | 0.0324825986078886 | 0.040000000000000015 | 0.0333333333333333 | 0.03058510638297871 | 0.040556199304750885 | 0.02388535031847131 | 0.028828828828828798 | 0.042553191489361715 | 0.021505376344086 | 0.03242320819112631 | 0.031525851197982284 | 0.042307692307692324 |
| 0.12000000000000002 | 0.023183925811437398 | 0.02275960170697011 | 0.022440392706872418 | 0.017953321364452407 | 0.0253411306042885 | 0.027842227378190313 | 0.0430769230769231 | 0.026315789473684202 | 0.0159574468085106 | 0.0289687137891078 | 0.02707006369426751 | 0.030630630630630602 | 0.03368794326241131 | 0.0230414746543779 | 0.02047781569965871 | 0.02269861286254731 | 0.0317307692307692 |
| 0.14000000000000001 | 0.020092735703245802 | 0.0170697012802276 | 0.03225806451612901 | 0.0269299820466786 | 0.0155945419103314 | 0.023201856148491892 | 0.0323076923076923 | 0.0105263157894737 | 0.0159574468085106 | 0.024333719582850518 | 0.011146496815286601 | 0.014414414414414399 | 0.0195035460992908 | 0.00768049155145929 | 0.017064846416382305 | 0.02143757881462801 | 0.022115384615384606 |
| 0.16 | 0.0185471406491499 | 0.029871977240398313 | 0.0168302945301543 | 0.017953321364452407 | 0.017543859649122813 | 0.00464037122969838 | 0.030769230769230802 | 0.017543859649122813 | 0.018617021276595706 | 0.015063731170336 | 0.015923566878980902 | 0.021621621621621602 | 0.0177304964539007 | 0.00921658986175115 | 0.03242320819112631 | 0.013871374527112205 | 0.014423076923076898 |
| 0.18000000000000005 | 0.023183925811437398 | 0.025604551920341397 | 0.0154277699859748 | 0.010771992818671498 | 0.0155945419103314 | 0.0162412993039443 | 0.029230769230769206 | 0.014035087719298199 | 0.00664893617021277 | 0.0173812282734647 | 0.0143312101910828 | 0.0162162162162162 | 0.0159574468085106 | 0.012288786482334899 | 0.029010238907849806 | 0.012610340479192898 | 0.0211538461538462 |
| 0.2 | 0.0247295208655332 | 0.01849217638691321 | 0.039270687237026605 | 0.014362657091561901 | 0.037037037037037014 | 0.013921113689095104 | 0.033846153846153804 | 0.0245614035087719 | 0.023936170212766002 | 0.02780996523754352 | 0.02388535031847131 | 0.018018018018018007 | 0.049645390070921995 | 0.018433179723502308 | 0.029010238907849806 | 0.02143757881462801 | 0.013461538461538509 |
| 0.22 | 0.044822256568779 | 0.027027027027027015 | 0.029453015427770027 | 0.016157989228007208 | 0.029239766081871312 | 0.020881670533642708 | 0.050769230769230816 | 0.017543859649122813 | 0.03989361702127662 | 0.03012746234067209 | 0.02229299363057321 | 0.019819819819819808 | 0.030141843971631208 | 0.043010752688171984 | 0.025597269624573413 | 0.03656998738965951 | 0.016346153846153805 |
| 0.24000000000000005 | 0.0494590417310665 | 0.0284495021337127 | 0.05189340813464242 | 0.0251346499102334 | 0.017543859649122813 | 0.027842227378190313 | 0.0538461538461538 | 0.0210526315789474 | 0.0518617021276596 | 0.035921205098493614 | 0.04936305732484084 | 0.04684684684684682 | 0.03368794326241131 | 0.0384024577572965 | 0.04436860068259392 | 0.02648171500630522 | 0.032692307692307715 |
| 0.26 | 0.0602782071097372 | 0.027027027027027015 | 0.056100981767180896 | 0.0251346499102334 | 0.0233918128654971 | 0.04872389791183292 | 0.0492307692307692 | 0.035087719298245605 | 0.0664893617021277 | 0.0393974507531866 | 0.054140127388534985 | 0.04684684684684682 | 0.049645390070921995 | 0.0552995391705069 | 0.03242320819112631 | 0.02269861286254731 | 0.027884615384615417 |
| 0.28000000000000008 | 0.0510046367851623 | 0.0341394025604552 | 0.06311360448807851 | 0.02333931777378821 | 0.04288499025341134 | 0.0324825986078886 | 0.03692307692307691 | 0.052631578947368404 | 0.0824468085106383 | 0.044032444959443855 | 0.0573248407643312 | 0.0504504504504504 | 0.026595744680851106 | 0.0445468509984639 | 0.04607508532423209 | 0.029003783102143802 | 0.026923076923076914 |
| 0.3000000000000001 | 0.0479134466769706 | 0.0483641536273115 | 0.06311360448807851 | 0.0215439856373429 | 0.0331384015594542 | 0.0464037122969838 | 0.033846153846153804 | 0.0385964912280702 | 0.05585106382978721 | 0.044032444959443855 | 0.054140127388534985 | 0.03963963963963961 | 0.010638297872340394 | 0.06605222734254992 | 0.037542662116041 | 0.029003783102143802 | 0.026923076923076914 |
| 0.32000000000000012 | 0.0324574961360124 | 0.0483641536273115 | 0.060308555399719486 | 0.037701974865350124 | 0.02144249512670571 | 0.027842227378190313 | 0.029230769230769206 | 0.0385964912280702 | 0.06117021276595737 | 0.0509849362688297 | 0.054140127388534985 | 0.0684684684684685 | 0.010638297872340394 | 0.07219662058371742 | 0.04266211604095562 | 0.03783102143757881 | 0.026923076923076914 |
| 0.34 | 0.026275115919629128 | 0.0312944523470839 | 0.0434782608695652 | 0.0251346499102334 | 0.019493177387914208 | 0.037122969837587 | 0.013846153846153805 | 0.028070175438596506 | 0.04388297872340431 | 0.032444959443800714 | 0.035031847133758016 | 0.0324324324324324 | 0.014184397163120598 | 0.0522273425499232 | 0.03583617747440272 | 0.0353089533417402 | 0.030769230769230802 |
| 0.3600000000000001 | 0.026275115919629128 | 0.029871977240398313 | 0.0434782608695652 | 0.014362657091561901 | 0.0253411306042885 | 0.0417633410672854 | 0.016923076923076902 | 0.028070175438596506 | 0.02260638297872342 | 0.02780996523754352 | 0.03025477707006372 | 0.025225225225225214 | 0.00886524822695037 | 0.047619047619047616 | 0.037542662116041 | 0.0201765447667087 | 0.02307692307692311 |
| 0.38000000000000012 | 0.026275115919629128 | 0.03271692745376961 | 0.0252454417952314 | 0.03231597845601442 | 0.0233918128654971 | 0.00232018561484919 | 0.009230769230769237 | 0.026315789473684202 | 0.018617021276595706 | 0.019698725376593305 | 0.0207006369426752 | 0.025225225225225214 | 0.00177304964539007 | 0.013824884792626705 | 0.017064846416382305 | 0.0163934426229508 | 0.015384615384615405 |
| 0.4 | 0.0185471406491499 | 0.0284495021337127 | 0.02103786816269281 | 0.0215439856373429 | 0.027290448343079914 | 0.011600928074245901 | 0.00615384615384615 | 0.017543859649122813 | 0.0159574468085106 | 0.01622247972190031 | 0.019108280254777107 | 0.021621621621621602 | 0.010638297872340394 | 0.018433179723502308 | 0.02047781569965871 | 0.018915510718789406 | 0.0125 |
| 0.4200000000000001 | 0.0185471406491499 | 0.027027027027027015 | 0.012622720897615708 | 0.008976660682226219 | 0.0155945419103314 | 0.006960556844547562 | 0.009230769230769237 | 0.0105263157894737 | 0.010638297872340394 | 0.008111239860950168 | 0.00955414012738853 | 0.0162162162162162 | 0.003546099290780141 | 0.016897081413210408 | 0.008532423208191134 | 0.012610340479192898 | 0.013461538461538509 |
| 0.44 | 0.00463678516228748 | 0.015647226173542 | 0.012622720897615708 | 0.007181328545780973 | 0.017543859649122813 | 0.00232018561484919 | 0.0015384615384615408 | 0.017543859649122813 | 0.00531914893617021 | 0.011587485515643104 | 0.007961783439490453 | 0.010810810810810801 | 0.0 | 0.012288786482334899 | 0.006825938566552898 | 0.0113493064312736 | 0.008653846153846156 |
| 0.46 | 0.00618238021638331 | 0.0170697012802276 | 0.00561009817671809 | 0.001795332136445241 | 0.01364522417154 | 0.00464037122969838 | 0.003076923076923081 | 0.003508771929824561 | 0.013297872340425504 | 0.010428736964078799 | 0.0015923566878980901 | 0.009009009009009018 | 0.003546099290780141 | 0.010752688172042998 | 0.008532423208191134 | 0.002522068095838589 | 0.008653846153846156 |
| 0.48000000000000009 | 0.0154559505409583 | 0.014224751066856304 | 0.009817671809256663 | 0.0125673249551167 | 0.0116959064327485 | 0.0 | 0.004615384615384622 | 0.005263157894736842 | 0.0026595744680851107 | 0.00347624565469293 | 0.004777070063694273 | 0.0018018018018018005 | 0.0 | 0.00768049155145929 | 0.0017064846416382305 | 0.002522068095838589 | 0.00288461538461538 |
| 0.5 | 0.00463678516228748 | 0.015647226173542 | 0.008415147265077147 | 0.003590664272890482 | 0.007797270955165692 | 0.0 | 0.0 | 0.005263157894736842 | 0.00531914893617021 | 0.0 | 0.0015923566878980901 | 0.0036036036036036 | 0.00177304964539007 | 0.0015360983102918604 | 0.005119453924914683 | 0.00126103404791929 | 0.0019230769230769212 |
| 0.52 | 0.00618238021638331 | 0.005689900426742531 | 0.00140252454417952 | 0.003590664272890482 | 0.0155945419103314 | 0.0 | 0.0 | 0.007017543859649122 | 0.003989361702127662 | 0.00347624565469293 | 0.0 | 0.0018018018018018005 | 0.0 | 0.00460829493087558 | 0.0017064846416382305 | 0.002522068095838589 | 0.00288461538461538 |
| 0.54 | 0.00618238021638331 | 0.002844950213371271 | 0.008415147265077147 | 0.001795332136445241 | 0.007797270955165692 | 0.00232018561484919 | 0.0015384615384615408 | 0.003508771929824561 | 0.0013297872340425506 | 0.0023174971031286198 | 0.0015923566878980901 | 0.0018018018018018005 | 0.0 | 0.0015360983102918604 | 0.0017064846416382305 | 0.002522068095838589 | 0.00288461538461538 |
| 0.56000000000000005 | 0.0 | 0.0042674253200568986 | 0.004207573632538572 | 0.003590664272890482 | 0.007797270955165692 | 0.0 | 0.0 | 0.0 | 0.0 | 0.0 | 0.0 | 0.0 | 0.003546099290780141 | 0.0015360983102918604 | 0.0 | 0.005044136191677181 | 0.0009615384615384628 |
| 0.58000000000000007 | 0.0015455950540958301 | 0.0014224751066856305 | 0.004207573632538572 | 0.003590664272890482 | 0.00584795321637427 | 0.00232018561484919 | 0.0 | 0.0 | 0.0013297872340425506 | 0.0 | 0.0 | 0.0018018018018018005 | 0.0 | 0.0 | 0.0 | 0.0 | 0.0009615384615384628 |
| 0.6000000000000002 | 0.003091190108191652 | 0.0014224751066856305 | 0.00140252454417952 | 0.001795332136445241 | 0.001949317738791421 | 0.00232018561484919 | 0.0 | 0.005263157894736842 | 0.0 | 0.0 | 0.0015923566878980901 | 0.0018018018018018005 | 0.0 | 0.0 | 0.0 | 0.0 | 0.0 |

## Slide 2
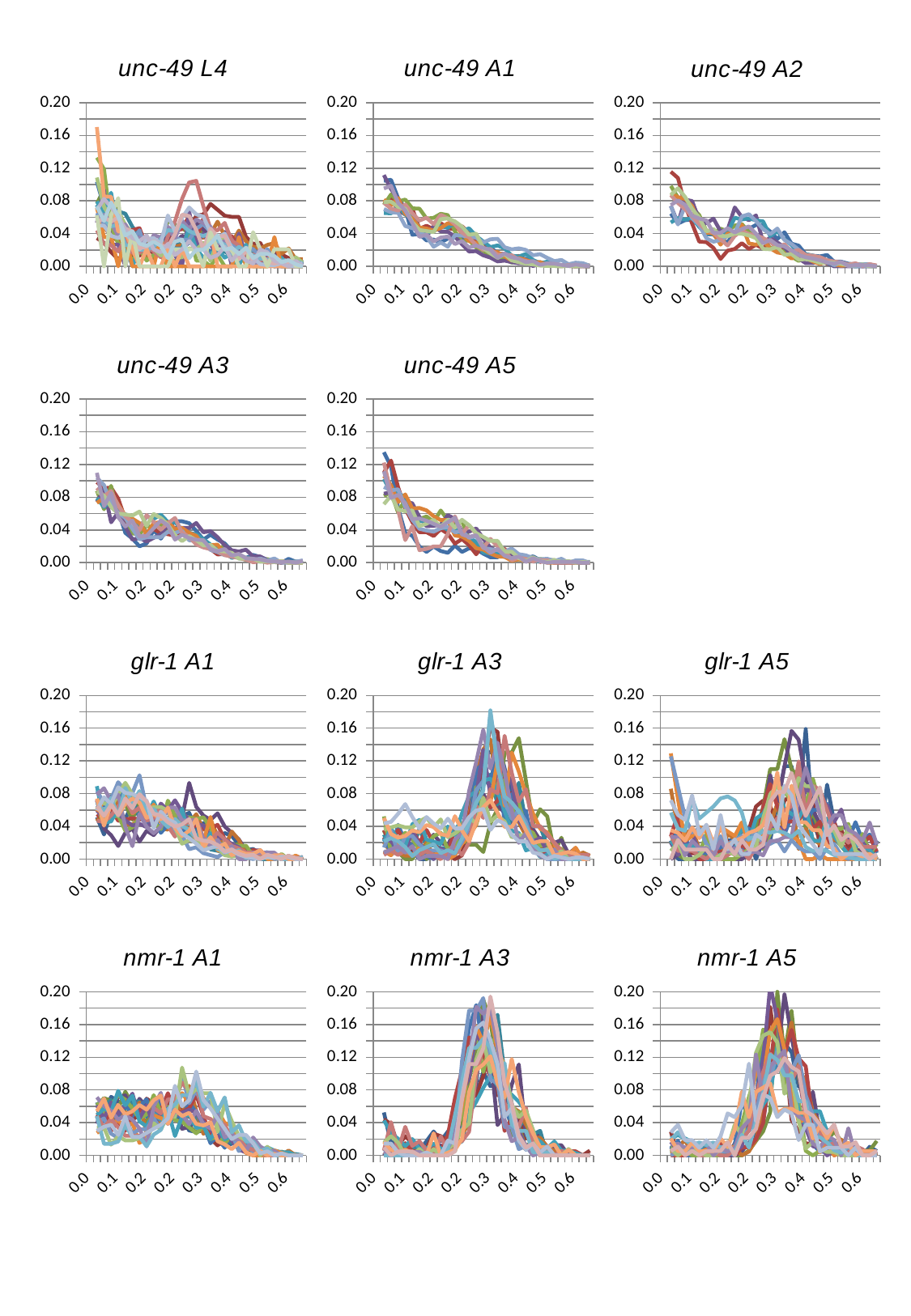

### Chart: unc-49 L4
| Category | unc-49_L4_0601 | unc-49_L4_0602 | unc-49_L4_06021 | unc-49_L4_06022 | unc-49_L4_06023 | unc-49_L4_06024 | unc-49_L4_06025 | unc-49_L4_06026 | unc-49_L4_0603 | unc-49_L4_0604 | unc-49_L4_0605 | unc-49_L4_0606 | unc-49_L4_0607 | unc-49_L4_0608 | unc-49_L4_0609 | unc-49_L4_0611 | unc-49_L4_0612 | unc-49_L4_0613 | unc-49_L4_0614 | unc-49_L4_0615 | unc-49_L4_0616 | unc-49_L4_0617 | unc-49_L4_0618 | unc-49_L4_0619 |
|---|---|---|---|---|---|---|---|---|---|---|---|---|---|---|---|---|---|---|---|---|---|---|---|---|
| 0 | None | None | None | None | None | None | None | None | None | None | None | None | None | None | None | None | None | None | None | None | None | None | None | None |
| 2.0000000000000007E-2 | None | 0.03453237410071942 | 0.07650273224043723 | 0.06065318818040438 | 0.07506702412868634 | 0.0611353711790393 | 0.10334788937408998 | 0.05850487540628388 | 0.13286713286713311 | 0.05333333333333334 | 0.0793650793650794 | 0.07142857142857141 | 0.0651340996168582 | 0.043956043956044015 | 0.10840707964601802 | 0.055800293685756217 | 0.07416267942583735 | 0.17021276595744705 | 0.04972375690607732 | 0.052901023890785014 | 0.0625 | 0.0687285223367698 | 0.07304785894206549 | None |
| 4.0000000000000015E-2 | None | 0.028776978417266213 | 0.0942622950819672 | 0.057542768273716995 | 0.05093833780160863 | 0.048034934497816636 | 0.07278020378457063 | 0.05417118093174432 | 0.11888111888111905 | 0.0933333333333333 | 0.0634920634920635 | 0.0357142857142857 | 0.05172413793103452 | 0.02747252747252752 | 0.0730088495575221 | 0.0631424375917768 | 0.057416267942583754 | 0.08510638297872347 | 0.04972375690607732 | 0.05972696245733792 | 0.0 | 0.08247422680412368 | 0.05541561712846352 | None |
| 6.0000000000000019E-2 | None | 0.0172661870503597 | 0.064207650273224 | 0.054432348367029613 | 0.064343163538874 | 0.037117903930131 | 0.06550218340611351 | 0.0422535211267606 | 0.048951048951049 | 0.026666666666666707 | 0.08994708994708994 | 0.0357142857142857 | 0.04980842911877392 | 0.02564102564102561 | 0.05752212389380532 | 0.054331864904552114 | 0.049043062200956895 | 0.08510638297872347 | 0.03729281767955802 | 0.054607508532423216 | 0.0625 | 0.0721649484536082 | 0.0780856423173804 | None |
| 8.0000000000000029E-2 | None | 0.00863309352517986 | 0.07103825136612021 | 0.0404354587869362 | 0.0670241286863271 | 0.03056768558951961 | 0.06841339155749643 | 0.05417118093174432 | 0.02797202797202801 | 0.026666666666666707 | 0.04232804232804231 | 0.0 | 0.05747126436781611 | 0.018315018315018305 | 0.06637168141592922 | 0.0367107195301028 | 0.05023923444976081 | 0.042553191489361715 | 0.0345303867403315 | 0.05972696245733792 | 0.08333333333333333 | 0.0618556701030928 | 0.060453400503778315 | None |
| 0.1 | None | 0.0158273381294964 | 0.0437158469945355 | 0.04976671850699843 | 0.064343163538874 | 0.0283842794759825 | 0.040756914119359514 | 0.041170097508125704 | 0.020979020979021008 | 0.0 | 0.02645502645502651 | 0.0357142857142857 | 0.0421455938697318 | 0.032967032967033016 | 0.0508849557522124 | 0.051395007342143924 | 0.026315789473684202 | 0.042553191489361715 | 0.041436464088397816 | 0.04607508532423209 | 0.0 | 0.0446735395189003 | 0.03778337531486151 | None |
| 0.12000000000000002 | None | 0.014388489208633106 | 0.0437158469945355 | 0.0217729393468118 | 0.04825737265415548 | 0.0152838427947598 | 0.03056768558951961 | 0.0444203683640303 | 0.006993006993006992 | 0.040000000000000015 | 0.0 | 0.0 | 0.03065134099616861 | 0.0164835164835165 | 0.0398230088495575 | 0.0337738619676946 | 0.023923444976076597 | 0.0212765957446809 | 0.030386740331491694 | 0.04266211604095562 | 0.0416666666666667 | 0.0343642611683849 | 0.04282115869017628 | None |
| 0.14000000000000001 | None | 0.012949640287769801 | 0.030054644808743206 | 0.023328149300155494 | 0.037533512064343216 | 0.021834061135371202 | 0.027656477438136807 | 0.04658721560130009 | 0.020979020979021008 | 0.026666666666666707 | 0.021164021164021198 | 0.0 | 0.0440613026819923 | 0.0164835164835165 | 0.0243362831858407 | 0.03524229074889871 | 0.02033492822966511 | 0.0 | 0.020718232044198894 | 0.0221843003412969 | 0.0 | 0.0309278350515464 | 0.027707808564231717 | None |
| 0.16 | None | 0.023021582733812992 | 0.03142076502732241 | 0.020217729393468088 | 0.008042895442359253 | 0.010917030567685601 | 0.0232896652110626 | 0.023835319609967518 | 0.006993006993006992 | 0.026666666666666707 | 0.0158730158730159 | 0.0 | 0.017241379310344803 | 0.023809523809523808 | 0.0353982300884956 | 0.020558002936857597 | 0.02033492822966511 | 0.0212765957446809 | 0.0331491712707182 | 0.023890784982935197 | 0.0 | 0.03780068728522342 | 0.02518891687657432 | None |
| 0.18000000000000005 | None | 0.0158273381294964 | 0.0286885245901639 | 0.023328149300155494 | 0.010723860589812305 | 0.010917030567685601 | 0.021834061135371202 | 0.0314192849404117 | 0.013986013986014 | 0.026666666666666707 | 0.02645502645502651 | 0.0 | 0.028735632183908007 | 0.018315018315018305 | 0.02212389380530971 | 0.038179148311306914 | 0.023923444976076597 | 0.0 | 0.0345303867403315 | 0.030716723549488085 | 0.0 | 0.02061855670103089 | 0.0302267002518892 | None |
| 0.2 | None | 0.0158273381294964 | 0.027322404371584685 | 0.0248833592534992 | 0.032171581769437 | 0.017467248908296894 | 0.02474526928675401 | 0.028169014084506998 | 0.013986013986014 | 0.013333333333333301 | 0.02645502645502651 | 0.0357142857142857 | 0.026819923371647507 | 0.010989010989011 | 0.0132743362831858 | 0.03524229074889871 | 0.0334928229665072 | 0.0 | 0.027624309392265206 | 0.025597269624573413 | 0.0 | 0.0171821305841924 | 0.02518891687657432 | None |
| 0.22 | None | 0.0273381294964029 | 0.05601092896174863 | 0.038880248833592514 | 0.024128686327077688 | 0.019650655021834103 | 0.029112081513828197 | 0.022751895991332597 | 0.0 | 0.013333333333333301 | 0.02645502645502651 | 0.0 | 0.017241379310344803 | 0.036630036630036604 | 0.015486725663716809 | 0.048458149779735685 | 0.04425837320574162 | 0.0212765957446809 | 0.062154696132596735 | 0.023890784982935197 | 0.0 | 0.027491408934707907 | 0.010075566750629698 | None |
| 0.24000000000000005 | None | 0.043165467625899304 | 0.0505464480874317 | 0.0279937791601866 | 0.032171581769437 | 0.0240174672489083 | 0.021834061135371202 | 0.04333694474539542 | 0.006993006993006992 | 0.013333333333333301 | 0.021164021164021198 | 0.0 | 0.03448275862068972 | 0.054945054945054896 | 0.019911504424778806 | 0.0337738619676946 | 0.043062200956937836 | 0.0 | 0.042817679558011135 | 0.03924914675767923 | 0.02083333333333331 | 0.013745704467354004 | 0.0302267002518892 | None |
| 0.26 | None | 0.04748201438848922 | 0.046448087431694 | 0.0373250388802488 | 0.024128686327077688 | 0.026200873362445407 | 0.021834061135371202 | 0.04550379198266523 | 0.006993006993006992 | 0.0 | 0.021164021164021198 | 0.0 | 0.032567049808429116 | 0.0824175824175824 | 0.011061946902654895 | 0.055800293685756217 | 0.05023923444976081 | 0.0212765957446809 | 0.059392265193370236 | 0.0631399317406143 | 0.02083333333333331 | 0.02061855670103089 | 0.0327455919395466 | None |
| 0.28000000000000008 | None | 0.044604316546762585 | 0.040983606557377004 | 0.0342146189735614 | 0.02949061662198391 | 0.03930131004366812 | 0.029112081513828197 | 0.061755146262188476 | 0.048951048951049 | 0.0666666666666667 | 0.047619047619047616 | 0.0 | 0.032567049808429116 | 0.10256410256410303 | 0.02212389380530971 | 0.0455212922173275 | 0.043062200956937836 | 0.0 | 0.0718232044198895 | 0.061433447098976114 | 0.02083333333333331 | 0.0309278350515464 | 0.010075566750629698 | None |
| 0.3000000000000001 | None | 0.061870503597122296 | 0.046448087431694 | 0.0528771384136858 | 0.037533512064343216 | 0.037117903930131 | 0.027656477438136807 | 0.0335861321776815 | 0.006993006993006992 | 0.026666666666666707 | 0.021164021164021198 | 0.0 | 0.04597701149425289 | 0.10439560439560402 | 0.019911504424778806 | 0.057268722466960395 | 0.039473684210526314 | 0.0 | 0.0635359116022099 | 0.04436860068259392 | 0.02083333333333331 | 0.02061855670103089 | 0.020151133501259407 | None |
| 0.32000000000000012 | None | 0.06330935251798557 | 0.03961748633879779 | 0.0451010886469673 | 0.021447721179624717 | 0.0349344978165939 | 0.02474526928675401 | 0.05850487540628388 | 0.006993006993006992 | 0.040000000000000015 | 0.0105820105820106 | 0.0 | 0.05172413793103452 | 0.0732600732600733 | 0.028761061946902686 | 0.05286343612334805 | 0.03708133971291872 | 0.0 | 0.060773480662983416 | 0.02047781569965871 | 0.0 | 0.02061855670103089 | 0.020151133501259407 | None |
| 0.34 | None | 0.0762589928057554 | 0.03142076502732241 | 0.0342146189735614 | 0.0187667560321716 | 0.03930131004366812 | 0.0160116448326055 | 0.04333694474539542 | 0.0 | 0.026666666666666707 | 0.0158730158730159 | 0.0 | 0.0421455938697318 | 0.0457875457875458 | 0.006637168141592923 | 0.048458149779735685 | 0.0406698564593301 | 0.042553191489361715 | 0.041436464088397816 | 0.04607508532423209 | 0.0416666666666667 | 0.010309278350515504 | 0.027707808564231717 | None |
| 0.3600000000000001 | None | 0.06906474820143883 | 0.021857923497267808 | 0.03265940902021772 | 0.01340482573726541 | 0.054585152838428 | 0.020378457059679802 | 0.03033586132177681 | 0.013986013986014 | 0.013333333333333301 | 0.021164021164021198 | 0.0 | 0.032567049808429116 | 0.043956043956044015 | 0.0309734513274336 | 0.027900146842878108 | 0.031100478468899517 | 0.0 | 0.0345303867403315 | 0.03583617747440272 | 0.02083333333333331 | 0.013745704467354004 | 0.03778337531486151 | None |
| 0.38000000000000012 | None | 0.061870503597122296 | 0.027322404371584685 | 0.041990668740279895 | 0.0187667560321716 | 0.037117903930131 | 0.021834061135371202 | 0.024918743228602398 | 0.0 | 0.040000000000000015 | 0.0105820105820106 | 0.0 | 0.022988505747126402 | 0.0531135531135531 | 0.03318584070796461 | 0.030837004405286306 | 0.026315789473684202 | 0.0 | 0.04005524861878447 | 0.037542662116041 | 0.02083333333333331 | 0.02405498281786941 | 0.020151133501259407 | None |
| 0.4 | None | 0.060431654676258995 | 0.015027322404371598 | 0.020217729393468088 | 0.010723860589812305 | 0.0283842794759825 | 0.0232896652110626 | 0.03683640303358611 | 0.0 | 0.0 | 0.0158730158730159 | 0.0 | 0.032567049808429116 | 0.031135531135531094 | 0.008849557522123897 | 0.013215859030837008 | 0.0155502392344498 | 0.0 | 0.022099447513812213 | 0.0187713310580205 | 0.02083333333333331 | 0.00687285223367698 | 0.022670025188916913 | None |
| 0.4200000000000001 | None | 0.060431654676258995 | 0.0122950819672131 | 0.020217729393468088 | 0.002680965147453081 | 0.043668122270742384 | 0.014556040756914098 | 0.023835319609967518 | 0.006993006993006992 | 0.040000000000000015 | 0.0 | 0.0357142857142857 | 0.0134099616858238 | 0.02564102564102561 | 0.0132743362831858 | 0.014684287812041098 | 0.023923444976076597 | 0.0 | 0.0151933701657459 | 0.008532423208191134 | 0.0 | 0.0171821305841924 | 0.015113350125944598 | None |
| 0.44 | None | 0.0359712230215827 | 0.00409836065573771 | 0.020217729393468088 | 0.016085790884718506 | 0.03056768558951961 | 0.013100436681222703 | 0.017334777898158203 | 0.006993006993006992 | 0.013333333333333301 | 0.02645502645502651 | 0.0 | 0.022988505747126402 | 0.014652014652014699 | 0.0176991150442478 | 0.007342143906020562 | 0.01794258373205741 | 0.0 | 0.020718232044198894 | 0.0187713310580205 | 0.0 | 0.00687285223367698 | 0.022670025188916913 | None |
| 0.46 | None | 0.0273381294964029 | 0.010928961748633904 | 0.023328149300155494 | 0.016085790884718506 | 0.0240174672489083 | 0.008733624454148473 | 0.014084507042253504 | 0.0 | 0.013333333333333301 | 0.00529100529100529 | 0.0 | 0.0210727969348659 | 0.014652014652014699 | 0.008849557522123897 | 0.007342143906020562 | 0.013157894736842101 | 0.0 | 0.011049723756906101 | 0.006825938566552898 | 0.0416666666666667 | 0.00343642611683849 | 0.010075566750629698 | None |
| 0.48000000000000009 | None | 0.028776978417266213 | 0.00819672131147541 | 0.00933125972006221 | 0.008042895442359253 | 0.021834061135371202 | 0.007278020378457062 | 0.007583965330444202 | 0.0 | 0.0 | 0.0 | 0.0 | 0.0114942528735632 | 0.00732600732600733 | 0.011061946902654895 | 0.00146842878120411 | 0.009569377990430622 | 0.0 | 0.00276243093922652 | 0.017064846416382305 | 0.02083333333333331 | 0.02061855670103089 | 0.015113350125944598 | None |
| 0.5 | None | 0.011510791366906505 | 0.009562841530054657 | 0.006220839813374812 | 0.0053619302949061715 | 0.026200873362445407 | 0.008733624454148473 | 0.011917659804983704 | 0.0 | 0.013333333333333301 | 0.00529100529100529 | 0.0 | 0.0210727969348659 | 0.009157509157509165 | 0.006637168141592923 | 0.00293685756240822 | 0.008373205741626795 | 0.0 | 0.0013812154696132613 | 0.005119453924914683 | 0.0 | 0.0171821305841924 | 0.015113350125944598 | None |
| 0.52 | None | 0.0172661870503597 | 0.005464480874316938 | 0.0139968895800933 | 0.0 | 0.0283842794759825 | 0.00436681222707424 | 0.003250270855904661 | 0.006993006993006992 | 0.0 | 0.0 | 0.0357142857142857 | 0.005747126436781612 | 0.003663003663003661 | 0.011061946902654895 | 0.00146842878120411 | 0.008373205741626795 | 0.0 | 0.0013812154696132613 | 0.003412969283276451 | 0.02083333333333331 | 0.00687285223367698 | 0.012594458438287204 | None |
| 0.54 | None | 0.014388489208633106 | 0.00136612021857923 | 0.006220839813374812 | 0.002680965147453081 | 0.0152838427947598 | 0.008733624454148473 | 0.003250270855904661 | 0.0 | 0.0 | 0.0105820105820106 | 0.0 | 0.003831417624521071 | 0.0018315018315018308 | 0.0022123893805309713 | 0.00293685756240822 | 0.007177033492822974 | 0.0 | 0.0041436464088397814 | 0.005119453924914683 | 0.02083333333333331 | 0.00343642611683849 | 0.005037783375314863 | None |
| 0.56000000000000005 | None | 0.010071942446043198 | 0.00136612021857923 | 0.006220839813374812 | 0.0053619302949061715 | 0.021834061135371202 | 0.00436681222707424 | 0.00216684723726977 | 0.0 | 0.0 | 0.0 | 0.0 | 0.003831417624521071 | 0.00549450549450549 | 0.0022123893805309713 | 0.0 | 0.007177033492822974 | 0.0 | 0.0 | 0.005119453924914683 | 0.02083333333333331 | 0.0 | 0.007556675062972292 | None |
| 0.58000000000000007 | None | 0.001438848920863311 | 0.00136612021857923 | 0.0 | 0.002680965147453081 | 0.008733624454148473 | 0.00145560407569141 | 0.00216684723726977 | 0.0 | 0.0 | 0.0 | 0.0 | 0.0 | 0.003663003663003661 | 0.011061946902654895 | 0.00293685756240822 | 0.005980861244019144 | 0.0 | 0.0 | 0.005119453924914683 | 0.0 | 0.00687285223367698 | 0.002518891687657432 | None |
| 0.6000000000000002 | None | 0.005755395683453244 | 0.002732240437158469 | 0.0 | 0.008042895442359253 | 0.008733624454148473 | 0.00582241630276565 | 0.0010834236186348894 | 0.0 | 0.0 | 0.0 | 0.0 | 0.0 | 0.003663003663003661 | 0.00442477876106195 | 0.0 | 0.003588516746411483 | 0.0 | 0.0041436464088397814 | 0.0 | 0.0 | 0.0 | 0.0 | None |
### Chart: unc-49 A1
| Category | unc-49_REX_A1_01 | unc-49_REX_A1_02 | unc-49_REX_A1_03 | unc-49_REX_A1_04 | unc-49_REX_A1_05 | unc-49_REX_A1_06 | unc-49_REX_A1_07 | unc-49_REX_A1_08 | unc-49_REX_A1_09 | unc-49_REX_A1_10 |
|---|---|---|---|---|---|---|---|---|---|---|
| 0 | None | None | None | None | None | None | None | None | None | None |
| 2.0000000000000007E-2 | 0.10453762205628908 | 0.0759894459102902 | 0.07497928748964373 | 0.11147236414305597 | 0.06520605112154411 | 0.08146247594611933 | 0.0699481865284974 | 0.07758186397984891 | 0.07849364791288575 | 0.09510618651892888 |
| 4.0000000000000015E-2 | 0.105686387133831 | 0.08232189973614783 | 0.08782104391052203 | 0.0942870413376684 | 0.06468440271257171 | 0.07440667094291215 | 0.0660621761658031 | 0.0705289672544081 | 0.07894736842105257 | 0.09833795013850423 |
| 6.0000000000000019E-2 | 0.08328546812176917 | 0.0686015831134565 | 0.0791217895608948 | 0.08221086855550402 | 0.07303077725612943 | 0.08338678640153939 | 0.0654145077720207 | 0.06801007556675057 | 0.07259528130671512 | 0.07756232686980613 |
| 8.0000000000000029E-2 | 0.070649052268811 | 0.06332453825857523 | 0.08160729080364543 | 0.06967022758941012 | 0.0631194574856547 | 0.06414368184733804 | 0.04922279792746113 | 0.0755667506297229 | 0.0703266787658802 | 0.0683287165281625 |
| 0.1 | 0.038483630097645 | 0.07071240105540905 | 0.07083678541839272 | 0.056665118439386895 | 0.05946791862284823 | 0.0583707504810776 | 0.046632124352331626 | 0.06498740554156172 | 0.0539927404718693 | 0.0530932594644506 |
| 0.12000000000000002 | 0.03963239517518671 | 0.04591029023746702 | 0.0704225352112676 | 0.046446818392940084 | 0.04747000521648408 | 0.04746632456703012 | 0.042098445595854884 | 0.05591939546599499 | 0.04446460980036303 | 0.03601108033241 |
| 0.14000000000000001 | 0.0315910396323952 | 0.04960422163588392 | 0.0584092792046396 | 0.0362285183464933 | 0.0417318727177882 | 0.046183450930083414 | 0.03626943005181352 | 0.0594458438287154 | 0.04310344827586208 | 0.03878116343490302 |
| 0.16 | 0.0327398047099368 | 0.04538258575197891 | 0.0592377796188898 | 0.04923362749651653 | 0.0427751695357329 | 0.043617703656189895 | 0.0239637305699482 | 0.05138539042821163 | 0.0417422867513612 | 0.03047091412742382 |
| 0.18000000000000005 | 0.029293509477311915 | 0.0527704485488127 | 0.0642087821043911 | 0.0427310729215049 | 0.049034950443401226 | 0.0468248877485568 | 0.02914507772020731 | 0.06297229219143582 | 0.05762250453720511 | 0.035087719298245605 |
| 0.2 | 0.0338885697874785 | 0.046965699208443325 | 0.0625517812758906 | 0.04226660473757552 | 0.0469483568075117 | 0.05259781911481722 | 0.0239637305699482 | 0.058438287153652416 | 0.058529945553538985 | 0.036472760849492206 |
| 0.22 | 0.0361860999425617 | 0.0448548812664908 | 0.04266777133388572 | 0.0362285183464933 | 0.0537297861241523 | 0.0513149454778704 | 0.0382124352331606 | 0.05138539042821163 | 0.05490018148820332 | 0.02723915050784861 |
| 0.24000000000000005 | 0.03446295232624931 | 0.04907651715039582 | 0.0302402651201326 | 0.026939154667905207 | 0.043296817944705336 | 0.0397690827453496 | 0.0375647668393782 | 0.04130982367758188 | 0.048548094373865695 | 0.030932594644505998 |
| 0.26 | 0.0327398047099368 | 0.0401055408970976 | 0.03189726594863301 | 0.0181142591732466 | 0.0464267083985394 | 0.030788967286722313 | 0.03626943005181352 | 0.035264483627204 | 0.0385662431941924 | 0.023084025854109 |
| 0.28000000000000008 | 0.024124066628374508 | 0.029023746701847 | 0.0227837613918807 | 0.018578727357176 | 0.037037037037037014 | 0.02886465683130208 | 0.0349740932642487 | 0.0256926952141058 | 0.0399274047186933 | 0.0253924284395199 |
| 0.3000000000000001 | 0.021252153934520398 | 0.027968337730870714 | 0.0190555095277548 | 0.013469577333952607 | 0.030255607720396517 | 0.0211674150096216 | 0.02914507772020731 | 0.020151133501259407 | 0.022232304900181507 | 0.022622345337026808 |
| 0.32000000000000012 | 0.0235496840896037 | 0.021635883905013215 | 0.012427506213753105 | 0.010682768230376201 | 0.02347417840375591 | 0.0179602309172547 | 0.0330310880829016 | 0.0211586901763224 | 0.0158802177858439 | 0.020313942751615913 |
| 0.34 | 0.015508328546812207 | 0.014775725593667506 | 0.011184755592377806 | 0.0060380863910822116 | 0.0255607720396453 | 0.0173187940987813 | 0.0336787564766839 | 0.014105793450881598 | 0.010435571687840305 | 0.0106186518928901 |
| 0.3600000000000001 | 0.0143595634692705 | 0.015303430079155701 | 0.009113504556752288 | 0.006967022758941011 | 0.0219092331768388 | 0.012187299550994193 | 0.0233160621761658 | 0.014105793450881598 | 0.014972776769510003 | 0.015697137580794097 |
| 0.38000000000000012 | 0.004595060310166572 | 0.012137203166226894 | 0.0045567522783761414 | 0.00510915002322341 | 0.0130412102243088 | 0.007055805003207182 | 0.0207253886010363 | 0.013602015113350106 | 0.007259528130671513 | 0.011080332409972301 |
| 0.4 | 0.009764503159103967 | 0.012664907651715 | 0.0070422535211267625 | 0.00371574547143521 | 0.0119979134063641 | 0.0102629890955741 | 0.022020725388601 | 0.008060453400503783 | 0.00499092558983666 | 0.009233610341643576 |
| 0.4200000000000001 | 0.00919012062033314 | 0.006860158311345651 | 0.003314001657000831 | 0.00371574547143521 | 0.015127803860198201 | 0.007697241821680563 | 0.020077720207253905 | 0.006549118387909322 | 0.0031760435571687802 | 0.005078485687903971 |
| 0.44 | 0.00516944284893739 | 0.006860158311345651 | 0.00207125103562552 | 0.000464468183929401 | 0.00782472613458529 | 0.00513149454778704 | 0.013601036269430109 | 0.004030226700251893 | 0.002268602540834851 | 0.00646352723915051 |
| 0.46 | 0.00516944284893739 | 0.0021108179419525117 | 0.002485501242750622 | 0.00278680910357641 | 0.0036515388628064727 | 0.00513149454778704 | 0.014896373056994795 | 0.002015113350125941 | 0.0009074410163339384 | 0.0018467220683287212 |
| 0.48000000000000009 | 0.00172314761631246 | 0.003693931398416892 | 0.0016570008285004105 | 0.0018578727357176006 | 0.005738132498695882 | 0.0019243104554201407 | 0.010362694300518104 | 0.002015113350125941 | 0.00045372050816696935 | 0.003693444136657431 |
| 0.5 | 0.00172314761631246 | 0.0010554089709762509 | 0.00207125103562552 | 0.000464468183929401 | 0.00312989045383412 | 0.0019243104554201407 | 0.0064766839378238355 | 0.0005037783375314863 | 0.00045372050816696935 | 0.003231763619575251 |
| 0.52 | 0.0011487650775416404 | 0.0010554089709762509 | 0.0008285004142502075 | 0.000464468183929401 | 0.0015649452269170605 | 0.0012828736369467609 | 0.007772020725388605 | 0.0 | 0.0 | 0.0018467220683287212 |
| 0.54 | 0.0011487650775416404 | 0.00158311345646438 | 0.00041425020712510403 | 0.000464468183929401 | 0.002086593635889411 | 0.0006414368184733807 | 0.00259067357512953 | 0.00151133501259446 | 0.00045372050816696935 | 0.000461680517082179 |
| 0.56000000000000005 | 0.0 | 0.0021108179419525117 | 0.0008285004142502075 | 0.0 | 0.00104329681794471 | 0.0 | 0.004533678756476682 | 0.0 | 0.0 | 0.0023084025854109 |
| 0.58000000000000007 | 0.0005743825387708213 | 0.0005277044854881272 | 0.00041425020712510403 | 0.0 | 0.00104329681794471 | 0.0032071840923669024 | 0.0038860103626943017 | 0.0 | 0.00045372050816696935 | 0.000923361034164358 |
| 0.6000000000000002 | 0.0 | 0.0010554089709762509 | 0.0 | 0.0 | 0.0005216484089723534 | 0.0006414368184733807 | 0.0006476683937823836 | 0.0 | 0.0 | 0.0 |
### Chart: unc-49 A2
| Category | unc-49_REX_A2_01 | unc-49_REX_A2_02 | unc-49_REX_A2_03 | unc-49_REX_A2_04 | unc-49_REX_A2_05 | unc-49_REX_A2_06 | unc-49_REX_A2_07 | unc-49_REX_A2_08 | unc-49_REX_A2_09 | unc-49_REX_A2_10 |
|---|---|---|---|---|---|---|---|---|---|---|
| 0 | None | None | None | None | None | None | None | None | None | None |
| 2.0000000000000007E-2 | 0.06454248366013073 | 0.11568123393316203 | 0.09833729216152023 | 0.0739299610894942 | 0.0536036036036036 | 0.08903605592347318 | 0.07358093903293624 | 0.08712121212121214 | 0.08725157537566655 | 0.07480106100795762 |
| 4.0000000000000015E-2 | 0.05228758169934642 | 0.10796915167095103 | 0.08408551068883614 | 0.052529182879377384 | 0.059009009009009 | 0.0846210448859455 | 0.051156271899089015 | 0.0773809523809524 | 0.0954920019389239 | 0.08063660477453578 |
| 6.0000000000000019E-2 | 0.0563725490196078 | 0.0745501285347044 | 0.08266033254156774 | 0.08171206225680934 | 0.0572072072072072 | 0.08241353936718177 | 0.074281709880869 | 0.06980519480519483 | 0.08482792050412026 | 0.07533156498673743 |
| 8.0000000000000029E-2 | 0.05718954248366007 | 0.05205655526992289 | 0.07173396674584323 | 0.07976653696498054 | 0.059009009009009 | 0.07358351729212663 | 0.058864751226349016 | 0.061147186147186096 | 0.0702859912748425 | 0.06206896551724142 |
| 0.1 | 0.05065359477124182 | 0.030205655526992305 | 0.06270783847981 | 0.056420233463034986 | 0.0481981981981982 | 0.052244297277409896 | 0.050455501051156315 | 0.051948051948052 | 0.05962190984003883 | 0.0583554376657825 |
| 0.12000000000000002 | 0.0392156862745098 | 0.029562982005141392 | 0.0432304038004751 | 0.052529182879377384 | 0.039189189189189205 | 0.04120676968359088 | 0.03433777154870361 | 0.0438311688311688 | 0.0426563257392147 | 0.056233421750663114 |
| 0.14000000000000001 | 0.0392156862745098 | 0.02249357326478151 | 0.0394299287410926 | 0.0583657587548638 | 0.04234234234234231 | 0.0404709345106696 | 0.030133146461107214 | 0.0411255411255411 | 0.04556471158507031 | 0.04084880636604769 |
| 0.16 | 0.02859477124183008 | 0.008997429305912604 | 0.0460807600950119 | 0.04280155642023351 | 0.0369369369369369 | 0.026490066225165608 | 0.03223545900490541 | 0.033008658008657994 | 0.0373242850218129 | 0.04084880636604769 |
| 0.18000000000000005 | 0.0302287581699346 | 0.019280205655527006 | 0.042755344418052295 | 0.04474708171206231 | 0.041441441441441414 | 0.0404709345106696 | 0.0371408549404345 | 0.02543290043290041 | 0.0368395540475036 | 0.046684350132626 |
| 0.2 | 0.0392156862745098 | 0.021208226221079717 | 0.04988123515439432 | 0.0719844357976654 | 0.0594594594594595 | 0.03752759381898451 | 0.04064470918009808 | 0.0373376623376623 | 0.04023267086766842 | 0.0413793103448276 |
| 0.22 | 0.04738562091503274 | 0.0282776349614396 | 0.051781472684085485 | 0.06031128404669264 | 0.0572072072072072 | 0.05077262693156734 | 0.0609670637701472 | 0.0432900432900433 | 0.0397479398933592 | 0.045623342175066285 |
| 0.24000000000000005 | 0.045751633986928136 | 0.021208226221079717 | 0.04180522565320671 | 0.056420233463034986 | 0.05855855855855858 | 0.0279617365710081 | 0.06377014716187808 | 0.03950216450216451 | 0.037809015996122214 | 0.04880636604774542 |
| 0.26 | 0.05065359477124182 | 0.0263496143958869 | 0.0375296912114014 | 0.062256809338521416 | 0.05630630630630632 | 0.026490066225165608 | 0.05536089698668542 | 0.037878787878787915 | 0.0344158991759573 | 0.0397877984084881 |
| 0.28000000000000008 | 0.049836601307189525 | 0.0218508997429306 | 0.030878859857482198 | 0.0291828793774319 | 0.05495495495495501 | 0.030905077262693217 | 0.0455501051156272 | 0.0346320346320346 | 0.021328162869607398 | 0.0281167108753316 |
| 0.3000000000000001 | 0.04248366013071902 | 0.030848329048843208 | 0.022802850356294507 | 0.0350194552529183 | 0.0369369369369369 | 0.02207505518763801 | 0.03924316748423271 | 0.029761904761904802 | 0.02084343189529809 | 0.032360742705570315 |
| 0.32000000000000012 | 0.03349673202614381 | 0.0237789203084833 | 0.019952494061757708 | 0.0291828793774319 | 0.04279279279279282 | 0.0169242089771891 | 0.04625087596355986 | 0.02218614718614721 | 0.023751817741153706 | 0.025994694960212197 |
| 0.34 | 0.0416666666666667 | 0.02249357326478151 | 0.018052256532066498 | 0.0233463035019455 | 0.032882882882882915 | 0.015452538631346604 | 0.03223545900490541 | 0.02705627705627712 | 0.014057198254968501 | 0.02440318302387271 |
| 0.3600000000000001 | 0.02777777777777782 | 0.019280205655527006 | 0.0118764845605701 | 0.0136186770428016 | 0.0261261261261261 | 0.009565857247976454 | 0.027330063069376315 | 0.017857142857142898 | 0.015026660203587006 | 0.019628647214854106 |
| 0.38000000000000012 | 0.02532679738562091 | 0.016709511568123406 | 0.00665083135391924 | 0.00972762645914397 | 0.015315315315315305 | 0.013980868285504008 | 0.020322354590049094 | 0.02002164502164501 | 0.008240426563257394 | 0.013793103448275905 |
| 0.4 | 0.014705882352941199 | 0.010282776349614407 | 0.0118764845605701 | 0.00389105058365759 | 0.0148648648648649 | 0.010301692420897698 | 0.012613875262789108 | 0.0140692640692641 | 0.008725157537566659 | 0.011140583554376703 |
| 0.4200000000000001 | 0.0130718954248366 | 0.0077120822622108 | 0.008076009501187658 | 0.00389105058365759 | 0.013063063063063101 | 0.005886681383370135 | 0.00840925017519271 | 0.012987012987013 | 0.007270964614638884 | 0.009549071618037126 |
| 0.44 | 0.012254901960784298 | 0.0077120822622108 | 0.005225653206650832 | 0.00778210116731518 | 0.010360360360360404 | 0.002943340691685061 | 0.00420462508759636 | 0.011363636363636404 | 0.00436257876878333 | 0.007427055702917772 |
| 0.46 | 0.013888888888888907 | 0.003213367609254501 | 0.004750593824228032 | 0.00389105058365759 | 0.0045045045045045 | 0.005886681383370135 | 0.00700770847932726 | 0.007575757575757582 | 0.004847309743092583 | 0.00477453580901857 |
| 0.48000000000000009 | 0.0057189542483660075 | 0.0019280205655527014 | 0.001900237529691212 | 0.0 | 0.0018018018018018005 | 0.002207505518763801 | 0.001401541695865449 | 0.00595238095238095 | 0.00436257876878333 | 0.003183023872679052 |
| 0.5 | 0.0057189542483660075 | 0.002570694087403601 | 0.001900237529691212 | 0.0 | 0.0009009009009009019 | 0.0007358351729212664 | 0.004905395935529082 | 0.004870129870129872 | 0.0029083858458555517 | 0.00530503978779841 |
| 0.52 | 0.0032679738562091526 | 0.0006426735218509 | 0.00142517814726841 | 0.0 | 0.0018018018018018005 | 0.00147167034584253 | 0.0007007708479327262 | 0.002164502164502161 | 0.000969461948618517 | 0.000530503978779841 |
| 0.54 | 0.00245098039215686 | 0.002570694087403601 | 0.00142517814726841 | 0.0 | 0.0 | 0.0007358351729212664 | 0.0 | 0.003787878787878792 | 0.000969461948618517 | 0.0015915119363395206 |
| 0.56000000000000005 | 0.00245098039215686 | 0.0 | 0.0009501187648456065 | 0.0 | 0.0009009009009009019 | 0.0007358351729212664 | 0.0 | 0.0005411255411255412 | 0.002423654871546291 | 0.0015915119363395206 |
| 0.58000000000000007 | 0.00245098039215686 | 0.001285347043701801 | 0.00047505938242280323 | 0.0 | 0.0009009009009009019 | 0.0007358351729212664 | 0.0 | 0.002705627705627712 | 0.0 | 0.00106100795755968 |
| 0.6000000000000002 | 0.0 | 0.0006426735218509 | 0.0 | 0.0 | 0.00045045045045045 | 0.0007358351729212664 | 0.0 | 0.0010822510822510805 | 0.0 | 0.0 |
### Chart: unc-49 A3
| Category | unc-49_REX_A3_01 | unc-49_REX_A3_02 | unc-49_REX_A3_03 | unc-49_REX_A3_04 | unc-49_REX_A3_05 | unc-49_REX_A3_06 | unc-49_REX_A3_07 | unc-49_REX_A3_08 | unc-49_REX_A3_09 | unc-49_REX_A3_10 |
|---|---|---|---|---|---|---|---|---|---|---|
| 0 | None | None | None | None | None | None | None | None | None | None |
| 2.0000000000000007E-2 | 0.0788235294117647 | 0.0983935742971888 | 0.08782608695652169 | 0.07424242424242423 | 0.07893020221787342 | 0.0730789897904353 | 0.103761348897536 | 0.0912356321839081 | 0.08708708708708708 | 0.10977242302543504 |
| 4.0000000000000015E-2 | 0.0752941176470588 | 0.09103078982597049 | 0.06521739130434782 | 0.0901515151515151 | 0.06784083496412263 | 0.07898979043524991 | 0.09468223086900128 | 0.0811781609195402 | 0.08168168168168166 | 0.06894243641231591 |
| 6.0000000000000019E-2 | 0.0717647058823529 | 0.09170013386880858 | 0.09391304347826096 | 0.049242424242424226 | 0.07632093933463803 | 0.08543793659322947 | 0.0713359273670558 | 0.0876436781609195 | 0.0678678678678679 | 0.0850066934404284 |
| 8.0000000000000029E-2 | 0.06352941176470589 | 0.07831325301204824 | 0.0565217391304348 | 0.06136363636363643 | 0.05936073059360733 | 0.0709296077377754 | 0.06095979247730219 | 0.069683908045977 | 0.0600600600600601 | 0.05555555555555558 |
| 0.1 | 0.0364705882352941 | 0.052878179384203486 | 0.0565217391304348 | 0.0439393939393939 | 0.05936073059360733 | 0.05749596990865133 | 0.0544747081712062 | 0.05675287356321841 | 0.0588588588588589 | 0.04417670682730923 |
| 0.12000000000000002 | 0.029411764705882398 | 0.0381526104417671 | 0.03565217391304351 | 0.028030303030303 | 0.050228310502283095 | 0.053734551316496515 | 0.0415045395590143 | 0.04956896551724142 | 0.057657657657657714 | 0.051539491298527404 |
| 0.14000000000000001 | 0.020000000000000007 | 0.03145917001338691 | 0.0347826086956522 | 0.0303030303030303 | 0.04174820613176779 | 0.04836109618484692 | 0.028534370946822308 | 0.0395114942528736 | 0.0624624624624625 | 0.0321285140562249 |
| 0.16 | 0.02352941176470589 | 0.034136546184739 | 0.04521739130434782 | 0.02575757575757581 | 0.0567514677103718 | 0.0349274583557227 | 0.0311284046692607 | 0.0581896551724138 | 0.045045045045045 | 0.03279785809906291 |
| 0.18000000000000005 | 0.040000000000000015 | 0.041499330655957185 | 0.0426086956521739 | 0.0303030303030303 | 0.05348988910632749 | 0.04836109618484692 | 0.0311284046692607 | 0.04956896551724142 | 0.0594594594594595 | 0.04216867469879522 |
| 0.2 | 0.029411764705882398 | 0.03547523427041501 | 0.04695652173913042 | 0.034848484848484886 | 0.05870841487279843 | 0.04943578721117682 | 0.0311284046692607 | 0.0402298850574713 | 0.0546546546546547 | 0.051539491298527404 |
| 0.22 | 0.0411764705882353 | 0.03480589022757701 | 0.040000000000000015 | 0.0454545454545455 | 0.0495759947814742 | 0.04943578721117682 | 0.0376134889753567 | 0.048132183908046036 | 0.046246246246246216 | 0.04551539491298532 |
| 0.24000000000000005 | 0.050588235294117614 | 0.03279785809906291 | 0.040000000000000015 | 0.0424242424242424 | 0.0495759947814742 | 0.0413756045137023 | 0.0363164721141375 | 0.05459770114942535 | 0.0336336336336336 | 0.02811244979919679 |
| 0.26 | 0.050588235294117614 | 0.034136546184739 | 0.040000000000000015 | 0.0424242424242424 | 0.040443574690149996 | 0.04245029554003222 | 0.0389105058365759 | 0.036637931034482804 | 0.0264264264264264 | 0.034136546184739 |
| 0.28000000000000008 | 0.04823529411764711 | 0.0334672021419009 | 0.031304347826087014 | 0.0424242424242424 | 0.036529680365296795 | 0.0365394948952176 | 0.027237354085603117 | 0.031609195402298916 | 0.0324324324324324 | 0.03012048192771079 |
| 0.3000000000000001 | 0.038823529411764715 | 0.0321285140562249 | 0.0252173913043478 | 0.048484848484848485 | 0.0345727332028702 | 0.0327780763030629 | 0.027237354085603117 | 0.02227011494252871 | 0.027627627627627615 | 0.02811244979919679 |
| 0.32000000000000012 | 0.02823529411764711 | 0.022757697456492608 | 0.0252173913043478 | 0.03712121212121212 | 0.0287018917155903 | 0.02149382052659861 | 0.024643320363164713 | 0.0186781609195402 | 0.021021021021021002 | 0.027443105756358815 |
| 0.34 | 0.03411764705882352 | 0.0167336010709505 | 0.0173913043478261 | 0.038636363636363615 | 0.022178734507501602 | 0.02149382052659861 | 0.0155642023346304 | 0.016522988505747103 | 0.021621621621621602 | 0.0167336010709505 |
| 0.3600000000000001 | 0.02823529411764711 | 0.010040160642570305 | 0.0139130434782609 | 0.031060606060606108 | 0.0163078930202218 | 0.022031166039763617 | 0.012970168612192003 | 0.014367816091953999 | 0.014414414414414399 | 0.014056224899598398 |
| 0.38000000000000012 | 0.02352941176470589 | 0.010709504685408305 | 0.0217391304347826 | 0.0212121212121212 | 0.013046314416177401 | 0.0102095647501343 | 0.009079118028534372 | 0.009339080459770123 | 0.013813813813813804 | 0.0167336010709505 |
| 0.4 | 0.014117647058823493 | 0.006693440428380192 | 0.011304347826087004 | 0.015151515151515204 | 0.005870841487279844 | 0.00967221923696938 | 0.011673151750972804 | 0.007183908045977011 | 0.012012012012012 | 0.008701472556894244 |
| 0.4200000000000001 | 0.009411764705882354 | 0.008701472556894244 | 0.006086956521739132 | 0.013636363636363601 | 0.007827788649706457 | 0.0102095647501343 | 0.0051880674448767814 | 0.005747126436781612 | 0.0048048048048048 | 0.010709504685408305 |
| 0.44 | 0.005882352941176471 | 0.0053547523427041515 | 0.00347826086956522 | 0.0159090909090909 | 0.00521852576647097 | 0.0042987641053197235 | 0.0051880674448767814 | 0.003591954022988511 | 0.005405405405405412 | 0.0053547523427041515 |
| 0.46 | 0.009411764705882354 | 0.0006693440428380193 | 0.00434782608695652 | 0.007575757575757582 | 0.0019569471624266118 | 0.003761418592154761 | 0.002594033722438391 | 0.0007183908045977012 | 0.0048048048048048 | 0.004685408299866131 |
| 0.48000000000000009 | 0.007058823529411772 | 0.002008032128514061 | 0.002608695652173911 | 0.007575757575757582 | 0.0013046314416177405 | 0.002149382052659861 | 0.0051880674448767814 | 0.002155172413793099 | 0.0012012012012012 | 0.0040160642570281095 |
| 0.5 | 0.0035294117647058816 | 0.0006693440428380193 | 0.00434782608695652 | 0.00303030303030303 | 0.0019569471624266118 | 0.00107469102632993 | 0.002594033722438391 | 0.0 | 0.0018018018018018005 | 0.002008032128514061 |
| 0.52 | 0.0011764705882352905 | 0.0006693440428380193 | 0.0017391304347826105 | 0.0015151515151515208 | 0.000652315720808871 | 0.00107469102632993 | 0.0051880674448767814 | 0.0007183908045977012 | 0.000600600600600601 | 0.001338688085676041 |
| 0.54 | 0.0 | 0.0 | 0.0017391304347826105 | 0.0007575757575757582 | 0.000652315720808871 | 0.00107469102632993 | 0.0 | 0.002155172413793099 | 0.0018018018018018005 | 0.0006693440428380193 |
| 0.56000000000000005 | 0.004705882352941182 | 0.001338688085676041 | 0.0 | 0.0007575757575757582 | 0.0013046314416177405 | 0.0005373455131649652 | 0.0 | 0.0 | 0.0 | 0.001338688085676041 |
| 0.58000000000000007 | 0.0011764705882352905 | 0.0 | 0.0 | 0.0 | 0.0013046314416177405 | 0.00107469102632993 | 0.0012970168612192003 | 0.0 | 0.0 | 0.0 |
| 0.6000000000000002 | 0.00235294117647059 | 0.0006693440428380193 | 0.0008695652173913045 | 0.0 | 0.0 | 0.0 | 0.002594033722438391 | 0.0 | 0.0 | 0.002008032128514061 |
### Chart: unc-49 A5
| Category | unc-49_A5_40_30_03 | unc-49_A5_40_30_04 | unc-49_REX_A5_01 | unc-49_REX_A5_02 | unc-49_REX_A5_03 | unc-49_REX_A5_04 | unc-49_REX_A5_05 | unc-49_REX_A5_06 | unc-49_REX_A5_09 | unc-49_REX_A5_10 |
|---|---|---|---|---|---|---|---|---|---|---|
| 0 | None | None | None | None | None | None | None | None | None | None |
| 2.0000000000000007E-2 | 0.13501646542261306 | 0.10986964618249502 | 0.08147174770039416 | 0.08419630796938324 | 0.10224719101123603 | 0.10585452395032502 | 0.09241379310344827 | 0.12232415902140703 | 0.07142857142857141 | 0.11173184357541906 |
| 4.0000000000000015E-2 | 0.117453347969265 | 0.12476722532588504 | 0.0860709592641262 | 0.0864475461503827 | 0.08595505617977532 | 0.09461856889414551 | 0.08827586206896554 | 0.0856269113149847 | 0.08190476190476188 | 0.0791433891992551 |
| 6.0000000000000019E-2 | 0.06695938529088907 | 0.09217877094972068 | 0.0611038107752957 | 0.0792435839711842 | 0.0842696629213483 | 0.07392075694855121 | 0.08965517241379317 | 0.0642201834862385 | 0.06476190476190481 | 0.0828677839851024 |
| 8.0000000000000029E-2 | 0.0362239297475302 | 0.0661080074487896 | 0.0775295663600526 | 0.075191355245385 | 0.08033707865168543 | 0.08338261383796562 | 0.06827586206896546 | 0.027522935779816515 | 0.0628571428571429 | 0.07262569832402241 |
| 0.1 | 0.03293084522502742 | 0.04934823091247674 | 0.059132720105124825 | 0.0724898694281855 | 0.053370786516853896 | 0.06623299822590181 | 0.052413793103448354 | 0.044342507645259904 | 0.06380952380952383 | 0.053072625698324 |
| 0.12000000000000002 | 0.019758507135016506 | 0.03724394785847303 | 0.05321944809461238 | 0.05583070688878882 | 0.050561797752809015 | 0.0668243642814902 | 0.0413793103448276 | 0.0152905198776758 | 0.0447619047619048 | 0.05400372439478582 |
| 0.14000000000000001 | 0.013172338090011 | 0.03724394785847303 | 0.0565045992115637 | 0.0445745159837911 | 0.051123595505618 | 0.06386753400354823 | 0.0393103448275862 | 0.016819571865443406 | 0.047619047619047616 | 0.0512104283054004 |
| 0.16 | 0.019758507135016506 | 0.0325884543761639 | 0.04993429697766101 | 0.045024763619990985 | 0.047191011235955115 | 0.05677114133648729 | 0.03793103448275861 | 0.019877675840978607 | 0.052380952380952396 | 0.04748603351955312 |
| 0.18000000000000005 | 0.014270032930845198 | 0.040968342644320296 | 0.06373193166885682 | 0.046375506528590696 | 0.04550561797752812 | 0.052040212891779986 | 0.042068965517241416 | 0.019877675840978607 | 0.04380952380952382 | 0.04376163873370582 |
| 0.2 | 0.0120746432491767 | 0.03538175046554931 | 0.05190538764783181 | 0.058081945069788386 | 0.03988764044943821 | 0.052040212891779986 | 0.04344827586206898 | 0.0351681957186544 | 0.052380952380952396 | 0.04934823091247674 |
| 0.22 | 0.020856201975850707 | 0.02327746741154561 | 0.0486202365308804 | 0.05357946870778928 | 0.042134831460674184 | 0.033116499112950885 | 0.03724137931034482 | 0.0565749235474006 | 0.04285714285714291 | 0.05493482309124772 |
| 0.24000000000000005 | 0.013172338090011 | 0.02886405959031659 | 0.05059132720105131 | 0.03827104907699232 | 0.0314606741573034 | 0.031933767001774124 | 0.038620689655172395 | 0.0351681957186544 | 0.052380952380952396 | 0.0344506517690875 |
| 0.26 | 0.017563117453348 | 0.021415270018622017 | 0.02890932982917211 | 0.037820801440792404 | 0.03089887640449441 | 0.026611472501478415 | 0.030344827586206918 | 0.045871559633027484 | 0.045714285714285714 | 0.03165735567970211 |
| 0.28000000000000008 | 0.016465422612513707 | 0.010242085661080109 | 0.021681997371879123 | 0.041873030166591614 | 0.025280898876404518 | 0.017740981667652308 | 0.0324137931034483 | 0.0351681957186544 | 0.0361904761904762 | 0.0335195530726257 |
| 0.3000000000000001 | 0.010976948408342499 | 0.02234636871508381 | 0.022339027595269418 | 0.029266096352994094 | 0.0123595505617978 | 0.015966883500887007 | 0.017931034482758602 | 0.018348623853211 | 0.03142857142857142 | 0.019553072625698303 |
| 0.32000000000000012 | 0.006586169045005492 | 0.015828677839851 | 0.01708278580814721 | 0.018460153084196306 | 0.0112359550561798 | 0.0112359550561798 | 0.016551724137931007 | 0.02905198776758411 | 0.026666666666666707 | 0.012104283054003698 |
| 0.34 | 0.006586169045005492 | 0.008379888268156424 | 0.007884362680683314 | 0.013507429085997308 | 0.012921348314606701 | 0.0076877587226493235 | 0.0151724137931034 | 0.019877675840978607 | 0.026666666666666707 | 0.016759776536312807 |
| 0.3600000000000001 | 0.013172338090011 | 0.016759776536312807 | 0.011826544021025004 | 0.00810445745159838 | 0.00842696629213483 | 0.007096392667060912 | 0.013793103448275905 | 0.0152905198776758 | 0.013333333333333301 | 0.008379888268156424 |
| 0.38000000000000012 | 0.014270032930845198 | 0.006517690875232772 | 0.0032851511169513818 | 0.009905447996398028 | 0.004494382022471911 | 0.00236546422235364 | 0.017241379310344803 | 0.003058103975535171 | 0.013333333333333301 | 0.005586592178770952 |
| 0.4 | 0.006586169045005492 | 0.007448789571694603 | 0.003942181340341662 | 0.006753714542998651 | 0.005056179775280902 | 0.004139562389118874 | 0.0103448275862069 | 0.006116207951070342 | 0.00571428571428571 | 0.007448789571694603 |
| 0.4200000000000001 | 0.005488474204171242 | 0.0018621973929236505 | 0.008541392904073587 | 0.006753714542998651 | 0.003932584269662922 | 0.001774098166765231 | 0.00896551724137932 | 0.00458715596330275 | 0.0019047619047619013 | 0.0009310986964618257 |
| 0.44 | 0.0076838638858397436 | 0.005586592178770952 | 0.0013140604467805508 | 0.0013507429085997309 | 0.00168539325842697 | 0.002956830277942052 | 0.005517241379310342 | 0.0015290519877675804 | 0.006666666666666671 | 0.005586592178770952 |
| 0.46 | 0.003293084522502742 | 0.0009310986964618257 | 0.0045992115637319315 | 0.0013507429085997309 | 0.00168539325842697 | 0.00236546422235364 | 0.00413793103448276 | 0.003058103975535171 | 0.0019047619047619013 | 0.0018621973929236505 |
| 0.48000000000000009 | 0.0010976948408342499 | 0.002793296089385481 | 0.0032851511169513818 | 0.0031517334533993717 | 0.0022471910112359626 | 0.0 | 0.004827586206896546 | 0.003058103975535171 | 0.0009523809523809524 | 0.0 |
| 0.5 | 0.0021953896816685 | 0.0 | 0.0032851511169513818 | 0.00045024763619991016 | 0.00112359550561798 | 0.0 | 0.0020689655172413815 | 0.0 | 0.00285714285714286 | 0.0009310986964618257 |
| 0.52 | 0.0010976948408342499 | 0.0 | 0.0006570302233902764 | 0.0 | 0.0005617977528089888 | 0.0005913660555884095 | 0.004827586206896546 | 0.0 | 0.0009523809523809524 | 0.0009310986964618257 |
| 0.54 | 0.0 | 0.0 | 0.0006570302233902764 | 0.0 | 0.0005617977528089888 | 0.0 | 0.0 | 0.0 | 0.0019047619047619013 | 0.0009310986964618257 |
| 0.56000000000000005 | 0.0 | 0.0009310986964618257 | 0.0013140604467805508 | 0.0 | 0.0005617977528089888 | 0.0 | 0.002758620689655171 | 0.0 | 0.0 | 0.0009310986964618257 |
| 0.58000000000000007 | 0.0 | 0.0 | 0.0006570302233902764 | 0.0 | 0.0 | 0.001182732111176821 | 0.002758620689655171 | 0.0 | 0.0009523809523809524 | 0.0 |
| 0.6000000000000002 | 0.0 | 0.0 | 0.0 | 0.00045024763619991016 | 0.00112359550561798 | 0.0 | 0.0 | 0.0 | 0.0 | 0.0 |
### Chart: glr-1 A1
| Category | glr-1(n2461)_A1_01 | glr-1(n2461)_A1_02 | glr-1(n2461)_A1_03 | glr-1(n2461)_A1_04 | glr-1(n2461)_A1_05 | glr-1(n2461)_A1_06 | glr-1(n2461)_A1_07 | glr-1(n2461)_A1_08 | glr-1(n2461)_A1_09 | glr-1(n2461)_A1_10 | glr-1(n2461)_A1_11 | glr-1(n2461)_A1_12 | glr-1(n2461)_A1_13 | glr-1(n2461)_A1_14 | glr-1(n2461)_A1_15 | glr-1(n2461)_A1_16 | glr-1(n2461)_A1_17 | glr-1(n2461)_A1_18 | glr-1(n2461)_A1_19 | glr-1(n2461)_A1_20 |
|---|---|---|---|---|---|---|---|---|---|---|---|---|---|---|---|---|---|---|---|---|
| 0 | None | None | None | None | None | None | None | None | None | None | None | None | None | None | None | None | None | None | None | None |
| 2.0000000000000007E-2 | 0.051146384479717796 | 0.062182741116751324 | 0.0600343053173242 | 0.0718085106382979 | 0.06218487394957981 | 0.0464135021097046 | 0.06545961002785523 | 0.05161290322580652 | 0.063265306122449 | 0.05954825462012322 | 0.0890804597701149 | 0.0579399141630901 | 0.0831325301204819 | 0.04684975767366716 | 0.07142857142857141 | 0.078740157480315 | 0.06976744186046513 | 0.0738993710691824 | 0.05937921727395409 | 0.06481481481481483 |
| 4.0000000000000015E-2 | 0.0299823633156966 | 0.06725888324873101 | 0.0600343053173242 | 0.03723404255319152 | 0.0705882352941176 | 0.05907172995780592 | 0.06545961002785523 | 0.058064516129032316 | 0.04285714285714291 | 0.0492813141683778 | 0.0445402298850575 | 0.07081545064377683 | 0.0686746987951807 | 0.04523424878836828 | 0.0683229813664596 | 0.08661417322834652 | 0.0563035495716034 | 0.0408805031446541 | 0.07557354925775979 | 0.0529100529100529 |
| 6.0000000000000019E-2 | 0.05820105820105816 | 0.06979695431472085 | 0.0617495711835334 | 0.029255319148936202 | 0.07731092436974793 | 0.0464135021097046 | 0.06545961002785523 | 0.0612903225806452 | 0.051020408163265286 | 0.0718685831622177 | 0.04597701149425289 | 0.0686695278969957 | 0.07349397590361453 | 0.053311793214862714 | 0.0636645962732919 | 0.07086614173228352 | 0.07466340269277852 | 0.06761006289308183 | 0.0674763832658569 | 0.0661375661375661 |
| 8.0000000000000029E-2 | 0.0564373897707231 | 0.08375634517766513 | 0.05831903945111491 | 0.0159574468085106 | 0.0705882352941176 | 0.0717299578059072 | 0.0933147632311978 | 0.0467741935483871 | 0.0530612244897959 | 0.0554414784394251 | 0.061781609195402314 | 0.05579399141630904 | 0.09397590361445783 | 0.06462035541195477 | 0.0791925465838509 | 0.078740157480315 | 0.052631578947368404 | 0.053459119496855285 | 0.087719298245614 | 0.05423280423280422 |
| 0.1 | 0.049382716049382734 | 0.0685279187817259 | 0.06689536878216117 | 0.031914893617021316 | 0.08403361344537819 | 0.04219409282700422 | 0.0710306406685237 | 0.0483870967741935 | 0.03469387755102042 | 0.04722792607802872 | 0.06896551724137931 | 0.08154506437768251 | 0.0831325301204819 | 0.07269789983844913 | 0.09316770186335399 | 0.0393700787401575 | 0.0869033047735618 | 0.06761006289308183 | 0.0809716599190283 | 0.0740740740740741 |
| 0.12000000000000002 | 0.06701940035273374 | 0.0736040609137056 | 0.048027444253859304 | 0.03723404255319152 | 0.0722689075630252 | 0.04219409282700422 | 0.0752089136490251 | 0.05 | 0.0387755102040816 | 0.04106776180698152 | 0.06896551724137931 | 0.05579399141630904 | 0.08072289156626515 | 0.0565428109854604 | 0.0791925465838509 | 0.015748031496063006 | 0.0685434516523868 | 0.0738993710691824 | 0.07962213225371123 | 0.0621693121693122 |
| 0.14000000000000001 | 0.051146384479717796 | 0.07106598984771573 | 0.07204116638078897 | 0.0212765957446809 | 0.0521008403361345 | 0.048523206751054884 | 0.0710306406685237 | 0.0483870967741935 | 0.051020408163265286 | 0.04312114989733059 | 0.0718390804597701 | 0.0622317596566524 | 0.102409638554217 | 0.06300484652665587 | 0.0683229813664596 | 0.05511811023622051 | 0.08323133414932685 | 0.06918238993710693 | 0.07557354925775979 | 0.0793650793650794 |
| 0.16 | 0.044091710758377416 | 0.048223350253807085 | 0.0617495711835334 | 0.034574468085106405 | 0.0588235294117647 | 0.0654008438818565 | 0.04178272980501392 | 0.05322580645161288 | 0.06734693877551023 | 0.039014373716632404 | 0.05172413793103452 | 0.053648068669527864 | 0.0614457831325301 | 0.04684975767366716 | 0.05900621118012424 | 0.0393700787401575 | 0.05385556915544682 | 0.050314465408805 | 0.0715249662618084 | 0.0701058201058201 |
| 0.18000000000000005 | 0.0529100529100529 | 0.05583756345177672 | 0.0600343053173242 | 0.029255319148936202 | 0.050420168067226885 | 0.04219409282700422 | 0.05431754874651812 | 0.05161290322580652 | 0.044897959183673515 | 0.04722792607802872 | 0.07040229885057474 | 0.0665236051502146 | 0.0614457831325301 | 0.0500807754442649 | 0.0636645962732919 | 0.031496062992126005 | 0.0611995104039168 | 0.0550314465408805 | 0.03778677462887992 | 0.05555555555555558 |
| 0.2 | 0.045855379188712485 | 0.05456852791878174 | 0.051457975986277896 | 0.03989361702127662 | 0.050420168067226885 | 0.037974683544303806 | 0.03203342618384401 | 0.05645161290322582 | 0.03673469387755101 | 0.0677618069815195 | 0.050287356321839095 | 0.0450643776824034 | 0.0554216867469879 | 0.06300484652665587 | 0.0636645962732919 | 0.0393700787401575 | 0.04773561811505511 | 0.04874213836477988 | 0.058029689608637004 | 0.0529100529100529 |
| 0.22 | 0.04232804232804231 | 0.040609137055837616 | 0.0411663807890223 | 0.05053191489361702 | 0.040336134453781536 | 0.035864978902953606 | 0.04178272980501392 | 0.05645161290322582 | 0.07142857142857141 | 0.05954825462012322 | 0.04310344827586208 | 0.0450643776824034 | 0.046987951807228916 | 0.04038772213247173 | 0.05900621118012424 | 0.06299212598425201 | 0.0391676866585067 | 0.06289308176100633 | 0.048582995951417025 | 0.044973544973545 |
| 0.24000000000000005 | 0.04232804232804231 | 0.05203045685279194 | 0.03773584905660382 | 0.0531914893617021 | 0.040336134453781536 | 0.05063291139240512 | 0.04178272980501392 | 0.05322580645161288 | 0.0530612244897959 | 0.0718685831622177 | 0.0359195402298851 | 0.04935622317596572 | 0.03253012048192772 | 0.0274636510500808 | 0.03881987577639751 | 0.06299212598425201 | 0.048959608323133404 | 0.03773584905660382 | 0.044534412955465626 | 0.039682539682539715 |
| 0.26 | 0.0529100529100529 | 0.0342639593908629 | 0.051457975986277896 | 0.05053191489361702 | 0.04705882352941181 | 0.05907172995780592 | 0.0376044568245125 | 0.0483870967741935 | 0.04693877551020408 | 0.0574948665297741 | 0.061781609195402314 | 0.040772532188841235 | 0.0289156626506024 | 0.04038772213247173 | 0.0186335403726708 | 0.047244094488189 | 0.04039167686658512 | 0.028301886792452793 | 0.03103913630229421 | 0.04232804232804231 |
| 0.28000000000000008 | 0.0564373897707231 | 0.026649746192893408 | 0.030874785591766714 | 0.0930851063829787 | 0.04537815126050418 | 0.0443037974683544 | 0.0278551532033426 | 0.035483870967741915 | 0.051020408163265286 | 0.04722792607802872 | 0.0258620689655172 | 0.036480686695279 | 0.012048192771084298 | 0.04684975767366716 | 0.023291925465838508 | 0.047244094488189 | 0.035495716034271714 | 0.034591194968553514 | 0.02564102564102561 | 0.0489417989417989 |
| 0.3000000000000001 | 0.037037037037037014 | 0.024111675126903608 | 0.0291595197255575 | 0.06382978723404252 | 0.0302521008403361 | 0.0548523206751055 | 0.03342618384401109 | 0.0419354838709677 | 0.04897959183673472 | 0.04312114989733059 | 0.03017241379310351 | 0.0236051502145923 | 0.0144578313253012 | 0.0484652665589661 | 0.0217391304347826 | 0.047244094488189 | 0.025703794369645 | 0.04874213836477988 | 0.018893387314439906 | 0.025132275132275117 |
| 0.32000000000000012 | 0.044091710758377416 | 0.0190355329949239 | 0.024013722126929718 | 0.0531914893617021 | 0.026890756302521 | 0.035864978902953606 | 0.019498607242339806 | 0.0370967741935484 | 0.051020408163265286 | 0.04106776180698152 | 0.0158045977011494 | 0.021459227467811218 | 0.0072289156626506 | 0.025848142164781908 | 0.015527950310559004 | 0.0236220472440945 | 0.022031823745410014 | 0.014150943396226398 | 0.010796221322537101 | 0.02248677248677252 |
| 0.34 | 0.021164021164021198 | 0.0126903553299492 | 0.0188679245283019 | 0.047872340425531915 | 0.011764705882352905 | 0.048523206751054884 | 0.02924791086350972 | 0.0370967741935484 | 0.0387755102040816 | 0.028747433264887094 | 0.031609195402298916 | 0.051502145922746816 | 0.004819277108433742 | 0.022617124394184198 | 0.0170807453416149 | 0.015748031496063006 | 0.024479804161566705 | 0.026729559748427695 | 0.0134952766531714 | 0.021164021164021198 |
| 0.3600000000000001 | 0.02645502645502651 | 0.0228426395939086 | 0.022298456260720398 | 0.05585106382978721 | 0.010084033613445401 | 0.027426160337552692 | 0.012534818941504199 | 0.0419354838709677 | 0.018367346938775498 | 0.026694045174538 | 0.017241379310344803 | 0.0107296137339056 | 0.00240963855421687 | 0.03231017770597741 | 0.013975155279503108 | 0.0236220472440945 | 0.012239902080783398 | 0.014150943396226398 | 0.0161943319838057 | 0.017195767195767205 |
| 0.38000000000000012 | 0.0317460317460317 | 0.0126903553299492 | 0.0171526586620926 | 0.03989361702127662 | 0.008403361344537806 | 0.014767932489451498 | 0.0167130919220056 | 0.0241935483870968 | 0.018367346938775498 | 0.0246406570841889 | 0.0186781609195402 | 0.008583690987124463 | 0.008433734939759048 | 0.024232633279483 | 0.0046583850931677 | 0.007874015748031498 | 0.011015911872704997 | 0.0188679245283019 | 0.00809716599190283 | 0.00925925925925927 |
| 0.4 | 0.0282186948853616 | 0.0101522842639594 | 0.0154373927958834 | 0.031914893617021316 | 0.016806722689075605 | 0.0337552742616034 | 0.008356545961002798 | 0.009677419354838717 | 0.016326530612244903 | 0.0184804928131417 | 0.007183908045977011 | 0.012875536480686699 | 0.0072289156626506 | 0.016155088852988702 | 0.003105590062111802 | 0.015748031496063006 | 0.00734394124847001 | 0.00471698113207547 | 0.010796221322537101 | 0.011904761904761904 |
| 0.4200000000000001 | 0.017636684303351 | 0.011421319796954304 | 0.0188679245283019 | 0.023936170212766002 | 0.003361344537815131 | 0.023206751054852294 | 0.00974930362116992 | 0.011290322580645199 | 0.016326530612244903 | 0.014373716632443499 | 0.0114942528735632 | 0.008583690987124463 | 0.0012048192771084295 | 0.012924071082391 | 0.0046583850931677 | 0.0 | 0.011015911872704997 | 0.00943396226415094 | 0.00539811066126856 | 0.006613756613756612 |
| 0.44 | 0.0105820105820106 | 0.0 | 0.003430531732418522 | 0.010638297872340394 | 0.006722689075630252 | 0.006329113924050632 | 0.00278551532033426 | 0.004838709677419362 | 0.016326530612244903 | 0.004106776180698152 | 0.005747126436781612 | 0.002145922746781122 | 0.00240963855421687 | 0.008077544426494354 | 0.006211180124223604 | 0.0 | 0.0012239902080783398 | 0.006289308176100634 | 0.002699055330634279 | 0.0039682539682539715 |
| 0.46 | 0.012345679012345704 | 0.008883248730964473 | 0.00514579759862779 | 0.010638297872340394 | 0.001680672268907561 | 0.006329113924050632 | 0.004178272980501392 | 0.011290322580645199 | 0.004081632653061222 | 0.00205338809034908 | 0.005747126436781612 | 0.004291845493562234 | 0.0 | 0.008077544426494354 | 0.0015527950310559005 | 0.007874015748031498 | 0.003671970624235011 | 0.006289308176100634 | 0.00134952766531714 | 0.00529100529100529 |
| 0.48000000000000009 | 0.00529100529100529 | 0.0038071065989847717 | 0.0017152658662092605 | 0.010638297872340394 | 0.0 | 0.0021097046413502112 | 0.006963788300835652 | 0.008064516129032265 | 0.00204081632653061 | 0.00205338809034908 | 0.004310344827586208 | 0.0107296137339056 | 0.00240963855421687 | 0.004846526655896612 | 0.0015527950310559005 | 0.007874015748031498 | 0.0 | 0.011006289308176103 | 0.0 | 0.0 |
| 0.5 | 0.007054673721340392 | 0.00126903553299492 | 0.0017152658662092605 | 0.0026595744680851107 | 0.0 | 0.0042194092827004225 | 0.005571030640668522 | 0.003225806451612902 | 0.00612244897959184 | 0.00205338809034908 | 0.008620689655172415 | 0.0 | 0.003614457831325301 | 0.004846526655896612 | 0.0015527950310559005 | 0.007874015748031498 | 0.003671970624235011 | 0.0015723270440251608 | 0.002699055330634279 | 0.00529100529100529 |
| 0.52 | 0.003527336860670192 | 0.0038071065989847717 | 0.00514579759862779 | 0.0026595744680851107 | 0.003361344537815131 | 0.0 | 0.004178272980501392 | 0.003225806451612902 | 0.0 | 0.004106776180698152 | 0.0014367816091954001 | 0.0 | 0.0 | 0.0032310177705977415 | 0.0015527950310559005 | 0.007874015748031498 | 0.002447980416156671 | 0.00471698113207547 | 0.00134952766531714 | 0.002645502645502652 |
| 0.54 | 0.0017636684303351004 | 0.00126903553299492 | 0.003430531732418522 | 0.0 | 0.001680672268907561 | 0.0021097046413502112 | 0.0013927576601671305 | 0.0 | 0.00612244897959184 | 0.00205338809034908 | 0.0014367816091954001 | 0.002145922746781122 | 0.0012048192771084295 | 0.0016155088852988701 | 0.0046583850931677 | 0.0 | 0.0 | 0.00471698113207547 | 0.002699055330634279 | 0.0 |
| 0.56000000000000005 | 0.0017636684303351004 | 0.0038071065989847717 | 0.0017152658662092605 | 0.0026595744680851107 | 0.001680672268907561 | 0.0 | 0.0 | 0.0016129032258064505 | 0.0 | 0.0 | 0.0 | 0.0 | 0.0 | 0.0 | 0.0 | 0.0 | 0.0 | 0.0 | 0.002699055330634279 | 0.0039682539682539715 |
| 0.58000000000000007 | 0.0 | 0.0 | 0.0 | 0.0 | 0.0 | 0.0 | 0.00278551532033426 | 0.0 | 0.0 | 0.0 | 0.0 | 0.004291845493562234 | 0.0012048192771084295 | 0.0016155088852988701 | 0.0 | 0.0 | 0.0 | 0.0 | 0.0 | 0.0013227513227513205 |
| 0.6000000000000002 | 0.0017636684303351004 | 0.0 | 0.0 | 0.0026595744680851107 | 0.0 | 0.0021097046413502112 | 0.0013927576601671305 | 0.0016129032258064505 | 0.0 | 0.0 | 0.0 | 0.0 | 0.0 | 0.0016155088852988701 | 0.0 | 0.0 | 0.002447980416156671 | 0.0 | 0.0 | 0.0 |
### Chart: glr-1 A3
| Category | glr-1(n2461)_A3_01 | glr-1(n2461)_A3_02 | glr-1(n2461)_A3_03 | glr-1(n2461)_A3_04 | glr-1(n2461)_A3_05 | glr-1(n2461)_A3_06 | glr-1(n2461)_A3_07 | glr-1(n2461)_A3_08 | glr-1(n2461)_A3_09 | glr-1(n2461)_A3_10 | glr-1(n2461)_A3_11 | glr-1(n2461)_A3_12 | glr-1(n2461)_A3_13 | glr-1(n2461)_A3_14 | glr-1(n2461)_A3_15 | glr-1(n2461)_A3_16 | glr-1(n2461)_A3_17 | glr-1(n2461)_A3_18 | glr-1(n2461)_A3_19 |
|---|---|---|---|---|---|---|---|---|---|---|---|---|---|---|---|---|---|---|---|
| 0 | None | None | None | None | None | None | None | None | None | None | None | None | None | None | None | None | None | None | None |
| 2.0000000000000007E-2 | 0.00966183574879227 | 0.026666666666666707 | 0.0347826086956522 | 0.036423841059602606 | 0.030000000000000002 | 0.020325203252032485 | 0.02510460251046031 | 0.03557312252964431 | 0.05220883534136549 | 0.0258620689655172 | 0.0212765957446809 | 0.00930232558139535 | 0.014084507042253504 | 0.005025125628140698 | 0.036319612590799015 | 0.00790513833992095 | 0.021818181818181792 | 0.0502183406113537 | 0.0434782608695652 |
| 4.0000000000000015E-2 | 0.0048309178743961385 | 0.008888888888888894 | 0.0173913043478261 | 0.036423841059602606 | 0.013333333333333301 | 0.00813008130081301 | 0.008368200836820085 | 0.019762845849802414 | 0.00803212851405622 | 0.02155172413793099 | 0.03723404255319152 | 0.0046511627906976735 | 0.03521126760563382 | 0.0100502512562814 | 0.0387409200968523 | 0.0118577075098814 | 0.025454545454545514 | 0.03056768558951961 | 0.0454545454545455 |
| 6.0000000000000019E-2 | 0.0144927536231884 | 0.026666666666666707 | 0.008695652173913045 | 0.016556291390728502 | 0.0166666666666667 | 0.00813008130081301 | 0.041841004184100396 | 0.03557312252964431 | 0.0200803212851406 | 0.0129310344827586 | 0.023936170212766002 | 0.0186046511627907 | 0.0070422535211267625 | 0.005025125628140698 | 0.041162227602905624 | 0.0158102766798419 | 0.021818181818181792 | 0.026200873362445407 | 0.05533596837944662 |
| 8.0000000000000029E-2 | 0.00966183574879227 | 0.004444444444444442 | 0.0 | 0.029801324503311313 | 0.026666666666666707 | 0.0040650406504065 | 0.020920502092050198 | 0.019762845849802414 | 0.0321285140562249 | 0.008620689655172415 | 0.0159574468085106 | 0.00930232558139535 | 0.028169014084506998 | 0.0100502512562814 | 0.0387409200968523 | 0.00790513833992095 | 0.0145454545454545 | 0.0283842794759825 | 0.06719367588932815 |
| 0.1 | 0.00966183574879227 | 0.013333333333333301 | 0.0 | 0.026490066225165608 | 0.013333333333333301 | 0.0040650406504065 | 0.0167364016736402 | 0.03557312252964431 | 0.0240963855421687 | 0.03448275862068972 | 0.042553191489361715 | 0.0046511627906976735 | 0.028169014084506998 | 0.005025125628140698 | 0.0387409200968523 | 0.0158102766798419 | 0.00363636363636364 | 0.0349344978165939 | 0.05138339920948622 |
| 0.12000000000000002 | 0.0 | 0.004444444444444442 | 0.0173913043478261 | 0.013245033112582804 | 0.0366666666666667 | 0.00813008130081301 | 0.020920502092050198 | 0.019762845849802414 | 0.012048192771084298 | 0.017241379310344803 | 0.047872340425531915 | 0.013953488372093 | 0.0176056338028169 | 0.005025125628140698 | 0.04600484261501207 | 0.0 | 0.010909090909090898 | 0.0327510917030568 | 0.03754940711462452 |
| 0.14000000000000001 | 0.0 | 0.004444444444444442 | 0.0 | 0.013245033112582804 | 0.0166666666666667 | 0.020325203252032485 | 0.00418410041841004 | 0.03557312252964431 | 0.016064257028112403 | 0.0129310344827586 | 0.023936170212766002 | 0.00930232558139535 | 0.0105633802816901 | 0.0100502512562814 | 0.05084745762711862 | 0.00395256916996047 | 0.0145454545454545 | 0.04148471615720522 | 0.05138339920948622 |
| 0.16 | 0.0048309178743961385 | 0.004444444444444442 | 0.008695652173913045 | 0.029801324503311313 | 0.0166666666666667 | 0.0040650406504065 | 0.0 | 0.019762845849802414 | 0.02811244979919679 | 0.0 | 0.0212765957446809 | 0.0046511627906976735 | 0.0105633802816901 | 0.005025125628140698 | 0.0387409200968523 | 0.00395256916996047 | 0.018181818181818205 | 0.03930131004366812 | 0.0434782608695652 |
| 0.18000000000000005 | 0.00966183574879227 | 0.013333333333333301 | 0.008695652173913045 | 0.019867549668874208 | 0.013333333333333301 | 0.0 | 0.008368200836820085 | 0.00790513833992095 | 0.0321285140562249 | 0.0129310344827586 | 0.018617021276595706 | 0.00930232558139535 | 0.0105633802816901 | 0.0 | 0.048426150121065395 | 0.00395256916996047 | 0.007272727272727272 | 0.03056768558951961 | 0.03754940711462452 |
| 0.2 | 0.0048309178743961385 | 0.004444444444444442 | 0.008695652173913045 | 0.016556291390728502 | 0.013333333333333301 | 0.0121951219512195 | 0.008368200836820085 | 0.00790513833992095 | 0.012048192771084298 | 0.008620689655172415 | 0.03989361702127662 | 0.0 | 0.0070422535211267625 | 0.0100502512562814 | 0.026634382566586012 | 0.0 | 0.010909090909090898 | 0.0283842794759825 | 0.049407114624505914 |
| 0.22 | 0.00966183574879227 | 0.0 | 0.008695652173913045 | 0.036423841059602606 | 0.013333333333333301 | 0.016260162601626 | 0.012552301255230104 | 0.03557312252964431 | 0.016064257028112403 | 0.008620689655172415 | 0.029255319148936202 | 0.0046511627906976735 | 0.0070422535211267625 | 0.0150753768844221 | 0.03147699757869253 | 0.0237154150197628 | 0.007272727272727272 | 0.0524017467248908 | 0.03754940711462452 |
| 0.24000000000000005 | 0.01932367149758451 | 0.004444444444444442 | 0.026086956521739115 | 0.036423841059602606 | 0.030000000000000002 | 0.024390243902439 | 0.02510460251046031 | 0.027667984189723313 | 0.04417670682730923 | 0.02155172413793099 | 0.05585106382978721 | 0.0186046511627907 | 0.02112676056338029 | 0.005025125628140698 | 0.0387409200968523 | 0.0316205533596838 | 0.025454545454545514 | 0.0349344978165939 | 0.03754940711462452 |
| 0.26 | 0.0676328502415459 | 0.06222222222222222 | 0.0173913043478261 | 0.036423841059602606 | 0.05 | 0.052845528455284584 | 0.03765690376569041 | 0.05138339920948622 | 0.0240963855421687 | 0.03017241379310351 | 0.0771276595744681 | 0.027906976744186 | 0.0387323943661972 | 0.0201005025125628 | 0.05084745762711862 | 0.08300395256917004 | 0.043636363636363584 | 0.021834061135371202 | 0.05138339920948622 |
| 0.28000000000000008 | 0.10144927536231903 | 0.07111111111111111 | 0.0173913043478261 | 0.05298013245033112 | 0.04333333333333333 | 0.10162601626016303 | 0.10878661087866104 | 0.0790513833992095 | 0.0883534136546185 | 0.07758620689655173 | 0.0824468085106383 | 0.0418604651162791 | 0.08450704225352115 | 0.06532663316582923 | 0.0435835351089588 | 0.118577075098814 | 0.06909090909090912 | 0.04585152838427948 | 0.05928853754940712 |
| 0.3000000000000001 | 0.130434782608696 | 0.12000000000000002 | 0.008695652173913045 | 0.0794701986754967 | 0.09000000000000002 | 0.13414634146341506 | 0.11715481171548102 | 0.07509881422924902 | 0.06024096385542172 | 0.13362068965517193 | 0.09574468085106383 | 0.06976744186046513 | 0.09507042253521132 | 0.05025125628140698 | 0.07506053268765131 | 0.158102766798419 | 0.08363636363636368 | 0.06331877729257641 | 0.06126482213438739 |
| 0.32000000000000012 | 0.130434782608696 | 0.16 | 0.0434782608695652 | 0.06291390728476823 | 0.103333333333333 | 0.14634146341463405 | 0.0920502092050209 | 0.06324110671936756 | 0.07228915662650597 | 0.07758620689655173 | 0.0984042553191489 | 0.06976744186046513 | 0.17605633802816906 | 0.085427135678392 | 0.04600484261501207 | 0.110671936758893 | 0.18181818181818207 | 0.07205240174672493 | 0.03557312252964431 |
| 0.34 | 0.144927536231884 | 0.155555555555556 | 0.060869565217391314 | 0.06622516556291387 | 0.0666666666666667 | 0.08130081300813008 | 0.138075313807531 | 0.0711462450592885 | 0.0923694779116466 | 0.10775862068965503 | 0.0824468085106383 | 0.12093023255814005 | 0.12676056338028197 | 0.0804020100502513 | 0.0532687651331719 | 0.14229249011857706 | 0.11272727272727304 | 0.0567685589519651 | 0.04743083003952574 |
| 0.3600000000000001 | 0.111111111111111 | 0.08000000000000003 | 0.130434782608696 | 0.056291390728476796 | 0.08333333333333333 | 0.09756097560975616 | 0.041841004184100396 | 0.0988142292490119 | 0.09638554216867473 | 0.10775862068965503 | 0.058510638297872314 | 0.0651162790697674 | 0.0704225352112676 | 0.15075376884422106 | 0.04600484261501207 | 0.10671936758893302 | 0.07636363636363645 | 0.03930131004366812 | 0.0434782608695652 |
| 0.38000000000000012 | 0.07729468599033824 | 0.08000000000000003 | 0.130434782608696 | 0.0397350993377483 | 0.046666666666666703 | 0.0609756097560976 | 0.07949790794979085 | 0.05138339920948622 | 0.05622489959839358 | 0.09482758620689656 | 0.034574468085106405 | 0.13023255813953497 | 0.049295774647887314 | 0.10552763819095498 | 0.026634382566586012 | 0.05928853754940712 | 0.06909090909090912 | 0.03930131004366812 | 0.029644268774703615 |
| 0.4 | 0.05314009661835748 | 0.05333333333333334 | 0.14782608695652205 | 0.05298013245033112 | 0.0933333333333333 | 0.04471544715447153 | 0.0627615062761506 | 0.05138339920948622 | 0.06827309236947789 | 0.04741379310344834 | 0.031914893617021316 | 0.10697674418604704 | 0.0598591549295775 | 0.07035175879396978 | 0.029055690072639202 | 0.019762845849802414 | 0.05818181818181819 | 0.0524017467248908 | 0.0217391304347826 |
| 0.4200000000000001 | 0.0338164251207729 | 0.04444444444444442 | 0.09565217391304354 | 0.036423841059602606 | 0.04333333333333333 | 0.0284552845528455 | 0.05857740585774063 | 0.0434782608695652 | 0.036144578313253 | 0.03448275862068972 | 0.010638297872340394 | 0.07906976744186052 | 0.04577464788732392 | 0.085427135678392 | 0.029055690072639202 | 0.0316205533596838 | 0.040000000000000015 | 0.0349344978165939 | 0.0217391304347826 |
| 0.44 | 0.01932367149758451 | 0.022222222222222202 | 0.0434782608695652 | 0.036423841059602606 | 0.040000000000000015 | 0.016260162601626 | 0.0167364016736402 | 0.0316205533596838 | 0.0321285140562249 | 0.02155172413793099 | 0.013297872340425504 | 0.0558139534883721 | 0.02112676056338029 | 0.0402010050251256 | 0.0121065375302663 | 0.0118577075098814 | 0.018181818181818205 | 0.019650655021834103 | 0.00790513833992095 |
| 0.46 | 0.0144927536231884 | 0.013333333333333301 | 0.060869565217391314 | 0.0231788079470199 | 0.040000000000000015 | 0.016260162601626 | 0.0167364016736402 | 0.0158102766798419 | 0.016064257028112403 | 0.0258620689655172 | 0.0026595744680851107 | 0.037209302325581416 | 0.0070422535211267625 | 0.0402010050251256 | 0.009685230024213084 | 0.00395256916996047 | 0.010909090909090898 | 0.021834061135371202 | 0.005928853754940712 |
| 0.48000000000000009 | 0.0048309178743961385 | 0.004444444444444442 | 0.05217391304347832 | 0.029801324503311313 | 0.0166666666666667 | 0.0121951219512195 | 0.008368200836820085 | 0.0 | 0.016064257028112403 | 0.008620689655172415 | 0.00531914893617021 | 0.0325581395348837 | 0.0 | 0.0301507537688442 | 0.0121065375302663 | 0.0 | 0.010909090909090898 | 0.021834061135371202 | 0.0 |
| 0.5 | 0.0 | 0.004444444444444442 | 0.008695652173913045 | 0.019867549668874208 | 0.006666666666666671 | 0.016260162601626 | 0.0 | 0.0118577075098814 | 0.0040160642570281095 | 0.004310344827586208 | 0.0 | 0.0186046511627907 | 0.0 | 0.0201005025125628 | 0.00484261501210654 | 0.0118577075098814 | 0.0 | 0.00436681222707424 | 0.00395256916996047 |
| 0.52 | 0.0 | 0.0 | 0.026086956521739115 | 0.0231788079470199 | 0.0 | 0.0121951219512195 | 0.0 | 0.00395256916996047 | 0.012048192771084298 | 0.008620689655172415 | 0.0 | 0.0 | 0.0 | 0.0100502512562814 | 0.009685230024213084 | 0.0 | 0.00363636363636364 | 0.006550218340611352 | 0.001976284584980242 |
| 0.54 | 0.0 | 0.004444444444444442 | 0.0 | 0.006622516556291386 | 0.006666666666666671 | 0.0040650406504065 | 0.0 | 0.00790513833992095 | 0.0 | 0.0 | 0.0026595744680851107 | 0.0046511627906976735 | 0.003521126760563382 | 0.005025125628140698 | 0.0 | 0.0 | 0.0 | 0.008733624454148473 | 0.0 |
| 0.56000000000000005 | 0.0 | 0.0 | 0.008695652173913045 | 0.013245033112582804 | 0.0 | 0.0 | 0.0 | 0.0 | 0.0 | 0.008620689655172415 | 0.0 | 0.013953488372093 | 0.0 | 0.005025125628140698 | 0.0 | 0.0 | 0.0 | 0.006550218340611352 | 0.001976284584980242 |
| 0.58000000000000007 | 0.0 | 0.0 | 0.0 | 0.0 | 0.0 | 0.00813008130081301 | 0.0 | 0.0 | 0.0040160642570281095 | 0.0 | 0.0 | 0.0 | 0.003521126760563382 | 0.005025125628140698 | 0.0 | 0.0 | 0.0 | 0.0 | 0.001976284584980242 |
| 0.6000000000000002 | 0.0 | 0.0 | 0.0 | 0.0 | 0.0 | 0.0040650406504065 | 0.0 | 0.0 | 0.0 | 0.0 | 0.0 | 0.0 | 0.0 | 0.005025125628140698 | 0.0 | 0.0 | 0.0 | 0.0 | 0.0 |
### Chart: glr-1 A5
| Category | glr-1(n2461)_A5_01 | glr-1(n2461)_A5_02 | glr-1(n2461)_A5_03 | glr-1(n2461)_A5_04 | glr-1(n2461)_A5_05 | glr-1(n2461)_A5_06 | glr-1(n2461)_A5_07 | glr-1(n2461)_A5_08 | glr-1(n2461)_A5_09 | glr-1(n2461)_A5_10 | glr-1(n2461)_A5_11 | glr-1(n2461)_A5_12 | glr-1(n2461)_A5_13 | glr-1(n2461)_A5_14 | glr-1(n2461)_A5_15 | glr-1(n2461)_A5_16 | glr-1(n2461)_A5_17 | glr-1(n2461)_A5_18 | glr-1(n2461)_A5_19 | glr-1(n2461)_A5_20 |
|---|---|---|---|---|---|---|---|---|---|---|---|---|---|---|---|---|---|---|---|---|
| 0 | None | None | None | None | None | None | None | None | None | None | None | None | None | None | None | None | None | None | None | None |
| 2.0000000000000007E-2 | 0.022727272727272717 | 0.0 | 0.0209424083769634 | 0.0 | None | 0.0858895705521472 | 0.030075187969924817 | 0.027522935779816515 | 0.02453987730061352 | 0.0202020202020202 | 0.022099447513812213 | 0.12921348314606715 | 0.125581395348837 | 0.0298507462686567 | 0.009900990099009906 | 0.02678571428571432 | 0.056790123456790104 | 0.0352564102564103 | 0.07185628742514973 | 0.0 |
| 4.0000000000000015E-2 | 0.0 | 0.007142857142857142 | 0.005235602094240845 | 0.005405405405405412 | None | 0.03680981595092022 | 0.030075187969924817 | 0.055045871559633 | 0.02453987730061352 | 0.005050505050505048 | 0.011049723756906101 | 0.07303370786516852 | 0.0883720930232558 | 0.009950248756218917 | 0.01980198019801981 | 0.02678571428571432 | 0.037037037037037014 | 0.032051282051282 | 0.05389221556886231 | 0.0233918128654971 |
| 6.0000000000000019E-2 | 0.0 | 0.007142857142857142 | 0.0 | 0.010810810810810801 | None | 0.030674846625766916 | 0.022556390977443608 | 0.00917431192660551 | 0.012269938650306698 | 0.0202020202020202 | 0.01657458563535911 | 0.022471910112359623 | 0.0511627906976744 | 0.009950248756218917 | 0.0 | 0.00446428571428571 | 0.034567901234567884 | 0.022435897435897415 | 0.04790419161676651 | 0.0116959064327485 |
| 8.0000000000000029E-2 | 0.0 | 0.007142857142857142 | 0.0 | 0.0 | None | 0.03680981595092022 | 0.0150375939849624 | 0.018348623853211 | 0.012269938650306698 | 0.0303030303030303 | 0.01657458563535911 | 0.028089887640449406 | 0.0325581395348837 | 0.009950248756218917 | 0.0 | 0.0223214285714286 | 0.0740740740740741 | 0.0384615384615385 | 0.07784431137724551 | 0.0116959064327485 |
| 0.1 | 0.0 | 0.007142857142857142 | 0.0 | 0.0 | None | 0.012269938650306698 | 0.0075187969924812035 | 0.0 | 0.0061349693251533735 | 0.0101010101010101 | 0.0055248618784530384 | 0.03932584269662921 | 0.037209302325581416 | 0.004975124378109448 | 0.004950495049504951 | 0.013392857142857107 | 0.049382716049382734 | 0.02564102564102561 | 0.029940119760479014 | 0.0116959064327485 |
| 0.12000000000000002 | 0.0 | 0.014285714285714301 | 0.005235602094240845 | 0.0 | None | 0.012269938650306698 | 0.0075187969924812035 | 0.0 | 0.030674846625766916 | 0.0101010101010101 | 0.0 | 0.03370786516853931 | 0.0186046511627907 | 0.0 | 0.014851485148514901 | 0.013392857142857107 | 0.056790123456790104 | 0.0416666666666667 | 0.0419161676646707 | 0.0116959064327485 |
| 0.14000000000000001 | 0.0 | 0.0 | 0.0 | 0.0 | None | 0.0184049079754601 | 0.0 | 0.00917431192660551 | 0.0 | 0.005050505050505048 | 0.0 | 0.0168539325842697 | 0.00930232558139535 | 0.009950248756218917 | 0.0 | 0.00446428571428571 | 0.06419753086419752 | 0.019230769230769208 | 0.011976047904191595 | 0.0 |
| 0.16 | 0.0 | 0.0 | 0.0 | 0.0 | None | 0.0061349693251533735 | 0.0150375939849624 | 0.00917431192660551 | 0.0 | 0.0 | 0.0055248618784530384 | 0.03932584269662921 | 0.027906976744186 | 0.0 | 0.004950495049504951 | 0.0223214285714286 | 0.0740740740740741 | 0.0416666666666667 | 0.05389221556886231 | 0.0 |
| 0.18000000000000005 | 0.022727272727272717 | 0.021428571428571408 | 0.005235602094240845 | 0.0 | None | 0.030674846625766916 | 0.0150375939849624 | 0.0 | 0.0 | 0.005050505050505048 | 0.0055248618784530384 | 0.03370786516853931 | 0.0186046511627907 | 0.004975124378109448 | 0.009900990099009906 | 0.008928571428571423 | 0.07654320987654321 | 0.016025641025641 | 0.011976047904191595 | 0.017543859649122813 |
| 0.2 | 0.022727272727272717 | 0.014285714285714301 | 0.010471204188481699 | 0.0 | None | 0.02453987730061352 | 0.0150375939849624 | 0.0 | 0.0 | 0.015151515151515204 | 0.011049723756906101 | 0.028089887640449406 | 0.0232558139534884 | 0.009950248756218917 | 0.004950495049504951 | 0.013392857142857107 | 0.07160493827160487 | 0.022435897435897415 | 0.023952095808383197 | 0.00584795321637427 |
| 0.22 | 0.0 | 0.0357142857142857 | 0.0209424083769634 | 0.0 | None | 0.0184049079754601 | 0.0075187969924812035 | 0.018348623853211 | 0.0061349693251533735 | 0.0202020202020202 | 0.03867403314917131 | 0.0449438202247191 | 0.027906976744186 | 0.009950248756218917 | 0.004950495049504951 | 0.00446428571428571 | 0.056790123456790104 | 0.02564102564102561 | 0.005988023952095812 | 0.0233918128654971 |
| 0.24000000000000005 | 0.022727272727272717 | 0.0357142857142857 | 0.03141361256544502 | 0.005405405405405412 | None | 0.012269938650306698 | 0.037593984962406 | 0.00917431192660551 | 0.012269938650306698 | 0.0202020202020202 | 0.022099447513812213 | 0.0168539325842697 | 0.0232558139534884 | 0.0 | 0.009900990099009906 | 0.008928571428571423 | 0.02469135802469141 | 0.032051282051282 | 0.005988023952095812 | 0.0116959064327485 |
| 0.26 | 0.0 | 0.0642857142857143 | 0.03664921465968592 | 0.010810810810810801 | None | 0.02453987730061352 | 0.022556390977443608 | 0.00917431192660551 | 0.0184049079754601 | 0.0101010101010101 | 0.04972375690607732 | 0.028089887640449406 | 0.0325581395348837 | 0.014925373134328401 | 0.014851485148514901 | 0.008928571428571423 | 0.0271604938271605 | 0.0352564102564103 | 0.029940119760479014 | 0.0116959064327485 |
| 0.28000000000000008 | 0.022727272727272717 | 0.07142857142857141 | 0.06282722513089 | 0.0486486486486487 | None | 0.049079754601227 | 0.0150375939849624 | 0.0642201834862385 | 0.012269938650306698 | 0.015151515151515204 | 0.055248618784530384 | 0.028089887640449406 | 0.0186046511627907 | 0.01990049751243781 | 0.0495049504950495 | 0.00446428571428571 | 0.034567901234567884 | 0.0416666666666667 | 0.023952095808383197 | 0.017543859649122813 |
| 0.3000000000000001 | 0.022727272727272717 | 0.0642857142857143 | 0.10994764397905803 | 0.10270270270270303 | None | 0.0429447852760736 | 0.052631578947368404 | 0.09174311926605508 | 0.030674846625766916 | 0.0404040404040404 | 0.0718232044198895 | 0.050561797752809015 | 0.0186046511627907 | 0.03980099502487562 | 0.0792079207920792 | 0.0223214285714286 | 0.0320987654320988 | 0.06410256410256411 | 0.05389221556886231 | 0.0701754385964912 |
| 0.32000000000000012 | 0.0681818181818182 | 0.04285714285714291 | 0.10994764397905803 | 0.0648648648648649 | None | 0.0674846625766871 | 0.037593984962406 | 0.0642201834862385 | 0.0429447852760736 | 0.0404040404040404 | 0.0939226519337017 | 0.03932584269662921 | 0.0232558139534884 | 0.049751243781094495 | 0.08910891089108916 | 0.0223214285714286 | 0.034567901234567884 | 0.10576923076923106 | 0.035928143712574814 | 0.08187134502923986 |
| 0.34 | 0.11363636363636398 | 0.07142857142857141 | 0.146596858638743 | 0.11351351351351402 | None | 0.030674846625766916 | 0.030075187969924817 | 0.045871559633027484 | 0.0858895705521472 | 0.0353535353535354 | 0.060773480662983416 | 0.028089887640449406 | 0.00930232558139535 | 0.05970149253731343 | 0.0841584158415842 | 0.02678571428571432 | 0.0320987654320988 | 0.0384615384615385 | 0.08982035928143715 | 0.08187134502923986 |
| 0.3600000000000001 | 0.11363636363636398 | 0.0785714285714286 | 0.10994764397905803 | 0.156756756756757 | None | 0.07361963190184054 | 0.052631578947368404 | 0.045871559633027484 | 0.0674846625766871 | 0.0757575757575758 | 0.055248618784530384 | 0.028089887640449406 | 0.0232558139534884 | 0.06467661691542292 | 0.07425742574257431 | 0.02678571428571432 | 0.0271604938271605 | 0.0897435897435897 | 0.07784431137724551 | 0.105263157894737 |
| 0.38000000000000012 | 0.0681818181818182 | 0.0857142857142857 | 0.0994764397905759 | 0.14594594594594607 | None | 0.012269938650306698 | 0.08270676691729324 | 0.045871559633027484 | 0.0920245398773006 | 0.05555555555555558 | 0.04972375690607732 | 0.022471910112359623 | 0.013953488372093 | 0.119402985074627 | 0.09900990099009907 | 0.07589285714285712 | 0.034567901234567884 | 0.044871794871794914 | 0.0419161676646707 | 0.07602339181286552 |
| 0.4 | 0.15909090909090906 | 0.04285714285714291 | 0.03664921465968592 | 0.0972972972972973 | None | 0.03680981595092022 | 0.04511278195488724 | 0.0642201834862385 | 0.0674846625766871 | 0.0858585858585859 | 0.0662983425414365 | 0.0 | 0.00930232558139535 | 0.054726368159204015 | 0.08910891089108916 | 0.111607142857143 | 0.0148148148148148 | 0.044871794871794914 | 0.029940119760479014 | 0.052631578947368404 |
| 0.4200000000000001 | 0.0454545454545455 | 0.07142857142857141 | 0.057591623036649234 | 0.0594594594594595 | None | 0.02453987730061352 | 0.06015037593984962 | 0.03669724770642203 | 0.09815950920245403 | 0.0808080808080808 | 0.022099447513812213 | 0.0 | 0.00930232558139535 | 0.08955223880597013 | 0.08910891089108916 | 0.08482142857142867 | 0.009876543209876548 | 0.0352564102564103 | 0.017964071856287407 | 0.0701754385964912 |
| 0.44 | 0.022727272727272717 | 0.014285714285714301 | 0.03141361256544502 | 0.0432432432432432 | None | 0.0184049079754601 | 0.06015037593984962 | 0.045871559633027484 | 0.0674846625766871 | 0.0707070707070707 | 0.022099447513812213 | 0.005617977528089888 | 0.0 | 0.0845771144278607 | 0.059405940594059396 | 0.0535714285714286 | 0.012345679012345704 | 0.0352564102564103 | 0.005988023952095812 | 0.087719298245614 |
| 0.46 | 0.09090909090909094 | 0.0357142857142857 | 0.026178010471204216 | 0.03783783783783781 | None | 0.0184049079754601 | 0.06766917293233081 | 0.018348623853211 | 0.03680981595092022 | 0.0656565656565657 | 0.04972375690607732 | 0.0 | 0.013953488372093 | 0.05970149253731343 | 0.0346534653465347 | 0.040178571428571404 | 0.009876543209876548 | 0.016025641025641 | 0.0419161676646707 | 0.029239766081871312 |
| 0.48000000000000009 | 0.0454545454545455 | 0.021428571428571408 | 0.026178010471204216 | 0.03783783783783781 | None | 0.02453987730061352 | 0.030075187969924817 | 0.045871559633027484 | 0.03680981595092022 | 0.0454545454545455 | 0.027624309392265206 | 0.0 | 0.0186046511627907 | 0.03482587064676621 | 0.0346534653465347 | 0.0535714285714286 | 0.004938271604938274 | 0.012820512820512801 | 0.005988023952095812 | 0.040935672514619895 |
| 0.5 | 0.022727272727272717 | 0.0357142857142857 | 0.015706806282722505 | 0.0162162162162162 | None | 0.0184049079754601 | 0.022556390977443608 | 0.018348623853211 | 0.03680981595092022 | 0.0606060606060606 | 0.01657458563535911 | 0.0 | 0.0046511627906976735 | 0.0298507462686567 | 0.014851485148514901 | 0.0535714285714286 | 0.002469135802469141 | 0.0 | 0.005988023952095812 | 0.0233918128654971 |
| 0.52 | 0.022727272727272717 | 0.014285714285714301 | 0.005235602094240845 | 0.010810810810810801 | None | 0.0061349693251533735 | 0.022556390977443608 | 0.00917431192660551 | 0.0429447852760736 | 0.025252525252525308 | 0.0055248618784530384 | 0.0 | 0.0 | 0.01990049751243781 | 0.03960396039603961 | 0.0357142857142857 | 0.002469135802469141 | 0.006410256410256412 | 0.005988023952095812 | 0.029239766081871312 |
| 0.54 | 0.0 | 0.028571428571428602 | 0.0 | 0.010810810810810801 | None | 0.0061349693251533735 | 0.04511278195488724 | 0.018348623853211 | 0.012269938650306698 | 0.015151515151515204 | 0.01657458563535911 | 0.0 | 0.0046511627906976735 | 0.0298507462686567 | 0.009900990099009906 | 0.03125 | 0.0 | 0.006410256410256412 | 0.005988023952095812 | 0.0233918128654971 |
| 0.56000000000000005 | 0.0 | 0.021428571428571408 | 0.005235602094240845 | 0.010810810810810801 | None | 0.0061349693251533735 | 0.022556390977443608 | 0.018348623853211 | 0.030674846625766916 | 0.0202020202020202 | 0.01657458563535911 | 0.0 | 0.0 | 0.014925373134328401 | 0.004950495049504951 | 0.0223214285714286 | 0.0 | 0.0032051282051282107 | 0.005988023952095812 | 0.0116959064327485 |
| 0.58000000000000007 | 0.0 | 0.014285714285714301 | 0.005235602094240845 | 0.005405405405405412 | None | 0.0 | 0.030075187969924817 | 0.027522935779816515 | 0.0061349693251533735 | 0.0101010101010101 | 0.011049723756906101 | 0.0 | 0.0046511627906976735 | 0.014925373134328401 | 0.0 | 0.044642857142857095 | 0.0 | 0.006410256410256412 | 0.0 | 0.0 |
| 0.6000000000000002 | 0.022727272727272717 | 0.0 | 0.010471204188481699 | 0.0 | None | 0.0 | 0.0075187969924812035 | 0.00917431192660551 | 0.0184049079754601 | 0.0202020202020202 | 0.0 | 0.0 | 0.0046511627906976735 | 0.014925373134328401 | 0.004950495049504951 | 0.017857142857142898 | 0.0 | 0.0 | 0.005988023952095812 | 0.00584795321637427 |
### Chart: nmr-1 A1
| Category | nmr-1(ak4)_A1_01 | nmr-1(ak4)_A1_02 | nmr-1(ak4)_A1_03 | nmr-1(ak4)_A1_04 | nmr-1(ak4)_A1_05 | nmr-1(ak4)_A1_06 | nmr-1(ak4)_A1_07 | nmr-1(ak4)_A1_08 | nmr-1(ak4)_A1_09 | nmr-1(ak4)_A1_10 | nmr-1(ak4)_A1_11 | nmr-1(ak4)_A1_12 | nmr-1(ak4)_A1_13 | nmr-1(ak4)_A1_14 | nmr-1(ak4)_A1_15 | nmr-1(ak4)_A1_16 | nmr-1(ak4)_A1_17 | nmr-1(ak4)_A1_18 | nmr-1(ak4)_A1_20 |
|---|---|---|---|---|---|---|---|---|---|---|---|---|---|---|---|---|---|---|---|
| 0 | None | None | None | None | None | None | None | None | None | None | None | None | None | None | None | None | None | None | None |
| 2.0000000000000007E-2 | 0.05545617173524152 | 0.044897959183673515 | 0.06065318818040438 | 0.038696537678207715 | 0.05405405405405411 | 0.052631578947368404 | 0.0649350649350649 | 0.0460829493087558 | 0.06463195691202873 | 0.0611510791366906 | 0.049309664694280116 | 0.0274390243902439 | 0.03989361702127662 | 0.047945205479452066 | 0.03403141361256542 | 0.07103825136612021 | 0.04815864022662891 | 0.053949903660886284 | 0.030000000000000002 |
| 4.0000000000000015E-2 | 0.051878354203935585 | 0.063265306122449 | 0.06998444790046662 | 0.06313645621181263 | 0.06081081081081082 | 0.04655870445344132 | 0.0426716141001855 | 0.06221198156682032 | 0.05206463195691206 | 0.0449640287769784 | 0.05719921104536491 | 0.0335365853658537 | 0.04521276595744684 | 0.0342465753424658 | 0.03403141361256542 | 0.060109289617486315 | 0.014164305949008504 | 0.06936416184971106 | 0.03500000000000001 |
| 6.0000000000000019E-2 | 0.0715563506261181 | 0.04285714285714291 | 0.0668740279937792 | 0.05702647657841142 | 0.0675675675675676 | 0.044534412955465626 | 0.0333951762523191 | 0.029953917050691208 | 0.0610412926391382 | 0.06474820143884893 | 0.053254437869822514 | 0.039634146341463415 | 0.026595744680851106 | 0.04452054794520552 | 0.013089005235602108 | 0.05464480874316937 | 0.014164305949008504 | 0.04624277456647398 | 0.037500000000000006 |
| 8.0000000000000029E-2 | 0.0661896243291592 | 0.0571428571428571 | 0.057542768273716995 | 0.0712830957230143 | 0.04729729729729732 | 0.04655870445344132 | 0.048237476808905416 | 0.041474654377880185 | 0.0736086175942549 | 0.05035971223021581 | 0.07889546351084813 | 0.04268292682926834 | 0.0212765957446809 | 0.0342465753424658 | 0.0235602094240838 | 0.0437158469945355 | 0.016997167138810203 | 0.06165703275529871 | 0.025 |
| 0.1 | 0.042933810375670796 | 0.04897959183673472 | 0.07776049766718512 | 0.0733197556008147 | 0.057432432432432436 | 0.052631578947368404 | 0.0630797773654917 | 0.029953917050691208 | 0.06822262118491923 | 0.0611510791366906 | 0.06114398422090731 | 0.0335365853658537 | 0.04521276595744684 | 0.023972602739726002 | 0.018324607329842903 | 0.0491803278688525 | 0.03682719546742212 | 0.050096339113680236 | 0.047500000000000014 |
| 0.12000000000000002 | 0.0644007155635063 | 0.04285714285714291 | 0.062208398133748115 | 0.050916496945010256 | 0.0658783783783784 | 0.05060728744939272 | 0.048237476808905416 | 0.0552995391705069 | 0.05745062836624781 | 0.0755395683453237 | 0.0729783037475345 | 0.039634146341463415 | 0.026595744680851106 | 0.023972602739726002 | 0.018324607329842903 | 0.0437158469945355 | 0.02266288951841361 | 0.053949903660886284 | 0.022500000000000006 |
| 0.14000000000000001 | 0.06261180679785329 | 0.051020408163265286 | 0.04976671850699843 | 0.06924643584521392 | 0.0658783783783784 | 0.06477732793522274 | 0.05936920222634512 | 0.0552995391705069 | 0.04488330341113112 | 0.0449640287769784 | 0.0414201183431953 | 0.015243902439024399 | 0.031914893617021316 | 0.023972602739726002 | 0.0209424083769634 | 0.0327868852459016 | 0.02266288951841361 | 0.06165703275529871 | 0.022500000000000006 |
| 0.16 | 0.059033989266547425 | 0.06938775510204083 | 0.0404354587869362 | 0.059063136456211834 | 0.04729729729729732 | 0.026315789473684202 | 0.0686456400742115 | 0.0552995391705069 | 0.050269299820466816 | 0.0611510791366906 | 0.0355029585798817 | 0.0213414634146341 | 0.031914893617021316 | 0.0171232876712329 | 0.018324607329842903 | 0.010928961748633904 | 0.011331444759206801 | 0.05587668593448942 | 0.027500000000000007 |
| 0.18000000000000005 | 0.0483005366726297 | 0.063265306122449 | 0.05909797822706072 | 0.04073319755600812 | 0.05405405405405411 | 0.0688259109311741 | 0.038961038961039 | 0.05760368663594473 | 0.0736086175942549 | 0.0359712230215827 | 0.0512820512820513 | 0.04878048780487813 | 0.04521276595744684 | 0.058219178082191785 | 0.03926701570680632 | 0.060109289617486315 | 0.025495750708215314 | 0.0674373795761079 | 0.0325 |
| 0.2 | 0.0661896243291592 | 0.04897959183673472 | 0.0404354587869362 | 0.054989816700611 | 0.045608108108108086 | 0.0384615384615385 | 0.0705009276437848 | 0.0552995391705069 | 0.05206463195691206 | 0.052158273381295 | 0.04339250493096654 | 0.04573170731707321 | 0.031914893617021316 | 0.05136986301369861 | 0.03141361256544502 | 0.07650273224043723 | 0.0311614730878187 | 0.0732177263969171 | 0.037500000000000006 |
| 0.22 | 0.0536672629695885 | 0.0653061224489796 | 0.0404354587869362 | 0.046843177189409384 | 0.04054054054054049 | 0.052631578947368404 | 0.055658627087198514 | 0.07603686635944702 | 0.05924596050269298 | 0.057553956834532426 | 0.05522682445759372 | 0.039634146341463415 | 0.0531914893617021 | 0.0684931506849315 | 0.049738219895288024 | 0.0382513661202186 | 0.056657223796033995 | 0.03853564547206172 | 0.04500000000000001 |
| 0.24000000000000005 | 0.0500894454382827 | 0.055102040816326525 | 0.062208398133748115 | 0.05702647657841142 | 0.045608108108108086 | 0.0708502024291498 | 0.051948051948052 | 0.052995391705069096 | 0.04308797127468581 | 0.048561151079136715 | 0.023668639053254392 | 0.05487804878048778 | 0.058510638297872314 | 0.0684931506849315 | 0.04712041884816751 | 0.05464480874316937 | 0.06232294617563742 | 0.05587668593448942 | 0.085 |
| 0.26 | 0.04651162790697674 | 0.0571428571428571 | 0.03265940902021772 | 0.0325865580448065 | 0.0523648648648649 | 0.04655870445344132 | 0.046382189239332114 | 0.052995391705069096 | 0.04129263913824063 | 0.052158273381295 | 0.047337278106508916 | 0.076219512195122 | 0.0984042553191489 | 0.08904109589041104 | 0.10732984293193704 | 0.0491803278688525 | 0.07365439093484423 | 0.0481695568400771 | 0.06000000000000002 |
| 0.28000000000000008 | 0.030411449016100208 | 0.04693877551020408 | 0.0357698289269051 | 0.03462321792260691 | 0.0371621621621622 | 0.08502024291497985 | 0.0649350649350649 | 0.05760368663594473 | 0.034111310592459615 | 0.05575539568345319 | 0.049309664694280116 | 0.06402439024390241 | 0.06382978723404252 | 0.07876712328767123 | 0.07329842931937172 | 0.0382513661202186 | 0.07365439093484423 | 0.05202312138728321 | 0.0675 |
| 0.3000000000000001 | 0.028622540250447193 | 0.04897959183673472 | 0.0279937791601866 | 0.04276985743380862 | 0.045608108108108086 | 0.0546558704453441 | 0.051948051948052 | 0.07373271889400923 | 0.0269299820466786 | 0.052158273381295 | 0.0335305719921105 | 0.0884146341463415 | 0.07978723404255325 | 0.08904109589041104 | 0.08115183246073303 | 0.05464480874316937 | 0.09065155807365445 | 0.03853564547206172 | 0.10249999999999998 |
| 0.32000000000000012 | 0.030411449016100208 | 0.044897959183673515 | 0.03265940902021772 | 0.0325865580448065 | 0.0371621621621622 | 0.026315789473684202 | 0.0371057513914657 | 0.0483870967741935 | 0.035906642728904814 | 0.0413669064748201 | 0.0355029585798817 | 0.0701219512195122 | 0.05585106382978721 | 0.047945205479452066 | 0.07591623036649213 | 0.0382513661202186 | 0.059490084985835745 | 0.0366088631984586 | 0.0675 |
| 0.34 | 0.02683363148479432 | 0.020408163265306107 | 0.0248833592534992 | 0.0203665987780041 | 0.027027027027027015 | 0.03643724696356281 | 0.0148423005565863 | 0.0230414746543779 | 0.019748653500897703 | 0.03237410071942452 | 0.01972386587771201 | 0.0579268292682927 | 0.0664893617021277 | 0.04452054794520552 | 0.07591623036649213 | 0.0327868852459016 | 0.07648725212464591 | 0.04046242774566472 | 0.057500000000000016 |
| 0.3600000000000001 | 0.017889087656529502 | 0.012244897959183699 | 0.023328149300155494 | 0.03462321792260691 | 0.0219594594594595 | 0.0182186234817814 | 0.0241187384044527 | 0.020737327188940117 | 0.02333931777378821 | 0.01978417266187051 | 0.0335305719921105 | 0.05487804878048778 | 0.0531914893617021 | 0.03767123287671233 | 0.02879581151832461 | 0.0491803278688525 | 0.05382436260623229 | 0.017341040462427702 | 0.04500000000000001 |
| 0.38000000000000012 | 0.010733452593917704 | 0.020408163265306107 | 0.018662519440124408 | 0.012219959266802404 | 0.020270270270270313 | 0.01417004048583 | 0.009276437847866427 | 0.013824884792626705 | 0.014362657091561901 | 0.0341726618705036 | 0.01972386587771201 | 0.039634146341463415 | 0.029255319148936202 | 0.03767123287671233 | 0.06282722513089 | 0.021857923497267808 | 0.0708215297450425 | 0.013487475915221604 | 0.0325 |
| 0.4 | 0.014311270125223598 | 0.020408163265306107 | 0.0248833592534992 | 0.026476578411405313 | 0.015202702702702704 | 0.03036437246963561 | 0.029684601113172508 | 0.02534562211981571 | 0.0251346499102334 | 0.0179856115107914 | 0.023668639053254392 | 0.036585365853658514 | 0.026595744680851106 | 0.020547945205479513 | 0.041884816753926704 | 0.027322404371584685 | 0.02832861189801699 | 0.007707129094412336 | 0.020000000000000007 |
| 0.4200000000000001 | 0.014311270125223598 | 0.00816326530612245 | 0.00933125972006221 | 0.008146639511201637 | 0.0219594594594595 | 0.0182186234817814 | 0.0055658627087198514 | 0.011520737327188904 | 0.016157989228007208 | 0.0071942446043165515 | 0.017751479289940808 | 0.0213414634146341 | 0.00531914893617021 | 0.020547945205479513 | 0.0235602094240838 | 0.0163934426229508 | 0.03682719546742212 | 0.015414258188824699 | 0.025 |
| 0.44 | 0.0125223613595707 | 0.016326530612244903 | 0.006220839813374812 | 0.010183299389002004 | 0.00675675675675676 | 0.010121457489878501 | 0.011131725417439705 | 0.006912442396313365 | 0.007181328545780973 | 0.003597122302158271 | 0.0157790927021696 | 0.0030487804878048808 | 0.023936170212766002 | 0.0102739726027397 | 0.018324607329842903 | 0.005464480874316938 | 0.016997167138810203 | 0.0038535645472061726 | 0.025 |
| 0.46 | 0.00536672629695885 | 0.00816326530612245 | 0.0046656298600311 | 0.004073319755600813 | 0.008445945945945955 | 0.004048582995951422 | 0.012987012987013 | 0.006912442396313365 | 0.007181328545780973 | 0.00179856115107914 | 0.009861932938856025 | 0.0 | 0.0026595744680851107 | 0.006849315068493152 | 0.015706806282722505 | 0.021857923497267808 | 0.016997167138810203 | 0.0 | 0.014999999999999998 |
| 0.48000000000000009 | 0.00536672629695885 | 0.00612244897959184 | 0.003110419906687401 | 0.004073319755600813 | 0.0016891891891891904 | 0.004048582995951422 | 0.0018552875695732817 | 0.002304147465437791 | 0.001795332136445241 | 0.00179856115107914 | 0.005917159763313612 | 0.0 | 0.0026595744680851107 | 0.003424657534246581 | 0.0 | 0.010928961748633904 | 0.0056657223796034 | 0.005780346820809252 | 0.002500000000000001 |
| 0.5 | 0.0017889087656529504 | 0.00204081632653061 | 0.0015552099533437005 | 0.00203665987780041 | 0.0016891891891891904 | 0.0 | 0.0018552875695732817 | 0.002304147465437791 | 0.007181328545780973 | 0.0 | 0.009861932938856025 | 0.0 | 0.0 | 0.0 | 0.010471204188481699 | 0.005464480874316938 | 0.008498583569405103 | 0.0 | 0.005000000000000002 |
| 0.52 | 0.0 | 0.0 | 0.0 | 0.00203665987780041 | 0.0 | 0.002024291497975711 | 0.0018552875695732817 | 0.002304147465437791 | 0.001795332136445241 | 0.0 | 0.005917159763313612 | 0.00609756097560976 | 0.0026595744680851107 | 0.0 | 0.0026178010471204216 | 0.005464480874316938 | 0.0 | 0.0019267822736030809 | 0.0 |
| 0.54 | 0.0 | 0.00204081632653061 | 0.0046656298600311 | 0.0 | 0.0016891891891891904 | 0.0 | 0.0 | 0.0 | 0.001795332136445241 | 0.0 | 0.003944773175542412 | 0.0030487804878048808 | 0.0 | 0.0 | 0.0 | 0.0 | 0.0 | 0.0 | 0.002500000000000001 |
| 0.56000000000000005 | 0.0017889087656529504 | 0.0 | 0.0015552099533437005 | 0.0 | 0.0 | 0.006072874493927133 | 0.0018552875695732817 | 0.002304147465437791 | 0.0 | 0.0 | 0.003944773175542412 | 0.0 | 0.0 | 0.0 | 0.0 | 0.0 | 0.0 | 0.0 | 0.0 |
| 0.58000000000000007 | 0.0 | 0.0 | 0.0 | 0.0 | 0.0016891891891891904 | 0.0 | 0.0 | 0.0 | 0.0 | 0.0 | 0.0 | 0.0 | 0.0 | 0.0 | 0.0 | 0.0 | 0.0 | 0.0 | 0.0 |
| 0.6000000000000002 | 0.0 | 0.0 | 0.0 | 0.0 | 0.0 | 0.0 | 0.0 | 0.0 | 0.0 | 0.0 | 0.0 | 0.0 | 0.0 | 0.0 | 0.0 | 0.0 | 0.0 | 0.0 | 0.0 |
### Chart: nmr-1 A3
| Category | nmr-1(ak4)_A3_01 | nmr-1(ak4)_A3_02 | nmr-1(ak4)_A3_03 | nmr-1(ak4)_A3_04 | nmr-1(ak4)_A3_05 | nmr-1(ak4)_A3_06 | nmr-1(ak4)_A3_07 | nmr-1(ak4)_A3_08 | nmr-1(ak4)_A3_09 | nmr-1(ak4)_A3_10 | nmr-1(ak4)_A3_11 | nmr-1(ak4)_A3_12 | nmr-1(ak4)_A3_13 | nmr-1(ak4)_A3_14 | nmr-1(ak4)_A3_15 | nmr-1(ak4)_A3_16 | nmr-1(ak4)_A3_17 | nmr-1(ak4)_A3_18 | nmr-1(ak4)_A3_19 | nmr-1(ak4)_A3_20 |
|---|---|---|---|---|---|---|---|---|---|---|---|---|---|---|---|---|---|---|---|---|
| 0 | None | None | None | None | None | None | None | None | None | None | None | None | None | None | None | None | None | None | None | None |
| 2.0000000000000007E-2 | 0.052478134110787215 | 0.04411764705882354 | 0.0 | 0.0 | 0.008583690987124463 | 0.010752688172042998 | 0.00353356890459364 | 0.009950248756218917 | 0.00986842105263158 | 0.003355704697986581 | 0.0427350427350427 | 0.004048582995951422 | 0.007692307692307692 | 0.005813953488372092 | 0.015625 | 0.004329004329004332 | 0.0 | 0.0168350168350168 | 0.0144404332129964 | 0.00970873786407767 |
| 4.0000000000000015E-2 | 0.0145772594752187 | 0.03823529411764711 | 0.0 | 0.012345679012345704 | 0.004291845493562234 | 0.005376344086021512 | 0.0 | 0.0 | 0.0 | 0.003355704697986581 | 0.0284900284900285 | 0.0 | 0.0 | 0.040697674418604717 | 0.023437500000000007 | 0.0 | 0.0 | 0.006734006734006732 | 0.0144404332129964 | 0.0 |
| 6.0000000000000019E-2 | 0.020408163265306107 | 0.011764705882352905 | 0.0 | 0.012345679012345704 | 0.004291845493562234 | 0.010752688172042998 | 0.0 | 0.004975124378109448 | 0.003289473684210531 | 0.003355704697986581 | 0.019943019943019908 | 0.0 | 0.007692307692307692 | 0.005813953488372092 | 0.015625 | 0.0 | 0.0 | 0.003367003367003371 | 0.0036101083032491 | 0.004854368932038832 |
| 8.0000000000000029E-2 | 0.00291545189504373 | 0.02352941176470589 | 0.004878048780487803 | 0.0 | 0.008583690987124463 | 0.010752688172042998 | 0.00353356890459364 | 0.014925373134328401 | 0.003289473684210531 | 0.0 | 0.011396011396011404 | 0.0 | 0.00384615384615385 | 0.0348837209302326 | 0.0 | 0.0 | 0.0 | 0.006734006734006732 | 0.0 | 0.004854368932038832 |
| 0.1 | 0.00291545189504373 | 0.014705882352941199 | 0.009756097560975615 | 0.012345679012345704 | 0.012875536480686699 | 0.005376344086021512 | 0.0 | 0.004975124378109448 | 0.0 | 0.0 | 0.019943019943019908 | 0.0 | 0.0 | 0.011627906976744195 | 0.0 | 0.004329004329004332 | 0.0 | 0.003367003367003371 | 0.0 | 0.004854368932038832 |
| 0.12000000000000002 | 0.0058309037900874635 | 0.005882352941176471 | 0.004878048780487803 | 0.012345679012345704 | 0.0 | 0.005376344086021512 | 0.0 | 0.004975124378109448 | 0.0 | 0.0 | 0.00854700854700855 | 0.0 | 0.00384615384615385 | 0.017441860465116307 | 0.0078125 | 0.004329004329004332 | 0.0 | 0.0 | 0.0 | 0.0 |
| 0.14000000000000001 | 0.017492711370262405 | 0.008823529411764714 | 0.004878048780487803 | 0.0 | 0.004291845493562234 | 0.0 | 0.0 | 0.0 | 0.0 | 0.003355704697986581 | 0.00854700854700855 | 0.0 | 0.0 | 0.005813953488372092 | 0.0 | 0.0 | 0.0 | 0.0 | 0.0036101083032491 | 0.0 |
| 0.16 | 0.029154518950437285 | 0.026470588235294107 | 0.0 | 0.0 | 0.004291845493562234 | 0.0 | 0.00353356890459364 | 0.009950248756218917 | 0.0 | 0.0 | 0.014245014245014204 | 0.0 | 0.0 | 0.0 | 0.0078125 | 0.0 | 0.0 | 0.0269360269360269 | 0.0 | 0.0 |
| 0.18000000000000005 | 0.017492711370262405 | 0.02352941176470589 | 0.004878048780487803 | 0.012345679012345704 | 0.008583690987124463 | 0.0 | 0.00353356890459364 | 0.004975124378109448 | 0.006578947368421051 | 0.0 | 0.00854700854700855 | 0.0 | 0.0 | 0.0232558139534884 | 0.0 | 0.0 | 0.0 | 0.0 | 0.0 | 0.0 |
| 0.2 | 0.029154518950437285 | 0.0176470588235294 | 0.004878048780487803 | 0.0 | 0.0 | 0.005376344086021512 | 0.0141342756183746 | 0.0248756218905473 | 0.003289473684210531 | 0.006711409395973156 | 0.0056980056980057 | 0.004048582995951422 | 0.00384615384615385 | 0.005813953488372092 | 0.0078125 | 0.008658008658008665 | 0.0075187969924812035 | 0.003367003367003371 | 0.0180505415162455 | 0.0 |
| 0.22 | 0.0379008746355685 | 0.026470588235294107 | 0.004878048780487803 | 0.049382716049382734 | 0.012875536480686699 | 0.03763440860215051 | 0.038869257950530006 | 0.0696517412935323 | 0.03618421052631582 | 0.040268456375838896 | 0.0227920227920228 | 0.0161943319838057 | 0.019230769230769208 | 0.017441860465116307 | 0.015625 | 0.025974025974026007 | 0.0488721804511278 | 0.006734006734006732 | 0.0144404332129964 | 0.004854368932038832 |
| 0.24000000000000005 | 0.04081632653061222 | 0.04411764705882354 | 0.04390243902439023 | 0.02469135802469141 | 0.0171673819742489 | 0.02688172043010751 | 0.0918727915194346 | 0.10447761194029903 | 0.04605263157894742 | 0.07718120805369133 | 0.031339031339031286 | 0.060728744939271335 | 0.115384615384615 | 0.017441860465116307 | 0.03125 | 0.0692640692640693 | 0.09022556390977447 | 0.0202020202020202 | 0.0722021660649819 | 0.06796116504854373 |
| 0.26 | 0.0699708454810496 | 0.0735294117647059 | 0.09268292682926828 | 0.0617283950617284 | 0.05579399141630904 | 0.03763440860215051 | 0.15547703180212016 | 0.144278606965174 | 0.115131578947368 | 0.0939597315436242 | 0.0512820512820513 | 0.11336032388664002 | 0.17692307692307693 | 0.02906976744186051 | 0.0390625 | 0.112554112554113 | 0.131578947368421 | 0.0774410774410774 | 0.11913357400722004 | 0.111650485436893 |
| 0.28000000000000008 | 0.07288629737609335 | 0.0735294117647059 | 0.131707317073171 | 0.08641975308641968 | 0.09442060085836915 | 0.10752688172043005 | 0.18374558303886906 | 0.1393034825870651 | 0.151315789473684 | 0.15100671140939606 | 0.0655270655270655 | 0.15789473684210512 | 0.17692307692307693 | 0.0988372093023256 | 0.1171875 | 0.18181818181818207 | 0.131578947368421 | 0.104377104377104 | 0.155234657039711 | 0.111650485436893 |
| 0.3000000000000001 | 0.102040816326531 | 0.09705882352941182 | 0.19024390243902406 | 0.17283950617283905 | 0.1545064377682401 | 0.13978494623655893 | 0.13074204946996507 | 0.124378109452736 | 0.101973684210526 | 0.130872483221477 | 0.08262108262108264 | 0.137651821862348 | 0.192307692307692 | 0.13372093023255793 | 0.140625 | 0.173160173160173 | 0.14285714285714307 | 0.111111111111111 | 0.162454873646209 | 0.131067961165049 |
| 0.32000000000000012 | 0.0845481049562682 | 0.13235294117647106 | 0.16585365853658496 | 0.12345679012345702 | 0.09871244635193134 | 0.12903225806451593 | 0.11307420494699603 | 0.09452736318407974 | 0.14144736842105307 | 0.13422818791946306 | 0.09686609686609697 | 0.17004048582996012 | 0.09230769230769234 | 0.11046511627907002 | 0.10156250000000003 | 0.18181818181818207 | 0.131578947368421 | 0.12121212121212104 | 0.14079422382671505 | 0.1941747572815529 |
| 0.34 | 0.08746355685131182 | 0.0735294117647059 | 0.10243902439024398 | 0.037037037037037014 | 0.17167381974248894 | 0.145161290322581 | 0.11307420494699603 | 0.08955223880597013 | 0.138157894736842 | 0.10402684563758405 | 0.0854700854700855 | 0.129554655870445 | 0.0884615384615385 | 0.104651162790698 | 0.125 | 0.13419913419913407 | 0.09398496240601505 | 0.0875420875420875 | 0.11191335740072197 | 0.15048543689320415 |
| 0.3600000000000001 | 0.09329446064139948 | 0.08529411764705878 | 0.08292682926829277 | 0.049382716049382734 | 0.09871244635193134 | 0.08602150537634415 | 0.05653710247349818 | 0.0298507462686567 | 0.0625 | 0.10738255033557002 | 0.0797720797720798 | 0.10931174089068803 | 0.03461538461538461 | 0.063953488372093 | 0.10937500000000003 | 0.0432900432900433 | 0.11278195488721802 | 0.0707070707070707 | 0.04693140794223834 | 0.08737864077669903 |
| 0.38000000000000012 | 0.052478134110787215 | 0.026470588235294107 | 0.024390243902439 | 0.08641975308641968 | 0.05579399141630904 | 0.059139784946236645 | 0.038869257950530006 | 0.0447761194029851 | 0.055921052631578885 | 0.063758389261745 | 0.0740740740740741 | 0.052631578947368404 | 0.03461538461538461 | 0.04651162790697674 | 0.0625 | 0.017316017316017306 | 0.037593984962406 | 0.117845117845118 | 0.05776173285198564 | 0.06310679611650491 |
| 0.4 | 0.0379008746355685 | 0.04705882352941181 | 0.034146341463414616 | 0.111111111111111 | 0.0343347639484979 | 0.05376344086021512 | 0.017667844522968202 | 0.0248756218905473 | 0.052631578947368404 | 0.030201342281879227 | 0.0655270655270655 | 0.012145748987854298 | 0.007692307692307692 | 0.0348837209302326 | 0.046874999999999986 | 0.021645021645021606 | 0.030075187969924817 | 0.0774410774410774 | 0.0180505415162455 | 0.024271844660194213 |
| 0.4200000000000001 | 0.04081632653061222 | 0.020588235294117598 | 0.009756097560975615 | 0.02469135802469141 | 0.0472103004291846 | 0.03763440860215051 | 0.007067137809187278 | 0.009950248756218917 | 0.052631578947368404 | 0.0100671140939597 | 0.054131054131054096 | 0.00809716599190283 | 0.011538461538461501 | 0.058139534883720916 | 0.054687500000000014 | 0.012987012987013 | 0.030075187969924817 | 0.047138047138047104 | 0.0072202166064982 | 0.00970873786407767 |
| 0.44 | 0.026239067055393618 | 0.026470588235294107 | 0.019512195121951202 | 0.02469135802469141 | 0.025751072961373415 | 0.021505376344086 | 0.0141342756183746 | 0.004975124378109448 | 0.006578947368421051 | 0.006711409395973156 | 0.0227920227920228 | 0.012145748987854298 | 0.0 | 0.011627906976744195 | 0.03125 | 0.0 | 0.003759398496240601 | 0.0269360269360269 | 0.0144404332129964 | 0.014563106796116504 |
| 0.46 | 0.017492711370262405 | 0.005882352941176471 | 0.019512195121951202 | 0.02469135802469141 | 0.030042918454935612 | 0.021505376344086 | 0.0 | 0.004975124378109448 | 0.006578947368421051 | 0.006711409395973156 | 0.017094017094017103 | 0.0 | 0.00384615384615385 | 0.0 | 0.0 | 0.004329004329004332 | 0.003759398496240601 | 0.0101010101010101 | 0.0036101083032491 | 0.0 |
| 0.48000000000000009 | 0.008746355685131197 | 0.0 | 0.004878048780487803 | 0.0 | 0.0 | 0.010752688172042998 | 0.0 | 0.004975124378109448 | 0.003289473684210531 | 0.006711409395973156 | 0.00854700854700855 | 0.00809716599190283 | 0.00384615384615385 | 0.011627906976744195 | 0.0 | 0.0 | 0.003759398496240601 | 0.0101010101010101 | 0.0108303249097473 | 0.0 |
| 0.5 | 0.0058309037900874635 | 0.00294117647058824 | 0.004878048780487803 | 0.012345679012345704 | 0.0 | 0.005376344086021512 | 0.0 | 0.0 | 0.0 | 0.0 | 0.017094017094017103 | 0.0 | 0.0 | 0.0 | 0.0 | 0.0 | 0.0 | 0.013468013468013504 | 0.0 | 0.0 |
| 0.52 | 0.0 | 0.00294117647058824 | 0.0 | 0.012345679012345704 | 0.0 | 0.0 | 0.0 | 0.004975124378109448 | 0.0 | 0.003355704697986581 | 0.0056980056980057 | 0.0 | 0.0 | 0.005813953488372092 | 0.0 | 0.0 | 0.0 | 0.0 | 0.0 | 0.0 |
| 0.54 | 0.0 | 0.0 | 0.004878048780487803 | 0.0 | 0.0 | 0.0 | 0.0 | 0.004975124378109448 | 0.0 | 0.0 | 0.0056980056980057 | 0.0 | 0.0 | 0.0 | 0.0078125 | 0.0 | 0.0 | 0.006734006734006732 | 0.0 | 0.0 |
| 0.56000000000000005 | 0.0 | 0.0 | 0.0 | 0.0 | 0.004291845493562234 | 0.0 | 0.0 | 0.004975124378109448 | 0.0 | 0.0 | 0.002849002849002851 | 0.0 | 0.0 | 0.0 | 0.0 | 0.0 | 0.0 | 0.0 | 0.0 | 0.0 |
| 0.58000000000000007 | 0.0 | 0.0 | 0.0 | 0.0 | 0.0 | 0.0 | 0.0 | 0.0 | 0.0 | 0.0 | 0.0 | 0.0 | 0.0 | 0.0 | 0.0 | 0.0 | 0.0 | 0.0 | 0.0 | 0.0 |
| 0.6000000000000002 | 0.0 | 0.005882352941176471 | 0.0 | 0.0 | 0.0 | 0.0 | 0.0 | 0.0 | 0.0 | 0.0 | 0.0 | 0.0 | 0.0 | 0.0 | 0.0 | 0.0 | 0.0 | 0.0 | 0.0 | 0.0 |
### Chart: nmr-1 A5
| Category | nmr-1(ak4)_A5_01 | nmr-1(ak4)_A5_02 | nmr-1(ak4)_A5_03 | nmr-1(ak4)_A5_04 | nmr-1(ak4)_A5_05 | nmr-1(ak4)_A5_06 | nmr-1(ak4)_A5_07 | nmr-1(ak4)_A5_08 | nmr-1(ak4)_A5_09 | nmr-1(ak4)_A5_10 | nmr-1(ak4)_A5_11 | nmr-1(ak4)_A5_12 | nmr-1(ak4)_A5_13 | nmr-1(ak4)_A5_14 | nmr-1(ak4)_A5_15 | nmr-1(ak4)_A5_16 | nmr-1(ak4)_A5_17 | nmr-1(ak4)_A5_18 | nmr-1(ak4)_A5_19 | nmr-1(ak4)_A5_20 |
|---|---|---|---|---|---|---|---|---|---|---|---|---|---|---|---|---|---|---|---|---|
| 0 | None | None | None | None | None | None | None | None | None | None | None | None | None | None | None | None | None | None | None | None |
| 2.0000000000000007E-2 | 0.0105263157894737 | 0.0289855072463768 | 0.005882352941176471 | 0.010362694300518104 | None | 0.004784688995215312 | 0.008888888888888894 | 0.0 | 0.010989010989011 | 0.0164835164835165 | 0.020746887966805006 | 0.0037037037037037017 | 0.0 | 0.0075471698113207504 | 0.008368200836820085 | 0.014218009478672999 | 0.018927444794952706 | 0.0207253886010363 | 0.028037383177570117 | 0.005405405405405412 |
| 4.0000000000000015E-2 | 0.0 | 0.0 | 0.0 | 0.010362694300518104 | None | 0.009569377990430622 | 0.017777777777777802 | 0.0 | 0.010989010989011 | 0.00549450549450549 | 0.024896265560166008 | 0.0 | 0.004901960784313736 | 0.0 | 0.0 | 0.014218009478672999 | 0.02839116719242901 | 0.005181347150259074 | 0.037383177570093525 | 0.010810810810810801 |
| 6.0000000000000019E-2 | 0.0 | 0.007246376811594202 | 0.0 | 0.005181347150259074 | None | 0.019138755980861205 | 0.0 | 0.0 | 0.00549450549450549 | 0.00549450549450549 | 0.020746887966805006 | 0.0037037037037037017 | 0.004901960784313736 | 0.0075471698113207504 | 0.008368200836820085 | 0.009478672985781995 | 0.018927444794952706 | 0.005181347150259074 | 0.018691588785046703 | 0.0 |
| 8.0000000000000029E-2 | 0.005263157894736842 | 0.007246376811594202 | 0.0 | 0.0 | None | 0.0 | 0.0 | 0.004950495049504951 | 0.0 | 0.010989010989011 | 0.016597510373444 | 0.011111111111111101 | 0.00980392156862745 | 0.0150943396226415 | 0.008368200836820085 | 0.009478672985781995 | 0.012618296529968496 | 0.015544041450777204 | 0.018691588785046703 | 0.005405405405405412 |
| 0.1 | 0.005263157894736842 | 0.0 | 0.0 | 0.0 | None | 0.0 | 0.004444444444444442 | 0.0 | 0.0 | 0.0 | 0.00829875518672199 | 0.0037037037037037017 | 0.0 | 0.0 | 0.00418410041841004 | 0.014218009478672999 | 0.015772870662460612 | 0.005181347150259074 | 0.009345794392523367 | 0.0 |
| 0.12000000000000002 | 0.0 | 0.0 | 0.011764705882352905 | 0.005181347150259074 | None | 0.004784688995215312 | 0.008888888888888894 | 0.004950495049504951 | 0.00549450549450549 | 0.00549450549450549 | 0.016597510373444 | 0.00740740740740741 | 0.0 | 0.0 | 0.0 | 0.014218009478672999 | 0.012618296529968496 | 0.005181347150259074 | 0.018691588785046703 | 0.005405405405405412 |
| 0.14000000000000001 | 0.0 | 0.0 | 0.0 | 0.0 | None | 0.0 | 0.004444444444444442 | 0.004950495049504951 | 0.0 | 0.010989010989011 | 0.012448132780083 | 0.0 | 0.0 | 0.0037735849056603826 | 0.0 | 0.0 | 0.015772870662460612 | 0.005181347150259074 | 0.009345794392523367 | 0.005405405405405412 |
| 0.16 | 0.0105263157894737 | 0.0 | 0.0 | 0.0 | None | 0.0 | 0.013333333333333301 | 0.014851485148514901 | 0.0 | 0.0 | 0.0 | 0.0037037037037037017 | 0.004901960784313736 | 0.0 | 0.008368200836820085 | 0.009478672985781995 | 0.009463722397476348 | 0.0207253886010363 | 0.023364485981308386 | 0.005405405405405412 |
| 0.18000000000000005 | 0.0 | 0.0 | 0.011764705882352905 | 0.0 | None | 0.0 | 0.0 | 0.004950495049504951 | 0.0164835164835165 | 0.0 | 0.0 | 0.00740740740740741 | 0.004901960784313736 | 0.0 | 0.008368200836820085 | 0.004739336492891005 | 0.012618296529968496 | 0.010362694300518104 | 0.051401869158878497 | 0.0162162162162162 |
| 0.2 | 0.0 | 0.0144927536231884 | 0.0 | 0.0 | None | 0.009569377990430622 | 0.013333333333333301 | 0.0 | 0.0 | 0.00549450549450549 | 0.00829875518672199 | 0.011111111111111101 | 0.0 | 0.0075471698113207504 | 0.03765690376569041 | 0.009478672985781995 | 0.0031545741324921117 | 0.031088082901554407 | 0.04672897196261682 | 0.0 |
| 0.22 | 0.0210526315789474 | 0.036231884057971016 | 0.0 | 0.005181347150259074 | None | 0.0 | 0.0666666666666667 | 0.009900990099009906 | 0.0384615384615385 | 0.010989010989011 | 0.024896265560166008 | 0.022222222222222202 | 0.029411764705882398 | 0.0188679245283019 | 0.02510460251046031 | 0.033175355450237004 | 0.0252365930599369 | 0.07772020725388602 | 0.06074766355140192 | 0.021621621621621602 |
| 0.24000000000000005 | 0.0210526315789474 | 0.0579710144927536 | 0.005882352941176471 | 0.015544041450777204 | None | 0.004784688995215312 | 0.04444444444444442 | 0.009900990099009906 | 0.07142857142857141 | 0.049450549450549414 | 0.03319502074688799 | 0.059259259259259296 | 0.029411764705882398 | 0.030188679245282988 | 0.0627615062761506 | 0.042654028436018995 | 0.009463722397476348 | 0.046632124352331626 | 0.11214953271028003 | 0.021621621621621602 |
| 0.26 | 0.026315789473684202 | 0.07246376811594203 | 0.0176470588235294 | 0.0207253886010363 | None | 0.019138755980861205 | 0.0666666666666667 | 0.01980198019801981 | 0.09890109890109895 | 0.07142857142857141 | 0.04564315352697097 | 0.10370370370370405 | 0.049019607843137344 | 0.0754716981132075 | 0.12552301255230106 | 0.12322274881516604 | 0.041009463722397485 | 0.07772020725388602 | 0.042056074766355096 | 0.0324324324324324 |
| 0.28000000000000008 | 0.07894736842105257 | 0.08695652173913045 | 0.029411764705882398 | 0.0414507772020725 | None | 0.057416267942583754 | 0.15111111111111106 | 0.03960396039603961 | 0.15384615384615408 | 0.115384615384615 | 0.062240663900414904 | 0.09259259259259267 | 0.09803921568627451 | 0.10188679245283003 | 0.146443514644351 | 0.06635071090047391 | 0.07570977917981071 | 0.08290155440414503 | 0.07476635514018692 | 0.0702702702702703 |
| 0.3000000000000001 | 0.07894736842105257 | 0.18115942028985493 | 0.05294117647058818 | 0.0984455958549223 | None | 0.09090909090909094 | 0.12000000000000002 | 0.10891089108910898 | 0.14835164835164794 | 0.20879120879120908 | 0.0746887966804979 | 0.151851851851852 | 0.0931372549019608 | 0.124528301886792 | 0.150627615062762 | 0.10900473933649303 | 0.123028391167192 | 0.0880829015544041 | 0.07009345794392526 | 0.0972972972972973 |
| 0.32000000000000012 | 0.152631578947368 | 0.11594202898550703 | 0.2 | 0.11917098445595906 | None | 0.12918660287081293 | 0.10666666666666705 | 0.17821782178217807 | 0.16483516483516505 | 0.16483516483516505 | 0.132780082987552 | 0.16666666666666693 | 0.127450980392157 | 0.113207547169811 | 0.138075313807531 | 0.11848341232227498 | 0.11671924290220803 | 0.05181347150259072 | 0.04672897196261682 | 0.10270270270270303 |
| 0.34 | 0.13684210526315793 | 0.130434782608696 | 0.123529411764706 | 0.196891191709845 | None | 0.11961722488038302 | 0.09777777777777778 | 0.12376237623762404 | 0.08791208791208788 | 0.115384615384615 | 0.103734439834025 | 0.1296296296296299 | 0.10294117647058804 | 0.12075471698113205 | 0.07531380753138082 | 0.127962085308057 | 0.09779179810725552 | 0.0569948186528497 | 0.056074766355140214 | 0.11891891891891898 |
| 0.3600000000000001 | 0.12631578947368394 | 0.0434782608695652 | 0.176470588235294 | 0.14507772020725396 | None | 0.16267942583732106 | 0.0666666666666667 | 0.15346534653465307 | 0.0659340659340659 | 0.07692307692307691 | 0.10788381742738598 | 0.0777777777777778 | 0.10294117647058804 | 0.09056603773584918 | 0.10041841004184097 | 0.05687203791469193 | 0.09779179810725552 | 0.0569948186528497 | 0.051401869158878497 | 0.108108108108108 |
| 0.38000000000000012 | 0.08421052631578953 | 0.0289855072463768 | 0.09411764705882353 | 0.0984455958549223 | None | 0.105263157894737 | 0.0577777777777778 | 0.11881188118811903 | 0.0659340659340659 | 0.043956043956044015 | 0.05394190871369288 | 0.0407407407407407 | 0.12254901960784295 | 0.10943396226415103 | 0.041841004184100396 | 0.047393364928909935 | 0.06624605678233438 | 0.05181347150259072 | 0.018691588785046703 | 0.10270270270270303 |
| 0.4 | 0.07894736842105257 | 0.0144927536231884 | 0.0588235294117647 | 0.046632124352331626 | None | 0.09090909090909094 | 0.04444444444444442 | 0.10891089108910898 | 0.00549450549450549 | 0.032967032967033016 | 0.03734439834024901 | 0.04444444444444442 | 0.0735294117647059 | 0.041509433962264086 | 0.008368200836820085 | 0.042654028436018995 | 0.06624605678233438 | 0.05181347150259072 | 0.037383177570093525 | 0.05405405405405411 |
| 0.4200000000000001 | 0.0421052631578947 | 0.0144927536231884 | 0.0705882352941176 | 0.07772020725388602 | None | 0.052631578947368404 | 0.013333333333333301 | 0.03960396039603961 | 0.0 | 0.010989010989011 | 0.05394190871369288 | 0.02962962962962961 | 0.049019607843137344 | 0.0339622641509434 | 0.0167364016736402 | 0.014218009478672999 | 0.022082018927444817 | 0.0207253886010363 | 0.037383177570093525 | 0.0486486486486487 |
| 0.44 | 0.031578947368421116 | 0.0217391304347826 | 0.02352941176470589 | 0.025906735751295307 | None | 0.038277511961722514 | 0.004444444444444442 | 0.01980198019801981 | 0.00549450549450549 | 0.010989010989011 | 0.05394190871369288 | 0.0037037037037037017 | 0.0245098039215686 | 0.030188679245282988 | 0.008368200836820085 | 0.009478672985781995 | 0.022082018927444817 | 0.03626943005181352 | 0.004672897196261682 | 0.03783783783783781 |
| 0.46 | 0.031578947368421116 | 0.0144927536231884 | 0.0176470588235294 | 0.031088082901554407 | None | 0.023923444976076597 | 0.004444444444444442 | 0.014851485148514901 | 0.010989010989011 | 0.0 | 0.02904564315352701 | 0.0148148148148148 | 0.014705882352941199 | 0.011320754716981109 | 0.00418410041841004 | 0.023696682464455002 | 0.015772870662460612 | 0.0207253886010363 | 0.009345794392523367 | 0.027027027027027015 |
| 0.48000000000000009 | 0.005263157894736842 | 0.0144927536231884 | 0.029411764705882398 | 0.0 | None | 0.004784688995215312 | 0.008888888888888894 | 0.004950495049504951 | 0.00549450549450549 | 0.00549450549450549 | 0.00829875518672199 | 0.0 | 0.014705882352941199 | 0.0150943396226415 | 0.00418410041841004 | 0.018957345971564 | 0.006309148264984232 | 0.03626943005181352 | 0.009345794392523367 | 0.03783783783783781 |
| 0.5 | 0.0105263157894737 | 0.007246376811594202 | 0.0176470588235294 | 0.005181347150259074 | None | 0.019138755980861205 | 0.008888888888888894 | 0.0 | 0.0 | 0.0 | 0.00829875518672199 | 0.0037037037037037017 | 0.004901960784313736 | 0.0 | 0.0 | 0.0 | 0.0031545741324921117 | 0.010362694300518104 | 0.009345794392523367 | 0.010810810810810801 |
| 0.52 | 0.0105263157894737 | 0.0 | 0.005882352941176471 | 0.0 | None | 0.014354066985645893 | 0.004444444444444442 | 0.009900990099009906 | 0.0 | 0.00549450549450549 | 0.004149377593361002 | 0.0 | 0.00980392156862745 | 0.0037735849056603826 | 0.0 | 0.033175355450237004 | 0.012618296529968496 | 0.015544041450777204 | 0.0 | 0.010810810810810801 |
| 0.54 | 0.0 | 0.0 | 0.005882352941176471 | 0.005181347150259074 | None | 0.0 | 0.0 | 0.0 | 0.00549450549450549 | 0.0 | 0.00829875518672199 | 0.0 | 0.004901960784313736 | 0.011320754716981109 | 0.0 | 0.0 | 0.0 | 0.0 | 0.009345794392523367 | 0.0162162162162162 |
| 0.56000000000000005 | 0.0 | 0.0 | 0.005882352941176471 | 0.005181347150259074 | None | 0.0 | 0.0 | 0.0 | 0.0 | 0.0 | 0.004149377593361002 | 0.0 | 0.004901960784313736 | 0.0075471698113207504 | 0.0 | 0.0 | 0.0 | 0.005181347150259074 | 0.0 | 0.0 |
| 0.58000000000000007 | 0.0105263157894737 | 0.007246376811594202 | 0.005882352941176471 | 0.0 | None | 0.004784688995215312 | 0.0 | 0.0 | 0.0 | 0.0 | 0.0 | 0.0 | 0.004901960784313736 | 0.0 | 0.0 | 0.0 | 0.0 | 0.005181347150259074 | 0.0 | 0.0 |
| 0.6000000000000002 | 0.005263157894736842 | 0.0 | 0.0176470588235294 | 0.005181347150259074 | None | 0.0 | 0.004444444444444442 | 0.0 | 0.0 | 0.00549450549450549 | 0.0 | 0.0 | 0.004901960784313736 | 0.0 | 0.00418410041841004 | 0.004739336492891005 | 0.0 | 0.005181347150259074 | 0.0 | 0.005405405405405412 |

## Slide 3
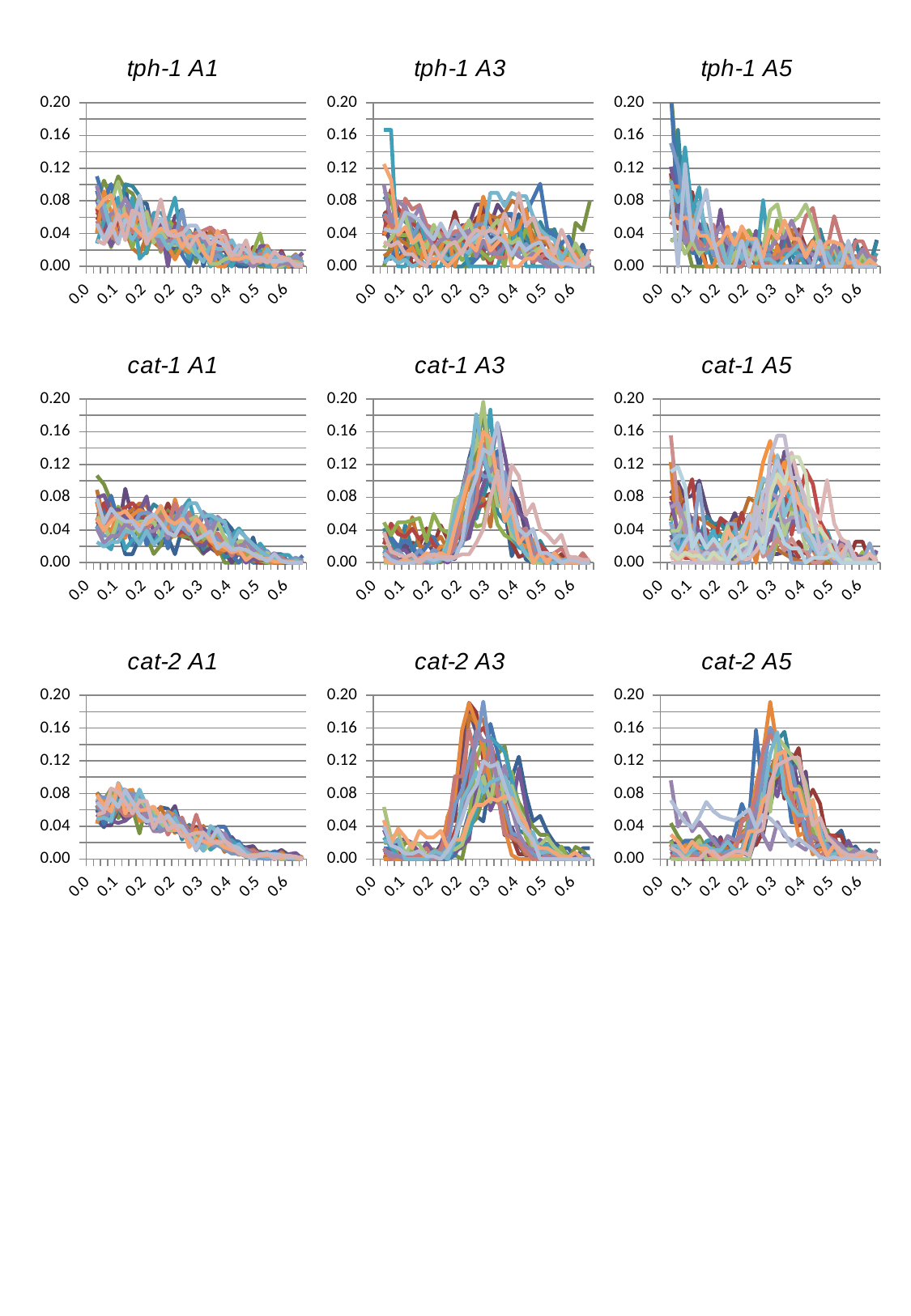

### Chart: tph-1 A1
| Category | thp-1(mg280)_A1_01 | thp-1(mg280)_A1_02 | thp-1(mg280)_A1_03 | thp-1(mg280)_A1_04 | thp-1(mg280)_A1_05 | thp-1(mg280)_A1_06 | thp-1(mg280)_A1_07 | thp-1(mg280)_A1_08 | thp-1(mg280)_A1_09 | thp-1(mg280)_A1_10 | tph-1(mg280)_A1_11 | tph-1(mg280)_A1_12 | tph-1(mg280)_A1_13 | tph-1(mg280)_A1_14 | tph-1(mg280)_A1_15 | tph-1(mg280)_A1_16 | tph-1(mg280)_A1_17 | tph-1(mg280)_A1_18 | tph-1(mg280)_A1_19 | tph-1(mg280)_A1_20 |
|---|---|---|---|---|---|---|---|---|---|---|---|---|---|---|---|---|---|---|---|---|
| 0 | None | None | None | None | None | None | None | None | None | None | None | None | None | None | None | None | None | None | None | None |
| 2.0000000000000007E-2 | 0.09230769230769234 | 0.056603773584905696 | 0.07853403141361263 | 0.08130081300813008 | 0.07361963190184054 | 0.06617647058823531 | 0.110047846889952 | 0.07106598984771573 | 0.0402298850574713 | 0.0578512396694215 | 0.028037383177570117 | 0.0416666666666667 | 0.07373271889400923 | 0.05555555555555558 | 0.09770114942528743 | 0.0987261146496815 | 0.08021390374331548 | 0.06976744186046513 | 0.051948051948052 | 0.0318181818181818 |
| 4.0000000000000015E-2 | 0.061538461538461514 | 0.07075471698113213 | 0.10471204188481703 | 0.052845528455284584 | 0.06543967280163603 | 0.05147058823529408 | 0.08133971291866028 | 0.040609137055837616 | 0.07471264367816093 | 0.0578512396694215 | 0.056074766355140214 | 0.09166666666666676 | 0.0783410138248848 | 0.0357142857142857 | 0.06896551724137931 | 0.0573248407643312 | 0.07486631016042783 | 0.08139534883720934 | 0.0324675324675325 | 0.027272727272727313 |
| 6.0000000000000019E-2 | 0.061538461538461514 | 0.042452830188679215 | 0.08376963350785344 | 0.0609756097560976 | 0.04703476482617592 | 0.0367647058823529 | 0.10047846889952194 | 0.0253807106598985 | 0.022988505747126402 | 0.024793388429752112 | 0.056074766355140214 | 0.0666666666666667 | 0.0460829493087558 | 0.039682539682539715 | 0.07758620689655173 | 0.0636942675159236 | 0.0427807486631016 | 0.0872093023255814 | 0.051948051948052 | 0.0409090909090909 |
| 8.0000000000000029E-2 | 0.06923076923076922 | 0.056603773584905696 | 0.10994764397905803 | 0.07723577235772362 | 0.0572597137014315 | 0.05147058823529408 | 0.062200956937799014 | 0.05837563451776652 | 0.0804597701149425 | 0.04132231404958682 | 0.0841121495327103 | 0.0333333333333333 | 0.06451612903225813 | 0.02777777777777782 | 0.10344827586206902 | 0.0668789808917197 | 0.0695187165775401 | 0.058139534883720916 | 0.02813852813852809 | 0.0636363636363636 |
| 0.1 | 0.1 | 0.056603773584905696 | 0.09424083769633508 | 0.0569105691056911 | 0.10020449897750502 | 0.04411764705882354 | 0.04784688995215312 | 0.06091370558375631 | 0.05747126436781611 | 0.0578512396694215 | 0.056074766355140214 | 0.058333333333333334 | 0.07373271889400923 | 0.07142857142857141 | 0.0603448275862069 | 0.0828025477707006 | 0.04812834224598928 | 0.063953488372093 | 0.05627705627705628 | 0.0318181818181818 |
| 0.12000000000000002 | 0.0384615384615385 | 0.056603773584905696 | 0.08900523560209422 | 0.04065040650406497 | 0.09815950920245403 | 0.022058823529411808 | 0.0669856459330144 | 0.05076142131979703 | 0.022988505747126402 | 0.0330578512396694 | 0.0841121495327103 | 0.0416666666666667 | 0.059907834101382514 | 0.043650793650793614 | 0.05172413793103452 | 0.0668789808917197 | 0.032085561497326213 | 0.04941860465116283 | 0.04978354978354981 | 0.0681818181818182 |
| 0.14000000000000001 | 0.07692307692307691 | 0.03773584905660382 | 0.06806282722513093 | 0.0609756097560976 | 0.0858895705521472 | 0.014705882352941199 | 0.0334928229665072 | 0.0685279187817259 | 0.05172413793103452 | 0.0330578512396694 | 0.009345794392523367 | 0.0666666666666667 | 0.07373271889400923 | 0.0357142857142857 | 0.04597701149425289 | 0.0605095541401274 | 0.04812834224598928 | 0.040697674418604717 | 0.08658008658008665 | 0.0636363636363636 |
| 0.16 | 0.07692307692307691 | 0.0471698113207547 | 0.015706806282722505 | 0.04878048780487813 | 0.05521472392638042 | 0.0367647058823529 | 0.057416267942583754 | 0.0456852791878173 | 0.0402298850574713 | 0.0661157024793388 | 0.018691588785046703 | 0.05 | 0.050691244239631325 | 0.0674603174603175 | 0.0660919540229885 | 0.035031847133758016 | 0.04812834224598928 | 0.0319767441860465 | 0.03679653679653679 | 0.027272727272727313 |
| 0.18000000000000005 | 0.0384615384615385 | 0.0330188679245283 | 0.04712041884816751 | 0.0609756097560976 | 0.05112474437627808 | 0.029411764705882398 | 0.038277511961722514 | 0.0532994923857868 | 0.04597701149425289 | 0.0330578512396694 | 0.0654205607476635 | 0.05 | 0.0460829493087558 | 0.043650793650793614 | 0.028735632183908007 | 0.0414012738853503 | 0.03743315508021393 | 0.0377906976744186 | 0.0432900432900433 | 0.0454545454545455 |
| 0.2 | 0.0461538461538462 | 0.051886792452830226 | 0.03141361256544502 | 0.04065040650406497 | 0.03680981595092022 | 0.022058823529411808 | 0.038277511961722514 | 0.0456852791878173 | 0.017241379310344803 | 0.04958677685950413 | 0.0654205607476635 | 0.0416666666666667 | 0.027649769585253527 | 0.01984126984126981 | 0.04310344827586208 | 0.02547770700636942 | 0.03743315508021393 | 0.04651162790697674 | 0.05844155844155838 | 0.08181818181818173 |
| 0.22 | 0.015384615384615405 | 0.051886792452830226 | 0.0209424083769634 | 0.032520325203252 | 0.0429447852760736 | 0.04411764705882354 | 0.028708133971291894 | 0.03807106598984771 | 0.05747126436781611 | 0.0 | 0.056074766355140214 | 0.025 | 0.050691244239631325 | 0.039682539682539715 | 0.028735632183908007 | 0.03184713375796181 | 0.021390374331550797 | 0.040697674418604717 | 0.038961038961039 | 0.0409090909090909 |
| 0.24000000000000005 | 0.02307692307692311 | 0.0330188679245283 | 0.015706806282722505 | 0.0609756097560976 | 0.0429447852760736 | 0.04411764705882354 | 0.0334928229665072 | 0.0532994923857868 | 0.04597701149425289 | 0.04958677685950413 | 0.0841121495327103 | 0.00833333333333333 | 0.03225806451612901 | 0.0317460317460317 | 0.0258620689655172 | 0.0286624203821656 | 0.032085561497326213 | 0.0436046511627907 | 0.03679653679653679 | 0.0363636363636364 |
| 0.26 | 0.0384615384615385 | 0.042452830188679215 | 0.03141361256544502 | 0.06910569105691064 | 0.0286298568507157 | 0.014705882352941199 | 0.014354066985645893 | 0.0355329949238579 | 0.0402298850574713 | 0.04132231404958682 | 0.028037383177570117 | 0.025 | 0.06912442396313365 | 0.047619047619047616 | 0.028735632183908007 | 0.03184713375796181 | 0.021390374331550797 | 0.0232558139534884 | 0.038961038961039 | 0.0409090909090909 |
| 0.28000000000000008 | 0.007692307692307692 | 0.042452830188679215 | 0.0209424083769634 | 0.020325203252032485 | 0.02453987730061352 | 0.04411764705882354 | 0.0 | 0.0355329949238579 | 0.0402298850574713 | 0.0330578512396694 | 0.009345794392523367 | 0.0166666666666667 | 0.0230414746543779 | 0.0357142857142857 | 0.022988505747126402 | 0.035031847133758016 | 0.0427807486631016 | 0.0377906976744186 | 0.04978354978354981 | 0.022727272727272717 |
| 0.3000000000000001 | 0.0461538461538462 | 0.00471698113207547 | 0.005235602094240845 | 0.032520325203252 | 0.012269938650306698 | 0.029411764705882398 | 0.028708133971291894 | 0.040609137055837616 | 0.028735632183908007 | 0.04132231404958682 | 0.037383177570093525 | 0.0416666666666667 | 0.027649769585253527 | 0.039682539682539715 | 0.014367816091953999 | 0.03184713375796181 | 0.016042780748663114 | 0.0348837209302326 | 0.04978354978354981 | 0.0363636363636364 |
| 0.32000000000000012 | 0.0 | 0.042452830188679215 | 0.026178010471204216 | 0.00813008130081301 | 0.020449897750511214 | 0.022058823529411808 | 0.009569377990430622 | 0.040609137055837616 | 0.028735632183908007 | 0.016528925619834708 | 0.028037383177570117 | 0.0333333333333333 | 0.018433179723502308 | 0.043650793650793614 | 0.0258620689655172 | 0.035031847133758016 | 0.0053475935828877 | 0.017441860465116307 | 0.0346320346320346 | 0.0409090909090909 |
| 0.34 | 0.030769230769230802 | 0.03773584905660382 | 0.0 | 0.0284552845528455 | 0.0143149284253579 | 0.04411764705882354 | 0.019138755980861205 | 0.015228426395939104 | 0.0 | 0.016528925619834708 | 0.0 | 0.025 | 0.00460829493087558 | 0.047619047619047616 | 0.0028735632183908015 | 0.015923566878980902 | 0.026737967914438505 | 0.005813953488372092 | 0.03679653679653679 | 0.027272727272727313 |
| 0.3600000000000001 | 0.015384615384615405 | 0.0330188679245283 | 0.010471204188481699 | 0.016260162601626 | 0.0061349693251533735 | 0.022058823529411808 | 0.014354066985645893 | 0.0228426395939086 | 0.017241379310344803 | 0.024793388429752112 | 0.018691588785046703 | 0.0 | 0.013824884792626705 | 0.039682539682539715 | 0.0028735632183908015 | 0.019108280254777107 | 0.021390374331550797 | 0.0436046511627907 | 0.0303030303030303 | 0.0363636363636364 |
| 0.38000000000000012 | 0.007692307692307692 | 0.0330188679245283 | 0.005235602094240845 | 0.020325203252032485 | 0.012269938650306698 | 0.0 | 0.0 | 0.0304568527918782 | 0.022988505747126402 | 0.0330578512396694 | 0.009345794392523367 | 0.0 | 0.00460829493087558 | 0.043650793650793614 | 0.008620689655172415 | 0.02229299363057321 | 0.021390374331550797 | 0.0232558139534884 | 0.025974025974026007 | 0.0363636363636364 |
| 0.4 | 0.007692307692307692 | 0.023584905660377402 | 0.010471204188481699 | 0.0 | 0.0081799591002045 | 0.014705882352941199 | 0.004784688995215312 | 0.027918781725888294 | 0.0114942528735632 | 0.016528925619834708 | 0.0 | 0.0166666666666667 | 0.00921658986175115 | 0.01984126984126981 | 0.0114942528735632 | 0.019108280254777107 | 0.032085561497326213 | 0.008720930232558145 | 0.012987012987013 | 0.013636363636363601 |
| 0.4200000000000001 | 0.0 | 0.00943396226415094 | 0.010471204188481699 | 0.0121951219512195 | 0.00408997955010225 | 0.022058823529411808 | 0.004784688995215312 | 0.0177664974619289 | 0.022988505747126402 | 0.024793388429752112 | 0.018691588785046703 | 0.00833333333333333 | 0.013824884792626705 | 0.023809523809523808 | 0.017241379310344803 | 0.006369426751592362 | 0.016042780748663114 | 0.008720930232558145 | 0.025974025974026007 | 0.013636363636363601 |
| 0.44 | 0.0 | 0.0 | 0.005235602094240845 | 0.0 | 0.002044989775051121 | 0.022058823529411808 | 0.004784688995215312 | 0.007614213197969542 | 0.005747126436781612 | 0.024793388429752112 | 0.018691588785046703 | 0.00833333333333333 | 0.00921658986175115 | 0.007936507936507941 | 0.005747126436781612 | 0.006369426751592362 | 0.0053475935828877 | 0.011627906976744195 | 0.019480519480519508 | 0.0318181818181818 |
| 0.46 | 0.007692307692307692 | 0.023584905660377402 | 0.005235602094240845 | 0.0121951219512195 | 0.0081799591002045 | 0.0 | 0.014354066985645893 | 0.005076142131979704 | 0.017241379310344803 | 0.0 | 0.0 | 0.0166666666666667 | 0.00460829493087558 | 0.0039682539682539715 | 0.0028735632183908015 | 0.0 | 0.016042780748663114 | 0.008720930232558145 | 0.010822510822510801 | 0.0045454545454545504 |
| 0.48000000000000009 | 0.0 | 0.014150943396226398 | 0.0 | 0.0040650406504065 | 0.002044989775051121 | 0.00735294117647059 | 0.004784688995215312 | 0.015228426395939104 | 0.0402298850574713 | 0.024793388429752112 | 0.018691588785046703 | 0.00833333333333333 | 0.00460829493087558 | 0.0158730158730159 | 0.005747126436781612 | 0.006369426751592362 | 0.0053475935828877 | 0.005813953488372092 | 0.010822510822510801 | 0.0090909090909091 |
| 0.5 | 0.0 | 0.0188679245283019 | 0.0 | 0.0040650406504065 | 0.0061349693251533735 | 0.022058823529411808 | 0.0 | 0.007614213197969542 | 0.0 | 0.024793388429752112 | 0.0 | 0.025 | 0.00921658986175115 | 0.007936507936507941 | 0.0114942528735632 | 0.00955414012738853 | 0.021390374331550797 | 0.0145348837209302 | 0.006493506493506492 | 0.0045454545454545504 |
| 0.52 | 0.0 | 0.00471698113207547 | 0.0 | 0.0121951219512195 | 0.002044989775051121 | 0.00735294117647059 | 0.0 | 0.002538071065989851 | 0.005747126436781612 | 0.008264462809917364 | 0.0 | 0.0 | 0.0 | 0.007936507936507941 | 0.0 | 0.0 | 0.0106951871657754 | 0.0 | 0.006493506493506492 | 0.018181818181818205 |
| 0.54 | 0.0 | 0.0188679245283019 | 0.0 | 0.0 | 0.0 | 0.00735294117647059 | 0.0 | 0.002538071065989851 | 0.005747126436781612 | 0.016528925619834708 | 0.0 | 0.00833333333333333 | 0.00460829493087558 | 0.007936507936507941 | 0.0 | 0.0031847133757961815 | 0.0053475935828877 | 0.011627906976744195 | 0.006493506493506492 | 0.0090909090909091 |
| 0.56000000000000005 | 0.0 | 0.0 | 0.005235602094240845 | 0.0 | 0.002044989775051121 | 0.00735294117647059 | 0.004784688995215312 | 0.0 | 0.0114942528735632 | 0.0 | 0.0 | 0.0 | 0.00460829493087558 | 0.0039682539682539715 | 0.005747126436781612 | 0.0031847133757961815 | 0.0106951871657754 | 0.008720930232558145 | 0.008658008658008665 | 0.0090909090909091 |
| 0.58000000000000007 | 0.0 | 0.0 | 0.0 | 0.0 | 0.002044989775051121 | 0.014705882352941199 | 0.0 | 0.005076142131979704 | 0.005747126436781612 | 0.008264462809917364 | 0.009345794392523367 | 0.00833333333333333 | 0.013824884792626705 | 0.007936507936507941 | 0.005747126436781612 | 0.0 | 0.0 | 0.0029069767441860512 | 0.002164502164502161 | 0.0 |
| 0.6000000000000002 | 0.0 | 0.0 | 0.0 | 0.0040650406504065 | 0.0 | 0.0 | 0.0 | 0.002538071065989851 | 0.0 | 0.016528925619834708 | 0.009345794392523367 | 0.00833333333333333 | 0.00460829493087558 | 0.0 | 0.0028735632183908015 | 0.0031847133757961815 | 0.0053475935828877 | 0.0029069767441860512 | 0.002164502164502161 | 0.0 |
### Chart: tph-1 A3
| Category | tph-1(mg280)_A3_01 | tph-1(mg280)_A3_02 | tph-1(mg280)_A3_03 | tph-1(mg280)_A3_04 | tph-1(mg280)_A3_05 | tph-1(mg280)_A3_06 | tph-1(mg280)_A3_07 | tph-1(mg280)_A3_08 | tph-1(mg280)_A3_09 | tph-1(mg280)_A3_10 | tph-1(mg280)_A3_11 | tph-1(mg280)_A3_12 | tph-1(mg280)_A3_13 | tph-1(mg280)_A3_14 | tph-1(mg280)_A3_15 | tph-1(mg280)_A3_16 | tph-1(mg280)_A3_17 | tph-1(mg280)_A3_18 | tph-1(mg280)_A3_19 | tph-1(mg280)_A3_20 |
|---|---|---|---|---|---|---|---|---|---|---|---|---|---|---|---|---|---|---|---|---|
| 0 | None | None | None | None | None | None | None | None | None | None | None | None | None | None | None | None | None | None | None | None |
| 2.0000000000000007E-2 | 0.06382978723404252 | 0.0666666666666667 | 0.0 | 0.0440251572327044 | 0.08181818181818173 | 0.012383900928792598 | 0.00917431192660551 | 0.037558685446009404 | 0.09160305343511449 | None | 0.16666666666666693 | 0.0387596899224806 | 0.0636363636363636 | 0.06149732620320863 | 0.0225988700564972 | 0.09960159362549803 | 0.004081632653061222 | 0.125 | 0.04605263157894742 | 0.0298507462686567 |
| 4.0000000000000015E-2 | 0.0212765957446809 | 0.058333333333333334 | 0.02654867256637171 | 0.03144654088050313 | 0.09090909090909094 | 0.018575851393188913 | 0.00917431192660551 | 0.09389671361502352 | 0.08396946564885506 | None | 0.16666666666666693 | 0.0542635658914729 | 0.05757575757575758 | 0.05080213903743323 | 0.03389830508474581 | 0.055776892430278904 | 0.012244897959183699 | 0.10576923076923106 | 0.042763157894736836 | 0.0248756218905473 |
| 6.0000000000000019E-2 | 0.047872340425531915 | 0.0333333333333333 | 0.008849557522123897 | 0.006289308176100634 | 0.0636363636363636 | 0.03405572755417961 | 0.027522935779816515 | 0.0704225352112676 | 0.03816793893129768 | None | 0.0 | 0.007751937984496123 | 0.048484848484848485 | 0.03743315508021393 | 0.05084745762711862 | 0.07968127490039843 | 0.004081632653061222 | 0.0384615384615385 | 0.042763157894736836 | 0.0298507462686567 |
| 8.0000000000000029E-2 | 0.031914893617021316 | 0.0416666666666667 | 0.008849557522123897 | 0.02515723270440251 | 0.05454545454545449 | 0.027863777089783322 | 0.00917431192660551 | 0.06103286384976532 | 0.03816793893129768 | None | 0.0 | 0.015503875968992208 | 0.0757575757575758 | 0.08288770053475938 | 0.0621468926553672 | 0.0717131474103586 | 0.00816326530612245 | 0.048076923076923114 | 0.06578947368421051 | 0.014925373134328401 |
| 0.1 | 0.010638297872340394 | 0.0 | 0.0619469026548673 | 0.012578616352201295 | 0.0454545454545455 | 0.02167182662538701 | 0.018348623853211 | 0.051643192488262865 | 0.03816793893129768 | None | 0.02777777777777782 | 0.0232558139534884 | 0.0363636363636364 | 0.0695187165775401 | 0.02824858757062151 | 0.055776892430278904 | 0.0 | 0.0288461538461538 | 0.0625 | 0.014925373134328401 |
| 0.12000000000000002 | 0.026595744680851106 | 0.00833333333333333 | 0.0353982300884956 | 0.03773584905660382 | 0.018181818181818205 | 0.012383900928792598 | 0.00917431192660551 | 0.0469483568075117 | 0.015267175572519104 | None | 0.05555555555555558 | 0.0 | 0.051515151515151486 | 0.07486631016042783 | 0.03389830508474581 | 0.0717131474103586 | 0.004081632653061222 | 0.0384615384615385 | 0.055921052631578885 | 0.004975124378109448 |
| 0.14000000000000001 | 0.031914893617021316 | 0.0166666666666667 | 0.008849557522123897 | 0.03144654088050313 | 0.0090909090909091 | 0.024767801857585092 | 0.0 | 0.03286384976525821 | 0.03053435114503819 | None | 0.02777777777777782 | 0.031007751937984492 | 0.048484848484848485 | 0.05080213903743323 | 0.0225988700564972 | 0.039840637450199216 | 0.004081632653061222 | 0.009615384615384628 | 0.042763157894736836 | 0.0 |
| 0.16 | 0.031914893617021316 | 0.0333333333333333 | 0.0176991150442478 | 0.0440251572327044 | 0.018181818181818205 | 0.0154798761609907 | 0.0 | 0.028169014084506998 | 0.0229007633587786 | None | 0.0 | 0.0 | 0.0393939393939394 | 0.0454545454545455 | 0.05084745762711862 | 0.023904382470119518 | 0.00816326530612245 | 0.009615384615384628 | 0.03289473684210532 | 0.0248756218905473 |
| 0.18000000000000005 | 0.010638297872340394 | 0.0166666666666667 | 0.02654867256637171 | 0.03144654088050313 | 0.018181818181818205 | 0.0154798761609907 | 0.018348623853211 | 0.0469483568075117 | 0.015267175572519104 | None | 0.0 | 0.0232558139534884 | 0.0363636363636364 | 0.0401069518716578 | 0.03389830508474581 | 0.03187250996015941 | 0.016326530612244903 | 0.009615384615384628 | 0.052631578947368404 | 0.009950248756218917 |
| 0.2 | 0.010638297872340394 | 0.0333333333333333 | 0.008849557522123897 | 0.02515723270440251 | 0.0090909090909091 | 0.02167182662538701 | 0.0 | 0.028169014084506998 | 0.0229007633587786 | None | 0.02777777777777782 | 0.0387596899224806 | 0.0393939393939394 | 0.034759358288770116 | 0.02824858757062151 | 0.03585657370517932 | 0.00816326530612245 | 0.0 | 0.03618421052631582 | 0.0248756218905473 |
| 0.22 | 0.042553191489361715 | 0.0666666666666667 | 0.0 | 0.03144654088050313 | 0.0 | 0.027863777089783322 | 0.018348623853211 | 0.0187793427230047 | 0.0229007633587786 | None | 0.0 | 0.0 | 0.0393939393939394 | 0.0427807486631016 | 0.02824858757062151 | 0.03187250996015941 | 0.012244897959183699 | 0.009615384615384628 | 0.055921052631578885 | 0.0298507462686567 |
| 0.24000000000000005 | 0.026595744680851106 | 0.0333333333333333 | 0.0 | 0.050314465408805 | 0.0 | 0.0309597523219814 | 0.027522935779816515 | 0.037558685446009404 | 0.007633587786259542 | None | 0.02777777777777782 | 0.04651162790697674 | 0.0363636363636364 | 0.0427807486631016 | 0.03389830508474581 | 0.039840637450199216 | 0.020408163265306107 | 0.019230769230769208 | 0.042763157894736836 | 0.014925373134328401 |
| 0.26 | 0.03723404255319152 | 0.0416666666666667 | 0.0 | 0.050314465408805 | 0.0090909090909091 | 0.040247678018575886 | 0.0 | 0.0187793427230047 | 0.015267175572519104 | None | 0.0 | 0.0542635658914729 | 0.0333333333333333 | 0.034759358288770116 | 0.0564971751412429 | 0.0438247011952191 | 0.016326530612244903 | 0.0288461538461538 | 0.029605263157894714 | 0.03482587064676621 |
| 0.28000000000000008 | 0.026595744680851106 | 0.058333333333333334 | 0.02654867256637171 | 0.0754716981132075 | 0.0090909090909091 | 0.03405572755417961 | 0.00917431192660551 | 0.028169014084506998 | 0.0229007633587786 | None | 0.0 | 0.04651162790697674 | 0.051515151515151486 | 0.029411764705882398 | 0.02824858757062151 | 0.011952191235059804 | 0.04081632653061222 | 0.019230769230769208 | 0.03618421052631582 | 0.0447761194029851 |
| 0.3000000000000001 | 0.06914893617021281 | 0.0666666666666667 | 0.008849557522123897 | 0.0754716981132075 | 0.027272727272727313 | 0.0619195046439628 | 0.018348623853211 | 0.0187793427230047 | 0.015267175572519104 | None | 0.0 | 0.0852713178294574 | 0.051515151515151486 | 0.0454545454545455 | 0.039548022598870115 | 0.03187250996015941 | 0.03673469387755101 | 0.048076923076923114 | 0.042763157894736836 | 0.03980099502487562 |
| 0.32000000000000012 | 0.026595744680851106 | 0.058333333333333334 | 0.0176991150442478 | 0.050314465408805 | 0.018181818181818205 | 0.0619195046439628 | 0.027522935779816515 | 0.0 | 0.007633587786259542 | None | 0.0 | 0.0542635658914729 | 0.0424242424242424 | 0.026737967914438505 | 0.0451977401129944 | 0.0159362549800797 | 0.08979591836734697 | 0.019230769230769208 | 0.029605263157894714 | 0.0447761194029851 |
| 0.34 | 0.058510638297872314 | 0.05 | 0.0353982300884956 | 0.0754716981132075 | 0.018181818181818205 | 0.0588235294117647 | 0.018348623853211 | 0.03286384976525821 | 0.015267175572519104 | None | 0.0 | 0.0387596899224806 | 0.0363636363636364 | 0.0106951871657754 | 0.0564971751412429 | 0.0159362549800797 | 0.08979591836734697 | 0.0288461538461538 | 0.03618421052631582 | 0.03980099502487562 |
| 0.3600000000000001 | 0.026595744680851106 | 0.00833333333333333 | 0.02654867256637171 | 0.06289308176100633 | 0.027272727272727313 | 0.06501547987616103 | 0.0642201834862385 | 0.0187793427230047 | 0.0 | None | 0.02777777777777782 | 0.0542635658914729 | 0.0303030303030303 | 0.0106951871657754 | 0.03389830508474581 | 0.023904382470119518 | 0.07346938775510202 | 0.0384615384615385 | 0.029605263157894714 | 0.06467661691542292 |
| 0.38000000000000012 | 0.031914893617021316 | 0.0333333333333333 | 0.008849557522123897 | 0.06289308176100633 | 0.027272727272727313 | 0.0804953560371517 | 0.0642201834862385 | 0.02347417840375591 | 0.053435114503816813 | None | 0.02777777777777782 | 0.0387596899224806 | 0.0212121212121212 | 0.018716577540107006 | 0.02824858757062151 | 0.0199203187250996 | 0.08979591836734697 | 0.0 | 0.013157894736842101 | 0.0447761194029851 |
| 0.4 | 0.06382978723404252 | 0.025 | 0.0353982300884956 | 0.02515723270440251 | 0.0454545454545455 | 0.0743034055727554 | 0.03669724770642203 | 0.028169014084506998 | 0.0229007633587786 | None | 0.05555555555555558 | 0.04651162790697674 | 0.015151515151515204 | 0.021390374331550797 | 0.03389830508474581 | 0.011952191235059804 | 0.0857142857142857 | 0.0 | 0.029605263157894714 | 0.08955223880597013 |
| 0.4200000000000001 | 0.0212765957446809 | 0.0166666666666667 | 0.0353982300884956 | 0.012578616352201295 | 0.0454545454545455 | 0.0588235294117647 | 0.0642201834862385 | 0.02347417840375591 | 0.0458015267175573 | None | 0.0 | 0.06976744186046513 | 0.027272727272727313 | 0.021390374331550797 | 0.0112994350282486 | 0.007968127490039844 | 0.0857142857142857 | 0.009615384615384628 | 0.019736842105263205 | 0.049751243781094495 |
| 0.44 | 0.031914893617021316 | 0.025 | 0.053097345132743404 | 0.0188679245283019 | 0.0 | 0.03715170278637772 | 0.08256880733944956 | 0.02347417840375591 | 0.03053435114503819 | None | 0.0 | 0.0232558139534884 | 0.018181818181818205 | 0.0053475935828877 | 0.016949152542372906 | 0.0159362549800797 | 0.0612244897959184 | 0.009615384615384628 | 0.026315789473684202 | 0.05970149253731343 |
| 0.46 | 0.0159574468085106 | 0.0166666666666667 | 0.02654867256637171 | 0.0188679245283019 | 0.05454545454545449 | 0.0309597523219814 | 0.10091743119266094 | 0.02347417840375591 | 0.007633587786259542 | None | 0.0 | 0.0387596899224806 | 0.00606060606060606 | 0.0106951871657754 | 0.0225988700564972 | 0.007968127490039844 | 0.044897959183673515 | 0.019230769230769208 | 0.029605263157894714 | 0.03482587064676621 |
| 0.48000000000000009 | 0.0159574468085106 | 0.00833333333333333 | 0.044247787610619496 | 0.03144654088050313 | 0.0363636363636364 | 0.027863777089783322 | 0.045871559633027484 | 0.0 | 0.0 | None | 0.0 | 0.031007751937984492 | 0.00606060606060606 | 0.016042780748663114 | 0.016949152542372906 | 0.0 | 0.03673469387755101 | 0.019230769230769208 | 0.013157894736842101 | 0.03482587064676621 |
| 0.5 | 0.00531914893617021 | 0.0166666666666667 | 0.044247787610619496 | 0.006289308176100634 | 0.0454545454545455 | 0.012383900928792598 | 0.03669724770642203 | 0.009389671361502353 | 0.007633587786259542 | None | 0.0 | 0.0232558139534884 | 0.0 | 0.0106951871657754 | 0.0225988700564972 | 0.0 | 0.032653061224489806 | 0.009615384615384628 | 0.006578947368421051 | 0.014925373134328401 |
| 0.52 | 0.00531914893617021 | 0.0 | 0.02654867256637171 | 0.006289308176100634 | 0.018181818181818205 | 0.00309597523219814 | 0.027522935779816515 | 0.0187793427230047 | 0.015267175572519104 | None | 0.0 | 0.015503875968992208 | 0.0090909090909091 | 0.00802139037433155 | 0.00564971751412429 | 0.0 | 0.00816326530612245 | 0.0 | 0.003289473684210531 | 0.0447761194029851 |
| 0.54 | 0.0159574468085106 | 0.025 | 0.0 | 0.006289308176100634 | 0.0090909090909091 | 0.0061919504643962895 | 0.03669724770642203 | 0.014084507042253504 | 0.015267175572519104 | None | 0.0 | 0.007751937984496123 | 0.0 | 0.0053475935828877 | 0.00564971751412429 | 0.003984063745019922 | 0.024489795918367314 | 0.009615384615384628 | 0.003289473684210531 | 0.0248756218905473 |
| 0.56000000000000005 | 0.010638297872340394 | 0.0 | 0.053097345132743404 | 0.006289308176100634 | 0.0090909090909091 | 0.00309597523219814 | 0.018348623853211 | 0.0 | 0.0229007633587786 | None | 0.0 | 0.015503875968992208 | 0.00303030303030303 | 0.0053475935828877 | 0.02824858757062151 | 0.0 | 0.004081632653061222 | 0.0 | 0.0 | 0.009950248756218917 |
| 0.58000000000000007 | 0.026595744680851106 | 0.0 | 0.044247787610619496 | 0.006289308176100634 | 0.0 | 0.009287925696594427 | 0.018348623853211 | 0.009389671361502353 | 0.0 | None | 0.0 | 0.0 | 0.012121212121212099 | 0.00802139037433155 | 0.0112994350282486 | 0.003984063745019922 | 0.004081632653061222 | 0.009615384615384628 | 0.0 | 0.0 |
| 0.6000000000000002 | 0.0 | 0.0 | 0.079646017699115 | 0.0 | 0.0090909090909091 | 0.0061919504643962895 | 0.018348623853211 | 0.00469483568075117 | 0.0 | None | 0.0 | 0.007751937984496123 | 0.00303030303030303 | 0.002673796791443851 | 0.00564971751412429 | 0.0 | 0.00816326530612245 | 0.009615384615384628 | 0.006578947368421051 | 0.01990049751243781 |
### Chart: tph-1 A5
| Category | tph-1(mg280)_A5_01 | tph-1(mg280)_A5_02 | tph-1(mg280)_A5_03 | tph-1(mg280)_A5_04 | tph-1(mg280)_A5_05 | tph-1(mg280)_A5_06 | tph-1(mg280)_A5_07 | tph-1(mg280)_A5_08 | tph-1(mg280)_A5_10 | tph-1(mg280)_A5_11 | tph-1(mg280)_A5_12 | tph-1(mg280)_A5_13 | tph-1(mg280)_A5_14 | tph-1(mg280)_A5_15 | tph-1(mg280)_A5_16 | tph-1(mg280)_A5_17 | tph-1(mg280)_A5_18 | tph-1(mg280)_A5_19 | tph-1(mg280)_A5_20 |
|---|---|---|---|---|---|---|---|---|---|---|---|---|---|---|---|---|---|---|---|
| 0 | None | None | None | None | None | None | None | None | None | None | None | None | None | None | None | None | None | None | None |
| 2.0000000000000007E-2 | 0.03225806451612901 | 0.11363636363636398 | 0.214285714285714 | None | 0.0606060606060606 | 0.057692307692307716 | 0.21311475409836106 | None | 0.106918238993711 | 0.12173913043478303 | 0.08064516129032265 | 0.09756097560975616 | 0.15068493150684906 | 0.051020408163265286 | 0.0303030303030303 | 0.0629370629370629 | 0.104347826086957 | 0.06367041198501873 | 0.09375000000000004 |
| 4.0000000000000015E-2 | 0.03225806451612901 | 0.0454545454545455 | 0.107142857142857 | None | 0.16666666666666693 | 0.09615384615384627 | 0.06557377049180331 | None | 0.0817610062893082 | 0.0782608695652174 | 0.08064516129032265 | 0.09756097560975616 | 0.12328767123287702 | 0.0612244897959184 | 0.037878787878787915 | 0.0629370629370629 | 0.0782608695652174 | 0.05243445692883903 | 0.0 |
| 6.0000000000000019E-2 | 0.03225806451612901 | 0.09090909090909094 | 0.0357142857142857 | None | 0.0454545454545455 | 0.044871794871794914 | 0.0491803278688525 | None | 0.050314465408805 | 0.05217391304347832 | 0.145161290322581 | 0.09756097560975616 | 0.0684931506849315 | 0.04081632653061222 | 0.015151515151515204 | 0.02797202797202801 | 0.09565217391304354 | 0.0599250936329588 | 0.125 |
| 8.0000000000000029E-2 | 0.03225806451612901 | 0.09090909090909094 | 0.0 | None | 0.0303030303030303 | 0.06410256410256411 | 0.0163934426229508 | None | 0.03144654088050313 | 0.0347826086956522 | 0.06451612903225813 | 0.04878048780487813 | 0.027397260273972608 | 0.020408163265306107 | 0.0303030303030303 | 0.041958041958042015 | 0.0434782608695652 | 0.056179775280898875 | 0.03125 |
| 0.1 | 0.0 | 0.0454545454545455 | 0.0 | None | 0.0454545454545455 | 0.0384615384615385 | 0.0163934426229508 | None | 0.0440251572327044 | 0.05217391304347832 | 0.09677419354838712 | 0.024390243902439 | 0.041095890410958895 | 0.030612244897959207 | 0.015151515151515204 | 0.020979020979021008 | 0.0782608695652174 | 0.03745318352059932 | 0.0625 |
| 0.12000000000000002 | 0.03225806451612901 | 0.0 | 0.0 | None | 0.0303030303030303 | 0.0512820512820513 | 0.0491803278688525 | None | 0.0440251572327044 | 0.026086956521739115 | 0.016129032258064505 | 0.0 | 0.041095890410958895 | 0.020408163265306107 | 0.022727272727272717 | 0.020979020979021008 | 0.0434782608695652 | 0.03745318352059932 | 0.09375000000000004 |
| 0.14000000000000001 | 0.03225806451612901 | 0.0 | 0.0 | None | 0.0 | 0.012820512820512801 | 0.0163934426229508 | None | 0.03144654088050313 | 0.0173913043478261 | 0.03225806451612901 | 0.0 | 0.0136986301369863 | 0.051020408163265286 | 0.022727272727272717 | 0.034965034965034995 | 0.026086956521739115 | 0.022471910112359623 | 0.03125 |
| 0.16 | 0.0 | 0.022727272727272717 | 0.0 | None | 0.0303030303030303 | 0.019230769230769208 | 0.0163934426229508 | None | 0.03144654088050313 | 0.06956521739130432 | 0.03225806451612901 | 0.024390243902439 | 0.0136986301369863 | 0.010204081632653106 | 0.0303030303030303 | 0.048951048951049 | 0.026086956521739115 | 0.0299625468164794 | 0.0 |
| 0.18000000000000005 | 0.0 | 0.022727272727272717 | 0.0 | None | 0.015151515151515204 | 0.012820512820512801 | 0.0327868852459016 | None | 0.012578616352201295 | 0.0173913043478261 | 0.0 | 0.04878048780487813 | 0.0136986301369863 | 0.0 | 0.0 | 0.02797202797202801 | 0.0 | 0.0449438202247191 | 0.0 |
| 0.2 | 0.0 | 0.0 | 0.0 | None | 0.0 | 0.02564102564102561 | 0.0163934426229508 | None | 0.03144654088050313 | 0.0173913043478261 | 0.03225806451612901 | 0.0 | 0.041095890410958895 | 0.0 | 0.015151515151515204 | 0.02797202797202801 | 0.0347826086956522 | 0.02621722846441952 | 0.0 |
| 0.22 | 0.0 | 0.0 | 0.0357142857142857 | None | 0.015151515151515204 | 0.019230769230769208 | 0.0 | None | 0.03773584905660382 | 0.026086956521739115 | 0.016129032258064505 | 0.04878048780487813 | 0.0136986301369863 | 0.0 | 0.0303030303030303 | 0.013986013986014 | 0.008695652173913045 | 0.048689138576778965 | 0.03125 |
| 0.24000000000000005 | 0.03225806451612901 | 0.022727272727272717 | 0.0 | None | 0.0303030303030303 | 0.012820512820512801 | 0.0163934426229508 | None | 0.0440251572327044 | 0.008695652173913045 | 0.0 | 0.0 | 0.0136986301369863 | 0.020408163265306107 | 0.015151515151515204 | 0.034965034965034995 | 0.0347826086956522 | 0.03370786516853931 | 0.0 |
| 0.26 | 0.0 | 0.0 | 0.0 | None | 0.015151515151515204 | 0.006410256410256412 | 0.0163934426229508 | None | 0.02515723270440251 | 0.0434782608695652 | 0.0 | 0.0 | 0.027397260273972608 | 0.010204081632653106 | 0.007575757575757582 | 0.020979020979021008 | 0.026086956521739115 | 0.03370786516853931 | 0.03125 |
| 0.28000000000000008 | 0.0 | 0.022727272727272717 | 0.0 | None | 0.0 | 0.012820512820512801 | 0.0 | None | 0.02515723270440251 | 0.0173913043478261 | 0.08064516129032265 | 0.0 | 0.0136986301369863 | 0.0 | 0.022727272727272717 | 0.020979020979021008 | 0.008695652173913045 | 0.0112359550561798 | 0.0 |
| 0.3000000000000001 | 0.0 | 0.0 | 0.0 | None | 0.015151515151515204 | 0.0384615384615385 | 0.0 | None | 0.012578616352201295 | 0.0173913043478261 | 0.0 | 0.0 | 0.0136986301369863 | 0.010204081632653106 | 0.0681818181818182 | 0.020979020979021008 | 0.008695652173913045 | 0.0449438202247191 | 0.0 |
| 0.32000000000000012 | 0.0 | 0.0 | 0.0 | None | 0.0 | 0.02564102564102561 | 0.0 | None | 0.056603773584905696 | 0.008695652173913045 | 0.016129032258064505 | 0.024390243902439 | 0.0 | 0.010204081632653106 | 0.0757575757575758 | 0.034965034965034995 | 0.0 | 0.03370786516853931 | 0.0 |
| 0.34 | 0.03225806451612901 | 0.022727272727272717 | 0.0 | None | 0.0303030303030303 | 0.012820512820512801 | 0.0 | None | 0.03773584905660382 | 0.0434782608695652 | 0.016129032258064505 | 0.0 | 0.0 | 0.020408163265306107 | 0.037878787878787915 | 0.034965034965034995 | 0.008695652173913045 | 0.056179775280898875 | 0.0 |
| 0.3600000000000001 | 0.0 | 0.022727272727272717 | 0.0 | None | 0.015151515151515204 | 0.044871794871794914 | 0.0 | None | 0.02515723270440251 | 0.0173913043478261 | 0.016129032258064505 | 0.0 | 0.0 | 0.020408163265306107 | 0.053030303030303 | 0.055944055944055895 | 0.0173913043478261 | 0.03370786516853931 | 0.0 |
| 0.38000000000000012 | 0.0 | 0.0454545454545455 | 0.0 | None | 0.0 | 0.006410256410256412 | 0.0 | None | 0.012578616352201295 | 0.026086956521739115 | 0.016129032258064505 | 0.0 | 0.0136986301369863 | 0.030612244897959207 | 0.0606060606060606 | 0.034965034965034995 | 0.026086956521739115 | 0.03370786516853931 | 0.0 |
| 0.4 | 0.0 | 0.022727272727272717 | 0.0 | None | 0.0 | 0.019230769230769208 | 0.0 | None | 0.012578616352201295 | 0.0 | 0.0 | 0.0 | 0.0136986301369863 | 0.0612244897959184 | 0.0757575757575758 | 0.020979020979021008 | 0.0173913043478261 | 0.0112359550561798 | 0.0 |
| 0.4200000000000001 | 0.0 | 0.0 | 0.0 | None | 0.0303030303030303 | 0.032051282051282 | 0.0 | None | 0.0188679245283019 | 0.0173913043478261 | 0.0 | 0.0 | 0.0 | 0.07142857142857141 | 0.053030303030303 | 0.013986013986014 | 0.026086956521739115 | 0.0299625468164794 | 0.0 |
| 0.44 | 0.0 | 0.0 | 0.0 | None | 0.0454545454545455 | 0.012820512820512801 | 0.0327868852459016 | None | 0.0188679245283019 | 0.008695652173913045 | 0.016129032258064505 | 0.0 | 0.0 | 0.030612244897959207 | 0.022727272727272717 | 0.013986013986014 | 0.0173913043478261 | 0.0149812734082397 | 0.03125 |
| 0.46 | 0.0 | 0.0 | 0.0 | None | 0.015151515151515204 | 0.019230769230769208 | 0.0 | None | 0.0188679245283019 | 0.0 | 0.0 | 0.0 | 0.0136986301369863 | 0.020408163265306107 | 0.022727272727272717 | 0.006993006993006992 | 0.0173913043478261 | 0.0299625468164794 | 0.0 |
| 0.48000000000000009 | 0.0 | 0.0 | 0.0 | None | 0.0 | 0.006410256410256412 | 0.0 | None | 0.012578616352201295 | 0.0173913043478261 | 0.016129032258064505 | 0.024390243902439 | 0.0 | 0.0612244897959184 | 0.022727272727272717 | 0.02797202797202801 | 0.0 | 0.0299625468164794 | 0.0 |
| 0.5 | 0.0 | 0.0 | 0.0 | None | 0.0303030303030303 | 0.019230769230769208 | 0.0 | None | 0.012578616352201295 | 0.0 | 0.0 | 0.0 | 0.0 | 0.030612244897959207 | 0.0 | 0.006993006993006992 | 0.0173913043478261 | 0.02621722846441952 | 0.0 |
| 0.52 | 0.0 | 0.0 | 0.0 | None | 0.015151515151515204 | 0.006410256410256412 | 0.0 | None | 0.006289308176100634 | 0.0 | 0.0 | 0.0 | 0.0136986301369863 | 0.0 | 0.022727272727272717 | 0.013986013986014 | 0.008695652173913045 | 0.003745318352059932 | 0.03125 |
| 0.54 | 0.03225806451612901 | 0.0 | 0.0 | None | 0.0303030303030303 | 0.012820512820512801 | 0.0 | None | 0.0 | 0.0 | 0.0 | 0.0 | 0.0 | 0.030612244897959207 | 0.0 | 0.006993006993006992 | 0.0 | 0.007490636704119854 | 0.0 |
| 0.56000000000000005 | 0.0 | 0.022727272727272717 | 0.0 | None | 0.0 | 0.006410256410256412 | 0.0163934426229508 | None | 0.006289308176100634 | 0.0 | 0.0 | 0.0 | 0.0 | 0.030612244897959207 | 0.015151515151515204 | 0.020979020979021008 | 0.0 | 0.0 | 0.0 |
| 0.58000000000000007 | 0.0 | 0.0 | 0.0 | None | 0.0 | 0.006410256410256412 | 0.0 | None | 0.012578616352201295 | 0.0 | 0.0 | 0.0 | 0.0 | 0.010204081632653106 | 0.0 | 0.006993006993006992 | 0.008695652173913045 | 0.007490636704119854 | 0.0 |
| 0.6000000000000002 | 0.03225806451612901 | 0.0 | 0.0 | None | 0.0303030303030303 | 0.0 | 0.0 | None | 0.006289308176100634 | 0.0 | 0.016129032258064505 | 0.0 | 0.0 | 0.010204081632653106 | 0.0 | 0.006993006993006992 | 0.0 | 0.0 | 0.0 |
### Chart: cat-1 A1
| Category | cat-1(CB1111)_A1_01 | cat-1(CB1111)_A1_02 | cat-1(CB1111)_A1_03 | cat-1(CB1111)_A1_04 | cat-1(CB1111)_A1_05 | cat-1(CB1111)_A1_06 | cat-1(CB1111)_A1_07 | cat-1(CB1111)_A1_08 | cat-1(CB1111)_A1_09 | cat-1(CB1111)_A1_10 | cat-1(CB1111)_A1_12 | cat-1(CB1111)_A1_13 | cat-1(CB1111)_A1_14 | cat-1(CB1111)_A1_15 | cat-1(CB1111)_A1_16 | cat-1(CB1111)_A1_17 | cat-1(CB1111)_A1_18 | cat-1(CB1111)_A1_19 | cat-1(CB1111)_A1_20 |
|---|---|---|---|---|---|---|---|---|---|---|---|---|---|---|---|---|---|---|---|
| 0 | None | None | None | None | None | None | None | None | None | None | None | None | None | None | None | None | None | None | None |
| 2.0000000000000007E-2 | 0.04123711340206188 | 0.047101449275362285 | 0.10638297872340402 | 0.07228915662650597 | 0.07443365695792885 | 0.08898944193061843 | 0.0603448275862069 | 0.0518234165067179 | 0.05428571428571431 | 0.0791738382099828 | 0.0442890442890443 | 0.07327586206896546 | 0.045081967213114804 | 0.05330490405117272 | 0.05622489959839358 | 0.0414201183431953 | 0.0251396648044693 | 0.053712480252764636 | 0.0782608695652174 |
| 4.0000000000000015E-2 | 0.0618556701030928 | 0.0579710144927536 | 0.09574468085106383 | 0.0662650602409639 | 0.03883495145631071 | 0.0512820512820513 | 0.0603448275862069 | 0.07293666026871402 | 0.05428571428571431 | 0.08261617900172119 | 0.0233100233100233 | 0.04094827586206898 | 0.0327868852459016 | 0.0383795309168444 | 0.04417670682730923 | 0.023668639053254392 | 0.019553072625698303 | 0.0394944707740916 | 0.048695652173913 |
| 6.0000000000000019E-2 | 0.0618556701030928 | 0.0289855072463768 | 0.07446808510638303 | 0.07831325301204824 | 0.06148867313915862 | 0.07541478129713425 | 0.08189655172413793 | 0.0518234165067179 | 0.028571428571428602 | 0.06196213425129092 | 0.016317016317016302 | 0.03879310344827591 | 0.0327868852459016 | 0.057569296375266504 | 0.02811244979919679 | 0.03254437869822492 | 0.0251396648044693 | 0.05055292259083732 | 0.0660869565217391 |
| 8.0000000000000029E-2 | 0.04123711340206188 | 0.03260869565217391 | 0.031914893617021316 | 0.036144578313253 | 0.051779935275080895 | 0.05429864253393673 | 0.05172413793103452 | 0.0633397312859885 | 0.0685714285714286 | 0.06540447504302933 | 0.048951048951049 | 0.0603448275862069 | 0.04303278688524593 | 0.057569296375266504 | 0.0542168674698795 | 0.03254437869822492 | 0.0251396648044693 | 0.05845181674565559 | 0.055652173913043515 |
| 0.1 | 0.010309278350515504 | 0.036231884057971016 | 0.06382978723404252 | 0.09036144578313256 | 0.0453074433656958 | 0.0588235294117647 | 0.04741379310344834 | 0.0671785028790787 | 0.05428571428571431 | 0.05507745266781408 | 0.016317016317016302 | 0.03017241379310351 | 0.0389344262295082 | 0.0341151385927505 | 0.04216867469879522 | 0.047337278106508916 | 0.0391061452513966 | 0.0631911532385466 | 0.0504347826086957 |
| 0.12000000000000002 | 0.010309278350515504 | 0.0543478260869565 | 0.0531914893617021 | 0.0542168674698795 | 0.058252427184466014 | 0.06485671191553555 | 0.02155172413793099 | 0.07293666026871402 | 0.05428571428571431 | 0.051635111876075696 | 0.02797202797202801 | 0.0581896551724138 | 0.040983606557377004 | 0.04264392324093822 | 0.036144578313253 | 0.0414201183431953 | 0.036312849162011204 | 0.05213270142180092 | 0.0504347826086957 |
| 0.14000000000000001 | 0.0309278350515464 | 0.06521739130434782 | 0.042553191489361715 | 0.07228915662650597 | 0.058252427184466014 | 0.07239819004524894 | 0.05603448275862072 | 0.0633397312859885 | 0.03142857142857142 | 0.06196213425129092 | 0.02797202797202801 | 0.06465517241379308 | 0.05942622950819673 | 0.0447761194029851 | 0.0502008032128514 | 0.047337278106508916 | 0.02234636871508381 | 0.045813586097946335 | 0.038260869565217404 |
| 0.16 | 0.0309278350515464 | 0.03985507246376811 | 0.031914893617021316 | 0.0542168674698795 | 0.055016181229773524 | 0.0693815987933635 | 0.02155172413793099 | 0.047984644913627625 | 0.0514285714285714 | 0.08089500860585205 | 0.041958041958042015 | 0.0603448275862069 | 0.061475409836065614 | 0.04051172707889132 | 0.0502008032128514 | 0.0384615384615385 | 0.0391061452513966 | 0.047393364928909935 | 0.05391304347826088 |
| 0.18000000000000005 | 0.0309278350515464 | 0.036231884057971016 | 0.010638297872340394 | 0.0542168674698795 | 0.07119741100323625 | 0.05429864253393673 | 0.04310344827586208 | 0.053742802303262976 | 0.045714285714285714 | 0.03442340791738381 | 0.0233100233100233 | 0.0538793103448276 | 0.0573770491803279 | 0.05117270788912581 | 0.0502008032128514 | 0.0384615384615385 | 0.02234636871508381 | 0.05529225908372832 | 0.05913043478260874 |
| 0.2 | 0.02061855670103089 | 0.0507246376811594 | 0.0212765957446809 | 0.04216867469879522 | 0.06472491909385111 | 0.0663650075414781 | 0.04741379310344834 | 0.059500959692898314 | 0.06571428571428573 | 0.030981067125645412 | 0.03729603729603731 | 0.0538793103448276 | 0.0573770491803279 | 0.057569296375266504 | 0.0321285140562249 | 0.047337278106508916 | 0.030726256983240198 | 0.06951026856240132 | 0.048695652173913 |
| 0.22 | 0.0309278350515464 | 0.07246376811594203 | 0.06382978723404252 | 0.0542168674698795 | 0.03559870550161811 | 0.0271493212669683 | 0.017241379310344803 | 0.059500959692898314 | 0.0514285714285714 | 0.051635111876075696 | 0.0442890442890443 | 0.03879310344827591 | 0.0532786885245902 | 0.04690831556503198 | 0.036144578313253 | 0.029585798816568 | 0.0558659217877095 | 0.05213270142180092 | 0.0365217391304348 |
| 0.24000000000000005 | 0.010309278350515504 | 0.0543478260869565 | 0.0531914893617021 | 0.04216867469879522 | 0.042071197411003215 | 0.03469079939668179 | 0.06465517241379308 | 0.032629558541266805 | 0.0571428571428571 | 0.0413080895008606 | 0.0536130536130536 | 0.07758620689655173 | 0.04303278688524593 | 0.07249466950959492 | 0.0321285140562249 | 0.053254437869822514 | 0.0391061452513966 | 0.047393364928909935 | 0.03304347826086961 |
| 0.26 | 0.04123711340206188 | 0.03260869565217391 | 0.031914893617021316 | 0.06024096385542172 | 0.0453074433656958 | 0.0331825037707391 | 0.03879310344827591 | 0.0383877159309021 | 0.04285714285714291 | 0.04475043029259902 | 0.0652680652680653 | 0.04956896551724142 | 0.04303278688524593 | 0.0447761194029851 | 0.05622489959839358 | 0.06804733727810651 | 0.0558659217877095 | 0.053712480252764636 | 0.048695652173913 |
| 0.28000000000000008 | 0.0309278350515464 | 0.0289855072463768 | 0.031914893617021316 | 0.04216867469879522 | 0.058252427184466014 | 0.04977375565610863 | 0.0603448275862069 | 0.0345489443378119 | 0.0571428571428571 | 0.0395869191049914 | 0.07692307692307691 | 0.04741379310344834 | 0.03483606557377053 | 0.061833688699360304 | 0.05220883534136549 | 0.047337278106508916 | 0.07262569832402241 | 0.0394944707740916 | 0.0434782608695652 |
| 0.3000000000000001 | 0.0309278350515464 | 0.036231884057971016 | 0.0212765957446809 | 0.03012048192771079 | 0.042071197411003215 | 0.02564102564102561 | 0.0258620689655172 | 0.02879078694817659 | 0.04285714285714291 | 0.022375215146299518 | 0.041958041958042015 | 0.04741379310344834 | 0.045081967213114804 | 0.02558635394456289 | 0.05622489959839358 | 0.0414201183431953 | 0.07262569832402241 | 0.053712480252764636 | 0.0278260869565217 |
| 0.32000000000000012 | 0.0618556701030928 | 0.0434782608695652 | 0.010638297872340394 | 0.012048192771084298 | 0.016181229773462806 | 0.024132730015083 | 0.03448275862068972 | 0.0345489443378119 | 0.028571428571428602 | 0.0206540447504303 | 0.0606060606060606 | 0.036637931034482804 | 0.04303278688524593 | 0.0383795309168444 | 0.036144578313253 | 0.0621301775147929 | 0.0558659217877095 | 0.0442338072669826 | 0.03304347826086961 |
| 0.34 | 0.0515463917525773 | 0.0289855072463768 | 0.031914893617021316 | 0.0180722891566265 | 0.042071197411003215 | 0.024132730015083 | 0.04310344827586208 | 0.02111324376199621 | 0.03142857142857142 | 0.030981067125645412 | 0.055944055944055895 | 0.0258620689655172 | 0.051229508196721285 | 0.0341151385927505 | 0.02811244979919679 | 0.0355029585798817 | 0.058659217877095 | 0.02685624012638231 | 0.038260869565217404 |
| 0.3600000000000001 | 0.04123711340206188 | 0.0217391304347826 | 0.0212765957446809 | 0.012048192771084298 | 0.02265372168284791 | 0.0105580693815988 | 0.017241379310344803 | 0.02111324376199621 | 0.020000000000000007 | 0.0172117039586919 | 0.0466200466200466 | 0.017241379310344803 | 0.02663934426229512 | 0.03624733475479741 | 0.0502008032128514 | 0.05621301775147931 | 0.053072625698324 | 0.023696682464455002 | 0.0173913043478261 |
| 0.38000000000000012 | 0.0515463917525773 | 0.025362318840579712 | 0.0 | 0.012048192771084298 | 0.006472491909385112 | 0.015082956259426801 | 0.02155172413793099 | 0.019193857965451103 | 0.03142857142857142 | 0.0172117039586919 | 0.03962703962703961 | 0.015086206896551699 | 0.0307377049180328 | 0.023454157782516007 | 0.03012048192771079 | 0.047337278106508916 | 0.050279329608938585 | 0.018957345971564 | 0.024347826086956507 |
| 0.4 | 0.04123711340206188 | 0.003623188405797101 | 0.0 | 0.0 | 0.00970873786407767 | 0.0196078431372549 | 0.008620689655172415 | 0.017274472168906 | 0.011428571428571404 | 0.008605851979345954 | 0.032634032634032605 | 0.01939655172413791 | 0.0204918032786885 | 0.008528784648187635 | 0.0180722891566265 | 0.029585798816568 | 0.02234636871508381 | 0.0126382306477093 | 0.0156521739130435 |
| 0.4200000000000001 | 0.02061855670103089 | 0.025362318840579712 | 0.0 | 0.012048192771084298 | 0.00970873786407767 | 0.015082956259426801 | 0.0 | 0.0134357005758157 | 0.020000000000000007 | 0.012048192771084298 | 0.041958041958042015 | 0.010775862068965504 | 0.024590163934426198 | 0.021321961620469117 | 0.0261044176706827 | 0.014792899408284 | 0.0391061452513966 | 0.015797788309636705 | 0.0173913043478261 |
| 0.44 | 0.010309278350515504 | 0.0144927536231884 | 0.010638297872340394 | 0.0180722891566265 | 0.00970873786407767 | 0.007541478129713423 | 0.0129310344827586 | 0.009596928982725527 | 0.00857142857142857 | 0.0 | 0.0303030303030303 | 0.0129310344827586 | 0.0204918032786885 | 0.023454157782516007 | 0.0261044176706827 | 0.02662721893491121 | 0.030726256983240198 | 0.0031595576619273323 | 0.0156521739130435 |
| 0.46 | 0.0309278350515464 | 0.010869565217391306 | 0.010638297872340394 | 0.0 | 0.0 | 0.0030165912518853727 | 0.008620689655172415 | 0.005758157389635322 | 0.011428571428571404 | 0.00516351118760757 | 0.013986013986014 | 0.0129310344827586 | 0.0122950819672131 | 0.008528784648187635 | 0.012048192771084298 | 0.014792899408284 | 0.02234636871508381 | 0.009478672985781995 | 0.0104347826086957 |
| 0.48000000000000009 | 0.0 | 0.010869565217391306 | 0.0 | 0.0 | 0.00970873786407767 | 0.00150829562594268 | 0.0 | 0.0 | 0.00285714285714286 | 0.003442340791738381 | 0.0233100233100233 | 0.010775862068965504 | 0.00819672131147541 | 0.008528784648187635 | 0.00803212851405622 | 0.017751479289940808 | 0.013966480446927408 | 0.004739336492891005 | 0.006956521739130442 |
| 0.5 | 0.0 | 0.010869565217391306 | 0.0 | 0.006024096385542173 | 0.00323624595469256 | 0.00150829562594268 | 0.0 | 0.0019193857965451105 | 0.0 | 0.003442340791738381 | 0.011655011655011701 | 0.002155172413793099 | 0.014344262295082004 | 0.0106609808102345 | 0.012048192771084298 | 0.005917159763313612 | 0.008379888268156424 | 0.004739336492891005 | 0.0017391304347826105 |
| 0.52 | 0.010309278350515504 | 0.0 | 0.0 | 0.0 | 0.0 | 0.0030165912518853727 | 0.008620689655172415 | 0.0019193857965451105 | 0.00285714285714286 | 0.0 | 0.006993006993006992 | 0.0 | 0.00409836065573771 | 0.006396588486140722 | 0.006024096385542173 | 0.011834319526627201 | 0.005586592178770952 | 0.0015797788309636705 | 0.0104347826086957 |
| 0.54 | 0.010309278350515504 | 0.0 | 0.0 | 0.0 | 0.0 | 0.0030165912518853727 | 0.008620689655172415 | 0.0 | 0.00571428571428571 | 0.0017211703958691898 | 0.009324009324009324 | 0.0 | 0.00409836065573771 | 0.006396588486140722 | 0.002008032128514061 | 0.0029585798816568008 | 0.002793296089385481 | 0.0015797788309636705 | 0.0017391304347826105 |
| 0.56000000000000005 | 0.0 | 0.0 | 0.0 | 0.0 | 0.00323624595469256 | 0.0 | 0.0 | 0.0 | 0.0 | 0.0017211703958691898 | 0.009324009324009324 | 0.002155172413793099 | 0.002049180327868851 | 0.0 | 0.0 | 0.0 | 0.0 | 0.0015797788309636705 | 0.0 |
| 0.58000000000000007 | 0.0 | 0.0 | 0.0 | 0.0 | 0.0 | 0.00150829562594268 | 0.0 | 0.0 | 0.0 | 0.0017211703958691898 | 0.0 | 0.0 | 0.00614754098360656 | 0.0021321961620469117 | 0.0 | 0.0 | 0.002793296089385481 | 0.0 | 0.0 |
| 0.6000000000000002 | 0.0 | 0.003623188405797101 | 0.0 | 0.0 | 0.0 | 0.0 | 0.008620689655172415 | 0.0 | 0.0 | 0.0 | 0.0 | 0.0 | 0.002049180327868851 | 0.0021321961620469117 | 0.0 | 0.0 | 0.0 | 0.0 | 0.0 |
### Chart: cat-1 A3
| Category | cat-1(CB1111)_A3_01 | cat-1(CB1111)_A3_02 | cat-1(CB1111)_A3_03 | cat-1(CB1111)_A3_04 | cat-1(CB1111)_A3_05 | cat-1(CB1111)_A3_06 | cat-1(CB1111)_A3_07 | cat-1(CB1111)_A3_08 | cat-1(CB1111)_A3_09 | cat-1(CB1111)_A3_10 | cat-1(CB1111)_A3_11 | cat-1(CB1111)_A3_12 | cat-1(CB1111)_A3_13 | cat-1(CB1111)_A3_14 | cat-1(CB1111)_A3_15 | cat-1(CB1111)_A3_16 | cat-1(CB1111)_A3_17 | cat-1(CB1111)_A3_18 | cat-1(CB1111)_A3_19 | cat-1(CB1111)_A3_20 |
|---|---|---|---|---|---|---|---|---|---|---|---|---|---|---|---|---|---|---|---|---|
| 0 | None | None | None | None | None | None | None | None | None | None | None | None | None | None | None | None | None | None | None | None |
| 2.0000000000000007E-2 | 0.01984126984126981 | 0.0385964912280702 | 0.020408163265306107 | 0.012987012987013 | 0.0421455938697318 | 0.02777777777777782 | 0.027343750000000007 | 0.02229299363057321 | 0.04922279792746113 | 0.011363636363636404 | 0.014018691588784996 | 0.012048192771084298 | 0.015789473684210506 | 0.005813953488372092 | 0.0 | 0.003355704697986581 | 0.0034843205574912927 | 0.00389105058365759 | 0.011799410029498499 | 0.037542662116041 |
| 4.0000000000000015E-2 | 0.011904761904761904 | 0.028070175438596506 | 0.004081632653061222 | 0.017316017316017306 | 0.026819923371647507 | 0.039682539682539715 | 0.03125 | 0.04777070063694272 | 0.0336787564766839 | 0.011363636363636404 | 0.0 | 0.0 | 0.0105263157894737 | 0.017441860465116307 | 0.0 | 0.006711409395973156 | 0.0034843205574912927 | 0.0 | 0.00294985250737463 | 0.013651877133105804 |
| 6.0000000000000019E-2 | 0.0039682539682539715 | 0.0210526315789474 | 0.0 | 0.012987012987013 | 0.0114942528735632 | 0.043650793650793614 | 0.019531250000000003 | 0.035031847133758016 | 0.04922279792746113 | 0.015151515151515204 | 0.0 | 0.0 | 0.015789473684210506 | 0.0 | 0.0 | 0.003355704697986581 | 0.0 | 0.0 | 0.0 | 0.010238907849829398 |
| 8.0000000000000029E-2 | 0.0039682539682539715 | 0.017543859649122813 | 0.0 | 0.021645021645021606 | 0.04597701149425289 | 0.02777777777777782 | 0.027343750000000007 | 0.03184713375796181 | 0.04922279792746113 | 0.015151515151515204 | 0.004672897196261682 | 0.0040160642570281095 | 0.005263157894736842 | 0.005813953488372092 | 0.0 | 0.0 | 0.0 | 0.00389105058365759 | 0.0 | 0.006825938566552898 |
| 0.1 | 0.01984126984126981 | 0.0245614035087719 | 0.004081632653061222 | 0.004329004329004332 | 0.015325670498084304 | 0.05555555555555558 | 0.019531250000000003 | 0.0414012738853503 | 0.05181347150259072 | 0.003787878787878792 | 0.009345794392523367 | 0.0 | 0.005263157894736842 | 0.005813953488372092 | 0.00363636363636364 | 0.003355704697986581 | 0.0034843205574912927 | 0.0 | 0.0 | 0.010238907849829398 |
| 0.12000000000000002 | 0.0 | 0.0210526315789474 | 0.0 | 0.004329004329004332 | 0.03065134099616861 | 0.01984126984126981 | 0.0078125 | 0.0286624203821656 | 0.054404145077720185 | 0.007575757575757582 | 0.004672897196261682 | 0.0 | 0.015789473684210506 | 0.005813953488372092 | 0.0 | 0.003355704697986581 | 0.0 | 0.00389105058365759 | 0.00294985250737463 | 0.0 |
| 0.14000000000000001 | 0.007936507936507941 | 0.0421052631578947 | 0.004081632653061222 | 0.025974025974026007 | 0.03448275862068972 | 0.01984126984126981 | 0.019531250000000003 | 0.0382165605095541 | 0.025906735751295307 | 0.007575757575757582 | 0.004672897196261682 | 0.0 | 0.005263157894736842 | 0.005813953488372092 | 0.00363636363636364 | 0.0 | 0.0034843205574912927 | 0.011673151750972804 | 0.0058997050147492625 | 0.006825938566552898 |
| 0.16 | 0.0 | 0.0210526315789474 | 0.004081632653061222 | 0.012987012987013 | 0.03065134099616861 | 0.023809523809523808 | 0.03125 | 0.02229299363057321 | 0.05958549222797931 | 0.011363636363636404 | 0.004672897196261682 | 0.00803212851405622 | 0.015789473684210506 | 0.005813953488372092 | 0.0 | 0.0 | 0.0 | 0.00778210116731518 | 0.00294985250737463 | 0.010238907849829398 |
| 0.18000000000000005 | 0.0039682539682539715 | 0.0456140350877193 | 0.0 | 0.017316017316017306 | 0.015325670498084304 | 0.0317460317460317 | 0.011718750000000003 | 0.012738853503184698 | 0.0414507772020725 | 0.003787878787878792 | 0.004672897196261682 | 0.0200803212851406 | 0.0105263157894737 | 0.011627906976744195 | 0.00363636363636364 | 0.0100671140939597 | 0.0034843205574912927 | 0.011673151750972804 | 0.0058997050147492625 | 0.006825938566552898 |
| 0.2 | 0.023809523809523808 | 0.028070175438596506 | 0.012244897959183699 | 0.004329004329004332 | 0.022988505747126402 | 0.0158730158730159 | 0.015625 | 0.00955414012738853 | 0.03626943005181352 | 0.0 | 0.028037383177570117 | 0.0200803212851406 | 0.0105263157894737 | 0.005813953488372092 | 0.025454545454545514 | 0.013422818791946301 | 0.020905923344947692 | 0.00778210116731518 | 0.00294985250737463 | 0.006825938566552898 |
| 0.22 | 0.0515873015873016 | 0.0245614035087719 | 0.03673469387755101 | 0.004329004329004332 | 0.026819923371647507 | 0.0158730158730159 | 0.015625 | 0.035031847133758016 | 0.03626943005181352 | 0.011363636363636404 | 0.04672897196261682 | 0.0200803212851406 | 0.015789473684210506 | 0.0232558139534884 | 0.07636363636363645 | 0.0335570469798658 | 0.0592334494773519 | 0.04280155642023351 | 0.0058997050147492625 | 0.006825938566552898 |
| 0.24000000000000005 | 0.08730158730158726 | 0.07368421052631581 | 0.0530612244897959 | 0.0303030303030303 | 0.03448275862068972 | 0.023809523809523808 | 0.027343750000000007 | 0.0286624203821656 | 0.05181347150259072 | 0.0265151515151515 | 0.06074766355140192 | 0.0642570281124498 | 0.0210526315789474 | 0.04651162790697674 | 0.08363636363636368 | 0.07718120805369133 | 0.08710801393728221 | 0.0739299610894942 | 0.02654867256637171 | 0.010238907849829398 |
| 0.26 | 0.126984126984127 | 0.09473684210526329 | 0.06938775510204083 | 0.0606060606060606 | 0.05363984674329497 | 0.047619047619047616 | 0.0390625 | 0.0382165605095541 | 0.062176165803108814 | 0.0303030303030303 | 0.11214953271028003 | 0.100401606425703 | 0.052631578947368404 | 0.05232558139534882 | 0.123636363636364 | 0.12416107382550304 | 0.101045296167247 | 0.10505836575875498 | 0.079646017699115 | 0.010238907849829398 |
| 0.28000000000000008 | 0.15873015873015905 | 0.08421052631578953 | 0.14285714285714307 | 0.09090909090909094 | 0.05363984674329497 | 0.07142857142857141 | 0.09765625000000003 | 0.0668789808917197 | 0.04404145077720208 | 0.0681818181818182 | 0.11682242990654203 | 0.11646586345381503 | 0.12105263157894702 | 0.07558139534883723 | 0.14181818181818207 | 0.120805369127517 | 0.18118466898954688 | 0.11284046692607 | 0.10324483775811204 | 0.023890784982935197 |
| 0.3000000000000001 | 0.18650793650793715 | 0.0807017543859649 | 0.17959183673469406 | 0.10822510822510806 | 0.0804597701149425 | 0.0793650793650794 | 0.12890625000000006 | 0.07006369426751592 | 0.046632124352331626 | 0.09469696969696977 | 0.12616822429906493 | 0.160642570281124 | 0.105263157894737 | 0.14534883720930206 | 0.19636363636363593 | 0.12416107382550304 | 0.13240418118466907 | 0.159533073929961 | 0.138643067846608 | 0.04095563139931742 |
| 0.32000000000000012 | 0.09523809523809525 | 0.08421052631578953 | 0.14285714285714307 | 0.11688311688311702 | 0.10727969348659006 | 0.043650793650793614 | 0.09765625000000003 | 0.07643312101910833 | 0.08290155440414503 | 0.12121212121212104 | 0.18691588785046717 | 0.1285140562249 | 0.105263157894737 | 0.0988372093023256 | 0.11636363636363603 | 0.11073825503355703 | 0.101045296167247 | 0.151750972762646 | 0.13274336283185806 | 0.07849829351535843 |
| 0.34 | 0.07142857142857141 | 0.07368421052631581 | 0.122448979591837 | 0.07792207792207793 | 0.061302681992337245 | 0.111111111111111 | 0.13671875000000006 | 0.0987261146496815 | 0.046632124352331626 | 0.170454545454545 | 0.07009345794392526 | 0.104417670682731 | 0.08947368421052623 | 0.104651162790698 | 0.06909090909090912 | 0.12751677852349005 | 0.10452961672473902 | 0.10116731517509697 | 0.1710914454277291 | 0.112627986348123 |
| 0.3600000000000001 | 0.0674603174603175 | 0.0701754385964912 | 0.0530612244897959 | 0.09523809523809525 | 0.04980842911877392 | 0.07142857142857141 | 0.0625 | 0.0573248407643312 | 0.0336787564766839 | 0.13257575757575796 | 0.102803738317757 | 0.08433734939759047 | 0.110526315789474 | 0.0930232558139535 | 0.06909090909090912 | 0.0973154362416107 | 0.09059233449477352 | 0.05058365758754862 | 0.079646017699115 | 0.061433447098976114 |
| 0.38000000000000012 | 0.007936507936507941 | 0.0245614035087719 | 0.07346938775510202 | 0.09090909090909094 | 0.05747126436781611 | 0.0674603174603175 | 0.046874999999999986 | 0.0636942675159236 | 0.0284974093264249 | 0.08333333333333333 | 0.0327102803738318 | 0.04819277108433742 | 0.08421052631578953 | 0.08139534883720934 | 0.03272727272727272 | 0.04697986577181206 | 0.04181184668989548 | 0.06614785992217899 | 0.07079646017699112 | 0.119453924914676 |
| 0.4 | 0.0317460317460317 | 0.007017543859649122 | 0.024489795918367314 | 0.0735930735930736 | 0.0383141762452107 | 0.039682539682539715 | 0.042968750000000014 | 0.02229299363057321 | 0.0207253886010363 | 0.0606060606060606 | 0.018691588785046703 | 0.0240963855421687 | 0.031578947368421116 | 0.040697674418604717 | 0.025454545454545514 | 0.0335570469798658 | 0.024390243902439 | 0.0233463035019455 | 0.0412979351032448 | 0.10580204778157005 |
| 0.4200000000000001 | 0.0039682539682539715 | 0.0105263157894737 | 0.012244897959183699 | 0.0346320346320346 | 0.022988505747126402 | 0.02777777777777782 | 0.0078125 | 0.0414012738853503 | 0.015544041450777204 | 0.053030303030303 | 0.014018691588784996 | 0.0200803212851406 | 0.0421052631578947 | 0.040697674418604717 | 0.010909090909090898 | 0.030201342281879227 | 0.010452961672473898 | 0.0350194552529183 | 0.03244837758112093 | 0.05802047781569972 |
| 0.44 | 0.0 | 0.017543859649122813 | 0.012244897959183699 | 0.021645021645021606 | 0.0076628352490421495 | 0.0158730158730159 | 0.011718750000000003 | 0.02229299363057321 | 0.0181347150259067 | 0.0189393939393939 | 0.014018691588784996 | 0.02811244979919679 | 0.0210526315789474 | 0.011627906976744195 | 0.0 | 0.006711409395973156 | 0.006968641114982585 | 0.0 | 0.0412979351032448 | 0.0716723549488055 |
| 0.46 | 0.0039682539682539715 | 0.0245614035087719 | 0.00816326530612245 | 0.025974025974026007 | 0.026819923371647507 | 0.0 | 0.00390625 | 0.02229299363057321 | 0.005181347150259074 | 0.0 | 0.004672897196261682 | 0.012048192771084298 | 0.005263157894736842 | 0.011627906976744195 | 0.0 | 0.003355704697986581 | 0.0034843205574912927 | 0.011673151750972804 | 0.011799410029498499 | 0.04095563139931742 |
| 0.48000000000000009 | 0.0039682539682539715 | 0.003508771929824561 | 0.004081632653061222 | 0.008658008658008665 | 0.0114942528735632 | 0.0 | 0.00390625 | 0.012738853503184698 | 0.00259067357512953 | 0.011363636363636404 | 0.0 | 0.00803212851405622 | 0.0105263157894737 | 0.005813953488372092 | 0.00363636363636364 | 0.0 | 0.0 | 0.0 | 0.011799410029498499 | 0.0341296928327645 |
| 0.5 | 0.0 | 0.0 | 0.0 | 0.004329004329004332 | 0.003831417624521071 | 0.011904761904761904 | 0.00390625 | 0.0 | 0.00259067357512953 | 0.0 | 0.004672897196261682 | 0.0040160642570281095 | 0.0105263157894737 | 0.011627906976744195 | 0.00363636363636364 | 0.003355704697986581 | 0.0 | 0.011673151750972804 | 0.0058997050147492625 | 0.023890784982935197 |
| 0.52 | 0.0 | 0.0105263157894737 | 0.0 | 0.004329004329004332 | 0.003831417624521071 | 0.0039682539682539715 | 0.0 | 0.0 | 0.0 | 0.003787878787878792 | 0.0 | 0.0 | 0.0 | 0.017441860465116307 | 0.0 | 0.003355704697986581 | 0.0034843205574912927 | 0.0 | 0.0 | 0.0341296928327645 |
| 0.54 | 0.0 | 0.0 | 0.0 | 0.0 | 0.0 | 0.0 | 0.0 | 0.0 | 0.0 | 0.0 | 0.0 | 0.00803212851405622 | 0.0 | 0.005813953488372092 | 0.0 | 0.0 | 0.0034843205574912927 | 0.0 | 0.00294985250737463 | 0.006825938566552898 |
| 0.56000000000000005 | 0.0 | 0.0 | 0.0 | 0.0 | 0.0 | 0.0 | 0.00390625 | 0.0 | 0.00259067357512953 | 0.0 | 0.0 | 0.0040160642570281095 | 0.0 | 0.0 | 0.0 | 0.0 | 0.0 | 0.0 | 0.00294985250737463 | 0.006825938566552898 |
| 0.58000000000000007 | 0.0 | 0.0 | 0.0 | 0.0 | 0.0 | 0.0 | 0.0 | 0.0 | 0.0 | 0.0 | 0.0 | 0.0 | 0.005263157894736842 | 0.011627906976744195 | 0.0 | 0.003355704697986581 | 0.0 | 0.0 | 0.0 | 0.003412969283276451 |
| 0.6000000000000002 | 0.0 | 0.0 | 0.0 | 0.0 | 0.0 | 0.0 | 0.0 | 0.0 | 0.0 | 0.0 | 0.0 | 0.0 | 0.0 | 0.0 | 0.0 | 0.0 | 0.0 | 0.0 | 0.0 | 0.003412969283276451 |
### Chart: cat-1 A5
| Category | cat-1(CB1111)_A5_01 | cat-1(CB1111)_A5_02 | cat-1(CB1111)_A5_03 | cat-1(CB1111)_A5_04 | cat-1(CB1111)_A5_05 | cat-1(CB1111)_A5_06 | cat-1(CB1111)_A5_07 | cat-1(CB1111)_A5_08 | cat-1(CB1111)_A5_10 | cat-1(CB1111)_A5_11 | cat-1(CB1111)_A5_12 | cat-1(CB1111)_A5_13 | cat-1(CB1111)_A5_14 | cat-1(CB1111)_A5_15 | cat-1(CB1111)_A5_16 | cat-1(CB1111)_A5_17 | cat-1(CB1111)_A5_18 | cat-1(CB1111)_A5_19 | cat-1(CB1111)_A5_20 | cat-1(CB1111)_A5_21 | cat-1(CB1111)_A5_22 | cat-1(CB1111)_A5_23 | cat-1(CB1111)_A5_24 | cat-1(CB1111)_A5_25 | cat-1(CB1111)_A5_26 | cat-1(CB1111)_A5_27 | cat-1(CB1111)_A5_28 | cat-1(CB1111)_A5_29 | cat-1(CB1111)_A5_30 |
|---|---|---|---|---|---|---|---|---|---|---|---|---|---|---|---|---|---|---|---|---|---|---|---|---|---|---|---|---|---|
| 0 | None | None | None | None | None | None | None | None | None | None | None | None | None | None | None | None | None | None | None | None | None | None | None | None | None | None | None | None | None |
| 2.0000000000000007E-2 | None | 0.0512820512820513 | 0.028301886792452793 | 0.08443271767810023 | 0.023880597014925408 | 0.07629427792915533 | None | 0.0816326530612245 | 0.04065040650406497 | 0.0339622641509434 | 0.04216867469879522 | 0.12307692307692306 | 0.020000000000000007 | 0.02824858757062151 | 0.051948051948052 | 0.0746268656716418 | 0.05017921146953409 | 0.012944983818770201 | 0.047619047619047616 | 0.155555555555556 | 0.0388888888888889 | 0.0674846625766871 | 0.02347417840375591 | 0.0112359550561798 | 0.024896265560166008 | 0.004807692307692311 | 0.0125 | 0.0 | 0.111111111111111 |
| 4.0000000000000015E-2 | None | 0.0897435897435897 | 0.012578616352201295 | 0.09762532981530346 | 0.03582089552238811 | 0.09264305177111719 | None | 0.0612244897959184 | 0.024390243902439 | 0.0150943396226415 | 0.0331325301204819 | 0.015384615384615405 | 0.030000000000000002 | 0.0112994350282486 | 0.0649350649350649 | 0.0373134328358209 | 0.014336917562723992 | 0.00970873786407767 | 0.023809523809523808 | 0.0666666666666667 | 0.0388888888888889 | 0.07361963190184054 | 0.03286384976525821 | 0.0 | 0.00829875518672199 | 0.009615384615384628 | 0.00416666666666667 | 0.0 | 0.11695906432748497 |
| 6.0000000000000019E-2 | None | 0.02564102564102561 | 0.0440251572327044 | 0.07915567282321903 | 0.023880597014925408 | 0.0599455040871935 | None | 0.07482993197278913 | 0.0121951219512195 | 0.011320754716981109 | 0.0692771084337349 | 0.015384615384615405 | 0.020000000000000007 | 0.00564971751412429 | 0.051948051948052 | 0.0671641791044776 | 0.028673835125448008 | 0.02265372168284791 | 0.07142857142857141 | 0.037037037037037014 | 0.0611111111111111 | 0.049079754601227 | 0.03286384976525821 | 0.0112359550561798 | 0.024896265560166008 | 0.009615384615384628 | 0.0125 | 0.0 | 0.09356725146198833 |
| 8.0000000000000029E-2 | None | 0.0512820512820513 | 0.0408805031446541 | 0.08179419525065967 | 0.0417910447761194 | 0.05449591280653952 | None | 0.102040816326531 | 0.0121951219512195 | 0.03773584905660382 | 0.04518072289156631 | 0.0461538461538462 | 0.020000000000000007 | 0.00564971751412429 | 0.0 | 0.0522388059701493 | 0.035842293906810006 | 0.00970873786407767 | 0.023809523809523808 | 0.02962962962962961 | 0.0333333333333333 | 0.0 | 0.03286384976525821 | 0.005617977528089888 | 0.03734439834024901 | 0.014423076923076898 | 0.00833333333333333 | 0.0 | 0.017543859649122813 |
| 0.1 | None | 0.02564102564102561 | 0.02515723270440251 | 0.10026385224274402 | 0.026865671641791 | 0.05449591280653952 | None | 0.02721088435374149 | 0.0121951219512195 | 0.026415094339622597 | 0.0331325301204819 | 0.015384615384615405 | 0.005000000000000002 | 0.016949152542372906 | 0.012987012987013 | 0.0373134328358209 | 0.014336917562723992 | 0.00970873786407767 | 0.09523809523809525 | 0.0148148148148148 | 0.011111111111111101 | 0.012269938650306698 | 0.009389671361502353 | 0.0 | 0.004149377593361002 | 0.009615384615384628 | 0.00833333333333333 | 0.0 | 0.035087719298245605 |
| 0.12000000000000002 | None | 0.0384615384615385 | 0.028301886792452793 | 0.0659630606860158 | 0.056716417910447854 | 0.04904632152588563 | None | 0.04081632653061222 | 0.0121951219512195 | 0.0188679245283019 | 0.03012048192771079 | 0.015384615384615405 | 0.025 | 0.0 | 0.025974025974026007 | 0.0298507462686567 | 0.025089605734767 | 0.006472491909385112 | 0.023809523809523808 | 0.00740740740740741 | 0.011111111111111101 | 0.012269938650306698 | 0.00469483568075117 | 0.0 | 0.012448132780083 | 0.0 | 0.0 | 0.0 | 0.017543859649122813 |
| 0.14000000000000001 | None | 0.012820512820512801 | 0.0188679245283019 | 0.03957783641160952 | 0.04776119402985072 | 0.04359673024523164 | None | 0.020408163265306107 | 0.00813008130081301 | 0.0037735849056603826 | 0.03012048192771079 | 0.0 | 0.005000000000000002 | 0.00564971751412429 | 0.012987012987013 | 0.014925373134328401 | 0.03942652329749099 | 0.006472491909385112 | 0.023809523809523808 | 0.022222222222222202 | 0.011111111111111101 | 0.0184049079754601 | 0.009389671361502353 | 0.0112359550561798 | 0.00829875518672199 | 0.004807692307692311 | 0.0125 | 0.0 | 0.029239766081871312 |
| 0.16 | None | 0.0 | 0.0157232704402516 | 0.0527704485488127 | 0.04776119402985072 | 0.03542234332425071 | None | 0.05442176870748298 | 0.0121951219512195 | 0.0188679245283019 | 0.0331325301204819 | 0.0 | 0.025 | 0.016949152542372906 | 0.0 | 0.022388059701492494 | 0.010752688172042998 | 0.00323624595469256 | 0.0 | 0.022222222222222202 | 0.022222222222222202 | 0.012269938650306698 | 0.014084507042253504 | 0.0 | 0.012448132780083 | 0.004807692307692311 | 0.00416666666666667 | 0.0 | 0.017543859649122813 |
| 0.18000000000000005 | None | 0.012820512820512801 | 0.0188679245283019 | 0.03957783641160952 | 0.03582089552238811 | 0.027247956403269817 | None | 0.047619047619047616 | 0.00813008130081301 | 0.0150943396226415 | 0.0180722891566265 | 0.0 | 0.025 | 0.0 | 0.0 | 0.022388059701492494 | 0.021505376344086 | 0.006472491909385112 | 0.047619047619047616 | 0.00740740740740741 | 0.04444444444444442 | 0.012269938650306698 | 0.0 | 0.03932584269662921 | 0.024896265560166008 | 0.004807692307692311 | 0.00416666666666667 | 0.0 | 0.0 |
| 0.2 | None | 0.0384615384615385 | 0.022012578616352207 | 0.06068601583113463 | 0.0417910447761194 | 0.05177111716621248 | None | 0.034013605442176915 | 0.024390243902439 | 0.0 | 0.006024096385542173 | 0.0 | 0.030000000000000002 | 0.0 | 0.0 | 0.0074626865671641816 | 0.03942652329749099 | 0.012944983818770201 | 0.047619047619047616 | 0.0148148148148148 | 0.0166666666666667 | 0.02453987730061352 | 0.02347417840375591 | 0.028089887640449406 | 0.02904564315352701 | 0.014423076923076898 | 0.0125 | 0.0 | 0.029239766081871312 |
| 0.22 | None | 0.02564102564102561 | 0.028301886792452793 | 0.03957783641160952 | 0.0447761194029851 | 0.05449591280653952 | None | 0.0612244897959184 | 0.016260162601626 | 0.022641509433962315 | 0.03012048192771079 | 0.0 | 0.020000000000000007 | 0.0112994350282486 | 0.038961038961039 | 0.022388059701492494 | 0.021505376344086 | 0.025889967637540513 | 0.0 | 0.022222222222222202 | 0.0388888888888889 | 0.012269938650306698 | 0.028169014084506998 | 0.03370786516853931 | 0.03319502074688799 | 0.004807692307692311 | 0.02083333333333331 | 0.008620689655172415 | 0.00584795321637427 |
| 0.24000000000000005 | None | 0.012820512820512801 | 0.050314465408805 | 0.0580474934036939 | 0.0417910447761194 | 0.0790190735694823 | None | 0.020408163265306107 | 0.04471544715447153 | 0.011320754716981109 | 0.0180722891566265 | 0.030769230769230802 | 0.055000000000000014 | 0.0112994350282486 | 0.025974025974026007 | 0.0074626865671641816 | 0.03942652329749099 | 0.048543689320388314 | 0.0 | 0.02962962962962961 | 0.05555555555555558 | 0.030674846625766916 | 0.014084507042253504 | 0.028089887640449406 | 0.0580912863070539 | 0.004807692307692311 | 0.025 | 0.017241379310344803 | 0.0116959064327485 |
| 0.26 | None | 0.012820512820512801 | 0.06603773584905663 | 0.06332453825857523 | 0.032835820895522415 | 0.07356948228882831 | None | 0.047619047619047616 | 0.016260162601626 | 0.03773584905660382 | 0.04819277108433742 | 0.0 | 0.04500000000000001 | 0.0225988700564972 | 0.012987012987013 | 0.022388059701492494 | 0.05017921146953409 | 0.07766990291262142 | 0.047619047619047616 | 0.037037037037037014 | 0.0611111111111111 | 0.049079754601227 | 0.07511737089201882 | 0.06741573033707873 | 0.04149377593361002 | 0.014423076923076898 | 0.0333333333333333 | 0.06896551724137931 | 0.035087719298245605 |
| 0.28000000000000008 | None | 0.0384615384615385 | 0.10062893081761003 | 0.03957783641160952 | 0.0447761194029851 | 0.07356948228882831 | None | 0.020408163265306107 | 0.052845528455284584 | 0.0830188679245283 | 0.07831325301204824 | 0.0461538461538462 | 0.06000000000000002 | 0.0451977401129944 | 0.051948051948052 | 0.014925373134328401 | 0.0788530465949821 | 0.12297734627831702 | 0.023809523809523808 | 0.00740740740740741 | 0.05555555555555558 | 0.07361963190184054 | 0.10328638497652604 | 0.06741573033707873 | 0.0871369294605809 | 0.0288461538461538 | 0.025 | 0.03448275862068972 | 0.0116959064327485 |
| 0.3000000000000001 | None | 0.06410256410256411 | 0.09748427672955978 | 0.010554089709762505 | 0.05074626865671642 | 0.04904632152588563 | None | 0.02721088435374149 | 0.09349593495934964 | 0.0754716981132075 | 0.051204819277108383 | 0.07692307692307691 | 0.04500000000000001 | 0.12429378531073405 | 0.0 | 0.0298507462686567 | 0.05376344086021512 | 0.148867313915858 | 0.0 | 0.0148148148148148 | 0.0666666666666667 | 0.0858895705521472 | 0.09389671361502352 | 0.0898876404494382 | 0.0954356846473029 | 0.043269230769230796 | 0.08333333333333333 | 0.129310344827586 | 0.052631578947368404 |
| 0.32000000000000012 | None | 0.0384615384615385 | 0.110062893081761 | 0.010554089709762505 | 0.05970149253731343 | 0.016348773841961903 | None | 0.034013605442176915 | 0.12195121951219498 | 0.09056603773584918 | 0.0542168674698795 | 0.10769230769230798 | 0.1 | 0.129943502824859 | 0.025974025974026007 | 0.0522388059701493 | 0.07526881720430112 | 0.103559870550162 | 0.047619047619047616 | 0.02962962962962961 | 0.0777777777777778 | 0.07975460122699393 | 0.13145539906103307 | 0.10112359550561803 | 0.12448132780083 | 0.06730769230769233 | 0.108333333333333 | 0.155172413793103 | 0.0467836257309941 |
| 0.34 | None | 0.02564102564102561 | 0.06603773584905663 | 0.013192612137203198 | 0.0776119402985075 | 0.0108991825613079 | None | 0.02721088435374149 | 0.12601626016260206 | 0.135849056603774 | 0.04518072289156631 | 0.10769230769230798 | 0.1 | 0.107344632768362 | 0.0649350649350649 | 0.0298507462686567 | 0.08960573476702513 | 0.12297734627831702 | 0.047619047619047616 | 0.0148148148148148 | 0.0333333333333333 | 0.049079754601227 | 0.056338028169014086 | 0.0842696629213483 | 0.09958506224066399 | 0.09134615384615377 | 0.0958333333333333 | 0.155172413793103 | 0.0233918128654971 |
| 0.3600000000000001 | None | 0.012820512820512801 | 0.050314465408805 | 0.010554089709762505 | 0.05074626865671642 | 0.005449591280653953 | None | 0.020408163265306107 | 0.10162601626016303 | 0.12075471698113205 | 0.04819277108433742 | 0.061538461538461514 | 0.1 | 0.03389830508474581 | 0.0649350649350649 | 0.0522388059701493 | 0.07526881720430112 | 0.08737864077669903 | 0.0 | 0.02962962962962961 | 0.0611111111111111 | 0.05521472392638042 | 0.07981220657277002 | 0.10112359550561803 | 0.0954356846473029 | 0.13461538461538505 | 0.12916666666666696 | 0.10344827586206902 | 0.017543859649122813 |
| 0.38000000000000012 | None | 0.02564102564102561 | 0.0440251572327044 | 0.0 | 0.05970149253731343 | 0.002724795640326982 | None | 0.006802721088435372 | 0.08943089430894313 | 0.06415094339622643 | 0.04518072289156631 | 0.07692307692307691 | 0.06000000000000002 | 0.07344632768361581 | 0.07792207792207793 | 0.0447761194029851 | 0.057347670250896134 | 0.048543689320388314 | 0.0 | 0.0148148148148148 | 0.05555555555555558 | 0.05521472392638042 | 0.08450704225352115 | 0.07303370786516852 | 0.03734439834024901 | 0.10096153846153805 | 0.12916666666666696 | 0.10344827586206902 | 0.017543859649122813 |
| 0.4 | None | 0.012820512820512801 | 0.034591194968553514 | 0.0 | 0.0298507462686567 | 0.0 | None | 0.013605442176870696 | 0.06910569105691064 | 0.041509433962264086 | 0.036144578313253 | 0.07692307692307691 | 0.04500000000000001 | 0.11299435028248603 | 0.0649350649350649 | 0.05970149253731343 | 0.043010752688171984 | 0.048543689320388314 | 0.0 | 0.0148148148148148 | 0.0166666666666667 | 0.030674846625766916 | 0.03286384976525821 | 0.06179775280898882 | 0.04149377593361002 | 0.07692307692307691 | 0.108333333333333 | 0.06896551724137931 | 0.0 |
| 0.4200000000000001 | None | 0.0 | 0.0188679245283019 | 0.0 | 0.026865671641791 | 0.0 | None | 0.0 | 0.04065040650406497 | 0.0490566037735849 | 0.0331325301204819 | 0.015384615384615405 | 0.020000000000000007 | 0.096045197740113 | 0.051948051948052 | 0.014925373134328401 | 0.025089605734767 | 0.02265372168284791 | 0.023809523809523808 | 0.0 | 0.02777777777777782 | 0.0184049079754601 | 0.0187793427230047 | 0.03932584269662921 | 0.02904564315352701 | 0.0625 | 0.037500000000000006 | 0.0603448275862069 | 0.00584795321637427 |
| 0.44 | None | 0.012820512820512801 | 0.0157232704402516 | 0.0 | 0.032835820895522415 | 0.0 | None | 0.013605442176870696 | 0.016260162601626 | 0.026415094339622597 | 0.0180722891566265 | 0.0 | 0.014999999999999998 | 0.05084745762711862 | 0.038961038961039 | 0.014925373134328401 | 0.03225806451612901 | 0.00970873786407767 | 0.0 | 0.0 | 0.00555555555555556 | 0.02453987730061352 | 0.009389671361502353 | 0.0449438202247191 | 0.00829875518672199 | 0.05288461538461542 | 0.0416666666666667 | 0.017241379310344803 | 0.00584795321637427 |
| 0.46 | None | 0.012820512820512801 | 0.0 | 0.0 | 0.017910447761194 | 0.0 | None | 0.006802721088435372 | 0.0040650406504065 | 0.0188679245283019 | 0.0391566265060241 | 0.015384615384615405 | 0.010000000000000004 | 0.03389830508474581 | 0.025974025974026007 | 0.0074626865671641816 | 0.021505376344086 | 0.00970873786407767 | 0.023809523809523808 | 0.00740740740740741 | 0.022222222222222202 | 0.0184049079754601 | 0.014084507042253504 | 0.022471910112359623 | 0.024896265560166008 | 0.10096153846153805 | 0.02916666666666669 | 0.0258620689655172 | 0.00584795321637427 |
| 0.48000000000000009 | None | 0.0 | 0.012578616352201295 | 0.0 | 0.00298507462686567 | 0.0 | None | 0.006802721088435372 | 0.0 | 0.0 | 0.015060240963855399 | 0.0 | 0.020000000000000007 | 0.0112994350282486 | 0.0 | 0.0 | 0.0 | 0.0 | 0.0 | 0.00740740740740741 | 0.011111111111111101 | 0.0 | 0.00469483568075117 | 0.005617977528089888 | 0.00829875518672199 | 0.048076923076923114 | 0.00833333333333333 | 0.0258620689655172 | 0.0116959064327485 |
| 0.5 | None | 0.012820512820512801 | 0.0 | 0.0 | 0.00298507462686567 | 0.0 | None | 0.006802721088435372 | 0.0 | 0.0037735849056603826 | 0.006024096385542173 | 0.015384615384615405 | 0.005000000000000002 | 0.016949152542372906 | 0.0 | 0.0298507462686567 | 0.003584229390681001 | 0.00970873786407767 | 0.0 | 0.022222222222222202 | 0.00555555555555556 | 0.0061349693251533735 | 0.00469483568075117 | 0.0112359550561798 | 0.0 | 0.0288461538461538 | 0.00416666666666667 | 0.0 | 0.0 |
| 0.52 | None | 0.0 | 0.0 | 0.0 | 0.005970149253731343 | 0.0 | None | 0.0 | 0.0040650406504065 | 0.0075471698113207504 | 0.006024096385542173 | 0.0 | 0.010000000000000004 | 0.0 | 0.0 | 0.0074626865671641816 | 0.0 | 0.0 | 0.0 | 0.0148148148148148 | 0.011111111111111101 | 0.0184049079754601 | 0.00469483568075117 | 0.022471910112359623 | 0.0 | 0.0240384615384615 | 0.00416666666666667 | 0.0258620689655172 | 0.0 |
| 0.54 | None | 0.02564102564102561 | 0.0 | 0.0 | 0.0 | 0.0 | None | 0.0 | 0.0 | 0.0 | 0.0030120481927710797 | 0.0 | 0.010000000000000004 | 0.0112994350282486 | 0.0 | 0.0074626865671641816 | 0.0 | 0.0 | 0.0 | 0.0 | 0.0 | 0.0 | 0.00469483568075117 | 0.005617977528089888 | 0.0 | 0.004807692307692311 | 0.0 | 0.0 | 0.0 |
| 0.56000000000000005 | None | 0.02564102564102561 | 0.0 | 0.0 | 0.0 | 0.0 | None | 0.0 | 0.0 | 0.0 | 0.0 | 0.0 | 0.0 | 0.0 | 0.012987012987013 | 0.0074626865671641816 | 0.0 | 0.00323624595469256 | 0.0 | 0.0 | 0.0 | 0.0061349693251533735 | 0.00469483568075117 | 0.0 | 0.0 | 0.004807692307692311 | 0.0 | 0.0 | 0.0 |
| 0.58000000000000007 | None | 0.0 | 0.0 | 0.0 | 0.0 | 0.002724795640326982 | None | 0.0 | 0.0 | 0.0 | 0.0 | 0.0 | 0.0 | 0.0 | 0.0 | 0.0 | 0.0 | 0.0 | 0.023809523809523808 | 0.0 | 0.0 | 0.0 | 0.0 | 0.0112359550561798 | 0.0 | 0.009615384615384628 | 0.0 | 0.0 | 0.0 |
| 0.6000000000000002 | None | 0.0 | 0.0 | 0.0 | 0.0 | 0.002724795640326982 | None | 0.0 | 0.0 | 0.0 | 0.0 | 0.0 | 0.0 | 0.0 | 0.012987012987013 | 0.014925373134328401 | 0.0 | 0.0 | 0.0 | 0.0 | 0.0 | 0.0 | 0.0 | 0.0 | 0.0 | 0.004807692307692311 | 0.0 | 0.0 | 0.0 |
### Chart: cat-2 A1
| Category | cat-2(CB1112)_A1_01 | cat-2(CB1112)_A1_02 | cat-2(CB1112)_A1_03 | cat-2(CB1112)_A1_04 | cat-2(CB1112)_A1_05 | cat-2(CB1112)_A1_06 | cat-2(CB1112)_A1_07 | cat-2(CB1112)_A1_08 | cat-2(CB1112)_A1_09 | cat-2(CB1112)_A1_10 | cat-2(CB1112)_A1_11 | cat-2(CB1112)_A1_12 | cat-2(CB1112)_A1_13 | cat-2(CB1112)_A1_14 | cat-2(CB1112)_A1_15 | cat-2(CB1112)_A1_16 | cat-2(CB1112)_A1_17 | cat-2(CB1112)_A1_18 | cat-2(CB1112)_A1_19 | cat-2(CB1112)_A1_20 |
|---|---|---|---|---|---|---|---|---|---|---|---|---|---|---|---|---|---|---|---|---|
| 0 | None | None | None | None | None | None | None | None | None | None | None | None | None | None | None | None | None | None | None | None |
| 2.0000000000000007E-2 | 0.0484652665589661 | 0.0736086175942549 | 0.06240249609984404 | 0.0600315955766193 | 0.0811188811188811 | 0.08115942028985512 | 0.0652503793626707 | 0.06309523809523812 | 0.0664993726474278 | 0.051244509516837476 | 0.06331168831168833 | 0.043046357615893996 | 0.0764119601328904 | 0.0664335664335664 | 0.0746835443037975 | 0.0720338983050847 | 0.048484848484848485 | 0.0785973397823458 | 0.06952662721893493 | 0.05416666666666672 |
| 4.0000000000000015E-2 | 0.038772213247172914 | 0.0736086175942549 | 0.07176287051482062 | 0.04107424960505528 | 0.07132867132867132 | 0.060869565217391314 | 0.042488619119878626 | 0.07142857142857141 | 0.0564617314930991 | 0.06149341142020502 | 0.05681818181818183 | 0.07284768211920532 | 0.0753045404208195 | 0.0541958041958042 | 0.06455696202531652 | 0.05084745762711862 | 0.05050505050505048 | 0.06529625151148732 | 0.05917159763313612 | 0.0569444444444444 |
| 6.0000000000000019E-2 | 0.06300484652665587 | 0.066427289048474 | 0.08580343213728553 | 0.04107424960505528 | 0.0685314685314685 | 0.0463768115942029 | 0.05766312594840674 | 0.061904761904761914 | 0.07904642409033884 | 0.0468521229868228 | 0.0454545454545455 | 0.0612582781456954 | 0.0764119601328904 | 0.057692307692307716 | 0.0772151898734177 | 0.057203389830508516 | 0.0464646464646465 | 0.05804111245465538 | 0.07396449704142012 | 0.08611111111111104 |
| 8.0000000000000029E-2 | 0.061389337641357 | 0.07540394973070023 | 0.04992199687987521 | 0.061611374407582895 | 0.057342657342657324 | 0.06376811594202902 | 0.06676783004552353 | 0.0785714285714286 | 0.08155583437892105 | 0.0439238653001464 | 0.07142857142857141 | 0.0811258278145695 | 0.07862679955703211 | 0.0629370629370629 | 0.06329113924050633 | 0.08474576271186447 | 0.09292929292929297 | 0.09189842805320433 | 0.06360946745562132 | 0.08194444444444443 |
| 0.1 | 0.04684975767366716 | 0.05924596050269298 | 0.06084243369734792 | 0.047393364928909935 | 0.0699300699300699 | 0.0550724637681159 | 0.0652503793626707 | 0.07500000000000001 | 0.07026348808030111 | 0.0468521229868228 | 0.0746753246753247 | 0.08278145695364243 | 0.07973421926910303 | 0.052447552447552385 | 0.07974683544303802 | 0.08474576271186447 | 0.0767676767676768 | 0.0556227327690447 | 0.08431952662721888 | 0.06944444444444443 |
| 0.12000000000000002 | 0.058158319870759284 | 0.055655296229802496 | 0.06240249609984404 | 0.06477093206951033 | 0.0685314685314685 | 0.0666666666666667 | 0.07283763277693482 | 0.08095238095238103 | 0.07904642409033884 | 0.05417276720351392 | 0.08441558441558437 | 0.08443708609271519 | 0.07973421926910303 | 0.0541958041958042 | 0.06835443037974683 | 0.0741525423728814 | 0.0626262626262626 | 0.07013301088270862 | 0.06952662721893493 | 0.05555555555555558 |
| 0.14000000000000001 | 0.0662358642972536 | 0.0538599640933573 | 0.03120124804992202 | 0.05687203791469193 | 0.06713286713286712 | 0.0579710144927536 | 0.051593323216995404 | 0.05119047619047622 | 0.07904642409033884 | 0.06588579795021964 | 0.051948051948052 | 0.04635761589403972 | 0.07751937984496123 | 0.0664335664335664 | 0.07215189873417717 | 0.0656779661016949 | 0.0848484848484849 | 0.059250302297460686 | 0.053254437869822514 | 0.07083333333333333 |
| 0.16 | 0.058158319870759284 | 0.055655296229802496 | 0.0686427457098284 | 0.045813586097946335 | 0.055944055944055895 | 0.06376811594202902 | 0.06069802731411232 | 0.05595238095238104 | 0.06148055207026348 | 0.0439238653001464 | 0.0535714285714286 | 0.0480132450331126 | 0.058693244739756414 | 0.04720279720279722 | 0.06329113924050633 | 0.05084745762711862 | 0.06464646464646463 | 0.060459492140266 | 0.0458579881656805 | 0.07083333333333333 |
| 0.18000000000000005 | 0.0565428109854604 | 0.0610412926391382 | 0.04524180967238686 | 0.05529225908372832 | 0.037762237762237805 | 0.0492753623188406 | 0.04400606980273144 | 0.0464285714285714 | 0.043914680050188226 | 0.055636896046852104 | 0.0438311688311688 | 0.04635761589403972 | 0.04983388704318942 | 0.0594405594405594 | 0.05949367088607593 | 0.03389830508474581 | 0.05050505050505048 | 0.064087061668682 | 0.04881656804733732 | 0.037500000000000006 |
| 0.2 | 0.06300484652665587 | 0.04308797127468581 | 0.048361934477379104 | 0.042654028436018995 | 0.033566433566433594 | 0.06376811594202902 | 0.04097116843702578 | 0.05119047619047622 | 0.040150564617314886 | 0.0468521229868228 | 0.0357142857142857 | 0.04470198675496692 | 0.035437430786268015 | 0.050699300699300696 | 0.0443037974683544 | 0.03389830508474581 | 0.05252525252525248 | 0.050785973397823515 | 0.0458579881656805 | 0.05416666666666672 |
| 0.22 | 0.061389337641357 | 0.039497307001795316 | 0.0405616224648986 | 0.05213270142180092 | 0.0461538461538462 | 0.0434782608695652 | 0.04855842185128982 | 0.0321428571428571 | 0.043914680050188226 | 0.04831625183016114 | 0.042207792207792215 | 0.057947019867549715 | 0.05647840531561458 | 0.02972027972027971 | 0.03544303797468352 | 0.038135593220339 | 0.0383838383838384 | 0.04957678355501812 | 0.053254437869822514 | 0.036111111111111115 |
| 0.24000000000000005 | 0.037156704361874 | 0.04129263913824063 | 0.04524180967238686 | 0.06477093206951033 | 0.0503496503496504 | 0.03188405797101451 | 0.03490136570561461 | 0.048809523809523817 | 0.0363864491844417 | 0.04831625183016114 | 0.05844155844155838 | 0.05298013245033112 | 0.0365448504983389 | 0.043706293706293725 | 0.03544303797468352 | 0.05296610169491532 | 0.05050505050505048 | 0.0350665054413543 | 0.0414201183431953 | 0.0416666666666667 |
| 0.26 | 0.04038772213247173 | 0.035906642728904814 | 0.04992199687987521 | 0.0347551342812006 | 0.02377622377622381 | 0.0434782608695652 | 0.030349013657056112 | 0.0357142857142857 | 0.0326223337515684 | 0.040995607613470014 | 0.0340909090909091 | 0.0397350993377483 | 0.0409745293466224 | 0.050699300699300696 | 0.0405063291139241 | 0.0466101694915254 | 0.0323232323232323 | 0.03990326481257561 | 0.039940828402366915 | 0.02777777777777782 |
| 0.28000000000000008 | 0.021001615508885317 | 0.034111310592459615 | 0.02964118564742592 | 0.037914691943128 | 0.041958041958042015 | 0.03768115942028991 | 0.03641881638846741 | 0.030952380952381 | 0.0225846925972397 | 0.023426061493411397 | 0.030844155844155802 | 0.02483443708609271 | 0.024363233665559206 | 0.026223776223776214 | 0.031645569620253215 | 0.031779661016949214 | 0.024242424242424197 | 0.0145102781136638 | 0.02662721893491121 | 0.02916666666666669 |
| 0.3000000000000001 | 0.024232633279483 | 0.039497307001795316 | 0.02964118564742592 | 0.0347551342812006 | 0.02797202797202801 | 0.02318840579710149 | 0.028831562974203313 | 0.023809523809523808 | 0.0150564617314931 | 0.03660322108345533 | 0.02922077922077921 | 0.036423841059602606 | 0.016611295681063103 | 0.0541958041958042 | 0.025316455696202493 | 0.0105932203389831 | 0.0202020202020202 | 0.024183796856106398 | 0.011834319526627201 | 0.0319444444444444 |
| 0.32000000000000012 | 0.024232633279483 | 0.0215439856373429 | 0.0202808112324493 | 0.028436018957346 | 0.02377622377622381 | 0.0405797101449275 | 0.027314112291350508 | 0.0166666666666667 | 0.025094102885821812 | 0.03660322108345533 | 0.0275974025974026 | 0.016556291390728502 | 0.016611295681063103 | 0.02797202797202801 | 0.010126582278481 | 0.02754237288135592 | 0.0101010101010101 | 0.021765417170495807 | 0.03254437869822492 | 0.0319444444444444 |
| 0.34 | 0.037156704361874 | 0.02333931777378821 | 0.0265210608424337 | 0.018957345971564 | 0.0167832167832168 | 0.0115942028985507 | 0.019726858877086507 | 0.0202380952380952 | 0.023839397741530714 | 0.030746705710102507 | 0.011363636363636404 | 0.02814569536423839 | 0.016611295681063103 | 0.024475524475524518 | 0.0189873417721519 | 0.02118644067796611 | 0.0404040404040404 | 0.0181378476420798 | 0.022189349112426017 | 0.022222222222222202 |
| 0.3600000000000001 | 0.0306946688206785 | 0.0251346499102334 | 0.0202808112324493 | 0.03001579778830962 | 0.025174825174825208 | 0.02318840579710149 | 0.03945371775417303 | 0.0273809523809524 | 0.0301129234629862 | 0.03660322108345533 | 0.017857142857142898 | 0.019867549668874208 | 0.016611295681063103 | 0.033216783216783195 | 0.020253164556962 | 0.019067796610169507 | 0.0323232323232323 | 0.021765417170495807 | 0.03698224852071012 | 0.019444444444444403 |
| 0.38000000000000012 | 0.022617124394184198 | 0.0215439856373429 | 0.018720748829953206 | 0.0205371248025276 | 0.011188811188811204 | 0.008695652173913045 | 0.03945371775417303 | 0.0166666666666667 | 0.0163111668757842 | 0.027818448023426118 | 0.024350649350649397 | 0.0115894039735099 | 0.012181616832779598 | 0.013986013986014 | 0.013924050632911408 | 0.031779661016949214 | 0.0161616161616162 | 0.0145102781136638 | 0.022189349112426017 | 0.022222222222222202 |
| 0.4 | 0.012924071082391 | 0.014362657091561901 | 0.018720748829953206 | 0.02685624012638231 | 0.015384615384615405 | 0.0173913043478261 | 0.0227617602427921 | 0.009523809523809523 | 0.0150564617314931 | 0.023426061493411397 | 0.022727272727272717 | 0.009933774834437097 | 0.006644518272425248 | 0.012237762237762203 | 0.013924050632911408 | 0.016949152542372906 | 0.012121212121212099 | 0.010882708585247904 | 0.016272189349112415 | 0.013888888888888907 |
| 0.4200000000000001 | 0.012924071082391 | 0.008976660682226219 | 0.007800312012480502 | 0.015797788309636705 | 0.00979020979020979 | 0.008695652173913045 | 0.021244309559939313 | 0.011904761904761904 | 0.0112923462986198 | 0.0131771595900439 | 0.014610389610389601 | 0.0049668874172185415 | 0.006644518272425248 | 0.006993006993006992 | 0.011392405063291101 | 0.008474576271186448 | 0.018181818181818205 | 0.009673518742442556 | 0.013313609467455604 | 0.006944444444444443 |
| 0.44 | 0.006462035541195478 | 0.003590664272890482 | 0.010920436817472701 | 0.014218009478672999 | 0.0027972027972028015 | 0.008695652173913045 | 0.009104704097116844 | 0.009523809523809523 | 0.0025094102885821817 | 0.010248901903367504 | 0.006493506493506492 | 0.009933774834437097 | 0.00332225913621262 | 0.008741258741258737 | 0.005063291139240512 | 0.0127118644067797 | 0.00808080808080808 | 0.0036275695284159627 | 0.0044378698224852116 | 0.00416666666666667 |
| 0.46 | 0.009693053311793215 | 0.00538599640933573 | 0.009360374414976605 | 0.015797788309636705 | 0.00559440559440559 | 0.0115942028985507 | 0.007587253414264045 | 0.003571428571428571 | 0.0025094102885821817 | 0.010248901903367504 | 0.0032467532467532517 | 0.0 | 0.00110741971207087 | 0.010489510489510504 | 0.005063291139240512 | 0.004237288135593224 | 0.00404040404040404 | 0.0048367593712212815 | 0.0 | 0.00416666666666667 |
| 0.48000000000000009 | 0.004846526655896612 | 0.007181328545780973 | 0.009360374414976605 | 0.004739336492891005 | 0.006993006993006992 | 0.00579710144927536 | 0.006069802731411232 | 0.0023809523809523807 | 0.0037641154328732717 | 0.007320644216691072 | 0.004870129870129872 | 0.0016556291390728505 | 0.00110741971207087 | 0.0052447552447552484 | 0.005063291139240512 | 0.004237288135593224 | 0.00404040404040404 | 0.0036275695284159627 | 0.005917159763313612 | 0.00416666666666667 |
| 0.5 | 0.0016155088852988701 | 0.003590664272890482 | 0.003120124804992202 | 0.0015797788309636705 | 0.00559440559440559 | 0.0028985507246376808 | 0.006069802731411232 | 0.00595238095238095 | 0.0012547051442910909 | 0.00878477306002928 | 0.008116883116883123 | 0.0033112582781457 | 0.0 | 0.0052447552447552484 | 0.0025316455696202493 | 0.006355932203389832 | 0.00202020202020202 | 0.0048367593712212815 | 0.005917159763313612 | 0.006944444444444443 |
| 0.52 | 0.009693053311793215 | 0.0 | 0.004680187207488298 | 0.006319115323854662 | 0.0027972027972028015 | 0.0 | 0.009104704097116844 | 0.003571428571428571 | 0.0037641154328732717 | 0.0014641288433382106 | 0.0032467532467532517 | 0.0049668874172185415 | 0.00110741971207087 | 0.0017482517482517509 | 0.003797468354430381 | 0.002118644067796611 | 0.00404040404040404 | 0.0048367593712212815 | 0.005917159763313612 | 0.0 |
| 0.54 | 0.0032310177705977415 | 0.003590664272890482 | 0.004680187207488298 | 0.011058451816745701 | 0.0 | 0.008695652173913045 | 0.00455235204855842 | 0.003571428571428571 | 0.00501882057716437 | 0.004392386530014641 | 0.006493506493506492 | 0.006622516556291386 | 0.0 | 0.0017482517482517509 | 0.0 | 0.004237288135593224 | 0.00202020202020202 | 0.00120918984280532 | 0.0044378698224852116 | 0.00416666666666667 |
| 0.56000000000000005 | 0.004846526655896612 | 0.003590664272890482 | 0.0 | 0.004739336492891005 | 0.0013986013986014001 | 0.0 | 0.0015174506828528104 | 0.0 | 0.0 | 0.00585651537335286 | 0.0032467532467532517 | 0.0016556291390728505 | 0.0 | 0.0034965034965035 | 0.00126582278481013 | 0.0 | 0.00202020202020202 | 0.00241837968561064 | 0.0014792899408284004 | 0.00416666666666667 |
| 0.58000000000000007 | 0.0032310177705977415 | 0.001795332136445241 | 0.0 | 0.0015797788309636705 | 0.0027972027972028015 | 0.0028985507246376808 | 0.003034901365705611 | 0.0011904761904761901 | 0.0025094102885821817 | 0.007320644216691072 | 0.0 | 0.0 | 0.0 | 0.0017482517482517509 | 0.0025316455696202493 | 0.0 | 0.0 | 0.00120918984280532 | 0.0029585798816568008 | 0.0027777777777777827 |
| 0.6000000000000002 | 0.0016155088852988701 | 0.0 | 0.0 | 0.0015797788309636705 | 0.0 | 0.0 | 0.0015174506828528104 | 0.0 | 0.0 | 0.0 | 0.0 | 0.0033112582781457 | 0.0 | 0.0 | 0.0 | 0.0 | 0.0 | 0.0 | 0.0 | 0.0 |
### Chart: cat-2 A3
| Category | cat-2(CB1112)_A3_01 | cat-2(CB1112)_A3_02 | cat-2(CB1112)_A3_04 | cat-2(CB1112)_A3_05 | cat-2(CB1112)_A3_06 | cat-2(CB1112)_A3_07 | cat-2(CB1112)_A3_08 | cat-2(CB1112)_A3_09 | cat-2(CB1112)_A3_10 | cat-2(CB1112)_A3_11 | cat-2(CB1112)_A3_12 | cat-2(CB1112)_A3_13 | cat-2(CB1112)_A3_14 | cat-2(CB1112)_A3_15 | cat-2(CB1112)_A3_16 | cat-2(CB1112)_A3_17 | cat-2(CB1112)_A3_18 | cat-2(CB1112)_A3_19 | cat-2(CB1112)_A3_20 |
|---|---|---|---|---|---|---|---|---|---|---|---|---|---|---|---|---|---|---|---|
| 0 | None | None | None | None | None | None | None | None | None | None | None | None | None | None | None | None | None | None | None |
| 2.0000000000000007E-2 | 0.013157894736842101 | 0.0 | 0.009852216748768476 | 0.0 | 0.0 | 0.005025125628140698 | 0.01304347826086961 | 0.008658008658008665 | 0.019011406844106505 | 0.03557312252964431 | 0.026239067055393618 | 0.0 | 0.010489510489510504 | 0.013157894736842101 | 0.0635838150289017 | 0.011406844106463901 | 0.022727272727272717 | 0.047619047619047616 | 0.0398671096345515 |
| 4.0000000000000015E-2 | 0.006578947368421051 | 0.0 | 0.009852216748768476 | 0.004310344827586208 | 0.0 | 0.0 | 0.01304347826086961 | 0.008658008658008665 | 0.022813688212927802 | 0.0158102766798419 | 0.0145772594752187 | 0.0 | 0.006993006993006992 | 0.0 | 0.026011560693641602 | 0.003802281368821292 | 0.019480519480519508 | 0.018518518518518507 | 0.016611295681063103 |
| 6.0000000000000019E-2 | 0.019736842105263205 | 0.005780346820809252 | 0.004926108374384242 | 0.0 | 0.00436681222707424 | 0.0 | 0.01304347826086961 | 0.008658008658008665 | 0.003802281368821292 | 0.0237154150197628 | 0.00291545189504373 | 0.0 | 0.0034965034965035 | 0.0043859649122807 | 0.03179190751445093 | 0.003802281368821292 | 0.022727272727272717 | 0.037037037037037014 | 0.0132890365448505 |
| 8.0000000000000029E-2 | 0.0 | 0.0 | 0.019704433497536908 | 0.0 | 0.0 | 0.0 | 0.00434782608695652 | 0.004329004329004332 | 0.0 | 0.0237154150197628 | 0.00291545189504373 | 0.0 | 0.0 | 0.0043859649122807 | 0.005780346820809252 | 0.0 | 0.0 | 0.02645502645502651 | 0.006644518272425248 |
| 0.1 | 0.006578947368421051 | 0.0 | 0.019704433497536908 | 0.004310344827586208 | 0.00436681222707424 | 0.0 | 0.00434782608695652 | 0.017316017316017306 | 0.011406844106463901 | 0.019762845849802414 | 0.0058309037900874635 | 0.0 | 0.0 | 0.0043859649122807 | 0.02023121387283241 | 0.0 | 0.0 | 0.013227513227513204 | 0.006644518272425248 |
| 0.12000000000000002 | 0.0 | 0.0 | 0.0 | 0.0 | 0.008733624454148473 | 0.005025125628140698 | 0.0 | 0.008658008658008665 | 0.003802281368821292 | 0.00395256916996047 | 0.00291545189504373 | 0.0 | 0.0 | 0.0043859649122807 | 0.017341040462427702 | 0.0 | 0.0 | 0.0343915343915344 | 0.009966777408637873 |
| 0.14000000000000001 | 0.0 | 0.0 | 0.0 | 0.0 | 0.0 | 0.0150753768844221 | 0.00434782608695652 | 0.004329004329004332 | 0.0 | 0.019762845849802414 | 0.0058309037900874635 | 0.00476190476190476 | 0.0 | 0.013157894736842101 | 0.00289017341040462 | 0.0 | 0.0 | 0.02645502645502651 | 0.00332225913621262 |
| 0.16 | 0.006578947368421051 | 0.011560693641618505 | 0.004926108374384242 | 0.004310344827586208 | 0.0 | 0.0 | 0.008695652173913045 | 0.008658008658008665 | 0.011406844106463901 | 0.00790513833992095 | 0.00291545189504373 | 0.0 | 0.0 | 0.0 | 0.00867052023121387 | 0.003802281368821292 | 0.00974025974025974 | 0.02645502645502651 | 0.00332225913621262 |
| 0.18000000000000005 | 0.0 | 0.005780346820809252 | 0.0 | 0.017241379310344803 | 0.0 | 0.0 | 0.0 | 0.004329004329004332 | 0.007604562737642592 | 0.00790513833992095 | 0.008746355685131197 | 0.00476190476190476 | 0.0034965034965035 | 0.013157894736842101 | 0.00289017341040462 | 0.003802281368821292 | 0.006493506493506492 | 0.0343915343915344 | 0.0 |
| 0.2 | 0.0 | 0.05202312138728321 | 0.004926108374384242 | 0.04310344827586208 | 0.008733624454148473 | 0.005025125628140698 | 0.0 | 0.008658008658008665 | 0.003802281368821292 | 0.0237154150197628 | 0.0 | 0.052380952380952396 | 0.017482517482517508 | 0.035087719298245605 | 0.011560693641618505 | 0.0 | 0.0 | 0.013227513227513204 | 0.009966777408637873 |
| 0.22 | 0.013157894736842101 | 0.04624277456647398 | 0.004926108374384242 | 0.05603448275862072 | 0.013100436681222703 | 0.0251256281407035 | 0.00434782608695652 | 0.0346320346320346 | 0.011406844106463901 | 0.00790513833992095 | 0.0145772594752187 | 0.0761904761904762 | 0.0664335664335664 | 0.100877192982456 | 0.011560693641618505 | 0.022813688212927802 | 0.022727272727272717 | 0.023809523809523808 | 0.026578073089701008 |
| 0.24000000000000005 | 0.013157894736842101 | 0.14450867052023106 | 0.0 | 0.112068965517241 | 0.0611353711790393 | 0.07035175879396978 | 0.026086956521739115 | 0.05627705627705628 | 0.026615969581749013 | 0.0158102766798419 | 0.020408163265306107 | 0.15714285714285706 | 0.08391608391608396 | 0.100877192982456 | 0.017341040462427702 | 0.05703422053231942 | 0.07142857142857141 | 0.023809523809523808 | 0.04983388704318942 |
| 0.26 | 0.039473684210526314 | 0.19075144508670505 | 0.0394088669950739 | 0.1810344827586211 | 0.117903930131004 | 0.17587939698492505 | 0.0565217391304348 | 0.09523809523809525 | 0.04942965779467681 | 0.0237154150197628 | 0.032069970845481 | 0.19047619047619 | 0.11888111888111905 | 0.15789473684210512 | 0.04624277456647398 | 0.08365019011406848 | 0.08766233766233769 | 0.05026455026455028 | 0.0764119601328904 |
| 0.28000000000000008 | 0.052631578947368404 | 0.179190751445087 | 0.049261083743842415 | 0.155172413793103 | 0.1572052401746721 | 0.16582914572864293 | 0.0739130434782609 | 0.15151515151515207 | 0.12167300380228104 | 0.08300395256917004 | 0.05830903790087462 | 0.16666666666666693 | 0.13986013986014006 | 0.12280701754385999 | 0.06647398843930642 | 0.15589353612167306 | 0.107142857142857 | 0.0661375661375661 | 0.08970099667774094 |
| 0.3000000000000001 | 0.04605263157894742 | 0.12138728323699402 | 0.07389162561576362 | 0.13793103448275906 | 0.13973799126637607 | 0.17085427135678397 | 0.11304347826087002 | 0.16017316017316 | 0.14448669201520906 | 0.110671936758893 | 0.09329446064139948 | 0.13333333333333305 | 0.192307692307692 | 0.105263157894737 | 0.10115606936416198 | 0.14448669201520906 | 0.08116883116883122 | 0.0661375661375661 | 0.11960132890365405 |
| 0.32000000000000012 | 0.0986842105263158 | 0.08092485549132956 | 0.10344827586206902 | 0.07327586206896546 | 0.10043668122270702 | 0.135678391959799 | 0.16521739130434807 | 0.14285714285714307 | 0.09885931558935367 | 0.05928853754940712 | 0.14868804664723007 | 0.0904761904761905 | 0.125874125874126 | 0.140350877192982 | 0.06936416184971106 | 0.14448669201520906 | 0.09415584415584427 | 0.0740740740740741 | 0.112956810631229 |
| 0.34 | 0.07236842105263165 | 0.08670520231213871 | 0.12807881773398988 | 0.08620689655172414 | 0.11353711790393 | 0.10552763819095498 | 0.130434782608696 | 0.10822510822510806 | 0.0684410646387833 | 0.0790513833992095 | 0.13994169096209907 | 0.08095238095238103 | 0.08391608391608396 | 0.07894736842105257 | 0.11849710982659002 | 0.091254752851711 | 0.09740259740259734 | 0.07142857142857141 | 0.116279069767442 |
| 0.3600000000000001 | 0.0986842105263158 | 0.028901734104046197 | 0.13793103448275906 | 0.03879310344827591 | 0.09606986899563327 | 0.05025125628140698 | 0.08695652173913045 | 0.0692640692640693 | 0.09885931558935367 | 0.08300395256917004 | 0.13119533527696806 | 0.0380952380952381 | 0.0629370629370629 | 0.03070175438596491 | 0.06936416184971106 | 0.11406844106463904 | 0.10064935064935097 | 0.0767195767195767 | 0.08970099667774094 |
| 0.38000000000000012 | 0.0986842105263158 | 0.028901734104046197 | 0.09359605911330056 | 0.03879310344827591 | 0.074235807860262 | 0.0251256281407035 | 0.0826086956521739 | 0.025974025974026007 | 0.10646387832699603 | 0.05928853754940712 | 0.09620991253644313 | 0.00476190476190476 | 0.0314685314685315 | 0.026315789473684202 | 0.08959537572254338 | 0.060836501901140747 | 0.08116883116883122 | 0.0740740740740741 | 0.06644518272425247 |
| 0.4 | 0.125 | 0.005780346820809252 | 0.06896551724137931 | 0.017241379310344803 | 0.0349344978165939 | 0.0201005025125628 | 0.0434782608695652 | 0.021645021645021606 | 0.064638783269962 | 0.110671936758893 | 0.0612244897959184 | 0.0 | 0.02797202797202801 | 0.021929824561403508 | 0.060693641618497114 | 0.030418250950570307 | 0.0454545454545455 | 0.05555555555555558 | 0.04651162790697674 |
| 0.4200000000000001 | 0.07894736842105257 | 0.005780346820809252 | 0.05418719211822662 | 0.004310344827586208 | 0.03056768558951961 | 0.005025125628140698 | 0.0434782608695652 | 0.017316017316017306 | 0.026615969581749013 | 0.06324110671936756 | 0.03498542274052481 | 0.0 | 0.010489510489510504 | 0.0087719298245614 | 0.04335260115606942 | 0.015209125475285206 | 0.0357142857142857 | 0.04232804232804231 | 0.03322259136212619 |
| 0.44 | 0.04605263157894742 | 0.0 | 0.0394088669950739 | 0.0 | 0.0 | 0.0100502512562814 | 0.0173913043478261 | 0.008658008658008665 | 0.011406844106463901 | 0.0118577075098814 | 0.0233236151603499 | 0.0 | 0.006993006993006992 | 0.0 | 0.014450867052023099 | 0.011406844106463901 | 0.0324675324675325 | 0.018518518518518507 | 0.0232558139534884 |
| 0.46 | 0.052631578947368404 | 0.005780346820809252 | 0.029556650246305397 | 0.004310344827586208 | 0.013100436681222703 | 0.0 | 0.0217391304347826 | 0.0 | 0.019011406844106505 | 0.0237154150197628 | 0.017492711370262405 | 0.0 | 0.0 | 0.0 | 0.017341040462427702 | 0.015209125475285206 | 0.006493506493506492 | 0.013227513227513204 | 0.0 |
| 0.48000000000000009 | 0.03289473684210532 | 0.0 | 0.029556650246305397 | 0.008620689655172415 | 0.0 | 0.0 | 0.008695652173913045 | 0.0 | 0.003802281368821292 | 0.0158102766798419 | 0.017492711370262405 | 0.0 | 0.0034965034965035 | 0.0043859649122807 | 0.026011560693641602 | 0.0 | 0.00974025974025974 | 0.013227513227513204 | 0.0 |
| 0.5 | 0.019736842105263205 | 0.0 | 0.009852216748768476 | 0.0 | 0.00436681222707424 | 0.005025125628140698 | 0.01304347826086961 | 0.0 | 0.0 | 0.0118577075098814 | 0.008746355685131197 | 0.0 | 0.0 | 0.0 | 0.014450867052023099 | 0.003802281368821292 | 0.006493506493506492 | 0.007936507936507941 | 0.0 |
| 0.52 | 0.013157894736842101 | 0.0 | 0.0147783251231527 | 0.0 | 0.00436681222707424 | 0.0 | 0.01304347826086961 | 0.0 | 0.015209125475285206 | 0.00395256916996047 | 0.00291545189504373 | 0.0 | 0.0 | 0.0 | 0.005780346820809252 | 0.0 | 0.0032467532467532517 | 0.002645502645502652 | 0.0 |
| 0.54 | 0.013157894736842101 | 0.0 | 0.0 | 0.004310344827586208 | 0.0 | 0.0 | 0.00434782608695652 | 0.0 | 0.0 | 0.0 | 0.0 | 0.0 | 0.0 | 0.0 | 0.0 | 0.0 | 0.0 | 0.002645502645502652 | 0.0 |
| 0.56000000000000005 | 0.0 | 0.0 | 0.0147783251231527 | 0.0 | 0.0 | 0.0 | 0.0 | 0.0 | 0.0 | 0.00395256916996047 | 0.0 | 0.0 | 0.0 | 0.0 | 0.0 | 0.0 | 0.0 | 0.007936507936507941 | 0.0 |
| 0.58000000000000007 | 0.013157894736842101 | 0.0 | 0.009852216748768476 | 0.0 | 0.0 | 0.0 | 0.0 | 0.0 | 0.0 | 0.0 | 0.00291545189504373 | 0.0 | 0.0 | 0.0 | 0.00289017341040462 | 0.003802281368821292 | 0.0 | 0.0 | 0.0 |
| 0.6000000000000002 | 0.013157894736842101 | 0.0 | 0.0 | 0.0 | 0.0 | 0.0 | 0.0 | 0.0 | 0.0 | 0.00395256916996047 | 0.00291545189504373 | 0.0 | 0.0 | 0.0 | 0.0 | 0.0 | 0.0 | 0.0 | 0.0 |
### Chart: cat-2 A5
| Category | cat-2(CB1112)_A5_01 | cat-2(CB1112)_A5_02 | cat-2(CB1112)_A5_03 | cat-2(CB1112)_A5_04 | cat-2(CB1112)_A5_05 | cat-2(CB1112)_A5_06 | cat-2(CB1112)_A5_07 | cat-2(CB1112)_A5_08 | cat-2(CB1112)_A5_09 | cat-2(CB1112)_A5_10 | cat-2(CB1112)_A5_11 | cat-2(CB1112)_A5_12 | cat-2(CB1112)_A5_13 | cat-2(CB1112)_A5_14 | cat-2(CB1112)_A5_15 | cat-2(CB1112)_A5_16 | cat-2(CB1112)_A5_17 | cat-2(CB1112)_A5_18 | cat-2(CB1112)_A5_19 | cat-2(CB1112)_A5_20 |
|---|---|---|---|---|---|---|---|---|---|---|---|---|---|---|---|---|---|---|---|---|
| 0 | None | None | None | None | None | None | None | None | None | None | None | None | None | None | None | None | None | None | None | None |
| 2.0000000000000007E-2 | 0.0231660231660232 | 0.016949152542372906 | 0.0438247011952191 | 0.016042780748663114 | 0.00555555555555556 | 0.017937219730941697 | 0.0112359550561798 | 0.005747126436781612 | 0.022222222222222202 | 0.013452914798206301 | 0.0 | 0.016949152542372906 | 0.013392857142857107 | 0.00448430493273543 | 0.0 | 0.096045197740113 | 0.016042780748663114 | 0.029787234042553207 | 0.0719101123595506 | 0.013761467889908304 |
| 4.0000000000000015E-2 | 0.007722007722007722 | 0.00564971751412429 | 0.027888446215139407 | 0.021390374331550797 | 0.00555555555555556 | 0.0 | 0.0112359550561798 | 0.0114942528735632 | 0.0 | 0.0 | 0.012461059190031204 | 0.0 | 0.00446428571428571 | 0.0 | 0.0 | 0.039548022598870115 | 0.016042780748663114 | 0.0212765957446809 | 0.05842696629213481 | 0.00917431192660551 |
| 6.0000000000000019E-2 | 0.0038610038610038602 | 0.0 | 0.0159362549800797 | 0.0053475935828877 | 0.00555555555555556 | 0.00896860986547085 | 0.022471910112359623 | 0.005747126436781612 | 0.013333333333333301 | 0.00448430493273543 | 0.012461059190031204 | 0.016949152542372906 | 0.0 | 0.0 | 0.0 | 0.0564971751412429 | 0.0053475935828877 | 0.008510638297872348 | 0.0449438202247191 | 0.0 |
| 8.0000000000000029E-2 | 0.0038610038610038602 | 0.0225988700564972 | 0.0199203187250996 | 0.0053475935828877 | 0.011111111111111101 | 0.00448430493273543 | 0.022471910112359623 | 0.0 | 0.0 | 0.00448430493273543 | 0.018691588785046703 | 0.0 | 0.008928571428571423 | 0.00448430493273543 | 0.0114942528735632 | 0.03389830508474581 | 0.0106951871657754 | 0.0212765957446809 | 0.0382022471910112 | 0.0 |
| 0.1 | 0.015444015444015408 | 0.0 | 0.027888446215139407 | 0.0106951871657754 | 0.00555555555555556 | 0.0 | 0.0112359550561798 | 0.0114942528735632 | 0.0 | 0.0 | 0.0031152647975077907 | 0.00564971751412429 | 0.00446428571428571 | 0.0 | 0.022988505747126402 | 0.0451977401129944 | 0.016042780748663114 | 0.012765957446808501 | 0.051685393258427 | 0.0 |
| 0.12000000000000002 | 0.019305019305019308 | 0.0 | 0.011952191235059804 | 0.0 | 0.0 | 0.00448430493273543 | 0.0112359550561798 | 0.0 | 0.008888888888888894 | 0.00448430493273543 | 0.021806853582554513 | 0.016949152542372906 | 0.0 | 0.013452914798206301 | 0.0 | 0.03389830508474581 | 0.016042780748663114 | 0.012765957446808501 | 0.06966292134831466 | 0.00917431192660551 |
| 0.14000000000000001 | 0.015444015444015408 | 0.0 | 0.0199203187250996 | 0.0053475935828877 | 0.0 | 0.00896860986547085 | 0.0112359550561798 | 0.0114942528735632 | 0.013333333333333301 | 0.026905829596412602 | 0.0249221183800623 | 0.00564971751412429 | 0.0 | 0.0 | 0.0 | 0.0225988700564972 | 0.0106951871657754 | 0.004255319148936172 | 0.05842696629213481 | 0.00917431192660551 |
| 0.16 | 0.019305019305019308 | 0.0 | 0.0159362549800797 | 0.026737967914438505 | 0.00555555555555556 | 0.013452914798206301 | 0.0 | 0.022988505747126402 | 0.0 | 0.017937219730941697 | 0.009345794392523367 | 0.00564971751412429 | 0.00446428571428571 | 0.00448430493273543 | 0.0 | 0.0112994350282486 | 0.0106951871657754 | 0.0 | 0.051685393258427 | 0.0 |
| 0.18000000000000005 | 0.0038610038610038602 | 0.00564971751412429 | 0.003984063745019922 | 0.0 | 0.0 | 0.013452914798206301 | 0.0 | 0.017241379310344803 | 0.008888888888888894 | 0.022421524663677108 | 0.009345794392523367 | 0.0 | 0.008928571428571423 | 0.00448430493273543 | 0.0 | 0.02824858757062151 | 0.021390374331550797 | 0.004255319148936172 | 0.04943820224719101 | 0.00458715596330275 |
| 0.2 | 0.0038610038610038602 | 0.0 | 0.0159362549800797 | 0.0053475935828877 | 0.00555555555555556 | 0.00896860986547085 | 0.03370786516853931 | 0.022988505747126402 | 0.013333333333333301 | 0.013452914798206301 | 0.0 | 0.016949152542372906 | 0.008928571428571423 | 0.00448430493273543 | 0.0 | 0.0225988700564972 | 0.0106951871657754 | 0.004255319148936172 | 0.047191011235955115 | 0.00917431192660551 |
| 0.22 | 0.007722007722007722 | 0.00564971751412429 | 0.03187250996015941 | 0.0053475935828877 | 0.011111111111111101 | 0.049327354260089704 | 0.06741573033707873 | 0.022988505747126402 | 0.008888888888888894 | 0.0 | 0.012461059190031204 | 0.02824858757062151 | 0.008928571428571423 | 0.0448430493273543 | 0.0 | 0.02824858757062151 | 0.0106951871657754 | 0.004255319148936172 | 0.0539325842696629 | 0.00917431192660551 |
| 0.24000000000000005 | 0.0038610038610038602 | 0.016949152542372906 | 0.039840637450199216 | 0.026737967914438505 | 0.0166666666666667 | 0.049327354260089704 | 0.0449438202247191 | 0.0402298850574713 | 0.05333333333333334 | 0.0 | 0.006230529595015582 | 0.039548022598870115 | 0.044642857142857095 | 0.053811659192825115 | 0.0 | 0.02824858757062151 | 0.032085561497326213 | 0.0340425531914894 | 0.06067415730337078 | 0.00458715596330275 |
| 0.26 | 0.0347490347490347 | 0.016949152542372906 | 0.05976095617529882 | 0.053475935828877004 | 0.02777777777777782 | 0.0762331838565022 | 0.157303370786517 | 0.08620689655172414 | 0.05333333333333334 | 0.026905829596412602 | 0.0654205607476635 | 0.0903954802259887 | 0.0580357142857143 | 0.0762331838565022 | 0.03448275862068972 | 0.0621468926553672 | 0.03743315508021393 | 0.0340425531914894 | 0.0382022471910112 | 0.03211009174311931 |
| 0.28000000000000008 | 0.0308880308880309 | 0.03389830508474581 | 0.07569721115537853 | 0.0641711229946524 | 0.0666666666666667 | 0.0762331838565022 | 0.0786516853932584 | 0.09195402298850573 | 0.12000000000000002 | 0.06278026905829605 | 0.115264797507788 | 0.129943502824859 | 0.10267857142857104 | 0.13452914798206306 | 0.0804597701149425 | 0.02824858757062151 | 0.07486631016042783 | 0.0723404255319149 | 0.0539325842696629 | 0.03669724770642203 |
| 0.3000000000000001 | 0.07335907335907342 | 0.08474576271186447 | 0.06374501992031872 | 0.0695187165775401 | 0.0666666666666667 | 0.10762331838565004 | 0.0898876404494382 | 0.10344827586206902 | 0.0933333333333333 | 0.09865470852017949 | 0.10903426791277303 | 0.19209039548022608 | 0.16071428571428606 | 0.15246636771300406 | 0.05747126436781611 | 0.0112994350282486 | 0.128342245989305 | 0.08085106382978723 | 0.04943820224719101 | 0.09174311926605508 |
| 0.32000000000000012 | 0.131274131274131 | 0.12429378531073405 | 0.10358565737051803 | 0.09625668449197858 | 0.1444444444444441 | 0.13004484304932706 | 0.11235955056179797 | 0.10344827586206902 | 0.10222222222222209 | 0.0762331838565022 | 0.102803738317757 | 0.13559322033898297 | 0.14285714285714307 | 0.13452914798206306 | 0.14942528735632213 | 0.0451977401129944 | 0.15508021390374294 | 0.1276595744680849 | 0.0404494382022472 | 0.11467889908256895 |
| 0.34 | 0.10424710424710402 | 0.07344632768361581 | 0.10756972111553803 | 0.10695187165775398 | 0.155555555555556 | 0.12107623318385703 | 0.11235955056179797 | 0.08620689655172414 | 0.12000000000000002 | 0.11659192825112104 | 0.12461059190031203 | 0.10169491525423703 | 0.125 | 0.12107623318385703 | 0.13793103448275906 | 0.02824858757062151 | 0.08021390374331548 | 0.1319148936170211 | 0.0314606741573034 | 0.11926605504587204 |
| 0.3600000000000001 | 0.10038610038610005 | 0.11864406779661005 | 0.07569721115537853 | 0.128342245989305 | 0.111111111111111 | 0.0852017937219731 | 0.0449438202247191 | 0.08620689655172414 | 0.0666666666666667 | 0.10313901345291503 | 0.0965732087227414 | 0.06779661016949155 | 0.111607142857143 | 0.0762331838565022 | 0.12643678160919505 | 0.0225988700564972 | 0.0641711229946524 | 0.08510638297872347 | 0.0157303370786517 | 0.123853211009174 |
| 0.38000000000000012 | 0.08494208494208488 | 0.13559322033898297 | 0.047808764940239036 | 0.08556149732620325 | 0.11666666666666703 | 0.06726457399103143 | 0.0449438202247191 | 0.0402298850574713 | 0.08888888888888893 | 0.11659192825112104 | 0.08099688473520254 | 0.02824858757062151 | 0.07142857142857141 | 0.0448430493273543 | 0.11494252873563203 | 0.016949152542372906 | 0.053475935828877004 | 0.08510638297872347 | 0.0247191011235955 | 0.123853211009174 |
| 0.4 | 0.0888030888030888 | 0.06779661016949155 | 0.055776892430278904 | 0.10695187165775398 | 0.0777777777777778 | 0.022421524663677108 | 0.0449438202247191 | 0.06896551724137931 | 0.07111111111111111 | 0.08071748878923773 | 0.05295950155763241 | 0.03389830508474581 | 0.03125 | 0.0582959641255605 | 0.09195402298850573 | 0.0112994350282486 | 0.053475935828877004 | 0.0553191489361702 | 0.017977528089887607 | 0.08256880733944956 |
| 0.4200000000000001 | 0.0501930501930502 | 0.08474576271186447 | 0.055776892430278904 | 0.026737967914438505 | 0.0388888888888889 | 0.026905829596412602 | 0.0112359550561798 | 0.005747126436781612 | 0.031111111111111114 | 0.0448430493273543 | 0.05295950155763241 | 0.00564971751412429 | 0.013392857142857107 | 0.013452914798206301 | 0.04597701149425289 | 0.02824858757062151 | 0.0588235294117647 | 0.0723404255319149 | 0.0112359550561798 | 0.04128440366972478 |
| 0.44 | 0.042471042471042476 | 0.06779661016949155 | 0.011952191235059804 | 0.03743315508021393 | 0.02777777777777782 | 0.017937219730941697 | 0.022471910112359623 | 0.022988505747126402 | 0.031111111111111114 | 0.04035874439461881 | 0.0249221183800623 | 0.0112994350282486 | 0.017857142857142898 | 0.00448430493273543 | 0.022988505747126402 | 0.0225988700564972 | 0.03743315508021393 | 0.025531914893617006 | 0.0022471910112359626 | 0.05045871559633031 |
| 0.46 | 0.0308880308880309 | 0.0225988700564972 | 0.03585657370517932 | 0.016042780748663114 | 0.022222222222222202 | 0.013452914798206301 | 0.0 | 0.028735632183908007 | 0.008888888888888894 | 0.017937219730941697 | 0.009345794392523367 | 0.0112994350282486 | 0.017857142857142898 | 0.00896860986547085 | 0.022988505747126402 | 0.02824858757062151 | 0.0053475935828877 | 0.004255319148936172 | 0.0 | 0.027522935779816515 |
| 0.48000000000000009 | 0.027027027027027015 | 0.02824858757062151 | 0.007968127490039844 | 0.016042780748663114 | 0.011111111111111101 | 0.017937219730941697 | 0.0 | 0.028735632183908007 | 0.022222222222222202 | 0.017937219730941697 | 0.012461059190031204 | 0.0 | 0.00446428571428571 | 0.00448430493273543 | 0.022988505747126402 | 0.0225988700564972 | 0.0053475935828877 | 0.0170212765957447 | 0.0022471910112359626 | 0.018348623853211 |
| 0.5 | 0.0347490347490347 | 0.02824858757062151 | 0.007968127490039844 | 0.0106951871657754 | 0.0166666666666667 | 0.00448430493273543 | 0.0112359550561798 | 0.005747126436781612 | 0.0 | 0.00448430493273543 | 0.0031152647975077907 | 0.0 | 0.00446428571428571 | 0.00448430493273543 | 0.0114942528735632 | 0.016949152542372906 | 0.0106951871657754 | 0.004255319148936172 | 0.0 | 0.00917431192660551 |
| 0.52 | 0.007722007722007722 | 0.00564971751412429 | 0.0 | 0.0106951871657754 | 0.011111111111111101 | 0.0 | 0.0 | 0.0114942528735632 | 0.022222222222222202 | 0.022421524663677108 | 0.0031152647975077907 | 0.00564971751412429 | 0.0 | 0.00448430493273543 | 0.0114942528735632 | 0.00564971751412429 | 0.0053475935828877 | 0.0 | 0.0022471910112359626 | 0.00458715596330275 |
| 0.54 | 0.015444015444015408 | 0.00564971751412429 | 0.0 | 0.0053475935828877 | 0.00555555555555556 | 0.00448430493273543 | 0.0 | 0.0 | 0.0 | 0.00448430493273543 | 0.006230529595015582 | 0.0 | 0.00446428571428571 | 0.0 | 0.0114942528735632 | 0.0112994350282486 | 0.0 | 0.0 | 0.0 | 0.00458715596330275 |
| 0.56000000000000005 | 0.0038610038610038602 | 0.0 | 0.0 | 0.0 | 0.00555555555555556 | 0.0 | 0.0 | 0.0 | 0.004444444444444442 | 0.00448430493273543 | 0.0 | 0.0 | 0.0 | 0.0 | 0.0 | 0.00564971751412429 | 0.0 | 0.004255319148936172 | 0.0 | 0.00917431192660551 |
| 0.58000000000000007 | 0.0038610038610038602 | 0.0112994350282486 | 0.0 | 0.0106951871657754 | 0.011111111111111101 | 0.0 | 0.0 | 0.0 | 0.0 | 0.00448430493273543 | 0.0 | 0.00564971751412429 | 0.0 | 0.0 | 0.0 | 0.0 | 0.0053475935828877 | 0.0 | 0.0022471910112359626 | 0.00458715596330275 |
| 0.6000000000000002 | 0.0 | 0.0 | 0.0 | 0.0 | 0.0 | 0.0 | 0.0 | 0.0 | 0.0 | 0.0 | 0.0 | 0.0 | 0.0 | 0.00448430493273543 | 0.0 | 0.0112994350282486 | 0.0 | 0.0 | 0.0 | 0.00458715596330275 |
