# Supplementary figures and images for: Evaluation of Head Movement Periodicity and Irregularity during Locomotion of Caenorhabditis elegans
Source: Front Behav Neurosci. 2013 Mar 21;7:20. doi: 10.3389/fnbeh.2013.00020 (PMC3604732; doi:10.3389/fnbeh.2013.00020)

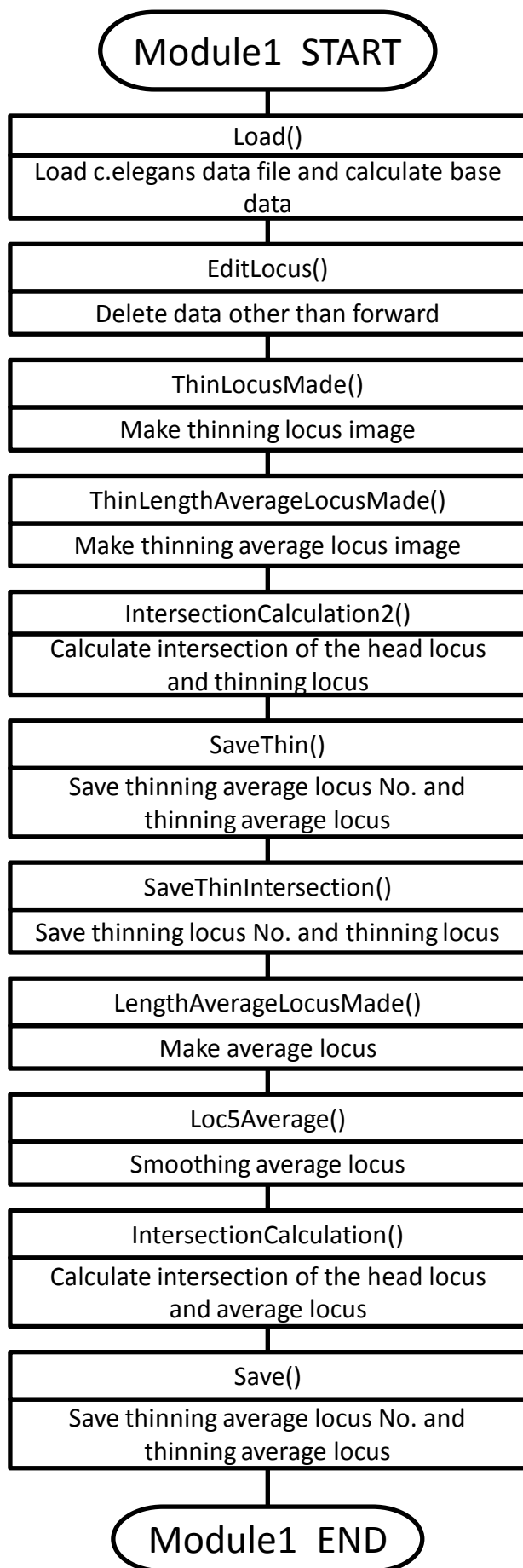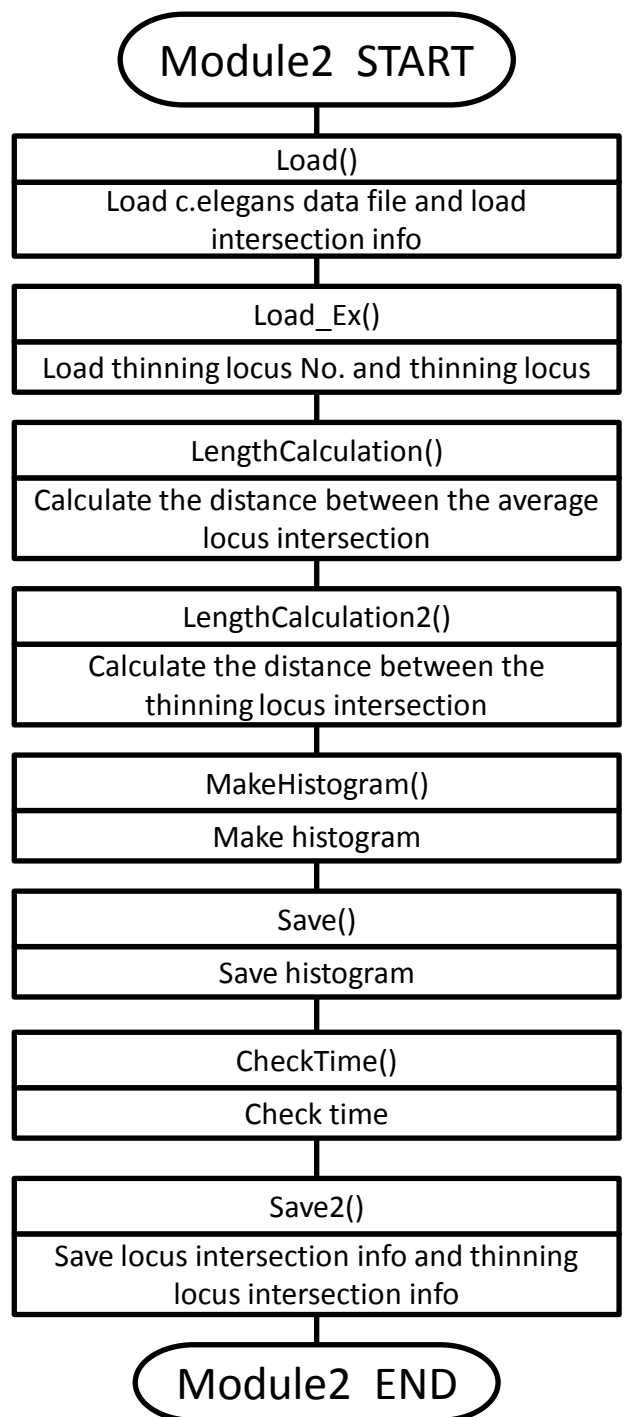

Outline of the program (Type-1 segment-length)

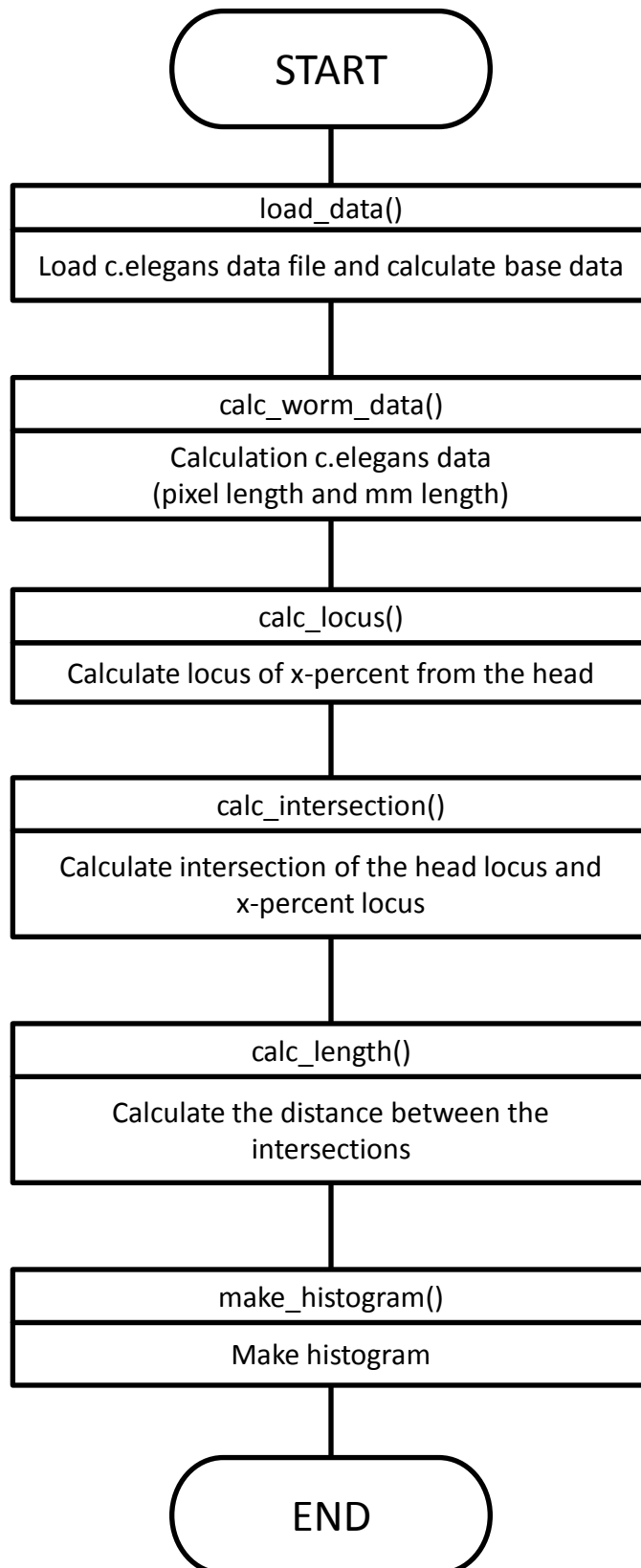

Outline of the program (Type-2 segment-length)

Supplement: Supplementary Software S1 — Outline of program. [file 42064_Shingai_DataSheet1.PDF]

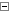

Supplement: Supplementary Software S3 — Computer programs for Type-1 segment-lengths. [file 42064_Shingai_DataSheet3.ZIP › type1/_UpgradeReport_Files/UpgradeReport_Minus.gif]

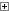

Supplement: Supplementary Software S3 — Computer programs for Type-1 segment-lengths. [file 42064_Shingai_DataSheet3.ZIP › type1/_UpgradeReport_Files/UpgradeReport_Plus.gif]
